# Sex differences in the effects of prenatal bisphenol A exposure on autism-related genes and their relationships with the hippocampus functions

**DOI:** 10.1038/s41598-020-80390-2

**Published:** 2021-01-13

**Authors:** Surangrat Thongkorn, Songphon Kanlayaprasit, Pawinee Panjabud, Thanit Saeliw, Thanawin Jantheang, Kasidit Kasitipradit, Suthathip Sarobol, Depicha Jindatip, Valerie W. Hu, Tewin Tencomnao, Takako Kikkawa, Tatsuya Sato, Noriko Osumi, Tewarit Sarachana

**Affiliations:** 1 The Ph.D. Program in Clinical Biochemistry and Molecular Medicine, Department of Clinical Chemistry, Faculty of Allied Health Sciences, Chulalongkorn University, Bangkok, Thailand; 2 Specimen Center, Department of Laboratory Medicine, King Chulalongkorn Memorial Hospital, Bangkok, Thailand; 3 Department of Anatomy, Faculty of Medicine, Chulalongkorn University, Bangkok, Thailand; 4 SYstems Neuroscience of Autism and PSychiatric Disorders (SYNAPS) Research Unit, Department of Clinical Chemistry, Faculty of Allied Health Sciences, Chulalongkorn University, Bangkok, Thailand; 5 Department of Biochemistry and Molecular Medicine, The George Washington University School of Medicine and Health Sciences, The George Washington University, Washington, DC USA; 6 Age-Related Inflammation and Degeneration Research Unit, Department of Clinical Chemistry, Faculty of Allied Health Sciences, Chulalongkorn University, Bangkok, Thailand; 7 Department of Developmental Neuroscience, United Centers for Advanced Research and Translational Medicine (ART), Tohoku University Graduate School of Medicine, Sendai, Miyagi Japan; 8 Department of Healthcare Management, Faculty of Health Sciences, Tohoku Fukushi University, Sendai, Miyagi Japan

**Keywords:** Chemical biology, RNA, Cellular neuroscience, Molecular neuroscience

## Abstract

Our recent study has shown that prenatal exposure to bisphenol A (BPA) altered the expression of genes associated with autism spectrum disorder (ASD). In this study, we further investigated the effects of prenatal BPA exposure on ASD-related genes known to regulate neuronal viability, neuritogenesis, and learning/memory, and assessed these functions in the offspring of exposed pregnant rats. We found that prenatal BPA exposure increased neurite length, the number of primary neurites, and the number of neurite branches, but reduced the size of the hippocampal cell body in both sexes of the offspring. However, in utero exposure to BPA decreased the neuronal viability and the neuronal density in the hippocampus and impaired learning/memory only in the male offspring while the females were not affected. Interestingly, the expression of several ASD-related genes (e.g. *Mief2*, *Eif3h*, *Cux1*, and *Atp8a1*) in the hippocampus were dysregulated and showed a sex-specific correlation with neuronal viability, neuritogenesis, and/or learning/memory. The findings from this study suggest that prenatal BPA exposure disrupts ASD-related genes involved in neuronal viability, neuritogenesis, and learning/memory in a sex-dependent manner, and these genes may play an important role in the risk and the higher prevalence of ASD in males subjected to prenatal BPA exposure.

## Introduction

Bisphenol A (BPA) is an endocrine-disrupting chemical found in polycarbonate plastic products, linings inside food cans, epoxy resins, thermal receipts, and micro/nanoplastics^1–5^. Once exposed to a high temperature or basic/acid conditions, BPA can leach out from these products or micro/nanoplastics, and contaminate food or pollute the environment^6–8^. Recent studies have shown that humans are widely exposed to BPA^9,10^. Several studies in rodents and human tissues have shown that BPA can circulate throughout the body and readily transfer across the placenta^11–13^ and the blood–brain barrier^14,15^. Calafat and colleagues have measured the urinary concentration of BPA in more than 2500 participants who were older than 6-years-old and found that BPA was detectable in as much as 92.6% of the population^9^. BPA was also detectable in the human placenta, cord serum, neonatal urine, maternal urine, maternal serum, and breast milk^16^.

BPA is thought to be an environmental risk factor for neurological disorders, including autism spectrum disorder (ASD)^17–21^, Alzheimer’s disease^22^, and others^23,24^. Autism spectrum disorder (ASD) is a neurodevelopmental disorder characterized by (1) deficits in social interaction and communication, and (2) restricted interests and repetitive/stereotyped behaviors, with high heterogeneity of clinical manifestations and molecular signatures^25–30^. ASD occurs in all racial, ethnic, and socioeconomic groups, with the prevalence of 1 in 54 children in the United States according to the Autism and Developmental Disabilities Monitoring (ADDM) Network, the Centers for Disease Control and Prevention (CDC)^31^. ASD is at least four times more common in males than in females. The exact causes and the molecular mechanisms underlying the male bias of ASD remain unclear but are thought to involve both genetic and environmental factors^32–36^, as well as epigenetic regulatory mechanisms, including DNA methylation^37,38^, histone modifications^39,40^, and RNA-associated mechanisms^41^. Environmental pollutants that have been associated with increased susceptibility of ASD include BPA^17,18,42^, phthalates^43,44^, pesticides^42,45^, cigarette smoke^46^, lead^47^, and mercury^48^. Elevated levels of BPA have been reported in the blood and urine of children with ASD^19–21^. Kondolot et al. found that plasma BPA levels of children with pervasive developmental disorder not otherwise specified (PDD-NOS) which is a subtype of ASD were significantly higher than age-/sex-matched typically developing children^20^. Consistently, Kardas et al. reported that children with autism spectrum disorder had significantly increased serum BPA concentrations compared to unaffected individuals^19^.

BPA altered gene expression profiles in various brain regions of prenatally exposed rodent offspring, including the hippocampus^17,49^, hypothalamus^49^, cerebellum^50^, and prefrontal cortex^51^. Arambula et al. investigated the effects of prenatal BPA exposure on the hippocampal and hypothalamic transcriptome of the offspring using RNA-seq and qRT-PCR analyses. They found that BPA induced sex-specific effects on hypothalamic *ERα* and *ERβ* (*Esr1* and *Esr2*) expression and hippocampal and hypothalamic oxytocin (*Oxt*) expression^49^. It is noteworthy that these genes have been implicated in ASD susceptibility and the male bias of ASD^52,53^. In addition, we have recently performed a transcriptome profiling analysis of hippocampal tissues isolated from neonatal pups prenatally exposed to BPA or vehicle control using the RNA-seq technique^17^. We found that as many as 2078 genes and 3522 genes were significantly differentially expressed in the hippocampus of BPA-treated male and female pups, respectively, compared to controls. These genes were predicted to be significantly associated with ASD, and the differentially expressed genes in male hippocampal tissues tended to exhibit stronger associations with ASD genes than those in female tissues. The reanalysis of transcriptome profiling data from previously published BPA studies also showed that BPA-responsive genes were significantly associated with ASD-related genes^17^. These findings strongly support that prenatal BPA exposure may increase the risk and involve in the male bias of ASD.

There is accumulating evidence that exposure to BPA impaired neurological functions and behaviors, including synaptogenesis^54^, neuronal differentiation^55^, social interaction^56^, anxiety^57^, and learning/memory^58–62^, all of which have been associated with ASD^63–65^. Zhang et al. reported that postnatal exposure to BPA decreased dendritic spine density in the hippocampus and impaired spatial learning and memory ability in rats^61^. The same group of researchers also investigated the effects of maternal exposure to BPA on the offspring by treating pregnant mice with BPA daily until weaning^62^. Similarly, they found that maternal BPA exposure reduced neuron quantities and spine densities in the hippocampus of offspring, and impaired the learning and memory ability of male offspring. Although these studies provided supporting evidence that BPA exposure can disrupt neurological functions and behaviors related to ASD, the link between the dysregulations of ASD-related genes and the altered neurological functions resulting from BPA exposure has never been investigated. Moreover, since most studies that investigated the effects of maternal exposure to BPA would treat the animals from the gestational period to weaning, it is difficult to distinguish between the effects of prenatal and postnatal exposure to BPA on the offspring brain. Inasmuch as ASD is known to be an early-onset disorder and its pathobiology is thought to occur as early as in the embryonic stage^66,67^, it is important to focus on the prenatal effects to understand the role of BPA in ASD susceptibility.

In this study, we sought to investigate the effects of prenatal exposure to BPA on hippocampal cell viability, neuritogenesis, and learning/memory, and also identify BPA-responsive genes that are associated with those functions and ASD. First, we obtained the transcriptome data of hippocampal tissues isolated from rat offspring prenatally exposed to BPA or control and reanalyzed the data to identify genes that were dysregulated in response to BPA and significantly associated with neuronal viability, neuritogenesis, and learning/memory. Those genes were then selected for further confirmation by qRT-PCR analysis. To investigate the effects of direct BPA exposure on hippocampal cell viability, in vitro cell viability assays were performed using primary rat hippocampal cells treated with BPA or vehicle control in culture. Moreover, in vivo assays were also performed to confirm the effects of prenatal BPA exposure on the viability of hippocampal neurons using confocal immunofluorescence microscopy analysis of hippocampal tissues from rat offspring prenatally exposed to BPA or vehicle control. To investigate the effects of prenatal BPA exposure on neuritogenesis, neurite formation assays were performed using primary hippocampal cells isolated from the rat offspring that were prenatally exposed to BPA or vehicle control. The T-maze and novel object recognition tests were conducted to assess the learning and memory ability of rat offspring. The correlation analyses between the expression levels of BPA-responsive genes which are also ASD candidate genes and the neurological functions were performed to investigate the relationships between those genes and the neurological functions altered by prenatal BPA exposure.

## Results

### Prenatal BPA exposure altered the expression of genes associated with neuronal viability, neuritogenesis, and learning/memory in the hippocampus

Our previous study has already shown that maternal BPA exposure dysregulated transcriptome profiles of ASD-related genes in the offspring hippocampus^17^, but the effects of prenatal exposure to BPA on neurological functions and behaviors associated with the brain region are still unclear. To select neurological functions and behaviors impacted by prenatal BPA exposure for further investigations, we reanalyzed our previously published transcriptome profiling data. First, we obtained the lists of BPA-responsive genes from our previous RNA-seq analysis of hippocampal tissues isolated from neonatal rats prenatally exposed to BPA. These data were provided in the NCBI Gene Expression Omnibus (GEO) datasets database (accession: GSE140298; https://www.ncbi.nlm.nih.gov/gds/). Regarding the samples used for the RNA-seq analysis, RNA samples were isolated from hippocampal tissues of neonatal rat pups (postnatal day 1, PND1) whose mothers were exposed to BPA at 5000 µg/kg maternal body weight daily (BPA n = 6, male pups n = 3 and female pups n = 3) or to vehicle control (control n = 6, male pups n = 3 and female pups n = 3) from gestational day 0 (GD0) until parturition as shown in Supplementary Fig. S1. All replicates were obtained from independent litters. A total of 4525 genes were identified to be significantly differentially expressed in the hippocampus of pups prenatally exposed to BPA compared to controls when all male and female pups were combined. A separate analysis of male and female pups identified 2078 and 3522 significantly differentially expressed genes in males and females, respectively. These lists of DEGs were reanalyzed through the use of IPA (QIAGEN Inc.,https://www.qiagenbioinformatics.com/products/ingenuitypathway-analysis)^68^ to predict neurological functions and behaviors associated with the hippocampus. The IPA analysis revealed that the DEGs in the hippocampal tissues were significantly associated with “cell death and survival” and “neuritogenesis” (*P* value < 0.05, Supplementary Table S1). Behaviors related to the hippocampus, including “learning”, “memory”, and “spatial learning”, were also significantly associated with DEGs (*P* value < 0.05, Supplementary Table S1).

### Quantitative RT-PCR analysis of ASD candidate genes associated with neuronal cell death, neuritogenesis, and learning/memory

**Figure 1:**
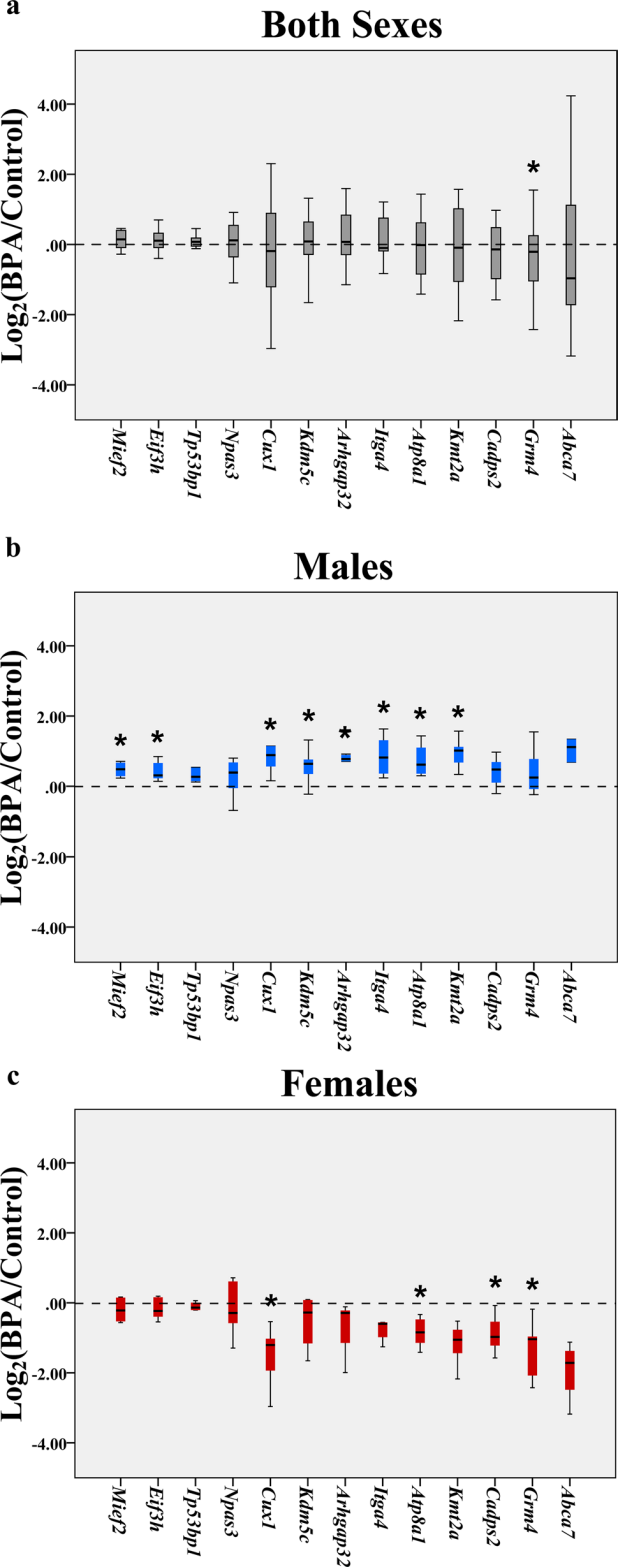
Box plot of the expression levels of DEGs involved in neuronal cell death, neuritogenesis, and learning/memory in the hippocampus of offspring. Quantitative RT-PCR analysis of the hippocampus of offspring (PND1) prenatally exposed to BPA or the vehicle control was performed to assess the expression levels of the selected genes when both sexes of offspring were combined (**a**), in male offspring only (**b**), and female offspring only (**c**). *P value < 0.05.

We then further confirmed whether prenatal BPA exposure altered the expression of ASD candidate genes associated with neuronal viability, neuritogenesis, and learning/memory ability. The animals were treated as previously described (Supplementary Fig. S1). RNA samples were isolated from hippocampal tissues from neonatal rat pups (PND1) prenatally exposed to BPA at 5000 µg/kg maternal body weight daily (BPA n = 12, male pups n = 6 and female pups n = 6) or to vehicle control (control n = 12, male pups n = 6 and female pups n = 6) from GD0 until parturition. All replicates were obtained from independent litters. We selected four DEGs associated with cell death and survival (i.e. *Mief2*, *Eif3h*, *Tp53bp1*, and *Npas3*), four DEGs associated with neuritogenesis (i.e. *Cux1*, *Kdm5c*, *Arhgap32*, and *Itga4*), and five DEGs associated with learning/memory behaviors (i.e. *Atp8a1*, *Kmt2a*, *Cadps2*, *Grm4*, and *Abca7*) for qRT-PCR analysis (Fig. 1). These BPA-responsive genes have also been identified to be ASD candidate genes. The qRT-PCR analysis showed that when male and female pups were combined, the expression levels of all selected genes were not significantly altered, except *Grm4* which was slightly down-regulated in the BPA group compared to the control (Fig. 1a). However, when the expression of these genes was analyzed separately in males and females, a sex-specific dysregulation of these ASD candidate genes was observed. The expression levels of *Mief2*, *Eif3h*, *Cux1*, *Kdm5c*, *Arhgap32*, *Itga4*, *Atp8a1*, and *Kmt2a* were significantly increased in the male offspring (Fig. 1b), whereas the expression levels of *Cux1*, *Atp8a1*, *Cadps2*, and *Grm4* were significantly reduced in the female offspring (Fig. 1c).

### Sex difference in the effects of direct BPA exposure on the viability of primary hippocampal cells

**Figure 2:**
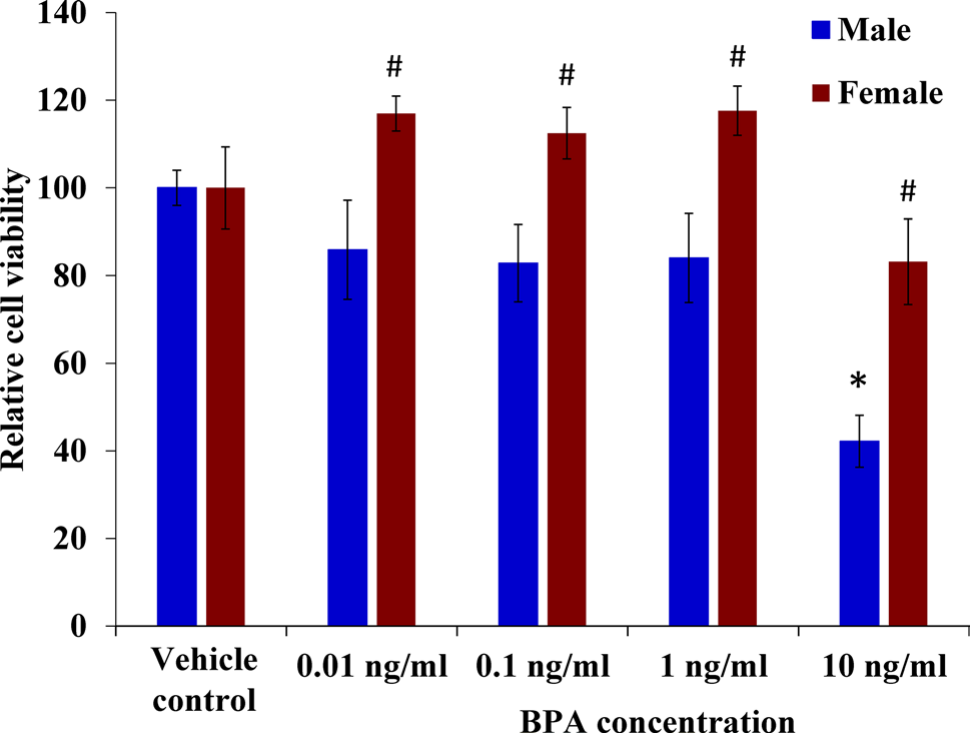
Effect of direct BPA exposure on the viability of primary hippocampal cells. Primary hippocampal cells were isolated from male and female neonatal rat pups that were not exposed to BPA and treated with 0.01, 0.1, 1, or 10 ng/mL BPA or vehicle control for 48 h. MTS assays were then performed to determine the number of viable cells. *^,#^*P* value < 0.05. *Indicates a statistically significant difference between the BPA treatment and the vehicle control groups. ^#^Indicates a statistically significant difference between males and females treated with the same concentration of BPA. Data are presented as the mean ± SEM.

Since *Mief2* and *Eif3h* which encode for the proteins Mitochondrial Elongation Factor 2 and Eukaryotic Translation Initiation Factor 3 Subunit H, respectively, play an important role in cell death^69–71^, we therefore sought to determine whether BPA exposure impacted the viability of primary hippocampal neurons. The effects of direct BPA exposure were assessed using the viability assays of cells isolated from the hippocampus of neonatal rats (Fig. 2). Primary hippocampal cells were isolated from three independent litters of neonatal rats that were not exposed to BPA. The primary cells from each litter were then divided into multiple wells and treated with BPA for 48 h at the concentrations of 0.01, 0.1, 1, 10 ng/mL, or vehicle control (n = 3 replicates/treatment group/sex/litter). The number of viable cells was assessed using MTS assays. We found that direct exposure to BPA markedly reduced the number of primary hippocampal cells from male offspring whereas the number of viable cells from females was not significantly altered compared to the vehicle control (Fig. 2). Notably, the sex difference in the BPA effects on the neuronal viability was observed when the cells were directly exposed to BPA at a concentration as low as 0.01 ng/mL. When treated with 10 ng/mL BPA, the number of viable cells from male offspring was significantly lower than those from female offspring (*P* value < 0.05).

### Sex difference in the effects of prenatal BPA exposure on the number of cells in the offspring hippocampus

**Figure 3:**
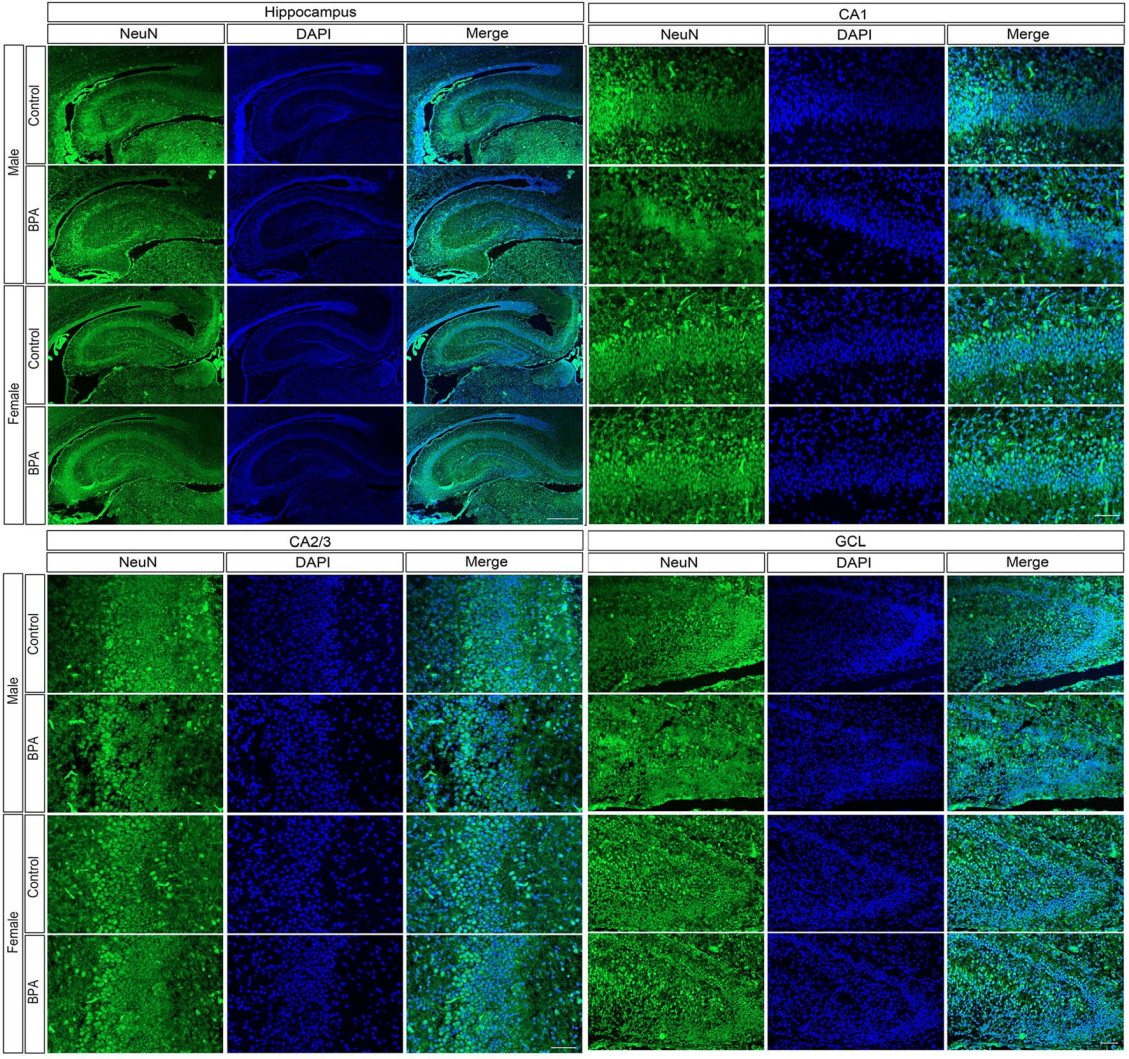
Immunofluorescence images of the hippocampus, CA1, CA2/3, and dentate gyrus of the rat offspring prenatally exposed to BPA or vehicle control. Coronal sections of the offspring hippocampus at PND1 immunostained for NeuN (green) and DAPI (blue). CA1, cornu ammonis 1; CA2/3, cornu ammonis 2/3; GCL, the granule cell layer of the dentate gyrus. Scale bar = 500 µm.

**Figure 4:**
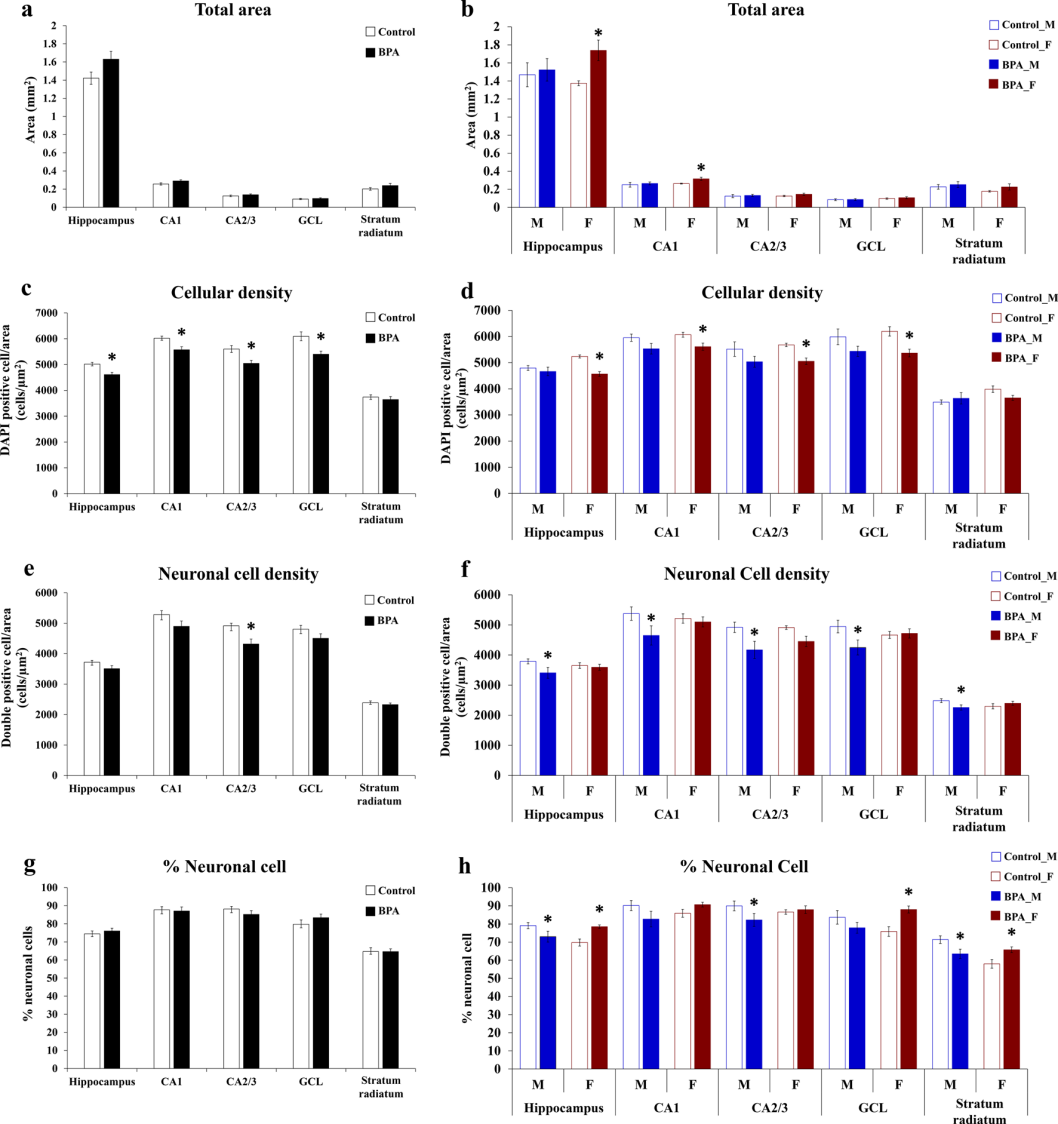
Quantification of the total area, the number of NeuN-positive cells, and the number of NeuN/DAPI double-positive cells in the hippocampus, CA1, CA2/3, DG, and stratum radiatum of the rat offspring prenatally exposed to BPA compared to those in the control group. The total area (**a**,**b**), cellular density (**c**,**d**), neuronal cell density (**e**,**f**), and percentage of neuronal cells (**g**,**h**) in the hippocampus, CA1, CA2/3, DG, and stratum radiatum were determined in the BPA group and compared with those in the vehicle control group. Hippocampus tissues (both sides) from three male and three female offspring were used in each treatment group. Data are presented as the mean ± SEM. *P value < 0.05. *Indicates a statistically significant difference between the BPA treatment and the vehicle control groups.

To further confirm the effects of BPA on the number of cells in the hippocampus of offspring, hippocampal tissues were isolated from neonatal rat pups (PND1) prenatally exposed to BPA (n = 6, male pups n = 3 and female pups n = 3, from independent litters) or vehicle control (n = 6, male pups n = 3 and female pups n = 3, from independent litters), and then immunostained with an anti-NeuN antibody which is a marker for mature neurons and counterstained with DAPI for nuclear staining (Supplementary Fig. S2a,b). The total area, cellular density, neuronal cell density, and percentage of neuronal cells in different areas of the hippocampus, including the cornu ammonis 1 (CA1), cornu ammonis 2/3 (CA2/3), and the granular cell layer (GCL) of the dentate gyrus (DG), and the stratum radiatum were assessed (Fig. 3). We found that when the data from both sexes of the offspring were combined, prenatal exposure to BPA significantly reduced cellular density in the hippocampus, CA1, CA2/3, and DG areas, and reduced the neuronal density in the CA2/3 area of the offspring (Fig. 4a,c,e, and g). When the data from the male and female offspring were analyzed separately, we found that prenatal exposure to BPA exhibited sex-dependent effects on the size, cellular density, neuronal density, and percentage of neuronal cells in the hippocampus of offspring (Fig. 4b,d,f, and h). In female offspring, prenatal BPA exposure caused a significant increase in the sizes of the hippocampus and the CA1 region (Fig. 4b), and the percentage of neuronal cells in the hippocampus, CA2/3, DG, and stratum radiatum areas (Fig. 4h). In male offspring, BPA exposure reduced the density of neurons in the hippocampus, CA1, CA2/3, DG, and striatum radiatum areas (Fig. 4f), and reduced the percentage of neuronal cells in the hippocampus, CA2/3, and stratum radiatum (Fig. 4h). It is noteworthy that the reductions of neuronal cell density and the percentage of neuronal cells in the hippocampus resulting from prenatal exposure to BPA were observed only in male but not in female offspring, consistent with the upregulation of *Mief2* and *Eif3h* (Fig. 1) and the reduced hippocampal cell viability after direct BPA exposure (Fig. 2) which were observed only in males.

### Prenatal exposure to BPA enhanced neurite formation of primary hippocampal cells from offspring

**Figure 5:**
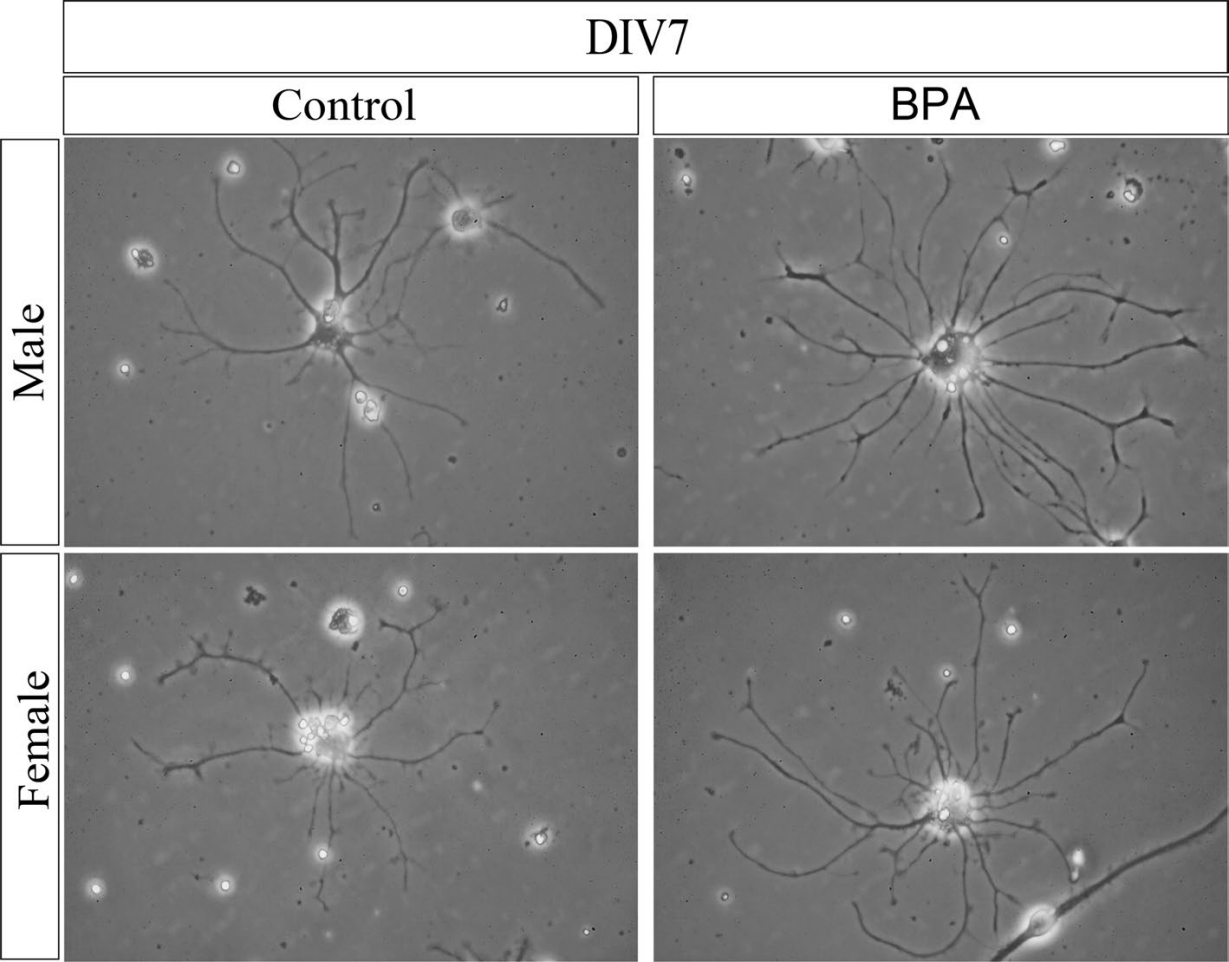
Representative images of primary hippocampal cells prenatally exposed to BPA or vehicle control. Primary hippocampal cells were isolated from male and female rat pups prenatally exposed to BPA or the vehicle control and cultured for 7 days. Images of the cells were taken using an inverted microscope.

**Figure 6:**
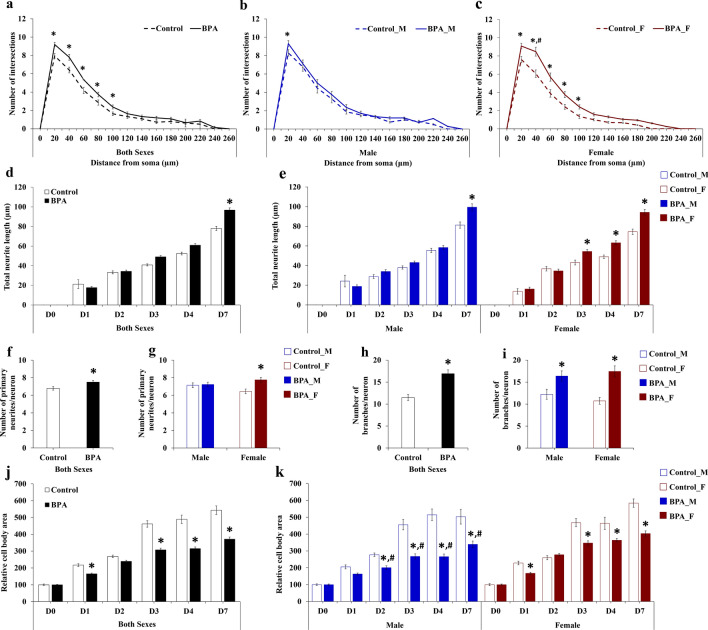
Quantification of neurite formation in primary hippocampal cells isolated from the hippocampus of the rat offspring prenatally exposed to BPA or vehicle control. Hippocampal cells were isolated from neonatal rat pups prenatally exposed to BPA or the vehicle control and cultured for 7 days. Images of the cells were taken using an inverted microscope. Sholl analysis (**a**–**c**) and evaluation of total neurite length (**d**–**e**), number of primary neurites and branching (**f**–**i**), and cell body area (**j**–**k**) were performed. Data are presented as the mean ± SEM. *^,#^*P* value < 0.05. *Indicates a statistically significant difference between the BPA treatment and the vehicle control groups. ^#^Indicates a statistically significant difference between males and females in the BPA group on the same day.

Our transcriptome profiling and qRT-PCR analyses of hippocampal tissues isolated from neonatal rat offspring showed that four ASD candidate genes (i.e. *Cux1*, *Kdm5c*, *Arhgap32*, and *Itga4*) were significantly upregulated in males prenatally exposed to BPA compared to the control. Since the upregulations of these genes are known to enhance neuritogenesis^72–75^, we therefore further examined whether prenatal BPA exposure altered the formation of neurites in offspring hippocampus. Neurite formation assays were performed using primary hippocampal cells isolated from six independent litters of neonatal rat pups prenatally exposed to BPA (n = 3 l) or vehicle control (n = 3 l). For each litter, cells from male and female offspring were obtained and pooled separately by sex. The primary cells were then cultured for 7 days without additional treatment, and a total of 150 cells/sex/treatment group were analyzed each day in vitro (DIV). The results are shown in Figs. 5 and 6a–k. Using Sholl analysis to characterize the morphological characteristics of the primary hippocampal cells, we found that primary hippocampal cells isolated from rat pups prenatally exposed to BPA exhibited a significant increase in the number of neurite intersections between 20 and 100 µm from the cell body compared to the control (Fig. 6a). A detailed neurite analysis revealed that prenatal exposure to BPA also significantly enhanced the average total neurite length (Fig. 6d), the average number of primary neurites per cell (Fig. 6f), and the average number of neurite branches per cell (Fig. 6h), but decreased the average size of the hippocampal cell body (Fig. 6j). When cells from each sex were analyzed separately, we found that all of these effects were observed in the cells from both male and female offspring, except an increase in the number of primary neurites which was found only in hippocampal cells from female offspring (Fig. 6b,c,e,g,i, and k). Notably, an increase in the length and branching of neurites of the hippocampal cells is consistent with the upregulation of the ASD candidate genes *Cux1*, *Kdm5c*, *Arhgap32*, and *Itga4* in the male hippocampus. However, in females, such relationships between the expression of these genes and neurite formation were not observed.

### Sex difference in the effects of prenatal BPA exposure on learning and memory of rat offspring

**Figure 7:**
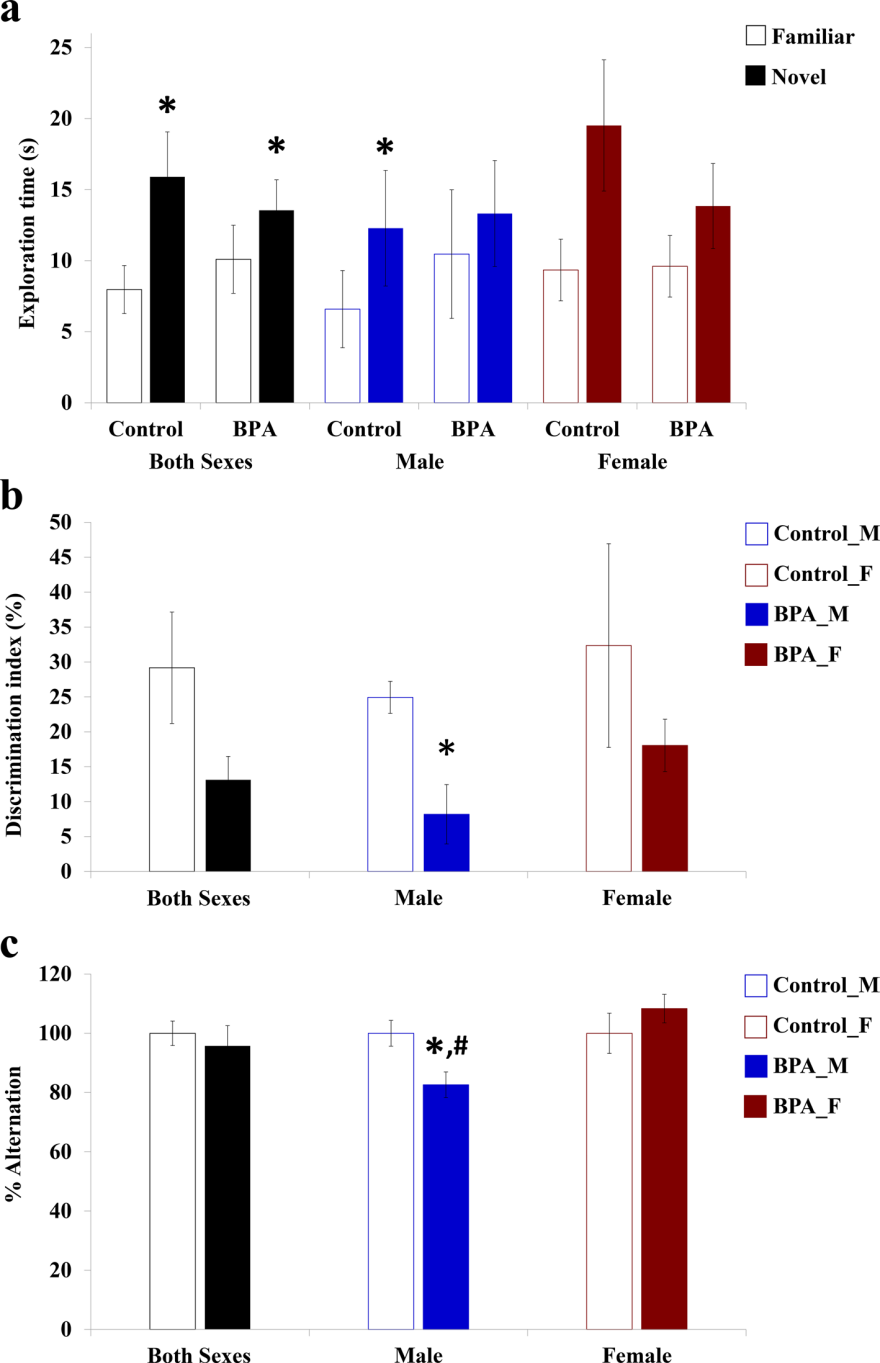
Prenatal exposure to BPA reduces learning and memory functions evaluated by the NOR test and T-maze spontaneous alternation test. The learning and memory functions in the offspring were assessed using NOR and T-maze tests. In the NOR test, the times that the rat spent exploring a familiar object and exploring a novel object were measured (**a**) and used for calculating the discrimination index (**b**). In the T-maze test, the number of turns in each goal arm was recorded, and the percentage of alternation was calculated (**c**). Data are presented as the mean ± SEM. *^,#^*P* value < 0.05. *Indicates a statistically significant difference between the BPA treatment and the vehicle control groups. ^#^Indicates a statistically significant difference between males and females in the BPA group.

The hippocampus is known to have a major role in learning and memory^76^. In addition to the genes previously linked to neuronal viability and neuritogenesis, our qRT-PCR analysis also showed that ASD candidate genes associated with learning and memory were differentially expressed in the hippocampus of offspring prenatally exposed to BPA. Among these genes, *Atp8a1* encoding for ATPase Phospholipid Transporting 8A1 protein was significantly up-regulated in the hippocampus tissues from male offspring but down-regulated in the hippocampus of the female. Increased *Atp8a1* expression was found in the hippocampus and temporal cortex of juvenile individuals with ASD, and was linked to impaired learning/memory and social behavior^77^. To further interrogate the effects of prenatal exposure to BPA on learning and memory of offspring, the novel object recognition (NOR) and the T-maze spontaneous alternation tests were performed using 16 rat pups prenatally exposed to BPA (n = 8, 4 males and 4 females) or vehicle control (n = 8, 4 males and 4 females) (Fig. 7a–c). All animal replicates were obtained by random selection from independent litters. The NOR test and the T-maze spontaneous alternation test were performed when the rat offspring were PND37-40 and PND50-55, respectively. The NOR test revealed that the time male and female offspring prenatally exposed to BPA spent exploring the novel object was not significantly different from the time they spent exploring the familiar object (Fig. 7a). A significant reduction in discrimination index was observed in male offspring prenatally exposed to BPA (Fig. 7b), indicating that prenatal BPA exposure impaired object recognition memory in male offspring. In line with the NOR test, the T-maze spontaneous alternation test showed that the male offspring exposed to BPA exhibited decreased alternation when compared to the male control (Fig. 7c). The alternation was not altered in the female offspring exposed to BPA. This finding indicates that prenatal BPA exposure impaired the working memory and spatial learning of the male offspring but did not affect those of the female. This observation is in line with the overexpression of *Atp8a1* and the loss in neuronal cell density and the percentage of neuronal cells in multiple areas of the male hippocampus.

### Correspondence between BPA-responsive genes and the disrupted neurological functions

**Figure 8:**
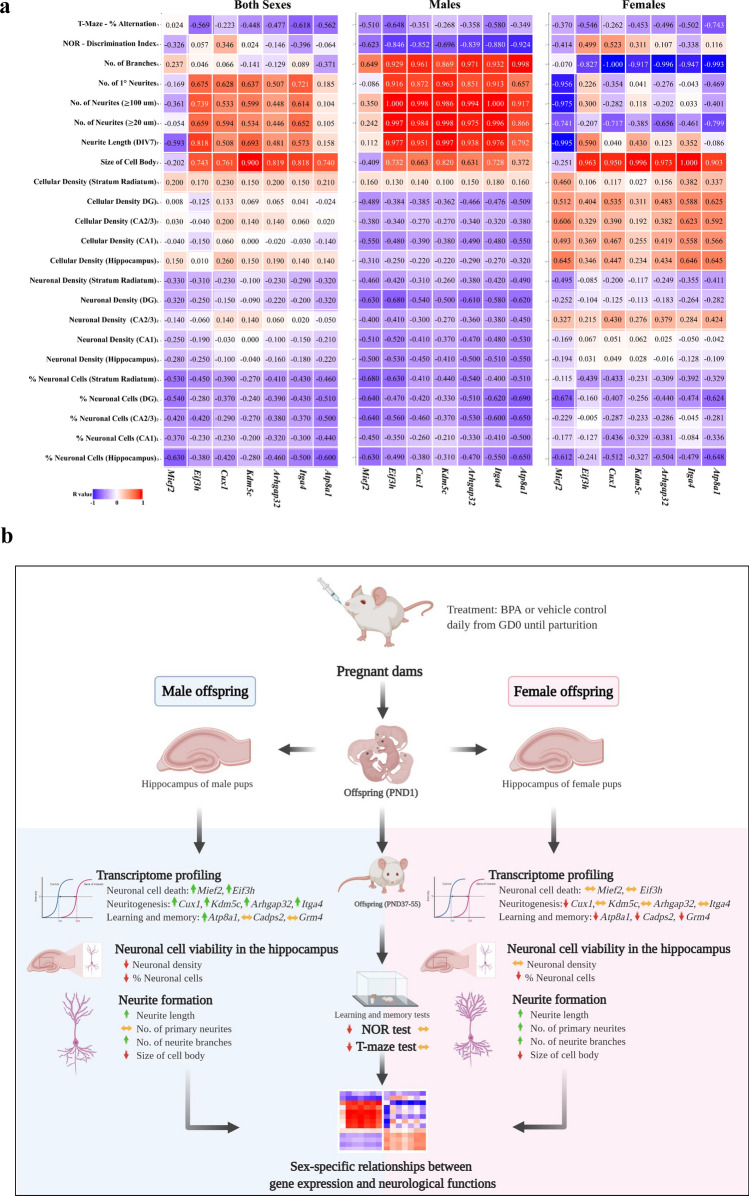
Heatmap of the correlation matrix between gene expression levels and neurological phenotypes, and schematic illustration of the main findings. (**a**) Correlation heatmap of the correlations between the expression levels of *Mief2*, *Eif3h*, *Cux1*, *Kdm5c*, *Arhgap32*, *Itga4*, and *Atp8a1* and neurological functions. Color scale denotes R^2^ from red (positive correlation) to blue (negative correlation). (**b**) A schematic diagram summarizing the main findings of this study. This figure was created with BioRender.com (http://biorender.com).

To examine the possibility that changes in the expression levels of genes in response to prenatal BPA exposure could result in changes in the neuronal viability, neuritogenesis, and learning/memory, DEGs from our previous RNA-seq analysis of hippocampal tissues isolated from neonatal rat pups prenatally exposed to BPA or vehicle control were compared with altered neurological traits assessed in this study. The neurological parameters used for this analysis were the percentage of neuronal cells in CA2/3, the neuronal density in different areas of the hippocampus (i.e. CA1, CA2/3, and DG), the cellular density in different areas of the hippocampus (i.e. CA1, CA2/3, and DG), the length of neurites on DIV7, the number of neurites and branching, the size of hippocampal cell body on DIV7, the NOR discrimination index, and the percentage of alternation in T-maze. BPA-responsive genes exhibiting changes correlated with these changes in neurological functions were identified by Pavlidis Template Matching (PTM) analysis (*P* value < 0.05). Of the 5624 genes differentially expressed in response to prenatal BPA exposure, 3256 were found to exhibit changes correlated with at least one neurological trait (*P* value < 0.05, Supplementary Table S2). Among those, a total of 2375, 2091, and 730 DEGs exhibited changes correlated with neuronal viability, neuritogenesis, and learning/memory, respectively. The lists of DEGs whose expression levels were correlated with neurological characteristics are provided in Supplementary Tables S3–S19. In addition to DEGs in response to BPA from previous RNA-seq analysis, we also examined the correspondence between selected ASD candidate genes (i.e. *Mief2*, *Eif3h*, *Cux1*, *Kdm5c*, *Arhgap32*, *Itga4*, and *Atp8a1*) and changes in neuronal viability, neuritogenesis, and learning/memory (Fig. 8a). Some neurological traits were found to have the same relationships with the gene expression regardless of the sex of offspring. An example is the expression of *Mief2* and *Atp8a1* and the percentage of neuronal cells in the hippocampus and in the dentate gyrus which exhibited an inverse correlation in both sexes of the offspring. Interestingly, we found that certain neurological traits exhibited sex-dependent relationships with the expression of these selected genes. An example of those neurological traits is the number of neurite branches that showed a positive correlation with the expression of these genes (i.e. *Mief2*, *Eif3h*, *Cux1*, *Kdm5c*, *Arhgap32*, *Itga4*, and *Atp8a1*) in the male offspring but exhibited an inverse correlation in the female. These results suggest that dysregulation of the genes induced by prenatal BPA exposure is specifically correlated with the impairment of neurological functions and with learning and memory behaviors in a sex-dependent manner, suggesting that BPA may exert its effects on neuronal viability, neuritogenesis, and learning/memory of offspring through sex-specific molecular mechanisms.

## Discussion

Our recent study has shown that prenatal exposure to BPA caused sex-dependent changes in the transcriptome profiles of genes associated with biological functions known to be negatively impacted in ASD^17^. Moreover, we found that genes previously identified to be associated with ASD were significantly enriched among those BPA-responsive genes, suggesting that prenatal BPA exposure may increase the risk of ASD by disrupting the expression of ASD candidate genes in the hippocampus and, in turn, altering neurological functions associated with ASD. In the present study, we reanalyzed the transcriptome profiling data to identify neurological functions associated with the hippocampus and ASD, and found that BPA-responsive genes in the hippocampus were significantly associated with “cell death and survival”, “neuritogenesis”, “learning”, and “memory”. These neurological functions and behaviors were therefore selected for the subsequent analyses in this study. We also conducted a correlation analysis to identify genes that exhibited the changes in the expression levels correlated with neurological functions and learning/memory ability. The major findings of our study are shown in Fig. 8b.

The no-observed-adverse-effect level (NOAEL) for BPA exposure in humans was determined by the United States Food and Drug Administration (FDA) and the European Food Safety Authority (EFSA) to be 5000 µg/kg maternal BW^78^. According to accumulating reports about BPA, EFSA reduced the tolerable daily intake (TDI) level for BPA from 50 to 4 µg/kg BW/day^79^. However, we demonstrated in this study that neonatal rat pups whose mothers were exposed to BPA at the NOAEL level during gestation exhibited disrupted transcriptome profiles, hippocampal cell viability and density, neuritogenesis, and learning/memory ability. This finding is in line with previous studies which showed that prenatal exposure to BPA could disrupt the transcriptome profiles in several brain regions, including the hippocampus, hypothalamus, and amygdala^17,22,49–51^. Although daily exposure levels of BPA in humans were thought to be lower than the NOAEL level^80^, a recent study has shown that the human exposure level could be much higher than what was previously reported due to the limitations of the analytical technique used in previous studies to measure BPA levels in human blood and urine samples^81^. Moreover, in addition to plastic products in daily life, polycarbonate microplastics and nanoplastics which were found to widely pollute food and the environment may also release BPA once ingested^4^. These findings from our studies and others strongly suggest that the NOAEL and TDI levels of BPA should be reconsidered especially for pregnant women and deserve further investigations. Since it is now clear that BPA exerts its effects on the brain transcriptome and neurological functions in a sex-dependent manner and males seem to be more impacted by BPA exposure^17,82–84^, future studies regarding the safety levels or the biological effects and mechanisms of BPA and other endocrine-disrupting chemicals should take sex differences into account. The effects of BPA at the concentrations reminiscent of actual daily exposure levels both obtained from the environment and micro/nanoplastics pollutions in humans, particularly pregnant women, deserve further investigations.

Our qRT-PCR analysis of hippocampus tissues isolated from neonatal rat pups prenatally exposed to BPA or vehicle control confirmed that maternal exposure to BPA during gestation altered the expression of ASD candidate genes involved in neuronal viability, neuritogenesis, and learning/memory in the hippocampus of offspring in a sex-dependent manner. These DEGs were *Mief2*, *Eif3h*, *Cux1*, *Kdm5c*, *Arhgap32*, *Itga4*, *Atp8a1*, *Kmt2a*, *Cadps2*, and *Grm4* (Fig. 1). Significant upregulations of *Mief2*, *Eif3h*, *Cux1*, *Kdm5c*, *Arhgap32*, *Itga4*, *Atp8a1*, and *Kmt2a* were observed in the hippocampus of male offspring, while in the female *Cux1*, *Atp8a1*, *Cadps2*, and *Grm4* were reduced.

*Mief2* and *Eif3h* play an important role in neuronal cell death. *Mief2* encodes the Mitochondrial Elongation Factor 2 which is an outer mitochondrial membrane protein that regulates mitochondrial organization and fission—a key process in cell death and mitophagy^85^. *Mief2* is essential for cristae remodeling to facilitate cytochrome c release into the cytoplasm during the early phase of intrinsic apoptosis^86^. Although the effect of *Mief2* overexpression in the hippocampus is unclear, loss of *Mief2* expression has been reported to protect the cells against intrinsic apoptosis^86^. Our correlation analysis showed that the expression of *Mief2* was inversely correlated with the percentage of neuronal cells and neuronal density in the hippocampus, and with learning/memory ability particularly in male offspring, suggesting that the overexpression of this gene might involve in the BPA-mediated loss of hippocampal neurons observed in males. *Eif3h* encodes for Eukaryotic Translation Initiation Factor 3 Subunit H which is involved in translational activation or repression and plays an important role in proliferation, cell cycle, differentiation, and apoptosis^87^. *Eif3h* is expressed during embryogenesis and regulates the development of the brain, heart, vasculature, and lateral line^88^. Overexpression of *Eif3h* was mainly associated with proliferation, invasion, and tumorigenicity in human hepatocellular carcinoma and other types of cancer^71,89,90^, but its role with respect to BPA exposure in the hippocampus is still unclear. Notably, eif3h protein was found to be significantly reduced in the hippocampus of non-transgenic mice in response to learning^91^. This learning-induced reduction in the level of eif3h was lost in a transgenic mouse model for Alzheimer’s disease which exhibited impaired learning/memory, suggesting that *Eif3h* may be involved in hippocampus-dependent learning and memory. In line with these findings, our results showed that the overexpression of *Eif3h* was inversely correlated with the learning and memory ability of male offspring assessed by the T-maze spontaneous alternation test and the NOR test. Although the role of *Eif3h* in ASD is not clearly understood, a recent study has shown that *Eif3h* can directly interact with the mammalian target of rapamycin (mTOR) and collybistin (a neuron-specific Rho-GEF) to downregulate the mTORC1 signaling cascade which is highly implicated in ASD^92^. Similar to our study, Wang et al. have investigated the effects of BPA exposure during pregnancy and lactation by treating the dams with 5 mg/kg maternal BW/day, 50 mg/kg maternal BW/day, or vehicle control from the discovery of pregnancy to 21 days after birth^93^. They found that the high concentration of BPA could promote the apoptosis of CA1 hippocampal neurons of male offspring rats, but had little effect on female offspring, suggesting that the sex difference in the BPA effects on hippocampal viability is reproducible at least at the high concentrations. However, the role of *Mief2* and *Eif3h* in BPA-induced loss of hippocampal neurons and ASD susceptibility deserve further investigations.

In addition to the genes involved in neuronal cell death and viability, many ASD candidate genes related to neuritogenesis were also differentially expressed in the hippocampus of exposed offspring in a sex-dependent manner. Among those were *Cux1*, *Kdm5c*, *Arhgap32*, and *Itga4* which were increased in the hippocampus of male offspring prenatally exposed to BPA but not in that of the female. In fact, *Cux1* was found to be significantly downregulated in the female offspring. *Cux1* (cut like homeobox 1) is a protein-coding gene that controls neuronal differentiation in the brain, and the downregulation of this gene results in a dendritic reduction in neurons^73^. *Kdm5c* (lysine demethylase 5C) mediates the methylation modifications of histone H3 at lysine 4 and plays an important role in the neuronal cell differentiation and dendritic growth^94^. Loss-of-function mutation of this gene in a human neuronal cell line resulted in decreased neurite outgrowth^72^. *Arhgap32* (Rho GTPase-activating protein 32) is involved in the differentiation of neuronal cells during the formation of neurite extensions^95^. *Itga4* (integrin subunit alpha 4) encodes a member of the integrin alpha chain family of proteins, and the upregulation of *Itga4* in neuronal cells is associated with neurite outgrowth in vitro^74^. All of these genes have been implicated in ASD^96–100^. In male offspring, the expression levels of these genes in response to BPA exposure were correlated with the number of neurite branches, the number of primary neurites, and the total neurite length, but inversely correlated with learning and memory, especially object recognition memory. Although we found that prenatal exposure to BPA significantly promoted the neuritogenesis-related phenotypes, including the length of neurites, the number of primary neurites and the number of neurite branches, the relationships between the expression of these genes and theses neuritogenesis parameters were not as clear as those observed in the male. A recent study has shown that an autism-related genomic variant in the Human Accelerated Regions 426 (HAR426) increased human *Cux1* promoter activity and the overexpression of *Cux1* resulted in increased spine density and spine head surface area^96^. Moreover, *Cux1* binds an intronic haplotype (rs1861972-rs1861973 A-C) in the homeobox transcription factor ENGRAILED2 (EN2) which was found to be associated with ASD^97^, suggesting that *Cux1* may involve in the risk of ASD. Contrary to the finding in male hippocampal cells, the expressions of these genes were inversely correlated with the number of neurite branches of female hippocampal cells. These findings suggest that the effects of prenatal BPA exposure on neurite formation are sex-dependent and that there may be other factors involved in neuritogenesis-promoting effects of BPA in the female besides these selected genes. Unlike our findings, previous studies have reported that exposure to BPA decreased the neurite lengths and complexity^101–104^. Wang et al. (2019) investigated the effect of chronic exposure to BPA on human glutamatergic neurons derived from human embryonic stem cells (hESCs)^103^. They found that chronic exposure of different concentrations of BPA (0, 0.1, 1.0, and 10 μM) to human glutamatergic neurons for 14 days reduced neurite outgrowth in a concentration-dependent manner. Similarly, Liang et al. reported that direct exposure to 1 nM BPA in vitro decreased the neurite length of neuron-like cells differentiated from neural stem cells derived from human embryonic stem cells (hESCs)^102^. The differences observed in our findings and theirs are possibly due to the difference in cell models and BPA treatment procedure. Inasmuch as ASD is an early-onset neurodevelopmental disorder and the pathobiology is thought to begin as early as in the embryonic stage, in this study we therefore focused on the effects of prenatal exposure to BPA and did not further treat the cells or the pups with BPA after birth. Thus, the effects of prenatal BPA exposure on neurite formation in the offspring observed here are likely to result from multiple factors both from the mother and from the offspring during the embryonic stage rather than direct toxicity of BPA on the cells only. The interplay between changes in the mother and in the offspring in response to gestational exposure to BPA should be further investigated. In addition to enhanced neuritogenesis, we found that prenatal BPA exposure markedly decreased the size of the hippocampal cell body, reminiscent of ASD^105,106^.

*Atp8A1* (ATPase Phospholipid Transporting 8A1) is another gene differentially expressed in the hippocampus of the offspring in response to BPA exposure. *Atp8A1* is upregulated in the hippocampus of male offspring but suppressed in the female. Our correlation analysis showed that changes in the expression levels of this gene were inversely correlated with the learning and memory ability of offspring. This gene encodes an aminophospholipid translocase (APLT) or flippase that translocates phosphatidylserine (PS) and phosphatidylethanolamine (PE) across the lipid bilayers^77^. Kerr et al. has found that Atp8a1 protein was overexpressed in post-mortem tissue homogenates from the hippocampus and temporal lobe of children with ASD compared to age-matched controls^107^. Both increased and decreased levels of *Atp8a1* during early development reduced excitatory synapses in CA1. Overexpression of this gene resulted in autistic-like sociability behavior in adult mice^107^. Moreover, we found that male offspring prenatally exposed to BPA, but not female, exhibited significant impairments in learning and memory. Our results are consistent with previous studies which demonstrated that maternal BPA exposure impairs behaviors in the offspring^56,60,108–110^, particularly learning and memory in males^83,104^.

## Conclusion

This is the first study to demonstrate that prenatal exposure to BPA disrupts ASD-candidate genes that are involved in neuronal viability, neuritogenesis, and learning/memory and that changes in the expression of these genes are correlated with neuronal characteristics and behaviors disrupted in response to BPA in a sex-dependent manner. By integrating the transcriptome profiling data and neurological phenotypes both at the cellular and behavioral levels, we identified candidate genes that are potentially involved in each neurological trait. Moreover, the sex-specific relationships between the expression of these genes and neurological traits strongly suggest that BPA negatively impacts the brain transcriptome and neurological functions in male and female offspring through different molecular mechanisms, which should be investigated thoroughly in the future. In addition, the role of these genes in neuronal viability, neuritogenesis, and learning/memory and the link between prenatal BPA exposure, dysregulation of these genes, and ASD susceptibility in each sex should be further studied. A better understanding of BPA effects on the brain and its underlying mechanisms would raise awareness about the safety of BPA or other endocrine-disrupting chemicals. Moreover, since microplastics and nanoplastics pollutions have now become a global problem and humans are widely exposed to BPA, this kind of study may also lead to the identification of molecular targets for prevention and/or treatment of diseases related to BPA toxicity including ASD in the future.

## Materials and methods

### Animals and treatment

Eight-week-old female Wistar rats were purchased from the National Laboratory Animal Center (NLAC), Thailand. Female rats at gestational day 1 (GD1) were divided into 2 groups (BPA treatment group and control group). Each rat was weighed daily, and the weight was used to calculate the dose of BPA or vehicle control. Female rats were treated with 5000 µg/kg BW of BPA or an equivalent volume of vehicle control as described in our previously published study^17^. This dose was selected based on the no-observed-adverse-effect level (NOAEL) for BPA determined by the US Food and Drug Administration (FDA) and the European Food Safety Authority (EFSA). Briefly, a stock BPA solution was prepared in absolute ethanol at a concentration of 250 mg/mL and initially diluted in corn oil to a final concentration of 5000 µg/kg BW. Each rat was treated daily with 5000 µg/kg BW or vehicle control by intragastric administration from GD1 until delivery. All animals were housed at the Chulalongkorn University Laboratory Animal Center (CULAC) under standard temperature (21 ± 1 °C) and humidity (30–70%) on a 12 h/12 h light/dark cycle with food and reverse osmosis-UV water available ad libitum. All animal procedures were approved by the Chulalongkorn University Animal Care and Use Committee (Animal Use Protocol Numbers 1673007, 1773011, and 2073011), Chulalongkorn University. All procedures were performed in accordance with relevant guidelines and regulations.

### RNA isolation and quantitative RT-PCR

Total RNA was extracted from the hippocampi using a mirVana miRNA isolation kit (Ambion, USA) according to the manufacturer’s protocol to confirm the expression profiles of the genes related to cell death and survival, neuritogenesis, and behaviors (BPA n = 12, male pups n = 6 and female pups n = 6; control n = 12, male pups n = 6 and female pups n = 6). Information about the samples used in this study is presented in Supplementary Table S20. Four genes related to cell death, four neuritogenesis-related genes, and five behavior-related genes were selected based on the analysis of our transcriptome data (accession no. GSE140298; https://www.ncbi.nlm.nih.gov/gds/) for confirmation using qRT-PCR. The prediction of biological functions associated with these genes was analyzed through the use of IPA (QIAGEN Inc., https://www.qiagenbioinformatics.com/products/ingenuitypathway-analysis)^68^. Significant biological functions associated with these genes were identified using Fisher's exact test (*P* value < 0.05). Total RNA was used to synthesize cDNA using a RevertAid RT reverse transcription kit (Thermo Fisher Scientific, USA) according to the manufacturer’s protocol as described previously^17,22^. Briefly, 0.5 µg of total RNA was used to generate the first-strand cDNA in a reaction containing 1 µL of oligo dT_18_ primers and DEPC-treated water. The samples were then incubated at 65 °C for 5 min and chilled on ice for 10 min. Then, the sample was mixed with 4 µL of reaction buffer, 1 µL of RNase inhibitor, 1.5 µL of 10 mM dNTP mix, and 1 µL of reverse transcriptase to synthesize the cDNA templates. The cDNA synthesis reaction was incubated at 25 °C for 5 min followed by incubation at 42 °C for 60 min. The cDNA reaction mixture was diluted to a volume of 50 μl with nuclease-free water and used as a template for subsequent qRT-PCR analysis as described previously^17,22^. qRT-PCR was performed using AccuPower 2X GreenStar qPCR MasterMix (Bioneer, Korea) according to the manufacturer’s protocol. Briefly, 1 μl of cDNA template was mixed with 2× Greenstar Master Mix, forward primers, reverse primers, and nuclease-free water. The reaction was performed under the following conditions: predenaturation at 95 °C for 15 min followed by 40 cycles of denaturation at 95 °C and annealing/extension at 55 °C for 30 s. All reactions were performed in at least 3 replicates. Product formation was confirmed by a melting curve analysis (65–95 °C). The expression levels were calculated using the 2^−ΔΔCt^ method with the 18S ribosomal RNA (*Rn18s*) gene as an endogenous control as described previously^17,22^. The sequences of the forward and reverse qPCR primers for rat *Mief2*, *Eif3h*, *Tp53bp1*, *Npas3*, *Cux1*, *Kdm5c*, *Arhgap32*, *Itga4*, *Atp8a1*, *Kmt2a*, *Cadps2*, *Grm4*, *Abca7*, and *Rn18s* are listed in Supplementary Table S21.

### Hippocampal cell viability assay

Primary hippocampal cells were isolated from three independent litters of neonatal rats that were not exposed to BPA using a previously published protocol with slight modifications^111^. Briefly, hippocampi were dissected under a stereomicroscope (Nikon, Japan) in the dissection medium (Supplementary Table S22). Then, the Neural Tissue Dissociation kit (Miltenyi Biotec, Germany) was used to dissociate the cells. Initially, 2 mL of Enzyme Mix 1 was added to the solution containing the dissection medium. The tube containing tissue chunks and enzyme mix 1 was rotated at room temperature for 20 min. Then, 30 µL of Enzyme Mix 2 was added, and the tubes were rotated for another 20 min. A fire-polished glass pipette was used to triturate the tissue. The cells were then washed twice with the N2 medium. Primary hippocampal cells were seeded in a 96-well plate precoated with poly-l-lysine (Sigma-Aldrich, USA) and cultured in the seeding medium (Supplementary Table S22) overnight. The cells were seeded at a density of 10,000 cells/well and incubated for 4 h to ensure that the cells were attached to the well. After 4 h incubation, the cells were treated with various concentrations of BPA (0, 0.01, 0.1, 1, or 10 ng/mL) and incubated for 48 h. Then, 20 µL of CellTiter 96 AQueous One Solution reagent (Promega, USA) was added to each well to detect cell viability, and the plates were incubated at 37 °C. The absorbance was read at 490 nm after 1, 2, 3, and 4 h incubation using a plate reader (Perkin Elmer, USA). The experiment was performed in at least 3 replicates.

### Immunofluorescence staining and confocal microscopy

Neonatal rat pups at postnatal day 1 (PND1) were euthanized, and the brain was collected from 3 independent litters (BPA n = 6; control n = 6). The brain was submerged in a freshly prepared solution of 4% paraformaldehyde in PBS overnight. The paraformaldehyde solution was replaced with a 30% sucrose solution in PBS, and the brain was submerged overnight at 4 °C. Excess sucrose solution was removed from the exterior of the tissue prior to embedding it in OCT (Sakura Finetek, USA). The brain was sectioned using a Leica cryostat (Leica, USA) to obtain the sections of the hippocampus. The hippocampal sections were immunostained with an antibody against NeuN, a mature neuronal marker. Briefly, the sections were dried for 15 min and washed three times with TBST (0.1% Triton X-100) for 5 min. Antigen was retrieved by boiling of the sections in 0.01 M citric acid, pH 6.0, for 30 min. Hippocampal tissue sections were then blocked with 3% BSA for 1 h at room temperature. A mouse anti-NeuN antibody (1:1000, ab104224, Abcam) was then added to the sections and incubated at 4 °C overnight. Sections were washed three times to remove the primary antibody. A secondary donkey anti-mouse antibody Alexa Fluor 488 (1:2000, ab150109, Abcam) was added and the sections were counterstained with DAPI followed by incubation at room temperature for 1 h. The sections were then washed again with TBST. The sections were mounted using ProLong Diamond Antifade reagent (Invitrogen, USA) and NeuN^+^ cells were detected using an LSM800 confocal laser scanning microscope (Carl Zeiss, Germany). The numbers of NeuN^+^, DAPI^+^, and double-positive cells in the hippocampus were counted using ImageJ software plugin with U-Net according to previously published protocol^112^.

### Neurite formation assay

A 35-mm dish (Falcon, USA) was precoated with poly-l-lysine (Sigma-Aldrich, USA) for 30 min before the cells were seeded. Primary hippocampal cells were isolated from the hippocampi of 3 different litters of neonatal rats that were prenatally treated with BPA (5000 µg/kg BW per day) or vehicle control during the gestation period. Briefly, hippocampi were digested using the Neural Tissue Dissociation kit (Miltenyi Biotec, Germany) to separate single cells using the same procedure that was used in the cell viability experiment. Subsequently, the cells were suspended in the plating medium (see detailed description in Supplementary Table S22) and seeded at a density of 65 cells per mm^2^. The cells were incubated in a cell culture incubator at 37 °C in an atmosphere containing 5% CO_2_ for 2–4 h to ensure that all cells are attached to the dish. Four hours after plating of the cells, the maintenance medium (see detailed description in Supplementary Table S22) was added to the dish to a final volume of 3 mL. Cells (150 cells/sex/treatment group) were imaged using an inverted microscope (Carl Zeiss Axio Observer, Germany) equipped with a digital camera (Nikon Eclipse TE 300, Japan) on days 0, 1, 2, 3, 4, and 7 for the neurite formation assay. The results of the neurite formation assay were analyzed using the Neurolucida software (http://www.mbfbioscience.com/neurolucida). Various parameters were evaluated, including Sholl analysis, total neurite length, number of primary neurites, neurite branching, and cell body area. Sholl analysis was performed to evaluate the total length of neurites or the number of intersections in the spheres with increasing radii, which were constructed as the radii around the cell body with a 20 µm distance between the radii. The total neurite length, number of primary neurites, and branching were measured for each hippocampal cell. A primary neurite was defined as a neurite that directly emerges from the cell body. The total area of the cell body of each hippocampal cell was measured on DIV0-DIV7 and normalized using the area of the cell body on DIV0.

### Behavioral tests

#### The NOR task

The NOR procedure was performed using previously published methods^113^. The test consists of two phases: the habituation phase and the test phase. The initial habituation phase included a 10 min session for each rat at 37–40 days of age (BPA n = 8; control n = 8). Each rat was allowed to freely explore the arena on three consecutive days. Rats were placed in the arena, and a video was recorded. The arena was cleaned with 70% ethanol before and after the experiment. The trial phase of the test consisted of (1) the familiarization trial followed by (2) the test trial. This test was performed on day 4 after three days of the habituation phase. In the familiarization trial, each rat was placed in the arena containing 2 identical objects against the center of the opposite wall with its back to the objects. The interaction between each rat and the objects was defined as direct entry into the object zone and contact with the object with the nose or whiskers. All rats were returned to their home cages between the habituation and test trials for 15 min. The test trial was then performed in the same manner as the familiarization trial, except that one object in the familiarization trial was replaced with a novel object that had a different shape and color. The object zone was randomly selected. An animal was predicted to spend more than 50% of its total object interaction time exploring the novel object. A video of the entire experiment was recorded. Experimenters were blinded to the treatment and control groups during all trials and analyses.

#### T-maze spontaneous alternation test

The T-maze spontaneous alternation test is a behavioral test measuring exploratory behavior based on the willingness of the rats to explore a new environment, i.e., a new arm of the maze. Rats 50–55 days of age (BPA n = 8; control n = 8) were initially placed in the starting arm of the T-maze; then, the animals decided to enter the left or right goal arm in eleven repeated trials. A video camera was used to record the entire experiment. The percentage of choices in favor of each goal arm was determined. Experimenters were blinded to the treatment and control group during the test and analysis of the data.

### Correlation analysis

Correlation analysis was performed to examine the possibility of the association between the gene expression level in response to BPA and the changes in neurological function. The expression value of DEGs obtained from RNA-seq data and the mean values of each neurological function were uploaded to MeV software^114,115^ to perform Pavlidis Template Matching (PTM) analysis^116^. The list of DEGs, R values, and *P* values was determined. Moreover, the expression values of selected DEGs which are ASD candidate genes were used to further examine sex differences in the correspondence of neuronal viability, neuritogenesis, and learning/memory. The expression values of DEGs were obtained from qRT-PCR analysis and matched with the mean values of each neurological function in both sexes, males, and females. R values of each correlation were determined and used to construct a heatmap in both sexes, males, and females.

### Statistical analyses

Statistical analysis was performed using the SPSS software package for Windows. The differences in cell viability between the groups, significance of the differences of the means of the numbers of NeuN^+^, DAPI^+^, and double-positive cells, the total neurite length, and cell body area in more than 2 groups were assessed using ANOVA followed by LSD post hoc test. Two-tailed Student’s t-test was performed to determine the statistical significance of the differences of the mean values of two groups in Sholl analysis, the number of primary neurites and branching of hippocampal cells, and behavioral tests. A *P* value < 0.05 was considered statistically significant.

### Ethical approval and informed consent

All animal experimental procedures were approved by the Chulalongkorn University Animal Care and Use Committee (Animal Use Protocol No. 1673007, No. 1773011, and No. 2073011), Chulalongkorn University.

## Supplementary Information

Supplementary Information.

## Data Availability

The transcriptome profiling data used in this study have been deposited in the NCBI GEO dataset database (GSE140298).
