# Supplementary material for: Sex differences in the effects of prenatal bisphenol A exposure on autism-related genes and their relationships with the hippocampus functions
Source: Sci Rep. 2021 Jan 13;11:1241. doi: 10.1038/s41598-020-80390-2 (PMC7806752; doi:10.1038/s41598-020-80390-2)
Supplement: Supplementary file 1 — Supplementary Information. [file 41598_2020_80390_MOESM1_ESM.pdf]

Sex differences in the Effects of Prenatal Bisphenol A Exposure on Autism-related Genes and their Relationships with the Hippocampus Functions

Surangrat Thongkorn[1], Songphon Kanlayaprasit[1], Pawinee Panjabud[1], Thanit Saeliw[1], Thanawin Jantheang[1], Kasidit Kasitipradit[1], Suthathip Sarobol[2], Depicha Jindatip[3,4], Valerie W. Hu[5], Tewin Tencomnao[6], Takako Kikkawa[7], Tatsuya Sato[8], Noriko Osumi[7], and Tewarit Sarachana[4,6,*]

[1]The Ph.D. Program in Clinical Biochemistry and Molecular Medicine, Department of Clinical Chemistry, Faculty of Allied Health Sciences, Chulalongkorn University, Bangkok, Thailand

[2]Specimen Center, Department of Laboratory Medicine, King Chulalongkorn Memorial Hospital, Bangkok, Thailand

[3]Department of Anatomy, Faculty of Medicine, Chulalongkorn University, Bangkok, Thailand

[4]SYstems Neuroscience of Autism and PSychiatric disorders (SYNAPS) Research Unit, Department of Clinical Chemistry, Faculty of Allied Health Sciences, Chulalongkorn University, Bangkok, Thailand

[5]Department of Biochemistry and Molecular Medicine, The George Washington University School of Medicine and Health Sciences, The George Washington University, Washington, DC USA

[6]Age-related Inflammation and Degeneration Research Unit, Department of Clinical Chemistry, Faculty of Allied Health Sciences, Chulalongkorn University, Bangkok, Thailand

[7]Department of Developmental Neuroscience, United Centers for Advanced Research and Translational Medicine (ART), Tohoku University Graduate School of Medicine, Sendai, Miyagi, Japan

[8]Department of Healthcare Management, Faculty of Health Sciences, Tohoku Fukushi University, Sendai, Miyagi, Japan

*Corresponding author:

Asst. Prof. Tewarit Sarachana, Ph.D.

SYstems Neuroscience of Autism and PSychiatric disorders (SYNAPS) Research Unit,

Age-related Inflammation and Degeneration Research Unit,

Department of Clinical Chemistry, Faculty of Allied Health Sciences, Chulalongkorn University, Bangkok, Thailand

154 Soi Chula 12, Rama 1 Road, Wangmai, Pathumwan, Bangkok, 10330, Thailand

Tel. 662-218-1081 ext. 313

Fax. 662-218-1082

E-mail: tewarit.sa@chula.ac.th

E-mail addresses of co-authors:

ST	E-mail: 6176957037@student.chula.ac.th
SK	E-mail: songphon.ka@student.chula.ac.th
PP	E-mail: 6176952837@student.chula.ac.th
TSae	E-mail: 6271004937@student.chula.ac.th
TJ	E-mail: thanawin.ja@student.chula.ac.th
KK	E-mail: 6076951037@student.chula.ac.th
SS	E-mail: sa.suthathip@gmail.com
DJ	E-mail: depicha.j@chula.ac.th

VWH E-mail: valhu@gwu.edu

TT E-mail: tewin.t@chula.ac.th

TK E-mail: kikkawa@med.tohoku.ac.jp

TSato E-mail: tatsuyasato1118@gmail.com

NO E-mail: osumi@med.tohoku.ac.jp

Supplementary Figure S1. Schematic diagram of the animal experimental design. This figure was created with BioRender.com (http://biorender.com).

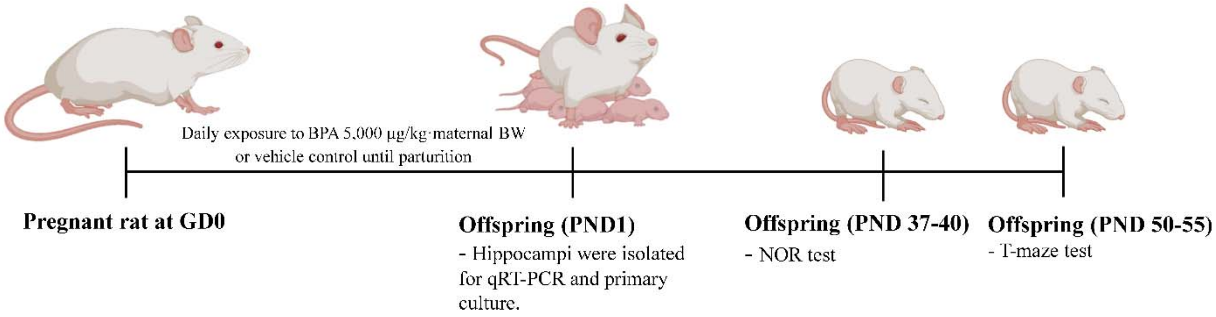

Supplementary Figure S2. Representative images of PND1 rat offspring hippocampus. a) a coronal section of the offspring hippocampus at PND1 immunostained for NeuN (green) and DAPI (blue). The image is magnified in b). CA1, cornu ammonis 1; CA2, cornu ammonis 2; CA3, cornu ammonis 3.

a

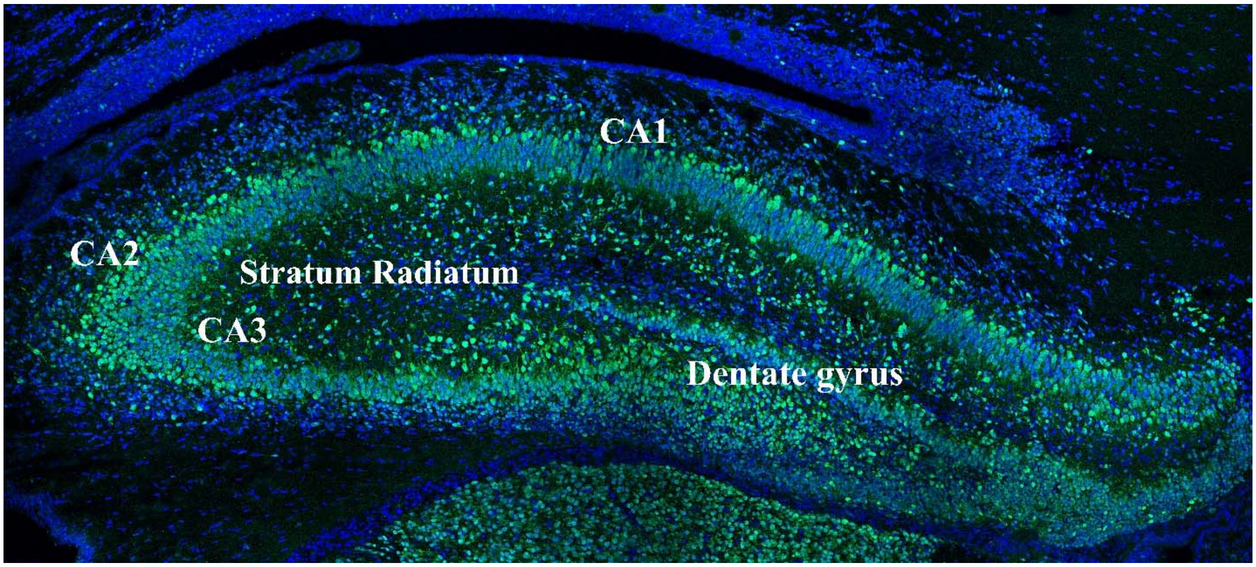

b

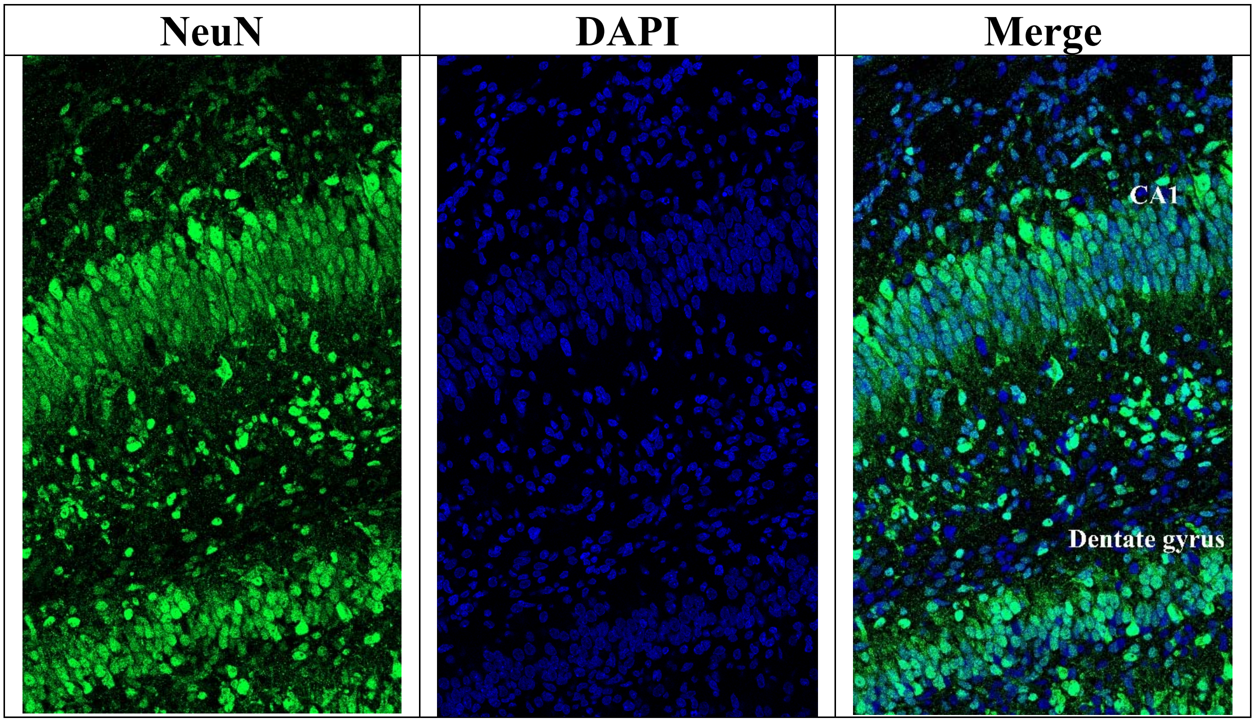

Supplementary Table S1. Neurological functions significantly associated with DEGs in the neonatal rat hippocampus prenatally exposed to BPA. DEGs in the neonatal rat hippocampus prenatally exposed to BPA from RNA-seq analysis were analyzed through the use of IPA (QIAGEN Inc., https://www.qiagenbioinformatics.com/products/ingenuitypathway-analysis)[68]. to predict neurological functions associated with DEGs using DEGs list from both sexes, males, and females. P-values were calculated using Fisher's exact test. P-value < 0.05 is considered as significant.

**Categories**	**P-value**	**# DEGs**
**Both sexes**		
Cell death and survival		
Cell survival	1.17E-12	587
Necrosis	1.66E-11	977
Apoptosis	2.46E-11	975
Cell viability	2.52E-11	552
Neuronal cell death	2.95E-07	258
Nervous System Development and function		
Development of neurons	3.72E-09	322
Morphogenesis of neurons	2.19E-08	251
Neuritogenesis	6.07E-08	246
Abnormal morphology of cerebellum	8.39E-07	63
Abnormal morphology of neurons	9.68E-07	193
**Male**		
Cell death and survival		
Neuronal cell death	4.98E-09	120
Necrosis	1.61E-06	369
Cell survival	1.59E-05	216
Apoptosis of neurons	1.74E-05	72
Nervous System Development and function		
Development of neurons	1.62E-27	195
Morphogenesis of neurons	4.17E-27	162
Neuritogenesis	7.59E-26	158
Proliferation of neuronal cells	3.47E-13	113
Branching of neurites	2.11E-12	74
Behavior		
Learning	1.15E-14	100
Cognition	1.44E-13	104
Anxiety	8.40E-10	50
Memory	1.91E-09	61
Contextual conditioning	3.09E-08	26
**Female**		
Cell death and survival		
Necrosis	2.44E-30	735
Apoptosis	2.50E-22	699
Cell survival	1.67E-21	432

Cell viability	8.00E-18	399
Neuronal cell death	2.51E-15	206
Nervous System Development and function		
Morphology of nervous system	5.76E-41	359
Development of neurons	6.22E-40	313
Neuritogenesis	1.89E-36	250
Morphogenesis of neurons	2.15E-27	252
Proliferation of neuronal cells	1.77E-25	200
Behavior		
Learning	2.25E-20	159
Cognition	2.65E-20	170
Spatial learning	2.20E-13	67
Memory	1.00E-11	94
Locomotion	3.51E-11	112

Supplementary Table S2. The number of differentially expressed genes in the hippocampus of rat offspring prenatally exposed to BPA that exhibited the changes in the expression levels correlated with each neurological function.

Neurological Functions	Number of DEGs
**Neuronal viablity, neuritogenesis, or learning/memory**	3,256
**Neuronal viablity**	2,375
- Percentage of neuron in CA2/3	132
- Neuronal density in the hippocampi	450
- Neuronal density in CA1	369
- Neuronal density in CA2/3	608
- Neuronal density in GCL	132
- Cellular density in the hippocampi	646
- Cellular density in CA1	717
- Cellular density in CA2/3	777
- Cellular density in GCL	793
**Neuritogenesis**	2,091
- Neurite length at DIV3	443
- Neurite length at DIV7	722
- Number of neurites (≥ 20 μm)	697
- Number of neurites (≥ 100 μm)	764
- Number of primary neurites	479
- Number of branches	795
**Learning/memory**	730
- NOR Discrimination index	562
- % Alternation in T-maze	186

Supplementary Table S3. The list of genes that are differentially expressed in the offspring hippocampus in response to prenatal BPA exposure that exhibited the changes in the expression levels correlated with the percentage of neurons in CA2/3 of the hippocampus. The transcriptome profiling data of DEGs in male and female rat offspring prenatally exposed to BPA (n = 6, male pups n = 3 and female pups n = 3, from independent litters) or the vehicle control (n = 6, male pups n = 3 and female pups n = 3, from independent litters) were obtained and used for the PTM analyses to identify DEGs that exhibited the changes in the expression levels correlated with the percentage of neurons in CA2/3 of the hippocampus in both sexes of offspring.

ID	Symbol	Entrez Gene Name	log2(FC)	R values	P-values
281371353	IL17B	interleukin 17B	-1.507	0.967	0.033
157822647	CD70	CD70 molecule	-1.492	0.966	0.034
157820541	RGS9BP	regulator of G protein signaling 9 binding protein	-1.480	0.971	0.029
564352410	ARTN	artemin	-1.379	0.971	0.029
157952196	Tmem125	transmembrane protein 125	-1.379	0.971	0.029
282847351	LRRC36	leucine rich repeat containing 36	-1.322	0.952	0.048
71043730	VNN1	vanin 1	-1.322	0.952	0.048
157819313	RGD1561661	similar to Ferritin light chain (Ferritin L subunit)	-1.206	0.990	0.010
157824077	CCRL2	C-C motif chemokine receptor like 2	-1.206	0.990	0.010
157819399	NOXO1	NADPH oxidase organizer 1	-1.206	0.990	0.010
58865684	MCOLN3	mucolipin 3	-1.158	0.989	0.011
13929148	CNGB1	cyclic nucleotide gated channel subunit beta 1	-1.000	0.971	0.029
198386353	MYO1G	myosin IG	-1.000	0.986	0.014
157819701	Ctla2a	cytotoxic T lymphocyte-associated protein 2 alpha	-0.966	0.980	0.020
564382020	TMEM63A	transmembrane protein 63A	-0.896	0.955	0.045
672022227	RGS22	regulator of G protein signaling 22	-0.855	0.974	0.026
119226204	CFAP206	cilia and flagella associated protein 206	-0.849	0.953	0.047
162287322	LSP1	lymphocyte specific protein 1	-0.781	0.989	0.011
66730425	MGC105567	similar to cDNA sequence BC023105	-0.755	0.981	0.019
157822593	NEIL2	nei like DNA glycosylase 2	-0.753	0.964	0.036
319009550	PPM1N	protein phosphatase, Mg2+/Mn2+ dependent 1N (putative)	-0.750	0.979	0.021
760997729	SYNPO2L	synaptopodin 2 like	-0.621	0.956	0.044
291463305	SHISA9	shisa family member 9	-0.574	0.968	0.032
188497675	RADX	RPA1 related single stranded DNA binding protein, X-linked	-0.542	0.968	0.032
17105368	KLF9	Kruppel like factor 9	-0.505	0.953	0.047
157824113	GPR84	G protein-coupled receptor 84	-0.485	0.971	0.029

672084224	CCDC113	coiled-coil domain containing 113	-0.458	0.988	0.012
194440693	Maml2	mastermind like transcriptional coactivator 2	-0.457	0.988	0.012
564346692	GIMAP8	GTPase, IMAP family member 8	-0.455	0.953	0.047
6978629	CD38	CD38 molecule	-0.440	0.962	0.038
157824150	PTPN22	protein tyrosine phosphatase non-receptor type 22	-0.389	0.972	0.028
8393919	LOC100911216/Pcsk1	proprotein convertase subtilisin/kexin type 1	-0.371	0.951	0.049
26024221	PRSS12	serine protease 12	-0.366	0.973	0.027
56605758	THAP1	THAP domain containing 1	-0.340	0.980	0.020
70794782	RBMS2	RNA binding motif single stranded interacting protein 2	-0.316	0.967	0.033
12621142	RASSF9	Ras association domain family member 9	-0.299	0.953	0.047
157822577	MAN1C1	mannosidase alpha class 1C member 1	-0.292	0.984	0.016
31560385	RPL21	ribosomal protein L21	-0.252	0.974	0.026
157822599	GSAP	gamma-secretase activating protein	-0.237	0.963	0.037
62078935	FLACC1	flagellum associated containing coiled-coil domains 1	-0.233	0.975	0.025
9506425	BET1	Bet1 golgi vesicular membrane trafficking protein	-0.215	0.955	0.045
672089580	LOC103694865	TATA-binding protein-associated factor 2N-like	-0.197	0.951	0.049
123780073	YOD1	YOD1 deubiquitinase	-0.193	0.974	0.026
300797496	TDRD6	tudor domain containing 6	-0.184	0.990	0.010
112984202	FZD8	frizzled class receptor 8	-0.183	0.958	0.042
269954719	JAZF1	JAZF zinc finger 1	-0.171	0.961	0.039
157822019	ITGB1BP1	integrin subunit beta 1 binding protein 1	-0.166	0.987	0.013
53850598	DDX59	DEAD-box helicase 59	-0.134	0.955	0.045
62078739	TCTA	T cell leukemia translocation altered	-0.131	0.980	0.020
300794591	FXN	frataxin	-0.110	0.985	0.015
81884516	Rhno1	RAD9-HUS1-RAD1 interacting nuclear orphan 1	-0.107	0.968	0.032
300794891	DDX20	DEAD-box helicase 20	-0.060	0.950	0.050
157817861	NDUFA2	NADH:ubiquinone oxidoreductase subunit A2	-0.057	0.958	0.042
188536098	SLC48A1	solute carrier family 48 member 1	-0.055	0.986	0.014
157820373	ELK1	ETS transcription factor ELK1	-0.043	0.954	0.046
149045755	CREB3	cAMP responsive element binding protein 3	-0.043	0.968	0.032

148686551	PPWD1	peptidylprolyl isomerase domain and WD repeat containing 1	0.038	-0.987	0.013
149049696	MKRN2	makorin ring finger protein 2	0.052	-0.976	0.024
149052177	MRPL28	mitochondrial ribosomal protein L28	0.052	-0.986	0.014
50511039	GNB1L	G protein subunit beta 1 like	0.054	-0.964	0.036
157819325	SRP68	signal recognition particle 68	0.056	-0.981	0.019
148672705	TMEM184B	transmembrane protein 184B	0.080	-0.965	0.035
157818421	TVP23A	trans-golgi network vesicle protein 23 homolog A	0.085	-0.983	0.017
564388185	ERCC6	ERCC excision repair 6, chromatin remodeling factor	0.112	-0.959	0.041
189011602	NLE1	notchless homolog 1	0.118	-0.977	0.023
11968114	MRPL23	mitochondrial ribosomal protein L23	0.127	-0.977	0.023
564361244	TCF20	transcription factor 20	0.128	-0.960	0.040
157821561	ATRIP	ATR interacting protein	0.132	-0.987	0.013
11177894	TSC1	TSC complex subunit 1	0.134	-0.959	0.041
157819423	SPSB3	splA/ryanodine receptor domain and SOCS box containing 3	0.150	-0.974	0.026
50511177	SLITRK1	SLIT and NTRK like family member 1	0.155	-0.968	0.032
157820917	CDC7	cell division cycle 7	0.156	-0.977	0.023
74200325	UBE2G2	ubiquitin conjugating enzyme E2 G2	0.158	-0.951	0.049
11559951	NRBF2	nuclear receptor binding factor 2	0.163	-0.987	0.013
300797915	Rbm33	RNA binding motif protein 33	0.166	-0.964	0.036
46485387	NAPRT	nicotinate phosphoribosyltransferase	0.167	-0.962	0.038
40807349	DSTYK	dual serine/threonine and tyrosine protein kinase	0.172	-0.957	0.043
392355126	HAUS2	HAUS augmin like complex subunit 2	0.175	-0.961	0.039
564303143	KMT2C*	lysine methyltransferase 2C	0.176	-0.981	0.019
38454226	TPD52L2	TPD52 like 2	0.180	-0.973	0.027
8393959	PIM1	Pim-1 proto-oncogene, serine/threonine kinase	0.188	-0.981	0.019
9507235	UGT8	UDP glycosyltransferase 8	0.196	-0.971	0.029
672031398	ANKRD11	ankyrin repeat domain 11	0.203	-0.973	0.027
149030718	PIP5K1A	phosphatidylinositol-4-phosphate 5-kinase type 1 alpha	0.211	-0.971	0.029
109470195	TNKS1BP1	tankyrase 1 binding protein 1	0.213	-0.957	0.043
189181698	ZNF131	zinc finger protein 131	0.225	-0.979	0.021
19173786	SYF2	SYF2 pre-mRNA splicing factor	0.229	-0.960	0.040
67078478	NAF1	nuclear assembly factor 1 ribonucleoprotein	0.237	-0.978	0.022
256818763	PLEKHH1	pleckstrin homology, MyTH4 and FERM domain containing H1	0.252	-0.966	0.034

148666908	ADAMTS9	ADAM metallopeptidase with thrombospondin type 1 motif 9	0.256	-0.955	0.045
205830446	C11orf98	chromosome 11 open reading frame 98	0.275	-0.990	0.010
109464919	ARHGEF26	Rho guanine nucleotide exchange factor 26	0.278	-0.951	0.049
145553978	SFMBT1	Scm like with four mbt domains 1	0.297	-0.971	0.029
293349725	AMER3	APC membrane recruitment protein 3	0.303	-0.957	0.043
72255513	AGA	aspartylglucosaminidase	0.309	-0.950	0.050
149048116	KHDC4	KH domain containing 4, pre-mRNA splicing factor	0.314	-0.951	0.049
76096340	ANKRD16	ankyrin repeat domain 16	0.354	-0.958	0.042
16758666	TIMP1	TIMP metallopeptidase inhibitor 1	0.376	-0.961	0.039
13928944	P2RY4	pyrimidinergic receptor P2Y4	0.415	-0.967	0.033
67078462	SOX18	SRY-box transcription factor 18	0.460	-0.954	0.046
157819737	SARS2	seryl-tRNA synthetase 2, mitochondrial	0.463	-0.970	0.030
281306771	ADAMTS4	ADAM metallopeptidase with thrombospondin type 1 motif 4	0.489	-0.968	0.032
300798653	ALPK3	alpha kinase 3	0.515	-0.979	0.021
564303143	KMT2C*	lysine methyltransferase 2C	0.593	-0.956	0.044
148683584	VEPH1	ventricular zone expressed PH domain containing 1	0.603	-0.973	0.027
672014266	TMEM219	transmembrane protein 219	0.735	-0.969	0.031
6978663	CLCN1	chloride voltage-gated channel 1	0.742	-0.969	0.031
293347270	OSGIN2	oxidative stress induced growth inhibitor family member 2	0.861	-0.955	0.045
672088752	MCF2	MCF.2 cell line derived transforming sequence	0.909	-0.957	0.043
157820193	Tbx2	T-box transcription factor 2	0.909	-0.967	0.033
564378170	PAN3	poly(A) specific ribonuclease subunit PAN3	0.932	-0.986	0.014
53734355	P2RY14	purinergic receptor P2Y14	1.000	-0.990	0.010
61097928	SNAI1	snail family transcriptional repressor 1	1.053	-0.952	0.048
564306247	PHACTR4	phosphatase and actin regulator 4	1.189	-0.985	0.015
40786461	NAPEPLD	N-acyl phosphatidylethanolamine phospholipase D	1.199	-0.961	0.039
7106248	ANKRD1	ankyrin repeat domain 1	1.252	-0.955	0.045
13928802	CCN5	cellular communication network factor 5	1.322	-0.971	0.029
140969796	Cyp2c23	cytochrome P450, family 2, subfamily c, polypeptide 23	1.363	-0.981	0.019
672084625	LOC100909409 (includes others)	RGD1562660	1.407	-0.979	0.021

56606094	Aox2	aldehyde oxidase 2	1.459	-0.986	0.014
54312124	HCST	hematopoietic cell signal transducer	1.554	-0.975	0.025
23463269	NPB	neuropeptide B	1.609	-0.975	0.025
204595	H1f4	H1.4 linker histone, cluster member	1.688	-0.974	0.026
408407614	DNA2	DNA replication helicase/nuclease 2	1.697	-0.979	0.021
254553399	FBXO24	F-box protein 24	1.716	-0.989	0.011
157786614	MFSD6L	major facilitator superfamily domain containing 6 like	1.716	-0.989	0.011
148230802	Akr1c12	aldo-keto reductase family 1, member C12	1.826	-0.989	0.011
207446700	Sec1	secretory blood group 1	1.826	-0.989	0.011
11024668	AIPL1	aryl hydrocarbon receptor interacting protein like 1	2.188	-0.974	0.026
158508517	SDS	serine dehydratase	2.202	-0.975	0.025
392342139	TTC21A	tetratricopeptide repeat domain 21A	2.389	-0.977	0.023
672071273	GRAMD1C	GRAM domain containing 1C	2.450	-0.957	0.043

Supplementary Table S4. The list of genes that are differentially expressed in the offspring hippocampus in response to prenatal BPA exposure that exhibited the changes in the expression levels correlated with the neuronal density in the hippocampus. The transcriptome profiling data of DEGs in male and female rat offspring prenatally exposed to BPA (n = 6, male pups n = 3 and female pups n = 3, from independent litters) or the vehicle control (n = 6, male pups n = 3 and female pups n = 3, from independent litters) were obtained and used for the PTM analyses to identify DEGs that exhibited the changes in the expression levels correlated with the neuronal density in the hippocampus.

ID	Symbol	Entrez Gene Name	log2(FC)	R values	P-values
291327518	AVP	arginine vasopressin	-2.734	0.955	0.045
149053857	LOC100363423	rCG35357-like	-2.421	0.994	0.006
157822087	ACTRT3	actin related protein T3	-2.170	1.000	0.000
187937026	NCF4	neutrophil cytosolic factor 4	-2.062	0.982	0.018
19923094	OSGIN1	oxidative stress induced growth inhibitor 1	-2.059	0.999	0.001
564388803	SLC18A1	solute carrier family 18 member A1	-2.059	0.999	0.001
675294847	ATP12A	ATPase H+/K+ transporting non-gastric alpha2 subunit	-2.051	0.963	0.037
84781670	MCOLN2	mucolipin 2	-1.939	0.998	0.002
54019438	PCDHAC1	protocadherin alpha subfamily C, 1	-1.704	0.989	0.011
392331668	HAGHL	hydroxyacylglutathione hydrolase like	-1.665	0.961	0.039
158711755	C17orf97	chromosome 17 open reading frame 97	-1.592	0.980	0.020
815890866	HNF1B	HNF1 homeobox B	-1.585	0.960	0.040
564305934	BTBD19	BTB domain containing 19	-1.458	0.993	0.007
119709837	SLC15A1	solute carrier family 15 member 1	-1.364	0.997	0.003
340523096	IL10RA	interleukin 10 receptor subunit alpha	-1.347	0.992	0.008
672034215	ZNF729	zinc finger protein 729	-1.256	0.967	0.033
9910536	RNASE4	ribonuclease A family member 4	-1.245	0.958	0.042
157820267	MEI4	meiotic double-stranded break formation protein 4	-1.205	0.990	0.010
564393142	WDR36	WD repeat domain 36	-1.184	0.950	0.050
149408137	DHX58	DExH-box helicase 58	-1.183	0.969	0.031
564310671	NBEAL2	neurobeachin like 2	-1.084	0.961	0.039
564358455	RASAL3	RAS protein activator like 3	-1.052	0.982	0.018
32189322	ADGRG2	adhesion G protein-coupled receptor G2	-1.027	0.980	0.020
68163493	DAW1	dynein assembly factor with WD repeats 1	-1.005	0.976	0.024
16758344	SEC16B	SEC16 homolog B, endoplasmic reticulum export factor	-0.958	0.976	0.024
157820109	SLC43A1	solute carrier family 43 member 1	-0.950	0.961	0.039

564315207	TMEM270	transmembrane protein 270	-0.933	0.994	0.006
40254742	NCF1	neutrophil cytosolic factor 1	-0.929	0.973	0.027
293347270	OSGIN2	oxidative stress induced growth inhibitor family member 2	-0.915	0.970	0.030
564345430	LOC100910079	actin-related protein 3B-like	-0.913	0.964	0.036
164663906	PDIA2	protein disulfide isomerase family A member 2	-0.909	0.985	0.015
672016875	LOC103690320	FERM and PDZ domain-containing protein 3	-0.897	0.981	0.019
12621098	EPHX2	epoxide hydrolase 2	-0.887	0.981	0.019
148670853	BBOF1	basal body orientation factor 1	-0.878	0.953	0.047
62945324	LCA5L	lebercilin LCA5 like	-0.853	0.985	0.015
119226204	CFAP206	cilia and flagella associated protein 206	-0.849	0.969	0.031
77020254	GPR182	G protein-coupled receptor 182	-0.845	0.981	0.019
227116255	P2RX6	purinergic receptor P2X 6	-0.838	0.998	0.002
564372514	SHBG	sex hormone binding globulin	-0.822	0.983	0.017
564388219	ARHGAP22	Rho GTPase activating protein 22	-0.795	0.984	0.016
18426812	ADA	adenosine deaminase	-0.787	0.953	0.047
157819203	TECTA	tectorin alpha	-0.778	0.993	0.007
818015	HBB	hemoglobin subunit beta	-0.770	0.962	0.038
61097937	VEGFB	vascular endothelial growth factor B	-0.766	0.957	0.043
157822593	NEIL2	nei like DNA glycosylase 2	-0.753	0.962	0.038
149056609	DEDD2	death effector domain containing 2	-0.745	0.980	0.020
157822391	OTOG	otogelin	-0.737	0.966	0.034
76253906	CASP4	caspase 4	-0.722	0.965	0.035
568939712	KCP	kielin cysteine rich BMP regulator	-0.718	0.958	0.042
68341959	CASTOR1	cytosolic arginine sensor for mTORC1 subunit 1	-0.712	0.951	0.049
46485501	CDH15	cadherin 15	-0.681	0.963	0.037
300797913	PALB2	partner and localizer of BRCA2	-0.679	0.956	0.044
293349725	AMER3	APC membrane recruitment protein 3	-0.672	0.975	0.025
281306821	HEY2	hes related family bHLH transcription factor with YRPW motif 2	-0.653	0.952	0.048
149066324	LOC690120	hypothetical protein LOC690120	-0.652	0.968	0.032
564348231	RPUSD3	RNA pseudouridine synthase D3	-0.645	0.994	0.006
31542804	FCGR2A	Fc fragment of IgG receptor IIa	-0.633	0.987	0.013
300797715	NDST4	N-deacetylase and N-sulfotransferase 4	-0.627	0.980	0.020
74203836	PNPLA7	patatin like phospholipase domain containing 7	-0.626	0.983	0.017
157819949	ITGA4	integrin subunit alpha 4	-0.620	0.966	0.034
157821551	EBI3	Epstein-Barr virus induced 3	-0.609	0.980	0.020
157818181	GPR146	G protein-coupled receptor 146	-0.602	0.975	0.025

82654224	IDNK	IDNK gluconokinase	-0.597	0.959	0.041
189230091	MIS18A	MIS18 kinetochore protein A	-0.585	0.986	0.014
83320101	AFG1L	AFG1 like ATPase	-0.583	0.974	0.026
187282394	USP43	ubiquitin specific peptidase 43	-0.577	0.980	0.020
291463305	SHISA9	shisa family member 9	-0.574	0.961	0.039
281182643	ALK	ALK receptor tyrosine kinase	-0.565	0.955	0.045
149048141	PMF1/PMF1-BGLAP	polyamine modulated factor 1	-0.565	1.000	0.000
61889110	OSTF1	osteoclast stimulating factor 1	-0.563	0.951	0.049
120474989	KRT1	keratin 1	-0.563	0.986	0.014
157820141	KLHDC1	kelch domain containing 1	-0.546	0.993	0.007
56676356	SLC10A4	solute carrier family 10 member 4	-0.520	0.964	0.036
17105368	KLF9	Kruppel like factor 9	-0.505	0.976	0.024
9506709	GALR2	galanin receptor 2	-0.500	0.988	0.012
12738847	MERTK	MER proto-oncogene, tyrosine kinase	-0.488	0.952	0.048
157817033	TJAP1	tight junction associated protein 1	-0.487	0.971	0.029
672084625	LOC100909409 (includes others)	RGD1562660	-0.487	0.982	0.018
157786612	B9D1	B9 domain containing 1	-0.482	0.964	0.036
564306247	PHACTR4	phosphatase and actin regulator 4	-0.477	0.997	0.003
149032914	MOXD1	monooxygenase DBH like 1	-0.469	0.995	0.005
219281893	ZNF583	zinc finger protein 583	-0.467	0.966	0.034
157822891	ADCK2	aarF domain containing kinase 2	-0.465	0.983	0.017
157823151	DLEU7	deleted in lymphocytic leukemia 7	-0.459	0.989	0.011
293346302	FBLN7	fibulin 7	-0.448	0.982	0.018
201066363	LOXL2	lysyl oxidase like 2	-0.435	0.982	0.018
210031518	MOGAT2	monoacylglycerol O-acyltransferase 2	-0.432	0.970	0.030
11560087	PYGL	glycogen phosphorylase L	-0.431	0.993	0.007
110347493	PCDHA9	protocadherin alpha 9	-0.429	0.989	0.011
672089660	ZNF280B	zinc finger protein 280B	-0.426	0.954	0.046
62078447	HBA1/HBA2	hemoglobin subunit alpha 2	-0.420	0.989	0.011
157823313	KANK3	KN motif and ankyrin repeat domains 3	-0.419	0.951	0.049
194473652	TTC38	tetratricopeptide repeat domain 38	-0.413	0.960	0.040
157819765	OGDHL	oxoglutarate dehydrogenase like	-0.413	0.996	0.004
187469604	EPS8L2	EPS8 like 2	-0.412	0.960	0.040
148697062	TMEM255A	transmembrane protein 255A	-0.408	0.957	0.043
58865490	LCMT2	leucine carboxyl methyltransferase 2	-0.407	0.980	0.020
188536090	FAM241B	family with sequence similarity 241 member B	-0.400	0.996	0.004
9507041	RESP18	regulated endocrine specific protein 18	-0.399	0.983	0.017
312836782	MRPS27	mitochondrial ribosomal protein S27	-0.395	0.980	0.020
77628031	SP110	SP110 nuclear body protein	-0.391	0.980	0.020

157786698	RDM1	RAD52 motif containing 1	-0.383	0.982	0.018
149042939	WFDC2	WAP four-disulfide core domain 2	-0.373	0.962	0.038
8393919	LOC100911216/Pcsk1	proprotein convertase subtilisin/kexin type 1	-0.371	0.980	0.020
26024221	PRSS12	serine protease 12	-0.366	0.964	0.036
66730351	C11orf16	chromosome 11 open reading frame 16	-0.364	0.951	0.049
296470851	PABPC1L2A	poly(A) binding protein cytoplasmic 1 like 2A	-0.359	0.953	0.047
40786491	CYP20A1	cytochrome P450 family 20 subfamily A member 1	-0.358	0.951	0.049
157822187	WWOX	WW domain containing oxidoreductase	-0.355	0.961	0.039
568918806	TP53RK	TP53 regulating kinase	-0.354	0.977	0.023
158749582	MST1R	macrophage stimulating 1 receptor	-0.349	0.995	0.005
672015093	VWA2	von Willebrand factor A domain containing 2	-0.344	0.994	0.006
50511312	POFUT1	protein O-fucosyltransferase 1	-0.343	0.975	0.025
149027325	DNAJC21	DnaJ heat shock protein family (Hsp40) member C21	-0.339	0.996	0.004
57527498	KLC4	kinesin light chain 4	-0.331	0.978	0.022
300795285	Cyp4f17	cytochrome P450, family 4, subfamily f, polypeptide 17	-0.330	0.974	0.026
19424260	CDC25B	cell division cycle 25B	-0.317	0.993	0.007
443940	CCNE1	cyclin E1	-0.316	0.976	0.024
139948516	MANEA	mannosidase endo-alpha	-0.316	0.995	0.005
70794782	RBMS2	RNA binding motif single stranded interacting protein 2	-0.316	0.971	0.029
672080674	Myo16	myosin XVI	-0.315	0.978	0.022
16758656	BAK1	BCL2 antagonist/killer 1	-0.305	0.989	0.011
157819589	BOLA3	bolA family member 3	-0.303	0.999	0.001
149030301	PNOC	prepronociceptin	-0.298	0.969	0.031
83816933	AP4M1	adaptor related protein complex 4 subunit mu 1	-0.291	0.982	0.018
672065746	SCLY	selenocysteine lyase	-0.291	0.994	0.006
672083256	MYO5B	myosin VB	-0.287	0.956	0.044
6754024	GNG4	G protein subunit gamma 4	-0.284	0.974	0.026
189181708	RWDD2B	RWD domain containing 2B	-0.283	0.981	0.019
157818005	HPS3	HPS3 biogenesis of lysosomal organelles complex 2 subunit 1	-0.277	0.997	0.003
62079057	IL33	interleukin 33	-0.273	0.975	0.025
157821645	RBIS	ribosomal biogenesis factor	-0.270	0.979	0.021
564338026	BCL9	BCL9 transcription coactivator	-0.265	0.950	0.050
205294	ME1	malic enzyme 1	-0.264	0.990	0.010

157821975	ZCCHC24	zinc finger CCHC-type containing 24	-0.263	0.994	0.006
148696365	AP5S1	adaptor related protein complex 5 subunit sigma 1	-0.262	0.976	0.024
157817017	MRPS16	mitochondrial ribosomal protein S16	-0.261	0.967	0.033
18266702	BNIP1	BCL2 interacting protein 1	-0.258	0.962	0.038
148700512	NRSN1	neurensin 1	-0.256	0.968	0.032
51948504	KNSTRN	kinetochore localized astrin (SPAG5) binding protein	-0.254	0.974	0.026
564341663	SLC43A3	solute carrier family 43 member 3	-0.254	0.964	0.036
31560385	RPL21	ribosomal protein L21	-0.252	0.963	0.037
568959109	CEP164	centrosomal protein 164	-0.250	0.959	0.041
149046389	ARID5A	AT-rich interaction domain 5A	-0.249	0.960	0.040
298493223	TMEM132B	transmembrane protein 132B	-0.246	0.987	0.013
300794684	MSH3	mutS homolog 3	-0.244	0.995	0.005
564369492	HJURP	Holliday junction recognition protein	-0.243	0.983	0.017
25453420	GSTP1	glutathione S-transferase pi 1	-0.241	0.953	0.047
236467366	CGREF1	cell growth regulator with EF-hand domain 1	-0.241	0.985	0.015
157822599	GSAP	gamma-secretase activating protein	-0.237	0.974	0.026
449784888	ALDH5A1	aldehyde dehydrogenase 5 family member A1	-0.236	0.963	0.037
160333179	FBXO27	F-box protein 27	-0.236	0.972	0.028
157817480	RWDD2A	RWD domain containing 2A	-0.235	0.955	0.045
564358836	NT5DC3	5'-nucleotidase domain containing 3	-0.232	0.983	0.017
197927216	TBC1D5	TBC1 domain family member 5	-0.230	0.975	0.025
73661200	SPRN	shadow of prion protein	-0.229	0.971	0.029
404434384	GALNT11	polypeptide N-acetylgalactosaminyltransferase 11	-0.229	0.979	0.021
392334157	SEMA6A	semaphorin 6A	-0.228	0.978	0.022
149033235	PYURF	PIGY upstream reading frame	-0.227	0.985	0.015
13592117	KLF10	Kruppel like factor 10	-0.225	0.977	0.023
157818283	UQCC2	ubiquinol-cytochrome c reductase complex assembly factor 2	-0.222	0.955	0.045
288541382	DIS3L2	DIS3 like 3'-5' exoribonuclease 2	-0.214	0.998	0.002
205755	TAGLN3	transgelin 3	-0.213	0.977	0.023
157786612	B9D1	B9 domain containing 1	-0.211	0.994	0.006
564386387	EEF1AKMT1	EEF1A lysine methyltransferase 1	-0.209	0.961	0.039
398303848	RNH1	ribonuclease/angiogenin inhibitor 1	-0.207	0.980	0.020
13540703	PDE1A	phosphodiesterase 1A	-0.205	0.993	0.007
18426866	ACAA2	acetyl-CoA acyltransferase 2	-0.203	0.974	0.026
451770389	HMGCLL1	3-hydroxymethyl-3-methylglutaryl-CoA lyase like 1	-0.202	0.983	0.017
309243082	PTPRJ	protein tyrosine phosphatase receptor type J	-0.201	0.969	0.031

149033449	RGD1559747	similar to Zinc finger and SCAN domain containing protein 2 (Zinc finger protein 29)	-0.200	0.999	0.001
451172098	KCTD1	potassium channel tetramerization domain containing 1	-0.197	0.965	0.035
123780073	YOD1	YOD1 deubiquitinase	-0.193	0.955	0.045
62078931	PAQR8	progestin and adipoQ receptor family member 8	-0.192	0.952	0.048
13591963	GRM7	glutamate metabotropic receptor 7	-0.191	0.951	0.049
293349793	RFTN2	raftlin family member 2	-0.189	0.999	0.001
42491372	ERMP1	endoplasmic reticulum metallopeptidase 1	-0.189	1.000	0.000
6981112	IVD	isovaleryl-CoA dehydrogenase	-0.189	0.956	0.044
564342864	FAHD2B	fumarylacetoacetate hydrolase domain containing 2B	-0.189	0.994	0.006
19705437	EPHA7	EPH receptor A7	-0.186	0.959	0.041
392343399	COL4A6	collagen type IV alpha 6 chain	-0.182	0.981	0.019
194240569	TRMT44	tRNA methyltransferase 44 homolog	-0.174	0.990	0.010
672062144	FAM219B	family with sequence similarity 219 member B	-0.174	0.982	0.018
124248495	CHID1	chitinase domain containing 1	-0.171	0.959	0.041
300797936	ACTR3B	actin related protein 3B	-0.171	0.997	0.003
269954719	JAZF1	JAZF zinc finger 1	-0.171	0.958	0.042
148682229	PDZD11	PDZ domain containing 11	-0.171	0.953	0.047
209529691	TMCO3	transmembrane and coiled-coil domains 3	-0.169	0.956	0.044
348041331	SERTAD2	SERTA domain containing 2	-0.169	0.971	0.029
58219062	CNDP2	carnosine dipeptidase 2	-0.168	0.999	0.001
79750129	CAMK1D	calcium/calmodulin dependent protein kinase ID	-0.164	0.957	0.043
197384515	UAP1L1	UDP-N-acetylglucosamine pyrophosphorylase 1 like 1	-0.163	0.958	0.042
76096352	ALDH16A1	aldehyde dehydrogenase 16 family member A1	-0.160	0.983	0.017
154146247	TMEM38A	transmembrane protein 38A	-0.159	0.969	0.031
62078637	LCA5	lebercilin LCA5	-0.158	0.965	0.035
564397835	ASCC1	activating signal cointegrator 1 complex subunit 1	-0.158	0.977	0.023
74201328	ST6GALNAC4	ST6 N-acetylgalactosaminide alpha-2,6-sialyltransferase 4	-0.158	0.962	0.038
564324344	LOC363306 (includes others)	hypothetical protein LOC363306	-0.154	0.954	0.046
6978765	DLX5	distal-less homeobox 5	-0.154	0.972	0.028
195976798	SLBP	stem-loop binding protein	-0.153	0.984	0.016

8394496	TYRO3	TYRO3 protein tyrosine kinase	-0.152	0.985	0.015
149024719	NOL9	nucleolar protein 9	-0.150	0.978	0.022
149032539	HECW1	HECT, C2 and WW domain containing E3 ubiquitin protein ligase 1	-0.150	0.970	0.030
755498773	ITGA6	integrin subunit alpha 6	-0.149	0.961	0.039
8394227	PTPRO	protein tyrosine phosphatase receptor type O	-0.148	0.982	0.018
84781723	TRAP1	TNF receptor associated protein 1	-0.148	0.958	0.042
755553984	THAP7	THAP domain containing 7	-0.147	0.956	0.044
564364792	SNAP91	synaptosome associated protein 91	-0.147	0.959	0.041
149064661	PLCH1	phospholipase C eta 1	-0.146	0.962	0.038
71122474	PPA1	inorganic pyrophosphatase 1	-0.142	0.965	0.035
157822025	NDOR1	NADPH dependent diflavin oxidoreductase 1	-0.142	0.975	0.025
148671875	TMEM50B	transmembrane protein 50B	-0.142	0.964	0.036
148692881	OXNAD1	oxidoreductase NAD binding domain containing 1	-0.140	1.000	0.000
66730507	CCDC134	coiled-coil domain containing 134	-0.140	0.990	0.010
281306811	ATP4A	ATPase H+/K+ transporting subunit alpha	-0.140	0.986	0.014
77627979	SRPRA	SRP receptor subunit alpha	-0.138	0.967	0.033
564389848	ERICH1	glutamate rich 1	-0.137	0.972	0.028
68163557	CDCA8	cell division cycle associated 8	-0.136	0.998	0.002
53850598	DDX59	DEAD-box helicase 59	-0.134	0.972	0.028
62078739	TCTA	T cell leukemia translocation altered	-0.131	0.952	0.048
157822423	LRSAM1	leucine rich repeat and sterile alpha motif containing 1	-0.121	0.968	0.032
274325671	SCAMP3	secretory carrier membrane protein 3	-0.119	0.972	0.028
67078458	CRELD1	cysteine rich with EGF like domains 1	-0.115	0.959	0.041
160333166	Pid1	phosphotyrosine interaction domain containing 1	-0.113	0.989	0.011
349501010	FAM120B	family with sequence similarity 120B	-0.110	0.999	0.001
51948408	FUCA2	alpha-L-fucosidase 2	-0.107	0.956	0.044
148673176	FABP7	fatty acid binding protein 7	-0.107	0.966	0.034
149040289	ARMH3	armadillo like helical domain containing 3	-0.106	0.974	0.026
19424310	SLC31A1	solute carrier family 31 member 1	-0.106	0.983	0.017
157821419	ARMH4	armadillo like helical domain containing 4	-0.101	0.956	0.044
157821685	ABL2	ABL proto-oncogene 2, non-receptor tyrosine kinase	-0.099	0.976	0.024
40254595	DPYSL2	dihydropyrimidinase like 2	-0.096	0.993	0.007

253970439	KCNA6	potassium voltage-gated channel subfamily A member 6	-0.096	0.979	0.021
224549858	Poldip2	DNA polymerase delta interacting protein 2	-0.090	0.985	0.015
6981370	PLCG1	phospholipase C gamma 1	-0.088	0.952	0.048
195927000	DLST	dihydrolipoamide S-succinyltransferase	-0.084	0.983	0.017
564380332	R3HDM1	R3H domain containing 1	-0.079	0.972	0.028
564343911	RPN2	ribophorin II	-0.076	0.956	0.044
189011661	PELI3	pellino E3 ubiquitin protein ligase family member 3	-0.071	0.960	0.040
564328458	Ldha/RGD1562690	lactate dehydrogenase A	-0.064	0.976	0.024
300794891	DDX20	DEAD-box helicase 20	-0.060	0.957	0.043
18543177	CS	citrate synthase	-0.059	0.951	0.049
213688386	GTF2E1	general transcription factor IIE subunit 1	-0.058	0.956	0.044
157821073	IPO9	importin 9	-0.058	0.969	0.031
77627990	ATP6V0A1	ATPase H+ transporting V0 subunit a1	-0.052	0.977	0.023
67078460	PRKRA	protein activator of interferon induced protein kinase EIF2AK2	-0.051	0.995	0.005
157821319	ATP6V0B	ATPase H+ transporting V0 subunit b	-0.045	0.991	0.009
149045755	CREB3	cAMP responsive element binding protein 3	-0.043	0.967	0.033
157819901	STAM	signal transducing adaptor molecule	-0.031	0.976	0.024
149047197	STIM2	stromal interaction molecule 2	-0.023	0.994	0.006
157818483	GSPT2	G1 to S phase transition 2	0.024	-0.976	0.024
300798184	GRAMD1B	GRAM domain containing 1B	0.049	-0.956	0.044
20544149	CSNK1D	casein kinase 1 delta	0.053	-0.956	0.044
50511039	GNB1L	G protein subunit beta 1 like	0.054	-0.961	0.039
157786896	FIS1	fission, mitochondrial 1	0.073	-0.992	0.008
11120706	PLRG1	pleiotropic regulator 1	0.075	-0.955	0.045
157822653	CD2BP2	CD2 cytoplasmic tail binding protein 2	0.075	-0.996	0.004
51854215	RNF34	ring finger protein 34	0.080	-0.956	0.044
564387037	COG3	component of oligomeric golgi complex 3	0.091	-0.990	0.010
241666404	EPHA4	EPH receptor A4	0.100	-0.988	0.012
564368059	KANSL3	KAT8 regulatory NSL complex subunit 3	0.103	-0.961	0.039
62543537	TBC1D10A	TBC1 domain family member 10A	0.108	-0.954	0.046
157822563	AREL1	apoptosis resistant E3 ubiquitin protein ligase 1	0.109	-0.998	0.002

564320563	RBM27	RNA binding motif protein 27	0.109	-0.994	0.006
686661085	MTMR4	myotubularin related protein 4	0.111	-0.964	0.036
30794434	SRRM4	serine/arginine repetitive matrix 4	0.112	-0.953	0.047
157817811	C5orf22	chromosome 5 open reading frame 22	0.113	-0.960	0.040
170295834	NDUFA10	NADH:ubiquinone oxidoreductase subunit A10	0.114	-0.955	0.045
293347618	RLF	RLF zinc finger	0.117	-0.996	0.004
403224979	PPP4R3A	protein phosphatase 4 regulatory subunit 3A	0.118	-0.955	0.045
66730484	TAOK3	TAO kinase 3	0.121	-0.982	0.018
672017866	DIDO1	death inducer-obliterator 1	0.123	-0.981	0.019
672060579	SENP1	SUMO specific peptidase 1	0.123	-0.986	0.014
392347634	CHD4	chromodomain helicase DNA binding protein 4	0.124	-0.998	0.002
564361462	BRD1	bromodomain containing 1	0.130	-0.971	0.029
672042705	FNIP2	folliculin interacting protein 2	0.130	-0.982	0.018
157821015	KDM5B	lysine demethylase 5B	0.131	-0.956	0.044
564333160	CEMIP2	cell migration inducing hyaluronidase 2	0.133	-0.951	0.049
11177894	TSC1	TSC complex subunit 1	0.134	-0.965	0.035
162287067	VAV1	vav guanine nucleotide exchange factor 1	0.135	-0.996	0.004
213972547	KAT6A	lysine acetyltransferase 6A	0.143	-0.988	0.012
6981458	RAF1	Raf-1 proto-oncogene, serine/threonine kinase	0.144	-0.986	0.014
12408292	PGR	progesterone receptor	0.144	-0.990	0.010
291042494	MED13L	mediator complex subunit 13L	0.145	-0.960	0.040
966923099	SMG7	SMG7 nonsense mediated mRNA decay factor	0.146	-0.992	0.008
300798201	PYGO1	pygopus family PHD finger 1	0.147	-0.951	0.049
62079033	PRMT7	protein arginine methyltransferase 7	0.148	-0.963	0.037
61556879	PKNOX1	PBX/knotted 1 homeobox 1	0.148	-0.956	0.044
157819423	SPSB3	splA/ryanodine receptor domain and SOCS box containing 3	0.150	-0.957	0.043
157820079	C14orf28	chromosome 14 open reading frame 28	0.152	-0.997	0.003
157820865	DDX28	DEAD-box helicase 28	0.153	-0.987	0.013
2088637	EED	embryonic ectoderm development	0.155	-0.981	0.019
50511177	SLITRK1	SLIT and NTRK like family member 1	0.155	-0.970	0.030
157820917	CDC7	cell division cycle 7	0.156	-0.958	0.042
157822569	Tubgcp3	tubulin, gamma complex associated protein 3	0.156	-0.983	0.017
300798312	POLR3B	RNA polymerase III subunit B	0.158	-0.990	0.010

74200325	UBE2G2	ubiquitin conjugating enzyme E2 G2	0.158	-0.957	0.043
19705483	CLSTN2	calsyntenin 2	0.160	-0.966	0.034
19924085	FAT3	FAT atypical cadherin 3	0.161	-0.968	0.032
114145788	NAA25	N-alpha-acetyltransferase 25, NatB auxiliary subunit	0.162	-0.980	0.020
8393104	CHKB	choline kinase beta	0.166	-0.984	0.016
46485387	NAPRT	nicotinate phosphoribosyltransferase	0.167	-0.972	0.028
392333169	CCDC88A	coiled-coil domain containing 88A	0.168	-0.967	0.033
564392297	SLC39A12	solute carrier family 39 member 12	0.170	-0.992	0.008
16758536	AATF	apoptosis antagonizing transcription factor	0.171	-0.988	0.012
40807349	DSTYK	dual serine/threonine and tyrosine protein kinase	0.172	-0.975	0.025
300794761	Fat4	FAT atypical cadherin 4	0.173	-0.974	0.026
568972665	TSPOAP1	TSPO associated protein 1	0.173	-0.950	0.050
148806879	DNTTIP1	deoxynucleotidyltransferase terminal interacting protein 1	0.174	-0.999	0.001
564298823	EML3	EMAP like 3	0.177	-0.970	0.030
164565360	CTTNBP2NL	CTTNBP2 N-terminal like	0.180	-0.965	0.035
38454226	TPD52L2	TPD52 like 2	0.180	-0.963	0.037
9506755	GRIK2	glutamate ionotropic receptor kainate type subunit 2	0.181	-0.990	0.010
157822303	GPR107	G protein-coupled receptor 107	0.182	-0.955	0.045
28076889	YIPF4	Yip1 domain family member 4	0.183	-0.959	0.041
392342217	RANBP3	RAN binding protein 3	0.184	-0.974	0.026
157818115	PATL1	PAT1 homolog 1, processing body mRNA decay factor	0.187	-0.999	0.001
403310664	KMT2E	lysine methyltransferase 2E (inactive)	0.194	-0.962	0.038
62078579	NUB1	negative regulator of ubiquitin like proteins 1	0.195	-0.967	0.033
157822191	MTMR2	myotubularin related protein 2	0.195	-0.975	0.025
157821415	GZF1	GDNF inducible zinc finger protein 1	0.201	-0.958	0.042
149057558	ABHD13	abhydrolase domain containing 13	0.203	-0.980	0.020
148674145	CHMP4B	charged multivesicular body protein 4B	0.203	-0.992	0.008
672031398	ANKRD11	ankyrin repeat domain 11	0.203	-0.960	0.040
27229022	2610002M06Rik	RIKEN cDNA 2610002M06 gene	0.209	-0.998	0.002
149030718	PIP5K1A	phosphatidylinositol-4-phosphate 5-kinase type 1 alpha	0.211	-0.961	0.039
392340953	ITSN2	intersectin 2	0.214	-0.966	0.034
66911118	NFX1	nuclear transcription factor, X-box binding 1	0.217	-0.957	0.043
56090383	TMEM43	transmembrane protein 43	0.218	-0.978	0.022

281604112	BAZ1A	bromodomain adjacent to zinc finger domain 1A	0.219	-0.987	0.013
149045074	NUP153	nucleoporin 153	0.219	-0.975	0.025
13027450	SENP2	SUMO specific peptidase 2	0.220	-0.971	0.029
157820985	NCK1	NCK adaptor protein 1	0.221	-0.987	0.013
71051128	ANKRD10	ankyrin repeat domain 10	0.222	-0.997	0.003
293340917	C3orf70	chromosome 3 open reading frame 70	0.223	-0.962	0.038
125988381	JMJD6	jumonji domain containing 6, arginine demethylase and lysine hydroxylase	0.223	-0.970	0.030
51871603	ST7	suppression of tumorigenicity 7	0.225	-0.987	0.013
189181698	ZNF131	zinc finger protein 131	0.225	-0.954	0.046
300793975	ZNF286A	zinc finger protein 286A	0.228	-0.994	0.006
84781676	MTRR	5-methyltetrahydrofolate-homocysteine methyltransferase reductase	0.231	-0.997	0.003
76096324	ORC6	origin recognition complex subunit 6	0.233	-0.994	0.006
149032040	SLC11A2	solute carrier family 11 member 2	0.234	-0.962	0.038
404351667	BOD1	biorientation of chromosomes in cell division 1	0.234	-0.978	0.022
56676358	IPPK	inositol-pentakisphosphate 2-kinase	0.235	-0.953	0.047
149067780	MVP	major vault protein	0.236	-0.990	0.010
67078478	NAF1	nuclear assembly factor 1 ribonucleoprotein	0.237	-0.957	0.043
121583782	ZNF426	zinc finger protein 426	0.237	-0.994	0.006
13928816	EIF2AK3	eukaryotic translation initiation factor 2 alpha kinase 3	0.239	-0.993	0.007
149023044	TMEM87A	transmembrane protein 87A	0.241	-0.987	0.013
300794867	RSBN1	round spermatid basic protein 1	0.241	-0.965	0.035
157821413	USP30	ubiquitin specific peptidase 30	0.250	-0.951	0.049
564354018	DFFA	DNA fragmentation factor subunit alpha	0.251	-0.955	0.045
256818763	PLEKHH1	pleckstrin homology, MyTH4 and FERM domain containing H1	0.252	-0.971	0.029
149063273	MPHOSPH9	M-phase phosphoprotein 9	0.256	-1.000	0.000
148666908	ADAMTS9	ADAM metallopeptidase with thrombospondin type 1 motif 9	0.256	-0.963	0.037
564328896	CHD2	chromodomain helicase DNA binding protein 2	0.258	-0.998	0.002
157820043	ZKSCAN5	zinc finger with KRAB and SCAN domains 5	0.267	-0.998	0.002
56090325	PACC1	proton activated chloride channel 1	0.269	-0.961	0.039
564365504	CCDC51	coiled-coil domain containing 51	0.277	-0.979	0.021
109464919	ARHGEF26	Rho guanine nucleotide exchange factor 26	0.278	-0.980	0.020

194474032	RNF19A	ring finger protein 19A, RBR E3 ubiquitin protein ligase	0.278	-0.995	0.005
197386048	PTRHD1	peptidyl-tRNA hydrolase domain containing 1	0.280	-0.967	0.033
315259095	UBN1	ubinuclein 1	0.281	-0.980	0.020
281599335	BMS1	BMS1 ribosome biogenesis factor	0.286	-0.959	0.041
392334341	USP38	ubiquitin specific peptidase 38	0.289	-0.995	0.005
157822011	TGS1	trimethylguanosine synthase 1	0.291	-0.975	0.025
586597897	DBP	D-box binding PAR bZIP transcription factor	0.292	-0.980	0.020
204744	IGFBP3	insulin like growth factor binding protein 3	0.292	-0.990	0.010
145553978	SFMBT1	Scm like with four mbt domains 1	0.297	-0.962	0.038
51948532	TBC1D20	TBC1 domain family member 20	0.302	-0.975	0.025
293349725	AMER3	APC membrane recruitment protein 3	0.303	-0.973	0.027
6680532	KCNJ3	potassium inwardly rectifying channel subfamily J member 3	0.308	-0.988	0.012
72255513	AGA	aspartylglucosaminidase	0.309	-0.956	0.044
50510821	AMIGO1	adhesion molecule with Ig like domain 1	0.310	-0.959	0.041
33086606	SRPRB	SRP receptor subunit beta	0.318	-0.966	0.034
148681991	MIOS	meiosis regulator for oocyte development	0.325	-0.988	0.012
157820341	GPR63	G protein-coupled receptor 63	0.326	-0.979	0.021
404501489	DDX55	DEAD-box helicase 55	0.329	-0.979	0.021
672063138	IP6K2	inositol hexakisphosphate kinase 2	0.332	-0.980	0.020
157820863	RPIA	ribose 5-phosphate isomerase A	0.336	-0.968	0.032
672055128	SAMD11	sterile alpha motif domain containing 11	0.342	-0.985	0.015
300794780	GPSM2	G protein signaling modulator 2	0.342	-0.964	0.036
77917610	GPBP1L1	GC-rich promoter binding protein 1 like 1	0.344	-0.974	0.026
160333172	COG2	component of oligomeric golgi complex 2	0.346	-0.999	0.001
672067227	ZNF354A	zinc finger protein 354A	0.351	-0.986	0.014
149067193	Cfap54	cilia and flagella associated protein 54	0.351	-0.970	0.030
157821859	OBI1	ORC ubiquitin ligase 1	0.352	-0.958	0.042
157822711	RBM28	RNA binding motif protein 28	0.353	-0.990	0.010
56119156	Zfp58	zinc finger protein 58	0.353	-0.958	0.042
672065395	CCNYL1	cyclin Y like 1	0.356	-0.989	0.011
564393142	WDR36	WD repeat domain 36	0.359	-0.968	0.032
74183022	Zfp773	zinc finger protein 773	0.360	-0.973	0.027
319996608	Spin2c	spindlin family, member 2C	0.363	-0.985	0.015
62642955	NIM1K	NIM1 serine/threonine protein kinase	0.364	-0.992	0.008

149067053	LRRIQ1	leucine rich repeats and IQ motif containing 1	0.370	-0.990	0.010
148704240	ZMYM2	zinc finger MYM-type containing 2	0.371	-0.992	0.008
16758666	TIMP1	TIMP metallopeptidase inhibitor 1	0.376	-0.963	0.037
157822681	EFNB2	ephrin B2	0.379	-0.980	0.020
564338579	SASS6	SAS-6 centriolar assembly protein	0.381	-0.975	0.025
564323985	LOC108348337	uncharacterized LOC108348337	0.382	-0.988	0.012
56605820	TENT2	terminal nucleotidyltransferase 2	0.387	-0.996	0.004
51948522	PLA2G15	phospholipase A2 group XV	0.404	-0.961	0.039
564318578	ANKRD28	ankyrin repeat domain 28	0.424	-0.976	0.024
188595675	RFX7	regulatory factor X7	0.426	-0.982	0.018
755537242	CLK4	CDC like kinase 4	0.456	-0.973	0.027
8392993	BMP3	bone morphogenetic protein 3	0.461	-0.965	0.035
157822327	ATG14	autophagy related 14	0.464	-0.993	0.007
564347830	ZXDC	ZXD family zinc finger C	0.468	-0.998	0.002
76096320	USHBP1	USH1 protein network component harmonin binding protein 1	0.504	-0.985	0.015
672084062	TEPP	testis, prostate and placenta expressed	0.510	-0.963	0.037
77627983	MLX	MAX dimerization protein MLX	0.531	-0.997	0.003
564320728	Fbxo38	F-box protein 38	0.537	-0.965	0.035
306482607	DENND2C	DENN domain containing 2C	0.547	-0.956	0.044
568916876	GREM1	gremlin 1, DAN family BMP antagonist	0.559	-0.992	0.008
62078827	CTDSPL2	CTD small phosphatase like 2	0.570	-0.980	0.020
564315183	CUX1	cut like homeobox 1	0.585	-0.980	0.020
564386624	AMER2	APC membrane recruitment protein 2	0.591	-0.969	0.031
564304579	ATF7IP	activating transcription factor 7 interacting protein	0.591	-0.992	0.008
148683584	VEPH1	ventricular zone expressed PH domain containing 1	0.603	-0.951	0.049
148702599	UNK	unk zinc finger	0.619	-0.971	0.029
187957728	FANCM	FA complementation group M	0.625	-0.961	0.039
198386330	CCDC89	coiled-coil domain containing 89	0.630	-0.988	0.012
157817525	LGR5	leucine rich repeat containing G protein-coupled receptor 5	0.772	-0.986	0.014
568921554	Ank2	ankyrin 2, brain	0.813	-0.998	0.002
293348634	LRIG3	leucine rich repeats and immunoglobulin like domains 3	0.825	-0.951	0.049
189011634	ARMC7	armadillo repeat containing 7	0.850	-0.955	0.045
6978657	CHRNB1	cholinergic receptor nicotinic beta 1 subunit	0.865	-0.994	0.006
78097110	N4BP2L1	NEDD4 binding protein 2 like 1	0.885	-0.991	0.009

672015368	MAST4	microtubule associated serine/threonine kinase family member 4	0.909	-0.963	0.037
149035338	APBB2	amyloid beta precursor protein binding family B member 2	1.000	-0.981	0.019
61097928	SNAI1	snail family transcriptional repressor 1	1.053	-0.980	0.020
157818609	NT5C1A	5'-nucleotidase, cytosolic IA	1.075	-0.967	0.033
7242211	TRH	thyrotropin releasing hormone	1.113	-0.993	0.007
11596857	KCNE3	potassium voltage-gated channel subfamily E regulatory subunit 3	1.222	-1.000	0.000
11024678	Dbil5	diazepam binding inhibitor-like 5	1.423	-0.982	0.018
350534944	STAB2	stabilin 2	1.459	-0.990	0.010
84000579	FTL	ferritin light chain	1.478	-0.993	0.007
155369646	AGBL4	ATP/GTP binding protein like 4	1.510	-0.960	0.040
6981536	SI	sucrase-isomaltase	1.585	-0.997	0.003
23463269	NPB	neuropeptide B	1.609	-0.952	0.048
204595	H1f4	H1.4 linker histone, cluster member	1.688	-0.961	0.039
408407614	DNA2	DNA replication helicase/nuclease 2	1.697	-0.953	0.047
149020581	ZNF560	zinc finger protein 560	1.703	-0.954	0.046
157818127	CA7	carbonic anhydrase 7	1.824	-0.975	0.025
755495595	PRRC2C	proline rich coiled-coil 2C	1.981	-0.988	0.012
109480102	SMARCC2	SWI/SNF related, matrix associated, actin dependent regulator of chromatin subfamily c member 2	2.178	-0.985	0.015
564329920	EMSY	EMSY transcriptional repressor, BRCA2 interacting	2.185	-0.995	0.005
672071273	GRAMD1C	GRAM domain containing 1C	2.450	-0.956	0.044
293356488	RIC1	RIC1 homolog, RAB6A GEF complex partner 1	2.479	-0.999	0.001
149020413	Zfp599	zinc finger protein 599	3.158	-0.987	0.013

Supplementary Table S5. The list of genes that are differentially expressed in the offspring hippocampus in response to prenatal BPA exposure that exhibited the changes in the expression levels correlated with the neuronal density in CA1 of the hippocampus. The transcriptome profiling data of DEGs in male and female rat offspring prenatally exposed to BPA (n = 6, male pups n = 3 and female pups n = 3, from independent litters) or the vehicle control (n = 6, male pups n = 3 and female pups n = 3, from independent litters) were obtained and used for the PTM analyses to identify DEGs that exhibited the changes in the expression levels correlated with the neuronal density in CA1 of the hippocampus.

ID	Symbol	Entrez Gene Name	log2(FC)	R values	P-values
226698394	UNC80	unc-80 homolog, NALCN channel complex subunit	-1.781	0.967	0.033
300796107	PROX2	prospero homeobox 2	-1.700	0.952	0.048
392331668	HAGHL	hydroxyacylglutathione hydrolase like	-1.665	0.988	0.012
155369702	ECHDC3	enoyl-CoA hydratase domain containing 3	-1.497	0.984	0.016
149020413	Zfp599	zinc finger protein 599	-1.342	0.983	0.017
564312627	ZFP62	ZFP62 zinc finger protein	-1.253	0.970	0.030
157819313	RGD1561661	similar to Ferritin light chain (Ferritin L subunit)	-1.206	0.976	0.024
157824077	CCRL2	C-C motif chemokine receptor like 2	-1.206	0.976	0.024
157819399	NOXO1	NADPH oxidase organizer 1	-1.206	0.976	0.024
149408137	DHX58	DExH-box helicase 58	-1.183	0.969	0.031
58865684	MCOLN3	mucolipin 3	-1.158	0.984	0.016
406035319	KIRREL2	kirre like nephrin family adhesion molecule 2	-1.154	0.974	0.026
564311678	PLEKHM3	pleckstrin homology domain containing M3	-1.152	0.988	0.012
392331829	ATAD5	ATPase family AAA domain containing 5	-1.122	0.974	0.026
392342053	GK5	glycerol kinase 5	-1.000	0.952	0.048
198386353	MYO1G	myosin IG	-1.000	0.987	0.013
157819465	CLEC9A	C-type lectin domain containing 9A	-0.967	0.964	0.036
564349125	TEAD4	TEA domain transcription factor 4	-0.967	0.965	0.035
157819701	Ctla2a	cytotoxic T lymphocyte-associated protein 2 alpha	-0.966	0.992	0.008
24638442	RLN3	relaxin 3	-0.949	0.965	0.035
124486586	AUTS2	activator of transcription and developmental regulator AUTS2	-0.941	0.994	0.006
16758094	FABP4	fatty acid binding protein 4	-0.933	0.965	0.035
293347270	OSGIN2	oxidative stress induced growth inhibitor family member 2	-0.915	0.977	0.023
47576123	Olr1387/Olr1388	olfactory receptor 1387	-0.893	0.965	0.035

157822605	OTOR	otoraplin	-0.893	0.965	0.035
148670853	BBOF1	basal body orientation factor 1	-0.878	0.968	0.032
672036437	ACP4	acid phosphatase 4	-0.862	0.965	0.035
672022227	RGS22	regulator of G protein signaling 22	-0.855	0.992	0.008
6978791	EDN1	endothelin 1	-0.850	0.982	0.018
77020254	GPR182	G protein-coupled receptor 182	-0.845	0.973	0.027
564343851	BPIFB1	BPI fold containing family B member 1	-0.807	0.965	0.035
53791211	PHOX2A	paired like homeobox 2A	-0.807	0.965	0.035
16758272	CPN1	carboxypeptidase N subunit 1	-0.807	0.965	0.035
61556945	MOAP1	modulator of apoptosis 1	-0.795	0.967	0.033
162287322	LSP1	lymphocyte specific protein 1	-0.781	0.982	0.018
74178753	DENND2D	DENN domain containing 2D	-0.778	0.965	0.035
293349343	MYO6	myosin VI	-0.755	0.997	0.003
568927637	ADAMTSL1	ADAMTS like 1	-0.716	0.976	0.024
62821825	OPALIN	oligodendrocytic myelin paranodal and inner loop protein	-0.678	0.965	0.035
293349725	AMER3	APC membrane recruitment protein 3	-0.672	0.981	0.019
672076564	DDC	dopa decarboxylase	-0.632	0.973	0.027
8394221	Rps3a1	ribosomal protein S3A1	-0.630	0.966	0.034
760997729	SYNPO2L	synaptopodin 2 like	-0.621	0.998	0.002
62078563	CD302	CD302 molecule	-0.613	0.983	0.017
281332166	GPR158	G protein-coupled receptor 158	-0.599	0.968	0.032
392339456	CKAP2L	cytoskeleton associated protein 2 like	-0.579	0.992	0.008
291463305	SHISA9	shisa family member 9	-0.574	0.981	0.019
120474989	KRT1	keratin 1	-0.563	0.954	0.046
392331598	MPV17L	MPV17 mitochondrial inner membrane protein like	-0.549	0.987	0.013
188497675	RADX	RPA1 related single stranded DNA binding protein, X-linked	-0.542	0.991	0.009
11067395	Tcam1	testicular cell adhesion molecule 1	-0.516	0.985	0.015
293355224	Rps12-ps24	ribosomal protein S12, pseudogene 24	-0.509	0.991	0.009
77020250	PCSK9	proprotein convertase subtilisin/kexin type 9	-0.485	0.965	0.035
157786612	B9D1	B9 domain containing 1	-0.482	0.953	0.047
225007623	TCFL5	transcription factor like 5	-0.472	0.961	0.039
148709823	PCGF5	polycomb group ring finger 5	-0.460	0.962	0.038
157823151	DLEU7	deleted in lymphocytic leukemia 7	-0.459	0.964	0.036
672084224	CCDC113	coiled-coil domain containing 113	-0.458	0.982	0.018
194440693	Maml2	mastermind like transcriptional coactivator 2	-0.457	0.964	0.036
564346692	GIMAP8	GTPase, IMAP family member 8	-0.455	0.994	0.006
6978629	CD38	CD38 molecule	-0.440	0.998	0.002
201066363	LOXL2	lysyl oxidase like 2	-0.435	0.972	0.028

469663646	NDUFA13	NADH:ubiquinone oxidoreductase subunit A13	-0.433	0.971	0.029
148697062	TMEM255A	transmembrane protein 255A	-0.408	0.951	0.049
58865490	LCMT2	leucine carboxyl methyltransferase 2	-0.407	0.953	0.047
312836782	MRPS27	mitochondrial ribosomal protein S27	-0.395	0.968	0.032
157824150	PTPN22	protein tyrosine phosphatase non-receptor type 22	-0.389	0.994	0.006
157823299	CSGALNACT1	chondroitin sulfate N-acetylgalactosaminyltransferase 1	-0.378	0.981	0.019
16758778	EFNA5	ephrin A5	-0.372	0.974	0.026
8393919	LOC100911216/Pcsk1	proprotein convertase subtilisin/kexin type 1	-0.371	0.968	0.032
672087893	Dmrtc1b	DMRT-like family C1b	-0.371	0.957	0.043
26024221	PRSS12	serine protease 12	-0.366	0.956	0.044
213512607	CLYBL	citramalyl-CoA lyase	-0.365	0.984	0.016
56605758	THAP1	THAP domain containing 1	-0.340	0.980	0.020
672038342	XYLT1	xylosyltransferase 1	-0.329	0.960	0.040
157821925	IFT88	intraflagellar transport 88	-0.320	0.998	0.002
70794782	RBMS2	RNA binding motif single stranded interacting protein 2	-0.316	0.959	0.041
40352944	NXT2	nuclear transport factor 2 like export factor 2	-0.302	0.992	0.008
12621142	RASSF9	Ras association domain family member 9	-0.299	1.000	0.000
149030301	PNOC	prepronociceptin	-0.298	0.984	0.016
157822577	MAN1C1	mannosidase alpha class 1C member 1	-0.292	0.963	0.037
564302385	SHLD1	shieldin complex subunit 1	-0.289	0.955	0.045
157818065	GPR21	G protein-coupled receptor 21	-0.285	0.993	0.007
6754024	GNG4	G protein subunit gamma 4	-0.284	0.981	0.019
392338823	TIPARP	TCDD inducible poly(ADP-ribose) polymerase	-0.281	0.956	0.044
52138628	RAP1B	RAP1B, member of RAS oncogene family	-0.267	0.961	0.039
148703340	SERTM1	serine rich and transmembrane domain containing 1	-0.263	0.968	0.032
148696365	AP5S1	adaptor related protein complex 5 subunit sigma 1	-0.262	0.973	0.027
564341663	SLC43A3	solute carrier family 43 member 3	-0.254	0.988	0.012
564320452	SAP130	Sin3A associated protein 130	-0.253	0.968	0.032
62078973	MIF4GD	MIF4G domain containing	-0.253	0.986	0.014
31560385	RPL21	ribosomal protein L21	-0.252	0.954	0.046
56605728	TMEM218	transmembrane protein 218	-0.251	0.974	0.026
568959109	CEP164	centrosomal protein 164	-0.250	0.986	0.014

6978435	ACADVL	acyl-CoA dehydrogenase very long chain	-0.249	0.952	0.048
298493223	TMEM132B	transmembrane protein 132B	-0.246	0.952	0.048
157822599	GSAP	gamma-secretase activating protein	-0.237	0.958	0.042
449784888	ALDH5A1	aldehyde dehydrogenase 5 family member A1	-0.236	0.979	0.021
62543563	KYAT3	kynurenine aminotransferase 3	-0.233	0.957	0.043
62078935	FLACC1	flagellum associated containing coiled-coil domains 1	-0.233	0.993	0.007
197927216	TBC1D5	TBC1 domain family member 5	-0.230	0.972	0.028
9506425	BET1	Bet1 golgi vesicular membrane trafficking protein	-0.215	1.000	0.000
51948390	HSD17B11	hydroxysteroid 17-beta dehydrogenase 11	-0.213	0.993	0.007
157819089	EOLA1/EOLA2	endothelium and lymphocyte associated ASCH domain 1	-0.213	0.989	0.011
58865418	SUGP1	SURP and G-patch domain containing 1	-0.213	0.998	0.002
564398053	MAN1A1	mannosidase alpha class 1A member 1	-0.213	0.951	0.049
18266704	TRPC5	transient receptor potential cation channel subfamily C member 5	-0.206	0.987	0.013
13540703	PDE1A	phosphodiesterase 1A	-0.205	0.954	0.046
18426866	ACAA2	acetyl-CoA acyltransferase 2	-0.203	0.953	0.047
451770389	HMGCLL1	3-hydroxymethyl-3-methylglutaryl-CoA lyase like 1	-0.202	0.972	0.028
188536087	RAMAC	RNA guanine-7 methyltransferase activating subunit	-0.201	0.951	0.049
309243082	PTPRJ	protein tyrosine phosphatase receptor type J	-0.201	0.957	0.043
148664829	NMRAL1	NmrA like redox sensor 1	-0.200	0.962	0.038
189163499	CYHR1	cysteine and histidine rich 1	-0.198	0.970	0.030
149016262	Col4a4	collagen type IV alpha 4 chain	-0.195	0.994	0.006
564332776	LRRN4CL	LRRN4 C-terminal like	-0.193	0.965	0.035
19705437	EPHA7	EPH receptor A7	-0.186	0.963	0.037
300797496	TDRD6	tudor domain containing 6	-0.184	0.980	0.020
112984202	FZD8	frizzled class receptor 8	-0.183	0.999	0.001
269954719	JAZF1	JAZF zinc finger 1	-0.171	0.988	0.012
50356003	SCP2	sterol carrier protein 2	-0.170	0.967	0.033
149042395	PRDX4	peroxiredoxin 4	-0.169	0.956	0.044
149040047	SYNPR	synaptoporin	-0.167	0.991	0.009
157822019	ITGB1BP1	integrin subunit beta 1 binding protein 1	-0.166	0.981	0.019

79750129	CAMK1D	calcium/calmodulin dependent protein kinase ID	-0.164	0.989	0.011
149057384	C15orf40	chromosome 15 open reading frame 40	-0.163	0.992	0.008
346989661	CPEB2	cytoplasmic polyadenylation element binding protein 2	-0.162	0.968	0.032
157820049	LRFN5	leucine rich repeat and fibronectin type III domain containing 5	-0.161	0.963	0.037
401664552	MRPS7	mitochondrial ribosomal protein S7	-0.159	0.987	0.013
564397835	ASCC1	activating signal cointegrator 1 complex subunit 1	-0.158	0.967	0.033
12838537	C19orf81	chromosome 19 open reading frame 81	-0.156	0.991	0.009
6980978	GPD2	glycerol-3-phosphate dehydrogenase 2	-0.146	0.984	0.016
40254752	PGK1	phosphoglycerate kinase 1	-0.146	0.993	0.007
19173762	NAP1L3	nucleosome assembly protein 1 like 3	-0.144	0.997	0.003
189027115	AIDA	axin interactor, dorsalization associated	-0.140	0.983	0.017
51948478	FARSB	phenylalanyl-tRNA synthetase subunit beta	-0.138	0.975	0.025
6978751	CYP51A1	cytochrome P450 family 51 subfamily A member 1	-0.138	0.954	0.046
672044529	MIGA1	mitoguardin 1	-0.135	0.972	0.028
62078739	TCTA	T cell leukemia translocation altered	-0.131	0.950	0.050
62078667	SLC46A1	solute carrier family 46 member 1	-0.131	0.996	0.004
564351227	E130308A19Rik	RIKEN cDNA E130308A19 gene	-0.129	0.987	0.013
56605990	LRPPRC	leucine rich pentatricopeptide repeat containing	-0.115	0.963	0.037
300794591	FXN	frataxin	-0.110	0.964	0.036
148673176	FABP7	fatty acid binding protein 7	-0.107	0.972	0.028
81884516	Rhno1	RAD9-HUS1-RAD1 interacting nuclear orphan 1	-0.107	0.997	0.003
58865384	NDUFS2	NADH:ubiquinone oxidoreductase core subunit S2	-0.106	0.961	0.039
341823648	RAPH1	Ras association (RalGDS/AF-6) and pleckstrin homology domains 1	-0.103	0.980	0.020
148707802	DARS1	aspartyl-tRNA synthetase 1	-0.103	0.991	0.009
164519053	FAM131B	family with sequence similarity 131 member B	-0.101	0.997	0.003
6981370	PLCG1	phospholipase C gamma 1	-0.088	0.960	0.040
157821365	IFFO1	intermediate filament family orphan 1	-0.084	0.980	0.020
157818729	SPATA33	spermatogenesis associated 33	-0.065	0.994	0.006
300794891	DDX20	DEAD-box helicase 20	-0.060	0.992	0.008

186910247	MRPS21	mitochondrial ribosomal protein S21	-0.059	0.988	0.012
157817861	NDUFA2	NADH:ubiquinone oxidoreductase subunit A2	-0.057	0.992	0.008
300794036	TMEM185B	transmembrane protein 185B	-0.056	0.975	0.025
188536098	SLC48A1	solute carrier family 48 member 1	-0.055	0.959	0.041
71795619	SLC19A2	solute carrier family 19 member 2	-0.052	0.974	0.026
83320121	RBM8A	RNA binding motif protein 8A	-0.052	0.989	0.011
60678266	ENPP5	ectonucleotide pyrophosphatase/phosphodiesterase family member 5	-0.049	0.951	0.049
18426824	KHDRBS1	KH RNA binding domain containing, signal transduction associated 1	-0.047	0.964	0.036
402743461	DPY19L3	dpy-19 like C-mannosyltransferase 3	-0.046	0.973	0.027
157823867	TLL1	tolloid like 1	-0.044	0.989	0.011
24638208	EXOC2	exocyst complex component 2	-0.041	0.981	0.019
392333100	FAM193A	family with sequence similarity 193 member A	0.027	-0.958	0.042
58866026	XK	X-linked Kx blood group	0.033	-0.986	0.014
672046314	AMBRA1	autophagy and beclin 1 regulator 1	0.034	-0.965	0.035
148686551	PPWD1	peptidylprolyl isomerase domain and WD repeat containing 1	0.038	-0.956	0.044
157821407	FBXO28	F-box protein 28	0.039	-0.999	0.001
9506875	SMAD4	SMAD family member 4	0.047	-0.987	0.013
149049696	MKRN2	makorin ring finger protein 2	0.052	-0.994	0.006
149052177	MRPL28	mitochondrial ribosomal protein L28	0.052	-0.987	0.013
157817871	MEGF9	multiple EGF like domains 9	0.055	-0.985	0.015
157819325	SRP68	signal recognition particle 68	0.056	-0.986	0.014
18644718	RGS3	regulator of G protein signaling 3	0.057	-0.988	0.012
157786974	Wdr83os	WD repeat domain 83 opposite strand	0.067	-0.965	0.035
37360568	RANGAP1	Ran GTPase activating protein 1	0.068	-0.968	0.032
89363040	PCDHGA11	protocadherin gamma subfamily A, 11	0.075	-0.981	0.019
148672705	TMEM184B	transmembrane protein 184B	0.080	-0.997	0.003
13162349	ASIC1	acid sensing ion channel subunit 1	0.082	-0.971	0.029
157818421	TVP23A	trans-golgi network vesicle protein 23 homolog A	0.085	-0.988	0.012
564387037	COG3	component of oligomeric golgi complex 3	0.091	-0.956	0.044
148673403	GRSF1	G-rich RNA sequence binding factor 1	0.108	-0.956	0.044
564388185	ERCC6	ERCC excision repair 6, chromatin remodeling factor	0.112	-0.999	0.001
274325505	Pwp2	PWP2 periodic tryptophan protein homolog (yeast)	0.115	-0.982	0.018

189011602	NLE1	notchless homolog 1	0.118	-0.990	0.010
81158095	PCDHGA3	protocadherin gamma subfamily A, 3	0.118	-0.972	0.028
157818061	2510002D24Rik	RIKEN cDNA 2510002D24 gene	0.120	-0.989	0.011
61556860	MRPL46	mitochondrial ribosomal protein L46	0.120	-0.966	0.034
11968114	MRPL23	mitochondrial ribosomal protein L23	0.127	-0.994	0.006
16758194	RGS2	regulator of G protein signaling 2	0.127	-0.953	0.047
564361244	TCF20	transcription factor 20	0.128	-0.993	0.007
62656582	KIAA0100	KIAA0100	0.130	-0.992	0.008
157821561	ATRIP	ATR interacting protein	0.132	-0.968	0.032
11177894	TSC1	TSC complex subunit 1	0.134	-0.985	0.015
564398269	SCML4	Scm polycomb group protein like 4	0.135	-0.951	0.049
157816981	DNAJC30	DnaJ heat shock protein family (Hsp40) member C30	0.137	-0.998	0.002
148491097	DYNC1H1	dynein cytoplasmic 1 heavy chain 1	0.137	-0.964	0.036
157820897	MTHFD2	methylenetetrahydrofolate dehydrogenase (NADP+ dependent) 2, methenyltetrahydrofolate cyclohydrolase	0.141	-0.969	0.031
19924073	TTL	tubulin tyrosine ligase	0.142	-0.963	0.037
157819431	BRD3	bromodomain containing 3	0.142	-0.979	0.021
564370907	ZNF598	zinc finger protein 598, E3 ubiquitin ligase	0.144	-0.955	0.045
291042494	MED13L	mediator complex subunit 13L	0.145	-0.967	0.033
392333209	DLG5	discs large MAGUK scaffold protein 5	0.146	-0.968	0.032
300798201	PYGO1	pygopus family PHD finger 1	0.147	-0.993	0.007
41386755	FGFR1OP2	FGFR1 oncogene partner 2	0.148	-0.984	0.016
281427188	ZC3H13	zinc finger CCCH-type containing 13	0.149	-0.977	0.023
564321656	TCF25	transcription factor 25	0.149	-0.969	0.031
157820865	DDX28	DEAD-box helicase 28	0.153	-0.966	0.034
50511177	SLITRK1	SLIT and NTRK like family member 1	0.155	-0.962	0.038
157820917	CDC7	cell division cycle 7	0.156	-0.958	0.042
157822569	Tubgcp3	tubulin, gamma complex associated protein 3	0.156	-0.972	0.028
74200325	UBE2G2	ubiquitin conjugating enzyme E2 G2	0.158	-0.992	0.008
41053837	GPX3	glutathione peroxidase 3	0.159	-0.962	0.038
157818167	PDPR	pyruvate dehydrogenase phosphatase regulatory subunit	0.160	-0.963	0.037
300796412	ATMIN	ATM interactor	0.160	-0.997	0.003
11559951	NRBF2	nuclear receptor binding factor 2	0.163	-0.987	0.013
112984440	TNFRSF19	TNF receptor superfamily member 19	0.164	-0.972	0.028
300797915	Rbm33	RNA binding motif protein 33	0.166	-0.998	0.002
8393104	CHKB	choline kinase beta	0.166	-0.971	0.029

37360236	SMG5	SMG5 nonsense mediated mRNA decay factor	0.169	-0.975	0.025
6981572	SP4	Sp4 transcription factor	0.169	-0.983	0.017
57528294	NEPRO	nucleolus and neural progenitor protein	0.169	-0.976	0.024
40807349	DSTYK	dual serine/threonine and tyrosine protein kinase	0.172	-0.973	0.027
149044495	CAAP1	caspase activity and apoptosis inhibitor 1	0.172	-1.000	0.000
300794761	Fat4	FAT atypical cadherin 4	0.173	-0.980	0.020
157823565	COQ10A	coenzyme Q10A	0.174	-0.965	0.035
392355126	HAUS2	HAUS augmin like complex subunit 2	0.175	-0.995	0.005
564303143	KMT2C	lysine methyltransferase 2C	0.176	-0.991	0.009
37360004	KDM1A	lysine demethylase 1A	0.177	-0.969	0.031
164565360	CTTNBP2NL	CTTNBP2 N-terminal like	0.180	-0.983	0.017
38454226	TPD52L2	TPD52 like 2	0.180	-0.970	0.030
300797562	BCOR	BCL6 corepressor	0.183	-0.980	0.020
8393959	PIM1	Pim-1 proto-oncogene, serine/threonine kinase	0.188	-0.977	0.023
157823683	HDDC2	HD domain containing 2	0.189	-0.983	0.017
29789319	CBLB	Cbl proto-oncogene B	0.192	-0.990	0.010
392332910	TP53BP2	tumor protein p53 binding protein 2	0.194	-0.953	0.047
157818041	YEATS2	YEATS domain containing 2	0.195	-0.996	0.004
148674304	RPRD1B	regulation of nuclear pre-mRNA domain containing 1B	0.198	-0.963	0.037
157821415	GZF1	GDNF inducible zinc finger protein 1	0.201	-0.990	0.010
672031398	ANKRD11	ankyrin repeat domain 11	0.203	-0.978	0.022
300794743	TSC22D2	TSC22 domain family member 2	0.206	-0.979	0.021
149030718	PIP5K1A	phosphatidylinositol-4-phosphate 5-kinase type 1 alpha	0.211	-0.979	0.021
109470195	TNKS1BP1	tankyrase 1 binding protein 1	0.213	-0.992	0.008
564320608	SEMA6A	semaphorin 6A	0.214	-0.978	0.022
124249254	ZNF639	zinc finger protein 639	0.217	-0.977	0.023
564370219	LPIN2	lipin 2	0.218	-0.991	0.009
157820985	NCK1	NCK adaptor protein 1	0.221	-0.957	0.043
568992461	DIP2B	disco interacting protein 2 homolog B	0.223	-0.963	0.037
270483881	CBFA2T2	CBFA2/RUNX1 partner transcriptional co-repressor 2	0.231	-0.952	0.048
672039306	RCOR2	REST corepressor 2	0.232	-0.976	0.024
11560052	DUSP12	dual specificity phosphatase 12	0.234	-0.972	0.028
149062169	MEN1	menin 1	0.236	-0.975	0.025
67078478	NAF1	nuclear assembly factor 1 ribonucleoprotein	0.237	-0.969	0.031
149023044	TMEM87A	transmembrane protein 87A	0.241	-0.964	0.036

256818763	PLEKHH1	pleckstrin homology, MyTH4 and FERM domain containing H1	0.252	-0.956	0.044
672036088	KMT2B	lysine methyltransferase 2B	0.253	-0.970	0.030
157819581	SESN2	sestrin 2	0.267	-0.979	0.021
31543579	RELN	reelin	0.268	-0.987	0.013
244792650	TNIK	TRAF2 and NCK interacting kinase	0.268	-0.990	0.010
56090325	PACC1	proton activated chloride channel 1	0.269	-0.990	0.010
205830446	C11orf98	chromosome 11 open reading frame 98	0.275	-0.966	0.034
157819811	C21orf91	chromosome 21 open reading frame 91	0.275	-0.966	0.034
109464919	ARHGEF26	Rho guanine nucleotide exchange factor 26	0.278	-0.970	0.030
347921954	Lilrb2	leukocyte immunoglobulin like receptor B2	0.290	-0.965	0.035
586597897	DBP	D-box binding PAR bZIP transcription factor	0.292	-0.974	0.026
564363988	ISLR2	immunoglobulin superfamily containing leucine rich repeat 2	0.293	-0.955	0.045
148683687	RHBDL3	rhomboid like 3	0.301	-0.971	0.029
13928740	RGN	regucalcin	0.303	-0.965	0.035
56605628	SFT2D1	SFT2 domain containing 1	0.306	-0.969	0.031
62078701	UTP25	UTP25 small subunit processor component	0.308	-0.953	0.047
6680532	KCNJ3	potassium inwardly rectifying channel subfamily J member 3	0.308	-0.966	0.034
300797262	BRPF1	bromodomain and PHD finger containing 1	0.308	-0.973	0.027
6978497	AMBP	alpha-1-microglobulin/bikunin precursor	0.308	-0.965	0.035
72255513	AGA	aspartylglucosaminidase	0.309	-0.992	0.008
157819887	LACTB	lactamase beta	0.314	-0.968	0.032
149048116	KHDC4	KH domain containing 4, pre-mRNA splicing factor	0.314	-0.993	0.007
157816943	MCM8	minichromosome maintenance 8 homologous recombination repair factor	0.316	-0.981	0.019
17865345	CDH23	cadherin related 23	0.322	-0.976	0.024
404501489	DDX55	DEAD-box helicase 55	0.329	-0.976	0.024
349732232	NFATC1	nuclear factor of activated T cells 1	0.335	-0.984	0.016
672055128	SAMD11	sterile alpha motif domain containing 11	0.342	-0.957	0.043
77917610	GPBP1L1	GC-rich promoter binding protein 1 like 1	0.344	-0.980	0.020

164518930	SDK1	sidekick cell adhesion molecule 1	0.347	-0.996	0.004
76096340	ANKRD16	ankyrin repeat domain 16	0.354	-0.999	0.001
564393142	WDR36	WD repeat domain 36	0.359	-0.973	0.027
157817592	HEXIM2	HEXIM P-TEFb complex subunit 2	0.366	-0.971	0.029
16758666	TIMP1	TIMP metallopeptidase inhibitor 1	0.376	-0.985	0.015
564326636	Zfp94	zinc finger protein 94	0.378	-0.977	0.023
171846573	FBXL4	F-box and leucine rich repeat protein 4	0.393	-0.990	0.010
61889068	MXI1	MAX interactor 1, dimerization protein	0.405	-0.957	0.043
56090421	PXYLP1	2-phosphoxylose phosphatase 1	0.429	-0.980	0.020
293341533	LOC108348225	feline leukemia virus subgroup C receptor-related protein 1	0.451	-0.991	0.009
845633640	TSSC4	tumor suppressing subtransferable candidate 4	0.458	-0.982	0.018
67078462	SOX18	SRY-box transcription factor 18	0.460	-0.990	0.010
126722629	HSPBAP1	HSPB1 associated protein 1	0.460	-0.969	0.031
157819737	SARS2	seryl-tRNA synthetase 2, mitochondrial	0.463	-0.995	0.005
157822327	ATG14	autophagy related 14	0.464	-0.955	0.045
157817797	PDCD2L	programmed cell death 2 like	0.468	-0.967	0.033
213385320	LRTOMT	leucine rich transmembrane and O-methyltransferase domain containing	0.476	-0.976	0.024
281306771	ADAMTS4	ADAM metallopeptidase with thrombospondin type 1 motif 4	0.489	-0.991	0.009
564320454	SAP130	Sin3A associated protein 130	0.559	-0.990	0.010
62640766	GDPGP1	GDP-D-glucose phosphorylase 1	0.562	-0.954	0.046
52851389	OSMR	oncostatin M receptor	0.570	-0.960	0.040
62078983	DNAJC28	DnaJ heat shock protein family (Hsp40) member C28	0.570	-0.978	0.022
16924020	XPNPEP2	X-prolyl aminopeptidase 2	0.585	-0.965	0.035
564303143	KMT2C	lysine methyltransferase 2C	0.593	-0.999	0.001
157786962	NANOS3	nanos C2HC-type zinc finger 3	0.596	-0.968	0.032
148683584	VEPH1	ventricular zone expressed PH domain containing 1	0.603	-0.986	0.014
197386987	HDX	highly divergent homeobox	0.620	-0.988	0.012
197387536	TEX26	testis expressed 26	0.706	-0.994	0.006
672014266	TMEM219	transmembrane protein 219	0.735	-0.997	0.003
6978663	CLCN1	chloride voltage-gated channel 1	0.742	-0.997	0.003
149042882	ZNF334	zinc finger protein 334	0.779	-1.000	0.000
755566692	HUWE1	HECT, UBA and WWE domain containing E3 ubiquitin protein ligase 1	0.787	-0.990	0.010
56606104	Aox4	aldehyde oxidase 4	0.807	-0.965	0.035

293348634	LRIG3	leucine rich repeats and immunoglobulin like domains 3	0.825	-0.953	0.047
293347270	OSGIN2	oxidative stress induced growth inhibitor family member 2	0.861	-0.998	0.002
672088752	MCF2	MCF.2 cell line derived transforming sequence	0.909	-0.996	0.004
157820193	Tbx2	T-box transcription factor 2	0.909	-0.991	0.009
564378170	PAN3	poly(A) specific ribonuclease subunit PAN3	0.932	-0.963	0.037
1438906	NPY5R	neuropeptide Y receptor Y5	1.000	-0.953	0.047
53734355	P2RY14	purinergic receptor P2Y14	1.000	-0.976	0.024
61097928	SNAI1	snail family transcriptional repressor 1	1.053	-0.968	0.032
564306247	PHACTR4	phosphatase and actin regulator 4	1.189	-0.986	0.014
40786461	NAPEPLD	N-acyl phosphatidylethanolamine phospholipase D	1.199	-0.997	0.003
672056787	PRIMA1	proline rich membrane anchor 1	1.234	-1.000	0.000
7106248	ANKRD1	ankyrin repeat domain 1	1.252	-0.998	0.002
157822485	H2BC15	H2B clustered histone 15	1.265	-0.965	0.035
672084625	LOC100909409 (includes others)	RGD1562660	1.407	-0.992	0.008
11024678	Dbil5	diazepam binding inhibitor-like 5	1.423	-0.974	0.026
157824012	TRIM45	tripartite motif containing 45	1.444	-0.996	0.004
56606094	Aox2	aldehyde oxidase 2	1.459	-0.987	0.013
11560026	STC2	stanniocalcin 2	1.585	-0.969	0.031
58866012	TRIM55	tripartite motif containing 55	1.644	-0.965	0.035
254553399	FBXO24	F-box protein 24	1.716	-0.984	0.016
157786614	MFSD6L	major facilitator superfamily domain containing 6 like	1.716	-0.984	0.016
148230802	Akr1c12	aldo-keto reductase family 1, member C12	1.826	-0.982	0.018
207446700	Sec1	secretory blood group 1	1.826	-0.982	0.018
157823427	KLHL31	kelch like family member 31	2.000	-0.991	0.009
148685413	ATP2A1	ATPase sarcoplasmic/endoplasmic reticulum Ca2+ transporting 1	2.000	-0.950	0.050
300794644	FREM3	FRAS1 related extracellular matrix 3	2.087	-0.953	0.047
564312627	ZFP62	ZFP62 zinc finger protein	2.139	-0.972	0.028
11024668	AIPL1	aryl hydrocarbon receptor interacting protein like 1	2.188	-0.991	0.009
158508517	SDS	serine dehydratase	2.202	-0.991	0.009
392340509	PTPRD	protein tyrosine phosphatase receptor type D	2.231	-0.986	0.014
56270329	ATP1A4	ATPase Na+/K+ transporting subunit alpha 4	2.322	-0.986	0.014
392342139	TTC21A	tetratricopeptide repeat domain 21A	2.389	-0.992	0.008

672071273	GRAMD1C	GRAM domain containing 1C	2.450	-0.991	0.009
157820271	LOXL4	lysyl oxidase like 4	2.585	-1.000	0.000
13994175	FUT2	fucosyltransferase 2	2.585	-0.970	0.030
9507065	SCN11A	sodium voltage-gated channel alpha subunit 11	2.755	-0.983	0.017
564329920	EMSY	EMSY transcriptional repressor, BRCA2 interacting	3.112	-0.969	0.031
8393891	P2RY2	purinergic receptor P2Y2	3.170	-0.964	0.036
112984288	STEAP4	STEAP4 metalloreductase	3.248	-0.991	0.009
672078236	Gucy1b2	guanylate cyclase 1, soluble, beta 2	3.322	-0.990	0.010
50370130	PALLD	palladin, cytoskeletal associated protein	3.361	-0.965	0.035
293340128	MIEF2	mitochondrial elongation factor 2	3.426	-0.978	0.022
402747041	FAM217A	family with sequence similarity 217 member A	3.954	-0.991	0.009
166157542	PABPN1L	PABPN1 like, cytoplasmic	3.954	-0.965	0.035
392351087	HAGHL	hydroxyacylglutathione hydrolase like	4.120	-0.981	0.019
47577861	OR7D2	olfactory receptor family 7 subfamily D member 2	4.248	-0.952	0.048
165970757	H2-T24	histocompatibility 2, T region locus 24	4.285	-0.987	0.013
11024666	NTRK1	neurotrophic receptor tyrosine kinase 1	4.285	-0.957	0.043
298231202	PRG2	proteoglycan 2, pro eosinophil major basic protein	4.426	-0.961	0.039
13928958	CRYBB3	crystallin beta B3	4.672	-0.956	0.044
392333013	CEP135	centrosomal protein 135	4.858	-0.985	0.015
672020628	ATXN7L1	ataxin 7 like 1	5.157	-0.957	0.043
300797609	ELOVL7	ELOVL fatty acid elongase 7	7.451	-0.991	0.009

Supplementary Table S6. The list of genes that are differentially expressed in the offspring hippocampus in response to prenatal BPA exposure that exhibited the changes in the expression levels correlated with the neuronal density in CA2/3 of the hippocampus. The transcriptome profiling data of DEGs in male and female rat offspring prenatally exposed to BPA (n = 6, male pups n = 3 and female pups n = 3, from independent litters) or the vehicle control (n = 6, male pups n = 3 and female pups n = 3, from independent litters) were obtained and used for the PTM analyses to identify DEGs that exhibited the changes in the expression levels correlated with the neuronal density in CA2/3 of the hippocampus.

ID	Symbol	Entrez Gene Name	log2(FC)	R values	P-values
564375502	Mxra7	matrix-remodelling associated 7	-7.209	0.951	0.049
567315993	LOC102550396	LRRGT00188	-5.600	0.951	0.049
564312230	LOC100912948	multidrug resistance-associated protein 1-like	-5.285	0.951	0.049
672035060	CIC	capicua transcriptional repressor	-4.863	0.960	0.040
209447125	Ctf2	cardiotrophin 2	-4.392	0.951	0.049
201860265	NRN1L	neuritin 1 like	-4.358	0.950	0.050
51591901	MPIG6B	megakaryocyte and platelet inhibitory receptor G6b	-4.170	0.951	0.049
6978515	APOA1	apolipoprotein A1	-3.807	0.951	0.049
117647210	CTRC	chymotrypsin C	-3.807	0.951	0.049
48040447	SUCNR1	succinate receptor 1	-3.700	0.951	0.049
194473646	UPK3A	uroplakin 3A	-3.585	0.951	0.049
164518908	RAB25	RAB25, member RAS oncogene family	-3.459	0.951	0.049
285026465	HS3ST3A1	heparan sulfate-glucosamine 3-sulfotransferase 3A1	-3.322	0.951	0.049
148710035	PITX3	paired like homeodomain 3	-3.322	0.951	0.049
56090299	ODF4	outer dense fiber of sperm tails 4	-3.170	0.984	0.016
157822159	CCDC42	coiled-coil domain containing 42	-3.170	0.951	0.049
123173794	GSG1	germ cell associated 1	-3.170	0.951	0.049
77917534	CBLC	Cbl proto-oncogene C	-2.807	0.951	0.049
157822121	LRMDA	leucine rich melanocyte differentiation associated	-2.747	0.966	0.034
672032217	REPS2	RALBP1 associated Eps domain containing 2	-2.683	0.974	0.026
298566276	Ces1a	carboxylesterase 1A	-2.585	0.951	0.049
57222328	PFN4	profilin family member 4	-2.585	0.951	0.049
157786772	KREMEN2	kringle containing transmembrane protein 2	-2.585	0.951	0.049
306482632	BPIFB4	BPI fold containing family B member 4	-2.585	0.951	0.049

57222300	Klra2	killer cell lectin-like receptor, subfamily A, member 2	-2.585	0.951	0.049
157822463	Nkx6-3	NK6 homeobox 3	-2.585	0.951	0.049
62945342	LAX1	lymphocyte transmembrane adaptor 1	-2.322	0.951	0.049
187469451	CLEC7A	C-type lectin domain containing 7A	-2.322	0.951	0.049
157822087	ACTRT3	actin related protein T3	-2.170	0.953	0.047
55741882	ZPBP2	zona pellucida binding protein 2	-2.022	0.950	0.050
157822587	PDE6B	phosphodiesterase 6B	-2.000	0.951	0.049
157818091	TMEM182	transmembrane protein 182	-2.000	0.951	0.049
296483047	SIX1	SIX homeobox 1	-2.000	0.951	0.049
117647206	DDX4	DEAD-box helicase 4	-2.000	0.951	0.049
19424304	CHRNB3	cholinergic receptor nicotinic beta 3 subunit	-2.000	0.951	0.049
47577151	Olfr1441	olfactory receptor 1441	-1.972	0.951	0.049
56676350	PRSS35	serine protease 35	-1.962	0.980	0.020
149066014	PVALB	parvalbumin	-1.939	0.994	0.006
157820217	Gsta4	glutathione S-transferase, alpha 4	-1.930	0.960	0.040
81295367	Abcg3	ATP binding cassette subfamily G member 3	-1.898	0.982	0.018
13592031	PTGER2	prostaglandin E receptor 2	-1.845	0.975	0.025
226698394	UNC80	unc-80 homolog, NALCN channel complex subunit	-1.781	0.980	0.020
157819477	GLOD5	glyoxalase domain containing 5	-1.768	0.952	0.048
300796107	PROX2	prospero homeobox 2	-1.700	0.992	0.008
9910234	IFIT1B	interferon induced protein with tetratricopeptide repeats 1B	-1.678	0.951	0.049
392331668	HAGHL	hydroxyacylglutathione hydrolase like	-1.665	0.956	0.044
58331159	GSTA3	glutathione S-transferase alpha 3	-1.603	0.968	0.032
157821527	RHOD	ras homolog family member D	-1.597	0.973	0.027
158711755	C17orf97	chromosome 17 open reading frame 97	-1.592	0.951	0.049
157819393	NNMT	nicotinamide N-methyltransferase	-1.585	0.964	0.036
157818603	CLCA2	chloride channel accessory 2	-1.585	0.951	0.049
685156911	NLRP4	NLR family pyrin domain containing 4	-1.585	0.951	0.049
155369702	ECHDC3	enoyl-CoA hydratase domain containing 3	-1.497	0.960	0.040
71043750	SYNGR4	synaptogyrin 4	-1.464	0.977	0.023
31377521	S1PR5	sphingosine-1-phosphate receptor 5	-1.426	0.999	0.001
77917586	GRAP2	GRB2 related adaptor protein 2	-1.412	0.999	0.001
157822105	SLC49A3	solute carrier family 49 member 3	-1.389	0.974	0.026
340523096	IL10RA	interleukin 10 receptor subunit alpha	-1.347	0.978	0.022
672025117	MBTD1	mbt domain containing 1	-1.344	0.956	0.044
6978717	CTRB2	chymotrypsinogen B2	-1.342	0.982	0.018

307746876	Pzp	PZP, alpha-2-macroglobulin like	-1.322	0.951	0.049
62656582	KIAA0100	KIAA0100	-1.266	0.962	0.038
672034215	ZNF729	zinc finger protein 729	-1.256	0.972	0.028
16758338	FTCD	formimidoyltransferase cyclodeaminase	-1.208	0.950	0.050
149408137	DHX58	DExH-box helicase 58	-1.183	0.982	0.018
281332082	THBS2	thrombospondin 2	-1.164	0.995	0.005
406035319	KIRREL2	kirre like nephrin family adhesion molecule 2	-1.154	0.969	0.031
60223053	SEPTIN1	septin 1	-1.133	0.967	0.033
392331829	ATAD5	ATPase family AAA domain containing 5	-1.122	0.968	0.032
157819247	CPA4	carboxypeptidase A4	-1.087	0.951	0.049
564310671	NBEAL2	neurobeachin like 2	-1.084	0.977	0.023
157818961	UBA7	ubiquitin like modifier activating enzyme 7	-1.078	0.966	0.034
157821423	TBX6	T-box transcription factor 6	-1.017	0.995	0.005
68163493	DAW1	dynein assembly factor with WD repeats 1	-1.005	0.960	0.040
156231008	PRND	prion like protein doppel	-1.000	0.951	0.049
25453414	ASS1	argininosuccinate synthase 1	-1.000	0.990	0.010
392342053	GK5	glycerol kinase 5	-1.000	0.992	0.008
59676595	FAM20A	FAM20A golgi associated secretory pathway pseudokinase	-1.000	0.965	0.035
765826426	Acot6	acyl-CoA thioesterase 6	-1.000	0.976	0.024
215276950	PKP2	plakophilin 2	-0.973	0.968	0.032
672033256	LOC100912904	disks large homolog 5-like	-0.960	0.966	0.034
157820951	PRSS53	serine protease 53	-0.959	0.951	0.049
16758344	SEC16B	SEC16 homolog B, endoplasmic reticulum export factor	-0.958	0.989	0.011
157818989	LRRC71	leucine rich repeat containing 71	-0.952	0.972	0.028
198442873	CDC14A	cell division cycle 14A	-0.936	0.956	0.044
62945330	SLC8B1	solute carrier family 8 member B1	-0.929	0.952	0.048
50657416	C1RL	complement C1r subcomponent like	-0.918	0.951	0.049
47059114	LTB	lymphotoxin beta	-0.916	0.978	0.022
293347270	OSGIN2	oxidative stress induced growth inhibitor family member 2	-0.915	0.973	0.027
164663906	PDIA2	protein disulfide isomerase family A member 2	-0.909	0.978	0.022
564325648	Zfp54	zinc finger protein 54	-0.907	0.991	0.009
672016875	LOC103690320	FERM and PDZ domain-containing protein 3	-0.897	0.980	0.020
12621098	EPHX2	epoxide hydrolase 2	-0.887	0.991	0.009
148670853	BBOF1	basal body orientation factor 1	-0.878	0.985	0.015

209870105	GPR37L1	G protein-coupled receptor 37 like 1	-0.870	0.977	0.023
57114338	SCN4B	sodium voltage-gated channel beta subunit 4	-0.859	0.981	0.019
62945324	LCA5L	lebercilin LCA5 like	-0.853	0.953	0.047
6978791	EDN1	endothelin 1	-0.850	0.957	0.043
564373460	SLFN13	schlafen family member 13	-0.831	0.994	0.006
56605720	GADD45B	growth arrest and DNA damage inducible beta	-0.828	0.952	0.048
6980992	GSTT2/GSTT2B	glutathione S-transferase theta 2 (gene/pseudogene)	-0.824	0.957	0.043
58865784	GPR157	G protein-coupled receptor 157	-0.812	0.993	0.007
259089426	AGER	advanced glycosylation end-product specific receptor	-0.804	0.971	0.029
18426812	ADA	adenosine deaminase	-0.787	0.997	0.003
307548437	NYAP2	neuronal tyrosine-phosphorylated phosphoinositide-3-kinase adaptor 2	-0.780	0.984	0.016
157819203	TECTA	tectorin alpha	-0.778	0.977	0.023
942523340	CAPRIN2	caprin family member 2	-0.774	0.959	0.041
157819205	EFHC2	EF-hand domain containing 2	-0.772	0.972	0.028
61097937	VEGFB	vascular endothelial growth factor B	-0.766	0.985	0.015
56090397	CYB5D2	cytochrome b5 domain containing 2	-0.764	0.995	0.005
27465577	Cyp4f16/Cyp4f37	cytochrome P450, family 4, subfamily f, polypeptide 16	-0.758	0.989	0.011
672069802	C1QTNF1	C1q and TNF related 1	-0.749	0.982	0.018
68341959	CASTOR1	cytosolic arginine sensor for mTORC1 subunit 1	-0.712	0.957	0.043
300797913	PALB2	partner and localizer of BRCA2	-0.679	0.953	0.047
293349725	AMER3	APC membrane recruitment protein 3	-0.672	0.951	0.049
70912395	CFAP20DC	CFAP20 domain containing	-0.659	0.964	0.036
157824216	RRAS	RAS related	-0.655	0.950	0.050
157823283	Coch	cochlin	-0.653	0.992	0.008
157819783	IRF6	interferon regulatory factor 6	-0.648	0.953	0.047
68534736	ERAP1	endoplasmic reticulum aminopeptidase 1	-0.646	0.958	0.042
564348231	RPUSD3	RNA pseudouridine synthase D3	-0.645	0.975	0.025
564316927	FRYL	FRY like transcription coactivator	-0.643	0.953	0.047
293340128	MIEF2	mitochondrial elongation factor 2	-0.642	0.985	0.015
31542804	FCGR2A	Fc fragment of IgG receptor IIa	-0.633	0.982	0.018
157824208	NTNG1	netrin G1	-0.628	0.993	0.007
564395215	LOC100909409 (includes others)	RGD1562660	-0.626	0.999	0.001
157819949	ITGA4	integrin subunit alpha 4	-0.620	0.967	0.033
157786914	OGFOD2	2-oxoglutarate and iron dependent oxygenase domain containing 2	-0.618	0.955	0.045

402478640	HTRA3	HtrA serine peptidase 3	-0.608	0.954	0.046
157818491	DUS2	dihydrouridine synthase 2	-0.602	0.966	0.034
27465529	SLC9A4	solute carrier family 9 member A4	-0.601	0.974	0.026
56605940	RXFP3	relaxin family peptide receptor 3	-0.599	0.988	0.012
281332166	GPR158	G protein-coupled receptor 158	-0.599	0.983	0.017
82654224	IDNK	IDNK gluconokinase	-0.597	0.994	0.006
567316103	Ac1576	uncharacterized LOC102552783	-0.587	0.956	0.044
300793858	PARP14	poly(ADP-ribose) polymerase family member 14	-0.585	0.989	0.011
564394999	CLGN	calmegin	-0.582	0.954	0.046
187282394	USP43	ubiquitin specific peptidase 43	-0.577	0.955	0.045
564300485	LOC102551095	uncharacterized LOC102551095	-0.576	0.984	0.016
564366772	MGC116197 (includes others)	similar to RIKEN cDNA 1700001E04	-0.569	0.999	0.001
157816941	PLXDC1	plexin domain containing 1	-0.567	0.990	0.010
62078799	QRSL1	glutaminyl-tRNA amidotransferase subunit QRSL1	-0.566	0.957	0.043
149048141	PMF1/PMF1-BGLAP	polyamine modulated factor 1	-0.565	0.954	0.046
75832150	GALNT3	polypeptide N-acetylgalactosaminyltransferase 3	-0.557	0.973	0.027
213385268	Gm10778	predicted gene 10778	-0.554	0.984	0.016
148747464	SCD	stearoyl-CoA desaturase	-0.524	0.991	0.009
6981180	MAOB	monoamine oxidase B	-0.518	0.979	0.021
149037033	PRDM5	PR/SET domain 5	-0.516	0.994	0.006
62078773	CCDC81	coiled-coil domain containing 81	-0.511	0.962	0.038
9506709	GALR2	galanin receptor 2	-0.500	0.979	0.021
12738847	MERTK	MER proto-oncogene, tyrosine kinase	-0.488	0.997	0.003
558611343	MCM3	minichromosome maintenance complex component 3	-0.488	0.959	0.041
157817033	TJAP1	tight junction associated protein 1	-0.487	0.959	0.041
672084625	LOC100909409 (includes others)	RGD1562660	-0.487	0.991	0.009
395759219	AQP4	aquaporin 4	-0.482	0.984	0.016
157786612	B9D1	B9 domain containing 1	-0.482	0.992	0.008
564306247	PHACTR4	phosphatase and actin regulator 4	-0.477	0.967	0.033
189011606	NCEH1	neutral cholesterol ester hydrolase 1	-0.476	0.968	0.032
225007623	TCFL5	transcription factor like 5	-0.472	0.972	0.028
148356229	CCND1	cyclin D1	-0.464	0.964	0.036
157823151	DLEU7	deleted in lymphocytic leukemia 7	-0.459	0.950	0.050
219275548	DUSP19	dual specificity phosphatase 19	-0.458	0.984	0.016
293346302	FBLN7	fibulin 7	-0.448	0.961	0.039
392331978	CDR2L	cerebellar degeneration related protein 2 like	-0.442	0.968	0.032

68534547	NUDT18	nudix hydrolase 18	-0.437	0.977	0.023
201066363	LOXL2	lysyl oxidase like 2	-0.435	0.965	0.035
61889119	TNFSF12	TNF superfamily member 12	-0.432	0.984	0.016
210031518	MOGAT2	monoacylglycerol O-acyltransferase 2	-0.432	0.979	0.021
11560087	PYGL	glycogen phosphorylase L	-0.431	0.979	0.021
110347493	PCDHA9	protocadherin alpha 9	-0.429	0.983	0.017
157823259	TMEM229A	transmembrane protein 229A	-0.424	0.984	0.016
62078447	HBA1/HBA2	hemoglobin subunit alpha 2	-0.420	0.964	0.036
194473652	TTC38	tetratricopeptide repeat domain 38	-0.413	0.983	0.017
157819765	OGDHL	oxoglutarate dehydrogenase like	-0.413	0.967	0.033
9506405	ARPC1B	actin related protein 2/3 complex subunit 1B	-0.410	1.000	0.000
148697062	TMEM255A	transmembrane protein 255A	-0.408	0.993	0.007
58865490	LCMT2	leucine carboxyl methyltransferase 2	-0.407	0.986	0.014
13162347	FDXR	ferredoxin reductase	-0.405	0.966	0.034
148666792	ARHGAP25	Rho GTPase activating protein 25	-0.396	0.958	0.042
312836782	MRPS27	mitochondrial ribosomal protein S27	-0.395	0.974	0.026
157820327	THSD1	thrombospondin type 1 domain containing 1	-0.391	0.972	0.028
149034469	GNG7	G protein subunit gamma 7	-0.381	0.959	0.041
149036529	DGUOK	deoxyguanosine kinase	-0.381	0.976	0.024
157823299	CSGALNACT1	chondroitin sulfate N-acetylgalactosaminyltransferase 1	-0.378	0.952	0.048
149042939	WFDC2	WAP four-disulfide core domain 2	-0.373	0.952	0.048
929981595	NPHP1	nephrocystin 1	-0.372	0.987	0.013
564299653	FAM169A	family with sequence similarity 169 member A	-0.372	0.969	0.031
672087893	Dmrtc1b	DMRT-like family C1b	-0.371	0.985	0.015
198386343	TRPS1	transcriptional repressor GATA binding 1	-0.370	0.981	0.019
564343748	CDK5RAP1	CDK5 regulatory subunit associated protein 1	-0.368	0.987	0.013
564357619	ITGB8	integrin subunit beta 8	-0.366	0.986	0.014
55741549	MRPL13	mitochondrial ribosomal protein L13	-0.360	0.953	0.047
40786491	CYP20A1	cytochrome P450 family 20 subfamily A member 1	-0.358	0.967	0.033
149066868	MDM1	Mdm1 nuclear protein	-0.357	0.970	0.030
157822187	WWOX	WW domain containing oxidoreductase	-0.355	0.997	0.003
11693172	CALR	calreticulin	-0.354	0.987	0.013
56605656	DONSON	DNA replication fork stabilization factor DONSON	-0.350	0.966	0.034
451172073	CHRM3	cholinergic receptor muscarinic 3	-0.349	0.977	0.023

672015093	VWA2	von Willebrand factor A domain containing 2	-0.344	0.952	0.048
50511312	POFUT1	protein O-fucosyltransferase 1	-0.343	0.994	0.006
77157795	MAL2	mal, T cell differentiation protein 2 (gene/pseudogene)	-0.342	0.968	0.032
157820807	GCDH	glutaryl-CoA dehydrogenase	-0.339	0.955	0.045
11693162	INSIG1	insulin induced gene 1	-0.339	0.969	0.031
13786174	TIMELESS	timeless circadian regulator	-0.332	0.995	0.005
57527498	KLC4	kinesin light chain 4	-0.331	0.994	0.006
281604125	Fam50a/LOC100910130	family with sequence similarity 50, member A	-0.327	1.000	0.000
62078809	TNFAIP8L2	TNF alpha induced protein 8 like 2	-0.324	0.976	0.024
451172111	HINT3	histidine triad nucleotide binding protein 3	-0.321	0.989	0.011
19424260	CDC25B	cell division cycle 25B	-0.317	0.974	0.026
149041432	THY1	Thy-1 cell surface antigen	-0.313	0.961	0.039
62078551	GNB4	G protein subunit beta 4	-0.309	0.994	0.006
149063353	IFT81	intraflagellar transport 81	-0.309	0.962	0.038
61556910	SNX10	sorting nexin 10	-0.306	0.990	0.010
31982028	RSU1	Ras suppressor protein 1	-0.304	0.978	0.022
56090361	EPDR1	ependymin related 1	-0.303	0.999	0.001
13540624	GRK5	G protein-coupled receptor kinase 5	-0.302	0.952	0.048
149030301	PNOC	prepronociceptin	-0.298	0.959	0.041
38259192	TOP2A	DNA topoisomerase II alpha	-0.297	0.961	0.039
672035779	Proser3	proline and serine rich 3	-0.293	0.996	0.004
83816933	AP4M1	adaptor related protein complex 4 subunit mu 1	-0.291	0.991	0.009
157817979	Egfem1	EGF-like and EMI domain containing 1	-0.291	0.954	0.046
672065746	SCLY	selenocysteine lyase	-0.291	0.969	0.031
672083256	MYO5B	myosin VB	-0.287	0.960	0.040
6981208	NR3C2	nuclear receptor subfamily 3 group C member 2	-0.286	0.954	0.046
157818065	GPR21	G protein-coupled receptor 21	-0.285	0.952	0.048
6754024	GNG4	G protein subunit gamma 4	-0.284	0.957	0.043
60097941	HP	haptoglobin	-0.279	0.990	0.010
157818005	HPS3	HPS3 biogenesis of lysosomal organelles complex 2 subunit 1	-0.277	0.970	0.030
56090313	MOCS2	molybdenum cofactor synthesis 2	-0.275	0.982	0.018
62079057	IL33	interleukin 33	-0.273	0.995	0.005
148701892	EBF1	EBF transcription factor 1	-0.270	0.988	0.012
205294	ME1	malic enzyme 1	-0.264	0.981	0.019
148703340	SERTM1	serine rich and transmembrane domain containing 1	-0.263	0.951	0.049

157821975	ZCCHC24	zinc finger CCHC-type containing 24	-0.263	0.956	0.044
148696365	AP5S1	adaptor related protein complex 5 subunit sigma 1	-0.262	0.973	0.027
157817017	MRPS16	mitochondrial ribosomal protein S16	-0.261	0.997	0.003
564382292	ANGEL2	angel homolog 2	-0.258	0.965	0.035
17865325	GLRB	glycine receptor beta	-0.256	0.958	0.042
148700512	NRSN1	neurensin 1	-0.256	0.997	0.003
56605728	TMEM218	transmembrane protein 218	-0.251	0.970	0.030
568959109	CEP164	centrosomal protein 164	-0.250	0.964	0.036
149046389	ARID5A	AT-rich interaction domain 5A	-0.249	0.992	0.008
6978435	ACADVL	acyl-CoA dehydrogenase very long chain	-0.249	0.954	0.046
298493223	TMEM132B	transmembrane protein 132B	-0.246	0.979	0.021
157819077	TRIM37	tripartite motif containing 37	-0.246	0.982	0.018
15805026	ZFAND6	zinc finger AN1-type containing 6	-0.245	0.954	0.046
300794684	MSH3	mutS homolog 3	-0.244	0.974	0.026
564369492	HJURP	Holliday junction recognition protein	-0.243	0.986	0.014
236467366	CGREF1	cell growth regulator with EF-hand domain 1	-0.241	0.970	0.030
57527332	PSPH	phosphoserine phosphatase	-0.241	0.972	0.028
58865958	RDH11	retinol dehydrogenase 11	-0.240	0.980	0.020
149033803	CDKL2	cyclin dependent kinase like 2	-0.237	0.951	0.049
449784888	ALDH5A1	aldehyde dehydrogenase 5 family member A1	-0.236	0.974	0.026
672047003	CDAN1	codanin 1	-0.236	0.996	0.004
157817480	RWDD2A	RWD domain containing 2A	-0.235	0.998	0.002
77404265	JAM2	junctional adhesion molecule 2	-0.233	0.994	0.006
13489067	NSF	N-ethylmaleimide sensitive factor, vesicle fusing ATPase	-0.231	0.969	0.031
197927216	TBC1D5	TBC1 domain family member 5	-0.230	0.975	0.025
73661200	SPRN	shadow of prion protein	-0.229	0.978	0.022
404434384	GALNT11	polypeptide N-acetylgalactosaminyltransferase 11	-0.229	0.953	0.047
564397761	GCC2	GRIP and coiled-coil domain containing 2	-0.229	0.964	0.036
157817710	FER	FER tyrosine kinase	-0.225	0.988	0.012
293345175	DHX29	DExH-box helicase 29	-0.225	0.971	0.029
564383995	EVC	EvC ciliary complex subunit 1	-0.225	0.994	0.006
148689145	CPNE4	copine 4	-0.222	0.974	0.026
288541382	DIS3L2	DIS3 like 3'-5' exoribonuclease 2	-0.214	0.967	0.033
205755	TAGLN3	transgelin 3	-0.213	0.992	0.008
564398053	MAN1A1	mannosidase alpha class 1A member 1	-0.213	0.989	0.011
57526927	LARS1	leucyl-tRNA synthetase 1	-0.213	0.964	0.036

392338550	IPO11	importin 11	-0.212	0.974	0.026
149058126	ALDH9A1	aldehyde dehydrogenase 9 family member A1	-0.208	0.961	0.039
148747414	GDA	guanine deaminase	-0.203	0.986	0.014
564391231	SERPINB9	serpin family B member 9	-0.203	0.954	0.046
18426866	ACAA2	acetyl-CoA acyltransferase 2	-0.203	0.990	0.010
451770389	HMGCLL1	3-hydroxymethyl-3-methylglutaryl-CoA lyase like 1	-0.202	0.961	0.039
55741502	ACAT2	acetyl-CoA acetyltransferase 2	-0.202	0.968	0.032
68163417	FAHD1	fumarylacetoacetate hydrolase domain containing 1	-0.201	0.977	0.023
309243082	PTPRJ	protein tyrosine phosphatase receptor type J	-0.201	0.989	0.011
189163499	CYHR1	cysteine and histidine rich 1	-0.198	0.981	0.019
11560055	KHDRBS3	KH RNA binding domain containing, signal transduction associated 3	-0.195	0.991	0.009
564334053	SORCS1	sortilin related VPS10 domain containing receptor 1	-0.195	0.978	0.022
62078931	PAQR8	progestin and adipoQ receptor family member 8	-0.192	0.995	0.005
17530977	ECHS1	enoyl-CoA hydratase, short chain 1	-0.191	0.968	0.032
293349793	RFTN2	raftlin family member 2	-0.189	0.953	0.047
148696094	TUBGCP4	tubulin gamma complex associated protein 4	-0.189	0.965	0.035
42491372	ERMP1	endoplasmic reticulum metallopeptidase 1	-0.189	0.953	0.047
19705437	EPHA7	EPH receptor A7	-0.186	0.988	0.012
157820421	SMIM17	small integral membrane protein 17	-0.183	0.975	0.025
13929208	Scd2	stearoyl-Coenzyme A desaturase 2	-0.178	0.989	0.011
124248495	CHID1	chitinase domain containing 1	-0.171	0.971	0.029
348041331	SERTAD2	SERTA domain containing 2	-0.169	0.994	0.006
149042395	PRDX4	peroxiredoxin 4	-0.169	0.990	0.010
19705545	RAB3IL1	RAB3A interacting protein like 1	-0.166	0.983	0.017
148747253	ATP1B1	ATPase Na+/K+ transporting subunit beta 1	-0.164	0.999	0.001
19173766	LONP1	lon peptidase 1, mitochondrial	-0.164	0.981	0.019
79750129	CAMK1D	calcium/calmodulin dependent protein kinase ID	-0.164	0.957	0.043
16758808	EPB41L3	erythrocyte membrane protein band 4.1 like 3	-0.159	0.992	0.008
62078637	LCA5	lebercilin LCA5	-0.158	0.991	0.009
564397835	ASCC1	activating signal cointegrator 1 complex subunit 1	-0.158	0.979	0.021
6649914	GDF11	growth differentiation factor 11	-0.154	0.953	0.047

149024719	NOL9	nucleolar protein 9	-0.150	0.991	0.009
149032539	HECW1	HECT, C2 and WW domain containing E3 ubiquitin protein ligase 1	-0.150	0.995	0.005
71043650	SRPK1	SRSF protein kinase 1	-0.150	0.955	0.045
755498773	ITGA6	integrin subunit alpha 6	-0.149	0.963	0.037
157819977	CERS4	ceramide synthase 4	-0.149	0.995	0.005
8394227	PTPRO	protein tyrosine phosphatase receptor type O	-0.148	0.980	0.020
48976085	GM2A	GM2 ganglioside activator	-0.147	0.988	0.012
148671875	TMEM50B	transmembrane protein 50B	-0.142	0.982	0.018
66730507	CCDC134	coiled-coil domain containing 134	-0.140	0.975	0.025
6978751	CYP51A1	cytochrome P450 family 51 subfamily A member 1	-0.138	0.990	0.010
77627979	SRPRA	SRP receptor subunit alpha	-0.138	0.985	0.015
564389848	ERICH1	glutamate rich 1	-0.137	0.990	0.010
68163557	CDCA8	cell division cycle associated 8	-0.136	0.954	0.046
672044529	MIGA1	mitoguardin 1	-0.135	0.970	0.030
56605798	RNF167	ring finger protein 167	-0.131	0.966	0.034
564351227	E130308A19Rik	RIKEN cDNA E130308A19 gene	-0.129	0.964	0.036
158711729	HACE1	HECT domain and ankyrin repeat containing E3 ubiquitin protein ligase 1	-0.128	0.997	0.003
253683488	NTRK2	neurotrophic receptor tyrosine kinase 2	-0.121	0.972	0.028
398303839	SH3GL2	SH3 domain containing GRB2 like 2, endophilin A1	-0.117	0.985	0.015
52138635	ETFDH	electron transfer flavoprotein dehydrogenase	-0.115	0.960	0.040
6978621	CCNG1	cyclin G1	-0.114	0.960	0.040
404247435	YLPM1	YLP motif containing 1	-0.113	0.956	0.044
56090463	GORASP2	golgi reassembly stacking protein 2	-0.113	0.983	0.017
157786602	NHP2	NHP2 ribonucleoprotein	-0.110	0.985	0.015
148673176	FABP7	fatty acid binding protein 7	-0.107	0.980	0.020
58865936	SIKE1	suppressor of IKBKE 1	-0.100	0.985	0.015
253970439	KCNA6	potassium voltage-gated channel subfamily A member 6	-0.096	0.953	0.047
672057488	CD63	CD63 molecule	-0.096	0.962	0.038
149060725	CEP19	centrosomal protein 19	-0.095	0.951	0.049
224549858	Poldip2	DNA polymerase delta interacting protein 2	-0.090	0.956	0.044
16258813	VHL	von Hippel-Lindau tumor suppressor	-0.089	0.997	0.003
6981370	PLCG1	phospholipase C gamma 1	-0.088	0.989	0.011
16758578	DPP3	dipeptidyl peptidase 3	-0.084	0.998	0.002

157821365	IFFO1	intermediate filament family orphan 1	-0.084	0.966	0.034
50054266	NLN	neurolysin	-0.082	0.989	0.011
564343911	RPN2	ribophorin II	-0.076	0.999	0.001
148747528	PTK2B	protein tyrosine kinase 2 beta	-0.066	0.952	0.048
564328458	Ldha/RGD1562690	lactate dehydrogenase A	-0.064	0.994	0.006
213688386	GTF2E1	general transcription factor IIE subunit 1	-0.058	0.998	0.002
149049470	TPI1	triosephosphate isomerase 1	-0.057	0.969	0.031
162287208	FADS1	fatty acid desaturase 1	-0.050	0.971	0.029
157821319	ATP6V0B	ATPase H+ transporting V0 subunit b	-0.045	0.982	0.018
149047197	STIM2	stromal interaction molecule 2	-0.023	0.977	0.023
157818483	GSPT2	G1 to S phase transition 2	0.024	-0.975	0.025
392333100	FAM193A	family with sequence similarity 193 member A	0.027	-0.971	0.029
58866026	XK	X-linked Kx blood group	0.033	-0.955	0.045
76443681	USP11	ubiquitin specific peptidase 11	0.053	-0.990	0.010
157817871	MEGF9	multiple EGF like domains 9	0.055	-0.967	0.033
149039803	UBQLN1	ubiquilin 1	0.065	-0.991	0.009
157822779	DNAJC11	DnaJ heat shock protein family (Hsp40) member C11	0.066	-0.954	0.046
37360568	RANGAP1	Ran GTPase activating protein 1	0.068	-0.962	0.038
157786896	FIS1	fission, mitochondrial 1	0.073	-0.981	0.019
564353714	FBXO42	F-box protein 42	0.074	-0.997	0.003
157822653	CD2BP2	CD2 cytoplasmic tail binding protein 2	0.075	-0.971	0.029
157817783	SNX18	sorting nexin 18	0.079	-0.994	0.006
169790975	MRPS9	mitochondrial ribosomal protein S9	0.082	-0.969	0.031
83649695	SMIM14	small integral membrane protein 14	0.082	-0.951	0.049
214010118	TMEM59	transmembrane protein 59	0.085	-0.995	0.005
290560659	ZNF609	zinc finger protein 609	0.090	-0.977	0.023
149038024	RIPOR1	RHO family interacting cell polarization regulator 1	0.091	-0.968	0.032
564332984	OSBP	oxysterol binding protein	0.094	-0.988	0.012
241666404	EPHA4	EPH receptor A4	0.100	-0.957	0.043
62543537	TBC1D10A	TBC1 domain family member 10A	0.108	-1.000	0.000
30794434	SRRM4	serine/arginine repetitive matrix 4	0.112	-0.998	0.002
157817811	C5orf22	chromosome 5 open reading frame 22	0.113	-0.998	0.002
170295834	NDUFA10	NADH:ubiquinone oxidoreductase subunit A10	0.114	-0.992	0.008
274321371	CRLF3	cytokine receptor like factor 3	0.118	-0.973	0.027
114326177	SHMT1	serine hydroxymethyltransferase 1	0.123	-0.994	0.006
392347634	CHD4	chromodomain helicase DNA binding protein 4	0.124	-0.953	0.047

454601639	NCOA6	nuclear receptor coactivator 6	0.127	-0.993	0.007
16758194	RGS2	regulator of G protein signaling 2	0.127	-0.985	0.015
564331450	EEF1AKMT2	EEF1A lysine methyltransferase 2	0.129	-0.991	0.009
564361462	BRD1	bromodomain containing 1	0.130	-0.986	0.014
672042705	FNIP2	folliculin interacting protein 2	0.130	-0.973	0.027
564363852	SNUPN	snurportin 1	0.131	-0.995	0.005
148693260	TIMM29	translocase of inner mitochondrial membrane 29	0.131	-0.962	0.038
162287067	VAV1	vav guanine nucleotide exchange factor 1	0.135	-0.971	0.029
19924073	TTL	tubulin tyrosine ligase	0.142	-0.970	0.030
157819431	BRD3	bromodomain containing 3	0.142	-0.962	0.038
564370907	ZNF598	zinc finger protein 598, E3 ubiquitin ligase	0.144	-0.953	0.047
6981458	RAF1	Raf-1 proto-oncogene, serine/threonine kinase	0.144	-0.961	0.039
291042494	MED13L	mediator complex subunit 13L	0.145	-0.986	0.014
157820585	SART3	spliceosome associated factor 3, U4/U6 recycling protein	0.146	-0.957	0.043
61556879	PKNOX1	PBX/knotted 1 homeobox 1	0.148	-0.966	0.034
281427188	ZC3H13	zinc finger CCCH-type containing 13	0.149	-0.976	0.024
564321656	TCF25	transcription factor 25	0.149	-0.977	0.023
40786455	BPGM	bisphosphoglycerate mutase	0.150	-0.968	0.032
157820865	DDX28	DEAD-box helicase 28	0.153	-0.961	0.039
77917548	DUS3L	dihydrouridine synthase 3 like	0.158	-0.956	0.044
19705483	CLSTN2	calsyntenin 2	0.160	-0.994	0.006
58219518	RND2	Rho family GTPase 2	0.161	-0.957	0.043
672072928	CUX2	cut like homeobox 2	0.164	-0.970	0.030
112984440	TNFRSF19	TNF receptor superfamily member 19	0.164	-0.976	0.024
293359997	SGPP1	sphingosine-1-phosphate phosphatase 1	0.170	-0.983	0.017
564392297	SLC39A12	solute carrier family 39 member 12	0.170	-0.974	0.026
568972665	TSPOAP1	TSPO associated protein 1	0.173	-0.999	0.001
148806879	DNTTIP1	deoxynucleotidyltransferase terminal interacting protein 1	0.174	-0.950	0.050
157823565	COQ10A	coenzyme Q10A	0.174	-0.954	0.046
37360004	KDM1A	lysine demethylase 1A	0.177	-0.970	0.030
564298823	EML3	EMAP like 3	0.177	-0.995	0.005
149054120	ORMDL3	ORMDL sphingolipid biosynthesis regulator 3	0.179	-0.957	0.043
149044006	TEDC1	tubulin epsilon and delta complex 1	0.179	-0.952	0.048
392333209	DLG5	discs large MAGUK scaffold protein 5	0.180	-0.965	0.035
164565360	CTTNBP2NL	CTTNBP2 N-terminal like	0.180	-0.966	0.034

157822303	GPR107	G protein-coupled receptor 107	0.182	-0.975	0.025
157817773	ZNF641	zinc finger protein 641	0.183	-0.962	0.038
392342217	RANBP3	RAN binding protein 3	0.184	-0.984	0.016
29789319	CBLB	Cbl proto-oncogene B	0.192	-0.958	0.042
157823197	NDUFB7	NADH:ubiquinone oxidoreductase subunit B7	0.194	-0.951	0.049
157822191	MTMR2	myotubularin related protein 2	0.195	-0.995	0.005
157821953	NXPE3	neurexophilin and PC-esterase domain family member 3	0.197	-0.959	0.041
148674304	RPRD1B	regulation of nuclear pre-mRNA domain containing 1B	0.198	-0.968	0.032
157821415	GZF1	GDNF inducible zinc finger protein 1	0.201	-0.954	0.046
157821579	BICD1	BICD cargo adaptor 1	0.202	-0.998	0.002
61557021	BFAR	bifunctional apoptosis regulator	0.204	-0.978	0.022
564383487	SLAIN2	SLAIN motif family member 2	0.206	-0.981	0.019
300794743	TSC22D2	TSC22 domain family member 2	0.206	-0.966	0.034
61556748	TSPYL1	TSPY like 1	0.211	-0.961	0.039
189163477	SCAF4	SR-related CTD associated factor 4	0.216	-0.971	0.029
124249254	ZNF639	zinc finger protein 639	0.217	-0.975	0.025
396080328	ADCYAP1R1	ADCYAP receptor type I	0.217	-0.997	0.003
66911118	NFX1	nuclear transcription factor, X-box binding 1	0.217	-0.999	0.001
564370219	LPIN2	lipin 2	0.218	-0.955	0.045
2804296	CDH8	cadherin 8	0.221	-0.953	0.047
293340917	C3orf70	chromosome 3 open reading frame 70	0.223	-0.973	0.027
568992461	DIP2B	disco interacting protein 2 homolog B	0.223	-0.968	0.032
125988381	JMJD6	jumonji domain containing 6, arginine demethylase and lysine hydroxylase	0.223	-0.963	0.037
117940043	MED22	mediator complex subunit 22	0.225	-0.959	0.041
148696931	ARRDC2	arrestin domain containing 2	0.227	-0.998	0.002
149032040	SLC11A2	solute carrier family 11 member 2	0.234	-0.982	0.018
40018556	NOB1	NIN1 (RPN12) binding protein 1 homolog	0.237	-0.998	0.002
162951835	CYTH1	cytohesin 1	0.237	-0.950	0.050
114145762	WDR83	WD repeat domain 83	0.238	-0.968	0.032
149023044	TMEM87A	transmembrane protein 87A	0.241	-0.966	0.034
157821413	USP30	ubiquitin specific peptidase 30	0.250	-0.992	0.008
157823719	TRAIP	TRAF interacting protein	0.252	-0.981	0.019
564328896	CHD2	chromodomain helicase DNA binding protein 2	0.258	-0.966	0.034
281427178	CEP76	centrosomal protein 76	0.262	-0.986	0.014
67078454	SLC25A51	solute carrier family 25 member 51	0.263	-0.964	0.036
564353880	DDI2	DNA damage inducible 1 homolog 2	0.264	-0.987	0.013
244792650	TNIK	TRAF2 and NCK interacting kinase	0.268	-0.957	0.043

148696370	PANK2	pantothenate kinase 2	0.270	-0.982	0.018
564365504	CCDC51	coiled-coil domain containing 51	0.277	-0.988	0.012
197386048	PTRHD1	peptidyl-tRNA hydrolase domain containing 1	0.280	-0.991	0.009
315259095	UBN1	ubinuclein 1	0.281	-0.979	0.021
32451765	FBXO10	F-box protein 10	0.283	-0.956	0.044
157821325	TWNK	twinkle mtDNA helicase	0.283	-0.995	0.005
149052738	RGD1561277	RGD1561277	0.285	-0.972	0.028
166795897	PIMREG	PICALM interacting mitotic regulator	0.293	-0.952	0.048
56789732	VSTM5	V-set and transmembrane domain containing 5	0.295	-0.991	0.009
157822519	CBLN4	cerebellin 4 precursor	0.299	-0.986	0.014
51948532	TBC1D20	TBC1 domain family member 20	0.302	-0.994	0.006
157819365	TBC1D25	TBC1 domain family member 25	0.302	-0.959	0.041
56605628	SFT2D1	SFT2 domain containing 1	0.306	-0.963	0.037
157819315	OSBPL11	oxysterol binding protein like 11	0.306	-0.966	0.034
6680532	KCNJ3	potassium inwardly rectifying channel subfamily J member 3	0.308	-0.957	0.043
300797262	BRPF1	bromodomain and PHD finger containing 1	0.308	-0.966	0.034
51491900	TOR1A	torsin family 1 member A	0.308	-0.952	0.048
50510821	AMIGO1	adhesion molecule with Ig like domain 1	0.310	-0.990	0.010
33086606	SRPRB	SRP receptor subunit beta	0.318	-0.998	0.002
149051028	RNF144A	ring finger protein 144A	0.322	-0.977	0.023
17865345	CDH23	cadherin related 23	0.322	-0.974	0.026
41386747	ZC3H18	zinc finger CCCH-type containing 18	0.322	-0.975	0.025
672030183	H2AC12	H2A clustered histone 12	0.323	-0.956	0.044
76362828	TEF	TEF transcription factor, PAR bZIP family member	0.327	-0.970	0.030
672063138	IP6K2	inositol hexakisphosphate kinase 2	0.332	-0.954	0.046
582015198	CRY2	cryptochrome circadian regulator 2	0.332	-0.999	0.001
349732232	NFATC1	nuclear factor of activated T cells 1	0.335	-0.968	0.032
564307081	ATXN7L1	ataxin 7 like 1	0.339	-0.998	0.002
672055128	SAMD11	sterile alpha motif domain containing 11	0.342	-0.979	0.021
160333172	COG2	component of oligomeric golgi complex 2	0.346	-0.951	0.049
157822711	RBM28	RNA binding motif protein 28	0.353	-0.980	0.020
40018598	ANGPTL4	angiopoietin like 4	0.356	-0.987	0.013
672065395	CCNYL1	cyclin Y like 1	0.356	-0.984	0.016
564393142	WDR36	WD repeat domain 36	0.359	-0.978	0.022
57528321	RIOK2	RIO kinase 2	0.360	-0.971	0.029
74183022	Zfp773	zinc finger protein 773	0.360	-0.991	0.009

62543527	TGIF1	TGFB induced factor homeobox 1	0.362	-0.966	0.034
62642955	NIM1K	NIM1 serine/threonine protein kinase	0.364	-0.981	0.019
213972545	MXD1	MAX dimerization protein 1	0.378	-0.952	0.048
564326636	Zfp94	zinc finger protein 94	0.378	-0.977	0.023
564323985	LOC108348337	uncharacterized LOC108348337	0.382	-0.966	0.034
14388593	SPATA2	spermatogenesis associated 2	0.385	-0.979	0.021
564382837	LIN54	lin-54 DREAM MuvB core complex component	0.387	-0.955	0.045
8392855	ADCYAP1	adenylate cyclase activating polypeptide 1	0.397	-0.977	0.023
149025439	DICER1	dicer 1, ribonuclease III	0.398	-0.959	0.041
157821403	RASSF7	Ras association domain family member 7	0.399	-0.956	0.044
564318492	TASOR	transcription activation suppressor	0.403	-0.994	0.006
157817446	LINGO2	leucine rich repeat and Ig domain containing 2	0.403	-0.950	0.050
61889068	MXI1	MAX interactor 1, dimerization protein	0.405	-0.980	0.020
564311452	TMEM131	transmembrane protein 131	0.443	-0.953	0.047
293341533	LOC108348225	feline leukemia virus subgroup C receptor-related protein 1	0.451	-0.958	0.042
149041559	BUD13	BUD13 homolog	0.456	-0.956	0.044
845633640	TSSC4	tumor suppressing subtransferable candidate 4	0.458	-0.971	0.029
8392993	BMP3	bone morphogenetic protein 3	0.461	-0.976	0.024
157822327	ATG14	autophagy related 14	0.464	-0.957	0.043
255708448	KATNA1	katanin catalytic subunit A1	0.466	-0.956	0.044
213385320	LRTOMT	leucine rich transmembrane and O-methyltransferase domain containing	0.476	-0.975	0.025
70912374	CCNQ	cyclin Q	0.479	-0.993	0.007
89145411	SULT2B1	sulfotransferase family 2B member 1	0.524	-0.989	0.011
213688370	EXOSC7	exosome component 7	0.551	-0.969	0.031
219879771	PGAP3	post-GPI attachment to proteins phospholipase 3	0.560	-0.961	0.039
13928942	PER2	period circadian regulator 2	0.562	-0.951	0.049
62640766	GDPGP1	GDP-D-glucose phosphorylase 1	0.562	-0.967	0.033
149056609	DEDD2	death effector domain containing 2	0.565	-0.951	0.049
62078983	DNAJC28	DnaJ heat shock protein family (Hsp40) member C28	0.570	-0.962	0.038
511094004	RUNX2	RUNX family transcription factor 2	0.585	-0.951	0.049
564315183	CUX1	cut like homeobox 1	0.585	-0.977	0.023
24415396	GPR3	G protein-coupled receptor 3	0.604	-0.951	0.049
157822359	PELI2	pellino E3 ubiquitin protein ligase family member 2	0.620	-0.954	0.046

157820433	CPEB1	cytoplasmic polyadenylation element binding protein 1	0.624	-0.976	0.024
1083798	Bmpr1b	bone morphogenetic protein receptor type 1B	0.630	-0.999	0.001
197386066	ZNF784	zinc finger protein 784	0.633	-0.953	0.047
392354293	Hmgb3	high mobility group box 3	0.709	-0.954	0.046
80861398	CRY1	cryptochrome circadian regulator 1	0.720	-0.973	0.027
293339965	RAB11FIP3	RAB11 family interacting protein 3	0.750	-0.951	0.049
293348634	LRIG3	leucine rich repeats and immunoglobulin like domains 3	0.825	-0.993	0.007
189011634	ARMC7	armadillo repeat containing 7	0.850	-0.996	0.004
78097110	N4BP2L1	NEDD4 binding protein 2 like 1	0.885	-0.979	0.021
672025117	MBTD1	mbt domain containing 1	0.886	-0.998	0.002
392339806	CFAP69	cilia and flagella associated protein 69	0.915	-0.967	0.033
9506775	HES2	hes family bHLH transcription factor 2	0.963	-0.951	0.049
157821687	NEURL2	neuralized E3 ubiquitin protein ligase 2	0.966	-0.971	0.029
148693657	DDX6	DEAD-box helicase 6	0.969	-0.982	0.018
148235584	CLEC4A	C-type lectin domain family 4 member A	1.000	-0.951	0.049
149065466	ARHGEF5	Rho guanine nucleotide exchange factor 5	1.000	-0.951	0.049
404501522	NXNL1	nucleoredoxin like 1	1.037	-0.951	0.049
19424314	KCNE2	potassium voltage-gated channel subfamily E regulatory subunit 2	1.066	-0.979	0.021
564296988	ZNF235	zinc finger protein 235	1.072	-0.954	0.046
157818609	NT5C1A	5'-nucleotidase, cytosolic IA	1.075	-0.971	0.029
7242211	TRH	thyrotropin releasing hormone	1.113	-0.969	0.031
62078917	PAQR5	progestin and adipoQ receptor family member 5	1.181	-0.963	0.037
6978493	ALOX5	arachidonate 5-lipoxygenase	1.193	-0.958	0.042
19173800	Actn3	actinin alpha 3	1.415	-0.952	0.048
11024678	Dbil5	diazepam binding inhibitor-like 5	1.423	-0.951	0.049
157817264	ANKRD23	ankyrin repeat domain 23	1.505	-0.983	0.017
158533972	SPTA1	spectrin alpha, erythrocytic 1	1.585	-0.951	0.049
149058209	SELE	selectin E	1.585	-0.951	0.049
41054896	FUT7	fucosyltransferase 7	1.700	-0.998	0.002
564318930	WDR17	WD repeat domain 17	1.807	-0.999	0.001
157821823	Ngp	neutrophilic granule protein	1.807	-0.999	0.001
157818127	CA7	carbonic anhydrase 7	1.824	-0.972	0.028
149042883	LOC100365365	rCG32328-like	1.861	-0.986	0.014
197381585	Urah	urate (5-hydroxyiso-) hydrolase	1.976	-0.951	0.049
755495595	PRRC2C	proline rich coiled-coil 2C	1.981	-0.980	0.020

160961485	MYLK3	myosin light chain kinase 3	2.000	-0.951	0.049
564312627	ZFP62	ZFP62 zinc finger protein	2.139	-0.967	0.033
28972866	CSMD3	CUB and Sushi multiple domains 3	2.140	-0.951	0.049
392340509	PTPRD	protein tyrosine phosphatase receptor type D	2.231	-0.964	0.036
197385133	RGD1561157	RGD1561157	2.303	-1.000	0.000
56270329	ATP1A4	ATPase Na+/K+ transporting subunit alpha 4	2.322	-0.955	0.045
71896592	IGFALS	insulin like growth factor binding protein acid labile subunit	2.322	-0.951	0.049
8393941	PADI4	peptidyl arginine deiminase 4	2.322	-0.951	0.049
564324736	L3MBTL3	L3MBTL histone methyl-lysine binding protein 3	2.353	-0.962	0.038
157818163	POF1B	POF1B actin binding protein	2.392	-0.985	0.015
149034139	TMEM273	transmembrane protein 273	2.406	-0.956	0.044
293356488	RIC1	RIC1 homolog, RAB6A GEF complex partner 1	2.479	-0.961	0.039
13994175	FUT2	fucosyltransferase 2	2.585	-0.980	0.020
564329376	SRPK3	SRSF protein kinase 3	2.585	-0.951	0.049
58866038	XKRX	XK related X-linked	2.585	-0.951	0.049
11120690	NR1H4	nuclear receptor subfamily 1 group H member 4	2.585	-0.951	0.049
13540693	MYOC	myocilin	2.585	-0.951	0.049
57222314	OAS3	2'-5'-oligoadenylate synthetase 3	2.585	-0.951	0.049
8394529	VDR	vitamin D receptor	2.585	-0.951	0.049
9507065	SCN11A	sodium voltage-gated channel alpha subunit 11	2.755	-0.961	0.039
25742760	AMH	anti-Mullerian hormone	2.807	-0.951	0.049
157787002	Dpt	dermatopontin	2.807	-0.951	0.049
197384923	C1orf87	chromosome 1 open reading frame 87	3.000	-0.951	0.049
13591993	MMP9	matrix metallopeptidase 9	3.000	-0.951	0.049
300796937	ESPNL	espin like	3.000	-0.951	0.049
564347547	LOC103690120	probable N-acetyltransferase CML1	3.030	-0.980	0.020
149020413	Zfp599	zinc finger protein 599	3.158	-0.987	0.013
8393891	P2RY2	purinergic receptor P2Y2	3.170	-0.985	0.015
281332212	SH2D4B	SH2 domain containing 4B	3.170	-0.951	0.049
260099641	MSH5	mutS homolog 5	3.170	-0.951	0.049
564392795	MOCOS	molybdenum cofactor sulfurase	3.248	-0.997	0.003
61556961	THEG	theg spermatid protein	3.322	-0.951	0.049
50370130	PALLD	palladin, cytoskeletal associated protein	3.361	-0.956	0.044
569009290	TENM1	teneurin transmembrane protein 1	3.450	-0.989	0.011
25282405	BPIFA1	BPI fold containing family A member 1	3.459	-0.951	0.049

16758550	BCL2L10	BCL2 like 10	3.459	-0.951	0.049
148670929	BATF	basic leucine zipper ATF-like transcription factor	3.700	-0.951	0.049
62078779	ORAI3	ORAI calcium release-activated calcium modulator 3	3.807	-0.995	0.005
21245088	Ly6a (includes others)	lymphocyte antigen 6 complex, locus A	3.807	-0.951	0.049
148747510	BAAT	bile acid-CoA:amino acid N-acyltransferase	3.807	-0.951	0.049
158187515	OAZ3	ornithine decarboxylase antizyme 3	3.807	-0.951	0.049
672052120	RBM12B	RNA binding motif protein 12B	3.907	-1.000	0.000
166157542	PABPN1L	PABPN1 like, cytoplasmic	3.954	-0.984	0.016
564309734	IGSF9B	immunoglobulin superfamily member 9B	4.173	-0.995	0.005
47577861	OR7D2	olfactory receptor family 7 subfamily D member 2	4.248	-0.992	0.008
8393641	AADAT	aminoadipate aminotransferase	4.248	-0.951	0.049
11024666	NTRK1	neurotrophic receptor tyrosine kinase 1	4.285	-0.989	0.011
165970757	H2-T24	histocompatibility 2, T region locus 24	4.285	-0.952	0.048
27545443	CEACAM4	CEA cell adhesion molecule 4	4.392	-0.985	0.015
298231202	PRG2	proteoglycan 2, pro eosinophil major basic protein	4.426	-0.987	0.013
341940965	MOS	MOS proto-oncogene, serine/threonine kinase	4.492	-1.000	0.000
13928958	CRYBB3	crystallin beta B3	4.672	-0.990	0.010
9506733	GJB5	gap junction protein beta 5	4.907	-0.951	0.049
672020628	ATXN7L1	ataxin 7 like 1	5.157	-0.989	0.011
59709455	EPOR	erythropoietin receptor	5.170	-0.998	0.002
8394516	PLAUR	plasminogen activator, urokinase receptor	5.229	-0.999	0.001
16758218	Hamp	hepcidin antimicrobial peptide	5.267	-0.956	0.044
20301998	PROK2	prokineticin 2	5.358	-0.990	0.010
293347435	PTPRD	protein tyrosine phosphatase receptor type D	7.710	-0.969	0.031
149057336	ZSCAN2	zinc finger and SCAN domain containing 2	7.758	-0.990	0.010
62650795	DACT1	dishevelled binding antagonist of beta catenin 1	7.762	-0.984	0.016
109472884	UBE3C	ubiquitin protein ligase E3C	8.197	-0.963	0.037
672029702	TUT7	terminal uridylyl transferase 7	8.441	-0.995	0.005

Supplementary Table S7. The list of genes that are differentially expressed in the offspring hippocampus in response to prenatal BPA exposure that exhibited the changes in the expression levels correlated with the neuronal density in the granular cell layer of the dentate gyrus. The transcriptome profiling data of DEGs in male and female rat offspring prenatally exposed to BPA (n = 6, male pups n = 3 and female pups n = 3, from independent litters) or the vehicle control (n = 6, male pups n = 3 and female pups n = 3, from independent litters) were obtained and used for the PTM analyses to identify DEGs that exhibited the changes in the expression levels correlated with the neuronal density in the granular cell layer of the dentate gyrus.

ID	Symbol	Entrez Gene Name	log2(FC)	R values	P-values
281371353	IL17B	interleukin 17B	-1.507	0.967	0.033
157822647	CD70	CD70 molecule	-1.492	0.966	0.034
157820541	RGS9BP	regulator of G protein signaling 9 binding protein	-1.480	0.971	0.029
564352410	ARTN	artemin	-1.379	0.971	0.029
157952196	Tmem125	transmembrane protein 125	-1.379	0.971	0.029
282847351	LRRC36	leucine rich repeat containing 36	-1.322	0.952	0.048
71043730	VNN1	vanin 1	-1.322	0.952	0.048
157819313	RGD1561661	similar to Ferritin light chain (Ferritin L subunit)	-1.206	0.990	0.010
157824077	CCRL2	C-C motif chemokine receptor like 2	-1.206	0.990	0.010
157819399	NOXO1	NADPH oxidase organizer 1	-1.206	0.990	0.010
58865684	MCOLN3	mucolipin 3	-1.158	0.989	0.011
13929148	CNGB1	cyclic nucleotide gated channel subunit beta 1	-1.000	0.971	0.029
198386353	MYO1G	myosin IG	-1.000	0.986	0.014
157819701	Ctla2a	cytotoxic T lymphocyte-associated protein 2 alpha	-0.966	0.980	0.020
564382020	TMEM63A	transmembrane protein 63A	-0.896	0.955	0.045
672022227	RGS22	regulator of G protein signaling 22	-0.855	0.974	0.026
119226204	CFAP206	cilia and flagella associated protein 206	-0.849	0.953	0.047
162287322	LSP1	lymphocyte specific protein 1	-0.781	0.989	0.011
66730425	MGC105567	similar to cDNA sequence BC023105	-0.755	0.981	0.019
157822593	NEIL2	nei like DNA glycosylase 2	-0.753	0.964	0.036
319009550	PPM1N	protein phosphatase, Mg2+/Mn2+ dependent 1N (putative)	-0.750	0.979	0.021
760997729	SYNPO2L	synaptopodin 2 like	-0.621	0.956	0.044
291463305	SHISA9	shisa family member 9	-0.574	0.968	0.032
188497675	RADX	RPA1 related single stranded DNA binding protein, X-linked	-0.542	0.968	0.032
17105368	KLF9	Kruppel like factor 9	-0.505	0.953	0.047
157824113	GPR84	G protein-coupled receptor 84	-0.485	0.971	0.029

672084224	CCDC113	coiled-coil domain containing 113	-0.458	0.988	0.012
194440693	Maml2	mastermind like transcriptional coactivator 2	-0.457	0.988	0.012
564346692	GIMAP8	GTPase, IMAP family member 8	-0.455	0.953	0.047
6978629	CD38	CD38 molecule	-0.440	0.962	0.038
157824150	PTPN22	protein tyrosine phosphatase non-receptor type 22	-0.389	0.972	0.028
8393919	LOC100911216/Pcsk1	proprotein convertase subtilisin/kexin type 1	-0.371	0.951	0.049
26024221	PRSS12	serine protease 12	-0.366	0.973	0.027
56605758	THAP1	THAP domain containing 1	-0.340	0.980	0.020
70794782	RBMS2	RNA binding motif single stranded interacting protein 2	-0.316	0.967	0.033
12621142	RASSF9	Ras association domain family member 9	-0.299	0.953	0.047
157822577	MAN1C1	mannosidase alpha class 1C member 1	-0.292	0.984	0.016
31560385	RPL21	ribosomal protein L21	-0.252	0.974	0.026
157822599	GSAP	gamma-secretase activating protein	-0.237	0.963	0.037
62078935	FLACC1	flagellum associated containing coiled-coil domains 1	-0.233	0.975	0.025
9506425	BET1	Bet1 golgi vesicular membrane trafficking protein	-0.215	0.955	0.045
672089580	LOC103694865	TATA-binding protein-associated factor 2N-like	-0.197	0.951	0.049
123780073	YOD1	YOD1 deubiquitinase	-0.193	0.974	0.026
300797496	TDRD6	tudor domain containing 6	-0.184	0.990	0.010
112984202	FZD8	frizzled class receptor 8	-0.183	0.958	0.042
269954719	JAZF1	JAZF zinc finger 1	-0.171	0.961	0.039
157822019	ITGB1BP1	integrin subunit beta 1 binding protein 1	-0.166	0.987	0.013
53850598	DDX59	DEAD-box helicase 59	-0.134	0.955	0.045
62078739	TCTA	T cell leukemia translocation altered	-0.131	0.980	0.020
300794591	FXN	frataxin	-0.110	0.985	0.015
81884516	Rhno1	RAD9-HUS1-RAD1 interacting nuclear orphan 1	-0.107	0.968	0.032
300794891	DDX20	DEAD-box helicase 20	-0.060	0.950	0.050
157817861	NDUFA2	NADH:ubiquinone oxidoreductase subunit A2	-0.057	0.958	0.042
188536098	SLC48A1	solute carrier family 48 member 1	-0.055	0.986	0.014
157820373	ELK1	ETS transcription factor ELK1	-0.043	0.954	0.046
149045755	CREB3	cAMP responsive element binding protein 3	-0.043	0.968	0.032

148686551	PPWD1	peptidylprolyl isomerase domain and WD repeat containing 1	0.038	-0.987	0.013
149049696	MKRN2	makorin ring finger protein 2	0.052	-0.976	0.024
149052177	MRPL28	mitochondrial ribosomal protein L28	0.052	-0.986	0.014
50511039	GNB1L	G protein subunit beta 1 like	0.054	-0.964	0.036
157819325	SRP68	signal recognition particle 68	0.056	-0.981	0.019
148672705	TMEM184B	transmembrane protein 184B	0.080	-0.965	0.035
157818421	TVP23A	trans-golgi network vesicle protein 23 homolog A	0.085	-0.983	0.017
564388185	ERCC6	ERCC excision repair 6, chromatin remodeling factor	0.112	-0.959	0.041
189011602	NLE1	notchless homolog 1	0.118	-0.977	0.023
11968114	MRPL23	mitochondrial ribosomal protein L23	0.127	-0.977	0.023
564361244	TCF20	transcription factor 20	0.128	-0.960	0.040
157821561	ATRIP	ATR interacting protein	0.132	-0.987	0.013
11177894	TSC1	TSC complex subunit 1	0.134	-0.959	0.041
157819423	SPSB3	splA/ryanodine receptor domain and SOCS box containing 3	0.150	-0.974	0.026
50511177	SLITRK1	SLIT and NTRK like family member 1	0.155	-0.968	0.032
157820917	CDC7	cell division cycle 7	0.156	-0.977	0.023
74200325	UBE2G2	ubiquitin conjugating enzyme E2 G2	0.158	-0.951	0.049
11559951	NRBF2	nuclear receptor binding factor 2	0.163	-0.987	0.013
300797915	Rbm33	RNA binding motif protein 33	0.166	-0.964	0.036
46485387	NAPRT	nicotinate phosphoribosyltransferase	0.167	-0.962	0.038
40807349	DSTYK	dual serine/threonine and tyrosine protein kinase	0.172	-0.957	0.043
392355126	HAUS2	HAUS augmin like complex subunit 2	0.175	-0.961	0.039
564303143	KMT2C*	lysine methyltransferase 2C	0.176	-0.981	0.019
38454226	TPD52L2	TPD52 like 2	0.180	-0.973	0.027
8393959	PIM1	Pim-1 proto-oncogene, serine/threonine kinase	0.188	-0.981	0.019
9507235	UGT8	UDP glycosyltransferase 8	0.196	-0.971	0.029
672031398	ANKRD11	ankyrin repeat domain 11	0.203	-0.973	0.027
149030718	PIP5K1A	phosphatidylinositol-4-phosphate 5-kinase type 1 alpha	0.211	-0.971	0.029
109470195	TNKS1BP1	tankyrase 1 binding protein 1	0.213	-0.957	0.043
189181698	ZNF131	zinc finger protein 131	0.225	-0.979	0.021
19173786	SYF2	SYF2 pre-mRNA splicing factor	0.229	-0.960	0.040
67078478	NAF1	nuclear assembly factor 1 ribonucleoprotein	0.237	-0.978	0.022
256818763	PLEKHH1	pleckstrin homology, MyTH4 and FERM domain containing H1	0.252	-0.966	0.034

148666908	ADAMTS9	ADAM metallopeptidase with thrombospondin type 1 motif 9	0.256	-0.955	0.045
205830446	C11orf98	chromosome 11 open reading frame 98	0.275	-0.990	0.010
109464919	ARHGEF26	Rho guanine nucleotide exchange factor 26	0.278	-0.951	0.049
145553978	SFMBT1	Scm like with four mbt domains 1	0.297	-0.971	0.029
293349725	AMER3	APC membrane recruitment protein 3	0.303	-0.957	0.043
72255513	AGA	aspartylglucosaminidase	0.309	-0.950	0.050
149048116	KHDC4	KH domain containing 4, pre-mRNA splicing factor	0.314	-0.951	0.049
76096340	ANKRD16	ankyrin repeat domain 16	0.354	-0.958	0.042
16758666	TIMP1	TIMP metallopeptidase inhibitor 1	0.376	-0.961	0.039
13928944	P2RY4	pyrimidinergic receptor P2Y4	0.415	-0.967	0.033
67078462	SOX18	SRY-box transcription factor 18	0.460	-0.954	0.046
157819737	SARS2	seryl-tRNA synthetase 2, mitochondrial	0.463	-0.970	0.030
281306771	ADAMTS4	ADAM metallopeptidase with thrombospondin type 1 motif 4	0.489	-0.968	0.032
300798653	ALPK3	alpha kinase 3	0.515	-0.979	0.021
564303143	KMT2C*	lysine methyltransferase 2C	0.593	-0.956	0.044
148683584	VEPH1	ventricular zone expressed PH domain containing 1	0.603	-0.973	0.027
672014266	TMEM219	transmembrane protein 219	0.735	-0.969	0.031
6978663	CLCN1	chloride voltage-gated channel 1	0.742	-0.969	0.031
293347270	OSGIN2	oxidative stress induced growth inhibitor family member 2	0.861	-0.955	0.045
672088752	MCF2	MCF.2 cell line derived transforming sequence	0.909	-0.957	0.043
157820193	Tbx2	T-box transcription factor 2	0.909	-0.967	0.033
564378170	PAN3	poly(A) specific ribonuclease subunit PAN3	0.932	-0.986	0.014
53734355	P2RY14	purinergic receptor P2Y14	1.000	-0.990	0.010
61097928	SNAI1	snail family transcriptional repressor 1	1.053	-0.952	0.048
564306247	PHACTR4	phosphatase and actin regulator 4	1.189	-0.985	0.015
40786461	NAPEPLD	N-acyl phosphatidylethanolamine phospholipase D	1.199	-0.961	0.039
7106248	ANKRD1	ankyrin repeat domain 1	1.252	-0.955	0.045
13928802	CCN5	cellular communication network factor 5	1.322	-0.971	0.029
140969796	Cyp2c23	cytochrome P450, family 2, subfamily c, polypeptide 23	1.363	-0.981	0.019
672084625	LOC100909409 (includes others)	RGD1562660	1.407	-0.979	0.021

56606094	Aox2	aldehyde oxidase 2	1.459	-0.986	0.014
54312124	HCST	hematopoietic cell signal transducer	1.554	-0.975	0.025
23463269	NPB	neuropeptide B	1.609	-0.975	0.025
204595	H1f4	H1.4 linker histone, cluster member	1.688	-0.974	0.026
408407614	DNA2	DNA replication helicase/nuclease 2	1.697	-0.979	0.021
254553399	FBXO24	F-box protein 24	1.716	-0.989	0.011
157786614	MFSD6L	major facilitator superfamily domain containing 6 like	1.716	-0.989	0.011
148230802	Akr1c12	aldo-keto reductase family 1, member C12	1.826	-0.989	0.011
207446700	Sec1	secretory blood group 1	1.826	-0.989	0.011
11024668	AIPL1	aryl hydrocarbon receptor interacting protein like 1	2.188	-0.974	0.026
158508517	SDS	serine dehydratase	2.202	-0.975	0.025
392342139	TTC21A	tetratricopeptide repeat domain 21A	2.389	-0.977	0.023
672071273	GRAMD1C	GRAM domain containing 1C	2.450	-0.957	0.043

Supplementary Table S8. The list of genes that are differentially expressed in the offspring hippocampus in response to prenatal BPA exposure that exhibited the changes in the expression levels correlated with the cell density in the hippocampus. The transcriptome profiling data of DEGs in male and female rat offspring prenatally exposed to BPA (n = 6, male pups n = 3 and female pups n = 3, from independent litters) or the vehicle control (n = 6, male pups n = 3 and female pups n = 3, from independent litters) were obtained and used for the PTM analyses to identify DEGs that exhibited the changes in the expression levels correlated with the cell density in the hippocampus.

ID	Symbol	Entrez Gene Name	log2(FC)	R values	P-values
564316241	CEP170	centrosomal protein 170	0.991	0.009	-8.600
564307173	HEATR5A	HEAT repeat containing 5A	0.988	0.012	-6.735
564310188	IGDCC4	immunoglobulin superfamily DCC subclass member 4	0.960	0.040	-6.728
672031167	C19orf57	chromosome 19 open reading frame 57	0.980	0.020	-6.476
149052470	ZNF454	zinc finger protein 454	0.982	0.018	-5.700
754169051	EPPIN	epididymal peptidase inhibitor	0.986	0.014	-5.066
114145748	LOC680227	LRRGT00193	0.959	0.041	-3.907
157819037	MBNL3	muscleblind like splicing regulator 3	0.991	0.009	-3.907
194474016	SLC30A8	solute carrier family 30 member 8	0.966	0.034	-3.807
8392926	ASGR2	asialoglycoprotein receptor 2	0.952	0.048	-3.700
72255533	ANXA8/ANXA8L1	annexin A8 like 1	0.990	0.010	-3.644
157823827	S1PR4	sphingosine-1-phosphate receptor 4	0.991	0.009	-3.392
148675704	TBX15	T-box transcription factor 15	0.966	0.034	-3.268
564324736	L3MBTL3	L3MBTL histone methyl-lysine binding protein 3	0.951	0.049	-3.262
564375434	CEP295NL	CEP295 N-terminal like	0.972	0.028	-3.248
62078965	SLC47A1	solute carrier family 47 member 1	1.000	0.000	-3.135
157817652	BNC2	basonuclin 2	0.978	0.022	-3.129
12408310	N5	DNA binding protein N5	0.976	0.024	-3.000
20302091	PLB1	phospholipase B1	0.966	0.034	-2.907
157821903	Slc7a15	solute carrier family 7 (cationic amino acid transporter, y+ system), member 15	0.966	0.034	-2.907
564355126	ADGRF3	adhesion G protein-coupled receptor F3	0.989	0.011	-2.907
157822377	ERICH4	glutamate rich 4	0.953	0.047	-2.716
156071424	Vom2r18 (includes others)	vomeronasal 2 receptor, 18	0.980	0.020	-2.700
564313782	Tha1	threonine aldolase 1	0.982	0.018	-2.683
148703035	CLDN11	claudin 11	0.992	0.008	-2.549

6978679	COMP	cartilage oligomeric matrix protein	0.988	0.012	-2.503
13928714	Ccl2	chemokine (C-C motif) ligand 2	0.991	0.009	-2.497
11120724	Sult1d1	sulfotransferase family 1D, member 1	0.953	0.047	-2.415
68163381	CALHM5	calcium homeostasis modulator family member 5	0.990	0.010	-2.392
112983998	C10orf62	chromosome 10 open reading frame 62	0.986	0.014	-2.273
300798413	FSD2	fibronectin type III and SPRY domain containing 2	0.976	0.024	-2.170
25742828	SCN7A	sodium voltage-gated channel alpha subunit 7	0.956	0.044	-2.129
148669850	GFRA1	GDNF family receptor alpha 1	0.965	0.035	-2.118
58331126	GJB6	gap junction protein beta 6	0.991	0.009	-2.104
156119589	FOXC2	forkhead box C2	0.995	0.005	-2.000
157818655	MPZL2	myelin protein zero like 2	0.977	0.023	-1.922
564380050	2410141K09Rik	RIKEN cDNA 2410141K09 gene	0.974	0.026	-1.920
58000421	Ggnbp1	gametogenetin binding protein 1	0.988	0.012	-1.918
19705467	Cyp2t4	cytochrome P450, family 2, subfamily t, polypeptide 4	0.956	0.044	-1.898
454526968	PDE4C	phosphodiesterase 4C	0.984	0.016	-1.874
11067389	BMP15	bone morphogenetic protein 15	0.991	0.009	-1.858
16758014	HPX	hemopexin	0.985	0.015	-1.830
300797073	CCDC27	coiled-coil domain containing 27	0.986	0.014	-1.778
57222306	Oas1f	2'-5' oligoadenylate synthetase 1F	0.983	0.017	-1.755
19424240	PCSK4	proprotein convertase subtilisin/kexin type 4	0.955	0.045	-1.740
480306394	Mcpt4	mast cell protease 4	0.963	0.037	-1.739
157816965	DKK2	dickkopf WNT signaling pathway inhibitor 2	0.979	0.021	-1.694
817473312	GATA6	GATA binding protein 6	0.989	0.011	-1.678
157822811	Fmo9	flavin containing monooxygenase 9	0.989	0.011	-1.678
157819871	EPB42	erythrocyte membrane protein band 4.2	0.991	0.009	-1.678
148671981	NFE2	nuclear factor, erythroid 2	0.965	0.035	-1.619
295391913	LOC100366054	Da1-10-like	0.964	0.036	-1.590
117647198	CFD	complement factor D	0.993	0.007	-1.585
62079089	MALL	mal, T cell differentiation protein like	0.979	0.021	-1.585
71896590	AOC3	amine oxidase copper containing 3	0.990	0.010	-1.585
194239635	Tpsab1	tryptase alpha/beta 1	0.982	0.018	-1.563
13929066	CPZ	carboxypeptidase Z	0.975	0.025	-1.549
158186711	F13A1	coagulation factor XIII A chain	0.980	0.020	-1.510
74202463	EYA2	EYA transcriptional coactivator and phosphatase 2	0.986	0.014	-1.497

194474002	MEI1	meiotic double-stranded break formation protein 1	0.989	0.011	-1.476
162138928	SLC13A3	solute carrier family 13 member 3	0.996	0.004	-1.467
16758434	DAO	D-amino acid oxidase	0.952	0.048	-1.459
18677739	CDKN2B	cyclin dependent kinase inhibitor 2B	0.984	0.016	-1.454
148704234	GJB2	gap junction protein beta 2	0.953	0.047	-1.451
157820117	FBXL8	F-box and leucine rich repeat protein 8	0.956	0.044	-1.441
6981148	LEP	leptin	0.981	0.019	-1.436
672019901	Oxct2a	3-oxoacid CoA transferase 2A	0.965	0.035	-1.420
157818369	Hils1	histone H1-like protein in spermatids 1	0.991	0.009	-1.411
19924087	Akr1c14	aldo-keto reductase family 1, member C14	0.989	0.011	-1.406
158187526	TFEC	transcription factor EC	0.965	0.035	-1.402
157823809	CD163	CD163 molecule	0.955	0.045	-1.385
149065466	ARHGEF5	Rho guanine nucleotide exchange factor 5	0.972	0.028	-1.382
157822063	Gm6377	predicted gene 6377	0.970	0.030	-1.379
48675870	PPP1R3B	protein phosphatase 1 regulatory subunit 3B	0.991	0.009	-1.379
166157470	PSTPIP1	proline-serine-threonine phosphatase interacting protein 1	0.985	0.015	-1.376
827012496	NLRC4	NLR family CARD domain containing 4	0.989	0.011	-1.346
157823345	LRR1	leucine rich repeat protein 1	0.952	0.048	-1.322
226958688	RBP4	retinol binding protein 4	0.994	0.006	-1.310
58865654	EFEMP1	EGF containing fibulin extracellular matrix protein 1	1.000	0.000	-1.284
57114286	HLA-DRB5	major histocompatibility complex, class II, DR beta 5	0.956	0.044	-1.284
66730461	Clec2d (includes others)	C-type lectin domain family 2, member D	0.989	0.011	-1.282
157821877	ATP8B1	ATPase phospholipid transporting 8B1	0.991	0.009	-1.248
6981068	ICAM1	intercellular adhesion molecule 1	0.995	0.005	-1.240
9506461	CAPN1	calpain 1	0.979	0.021	-1.237
58865664	SH2D4A	SH2 domain containing 4A	0.990	0.010	-1.204
155369293	AEBP1	AE binding protein 1	0.985	0.015	-1.198
568924481	COL25A1	collagen type XXV alpha 1 chain	0.973	0.027	-1.185
6981332	SERPINE1	serpin family E member 1	0.989	0.011	-1.184
13928752	PTGIS	prostaglandin I2 synthase	0.961	0.039	-1.183
71795615	UPP1	uridine phosphorylase 1	0.957	0.043	-1.167
218156285	CFB	complement factor B	0.982	0.018	-1.130

300798739	MYO3B	myosin IIIB	1.000	0.000	-1.100
149053909	COL1A1	collagen type I alpha 1 chain	0.980	0.020	-1.092
197385083	C1orf194	chromosome 1 open reading frame 194	0.956	0.044	-1.090
281371494	LAMC2	laminin subunit gamma 2	0.961	0.039	-1.087
18959230	SLC6A20	solute carrier family 6 member 20	0.986	0.014	-1.066
197384727	Smco4	single-pass membrane protein with coiled-coil domains 4	0.965	0.035	-1.065
189011701	PPP1R42	protein phosphatase 1 regulatory subunit 42	0.965	0.035	-1.044
157822059	HACD4	3-hydroxyacyl-CoA dehydratase 4	0.965	0.035	-1.031
7106240	AKR7A3	aldo-keto reductase family 7 member A3	0.977	0.023	-1.026
58865898	LIMS2	LIM zinc finger domain containing 2	0.999	0.001	-1.018
300795496	LAYN	layilin	0.987	0.013	-0.983
20302089	GABRR3	gamma-aminobutyric acid type A receptor subunit rho3 (gene/pseudogene)	0.987	0.013	-0.972
68163517	CCDC146	coiled-coil domain containing 146	0.998	0.002	-0.971
564340633	OLFML2A	olfactomedin like 2A	0.953	0.047	-0.968
6981176	MAK	male germ cell associated kinase	0.984	0.016	-0.931
157821719	CPM	carboxypeptidase M	0.992	0.008	-0.908
58865836	SP140	SP140 nuclear body protein	0.984	0.016	-0.903
16758080	COL1A2	collagen type I alpha 2 chain	0.974	0.026	-0.900
13540656	EMP3	epithelial membrane protein 3	0.990	0.010	-0.883
564377419	THPO	thrombopoietin	0.964	0.036	-0.882
564321163	CHD9	chromodomain helicase DNA binding protein 9	0.965	0.035	-0.868
201861483	LOC102548396 (includes others)	zinc finger protein 951	0.989	0.011	-0.848
157821541	ACSS3	acyl-CoA synthetase short chain family member 3	0.979	0.021	-0.830
61740621	RARRES2	retinoic acid receptor responder 2	0.990	0.010	-0.826
37591183	SLC10A6	solute carrier family 10 member 6	0.965	0.035	-0.807
157817065	KCNK16	potassium two pore domain channel subfamily K member 16	0.952	0.048	-0.807
564396113	ZCCHC14	zinc finger CCHC-type containing 14	0.954	0.046	-0.797
300798598	MYOF	myoferlin	0.995	0.005	-0.790
16758318	PDK4	pyruvate dehydrogenase kinase 4	0.954	0.046	-0.783
392352101	LRCH3	leucine rich repeats and calponin homology domain containing 3	0.992	0.008	-0.773
197385174	ENO4	enolase 4	0.991	0.009	-0.766
74177759	RPS2	ribosomal protein S2	0.991	0.009	-0.761
309319796	COL18A1	collagen type XVIII alpha 1 chain	0.994	0.006	-0.754

30794230	TNFRSF1B	TNF receptor superfamily member 1B	0.990	0.010	-0.749
255652942	EFCC1	EF-hand and coiled-coil domain containing 1	0.987	0.013	-0.747
13929084	THBD	thrombomodulin	0.987	0.013	-0.732
13929156	MYBPH	myosin binding protein H	0.966	0.034	-0.728
68163370	CARNMT1	carnosine N-methyltransferase 1	0.997	0.003	-0.724
402534539	ECRG4	ECRG4 augurin precursor	0.993	0.007	-0.723
66730475	Tpm2	tropomyosin 2, beta	0.989	0.011	-0.705
40018618	CBX7	chromobox 7	0.982	0.018	-0.698
281332190	APBB1IP	amyloid beta precursor protein binding family B member 1 interacting protein	0.994	0.006	-0.696
148671621	VIP	vasoactive intestinal peptide	0.980	0.020	-0.696
401461786	CP	ceruloplasmin	0.996	0.004	-0.695
564393107	STING1	stimulator of interferon response cGAMP interactor 1	0.970	0.030	-0.693
113206040	LRRC34	leucine rich repeat containing 34	0.996	0.004	-0.690
33286888	GJA1	gap junction protein alpha 1	0.988	0.012	-0.675
149039662	LAMA2	laminin subunit alpha 2	0.970	0.030	-0.670
305682588	PDZD7	PDZ domain containing 7	0.981	0.019	-0.660
300794803	SYNPO2	synaptopodin 2	0.990	0.010	-0.655
164565435	SYNJ2	synaptojanin 2	0.950	0.050	-0.654
281332078	TTLL2	tubulin tyrosine ligase like 2	0.965	0.035	-0.652
157822365	LAMC3	laminin subunit gamma 3	0.991	0.009	-0.651
13928928	NAPSA	napsin A aspartic peptidase	0.965	0.035	-0.644
148665664	PHLDB2	pleckstrin homology like domain family B member 2	0.993	0.007	-0.641
158508544	DDR2	discoidin domain receptor tyrosine kinase 2	0.998	0.002	-0.640
300794353	FANCL	FA complementation group L	0.959	0.041	-0.639
157786864	PHOSPHO1	phosphoethanolamine/phosphocholine phosphatase 1	0.975	0.025	-0.638
392355027	TANGO6	transport and golgi organization 6 homolog	0.989	0.011	-0.638
672060362	ELFN2	extracellular leucine rich repeat and fibronectin type III domain containing 2	0.957	0.043	-0.636
187282311	ISLR	immunoglobulin superfamily containing leucine rich repeat	0.971	0.029	-0.635
19424350	GBP2	guanylate binding protein 2	0.986	0.014	-0.635
392334060	LAMA3	laminin subunit alpha 3	0.982	0.018	-0.630
13591916	ABCC6	ATP binding cassette subfamily C member 6	0.986	0.014	-0.628
1763306	UNC13C	unc-13 homolog C	0.984	0.016	-0.626

564387543	UGGT2	UDP-glucose glycoprotein glucosyltransferase 2	0.974	0.026	-0.596
157817670	SLC2A10	solute carrier family 2 member 10	0.980	0.020	-0.595
149063098	LAT2	linker for activation of T cells family member 2	0.965	0.035	-0.585
164519095	SLC9A2	solute carrier family 9 member A2	0.999	0.001	-0.578
157820485	SLC9B2	solute carrier family 9 member B2	0.986	0.014	-0.576
19173754	TESK2	testis associated actin remodelling kinase 2	0.975	0.025	-0.567
564390348	Klhl3	kelch-like family member 3	0.966	0.034	-0.566
9845234	ANXA2	annexin A2	0.985	0.015	-0.565
8393469	S1PR2	sphingosine-1-phosphate receptor 2	0.980	0.020	-0.561
157821487	ANKRD34B	ankyrin repeat domain 34B	0.963	0.037	-0.559
569009290	TENM1	teneurin transmembrane protein 1	0.989	0.011	-0.559
300798350	LRRK1	leucine rich repeat kinase 1	0.955	0.045	-0.554
42476287	TGM2	transglutaminase 2	0.980	0.020	-0.553
16758788	PTPN6	protein tyrosine phosphatase non-receptor type 6	0.980	0.020	-0.552
300795183	SNTG1	syntrophin gamma 1	0.997	0.003	-0.543
815891318	ENTPD1	ectonucleoside triphosphate diphosphohydrolase 1	0.988	0.012	-0.541
75832132	ESYT1	extended synaptotagmin 1	0.962	0.038	-0.541
300794555	TMC7	transmembrane channel like 7	0.978	0.022	-0.531
46310239	SIDT1	SID1 transmembrane family member 1	0.977	0.023	-0.528
564319108	ADGRA2	adhesion G protein-coupled receptor A2	0.994	0.006	-0.527
157818843	EXTL1	exostosin like glycosyltransferase 1	0.987	0.013	-0.527
148678784	PTHLH	parathyroid hormone like hormone	0.981	0.019	-0.523
564350006	PREX2	phosphatidylinositol-3,4,5-trisphosphate dependent Rac exchange factor 2	0.992	0.008	-0.523
124244050	PPIP5K1	diphosphoinositol pentakisphosphate kinase 1	0.950	0.050	-0.522
392338379	SLC26A8	solute carrier family 26 member 8	0.960	0.040	-0.514
76159291	CAST	calpastatin	0.996	0.004	-0.514
157820973	RAB32	RAB32, member RAS oncogene family	0.965	0.035	-0.512
293344916	COL6A1	collagen type VI alpha 1 chain	0.988	0.012	-0.508
312922352	TTF2	transcription termination factor 2	0.993	0.007	-0.506
564399060	PIGA	phosphatidylinositol glycan anchor biosynthesis class A	0.998	0.002	-0.505
37693510	Bst2	bone marrow stromal cell antigen 2	0.977	0.023	-0.500
209529675	TXLNB	taxilin beta	0.991	0.009	-0.498

58865396	FIGNL1	fidgetin like 1	0.985	0.015	-0.497
84662732	DNASE1L1	deoxyribonuclease 1 like 1	0.960	0.040	-0.494
564301698	LY75	lymphocyte antigen 75	0.989	0.011	-0.489
281427229	COL6A2	collagen type VI alpha 2 chain	0.955	0.045	-0.485
13786160	SLC22A8	solute carrier family 22 member 8	0.965	0.035	-0.485
209954806	PIGN	phosphatidylinositol glycan anchor biosynthesis class N	0.993	0.007	-0.485
564399546	STARD8	StAR related lipid transfer domain containing 8	0.976	0.024	-0.484
13591971	HNMT	histamine N-methyltransferase	0.963	0.037	-0.481
6978505	ANXA5	annexin A5	0.971	0.029	-0.478
57528252	QPRT	quinolinate phosphoribosyltransferase	0.989	0.011	-0.475
392332443	PRKDC	protein kinase, DNA-activated, catalytic subunit	0.991	0.009	-0.475
16923978	SLC26A2	solute carrier family 26 member 2	0.960	0.040	-0.474
148695091	BBS5	Bardet-Biedl syndrome 5	0.969	0.031	-0.473
307078146	UACA	uveal autoantigen with coiled-coil domains and ankyrin repeats	0.999	0.001	-0.470
312922379	TNN	tenascin N	0.973	0.027	-0.469
157816939	WASHC3	WASH complex subunit 3	0.962	0.038	-0.469
19924069	SPON2	spondin 2	0.987	0.013	-0.467
75905809	AKAP12	A-kinase anchoring protein 12	0.961	0.039	-0.466
564380929	KCNT2	potassium sodium-activated channel subfamily T member 2	0.978	0.022	-0.465
148699893	COL6A1	collagen type VI alpha 1 chain	0.993	0.007	-0.457
149067372	MTERF2	mitochondrial transcription termination factor 2	0.957	0.043	-0.455
157819513	ABCA4	ATP binding cassette subfamily A member 4	0.952	0.048	-0.453
157820241	Marveld1	MARVEL domain containing 1	0.982	0.018	-0.452
40254754	OCLN	occludin	0.976	0.024	-0.450
201066407	EAPP	E2F associated phosphoprotein	0.985	0.015	-0.444
16758186	SLCO1C1	solute carrier organic anion transporter family member 1C1	0.976	0.024	-0.441
123782692	Cntnap5b	contactin associated protein-like 5B	0.980	0.020	-0.436
8393057	SERPINH1	serpin family H member 1	0.974	0.026	-0.436
172045714	MIIP	migration and invasion inhibitory protein	0.997	0.003	-0.433
187937018	ITPRIPL2	ITPRIP like 2	0.990	0.010	-0.428
564400410	AMOT	angiomotin	0.969	0.031	-0.428
157821557	CD248	CD248 molecule	0.981	0.019	-0.426
38454282	ETFBKMT	electron transfer flavoprotein subunit beta lysine methyltransferase	0.957	0.043	-0.424

76443683	LOC100912042/Surf2	surfeit 2	0.964	0.036	-0.423
157823279	CGNL1	cingulin like 1	0.972	0.028	-0.418
149057830	Hgsnat	heparan-alpha-glucosaminide N-acetyltransferase	0.989	0.011	-0.418
74218228	HNRNPC	heterogeneous nuclear ribonucleoprotein C	0.968	0.032	-0.418
564329859	COA4	cytochrome c oxidase assembly factor 4 homolog	0.967	0.033	-0.417
83642834	NAGK	N-acetylglucosamine kinase	0.965	0.035	-0.416
62078539	Pagr1	Paxip1-associated glutamate-rich protein 1	0.976	0.024	-0.415
564367529	ENPP4	ectonucleotide pyrophosphatase/phosphodiesterase 4	0.980	0.020	-0.414
68342019	LRRC17	leucine rich repeat containing 17	0.972	0.028	-0.413
672041704	NIPBL	NIPBL cohesin loading factor	0.994	0.006	-0.412
23463307	RIOX2	ribosomal oxygenase 2	0.954	0.046	-0.409
46402488	NOS3	nitric oxide synthase 3	0.998	0.002	-0.405
7949020	CDK2	cyclin dependent kinase 2	0.995	0.005	-0.405
149049048	RECQL	RecQ like helicase	0.962	0.038	-0.403
127140886	EML6	EMAP like 6	0.962	0.038	-0.403
77695926	STAT1	signal transducer and activator of transcription 1	0.975	0.025	-0.399
253683447	ETV1	ETS variant transcription factor 1	0.982	0.018	-0.398
564342244	NUTM1	NUT midline carcinoma family member 1	0.980	0.020	-0.390
124286858	B230217C12Rik	RIKEN cDNA B230217C12 gene	0.992	0.008	-0.389
564305413	E130308A19Rik	RIKEN cDNA E130308A19 gene	0.964	0.036	-0.381
672063869	MGC116197 (includes others)	similar to RIKEN cDNA 1700001E04	0.976	0.024	-0.378
219282679	ZNF43	zinc finger protein 43	0.954	0.046	-0.374
672083553	SLC14A1	solute carrier family 14 member 1 (Kidd blood group)	0.983	0.017	-0.373
157823373	TRHDE	thyrotropin releasing hormone degrading enzyme	0.969	0.031	-0.373
53850644	FAM151A	family with sequence similarity 151 member A	0.965	0.035	-0.369
157819753	RCN1	reticulocalbin 1	0.974	0.026	-0.368
53734563	ACCS	1-aminocyclopropane-1-carboxylate synthase homolog (inactive)	0.959	0.041	-0.367
149054665	ABCA9	ATP binding cassette subfamily A member 9	0.986	0.014	-0.364
219278723	ZNF23	zinc finger protein 23	0.990	0.010	-0.362
281604225	PUS7	pseudouridine synthase 7	0.991	0.009	-0.362

29293811	SERPINF1	serpin family F member 1	0.979	0.021	-0.359
672086023	HSF2BP	heat shock transcription factor 2 binding protein	0.981	0.019	-0.357
384368019	Snhg11	small nucleolar RNA host gene 11	0.984	0.016	-0.356
594191048	C19orf54	chromosome 19 open reading frame 54	0.998	0.002	-0.352
61557118	PCGF6	polycomb group ring finger 6	0.999	0.001	-0.351
149053793	TSPOAP1	TSPO associated protein 1	0.991	0.009	-0.349
148747194	SLC16A7	solute carrier family 16 member 7	0.998	0.002	-0.349
56119120	SNF8	SNF8 subunit of ESCRT-II	0.953	0.047	-0.343
162135927	DCLRE1C	DNA cross-link repair 1C	0.990	0.010	-0.338
157819569	TEAD2	TEA domain transcription factor 2	0.951	0.049	-0.333
77628027	PSMC3IP	PSMC3 interacting protein	0.952	0.048	-0.328
61557206	ZBTB16	zinc finger and BTB domain containing 16	0.976	0.024	-0.326
564368910	FN1	fibronectin 1	0.975	0.025	-0.321
50510855	RIMKLB	ribosomal modification protein rimK like family member B	0.996	0.004	-0.320
672033554	LOC102557335	uncharacterized LOC102557335	0.999	0.001	-0.319
564304076	FGD5	FYVE, RhoGEF and PH domain containing 5	0.973	0.027	-0.319
18426850	LCP2	lymphocyte cytosolic protein 2	1.000	0.000	-0.318
672050244	APLF	aprataxin and PNKP like factor	0.980	0.020	-0.318
148702301	CYB561	cytochrome b561	0.988	0.012	-0.317
157823879	NUDT12	nudix hydrolase 12	0.978	0.022	-0.317
157819457	MAP3K14	mitogen-activated protein kinase kinase kinase 14	0.967	0.033	-0.317
564318054	R3hcc1	R3H domain and coiled-coil containing 1	1.000	0.000	-0.316
300797242	SPG11	SPG11 vesicle trafficking associated, spatacsin	0.959	0.041	-0.316
57528352	DMAC2	distal membrane arm assembly complex 2	0.953	0.047	-0.315
281332095	RB1	RB transcriptional corepressor 1	0.969	0.031	-0.313
672084625	LOC100909409 (includes others)	RGD1562660	0.981	0.019	-0.312
157821393	LRRC20	leucine rich repeat containing 20	0.992	0.008	-0.312
68163523	TTC26	tetratricopeptide repeat domain 26	0.996	0.004	-0.311
109484871	HERC1	HECT and RLD domain containing E3 ubiquitin protein ligase family member 1	0.975	0.025	-0.310
545532952	EIF4E3	eukaryotic translation initiation factor 4E family member 3	0.963	0.037	-0.305
399124777	GLS2	glutaminase 2	0.966	0.034	-0.305

568961602	VPS13C	vacuolar protein sorting 13 homolog C	0.960	0.040	-0.304
149023323	GFRA4	GDNF family receptor alpha 4	0.976	0.024	-0.303
124107592	MYO1C	myosin IC	0.956	0.044	-0.298
19173736	SCPEP1	serine carboxypeptidase 1	0.953	0.047	-0.297
6978789	SPARCL1	SPARC like 1	0.997	0.003	-0.295
8393992	PMP22	peripheral myelin protein 22	0.985	0.015	-0.295
53850628	NDUFS1	NADH:ubiquinone oxidoreductase core subunit S1	0.995	0.005	-0.294
167560911	SGF29	SAGA complex associated factor 29	0.971	0.029	-0.293
27545388	ABCA5	ATP binding cassette subfamily A member 5	0.990	0.010	-0.291
402794599	PRR22	proline rich 22	0.965	0.035	-0.290
12844128	CCDC90B	coiled-coil domain containing 90B	0.990	0.010	-0.288
564329612	ME3	malic enzyme 3	0.990	0.010	-0.284
392338478	TTC37	tetratricopeptide repeat domain 37	0.988	0.012	-0.283
564367076	Mocs1	molybdenum cofactor synthesis 1	0.967	0.033	-0.281
77539442	EPHX1	epoxide hydrolase 1	0.971	0.029	-0.279
197927244	TIE1	tyrosine kinase with immunoglobulin like and EGF like domains 1	0.985	0.015	-0.278
13928886	MAP2K1	mitogen-activated protein kinase kinase 1	0.974	0.026	-0.278
21728400	Ggta1	glycoprotein galactosyltransferase alpha 1, 3	0.986	0.014	-0.278
18959266	KHDRBS2	KH RNA binding domain containing, signal transduction associated 2	0.994	0.006	-0.274
134948398	PDS5A	PDS5 cohesin associated factor A	0.993	0.007	-0.274
157822627	PLXDC2	plexin domain containing 2	0.989	0.011	-0.273
157820147	TNFRSF10A	TNF receptor superfamily member 10a	0.980	0.020	-0.272
392339871	PARP12	poly(ADP-ribose) polymerase family member 12	0.988	0.012	-0.270
158508684	BCAS1	brain enriched myelin associated protein 1	0.996	0.004	-0.270
157817953	RPGRIP1L	RPGRIP1 like	0.992	0.008	-0.269
219804406	DOCK1	dedicator of cytokinesis 1	0.964	0.036	-0.269
25742783	PLK1	polo like kinase 1	0.982	0.018	-0.269
149016965	GRB10	growth factor receptor bound protein 10	0.994	0.006	-0.268
149017535	HDAC10	histone deacetylase 10	0.976	0.024	-0.267
149041411	SC5D	sterol-C5-desaturase	0.969	0.031	-0.264
219275534	VPS13A	vacuolar protein sorting 13 homolog A	0.990	0.010	-0.264

149020634	TAF1D	TATA-box binding protein associated factor, RNA polymerase I subunit D	0.983	0.017	-0.262
564335541	Cplane1	ciliogenesis and planar polarity effector 1	0.979	0.021	-0.262
149060525	FSTL1	follistatin like 1	0.998	0.002	-0.258
114145534	Mtap	methylthioadenosine phosphorylase	0.955	0.045	-0.258
157787147	TEK	TEK receptor tyrosine kinase	0.995	0.005	-0.258
300795738	RASSF8	Ras association domain family member 8	1.000	0.000	-0.255
16758538	RASGRF2	Ras protein specific guanine nucleotide releasing factor 2	0.972	0.028	-0.254
50510975	RUFY2	RUN and FYVE domain containing 2	0.982	0.018	-0.253
18034793	GABRG1	gamma-aminobutyric acid type A receptor subunit gamma1	0.999	0.001	-0.252
392341280	NCAPG2	non-SMC condensin II complex subunit G2	0.965	0.035	-0.248
187469267	GPRC5B	G protein-coupled receptor class C group 5 member B	0.979	0.021	-0.247
61557172	GULP1	GULP PTB domain containing engulfment adaptor 1	0.972	0.028	-0.245
296439269	PHF10	PHD finger protein 10	0.999	0.001	-0.245
70794768	HDAC1	histone deacetylase 1	0.987	0.013	-0.244
12018300	AKAP6	A-kinase anchoring protein 6	0.983	0.017	-0.242
300793894	URB1	URB1 ribosome biogenesis homolog	0.950	0.050	-0.242
97537309	SYNJ1	synaptojanin 1	0.967	0.033	-0.240
309319799	EIF2AK4	eukaryotic translation initiation factor 2 alpha kinase 4	0.995	0.005	-0.239
57527084	HAT1	histone acetyltransferase 1	0.952	0.048	-0.238
119310200	KDM4D	lysine demethylase 4D	0.989	0.011	-0.237
51948438	PYROXD1	pyridine nucleotide-disulphide oxidoreductase domain 1	0.961	0.039	-0.237
48040475	GCNT2	glucosaminyl (N-acetyl) transferase 2 (I blood group)	1.000	0.000	-0.233
213688380	GXYLT1	glucoside xylosyltransferase 1	0.988	0.012	-0.232
306482651	DNAJB14	DnaJ heat shock protein family (Hsp40) member B14	0.987	0.013	-0.232
158261984	PSMG2	proteasome assembly chaperone 2	0.976	0.024	-0.231
62079099	ORC5	origin recognition complex subunit 5	0.971	0.029	-0.228
54312088	ATP2B4	ATPase plasma membrane Ca2+ transporting 4	0.997	0.003	-0.228
564384526	TACC3	transforming acidic coiled-coil containing protein 3	0.988	0.012	-0.226
672072960	KNTC1	kinetochore associated 1	0.955	0.045	-0.226
157818897	VANGL1	VANGL planar cell polarity protein 1	0.969	0.031	-0.223

13928806	P2RX4	purinergic receptor P2X 4	0.971	0.029	-0.223
392348438	NIPAL3	NIPA like domain containing 3	0.998	0.002	-0.223
155369680	Ces2b	carboxyesterase 2B	0.965	0.035	-0.222
56605770	RRP8	ribosomal RNA processing 8	0.981	0.019	-0.221
296010823	UBR1	ubiquitin protein ligase E3 component n-recognin 1	0.998	0.002	-0.220
158631207	YIF1A	Yip1 interacting factor homolog A, membrane trafficking protein	0.963	0.037	-0.220
158186672	Nedd4	neural precursor cell expressed, developmentally down-regulated 4	0.989	0.011	-0.220
564315812	NAV1	neuron navigator 1	0.959	0.041	-0.217
148697324	DEPTOR	DEP domain containing MTOR interacting protein	0.961	0.039	-0.216
564397593	RAB36	RAB36, member RAS oncogene family	0.998	0.002	-0.212
149066531	VPS13B	vacuolar protein sorting 13 homolog B	0.980	0.020	-0.212
25742763	HSPA5	heat shock protein family A (Hsp70) member 5	0.975	0.025	-0.211
281371490	LAMC1	laminin subunit gamma 1	0.999	0.001	-0.209
672065543	TNS1	tensin 1	0.985	0.015	-0.207
564385704	FLNB	filamin B	0.979	0.021	-0.207
300798704	TLL2	tolloid like 2	0.956	0.044	-0.206
157823243	FAM172A	family with sequence similarity 172 member A	0.951	0.049	-0.206
54019432	PCDHA7	protocadherin alpha 7	0.992	0.008	-0.205
6978673	CNR1	cannabinoid receptor 1	0.992	0.008	-0.205
564343174	PLCB4	phospholipase C beta 4	0.985	0.015	-0.204
6981504	ATXN1	ataxin 1	0.951	0.049	-0.204
157818191	SETD6	SET domain containing 6, protein lysine methyltransferase	0.977	0.023	-0.203
142385975	RNF25	ring finger protein 25	0.976	0.024	-0.202
148707634	SHISA4	shisa family member 4	1.000	0.000	-0.201
62078801	MEF2A	myocyte enhancer factor 2A	0.973	0.027	-0.199
61556891	OSBPL2	oxysterol binding protein like 2	0.965	0.035	-0.198
12018246	TSPAN2	tetraspanin 2	0.977	0.023	-0.195
564347675	AAK1	AP2 associated kinase 1	0.988	0.012	-0.195
58865716	PPP1R3C	protein phosphatase 1 regulatory subunit 3C	0.970	0.030	-0.194
13591979	LIFR	LIF receptor subunit alpha	0.965	0.035	-0.194
157818293	MMAA	metabolism of cobalamin associated A	0.990	0.010	-0.193
63706033	Gm5174 (includes others)	serine/threonine kinase, pseudogene 1	0.991	0.009	-0.192

18266684	MSMO1	methylsterol monooxygenase 1	0.994	0.006	-0.190
157819187	AGL	amylo-alpha-1, 6-glucosidase, 4-alpha-glucanotransferase	0.998	0.002	-0.189
157823948	ANO6	anoctamin 6	0.956	0.044	-0.187
815891121	ZEB1	zinc finger E-box binding homeobox 1	0.972	0.028	-0.187
149040053	C3orf14	chromosome 3 open reading frame 14	0.989	0.011	-0.187
300794608	CEP120	centrosomal protein 120	0.973	0.027	-0.186
157821901	PNMA3	PNMA family member 3	0.950	0.050	-0.186
17865335	DGAT1	diacylglycerol O-acyltransferase 1	0.987	0.013	-0.183
296010825	UBR2	ubiquitin protein ligase E3 component n-recognin 2	0.978	0.022	-0.182
56605784	MRRF	mitochondrial ribosome recycling factor	0.972	0.028	-0.182
84490431	DNM3	dynamin 3	0.978	0.022	-0.182
672088357	ZCCHC18	zinc finger CCHC-type containing 18	0.993	0.007	-0.180
19173794	LOC678813/Marf1	meiosis regulator and mRNA stability factor 1	0.992	0.008	-0.180
13592087	SOAT1	sterol O-acyltransferase 1	0.969	0.031	-0.180
6981672	Tpm4	tropomyosin 4	0.980	0.020	-0.178
16758644	TXN	thioredoxin	0.959	0.041	-0.178
13569846	PARVA	parvin alpha	0.983	0.017	-0.178
672017219	ZNF106	zinc finger protein 106	0.999	0.001	-0.177
61557085	SPTBN1	spectrin beta, non-erythrocytic 1	0.971	0.029	-0.174
392333710	COL4A2	collagen type IV alpha 2 chain	0.973	0.027	-0.173
18034783	ABCC3	ATP binding cassette subfamily C member 3	0.987	0.013	-0.170
281604190	INPP5B	inositol polyphosphate-5-phosphatase B	0.983	0.017	-0.169
6981076	IDE	insulin degrading enzyme	0.953	0.047	-0.165
157817971	FAM13B	family with sequence similarity 13 member B	0.998	0.002	-0.165
149046296	CREG2	cellular repressor of E1A stimulated genes 2	0.989	0.011	-0.165
124486885	LRRC7	leucine rich repeat containing 7	0.985	0.015	-0.162
392344250	SPTY2D1	SPT2 chromatin protein domain containing 1	0.999	0.001	-0.161
157819829	HACD3	3-hydroxyacyl-CoA dehydratase 3	0.979	0.021	-0.159
61556993	HIBCH	3-hydroxyisobutyryl-CoA hydrolase	0.999	0.001	-0.158
194474054	EFR3A	EFR3 homolog A	0.981	0.019	-0.157
62078609	Dync2li1	dynein cytoplasmic 2 light intermediate chain 1	0.988	0.012	-0.157
672022833	Scaper	S-phase cyclin A-associated protein in the ER	0.996	0.004	-0.157

48675845	ATIC	5-aminoimidazole-4-carboxamide ribonucleotide formyltransferase/IMP cyclohydrolase	0.982	0.018	-0.156
18266726	PAICS	phosphoribosylaminoimidazole carboxylase and phosphoribosylaminoimidazolesuccino carboxamide synthase	0.958	0.042	-0.155
158534079	CHRNA5	cholinergic receptor nicotinic alpha 5 subunit	0.973	0.027	-0.154
564385664	FERMT2	fermitin family member 2	0.960	0.040	-0.154
157817678	BUB1	BUB1 mitotic checkpoint serine/threonine kinase	0.952	0.048	-0.153
717324516	SCN8A	sodium voltage-gated channel alpha subunit 8	0.980	0.020	-0.147
672016955	MAP3K20	mitogen-activated protein kinase kinase kinase 20	0.998	0.002	-0.147
157820591	AVEN	apoptosis and caspase activation inhibitor	0.987	0.013	-0.146
55742755	CTNNA1	catenin alpha 1	0.996	0.004	-0.146
157820561	NAT10	N-acetyltransferase 10	0.958	0.042	-0.144
564347453	ALMS1	ALMS1 centrosome and basal body associated protein	0.975	0.025	-0.144
281485631	NAA38	N-alpha-acetyltransferase 38, NatC auxiliary subunit	0.953	0.047	-0.143
148693879	WDR61	WD repeat domain 61	0.984	0.016	-0.133
6981166	PLAGL1	PLAG1 like zinc finger 1	0.986	0.014	-0.133
9507177	USO1	USO1 vesicle transport factor	0.996	0.004	-0.130
8394354	SQLE	squalene epoxidase	0.992	0.008	-0.129
144445950	XRCC6	X-ray repair cross complementing 6	0.967	0.033	-0.129
149019723	PPIL2	peptidylprolyl isomerase like 2	0.985	0.015	-0.129
189181730	PRKD3	protein kinase D3	0.987	0.013	-0.128
19924067	NME7	NME/NM23 family member 7	0.983	0.017	-0.128
163659911	GRIK1	glutamate ionotropic receptor kainate type subunit 1	0.982	0.018	-0.126
564382183	EPRS1	glutamyl-prolyl-tRNA synthetase 1	0.991	0.009	-0.126
55926219	DDX39A	DExD-box helicase 39A	0.986	0.014	-0.126
149053938	SLC35B1	solute carrier family 35 member B1	0.953	0.047	-0.125
281604211	RAB3GAP2	RAB3 GTPase activating non-catalytic protein subunit 2	0.966	0.034	-0.123
157817043	ACOT13	acyl-CoA thioesterase 13	0.995	0.005	-0.122
564301782	CERS6	ceramide synthase 6	0.997	0.003	-0.122
149047238	ADGRA3	adhesion G protein-coupled receptor A3	0.985	0.015	-0.118

476007242	EPS8	epidermal growth factor receptor pathway substrate 8	0.961	0.039	-0.117
293339963	RAB11FIP3	RAB11 family interacting protein 3	0.999	0.001	-0.116
157823994	PNMA8A	PNMA family member 8A	0.979	0.021	-0.115
672019106	ASPH	aspartate beta-hydroxylase	0.953	0.047	-0.114
60360272	KLHL5	kelch like family member 5	0.953	0.047	-0.113
148698492	RIMKLA	ribosomal modification protein rimK like family member A	0.998	0.002	-0.112
8393038	CAPN2	calpain 2	0.995	0.005	-0.111
148673922	HSPH1	heat shock protein family H (Hsp110) member 1	0.992	0.008	-0.110
157818589	TSPAN7	tetraspanin 7	0.996	0.004	-0.100
392353562	ATP8A2	ATPase phospholipid transporting 8A2	0.998	0.002	-0.099
157819175	Gpr165	G protein-coupled receptor 165	0.965	0.035	-0.099
25282395	GSTM3	glutathione S-transferase mu 3	0.970	0.030	-0.098
157822707	PCDH20	protocadherin 20	0.990	0.010	-0.097
401461792	GOT1	glutamic-oxaloacetic transaminase 1	0.952	0.048	-0.094
148703191	HSPA4L	heat shock protein family A (Hsp70) member 4 like	0.977	0.023	-0.093
54035294	ADH5	alcohol dehydrogenase 5 (class III), chi polypeptide	0.974	0.026	-0.080
148747375	CDS1	CDP-diacylglycerol synthase 1	0.970	0.030	-0.078
157818189	CIDEB	cell death inducing DFFA like effector b	0.965	0.035	-0.078
568986834	KCNMA1	potassium calcium-activated channel subfamily M alpha 1	0.999	0.001	-0.075
34536836	EHD3	EH domain containing 3	0.966	0.034	-0.071
11559994	PIK3R3	phosphoinositide-3-kinase regulatory subunit 3	0.960	0.040	-0.070
564374948	MRC2	mannose receptor C type 2	0.991	0.009	-0.070
62657153	EFTUD2	elongation factor Tu GTP binding domain containing 2	0.971	0.029	-0.068
188595689	STK24	serine/threonine kinase 24	0.970	0.030	-0.067
67846018	CTNNBL1	catenin beta like 1	0.997	0.003	-0.066
52138624	SLC25A20	solute carrier family 25 member 20	0.997	0.003	-0.066
9507099	ST6GALNAC3	ST6 N-acetylgalactosaminide alpha-2,6-sialyltransferase 3	0.990	0.010	-0.065
58865442	BTBD1	BTB domain containing 1	0.994	0.006	-0.060
28212260	SSX2IP	SSX family member 2 interacting protein	0.987	0.013	-0.057
198278575	BRCC3	BRCA1/BRCA2-containing complex subunit 3	0.983	0.017	-0.056
189027133	TTC30B	tetratricopeptide repeat domain 30B	0.968	0.032	-0.044

20809990	XPA	XPA, DNA damage recognition and repair factor	0.998	0.002	-0.043
281332197	Akr1c12/Akr1c13	aldo-keto reductase family 1, member C13	0.965	0.035	-0.037
62079109	LANCL2	LanC like 2	0.967	0.033	-0.034
149022924	SCG5	secretogranin V	-0.976	0.024	0.034
672066031	DGKD	diacylglycerol kinase delta	-0.990	0.010	0.044
157819577	SAP30BP	SAP30 binding protein	-0.989	0.011	0.045
77627906	KLHDC2	kelch domain containing 2	-0.961	0.039	0.045
45478098	CMTR1	cap methyltransferase 1	-0.992	0.008	0.046
81295337	SLC25A40	solute carrier family 25 member 40	-0.988	0.012	0.052
29825827	VPS26B	VPS26, retromer complex component B	-0.976	0.024	0.052
148690831	PRMT1	protein arginine methyltransferase 1	-0.985	0.015	0.054
33468857	HINT1	histidine triad nucleotide binding protein 1	-0.983	0.017	0.055
55250051	TXNRD1	thioredoxin reductase 1	-0.960	0.040	0.056
58865796	PTDSS1	phosphatidylserine synthase 1	-0.976	0.024	0.057
8393519	MACROH2A1	macroH2A.1 histone	-0.992	0.008	0.062
189011600	GPN1	GPN-loop GTPase 1	-0.991	0.009	0.063
25453374	PEX14	peroxisomal biogenesis factor 14	-0.972	0.028	0.067
25742568	DPYSL3	dihydropyrimidinase like 3	-0.977	0.023	0.069
149042824	UBE2V1	ubiquitin conjugating enzyme E2 V1	-0.993	0.007	0.070
8393746	MAP2K5	mitogen-activated protein kinase kinase 5	-0.996	0.004	0.072
112984152	PES1	pescadillo ribosomal biogenesis factor 1	-0.965	0.035	0.076
755492511	MAP4K4	mitogen-activated protein kinase kinase kinase kinase 4	-0.991	0.009	0.078
12083657	BAD	BCL2 associated agonist of cell death	-0.997	0.003	0.078
9507059	RNF5	ring finger protein 5	-0.985	0.015	0.080
37360414	NPLOC4	NPL4 homolog, ubiquitin recognition factor	-0.991	0.009	0.081
672074758	NCSTN	nicastrin	-0.966	0.034	0.083
564305145	NOL6	nucleolar protein 6	-0.964	0.036	0.086
26346731	UBE2Z	ubiquitin conjugating enzyme E2 Z	-0.987	0.013	0.091
62543509	DPH2	diphthamide biosynthesis 2	-0.971	0.029	0.093
564356795	ALKBH1	alkB homolog 1, histone H2A dioxygenase	-0.950	0.050	0.094
209447030	DDX27	DEAD-box helicase 27	-0.989	0.011	0.097
119618921	RAN	RAN, member RAS oncogene family	-0.966	0.034	0.098
157821483	BORCS5	BLOC-1 related complex subunit 5	-0.987	0.013	0.103
58865712	RRP1	ribosomal RNA processing 1	-0.972	0.028	0.104
149047559	MTMR3	myotubularin related protein 3	-0.966	0.034	0.106

42476292	TALDO1	transaldolase 1	-0.978	0.022	0.106
672054841	RCC2	regulator of chromosome condensation 2	-0.996	0.004	0.107
564382285	RPS6KC1	ribosomal protein S6 kinase C1	-1.000	0.000	0.107
19526763	CRCP	CGRP receptor component	-0.985	0.015	0.109
47847438	EXOC3	exocyst complex component 3	-0.963	0.037	0.118
672044181	HS2ST1	heparan sulfate 2-O-sulfotransferase 1	-0.968	0.032	0.124
148705576	CRMP1	collapsin response mediator protein 1	-0.951	0.049	0.125
164664442	PIAS1	protein inhibitor of activated STAT 1	-0.964	0.036	0.125
19705555	IPMK	inositol polyphosphate multikinase	-0.973	0.027	0.126
209977101	TRPM4	transient receptor potential cation channel subfamily M member 4	-0.988	0.012	0.127
325974480	NDUFA7	NADH:ubiquinone oxidoreductase subunit A7	-0.982	0.018	0.131
58865976	KLHDC3	kelch domain containing 3	-0.994	0.006	0.133
8394405	SLC7A5	solute carrier family 7 member 5	-0.972	0.028	0.134
13242322	ATF4	activating transcription factor 4	-0.957	0.043	0.135
451770387	PRRT2	proline rich transmembrane protein 2	-0.999	0.001	0.136
564398139	FYN	FYN proto-oncogene, Src family tyrosine kinase	-0.974	0.026	0.137
157818305	GPR101	G protein-coupled receptor 101	-0.965	0.035	0.140
672069253	SOCS7	suppressor of cytokine signaling 7	-0.955	0.045	0.141
20301952	SLC2A1	solute carrier family 2 member 1	-0.985	0.015	0.142
451172120	DUSP7	dual specificity phosphatase 7	-0.998	0.002	0.142
46485444	NOP53	NOP53 ribosome biogenesis factor	-0.995	0.005	0.144
68534951	CD40	CD40 molecule	-0.965	0.035	0.145
148665617	NAA50	N-alpha-acetyltransferase 50, NatE catalytic subunit	-0.952	0.048	0.149
68342005	HEXIM1	HEXIM P-TEFb complex subunit 1	-0.976	0.024	0.150
564311031	CLPP	caseinolytic mitochondrial matrix peptidase proteolytic subunit	-0.957	0.043	0.150
261337195	WDR91	WD repeat domain 91	-0.997	0.003	0.153
148679797	DEF8	differentially expressed in FDCP 8 homolog	-0.996	0.004	0.155
9910378	CDC42SE2	CDC42 small effector 2	-0.977	0.023	0.158
149038734	EIF4EBP2	eukaryotic translation initiation factor 4E binding protein 2	-0.972	0.028	0.158
76559935	TUT1	terminal uridylyl transferase 1, U6 snRNA-specific	-0.983	0.017	0.160
157819421	CEP97	centrosomal protein 97	-0.996	0.004	0.160
213688411	LPCAT1	lysophosphatidylcholine acyltransferase 1	-0.966	0.034	0.162
403225023	BRAP	BRCA1 associated protein	-0.950	0.050	0.166

408772026	Afg3l1	AFG3-like AAA ATPase 1	-0.967	0.033	0.166
55926133	RFC2	replication factor C subunit 2	-0.969	0.031	0.168
422398900	CREBZF	CREB/ATF bZIP transcription factor	-0.993	0.007	0.169
157823165	DNAJB1	DnaJ heat shock protein family (Hsp40) member B1	-0.969	0.031	0.171
157822067	BAP1	BRCA1 associated protein 1	-0.967	0.033	0.172
672040432	Mrpl43	mitochondrial ribosomal protein L43	-0.961	0.039	0.174
189491869	KCMF1	potassium channel modulatory factor 1	-0.973	0.027	0.176
119569672	BUB3	BUB3 mitotic checkpoint protein	-0.979	0.021	0.176
56090552	GTF2F1	general transcription factor IIF subunit 1	-0.956	0.044	0.177
61556927	EIF3G	eukaryotic translation initiation factor 3 subunit G	-0.963	0.037	0.180
6981518	SDC1	syndecan 1	-0.975	0.025	0.182
157823413	THOC3	THO complex 3	-0.972	0.028	0.183
11139303	JTB	jumping translocation breakpoint	-0.997	0.003	0.185
568983220	TENT4A	terminal nucleotidyltransferase 4A	-0.959	0.041	0.187
58652154	TRIM26	tripartite motif containing 26	-0.963	0.037	0.188
72004267	AKIRIN1	akirin 1	-0.993	0.007	0.192
140971918	Hnrnpab	heterogeneous nuclear ribonucleoprotein A/B	-0.996	0.004	0.194
81295375	SLC35B2	solute carrier family 35 member B2	-0.969	0.031	0.200
58865352	NUBP2	nucleotide binding protein 2	-0.998	0.002	0.200
77797839	UBXN1	UBX domain protein 1	-0.955	0.045	0.202
14277700	RPS12	ribosomal protein S12	-0.997	0.003	0.203
564384353	SH3BP2	SH3 domain binding protein 2	-0.987	0.013	0.204
258614012	PSMB8	proteasome 20S subunit beta 8	-0.981	0.019	0.206
61557082	TERF2IP	TERF2 interacting protein	-0.959	0.041	0.211
574584811	TUBB4A	tubulin beta 4A class IVa	-0.957	0.043	0.211
37359818	KCTD5	potassium channel tetramerization domain containing 5	-0.998	0.002	0.212
310616720	DHX37	DEAH-box helicase 37	-0.954	0.046	0.212
24638440	RIMS4	regulating synaptic membrane exocytosis 4	-0.982	0.018	0.220
405113035	E2F4	E2F transcription factor 4	-0.976	0.024	0.225
70794793	MAP2K7	mitogen-activated protein kinase kinase 7	-0.979	0.021	0.228
223555981	UBE2QL1	ubiquitin conjugating enzyme E2 Q family like 1	-0.974	0.026	0.231
58865962	RNF41	ring finger protein 41	-0.973	0.027	0.231
70794766	MRPS25	mitochondrial ribosomal protein S25	-0.975	0.025	0.235
19173746	STK17B	serine/threonine kinase 17b	-0.967	0.033	0.237
76096328	COMMD9	COMM domain containing 9	-0.999	0.001	0.238

71043628	OGFRL1	opioid growth factor receptor like 1	-0.994	0.006	0.245
57164019	B4GALT3	beta-1,4-galactosyltransferase 3	-0.976	0.024	0.256
40254721	AMIGO2	adhesion molecule with Ig like domain 2	-0.991	0.009	0.264
392350322	DNAJC13	DnaJ heat shock protein family (Hsp40) member C13	-0.980	0.020	0.266
18376839	DDIT4	DNA damage inducible transcript 4	-0.992	0.008	0.277
37360160	ATP11B	ATPase phospholipid transporting 11B (putative)	-0.999	0.001	0.278
402794103	ATG101	autophagy related 101	-0.988	0.012	0.279
148679437	HAS3	hyaluronan synthase 3	-0.994	0.006	0.284
564307173	HEATR5A	HEAT repeat containing 5A	-0.985	0.015	0.284
143359181	SLC66A2	solute carrier family 66 member 2	-0.968	0.032	0.288
403310686	SOX4	SRY-box transcription factor 4	-0.984	0.016	0.289
51948506	AK8	adenylate kinase 8	-0.981	0.019	0.290
672053062	FKBP15	FKBP prolyl isomerase 15	-0.965	0.035	0.290
201066401	RCOR3	REST corepressor 3	-0.990	0.010	0.294
34328151	TBR1	T-box brain transcription factor 1	-0.963	0.037	0.295
157822367	PUS3	pseudouridine synthase 3	-0.952	0.048	0.301
300798394	NPHP3	nephrocystin 3	-0.969	0.031	0.303
29789082	COIL	coilin	-0.969	0.031	0.305
157818733	ZBTB2	zinc finger and BTB domain containing 2	-0.957	0.043	0.306
157822893	IMP3	IMP U3 small nucleolar ribonucleoprotein 3	-0.990	0.010	0.307
300798436	NME6	NME/NM23 nucleoside diphosphate kinase 6	-0.962	0.038	0.308
349501022	2410002F23Rik	RIKEN cDNA 2410002F23 gene	-0.972	0.028	0.309
62078923	DZIP1L	DAZ interacting zinc finger protein 1 like	-0.985	0.015	0.315
564397086	BRPF3	bromodomain and PHD finger containing 3	-0.975	0.025	0.326
149032986	MFSD4B	major facilitator superfamily domain containing 4B	-0.981	0.019	0.332
564303955	EMX1	empty spiracles homeobox 1	-0.996	0.004	0.335
281604129	HELQ	helicase, POLQ like	-0.987	0.013	0.354
392342123	ALS2CL	ALS2 C-terminal like	-0.961	0.039	0.360
77627740	ING3	inhibitor of growth family member 3	-0.989	0.011	0.363
406362836	HS6ST3	heparan sulfate 6-O-sulfotransferase 3	-0.993	0.007	0.365
564311478	CRACDL	CRACD like	-0.968	0.032	0.369
564352668	MYCL	MYCL proto-oncogene, bHLH transcription factor	-0.996	0.004	0.392
672019438	FKBP15	FKBP prolyl isomerase 15	-0.990	0.010	0.395

16758238	SPA17	sperm autoantigenic protein 17	-0.992	0.008	0.402
23097354	FADD	Fas associated via death domain	-0.998	0.002	0.403
226371633	CABLES1	Cdk5 and Abl enzyme substrate 1	-0.961	0.039	0.409
293348214	CCDC88C	coiled-coil domain containing 88C	-0.980	0.020	0.421
564322442	Kdm6a	lysine demethylase 6A	-0.995	0.005	0.432
672031975	LOC299312	similar to G protein-binding protein CRFG	-0.995	0.005	0.436
893846521	MARCHF11	membrane associated ring-CH-type finger 11	-0.963	0.037	0.445
157820727	RPL27A	ribosomal protein L27a	-0.975	0.025	0.452
60360636	GAREM1	GRB2 associated regulator of MAPK1 subtype 1	-0.986	0.014	0.454
564347830	ZXDC	ZXD family zinc finger C	-0.965	0.035	0.490
212549645	KIF18A	kinesin family member 18A	-0.975	0.025	0.505
240255436	T2	brachyury 2	-0.965	0.035	0.515
148705473	FAM53A	family with sequence similarity 53 member A	-0.972	0.028	0.585
16758872	CDH17	cadherin 17	-0.965	0.035	0.585
12621078	PTPRQ	protein tyrosine phosphatase receptor type Q	-0.965	0.035	0.585
38016150	QRFPR	pyroglutamylated RFamide peptide receptor	-0.991	0.009	0.625
148689488	SYN3	synapsin III	-0.972	0.028	0.627
256220048	PCDHGC5	protocadherin gamma subfamily C, 5	-0.989	0.011	0.666
149016574	ZNF324	zinc finger protein 324	-0.974	0.026	0.689
112984482	SBSN	suprabasin	-0.971	0.029	0.700
61557100	PLEKHF1	pleckstrin homology and FYVE domain containing 1	-0.989	0.011	0.707
84781680	CNKSR1	connector enhancer of kinase suppressor of Ras 1	-0.965	0.035	0.737
20302047	AMPD1	adenosine monophosphate deaminase 1	-0.965	0.035	0.737
672059431	SLC39A4	solute carrier family 39 member 4	-0.965	0.035	0.737
282397098	PRR30	proline rich 30	-0.965	0.035	0.778
453178	AMHR2	anti-Mullerian hormone receptor type 2	-0.965	0.035	0.778
6978747	Cyp2d26	cytochrome P450, family 2, subfamily d, polypeptide 26	-0.965	0.035	0.807
157818453	CXorf21	chromosome X open reading frame 21	-0.965	0.035	0.830
157822171	C1orf210	chromosome 1 open reading frame 210	-0.965	0.035	0.874
16758266	NME3	NME/NM23 nucleoside diphosphate kinase 3	-0.971	0.029	0.921

58865986	RNASE12	ribonuclease A family member 12 (inactive)	-0.965	0.035	0.954
157823859	METTL27	methyltransferase like 27	-0.964	0.036	0.958
149030324	CHRNA2	cholinergic receptor nicotinic alpha 2 subunit	-0.996	0.004	0.994
157817241	ISCA2	iron-sulfur cluster assembly 2	-0.998	0.002	1.006
300796953	SYCE2	synaptonemal complex central element protein 2	-0.993	0.007	1.163
6978525	FASLG	Fas ligand	-0.972	0.028	1.186
300793935	GSX1	GS homeobox 1	-0.985	0.015	1.225
672068318	PITPNM3	PITPNM family member 3	-0.966	0.034	1.283
56605846	DPEP3	dipeptidase 3	-0.958	0.042	1.348
124487463	GPR161	G protein-coupled receptor 161	-0.990	0.010	1.523
568914628	GARNL3	GTPase activating Rap/RanGAP domain like 3	-0.968	0.032	1.578
575403049	ERBIN	erbb2 interacting protein	-0.991	0.009	1.596
157816947	GUCA1B	guanylate cyclase activator 1B	-0.968	0.032	1.708
568979594	SYT16	synaptotagmin 16	-0.953	0.047	2.059

Supplementary Table S9. The list of genes that are differentially expressed in the offspring hippocampus in response to prenatal BPA exposure that exhibited the changes in the expression levels correlated with the cell density in CA1 of the hippocampus. The transcriptome profiling data of DEGs in male and female rat offspring prenatally exposed to BPA (n = 6, male pups n = 3 and female pups n = 3, from independent litters) or the vehicle control (n = 6, male pups n = 3 and female pups n = 3, from independent litters) were obtained and used for the PTM analyses to identify DEGs that exhibited the changes in the expression levels correlated with the cell density in CA1 of the hippocampus.

ID	Symbol	Entrez Gene Name	log2(FC)	R values	P-values
293347435	PTPRD	protein tyrosine phosphatase receptor type D	-7.731	0.970	0.030
564375502	Mxra7	matrix-remodelling associated 7	-7.209	0.970	0.030
392339806	CFAP69	cilia and flagella associated protein 69	-6.820	0.954	0.046
564314389	DZIP3	DAZ interacting zinc finger protein 3	-6.700	0.984	0.016
149020633	TAF1D	TATA-box binding protein associated factor, RNA polymerase I subunit D	-6.476	0.985	0.015
157818475	SMIM22	small integral membrane protein 22	-5.615	0.979	0.021
567315993	LOC102550396	LRRGT00188	-5.600	0.973	0.027
564312230	LOC100912948	multidrug resistance-associated protein 1-like	-5.285	0.970	0.030
197384778	Snorc	secondary ossification center associated regulator of chondrocyte maturation	-5.044	0.977	0.023
672035060	CIC	capicua transcriptional repressor	-4.863	0.959	0.041
564323305	LOC681300	similar to CXXC finger 5	-4.672	0.985	0.015
209447125	Ctf2	cardiotrophin 2	-4.392	0.970	0.030
201860265	NRN1L	neuritin 1 like	-4.358	0.976	0.024
300798104	IFNLR1	interferon lambda receptor 1	-4.248	0.977	0.023
51591901	MPIG6B	megakaryocyte and platelet inhibitory receptor G6b	-4.170	0.970	0.030
564298047	GDPD5	glycerophosphodiester phosphodiesterase domain containing 5	-4.163	0.969	0.031
300798035	NRG4	neuregulin 4	-4.044	0.977	0.023
61556838	Raet1d/Raet1e	retinoic acid early transcript 1E	-4.044	0.977	0.023
6978515	APOA1	apolipoprotein A1	-3.807	0.970	0.030
117647210	CTRC	chymotrypsin C	-3.807	0.970	0.030
8392926	ASGR2	asialoglycoprotein receptor 2	-3.700	0.951	0.049
48040447	SUCNR1	succinate receptor 1	-3.700	0.970	0.030
194473646	UPK3A	uroplakin 3A	-3.585	0.970	0.030
164518908	RAB25	RAB25, member RAS oncogene family	-3.459	0.970	0.030

285026465	HS3ST3A1	heparan sulfate-glucosamine 3-sulfotransferase 3A1	-3.322	0.970	0.030
148710035	PITX3	paired like homeodomain 3	-3.322	0.970	0.030
56788780	KRT19	keratin 19	-3.248	0.954	0.046
9506451	CA5A	carbonic anhydrase 5A	-3.248	0.981	0.019
56090299	ODF4	outer dense fiber of sperm tails 4	-3.170	0.988	0.012
157822159	CCDC42	coiled-coil domain containing 42	-3.170	0.970	0.030
123173794	GSG1	germ cell associated 1	-3.170	0.970	0.030
77917534	CBLC	Cbl proto-oncogene C	-2.807	0.970	0.030
157822121	LRMDA	leucine rich melanocyte differentiation associated	-2.747	0.997	0.003
51948496	SLC22A18	solute carrier family 22 member 18	-2.687	0.985	0.015
672032217	REPS2	RALBP1 associated Eps domain containing 2	-2.683	0.996	0.004
298566276	Ces1a	carboxylesterase 1A	-2.585	0.970	0.030
57222328	PFN4	profilin family member 4	-2.585	0.970	0.030
157786772	KREMEN2	kringle containing transmembrane protein 2	-2.585	0.970	0.030
306482632	BPIFB4	BPI fold containing family B member 4	-2.585	0.970	0.030
57222300	Klra2	killer cell lectin-like receptor, subfamily A, member 2	-2.585	0.970	0.030
157822463	Nkx6-3	NK6 homeobox 3	-2.585	0.970	0.030
564391680	MAP3K8	mitogen-activated protein kinase kinase kinase 8	-2.426	0.976	0.024
62945342	LAX1	lymphocyte transmembrane adaptor 1	-2.322	0.970	0.030
187469451	CLEC7A	C-type lectin domain containing 7A	-2.322	0.970	0.030
157820151	ERAS	ES cell expressed Ras	-2.248	0.981	0.019
564320724	Fbxo38	F-box protein 38	-2.220	0.970	0.030
66730349	SPIB	Spi-B transcription factor	-2.170	0.985	0.015
300797305	TMEM45A	transmembrane protein 45A	-2.093	0.987	0.013
148672128	SMAGP	small cell adhesion glycoprotein	-2.077	0.984	0.016
55741882	ZPBP2	zona pellucida binding protein 2	-2.022	0.975	0.025
157822587	PDE6B	phosphodiesterase 6B	-2.000	0.970	0.030
157818091	TMEM182	transmembrane protein 182	-2.000	0.970	0.030
296483047	SIX1	SIX homeobox 1	-2.000	0.970	0.030
117647206	DDX4	DEAD-box helicase 4	-2.000	0.970	0.030
19424304	CHRNB3	cholinergic receptor nicotinic beta 3 subunit	-2.000	0.970	0.030
47577151	Olfr1441	olfactory receptor 1441	-1.972	0.970	0.030
56676350	PRSS35	serine protease 35	-1.962	0.995	0.005
149066014	PVALB	parvalbumin	-1.939	0.954	0.046
157820217	Gsta4	glutathione S-transferase, alpha 4	-1.930	0.958	0.042
117647214	EDN3	endothelin 3	-1.913	0.984	0.016

81295367	Abcg3	ATP binding cassette subfamily G member 3	-1.898	0.995	0.005
672029702	TUT7	terminal uridylyl transferase 7	-1.854	0.966	0.034
13592031	PTGER2	prostaglandin E receptor 2	-1.845	1.000	0.000
297374767	TPSAB1/TPSB2	tryptase alpha/beta 1	-1.834	0.958	0.042
300796107	PROX2	prospero homeobox 2	-1.700	0.951	0.049
9910234	IFIT1B	interferon induced protein with tetratricopeptide repeats 1B	-1.678	0.970	0.030
31542125	ALOX15	arachidonate 15-lipoxygenase	-1.611	0.967	0.033
58331159	GSTA3	glutathione S-transferase alpha 3	-1.603	0.998	0.002
76443687	SLC4A1	solute carrier family 4 member 1 (Diego blood group)	-1.599	0.968	0.032
157821527	RHOD	ras homolog family member D	-1.597	0.999	0.001
209870037	INSRR	insulin receptor related receptor	-1.585	0.972	0.028
157819393	NNMT	nicotinamide N-methyltransferase	-1.585	0.996	0.004
157818603	CLCA2	chloride channel accessory 2	-1.585	0.970	0.030
685156911	NLRP4	NLR family pyrin domain containing 4	-1.585	0.970	0.030
149034165	GALNT15	polypeptide N-acetylgalactosaminyltransferase 15	-1.561	0.968	0.032
13929126	GALNT5	polypeptide N-acetylgalactosaminyltransferase 5	-1.478	0.965	0.035
71043750	SYNGR4	synaptogyrin 4	-1.464	0.976	0.024
81295349	SLC52A3	solute carrier family 52 member 3	-1.453	0.985	0.015
283806636	ZNF831	zinc finger protein 831	-1.433	0.983	0.017
31377521	S1PR5	sphingosine-1-phosphate receptor 5	-1.426	0.984	0.016
77917586	GRAP2	GRB2 related adaptor protein 2	-1.412	0.983	0.017
157822105	SLC49A3	solute carrier family 49 member 3	-1.389	0.999	0.001
672025117	MBTD1	mbt domain containing 1	-1.344	0.985	0.015
6978717	CTRB2	chymotrypsinogen B2	-1.342	0.989	0.011
29789044	SNAI2	snail family transcriptional repressor 2	-1.328	0.985	0.015
157823345	LRR1	leucine rich repeat protein 1	-1.322	0.951	0.049
307746876	Pzp	PZP, alpha-2-macroglobulin like	-1.322	0.970	0.030
148694035	SENP8	SUMO peptidase family member, NEDD8 specific	-1.276	0.974	0.026
62656582	KIAA0100	KIAA0100	-1.266	0.996	0.004
16758338	FTCD	formimidoyltransferase cyclodeaminase	-1.208	0.961	0.039
56119141	BTK	Bruton tyrosine kinase	-1.205	0.966	0.034
564300462	DCHS2	dachsous cadherin-related 2	-1.190	0.967	0.033
392334475	Myb	MYB proto-oncogene, transcription factor	-1.175	0.987	0.013
281332082	THBS2	thrombospondin 2	-1.164	0.991	0.009
157823801	SLC50A1	solute carrier family 50 member 1	-1.136	0.990	0.010

60223053	SEPTIN1	septin 1	-1.133	0.995	0.005
157819247	CPA4	carboxypeptidase A4	-1.087	0.970	0.030
564310671	NBEAL2	neurobeachin like 2	-1.084	0.956	0.044
157818961	UBA7	ubiquitin like modifier activating enzyme 7	-1.078	0.955	0.045
197386139	SSC5D	scavenger receptor cysteine rich family member with 5 domains	-1.060	0.954	0.046
157821423	TBX6	T-box transcription factor 6	-1.017	0.962	0.038
300797728	MGST3	microsomal glutathione S-transferase 3	-1.011	0.974	0.026
392342053	GK5	glycerol kinase 5	-1.000	0.951	0.049
156231008	PRND	prion like protein doppel	-1.000	0.970	0.030
25453414	ASS1	argininosuccinate synthase 1	-1.000	0.989	0.011
59676595	FAM20A	FAM20A golgi associated secretory pathway pseudokinase	-1.000	0.977	0.023
765826426	Acot6	acyl-CoA thioesterase 6	-1.000	0.982	0.018
569009290	TENM1	teneurin transmembrane protein 1	-0.985	0.992	0.008
407228396	THEMIS2	thymocyte selection associated family member 2	-0.980	0.976	0.024
157817157	FAM166C	family with sequence similarity 166 member C	-0.979	0.977	0.023
215276950	PKP2	plakophilin 2	-0.973	0.989	0.011
672033256	LOC100912904	disks large homolog 5-like	-0.960	0.968	0.032
157820951	PRSS53	serine protease 53	-0.959	0.958	0.042
16758344	SEC16B	SEC16 homolog B, endoplasmic reticulum export factor	-0.958	0.954	0.046
157818989	LRRC71	leucine rich repeat containing 71	-0.952	0.992	0.008
157818275	KCNG4	potassium voltage-gated channel modifier subfamily G member 4	-0.952	0.971	0.029
198442873	CDC14A	cell division cycle 14A	-0.936	0.996	0.004
62945330	SLC8B1	solute carrier family 8 member B1	-0.929	0.996	0.004
50657416	C1RL	complement C1r subcomponent like	-0.918	0.970	0.030
157819487	TACO1	translational activator of cytochrome c oxidase I	-0.917	0.966	0.034
47059114	LTB	lymphotoxin beta	-0.916	0.980	0.020
564325648	Zfp54	zinc finger protein 54	-0.907	0.987	0.013
564318923	WDR17	WD repeat domain 17	-0.904	0.994	0.006
209870105	GPR37L1	G protein-coupled receptor 37 like 1	-0.870	0.992	0.008
57114338	SCN4B	sodium voltage-gated channel beta subunit 4	-0.859	1.000	0.000
149031998	ACVRL1	activin A receptor like type 1	-0.852	0.994	0.006
157822457	SYNC	syncoilin, intermediate filament protein	-0.833	0.951	0.049
564373460	SLFN13	schlafen family member 13	-0.831	0.982	0.018

56605720	GADD45B	growth arrest and DNA damage inducible beta	-0.828	0.996	0.004
6980992	GSTT2/GSTT2B	glutathione S-transferase theta 2 (gene/pseudogene)	-0.824	0.951	0.049
58865784	GPR157	G protein-coupled receptor 157	-0.812	0.991	0.009
157817065	KCNK16	potassium two pore domain channel subfamily K member 16	-0.807	0.951	0.049
259089426	AGER	advanced glycosylation end-product specific receptor	-0.804	0.998	0.002
300798165	ZBTB40	zinc finger and BTB domain containing 40	-0.788	0.951	0.049
18426812	ADA	adenosine deaminase	-0.787	0.977	0.023
307548437	NYAP2	neuronal tyrosine-phosphorylated phosphoinositide-3-kinase adaptor 2	-0.780	0.999	0.001
942523340	CAPRIN2	caprin family member 2	-0.774	0.998	0.002
157819205	EFHC2	EF-hand domain containing 2	-0.772	0.986	0.014
61097937	VEGFB	vascular endothelial growth factor B	-0.766	0.967	0.033
56090397	CYB5D2	cytochrome b5 domain containing 2	-0.764	0.962	0.038
27465577	Cyp4f16/Cyp4f37	cytochrome P450, family 4, subfamily f, polypeptide 16	-0.758	0.990	0.010
13591914	ANPEP	alanyl aminopeptidase, membrane	-0.757	0.975	0.025
672069802	C1QTNF1	C1q and TNF related 1	-0.749	0.999	0.001
157786850	TUBD1	tubulin delta 1	-0.740	0.966	0.034
56119147	ARRDC3	arrestin domain containing 3	-0.732	0.963	0.037
16758232	PLCB2	phospholipase C beta 2	-0.721	0.953	0.047
19173756	ERG	ETS transcription factor ERG	-0.718	0.977	0.023
149047075	Spaca6	sperm acrosome associated 6	-0.715	0.984	0.016
564317714	Ktn1	kinectin 1	-0.687	0.963	0.037
74142284	DSE	dermatan sulfate epimerase	-0.667	0.972	0.028
157818909	Zim1	zinc finger, imprinted 1	-0.665	0.962	0.038
672057459	DGKA	diacylglycerol kinase alpha	-0.664	0.968	0.032
157824216	RRAS	RAS related	-0.655	0.975	0.025
9845261	LGALS1	galectin 1	-0.655	0.967	0.033
157823283	Coch	cochlin	-0.653	0.993	0.007
77020281	CD55	CD55 molecule (Cromer blood group)	-0.648	0.962	0.038
672039742	TKFC	triokinase and FMN cyclase	-0.646	0.957	0.043
68534736	ERAP1	endoplasmic reticulum aminopeptidase 1	-0.646	0.967	0.033
13786136	PDGFC	platelet derived growth factor C	-0.642	0.974	0.026
293340128	MIEF2	mitochondrial elongation factor 2	-0.642	0.958	0.042
149061352	ADAM12	ADAM metallopeptidase domain 12	-0.638	0.976	0.024
34734058	HCK	HCK proto-oncogene, Src family tyrosine kinase	-0.631	0.985	0.015
397529557	C8orf58	chromosome 8 open reading frame 58	-0.629	0.975	0.025

157824208	NTNG1	netrin G1	-0.628	0.989	0.011
16758622	IFT172	intraflagellar transport 172	-0.627	0.983	0.017
564395215	LOC100909409 (includes others)	RGD1562660	-0.626	0.980	0.020
157786914	OGFOD2	2-oxoglutarate and iron dependent oxygenase domain containing 2	-0.618	0.951	0.049
402478640	HTRA3	HtrA serine peptidase 3	-0.608	0.994	0.006
157819065	ADAMTS15	ADAM metallopeptidase with thrombospondin type 1 motif 15	-0.604	0.974	0.026
157818491	DUS2	dihydrouridine synthase 2	-0.602	0.978	0.022
27465529	SLC9A4	solute carrier family 9 member A4	-0.601	0.988	0.012
56605940	RXFP3	relaxin family peptide receptor 3	-0.599	0.991	0.009
293347888	SRBD1	S1 RNA binding domain 1	-0.598	0.984	0.016
567316103	Ac1576	uncharacterized LOC102552783	-0.587	0.987	0.013
197927123	LYRM7	LYR motif containing 7	-0.587	0.994	0.006
300793858	PARP14	poly(ADP-ribose) polymerase family member 14	-0.585	0.992	0.008
564394999	CLGN	calmegin	-0.582	0.989	0.011
564300485	LOC102551095	uncharacterized LOC102551095	-0.576	0.982	0.018
16758560	WIF1	WNT inhibitory factor 1	-0.573	0.991	0.009
564366772	MGC116197 (includes others)	similar to RIKEN cDNA 1700001E04	-0.569	0.984	0.016
55741859	XRCC4	X-ray repair cross complementing 4	-0.569	0.982	0.018
157816941	PLXDC1	plexin domain containing 1	-0.567	0.994	0.006
62078799	QRSL1	glutaminyl-tRNA amidotransferase subunit QRSL1	-0.566	0.997	0.003
57012436	Krt10	keratin 10	-0.562	0.969	0.031
75832150	GALNT3	polypeptide N-acetylgalactosaminyltransferase 3	-0.557	0.996	0.004
213385268	Gm10778	predicted gene 10778	-0.554	0.979	0.021
9507045	RGS5	regulator of G protein signaling 5	-0.538	0.972	0.028
148747464	SCD	stearoyl-CoA desaturase	-0.524	0.984	0.016
19424232	CSF2RB	colony stimulating factor 2 receptor subunit beta	-0.524	0.974	0.026
6981180	MAOB	monoamine oxidase B	-0.518	0.991	0.009
148692356	ARHGEF1	Rho guanine nucleotide exchange factor 1	-0.517	0.985	0.015
149037033	PRDM5	PR/SET domain 5	-0.516	0.992	0.008
149047863	LOC690190	hypothetical protein LOC690190	-0.514	0.971	0.029
147900684	TLR7	toll like receptor 7	-0.511	0.981	0.019
62078773	CCDC81	coiled-coil domain containing 81	-0.511	0.995	0.005
654824082	Fbxl21	F-box and leucine-rich repeat protein 21	-0.500	0.986	0.014
50233928	TMEM159	transmembrane protein 159	-0.497	0.970	0.030

157822759	PARP2	poly(ADP-ribose) polymerase 2	-0.493	0.958	0.042
399220341	SLC2A13	solute carrier family 2 member 13	-0.492	0.972	0.028
157819347	CDC6	cell division cycle 6	-0.492	0.984	0.016
12738847	MERTK	MER proto-oncogene, tyrosine kinase	-0.488	0.960	0.040
395759219	AQP4	aquaporin 4	-0.482	0.999	0.001
189011606	NCEH1	neutral cholesterol ester hydrolase 1	-0.476	0.994	0.006
41056215	XRCC5	X-ray repair cross complementing 5	-0.466	0.992	0.008
148356229	CCND1	cyclin D1	-0.464	0.963	0.037
56090632	DMAC2L	distal membrane arm assembly complex 2 like	-0.463	0.962	0.038
148669431	DNAJC27	DnaJ heat shock protein family (Hsp40) member C27	-0.459	0.953	0.047
68163403	SLC46A3	solute carrier family 46 member 3	-0.458	0.967	0.033
219275548	DUSP19	dual specificity phosphatase 19	-0.458	0.957	0.043
56090411	POLE3	DNA polymerase epsilon 3, accessory subunit	-0.456	0.990	0.010
157817743	CDH5	cadherin 5	-0.454	0.985	0.015
9437326	SLC4A4	solute carrier family 4 member 4	-0.453	0.960	0.040
158138494	PTPRC	protein tyrosine phosphatase receptor type C	-0.451	0.955	0.045
33414515	PXK	PX domain containing serine/threonine kinase like	-0.450	0.972	0.028
58865380	STAT2	signal transducer and activator of transcription 2	-0.450	0.973	0.027
392331978	CDR2L	cerebellar degeneration related protein 2 like	-0.442	0.985	0.015
149022245	SCRN3	secernin 3	-0.437	0.951	0.049
68534547	NUDT18	nudix hydrolase 18	-0.437	0.984	0.016
148686921	SLC24A4	solute carrier family 24 member 4	-0.435	0.962	0.038
404312655	SDR42E1	short chain dehydrogenase/reductase family 42E, member 1	-0.435	0.956	0.044
61889119	TNFSF12	TNF superfamily member 12	-0.432	0.997	0.003
210031518	MOGAT2	monoacylglycerol O-acyltransferase 2	-0.432	0.950	0.050
157823259	TMEM229A	transmembrane protein 229A	-0.424	0.998	0.002
300796997	ARHGAP28	Rho GTPase activating protein 28	-0.423	0.967	0.033
210032365	HSP90B1	heat shock protein 90 beta family member 1	-0.421	0.982	0.018
56090564	GALM	galactose mutarotase	-0.420	0.980	0.020
62945312	CXCL16	C-X-C motif chemokine ligand 16	-0.414	0.980	0.020
194473652	TTC38	tetratricopeptide repeat domain 38	-0.413	0.964	0.036
9506405	ARPC1B	actin related protein 2/3 complex subunit 1B	-0.410	0.980	0.020
13591949	GATM	glycine amidinotransferase	-0.409	0.991	0.009

672061813	ACSBG1	acyl-CoA synthetase bubblegum family member 1	-0.409	0.958	0.042
13162347	FDXR	ferredoxin reductase	-0.405	0.997	0.003
195973006	EGFLAM	EGF like, fibronectin type III and laminin G domains	-0.403	0.951	0.049
148666792	ARHGAP25	Rho GTPase activating protein 25	-0.396	0.983	0.017
78187977	TCF19	transcription factor 19	-0.393	0.964	0.036
157820327	THSD1	thrombospondin type 1 domain containing 1	-0.391	0.983	0.017
187937124	TMEM126B	transmembrane protein 126B	-0.389	0.969	0.031
149034469	GNG7	G protein subunit gamma 7	-0.381	0.992	0.008
149036529	DGUOK	deoxyguanosine kinase	-0.381	0.982	0.018
929981595	NPHP1	nephrocystin 1	-0.372	0.998	0.002
157824002	ATG10	autophagy related 10	-0.372	0.956	0.044
564299653	FAM169A	family with sequence similarity 169 member A	-0.372	0.999	0.001
198386343	TRPS1	transcriptional repressor GATA binding 1	-0.370	0.999	0.001
8393861	HPCAL4	hippocalcin like 4	-0.370	0.983	0.017
158303308	PCCA	propionyl-CoA carboxylase subunit alpha	-0.369	0.959	0.041
564343748	CDK5RAP1	CDK5 regulatory subunit associated protein 1	-0.368	0.996	0.004
564357619	ITGB8	integrin subunit beta 8	-0.366	0.992	0.008
16758712	PDIA4	protein disulfide isomerase family A member 4	-0.362	0.989	0.011
55741549	MRPL13	mitochondrial ribosomal protein L13	-0.360	0.993	0.007
40786491	CYP20A1	cytochrome P450 family 20 subfamily A member 1	-0.358	0.953	0.047
149066868	MDM1	Mdm1 nuclear protein	-0.357	0.998	0.002
157822187	WWOX	WW domain containing oxidoreductase	-0.355	0.972	0.028
672052705	FRRS1L	ferric chelate reductase 1 like	-0.354	0.959	0.041
11693172	CALR	calreticulin	-0.354	0.987	0.013
564331077	HIRIP3	HIRA interacting protein 3	-0.352	0.981	0.019
56605656	DONSON	DNA replication fork stabilization factor DONSON	-0.350	0.966	0.034
451172073	CHRM3	cholinergic receptor muscarinic 3	-0.349	0.986	0.014
62078847	TSEN2	tRNA splicing endonuclease subunit 2	-0.343	0.972	0.028
77157795	MAL2	mal, T cell differentiation protein 2 (gene/pseudogene)	-0.342	0.999	0.001
157820807	GCDH	glutaryl-CoA dehydrogenase	-0.339	0.959	0.041
11693162	INSIG1	insulin induced gene 1	-0.339	0.984	0.016
56605710	LTBR	lymphotoxin beta receptor	-0.338	0.960	0.040

300795679	CD84	CD84 molecule	-0.338	0.968	0.032
13786174	TIMELESS	timeless circadian regulator	-0.332	0.982	0.018
300794275	MFSD10	major facilitator superfamily domain containing 10	-0.330	0.970	0.030
564309649	CCDC159	coiled-coil domain containing 159	-0.329	0.969	0.031
281604125	Fam50a/LOC100910130	family with sequence similarity 50, member A	-0.327	0.978	0.022
149045696	Ccl27a	chemokine (C-C motif) ligand 27A	-0.325	0.960	0.040
62078809	TNFAIP8L2	TNF alpha induced protein 8 like 2	-0.324	0.958	0.042
51036680	SLC29A3	solute carrier family 29 member 3	-0.322	0.959	0.041
451172111	HINT3	histidine triad nucleotide binding protein 3	-0.321	0.994	0.006
40018538	ADI1	acireductone dioxygenase 1	-0.316	0.954	0.046
149041432	THY1	Thy-1 cell surface antigen	-0.313	0.977	0.023
157786608	MRPL55	mitochondrial ribosomal protein L55	-0.313	0.954	0.046
564336403	EXOSC8	exosome component 8	-0.309	0.982	0.018
62078551	GNB4	G protein subunit beta 4	-0.309	0.956	0.044
149063353	IFT81	intraflagellar transport 81	-0.309	0.995	0.005
167860097	FN3KRP	fructosamine 3 kinase related protein	-0.308	0.959	0.041
61556910	SNX10	sorting nexin 10	-0.306	0.989	0.011
31982028	RSU1	Ras suppressor protein 1	-0.304	0.987	0.013
300796069	THADA	THADA armadillo repeat containing	-0.303	0.978	0.022
56090361	EPDR1	ependymin related 1	-0.303	0.978	0.022
157820737	NUSAP1	nucleolar and spindle associated protein 1	-0.298	0.952	0.048
38259192	TOP2A	DNA topoisomerase II alpha	-0.297	0.989	0.011
6978888	GFRA1	GDNF family receptor alpha 1	-0.294	0.959	0.041
672035779	Proser3	proline and serine rich 3	-0.293	0.971	0.029
564358911	CHPT1	choline phosphotransferase 1	-0.292	0.974	0.026
157817979	Egfem1	EGF-like and EMI domain containing 1	-0.291	0.985	0.015
157823996	ELK3	ETS transcription factor ELK3	-0.282	0.954	0.046
197313643	GLTP	glycolipid transfer protein	-0.280	0.973	0.027
60097941	HP	haptoglobin	-0.279	0.954	0.046
142349612	GLUL	glutamate-ammonia ligase	-0.279	0.976	0.024
564364473	RNF111	ring finger protein 111	-0.276	0.967	0.033
62079057	IL33	interleukin 33	-0.273	0.955	0.045
197333840	CAMKMT	calmodulin-lysine N-methyltransferase	-0.270	0.958	0.042
149022319	AGPS	alkylglycerone phosphate synthase	-0.270	0.966	0.034
149068830	SLCO2B1	solute carrier organic anion transporter family member 2B1	-0.270	0.962	0.038
148701892	EBF1	EBF transcription factor 1	-0.270	0.966	0.034

57164113	NSDHL	NAD(P) dependent steroid dehydrogenase-like	-0.269	0.971	0.029
576796148	MAP7D2	MAP7 domain containing 2	-0.269	0.982	0.018
74354506	ACBD5	acyl-CoA binding domain containing 5	-0.269	0.980	0.020
197313676	AIG1	androgen induced 1	-0.268	0.980	0.020
38181552	SCG2	secretogranin II	-0.267	0.951	0.049
157819753	RCN1	reticulocalbin 1	-0.266	0.971	0.029
564382316	HSD11B1	hydroxysteroid 11-beta dehydrogenase 1	-0.265	0.971	0.029
157817017	MRPS16	mitochondrial ribosomal protein S16	-0.261	0.956	0.044
564382292	ANGEL2	angel homolog 2	-0.258	0.983	0.017
17865325	GLRB	glycine receptor beta	-0.256	0.998	0.002
148700512	NRSN1	neurensin 1	-0.256	0.955	0.045
149046389	ARID5A	AT-rich interaction domain 5A	-0.249	0.970	0.030
157819311	LRGUK	leucine rich repeats and guanylate kinase domain containing	-0.248	0.955	0.045
157819077	TRIM37	tripartite motif containing 37	-0.246	0.993	0.007
15805026	ZFAND6	zinc finger AN1-type containing 6	-0.245	0.989	0.011
6978631	CD4	CD4 molecule	-0.245	0.988	0.012
187937143	C2orf42	chromosome 2 open reading frame 42	-0.241	0.965	0.035
57527332	PSPH	phosphoserine phosphatase	-0.241	0.968	0.032
58865958	RDH11	retinol dehydrogenase 11	-0.240	0.990	0.010
57192	P3H4	prolyl 3-hydroxylase family member 4 (inactive)	-0.240	0.968	0.032
672047003	CDAN1	codanin 1	-0.236	0.965	0.035
157817480	RWDD2A	RWD domain containing 2A	-0.235	0.976	0.024
77404265	JAM2	junctional adhesion molecule 2	-0.233	0.988	0.012
584277046	SLC1A3	solute carrier family 1 member 3	-0.232	0.985	0.015
293346766	TCAF1	TRPM8 channel associated factor 1	-0.232	0.979	0.021
9457244	RBBP9	RB binding protein 9, serine hydrolase	-0.231	0.980	0.020
13489067	NSF	N-ethylmaleimide sensitive factor, vesicle fusing ATPase	-0.231	0.956	0.044
157817839	SEMA5A	semaphorin 5A	-0.230	0.965	0.035
564397761	GCC2	GRIP and coiled-coil domain containing 2	-0.229	0.995	0.005
58865718	HERC4	HECT and RLD domain containing E3 ubiquitin protein ligase 4	-0.228	0.977	0.023
672050038	NDNF	neuron derived neurotrophic factor	-0.226	0.974	0.026
205277356	TVP23B	trans-golgi network vesicle protein 23 homolog B	-0.226	0.951	0.049
157817710	FER	FER tyrosine kinase	-0.225	0.988	0.012
293345175	DHX29	DExH-box helicase 29	-0.225	1.000	0.000

564383995	EVC	EvC ciliary complex subunit 1	-0.225	0.961	0.039
25742576	NXF1	nuclear RNA export factor 1	-0.224	0.966	0.034
78369663	SLC38A9	solute carrier family 38 member 9	-0.222	0.960	0.040
148689145	CPNE4	copine 4	-0.222	0.997	0.003
148695758	CAPRIN1	cell cycle associated protein 1	-0.221	0.982	0.018
564298436	WDR11	WD repeat domain 11	-0.216	0.958	0.042
148687519	CALN1	calneuron 1	-0.214	0.980	0.020
8393643	KCNAB1	potassium voltage-gated channel subfamily A member regulatory beta subunit 1	-0.214	0.988	0.012
205755	TAGLN3	transgelin 3	-0.213	0.954	0.046
564398053	MAN1A1	mannosidase alpha class 1A member 1	-0.213	0.951	0.049
57526927	LARS1	leucyl-tRNA synthetase 1	-0.213	0.997	0.003
392338550	IPO11	importin 11	-0.212	0.982	0.018
415703079	NEBL	nebulette	-0.211	0.960	0.040
18959250	PRKCD	protein kinase C delta	-0.210	0.955	0.045
149058126	ALDH9A1	aldehyde dehydrogenase 9 family member A1	-0.208	0.985	0.015
148667192	LRTM2	leucine rich repeats and transmembrane domains 2	-0.207	0.975	0.025
148747414	GDA	guanine deaminase	-0.203	0.994	0.006
564391231	SERPINB9	serpin family B member 9	-0.203	0.989	0.011
55741502	ACAT2	acetyl-CoA acetyltransferase 2	-0.202	0.999	0.001
48675867	PLPP3	phospholipid phosphatase 3	-0.201	0.981	0.019
68163417	FAHD1	fumarylacetoacetate hydrolase domain containing 1	-0.201	0.951	0.049
11560055	KHDRBS3	KH RNA binding domain containing, signal transduction associated 3	-0.195	0.996	0.004
564334053	SORCS1	sortilin related VPS10 domain containing receptor 1	-0.195	1.000	0.000
62078931	PAQR8	progestin and adipoQ receptor family member 8	-0.192	0.953	0.047
17530977	ECHS1	enoyl-CoA hydratase, short chain 1	-0.191	0.996	0.004
157822043	PLGRKT	plasminogen receptor with a C-terminal lysine	-0.191	0.951	0.049
148696094	TUBGCP4	tubulin gamma complex associated protein 4	-0.189	0.999	0.001
564399352	TAF9B	TATA-box binding protein associated factor 9b	-0.187	0.970	0.030
672088942	ATP2B3	ATPase plasma membrane Ca2+ transporting 3	-0.186	0.958	0.042
197209847	JAK1	Janus kinase 1	-0.185	0.981	0.019
16923964	CNTN1	contactin 1	-0.185	0.969	0.031

157817420	NRIP3	nuclear receptor interacting protein 3	-0.183	0.957	0.043
76881830	Kcnip2	potassium voltage-gated channel interacting protein 2	-0.183	0.982	0.018
32185285	BCL2L2	BCL2 like 2	-0.181	0.980	0.020
187468990	DNAJB2	DnaJ heat shock protein family (Hsp40) member B2	-0.180	0.968	0.032
39930507	KCNK15	potassium two pore domain channel subfamily K member 15	-0.180	0.951	0.049
13929208	Scd2	stearoyl-Coenzyme A desaturase 2	-0.178	0.991	0.009
157818193	TTPAL	alpha tocopherol transfer protein like	-0.176	0.957	0.043
564395567	NFATC3	nuclear factor of activated T cells 3	-0.173	0.957	0.043
124248495	CHID1	chitinase domain containing 1	-0.171	0.952	0.048
61557212	CIAO3	cytosolic iron-sulfur assembly component 3	-0.170	0.975	0.025
396941666	Dync1i2	dynein cytoplasmic 1 intermediate chain 2	-0.169	0.969	0.031
148668175	EDNRB	endothelin receptor type B	-0.168	0.952	0.048
19705545	RAB3IL1	RAB3A interacting protein like 1	-0.166	0.999	0.001
148747253	ATP1B1	ATPase Na+/K+ transporting subunit beta 1	-0.164	0.972	0.028
19173766	LONP1	lon peptidase 1, mitochondrial	-0.164	0.999	0.001
16758808	EPB41L3	erythrocyte membrane protein band 4.1 like 3	-0.159	0.982	0.018
52345385	PDIA6	protein disulfide isomerase family A member 6	-0.159	0.960	0.040
62078637	LCA5	lebercilin LCA5	-0.158	0.966	0.034
209529636	PPA2	inorganic pyrophosphatase 2	-0.158	0.984	0.016
18034785	ABCB6	ATP binding cassette subfamily B member 6 (Langereis blood group)	-0.158	0.971	0.029
6649914	GDF11	growth differentiation factor 11	-0.154	0.994	0.006
149024719	NOL9	nucleolar protein 9	-0.150	0.952	0.048
149032539	HECW1	HECT, C2 and WW domain containing E3 ubiquitin protein ligase 1	-0.150	0.962	0.038
71043650	SRPK1	SRSF protein kinase 1	-0.150	0.990	0.010
157819977	CERS4	ceramide synthase 4	-0.149	0.980	0.020
50510837	KIAA1191	KIAA1191	-0.148	0.988	0.012
109505096	NID1	nidogen 1	-0.148	0.954	0.046
48976085	GM2A	GM2 ganglioside activator	-0.147	0.981	0.019
148672025	MAP3K12	mitogen-activated protein kinase kinase kinase 12	-0.144	0.972	0.028
148671875	TMEM50B	transmembrane protein 50B	-0.142	0.958	0.042
16758736	NLGN1	neuroligin 1	-0.139	0.954	0.046

201066369	LGI2	leucine rich repeat LGI family member 2	-0.139	0.958	0.042
77627979	SRPRA	SRP receptor subunit alpha	-0.138	0.959	0.041
564389848	ERICH1	glutamate rich 1	-0.137	0.959	0.041
157823401	PIGH	phosphatidylinositol glycan anchor biosynthesis class H	-0.135	0.988	0.012
300794317	SFI1	SFI1 centrin binding protein	-0.133	0.953	0.047
564345487	RINT1	RAD50 interactor 1	-0.132	0.952	0.048
56605798	RNF167	ring finger protein 167	-0.131	0.996	0.004
148747541	HNRNPU	heterogeneous nuclear ribonucleoprotein U	-0.130	0.977	0.023
158711729	HACE1	HECT domain and ankyrin repeat containing E3 ubiquitin protein ligase 1	-0.128	0.979	0.021
398650648	SLC8A1	solute carrier family 8 member A1	-0.127	0.981	0.019
37359832	SCRN1	secernin 1	-0.127	0.983	0.017
77415383	HSPA8	heat shock protein family A (Hsp70) member 8	-0.125	0.960	0.040
564339312	FUBP1	far upstream element binding protein 1	-0.123	0.962	0.038
58865700	GRWD1	glutamate rich WD repeat containing 1	-0.123	0.982	0.018
253683488	NTRK2	neurotrophic receptor tyrosine kinase 2	-0.121	0.989	0.011
158254369	CDK10	cyclin dependent kinase 10	-0.121	0.952	0.048
74229032	TPCN1	two pore segment channel 1	-0.121	0.960	0.040
398303839	SH3GL2	SH3 domain containing GRB2 like 2, endophilin A1	-0.117	0.998	0.002
8393390	GABRB3	gamma-aminobutyric acid type A receptor subunit beta3	-0.115	0.980	0.020
52138635	ETFDH	electron transfer flavoprotein dehydrogenase	-0.115	0.952	0.048
149059529	LOC100910558	uncharacterized LOC100910558	-0.114	0.977	0.023
6978621	CCNG1	cyclin G1	-0.114	0.994	0.006
404247435	YLPM1	YLP motif containing 1	-0.113	0.997	0.003
56090463	GORASP2	golgi reassembly stacking protein 2	-0.113	0.981	0.019
157786602	NHP2	NHP2 ribonucleoprotein	-0.110	0.995	0.005
274326692	UQCC3	ubiquinol-cytochrome c reductase complex assembly factor 3	-0.106	0.989	0.011
58865936	SIKE1	suppressor of IKBKE 1	-0.100	0.980	0.020
403377905	SRGAP2	SLIT-ROBO Rho GTPase activating protein 2	-0.098	0.961	0.039
672057488	CD63	CD63 molecule	-0.096	0.955	0.045
149060725	CEP19	centrosomal protein 19	-0.095	0.982	0.018

154800420	GNL3L	G protein nucleolar 3 like	-0.093	0.976	0.024
16258813	VHL	von Hippel-Lindau tumor suppressor	-0.089	0.962	0.038
8980843	GRIPAP1	GRIP1 associated protein 1	-0.089	0.974	0.026
16758578	DPP3	dipeptidyl peptidase 3	-0.084	0.987	0.013
50054266	NLN	neurolysin	-0.082	0.989	0.011
564343911	RPN2	ribophorin II	-0.076	0.973	0.027
297206894	E4F1	E4F transcription factor 1	-0.074	0.954	0.046
564375060	SLC39A11	solute carrier family 39 member 11	-0.070	0.991	0.009
148747528	PTK2B	protein tyrosine kinase 2 beta	-0.066	0.995	0.005
564328458	Ldha/RGD1562690	lactate dehydrogenase A	-0.064	0.953	0.047
255918181	NUS1	NUS1 dehydrodolichyl diphosphate synthase subunit	-0.062	0.985	0.015
20302113	STIP1	stress induced phosphoprotein 1	-0.060	0.973	0.027
213688386	GTF2E1	general transcription factor IIE subunit 1	-0.058	0.960	0.040
13385318	KDELR2	KDEL endoplasmic reticulum protein retention receptor 2	-0.057	0.966	0.034
149049470	TPI1	triosephosphate isomerase 1	-0.057	0.979	0.021
162287208	FADS1	fatty acid desaturase 1	-0.050	0.999	0.001
76443681	USP11	ubiquitin specific peptidase 11	0.053	-0.995	0.005
149039803	UBQLN1	ubiquilin 1	0.065	-0.965	0.035
157822779	DNAJC11	DnaJ heat shock protein family (Hsp40) member C11	0.066	-0.984	0.016
564353714	FBXO42	F-box protein 42	0.074	-0.985	0.015
564326269	RPL28	ribosomal protein L28	0.075	-0.952	0.048
157817783	SNX18	sorting nexin 18	0.079	-0.986	0.014
169790975	MRPS9	mitochondrial ribosomal protein S9	0.082	-0.957	0.043
83649695	SMIM14	small integral membrane protein 14	0.082	-0.986	0.014
60360532	OSBPL6	oxysterol binding protein like 6	0.083	-0.963	0.037
214010118	TMEM59	transmembrane protein 59	0.085	-0.983	0.017
6978449	ADD2	adducin 2	0.085	-0.961	0.039
290560659	ZNF609	zinc finger protein 609	0.090	-0.999	0.001
564363529	NCAM1	neural cell adhesion molecule 1	0.091	-0.958	0.042
149038024	RIPOR1	RHO family interacting cell polarization regulator 1	0.091	-1.000	0.000
564332984	OSBP	oxysterol binding protein	0.094	-0.994	0.006
14389301	SMPD2	sphingomyelin phosphodiesterase 2	0.096	-0.963	0.037
166064004	GTF3A	general transcription factor IIIA	0.101	-0.958	0.042
62543537	TBC1D10A	TBC1 domain family member 10A	0.108	-0.971	0.029
157818159	AAR2	AAR2 splicing factor	0.110	-0.959	0.041
157786720	HIVEP1	HIVEP zinc finger 1	0.111	-0.952	0.048
30794434	SRRM4	serine/arginine repetitive matrix 4	0.112	-0.977	0.023
157817811	C5orf22	chromosome 5 open reading frame 22	0.113	-0.971	0.029

170295834	NDUFA10	NADH:ubiquinone oxidoreductase subunit A10	0.114	-0.974	0.026
51948396	TUSC3	tumor suppressor candidate 3	0.116	-0.954	0.046
274321371	CRLF3	cytokine receptor like factor 3	0.118	-0.972	0.028
68163425	TMEM199	transmembrane protein 199	0.118	-0.953	0.047
18959272	KCNQ2	potassium voltage-gated channel subfamily Q member 2	0.119	-0.960	0.040
293344794	FAM160B1	family with sequence similarity 160 member B1	0.121	-0.966	0.034
114326177	SHMT1	serine hydroxymethyltransferase 1	0.123	-0.972	0.028
58866022	MGAT4A	alpha-1,3-mannosyl-glycoprotein 4-beta-N-acetylglucosaminyltransferase A	0.127	-0.977	0.023
454601639	NCOA6	nuclear receptor coactivator 6	0.127	-0.983	0.017
672068548	SUPT6H	SPT6 homolog, histone chaperone and transcription elongation factor	0.127	-0.988	0.012
564331450	EEF1AKMT2	EEF1A lysine methyltransferase 2	0.129	-0.992	0.008
564361462	BRD1	bromodomain containing 1	0.130	-0.957	0.043
564363852	SNUPN	snurportin 1	0.131	-0.975	0.025
300797157	TBC1D8	TBC1 domain family member 8	0.131	-0.958	0.042
60359978	KIF3C	kinesin family member 3C	0.131	-0.963	0.037
148693260	TIMM29	translocase of inner mitochondrial membrane 29	0.131	-0.979	0.021
157824037	USP4	ubiquitin specific peptidase 4	0.131	-0.976	0.024
74139306	TMED9	transmembrane p24 trafficking protein 9	0.143	-0.982	0.018
9624979	ENSA	endosulfine alpha	0.144	-0.962	0.038
157820585	SART3	spliceosome associated factor 3, U4/U6 recycling protein	0.146	-0.985	0.015
149064388	Hmgxb3	HMG-box containing 3	0.147	-0.964	0.036
40786455	BPGM	bisphosphoglycerate mutase	0.150	-0.996	0.004
300253233	LEMD3	LEM domain containing 3	0.154	-0.983	0.017
209863130	SEMA3F	semaphorin 3F	0.155	-0.953	0.047
50510427	IP6K1	inositol hexakisphosphate kinase 1	0.156	-0.967	0.033
77917548	DUS3L	dihydrouridine synthase 3 like	0.158	-0.993	0.007
300793740	TANC2	tetratricopeptide repeat, ankyrin repeat and coiled-coil containing 2	0.158	-0.966	0.034
149033480	Zfp956	zinc finger protein 956	0.163	-0.985	0.015
672072928	CUX2	cut like homeobox 2	0.164	-0.997	0.003
60359854	POLDIP3	DNA polymerase delta interacting protein 3	0.164	-0.956	0.044
564340867	MMADHC	metabolism of cobalamin associated D	0.165	-0.969	0.031

76559929	NOC2L	NOC2 like nucleolar associated transcriptional repressor	0.165	-0.990	0.010
293359997	SGPP1	sphingosine-1-phosphate phosphatase 1	0.170	-0.962	0.038
157818643	KCTD3	potassium channel tetramerization domain containing 3	0.170	-0.964	0.036
33356154	UBE2H	ubiquitin conjugating enzyme E2 H	0.172	-0.954	0.046
56605790	HCFC2	host cell factor C2	0.173	-0.975	0.025
568972665	TSPOAP1	TSPO associated protein 1	0.173	-0.965	0.035
564298823	EML3	EMAP like 3	0.177	-0.962	0.038
149054120	ORMDL3	ORMDL sphingolipid biosynthesis regulator 3	0.179	-0.983	0.017
149044006	TEDC1	tubulin epsilon and delta complex 1	0.179	-0.966	0.034
392333209	DLG5	discs large MAGUK scaffold protein 5	0.180	-0.984	0.016
62079005	SLAIN1	SLAIN motif family member 1	0.180	-0.956	0.044
157822303	GPR107	G protein-coupled receptor 107	0.182	-0.959	0.041
157817773	ZNF641	zinc finger protein 641	0.183	-0.980	0.020
392342217	RANBP3	RAN binding protein 3	0.184	-0.952	0.048
762006019	FAM8A1	family with sequence similarity 8 member A1	0.185	-0.970	0.030
66730335	SUMO3	small ubiquitin like modifier 3	0.188	-0.972	0.028
672085227	USP10	ubiquitin specific peptidase 10	0.191	-0.991	0.009
148683335	SLC25A44	solute carrier family 25 member 44	0.191	-0.987	0.013
197313795	MTX1	metaxin 1	0.192	-0.967	0.033
157823197	NDUFB7	NADH:ubiquinone oxidoreductase subunit B7	0.194	-0.989	0.011
157821953	NXPE3	neurexophilin and PC-esterase domain family member 3	0.197	-0.974	0.026
157820969	SBNO2	strawberry notch homolog 2	0.197	-0.977	0.023
157820401	ABHD2	abhydrolase domain containing 2, acylglycerol lipase	0.197	-0.966	0.034
57164107	NIPSNAP3A	nipsnap homolog 3A	0.199	-0.984	0.016
157821579	BICD1	BICD cargo adaptor 1	0.202	-0.984	0.016
219277692	NDUFB2	NADH:ubiquinone oxidoreductase subunit B2	0.203	-0.971	0.029
61557021	BFAR	bifunctional apoptosis regulator	0.204	-0.983	0.017
564383487	SLAIN2	SLAIN motif family member 2	0.206	-0.970	0.030
157817674	ATP5MF-PTCD1	ATP5MF-PTCD1 readthrough	0.207	-0.989	0.011
61556748	TSPYL1	TSPY like 1	0.211	-0.994	0.006
84781638	KLHL25	kelch like family member 25	0.215	-0.961	0.039
189163477	SCAF4	SR-related CTD associated factor 4	0.216	-0.991	0.009
396080328	ADCYAP1R1	ADCYAP receptor type I	0.217	-0.980	0.020

66911118	NFX1	nuclear transcription factor, X-box binding 1	0.217	-0.968	0.032
765099233	LMNB2	lamin B2	0.219	-0.959	0.041
2804296	CDH8	cadherin 8	0.221	-0.972	0.028
288541353	CMTM4	CKLF like MARVEL transmembrane domain containing 4	0.222	-0.970	0.030
148669751	SMNDC1	survival motor neuron domain containing 1	0.223	-0.973	0.027
293340917	C3orf70	chromosome 3 open reading frame 70	0.223	-0.951	0.049
117940043	MED22	mediator complex subunit 22	0.225	-0.960	0.040
148696931	ARRDC2	arrestin domain containing 2	0.227	-0.979	0.021
148670791	ZFYVE1	zinc finger FYVE-type containing 1	0.232	-0.963	0.037
149032040	SLC11A2	solute carrier family 11 member 2	0.234	-0.961	0.039
564340133	GTF3C4	general transcription factor IIIC subunit 4	0.236	-0.956	0.044
37360264	TRMT6	tRNA methyltransferase 6	0.236	-0.971	0.029
40018556	NOB1	NIN1 (RPN12) binding protein 1 homolog	0.237	-0.974	0.026
564359486	TBC1D30	TBC1 domain family member 30	0.237	-0.969	0.031
157821413	USP30	ubiquitin specific peptidase 30	0.250	-0.977	0.023
157823719	TRAIP	TRAF interacting protein	0.252	-0.979	0.021
209954792	PDCD2	programmed cell death 2	0.254	-0.961	0.039
157821283	C19orf47	chromosome 19 open reading frame 47	0.257	-0.950	0.050
119388826	TFPT	TCF3 fusion partner	0.258	-0.958	0.042
564367958	SEMA4C	semaphorin 4C	0.258	-0.969	0.031
281427178	CEP76	centrosomal protein 76	0.262	-0.995	0.005
67078454	SLC25A51	solute carrier family 25 member 51	0.263	-0.990	0.010
197382256	PHF12	PHD finger protein 12	0.263	-0.972	0.028
380877082	NAXE	NAD(P)HX epimerase	0.263	-0.959	0.041
564353880	DDI2	DNA damage inducible 1 homolog 2	0.264	-0.988	0.012
148696370	PANK2	pantothenate kinase 2	0.270	-0.989	0.011
564394925	TENT4B	terminal nucleotidyltransferase 4B	0.276	-0.963	0.037
564365504	CCDC51	coiled-coil domain containing 51	0.277	-0.950	0.050
197386048	PTRHD1	peptidyl-tRNA hydrolase domain containing 1	0.280	-0.964	0.036
157821325	TWNK	twinkle mtDNA helicase	0.283	-0.982	0.018
149052738	RGD1561277	RGD1561277	0.285	-0.999	0.001
62078733	MAK16	MAK16 homolog	0.287	-0.984	0.016
672020915	VCPKMT	valosin containing protein lysine methyltransferase	0.288	-0.954	0.046
76559919	N4BP3	NEDD4 binding protein 3	0.292	-0.994	0.006
166795897	PIMREG	PICALM interacting mitotic regulator	0.293	-0.981	0.019

56789732	VSTM5	V-set and transmembrane domain containing 5	0.295	-0.981	0.019
68163385	GPATCH4	G-patch domain containing 4	0.297	-0.966	0.034
157821997	MED28	mediator complex subunit 28	0.297	-0.955	0.045
157822519	CBLN4	cerebellin 4 precursor	0.299	-0.998	0.002
157819365	TBC1D25	TBC1 domain family member 25	0.302	-0.987	0.013
157819315	OSBPL11	oxysterol binding protein like 11	0.306	-0.994	0.006
51491900	TOR1A	torsin family 1 member A	0.308	-0.994	0.006
50510821	AMIGO1	adhesion molecule with Ig like domain 1	0.310	-0.971	0.029
33086606	SRPRB	SRP receptor subunit beta	0.318	-0.958	0.042
300793780	ZNF251	zinc finger protein 251	0.321	-0.995	0.005
149051028	RNF144A	ring finger protein 144A	0.322	-0.984	0.016
41386747	ZC3H18	zinc finger CCCH-type containing 18	0.322	-0.999	0.001
672030183	H2AC12	H2A clustered histone 12	0.323	-0.997	0.003
76362828	TEF	TEF transcription factor, PAR bZIP family member	0.327	-0.975	0.025
68163537	NXPE4	neurexophilin and PC-esterase domain family member 4	0.329	-0.967	0.033
157817260	LTO1	LTO1 maturation factor of ABCE1	0.330	-0.955	0.045
582015198	CRY2	cryptochrome circadian regulator 2	0.332	-0.979	0.021
564333920	PPRC1	PPARG related coactivator 1	0.333	-0.980	0.020
564307081	ATXN7L1	ataxin 7 like 1	0.339	-0.971	0.029
564350836	MELK	maternal embryonic leucine zipper kinase	0.341	-0.976	0.024
40018598	ANGPTL4	angiopoietin like 4	0.356	-0.979	0.021
57528321	RIOK2	RIO kinase 2	0.360	-0.997	0.003
74183022	Zfp773	zinc finger protein 773	0.360	-0.958	0.042
157822027	CSRNP2	cysteine and serine rich nuclear protein 2	0.361	-0.995	0.005
300794219	OPN3	opsin 3	0.362	-0.960	0.040
300797828	KAT14	lysine acetyltransferase 14	0.365	-0.981	0.019
157817720	SLC16A14	solute carrier family 16 member 14	0.374	-0.981	0.019
213972545	MXD1	MAX dimerization protein 1	0.378	-0.976	0.024
164565364	ITPKB	inositol-trisphosphate 3-kinase B	0.380	-0.955	0.045
14388593	SPATA2	spermatogenesis associated 2	0.385	-0.984	0.016
564382837	LIN54	lin-54 DREAM MuvB core complex component	0.387	-0.953	0.047
8392855	ADCYAP1	adenylate cyclase activating polypeptide 1	0.397	-0.961	0.039
40789237	PCDHA4	protocadherin alpha 4	0.398	-0.955	0.045
157821403	RASSF7	Ras association domain family member 7	0.399	-0.997	0.003
564318492	TASOR	transcription activation suppressor	0.403	-0.955	0.045

157817446	LINGO2	leucine rich repeat and Ig domain containing 2	0.403	-0.955	0.045
293345066	PPIL6	peptidylprolyl isomerase like 6	0.408	-0.965	0.035
66730347	PTPRCAP	protein tyrosine phosphatase receptor type C associated protein	0.415	-0.959	0.041
38454286	STIMATE-MUSTN1	STIMATE-MUSTN1 readthrough	0.425	-0.952	0.048
564311452	TMEM131	transmembrane protein 131	0.443	-0.973	0.027
53850630	LOC100362724/ MGC95208	similar to 4930453N24Rik protein	0.456	-0.983	0.017
8392993	BMP3	bone morphogenetic protein 3	0.461	-0.953	0.047
255708448	KATNA1	katanin catalytic subunit A1	0.466	-0.971	0.029
148706598	PKDCC	protein kinase domain containing, cytoplasmic	0.469	-0.987	0.013
51948492	NUDT19	nudix hydrolase 19	0.470	-0.985	0.015
70912374	CCNQ	cyclin Q	0.479	-0.984	0.016
19424300	GCHFR	GTP cyclohydrolase I feedback regulator	0.519	-0.957	0.043
89145411	SULT2B1	sulfotransferase family 2B member 1	0.524	-0.997	0.003
404434380	ZNF133	zinc finger protein 133	0.530	-0.982	0.018
148687591	TMEM132D	transmembrane protein 132D	0.550	-0.976	0.024
213688370	EXOSC7	exosome component 7	0.551	-0.979	0.021
219879771	PGAP3	post-GPI attachment to proteins phospholipase 3	0.560	-0.997	0.003
149023178	CEP152	centrosomal protein 152	0.572	-0.962	0.038
148664537	Gm10269	ribosomal protein L35 pseudogene	0.582	-0.993	0.007
511094004	RUNX2	RUNX family transcription factor 2	0.585	-0.970	0.030
24415396	GPR3	G protein-coupled receptor 3	0.604	-0.970	0.030
13994119	KHK	ketohexokinase	0.612	-0.951	0.049
22122541	LRRC3B	leucine rich repeat containing 3B	0.615	-0.973	0.027
157822359	PELI2	pellino E3 ubiquitin protein ligase family member 2	0.620	-0.996	0.004
157820433	CPEB1	cytoplasmic polyadenylation element binding protein 1	0.624	-0.970	0.030
1083798	Bmpr1b	bone morphogenetic protein receptor type 1B	0.630	-0.985	0.015
197386066	ZNF784	zinc finger protein 784	0.633	-0.996	0.004
157823803	DOK3	docking protein 3	0.639	-0.973	0.027
149025186	RPS6KL1	ribosomal protein S6 kinase like 1	0.661	-0.971	0.029
392354293	Hmgb3	high mobility group box 3	0.709	-0.996	0.004
80861398	CRY1	cryptochrome circadian regulator 1	0.720	-0.990	0.010
38454200	CHDH	choline dehydrogenase	0.734	-0.965	0.035
293339965	RAB11FIP3	RAB11 family interacting protein 3	0.750	-0.988	0.012

672086719	FAM184A	family with sequence similarity 184 member A	0.836	-0.986	0.014
189011634	ARMC7	armadillo repeat containing 7	0.850	-0.976	0.024
672025117	MBTD1	mbt domain containing 1	0.886	-0.988	0.012
392339806	CFAP69	cilia and flagella associated protein 69	0.915	-0.975	0.025
9506775	HES2	hes family bHLH transcription factor 2	0.963	-0.970	0.030
157821687	NEURL2	neuralized E3 ubiquitin protein ligase 2	0.966	-0.970	0.030
148693657	DDX6	DEAD-box helicase 6	0.969	-0.996	0.004
82654234	LILRA6	leukocyte immunoglobulin like receptor A6	1.000	-0.985	0.015
148235584	CLEC4A	C-type lectin domain family 4 member A	1.000	-0.970	0.030
149065466	ARHGEF5	Rho guanine nucleotide exchange factor 5	1.000	-0.970	0.030
404501522	NXNL1	nucleoredoxin like 1	1.037	-0.970	0.030
149067796	TMEM219	transmembrane protein 219	1.064	-0.958	0.042
19424314	KCNE2	potassium voltage-gated channel subfamily E regulatory subunit 2	1.066	-0.999	0.001
564296988	ZNF235	zinc finger protein 235	1.072	-0.974	0.026
188536090	FAM241B	family with sequence similarity 241 member B	1.127	-0.984	0.016
62078917	PAQR5	progestin and adipoQ receptor family member 5	1.181	-0.999	0.001
6978493	ALOX5	arachidonate 5-lipoxygenase	1.193	-0.985	0.015
157787081	WNT1	Wnt family member 1	1.222	-0.976	0.024
569012000	KLF8	Kruppel like factor 8	1.406	-0.966	0.034
157817264	ANKRD23	ankyrin repeat domain 23	1.505	-0.996	0.004
158533972	SPTA1	spectrin alpha, erythrocytic 1	1.585	-0.970	0.030
149058209	SELE	selectin E	1.585	-0.970	0.030
157818463	Zfp93	zinc finger protein 93	1.597	-0.955	0.045
672013187	DMWD	DM1 locus, WD repeat containing	1.605	-0.978	0.022
41054896	FUT7	fucosyltransferase 7	1.700	-0.982	0.018
568990288	NIPBL	NIPBL cohesin loading factor	1.705	-0.967	0.033
157819799	IQCH	IQ motif containing H	1.716	-0.970	0.030
149032888	LOC100910237	uncharacterized LOC100910237	1.751	-0.986	0.014
157820135	CHRDL2	chordin like 2	1.762	-0.985	0.015
564318930	WDR17	WD repeat domain 17	1.807	-0.980	0.020
157821823	Ngp	neutrophilic granule protein	1.807	-0.980	0.020
392342449	PRSS56	serine protease 56	1.848	-0.958	0.042
149042883	LOC100365365	rCG32328-like	1.861	-0.993	0.007
189181736	LAD1	ladinin 1	1.874	-0.972	0.028

16758254	CNGA1	cyclic nucleotide gated channel subunit alpha 1	1.874	-0.957	0.043
197381585	Urah	urate (5-hydroxyiso-) hydrolase	1.976	-0.970	0.030
160961485	MYLK3	myosin light chain kinase 3	2.000	-0.970	0.030
672070295	BAHCC1	BAH domain and coiled-coil containing 1	2.083	-0.975	0.025
28972866	CSMD3	CUB and Sushi multiple domains 3	2.140	-0.994	0.006
293352381	PAN3	poly(A) specific ribonuclease subunit PAN3	2.239	-0.983	0.017
197385133	RGD1561157	RGD1561157	2.303	-0.971	0.029
71896592	IGFALS	insulin like growth factor binding protein acid labile subunit	2.322	-0.970	0.030
8393941	PADI4	peptidyl arginine deiminase 4	2.322	-0.970	0.030
564324736	L3MBTL3	L3MBTL histone methyl-lysine binding protein 3	2.353	-0.956	0.044
157818163	POF1B	POF1B actin binding protein	2.392	-0.998	0.002
564329376	SRPK3	SRSF protein kinase 3	2.585	-0.970	0.030
58866038	XKRX	XK related X-linked	2.585	-0.970	0.030
11120690	NR1H4	nuclear receptor subfamily 1 group H member 4	2.585	-0.970	0.030
13540693	MYOC	myocilin	2.585	-0.970	0.030
57222314	OAS3	2'-5'-oligoadenylate synthetase 3	2.585	-0.970	0.030
8394529	VDR	vitamin D receptor	2.585	-0.970	0.030
25742760	AMH	anti-Mullerian hormone	2.807	-0.970	0.030
157787002	Dpt	dermatopontin	2.807	-0.970	0.030
197384923	C1orf87	chromosome 1 open reading frame 87	3.000	-0.970	0.030
13591993	MMP9	matrix metallopeptidase 9	3.000	-0.970	0.030
300796937	ESPNL	espin like	3.000	-0.970	0.030
564347547	LOC103690120	probable N-acetyltransferase CML1	3.030	-0.999	0.001
281332212	SH2D4B	SH2 domain containing 4B	3.170	-0.970	0.030
260099641	MSH5	mutS homolog 5	3.170	-0.970	0.030
564392795	MOCOS	molybdenum cofactor sulfurase	3.248	-0.983	0.017
61556961	THEG	theg spermatid protein	3.322	-0.970	0.030
28174920	RPL17	ribosomal protein L17	3.389	-0.978	0.022
569009290	TENM1	teneurin transmembrane protein 1	3.450	-0.975	0.025
25282405	BPIFA1	BPI fold containing family A member 1	3.459	-0.970	0.030
16758550	BCL2L10	BCL2 like 10	3.459	-0.970	0.030
148670929	BATF	basic leucine zipper ATF-like transcription factor	3.700	-0.970	0.030
21245088	Ly6a (includes others)	lymphocyte antigen 6 complex, locus A	3.807	-0.970	0.030
148747510	BAAT	bile acid-CoA:amino acid N-acyltransferase	3.807	-0.970	0.030

158187515	OAZ3	ornithine decarboxylase antizyme 3	3.807	-0.970	0.030
62078779	ORAI3	ORAI calcium release-activated calcium modulator 3	3.807	-0.958	0.042
672052120	RBM12B	RNA binding motif protein 12B	3.907	-0.978	0.022
564309734	IGSF9B	immunoglobulin superfamily member 9B	4.173	-0.970	0.030
8393641	AADAT	aminoadipate aminotransferase	4.248	-0.970	0.030
47577861	OR7D2	olfactory receptor family 7 subfamily D member 2	4.248	-0.951	0.049
27545443	CEACAM4	CEA cell adhesion molecule 4	4.392	-0.985	0.015
341940965	MOS	MOS proto-oncogene, serine/threonine kinase	4.492	-0.979	0.021
149038931	CNTRL	centriolin	4.492	-0.964	0.036
9506733	GJB5	gap junction protein beta 5	4.907	-0.970	0.030
59709455	EPOR	erythropoietin receptor	5.170	-0.964	0.036
8394516	PLAUR	plasminogen activator, urokinase receptor	5.229	-0.970	0.030
16758218	Hamp	hepcidin antimicrobial peptide	5.267	-0.973	0.027
20301998	PROK2	prokineticin 2	5.358	-0.985	0.015
672052120	RBM12B	RNA binding motif protein 12B	6.366	-0.969	0.031
293347435	PTPRD	protein tyrosine phosphatase receptor type D	7.710	-0.980	0.020
149057336	ZSCAN2	zinc finger and SCAN domain containing 2	7.758	-0.985	0.015
62650795	DACT1	dishevelled binding antagonist of beta catenin 1	7.762	-0.984	0.016
109472884	UBE3C	ubiquitin protein ligase E3C	8.197	-0.976	0.024
672029702	TUT7	terminal uridylyl transferase 7	8.441	-0.957	0.043

Supplementary Table S10. The list of genes that are differentially expressed in the offspring hippocampus in response to prenatal BPA exposure that exhibited the changes in the expression levels correlated with the cell density in CA2/3 of the hippocampus. The transcriptome profiling data of DEGs in male and female rat offspring prenatally exposed to BPA (n = 6, male pups n = 3 and female pups n = 3, from independent litters) or the vehicle control (n = 6, male pups n = 3 and female pups n = 3, from independent litters) were obtained and used for the PTM analyses to identify DEGs that exhibited the changes in the expression levels correlated with the cell density in CA2/3 of the hippocampus.

ID	Symbol	Entrez Gene Name	log2(FC)	R values	P-values
148675846	FAM114A2	family with sequence similarity 114 member A2	-9.453	0.967	0.033
672027854	APBB2	amyloid beta precursor protein binding family B member 2	-8.723	0.969	0.031
293347435	PTPRD	protein tyrosine phosphatase receptor type D	-7.731	0.978	0.022
564375502	Mxra7	matrix-remodelling associated 7	-7.209	0.990	0.010
392339806	CFAP69	cilia and flagella associated protein 69	-6.820	0.977	0.023
564314389	DZIP3	DAZ interacting zinc finger protein 3	-6.700	0.996	0.004
149020633	TAF1D	TATA-box binding protein associated factor, RNA polymerase I subunit D	-6.476	0.997	0.003
157818475	SMIM22	small integral membrane protein 22	-5.615	0.989	0.011
567315993	LOC102550396	LRRGT00188	-5.600	0.992	0.008
564312230	LOC100912948	multidrug resistance-associated protein 1-like	-5.285	0.990	0.010
672029704	TUT7	terminal uridylyl transferase 7	-5.211	0.958	0.042
564313676	FBF1	Fas binding factor 1	-5.170	0.950	0.050
197384778	Snorc	secondary ossification center associated regulator of chondrocyte maturation	-5.044	0.987	0.013
672035060	CIC	capicua transcriptional repressor	-4.863	0.975	0.025
564323305	LOC681300	similar to CXXC finger 5	-4.672	0.998	0.002
209447125	Ctf2	cardiotrophin 2	-4.392	0.990	0.010
201860265	NRN1L	neuritin 1 like	-4.358	0.994	0.006
300798104	IFNLR1	interferon lambda receptor 1	-4.248	0.986	0.014
51591901	MPIG6B	megakaryocyte and platelet inhibitory receptor G6b	-4.170	0.990	0.010
564298047	GDPD5	glycerophosphodiester phosphodiesterase domain containing 5	-4.163	0.993	0.007
300798035	NRG4	neuregulin 4	-4.044	0.995	0.005
61556838	Raet1d/Raet1e	retinoic acid early transcript 1E	-4.044	0.995	0.005
6978515	APOA1	apolipoprotein A1	-3.807	0.990	0.010

117647210	CTRC	chymotrypsin C	-3.807	0.990	0.010
8392926	ASGR2	asialoglycoprotein receptor 2	-3.700	0.955	0.045
48040447	SUCNR1	succinate receptor 1	-3.700	0.990	0.010
194473646	UPK3A	uroplakin 3A	-3.585	0.990	0.010
164518908	RAB25	RAB25, member RAS oncogene family	-3.459	0.990	0.010
285026465	HS3ST3A1	heparan sulfate-glucosamine 3-sulfotransferase 3A1	-3.322	0.990	0.010
148710035	PITX3	paired like homeodomain 3	-3.322	0.990	0.010
56788780	KRT19	keratin 19	-3.248	0.988	0.012
9506451	CA5A	carbonic anhydrase 5A	-3.248	0.998	0.002
56090299	ODF4	outer dense fiber of sperm tails 4	-3.170	0.987	0.013
157822159	CCDC42	coiled-coil domain containing 42	-3.170	0.990	0.010
123173794	GSG1	germ cell associated 1	-3.170	0.990	0.010
392339806	CFAP69	cilia and flagella associated protein 69	-2.947	0.967	0.033
77917534	CBLC	Cbl proto-oncogene C	-2.807	0.990	0.010
157822121	LRMDA	leucine rich melanocyte differentiation associated	-2.747	0.979	0.021
51948496	SLC22A18	solute carrier family 22 member 18	-2.687	1.000	0.000
672032217	REPS2	RALBP1 associated Eps domain containing 2	-2.683	0.973	0.027
298566276	Ces1a	carboxylesterase 1A	-2.585	0.990	0.010
57222328	PFN4	profilin family member 4	-2.585	0.990	0.010
157786772	KREMEN2	kringle containing transmembrane protein 2	-2.585	0.990	0.010
306482632	BPIFB4	BPI fold containing family B member 4	-2.585	0.990	0.010
57222300	Klra2	killer cell lectin-like receptor, subfamily A, member 2	-2.585	0.990	0.010
157822463	Nkx6-3	NK6 homeobox 3	-2.585	0.990	0.010
157822853	GUCA1A	guanylate cyclase activator 1A	-2.509	0.953	0.047
162417984	Vom2r12 (includes others)	vomeronasal 2 receptor, 80	-2.459	0.968	0.032
564391680	MAP3K8	mitogen-activated protein kinase kinase kinase 8	-2.426	0.995	0.005
62945342	LAX1	lymphocyte transmembrane adaptor 1	-2.322	0.990	0.010
187469451	CLEC7A	C-type lectin domain containing 7A	-2.322	0.990	0.010
62079023	ADTRP	androgen dependent TFPI regulating protein	-2.246	0.969	0.031
404247470	OLR1	oxidized low density lipoprotein receptor 1	-2.170	0.965	0.035
66730349	SPIB	Spi-B transcription factor	-2.170	0.998	0.002
300797305	TMEM45A	transmembrane protein 45A	-2.093	0.964	0.036
148672128	SMAGP	small cell adhesion glycoprotein	-2.077	1.000	0.000

55741882	ZPBP2	zona pellucida binding protein 2	-2.022	0.994	0.006
157822587	PDE6B	phosphodiesterase 6B	-2.000	0.990	0.010
157818091	TMEM182	transmembrane protein 182	-2.000	0.990	0.010
296483047	SIX1	SIX homeobox 1	-2.000	0.990	0.010
117647206	DDX4	DEAD-box helicase 4	-2.000	0.990	0.010
19424304	CHRNB3	cholinergic receptor nicotinic beta 3 subunit	-2.000	0.990	0.010
47577151	Olfr1441	olfactory receptor 1441	-1.972	0.990	0.010
56676350	PRSS35	serine protease 35	-1.962	0.968	0.032
117647214	EDN3	endothelin 3	-1.913	1.000	0.000
81295367	Abcg3	ATP binding cassette subfamily G member 3	-1.898	0.992	0.008
672029702	TUT7	terminal uridylyl transferase 7	-1.854	0.958	0.042
13592031	PTGER2	prostaglandin E receptor 2	-1.845	0.988	0.012
19424240	PCSK4	proprotein convertase subtilisin/kexin type 4	-1.740	0.952	0.048
9910234	IFIT1B	interferon induced protein with tetratricopeptide repeats 1B	-1.678	0.990	0.010
31542125	ALOX15	arachidonate 15-lipoxygenase	-1.611	0.994	0.006
58331159	GSTA3	glutathione S-transferase alpha 3	-1.603	0.979	0.021
76443687	SLC4A1	solute carrier family 4 member 1 (Diego blood group)	-1.599	0.995	0.005
157821527	RHOD	ras homolog family member D	-1.597	0.994	0.006
157819393	NNMT	nicotinamide N-methyltransferase	-1.585	0.976	0.024
209870037	INSRR	insulin receptor related receptor	-1.585	0.980	0.020
194473618	SCX	scleraxis bHLH transcription factor	-1.585	0.965	0.035
157818603	CLCA2	chloride channel accessory 2	-1.585	0.990	0.010
685156911	NLRP4	NLR family pyrin domain containing 4	-1.585	0.990	0.010
149034165	GALNT15	polypeptide N-acetylgalactosaminyltransferase 15	-1.561	0.989	0.011
13929126	GALNT5	polypeptide N-acetylgalactosaminyltransferase 5	-1.478	0.972	0.028
81295349	SLC52A3	solute carrier family 52 member 3	-1.453	0.997	0.003
283806636	ZNF831	zinc finger protein 831	-1.433	0.994	0.006
31377521	S1PR5	sphingosine-1-phosphate receptor 5	-1.426	0.958	0.042
77917586	GRAP2	GRB2 related adaptor protein 2	-1.412	0.963	0.037
157822105	SLC49A3	solute carrier family 49 member 3	-1.389	0.982	0.018
672025117	MBTD1	mbt domain containing 1	-1.344	0.952	0.048
6978717	CTRB2	chymotrypsinogen B2	-1.342	0.989	0.011
157786780	MELTF	melanotransferrin	-1.333	0.958	0.042
29789044	SNAI2	snail family transcriptional repressor 2	-1.328	0.999	0.001
157823345	LRR1	leucine rich repeat protein 1	-1.322	0.955	0.045
307746876	Pzp	PZP, alpha-2-macroglobulin like	-1.322	0.990	0.010

209364566	COL4A3	collagen type IV alpha 3 chain	-1.280	0.961	0.039
148694035	SENP8	SUMO peptidase family member, NEDD8 specific	-1.276	0.997	0.003
62656582	KIAA0100	KIAA0100	-1.266	0.998	0.002
13162326	SLC27A5	solute carrier family 27 member 5	-1.263	0.954	0.046
672034215	ZNF729	zinc finger protein 729	-1.256	0.951	0.049
187281975	DENND1C	DENN domain containing 1C	-1.216	0.972	0.028
56119141	BTK	Bruton tyrosine kinase	-1.205	0.973	0.027
564300462	DCHS2	dachsous cadherin-related 2	-1.190	0.991	0.009
58866008	TMC5	transmembrane channel like 5	-1.180	0.964	0.036
392334475	Myb	MYB proto-oncogene, transcription factor	-1.175	0.980	0.020
281332082	THBS2	thrombospondin 2	-1.164	0.966	0.034
157823801	SLC50A1	solute carrier family 50 member 1	-1.136	0.999	0.001
60223053	SEPTIN1	septin 1	-1.133	0.973	0.027
125347412	FAM72A	family with sequence similarity 72 member A	-1.106	0.961	0.039
24308484	SLC17A8	solute carrier family 17 member 8	-1.089	0.957	0.043
157819247	CPA4	carboxypeptidase A4	-1.087	0.990	0.010
564310671	NBEAL2	neurobeachin like 2	-1.084	0.960	0.040
320089574	FAM161A	FAM161 centrosomal protein A	-1.080	0.960	0.040
300797017	ATG16L2	autophagy related 16 like 2	-1.079	0.968	0.032
672020326	MTA3	metastasis associated 1 family member 3	-1.047	0.967	0.033
164448680	HBB	hemoglobin subunit beta	-1.042	0.956	0.044
300797728	MGST3	microsomal glutathione S-transferase 3	-1.011	0.968	0.032
25453414	ASS1	argininosuccinate synthase 1	-1.000	0.982	0.018
156231008	PRND	prion like protein doppel	-1.000	0.990	0.010
765826426	Acot6	acyl-CoA thioesterase 6	-1.000	0.988	0.012
59676595	FAM20A	FAM20A golgi associated secretory pathway pseudokinase	-1.000	0.990	0.010
569009290	TENM1	teneurin transmembrane protein 1	-0.985	0.999	0.001
407228396	THEMIS2	thymocyte selection associated family member 2	-0.980	0.995	0.005
157817157	FAM166C	family with sequence similarity 166 member C	-0.979	0.995	0.005
215276950	PKP2	plakophilin 2	-0.973	0.958	0.042
672033256	LOC100912904	disks large homolog 5-like	-0.960	0.981	0.019
157820951	PRSS53	serine protease 53	-0.959	0.979	0.021
157817989	MDFI	MyoD family inhibitor	-0.958	0.982	0.018
672052120	RBM12B	RNA binding motif protein 12B	-0.957	0.978	0.022
157818989	LRRC71	leucine rich repeat containing 71	-0.952	0.962	0.038

157818275	KCNG4	potassium voltage-gated channel modifier subfamily G member 4	-0.952	0.992	0.008
198442873	CDC14A	cell division cycle 14A	-0.936	0.997	0.003
62945330	SLC8B1	solute carrier family 8 member B1	-0.929	0.993	0.007
50657416	C1RL	complement C1r subcomponent like	-0.918	0.990	0.010
157819487	TACO1	translational activator of cytochrome c oxidase I	-0.917	0.953	0.047
564325648	Zfp54	zinc finger protein 54	-0.907	0.954	0.046
564318923	WDR17	WD repeat domain 17	-0.904	0.983	0.017
14861868	Ptprv	protein tyrosine phosphatase, receptor type, V	-0.893	0.958	0.042
392351290	DNAH9	dynein axonemal heavy chain 9	-0.875	0.969	0.031
209870105	GPR37L1	G protein-coupled receptor 37 like 1	-0.870	0.993	0.007
157817185	PPCDC	phosphopantothenoylcysteine decarboxylase	-0.866	0.977	0.023
57114338	SCN4B	sodium voltage-gated channel beta subunit 4	-0.859	0.985	0.015
149031998	ACVRL1	activin A receptor like type 1	-0.852	0.988	0.012
6980958	SLC2A4	solute carrier family 2 member 4	-0.837	0.977	0.023
56605720	GADD45B	growth arrest and DNA damage inducible beta	-0.828	0.991	0.009
6980992	GSTT2/GSTT2B	glutathione S-transferase theta 2 (gene/pseudogene)	-0.824	0.968	0.032
58865784	GPR157	G protein-coupled receptor 157	-0.812	0.979	0.021
157817065	KCNK16	potassium two pore domain channel subfamily K member 16	-0.807	0.955	0.045
259089426	AGER	advanced glycosylation end-product specific receptor	-0.804	0.979	0.021
564345556	CROT	carnitine O-octanoyltransferase	-0.790	0.970	0.030
300798165	ZBTB40	zinc finger and BTB domain containing 40	-0.788	0.986	0.014
18426812	ADA	adenosine deaminase	-0.787	0.961	0.039
307548437	NYAP2	neuronal tyrosine-phosphorylated phosphoinositide-3-kinase adaptor 2	-0.780	0.981	0.019
942523340	CAPRIN2	caprin family member 2	-0.774	0.988	0.012
157819205	EFHC2	EF-hand domain containing 2	-0.772	0.993	0.007
61097937	VEGFB	vascular endothelial growth factor B	-0.766	0.966	0.034
57528269	ABHD14A	abhydrolase domain containing 14A	-0.760	0.953	0.047
27465577	Cyp4f16/Cyp4f37	cytochrome P450, family 4, subfamily f, polypeptide 16	-0.758	0.959	0.041
13591914	ANPEP	alanyl aminopeptidase, membrane	-0.757	0.977	0.023
189011669	FERMT3	fermitin family member 3	-0.751	0.970	0.030
672069802	C1QTNF1	C1q and TNF related 1	-0.749	0.984	0.016
157786850	TUBD1	tubulin delta 1	-0.740	0.982	0.018

208973286	RBM46	RNA binding motif protein 46	-0.724	0.963	0.037
19173756	ERG	ETS transcription factor ERG	-0.718	0.997	0.003
58219500	SLC10A7	solute carrier family 10 member 7	-0.716	0.951	0.049
149047075	Spaca6	sperm acrosome associated 6	-0.715	0.987	0.013
68341959	CASTOR1	cytosolic arginine sensor for mTORC1 subunit 1	-0.712	0.956	0.044
564378828	TFR2	transferrin receptor 2	-0.707	0.975	0.025
55742713	ECM1	extracellular matrix protein 1	-0.699	0.960	0.040
157816997	BDH2	3-hydroxybutyrate dehydrogenase 2	-0.698	0.959	0.041
564320335	TMEM241	transmembrane protein 241	-0.675	0.965	0.035
74142284	DSE	dermatan sulfate epimerase	-0.667	0.996	0.004
157818909	Zim1	zinc finger, imprinted 1	-0.665	0.980	0.020
67514566	POLA2	DNA polymerase alpha 2, accessory subunit	-0.665	0.960	0.040
672057459	DGKA	diacylglycerol kinase alpha	-0.664	0.989	0.011
157823283	Coch	cochlin	-0.653	0.981	0.019
77020281	CD55	CD55 molecule (Cromer blood group)	-0.648	0.991	0.009
672039742	TKFC	triokinase and FMN cyclase	-0.646	0.985	0.015
68534736	ERAP1	endoplasmic reticulum aminopeptidase 1	-0.646	0.984	0.016
13786136	PDGFC	platelet derived growth factor C	-0.642	0.963	0.037
149061352	ADAM12	ADAM metallopeptidase domain 12	-0.638	0.994	0.006
50657355	TOP1MT	DNA topoisomerase I mitochondrial	-0.632	0.963	0.037
34734058	HCK	HCK proto-oncogene, Src family tyrosine kinase	-0.631	0.964	0.036
397529557	C8orf58	chromosome 8 open reading frame 58	-0.629	0.994	0.006
157824208	NTNG1	netrin G1	-0.628	0.978	0.022
58865854	SCRN2	secernin 2	-0.627	0.980	0.020
16758622	IFT172	intraflagellar transport 172	-0.627	0.999	0.001
564395215	LOC100909409 (includes others)	RGD1562660	-0.626	0.959	0.041
157786914	OGFOD2	2-oxoglutarate and iron dependent oxygenase domain containing 2	-0.618	0.969	0.031
478732983	MAP3K5	mitogen-activated protein kinase kinase kinase 5	-0.616	0.953	0.047
402478640	HTRA3	HtrA serine peptidase 3	-0.608	0.975	0.025
157819065	ADAMTS15	ADAM metallopeptidase with thrombospondin type 1 motif 15	-0.604	0.997	0.003
157818491	DUS2	dihydrouridine synthase 2	-0.602	0.990	0.010
27465529	SLC9A4	solute carrier family 9 member A4	-0.601	0.993	0.007
56605940	RXFP3	relaxin family peptide receptor 3	-0.599	0.985	0.015
293347888	SRBD1	S1 RNA binding domain 1	-0.598	0.993	0.007
108935976	DISC1	DISC1 scaffold protein	-0.590	0.973	0.027
567316103	Ac1576	uncharacterized LOC102552783	-0.587	0.956	0.044

157822207	HOGA1	4-hydroxy-2-oxoglutarate aldolase 1	-0.587	0.964	0.036
197927123	LYRM7	LYR motif containing 7	-0.587	0.994	0.006
300793858	PARP14	poly(ADP-ribose) polymerase family member 14	-0.585	0.962	0.038
568950242	Pgap2	post-GPI attachment to proteins 2	-0.583	0.961	0.039
564394999	CLGN	calmegin	-0.582	1.000	0.000
564300485	LOC102551095	uncharacterized LOC102551095	-0.576	0.982	0.018
16758560	WIF1	WNT inhibitory factor 1	-0.573	0.990	0.010
564366772	MGC116197 (includes others)	similar to RIKEN cDNA 1700001E04	-0.569	0.958	0.042
55741859	XRCC4	X-ray repair cross complementing 4	-0.569	0.951	0.049
157816941	PLXDC1	plexin domain containing 1	-0.567	0.984	0.016
62078799	QRSL1	glutaminyl-tRNA amidotransferase subunit QRSL1	-0.566	0.993	0.007
57012436	Krt10	keratin 10	-0.562	0.965	0.035
75832150	GALNT3	polypeptide N-acetylgalactosaminyltransferase 3	-0.557	0.973	0.027
213385268	Gm10778	predicted gene 10778	-0.554	0.979	0.021
300798350	LRRK1	leucine rich repeat kinase 1	-0.554	0.951	0.049
199562000	USP40	ubiquitin specific peptidase 40	-0.552	0.966	0.034
171846640	FBLN1	fibulin 1	-0.543	0.973	0.027
672066171	GIN1	gypsy retrotransposon integrase 1	-0.539	0.964	0.036
148683700	TMEM98	transmembrane protein 98	-0.533	0.959	0.041
56090301	NUDT5	nudix hydrolase 5	-0.526	0.969	0.031
148747464	SCD	stearoyl-CoA desaturase	-0.524	0.978	0.022
19424232	CSF2RB	colony stimulating factor 2 receptor subunit beta	-0.524	0.983	0.017
157822555	RIN3	Ras and Rab interactor 3	-0.520	0.975	0.025
568966731	HMG20B	high mobility group 20B	-0.518	0.977	0.023
6981180	MAOB	monoamine oxidase B	-0.518	0.992	0.008
148692356	ARHGEF1	Rho guanine nucleotide exchange factor 1	-0.517	0.988	0.012
149037033	PRDM5	PR/SET domain 5	-0.516	0.977	0.023
149047863	LOC690190	hypothetical protein LOC690190	-0.514	0.996	0.004
147900684	TLR7	toll like receptor 7	-0.511	0.998	0.002
62078773	CCDC81	coiled-coil domain containing 81	-0.511	0.974	0.026
308044487	KIAA0319	KIAA0319	-0.509	0.954	0.046
148698795	GPX7	glutathione peroxidase 7	-0.507	0.969	0.031
654824082	Fbxl21	F-box and leucine-rich repeat protein 21	-0.500	1.000	0.000
50233928	TMEM159	transmembrane protein 159	-0.497	0.996	0.004
564330609	SYT17	synaptotagmin 17	-0.494	0.961	0.039
399220341	SLC2A13	solute carrier family 2 member 13	-0.492	0.983	0.017
157819347	CDC6	cell division cycle 6	-0.492	0.990	0.010

558611343	MCM3	minichromosome maintenance complex component 3	-0.488	0.964	0.036
6978867	GABRB1	gamma-aminobutyric acid type A receptor subunit beta1	-0.487	0.984	0.016
395759219	AQP4	aquaporin 4	-0.482	0.986	0.014
157073947	C1orf74	chromosome 1 open reading frame 74	-0.478	0.960	0.040
189011606	NCEH1	neutral cholesterol ester hydrolase 1	-0.476	0.998	0.002
202070751	RFTN1	raftlin, lipid raft linker 1	-0.474	0.951	0.049
157823399	COG4	component of oligomeric golgi complex 4	-0.468	0.982	0.018
41056215	XRCC5	X-ray repair cross complementing 5	-0.466	0.975	0.025
672044191	TBCK	TBC1 domain containing kinase	-0.465	0.974	0.026
148356229	CCND1	cyclin D1	-0.464	0.977	0.023
56090632	DMAC2L	distal membrane arm assembly complex 2 like	-0.463	0.986	0.014
148669431	DNAJC27	DnaJ heat shock protein family (Hsp40) member C27	-0.459	0.984	0.016
68163403	SLC46A3	solute carrier family 46 member 3	-0.458	0.951	0.049
56090411	POLE3	DNA polymerase epsilon 3, accessory subunit	-0.456	0.993	0.007
157817743	CDH5	cadherin 5	-0.454	0.978	0.022
9437326	SLC4A4	solute carrier family 4 member 4	-0.453	0.952	0.048
157817911	C21orf62	chromosome 21 open reading frame 62	-0.451	0.959	0.041
33414515	PXK	PX domain containing serine/threonine kinase like	-0.450	0.970	0.030
58865380	STAT2	signal transducer and activator of transcription 2	-0.450	0.997	0.003
8393807	MYH7	myosin heavy chain 7	-0.447	0.953	0.047
77993368	ACSF2	acyl-CoA synthetase family member 2	-0.444	0.965	0.035
51854227	GSN	gelsolin	-0.444	0.952	0.048
392331978	CDR2L	cerebellar degeneration related protein 2 like	-0.442	0.994	0.006
157786756	CDC45	cell division cycle 45	-0.442	0.955	0.045
148686921	SLC24A4	solute carrier family 24 member 4	-0.435	0.970	0.030
404312655	SDR42E1	short chain dehydrogenase/reductase family 42E, member 1	-0.435	0.986	0.014
61889119	TNFSF12	TNF superfamily member 12	-0.432	0.974	0.026
210031518	MOGAT2	monoacylglycerol O-acyltransferase 2	-0.432	0.950	0.050
157823259	TMEM229A	transmembrane protein 229A	-0.424	0.988	0.012
300796997	ARHGAP28	Rho GTPase activating protein 28	-0.423	0.951	0.049
210032365	HSP90B1	heat shock protein 90 beta family member 1	-0.421	0.987	0.013

56090564	GALM	galactose mutarotase	-0.420	0.988	0.012
13994179	SLC24A2	solute carrier family 24 member 2	-0.416	0.979	0.021
62945312	CXCL16	C-X-C motif chemokine ligand 16	-0.414	0.988	0.012
194473652	TTC38	tetratricopeptide repeat domain 38	-0.413	0.963	0.037
50811823	NENF	neudesin neurotrophic factor	-0.413	0.969	0.031
9506405	ARPC1B	actin related protein 2/3 complex subunit 1B	-0.410	0.957	0.043
13591949	GATM	glycine amidinotransferase	-0.409	0.994	0.006
672061813	ACSBG1	acyl-CoA synthetase bubblegum family member 1	-0.409	0.985	0.015
109480433	GNPTAB	N-acetylglucosamine-1-phosphate transferase subunits alpha and beta	-0.407	0.974	0.026
13162347	FDXR	ferredoxin reductase	-0.405	0.997	0.003
62079139	C11orf54	chromosome 11 open reading frame 54	-0.403	0.950	0.050
122065191	ABAT	4-aminobutyrate aminotransferase	-0.401	0.974	0.026
58865466	SLC37A1	solute carrier family 37 member 1	-0.401	0.952	0.048
149041576	REXO2	RNA exonuclease 2	-0.398	0.974	0.026
148666792	ARHGAP25	Rho GTPase activating protein 25	-0.396	0.997	0.003
78187977	TCF19	transcription factor 19	-0.393	0.992	0.008
77917572	LIPA	lipase A, lysosomal acid type	-0.392	0.969	0.031
149052857	KCNJ12	potassium inwardly rectifying channel subfamily J member 12	-0.392	0.959	0.041
157820327	THSD1	thrombospondin type 1 domain containing 1	-0.391	0.991	0.009
187937124	TMEM126B	transmembrane protein 126B	-0.389	0.956	0.044
51948488	SIRT5	sirtuin 5	-0.388	0.978	0.022
296040479	TXNRD3	thioredoxin reductase 3	-0.387	0.966	0.034
149034469	GNG7	G protein subunit gamma 7	-0.381	0.969	0.031
149036529	DGUOK	deoxyguanosine kinase	-0.381	0.988	0.012
40786487	GPR108	G protein-coupled receptor 108	-0.379	0.968	0.032
929981595	NPHP1	nephrocystin 1	-0.372	0.979	0.021
564299653	FAM169A	family with sequence similarity 169 member A	-0.372	0.987	0.013
113061	CHRNA3	cholinergic receptor nicotinic alpha 3 subunit	-0.372	0.965	0.035
198386343	TRPS1	transcriptional repressor GATA binding 1	-0.370	0.984	0.016
8393861	HPCAL4	hippocalcin like 4	-0.370	0.988	0.012
158303308	PCCA	propionyl-CoA carboxylase subunit alpha	-0.369	0.973	0.027
564343748	CDK5RAP1	CDK5 regulatory subunit associated protein 1	-0.368	0.987	0.013
564357619	ITGB8	integrin subunit beta 8	-0.366	0.961	0.039

158534064	RET	ret proto-oncogene	-0.365	0.975	0.025
16758712	PDIA4	protein disulfide isomerase family A member 4	-0.362	0.994	0.006
55741549	MRPL13	mitochondrial ribosomal protein L13	-0.360	0.974	0.026
40786491	CYP20A1	cytochrome P450 family 20 subfamily A member 1	-0.358	0.964	0.036
58865802	SPAG1	sperm associated antigen 1	-0.357	0.956	0.044
149066868	MDM1	Mdm1 nuclear protein	-0.357	0.980	0.020
157822187	WWOX	WW domain containing oxidoreductase	-0.355	0.956	0.044
672052705	FRRS1L	ferric chelate reductase 1 like	-0.354	0.985	0.015
11693172	CALR	calreticulin	-0.354	0.983	0.017
16758024	SYT9	synaptotagmin 9	-0.351	0.956	0.044
149060100	AIFM1	apoptosis inducing factor mitochondria associated 1	-0.351	0.982	0.018
451172073	CHRM3	cholinergic receptor muscarinic 3	-0.349	0.950	0.050
62078847	TSEN2	tRNA splicing endonuclease subunit 2	-0.343	0.988	0.012
77157795	MAL2	mal, T cell differentiation protein 2 (gene/pseudogene)	-0.342	0.994	0.006
157820807	GCDH	glutaryl-CoA dehydrogenase	-0.339	0.978	0.022
11693162	INSIG1	insulin induced gene 1	-0.339	0.993	0.007
56605710	LTBR	lymphotoxin beta receptor	-0.338	0.985	0.015
57527061	ZGPAT	zinc finger CCCH-type and G-patch domain containing	-0.335	0.958	0.042
148683194	INTS3	integrator complex subunit 3	-0.335	0.977	0.023
672085480	MEAK7	MTOR associated protein, eak-7 homolog	-0.334	0.968	0.032
13786174	TIMELESS	timeless circadian regulator	-0.332	0.971	0.029
300794275	MFSD10	major facilitator superfamily domain containing 10	-0.330	0.985	0.015
672038615	GSG1L	GSG1 like	-0.330	0.974	0.026
564309649	CCDC159	coiled-coil domain containing 159	-0.329	0.971	0.029
22024392	KIF1C	kinesin family member 1C	-0.328	0.968	0.032
281604125	Fam50a/LOC100910130	family with sequence similarity 50, member A	-0.327	0.950	0.050
564344373	ZMYND8	zinc finger MYND-type containing 8	-0.325	0.962	0.038
149045696	Ccl27a	chemokine (C-C motif) ligand 27A	-0.325	0.973	0.027
51036680	SLC29A3	solute carrier family 29 member 3	-0.322	0.977	0.023
451172111	HINT3	histidine triad nucleotide binding protein 3	-0.321	0.968	0.032
58865398	LAP3	leucine aminopeptidase 3	-0.320	0.973	0.027
6678297	TEX261	testis expressed 261	-0.318	0.965	0.035
51948466	TMED3	transmembrane p24 trafficking protein 3	-0.316	0.970	0.030

40018538	ADI1	acireductone dioxygenase 1	-0.316	0.988	0.012
57528352	DMAC2	distal membrane arm assembly complex 2	-0.315	0.956	0.044
149041432	THY1	Thy-1 cell surface antigen	-0.313	0.991	0.009
157786608	MRPL55	mitochondrial ribosomal protein L55	-0.313	0.972	0.028
564336403	EXOSC8	exosome component 8	-0.309	0.971	0.029
149063353	IFT81	intraflagellar transport 81	-0.309	0.973	0.027
167860097	FN3KRP	fructosamine 3 kinase related protein	-0.308	0.977	0.023
61556910	SNX10	sorting nexin 10	-0.306	0.957	0.043
564372688	RPA1	replication protein A1	-0.305	0.953	0.047
31982028	RSU1	Ras suppressor protein 1	-0.304	0.952	0.048
300796069	THADA	THADA armadillo repeat containing	-0.303	0.970	0.030
198041989	PARVB	parvin beta	-0.302	0.962	0.038
13540624	GRK5	G protein-coupled receptor kinase 5	-0.302	0.966	0.034
157820737	NUSAP1	nucleolar and spindle associated protein 1	-0.298	0.969	0.031
38259192	TOP2A	DNA topoisomerase II alpha	-0.297	0.960	0.040
6978888	GFRA1	GDNF family receptor alpha 1	-0.294	0.954	0.046
564358911	CHPT1	choline phosphotransferase 1	-0.292	0.997	0.003
157817979	Egfem1	EGF-like and EMI domain containing 1	-0.291	0.999	0.001
74223968	5031425E22Rik	RIKEN cDNA 5031425E22 gene	-0.290	0.960	0.040
149057745	NEK3	NIMA related kinase 3	-0.290	0.961	0.039
149063995	GMPR2	guanosine monophosphate reductase 2	-0.289	0.958	0.042
672083256	MYO5B	myosin VB	-0.287	0.953	0.047
564387894	BTD	biotinidase	-0.287	0.977	0.023
157820833	HERC3	HECT and RLD domain containing E3 ubiquitin protein ligase 3	-0.287	0.977	0.023
6981208	NR3C2	nuclear receptor subfamily 3 group C member 2	-0.286	0.956	0.044
76559913	COG7	component of oligomeric golgi complex 7	-0.283	0.972	0.028
149034870	RNF6	ring finger protein 6	-0.283	0.971	0.029
142349612	GLUL	glutamate-ammonia ligase	-0.279	0.972	0.028
564364473	RNF111	ring finger protein 111	-0.276	0.991	0.009
62078997	WDR1	WD repeat domain 1	-0.273	0.965	0.035
149022319	AGPS	alkylglycerone phosphate synthase	-0.270	0.989	0.011
157819337	SLC35B4	solute carrier family 35 member B4	-0.270	0.950	0.050
57164113	NSDHL	NAD(P) dependent steroid dehydrogenase-like	-0.269	0.979	0.021
576796148	MAP7D2	MAP7 domain containing 2	-0.269	0.955	0.045
74354506	ACBD5	acyl-CoA binding domain containing 5	-0.269	0.993	0.007

197313676	AIG1	androgen induced 1	-0.268	0.959	0.041
392339847	CADPS2	calcium dependent secretion activator 2	-0.268	0.961	0.039
38181552	SCG2	secretogranin II	-0.267	0.960	0.040
157819753	RCN1	reticulocalbin 1	-0.266	0.995	0.005
564382316	HSD11B1	hydroxysteroid 11-beta dehydrogenase 1	-0.265	0.987	0.013
148372343	RAMP2	receptor activity modifying protein 2	-0.264	0.974	0.026
564382292	ANGEL2	angel homolog 2	-0.258	0.994	0.006
17865325	GLRB	glycine receptor beta	-0.256	0.992	0.008
672041250	ARHGEF28	Rho guanine nucleotide exchange factor 28	-0.253	0.984	0.016
149046389	ARID5A	AT-rich interaction domain 5A	-0.249	0.962	0.038
149057830	Hgsnat	heparan-alpha-glucosaminide N-acetyltransferase	-0.248	0.951	0.049
157819077	TRIM37	tripartite motif containing 37	-0.246	0.991	0.009
15805026	ZFAND6	zinc finger AN1-type containing 6	-0.245	0.964	0.036
6978631	CD4	CD4 molecule	-0.245	0.995	0.005
290563168	DUSP3	dual specificity phosphatase 3	-0.243	0.967	0.033
57527332	PSPH	phosphoserine phosphatase	-0.241	0.977	0.023
58865958	RDH11	retinol dehydrogenase 11	-0.240	0.991	0.009
57192	P3H4	prolyl 3-hydroxylase family member 4 (inactive)	-0.240	0.994	0.006
157817480	RWDD2A	RWD domain containing 2A	-0.235	0.958	0.042
77404265	JAM2	junctional adhesion molecule 2	-0.233	0.977	0.023
584277046	SLC1A3	solute carrier family 1 member 3	-0.232	0.989	0.011
149053021	TMEM107	transmembrane protein 107	-0.232	0.965	0.035
293346766	TCAF1	TRPM8 channel associated factor 1	-0.232	0.985	0.015
9457244	RBBP9	RB binding protein 9, serine hydrolase	-0.231	0.999	0.001
13489067	NSF	N-ethylmaleimide sensitive factor, vesicle fusing ATPase	-0.231	0.966	0.034
157820653	TMEM63C	transmembrane protein 63C	-0.229	0.953	0.047
564397761	GCC2	GRIP and coiled-coil domain containing 2	-0.229	0.973	0.027
58865718	HERC4	HECT and RLD domain containing E3 ubiquitin protein ligase 4	-0.228	0.988	0.012
149062310	BSCL2	BSCL2 lipid droplet biogenesis associated, seipin	-0.228	0.967	0.033
281485606	STT3B	STT3 oligosaccharyltransferase complex catalytic subunit B	-0.228	0.983	0.017
672050038	NDNF	neuron derived neurotrophic factor	-0.226	0.958	0.042
157817710	FER	FER tyrosine kinase	-0.225	0.984	0.016
293345175	DHX29	DExH-box helicase 29	-0.225	0.992	0.008

148697866	FAM3A	FAM3 metabolism regulating signaling molecule A	-0.222	0.955	0.045
157786994	C1orf21	chromosome 1 open reading frame 21	-0.222	0.982	0.018
78369663	SLC38A9	solute carrier family 38 member 9	-0.222	0.961	0.039
148689145	CPNE4	copine 4	-0.222	0.995	0.005
148695758	CAPRIN1	cell cycle associated protein 1	-0.221	0.971	0.029
157822873	FBH1	F-box DNA helicase 1	-0.219	0.961	0.039
564298436	WDR11	WD repeat domain 11	-0.216	0.952	0.048
6978890	GGH	gamma-glutamyl hydrolase	-0.215	0.969	0.031
8393643	KCNAB1	potassium voltage-gated channel subfamily A member regulatory beta subunit 1	-0.214	0.974	0.026
57526927	LARS1	leucyl-tRNA synthetase 1	-0.213	0.978	0.022
672066638	CLEC16A	C-type lectin domain containing 16A	-0.212	0.972	0.028
18959250	PRKCD	protein kinase C delta	-0.210	0.979	0.021
149058126	ALDH9A1	aldehyde dehydrogenase 9 family member A1	-0.208	0.997	0.003
37359962	PLPPR4	phospholipid phosphatase related 4	-0.207	0.982	0.018
225543229	TIAM1	TIAM Rac1 associated GEF 1	-0.207	0.961	0.039
148667192	LRTM2	leucine rich repeats and transmembrane domains 2	-0.207	0.993	0.007
6981504	ATXN1	ataxin 1	-0.204	0.958	0.042
148747414	GDA	guanine deaminase	-0.203	0.967	0.033
564391231	SERPINB9	serpin family B member 9	-0.203	1.000	0.000
55741502	ACAT2	acetyl-CoA acetyltransferase 2	-0.202	0.995	0.005
48675867	PLPP3	phospholipid phosphatase 3	-0.201	0.996	0.004
149053315	CAMKK1	calcium/calmodulin dependent protein kinase kinase 1	-0.200	0.971	0.029
11560055	KHDRBS3	KH RNA binding domain containing, signal transduction associated 3	-0.195	0.976	0.024
564334053	SORCS1	sortilin related VPS10 domain containing receptor 1	-0.195	0.986	0.014
17530977	ECHS1	enoyl-CoA hydratase, short chain 1	-0.191	0.998	0.002
148696094	TUBGCP4	tubulin gamma complex associated protein 4	-0.189	0.991	0.009
564334013	GBF1	golgi brefeldin A resistant guanine nucleotide exchange factor 1	-0.187	0.955	0.045
157821901	PNMA3	PNMA family member 3	-0.186	0.958	0.042
672088942	ATP2B3	ATPase plasma membrane Ca2+ transporting 3	-0.186	0.990	0.010
312283667	WNK1	WNK lysine deficient protein kinase 1	-0.185	0.954	0.046
197209847	JAK1	Janus kinase 1	-0.185	0.998	0.002
59891444	FUT10	fucosyltransferase 10	-0.185	0.952	0.048

16923964	CNTN1	contactin 1	-0.185	0.995	0.005
76881830	Kcnip2	potassium voltage-gated channel interacting protein 2	-0.183	0.998	0.002
149036441	SUCLG1	succinate-CoA ligase GDP/ADP-forming subunit alpha	-0.181	0.966	0.034
32185285	BCL2L2	BCL2 like 2	-0.181	0.971	0.029
157817620	PSD2	pleckstrin and Sec7 domain containing 2	-0.180	0.959	0.041
12621120	SFXN3	sideroflexin 3	-0.180	0.959	0.041
187468990	DNAJB2	DnaJ heat shock protein family (Hsp40) member B2	-0.180	0.995	0.005
13929208	Scd2	stearoyl-Coenzyme A desaturase 2	-0.178	0.984	0.016
201023331	MAPK11	mitogen-activated protein kinase 11	-0.176	0.962	0.038
157818193	TTPAL	alpha tocopherol transfer protein like	-0.176	0.964	0.036
78000203	Tpm1	tropomyosin 1, alpha	-0.174	0.982	0.018
124248495	CHID1	chitinase domain containing 1	-0.171	0.959	0.041
61557212	CIAO3	cytosolic iron-sulfur assembly component 3	-0.170	0.994	0.006
19705545	RAB3IL1	RAB3A interacting protein like 1	-0.166	0.989	0.011
157817201	NETO1	neuropilin and tolloid like 1	-0.165	0.983	0.017
149031018	COX4I2	cytochrome c oxidase subunit 4I2	-0.165	0.961	0.039
19173766	LONP1	lon peptidase 1, mitochondrial	-0.164	0.979	0.021
198278547	TMEM41A	transmembrane protein 41A	-0.159	0.966	0.034
62078637	LCA5	lebercilin LCA5	-0.158	0.957	0.043
209529636	PPA2	inorganic pyrophosphatase 2	-0.158	0.984	0.016
18034785	ABCB6	ATP binding cassette subfamily B member 6 (Langereis blood group)	-0.158	0.992	0.008
19173802	PPP1R14C	protein phosphatase 1 regulatory inhibitor subunit 14C	-0.155	0.950	0.050
6649914	GDF11	growth differentiation factor 11	-0.154	0.978	0.022
71043650	SRPK1	SRSF protein kinase 1	-0.150	0.965	0.035
755498773	ITGA6	integrin subunit alpha 6	-0.149	0.951	0.049
113461996	COA5	cytochrome c oxidase assembly factor 5	-0.149	0.968	0.032
50510837	KIAA1191	KIAA1191	-0.148	0.990	0.010
6980956	GLUD1	glutamate dehydrogenase 1	-0.146	0.954	0.046
148671875	TMEM50B	transmembrane protein 50B	-0.142	0.958	0.042
201066369	LGI2	leucine rich repeat LGI family member 2	-0.139	0.981	0.019
77627979	SRPRA	SRP receptor subunit alpha	-0.138	0.956	0.044
157823401	PIGH	phosphatidylinositol glycan anchor biosynthesis class H	-0.135	0.988	0.012
300794317	SFI1	SFI1 centrin binding protein	-0.133	0.976	0.024
564345487	RINT1	RAD50 interactor 1	-0.132	0.971	0.029

56605798	RNF167	ring finger protein 167	-0.131	0.998	0.002
148747541	HNRNPU	heterogeneous nuclear ribonucleoprotein U	-0.130	0.997	0.003
568985444	CADPS	calcium dependent secretion activator	-0.129	0.979	0.021
398650648	SLC8A1	solute carrier family 8 member A1	-0.127	0.956	0.044
37359832	SCRN1	secernin 1	-0.127	0.967	0.033
77415383	HSPA8	heat shock protein family A (Hsp70) member 8	-0.125	0.959	0.041
58865700	GRWD1	glutamate rich WD repeat containing 1	-0.123	0.995	0.005
157820919	POLE4	DNA polymerase epsilon 4, accessory subunit	-0.123	0.957	0.043
253683488	NTRK2	neurotrophic receptor tyrosine kinase 2	-0.121	0.995	0.005
158254369	CDK10	cyclin dependent kinase 10	-0.121	0.963	0.037
74229032	TPCN1	two pore segment channel 1	-0.121	0.987	0.013
398303839	SH3GL2	SH3 domain containing GRB2 like 2, endophilin A1	-0.117	0.978	0.022
8393390	GABRB3	gamma-aminobutyric acid type A receptor subunit beta3	-0.115	0.997	0.003
149059529	LOC100910558	uncharacterized LOC100910558	-0.114	0.985	0.015
6978621	CCNG1	cyclin G1	-0.114	0.971	0.029
404247435	YLPM1	YLP motif containing 1	-0.113	0.986	0.014
56090463	GORASP2	golgi reassembly stacking protein 2	-0.113	0.982	0.018
564353678	USP48	ubiquitin specific peptidase 48	-0.112	0.951	0.049
157786602	NHP2	NHP2 ribonucleoprotein	-0.110	0.969	0.031
89337260	FTO	FTO alpha-ketoglutarate dependent dioxygenase	-0.107	0.961	0.039
274326692	UQCC3	ubiquinol-cytochrome c reductase complex assembly factor 3	-0.106	0.968	0.032
298231200	INSR	insulin receptor	-0.104	0.974	0.026
149036808	ARL6IP5	ADP ribosylation factor like GTPase 6 interacting protein 5	-0.099	0.955	0.045
403377905	SRGAP2	SLIT-ROBO Rho GTPase activating protein 2	-0.098	0.985	0.015
149060725	CEP19	centrosomal protein 19	-0.095	0.998	0.002
154800420	GNL3L	G protein nucleolar 3 like	-0.093	0.970	0.030
8980843	GRIPAP1	GRIP1 associated protein 1	-0.089	0.996	0.004
16758578	DPP3	dipeptidyl peptidase 3	-0.084	0.964	0.036
50054266	NLN	neurolysin	-0.082	0.957	0.043
564343911	RPN2	ribophorin II	-0.076	0.951	0.049
297206894	E4F1	E4F transcription factor 1	-0.074	0.988	0.012
564375060	SLC39A11	solute carrier family 39 member 11	-0.070	0.991	0.009
148747528	PTK2B	protein tyrosine kinase 2 beta	-0.066	0.983	0.017

255918181	NUS1	NUS1 dehydrodolichyl diphosphate synthase subunit	-0.062	0.985	0.015
20302113	STIP1	stress induced phosphoprotein 1	-0.060	0.958	0.042
408535187	PRDM11	PR/SET domain 11	-0.058	0.961	0.039
13385318	KDELR2	KDEL endoplasmic reticulum protein retention receptor 2	-0.057	0.993	0.007
162287208	FADS1	fatty acid desaturase 1	-0.050	0.993	0.007
157820825	IFT57	intraflagellar transport 57	0.053	-0.958	0.042
76443681	USP11	ubiquitin specific peptidase 11	0.053	-0.972	0.028
157822779	DNAJC11	DnaJ heat shock protein family (Hsp40) member C11	0.066	-0.950	0.050
62089200	ZDHHC9	zinc finger DHHC-type palmitoyltransferase 9	0.068	-0.957	0.043
149031779	PRR3	proline rich 3	0.072	-0.968	0.032
564353714	FBXO42	F-box protein 42	0.074	-0.955	0.045
157817783	SNX18	sorting nexin 18	0.079	-0.954	0.046
169790975	MRPS9	mitochondrial ribosomal protein S9	0.082	-0.967	0.033
83649695	SMIM14	small integral membrane protein 14	0.082	-0.999	0.001
60360532	OSBPL6	oxysterol binding protein like 6	0.083	-0.953	0.047
290560659	ZNF609	zinc finger protein 609	0.090	-0.980	0.020
149038024	RIPOR1	RHO family interacting cell polarization regulator 1	0.091	-0.990	0.010
564332984	OSBP	oxysterol binding protein	0.094	-0.986	0.014
189491673	FXR2	FMR1 autosomal homolog 2	0.097	-0.960	0.040
970596961	MAPK10	mitogen-activated protein kinase 10	0.099	-0.958	0.042
166064004	GTF3A	general transcription factor IIIA	0.101	-0.967	0.033
157818159	AAR2	AAR2 splicing factor	0.110	-0.989	0.011
157786720	HIVEP1	HIVEP zinc finger 1	0.111	-0.985	0.015
30794434	SRRM4	serine/arginine repetitive matrix 4	0.112	-0.959	0.041
157817811	C5orf22	chromosome 5 open reading frame 22	0.113	-0.953	0.047
170295834	NDUFA10	NADH:ubiquinone oxidoreductase subunit A10	0.114	-0.966	0.034
564360651	LRRC14	leucine rich repeat containing 14	0.115	-0.965	0.035
68163425	TMEM199	transmembrane protein 199	0.118	-0.988	0.012
293344794	FAM160B1	family with sequence similarity 160 member B1	0.121	-0.974	0.026
58866022	MGAT4A	alpha-1,3-mannosyl-glycoprotein 4-beta-N-acetylglucosaminyltransferase A	0.127	-0.971	0.029
454601639	NCOA6	nuclear receptor coactivator 6	0.127	-0.975	0.025
672068548	SUPT6H	SPT6 homolog, histone chaperone and transcription elongation factor	0.127	-0.970	0.030
564331450	EEF1AKMT2	EEF1A lysine methyltransferase 2	0.129	-0.982	0.018
564361462	BRD1	bromodomain containing 1	0.130	-0.951	0.049

60359978	KIF3C	kinesin family member 3C	0.131	-0.985	0.015
148693260	TIMM29	translocase of inner mitochondrial membrane 29	0.131	-0.992	0.008
58865952	UBAP1	ubiquitin associated protein 1	0.139	-0.974	0.026
74139306	TMED9	transmembrane p24 trafficking protein 9	0.143	-0.956	0.044
9624979	ENSA	endosulfine alpha	0.144	-0.991	0.009
672024670	INSYN2B	inhibitory synaptic factor family member 2B	0.145	-0.965	0.035
157820585	SART3	spliceosome associated factor 3, U4/U6 recycling protein	0.146	-0.998	0.002
149064388	Hmgxb3	HMG-box containing 3	0.147	-0.993	0.007
61556879	PKNOX1	PBX/knotted 1 homeobox 1	0.148	-0.959	0.041
157821581	PSMD13	proteasome 26S subunit, non-ATPase 13	0.150	-0.973	0.027
40786455	BPGM	bisphosphoglycerate mutase	0.150	-0.998	0.002
564311031	CLPP	caseinolytic mitochondrial matrix peptidase proteolytic subunit	0.150	-0.953	0.047
208973276	TMEM185A	transmembrane protein 185A	0.151	-0.960	0.040
300253233	LEMD3	LEM domain containing 3	0.154	-0.981	0.019
66730376	Arxes1/Arxes2	adipocyte-related X-chromosome expressed sequence 2	0.155	-0.960	0.040
77917548	DUS3L	dihydrouridine synthase 3 like	0.158	-0.972	0.028
300793740	TANC2	tetratricopeptide repeat, ankyrin repeat and coiled-coil containing 2	0.158	-0.987	0.013
404312665	DKK3	dickkopf WNT signaling pathway inhibitor 3	0.160	-0.956	0.044
149033480	Zfp956	zinc finger protein 956	0.163	-0.998	0.002
672072928	CUX2	cut like homeobox 2	0.164	-0.997	0.003
157822501	MCM3AP	minichromosome maintenance complex component 3 associated protein	0.164	-0.971	0.029
564340867	MMADHC	metabolism of cobalamin associated D	0.165	-0.958	0.042
76559929	NOC2L	NOC2 like nucleolar associated transcriptional repressor	0.165	-0.992	0.008
197385832	RD3L	retinal degeneration 3 like	0.167	-0.967	0.033
50511063	EEPD1	endonuclease/exonuclease/phosphatase family domain containing 1	0.170	-0.963	0.037
33356154	UBE2H	ubiquitin conjugating enzyme E2 H	0.172	-0.987	0.013
56605790	HCFC2	host cell factor C2	0.173	-0.996	0.004
149054120	ORMDL3	ORMDL sphingolipid biosynthesis regulator 3	0.179	-0.997	0.003
62079005	SLAIN1	SLAIN motif family member 1	0.180	-0.979	0.021

157822303	GPR107	G protein-coupled receptor 107	0.182	-0.965	0.035
157817773	ZNF641	zinc finger protein 641	0.183	-0.994	0.006
401709959	Ppp1cc	protein phosphatase 1 catalytic subunit gamma	0.183	-0.953	0.047
9507007	PTGFRN	prostaglandin F2 receptor inhibitor	0.184	-0.966	0.034
162287391	RPL6	ribosomal protein L6	0.185	-0.953	0.047
564384443	EIF4ENIF1	eukaryotic translation initiation factor 4E nuclear import factor 1	0.187	-0.961	0.039
66730335	SUMO3	small ubiquitin like modifier 3	0.188	-0.977	0.023
672085227	USP10	ubiquitin specific peptidase 10	0.191	-0.998	0.002
148683335	SLC25A44	solute carrier family 25 member 44	0.191	-0.984	0.016
197313795	MTX1	metaxin 1	0.192	-0.992	0.008
157823197	NDUFB7	NADH:ubiquinone oxidoreductase subunit B7	0.194	-0.965	0.035
157821953	NXPE3	neurexophilin and PC-esterase domain family member 3	0.197	-0.990	0.010
157820969	SBNO2	strawberry notch homolog 2	0.197	-0.959	0.041
57164107	NIPSNAP3A	nipsnap homolog 3A	0.199	-0.964	0.036
213511844	ALG2	ALG2 alpha-1,3/1,6-mannosyltransferase	0.200	-0.978	0.022
157821579	BICD1	BICD cargo adaptor 1	0.202	-0.966	0.034
281604227	CEP104	centrosomal protein 104	0.203	-0.974	0.026
219277692	NDUFB2	NADH:ubiquinone oxidoreductase subunit B2	0.203	-0.991	0.009
61557021	BFAR	bifunctional apoptosis regulator	0.204	-0.987	0.013
564383487	SLAIN2	SLAIN motif family member 2	0.206	-0.973	0.027
157817674	ATP5MF-PTCD1	ATP5MF-PTCD1 readthrough	0.207	-0.995	0.005
40018540	DDX24	DEAD-box helicase 24	0.208	-0.955	0.045
61556748	TSPYL1	TSPY like 1	0.211	-0.999	0.001
189163477	SCAF4	SR-related CTD associated factor 4	0.216	-0.996	0.004
396080328	ADCYAP1R1	ADCYAP receptor type I	0.217	-0.965	0.035
2804296	CDH8	cadherin 8	0.221	-0.991	0.009
148669751	SMNDC1	survival motor neuron domain containing 1	0.223	-0.997	0.003
293340917	C3orf70	chromosome 3 open reading frame 70	0.223	-0.956	0.044
148696931	ARRDC2	arrestin domain containing 2	0.227	-0.962	0.038
157818775	AFF1	AF4/FMR2 family member 1	0.228	-0.955	0.045
148670791	ZFYVE1	zinc finger FYVE-type containing 1	0.232	-0.986	0.014
798974764	SRRD	SRR1 domain containing	0.233	-0.972	0.028
149032040	SLC11A2	solute carrier family 11 member 2	0.234	-0.961	0.039
564340133	GTF3C4	general transcription factor IIIC subunit 4	0.236	-0.979	0.021
37360264	TRMT6	tRNA methyltransferase 6	0.236	-0.974	0.026

19424174	DNPH1	2'-deoxynucleoside 5'-phosphate N-hydrolase 1	0.243	-0.956	0.044
157821413	USP30	ubiquitin specific peptidase 30	0.250	-0.970	0.030
291042683	DCAF5	DDB1 and CUL4 associated factor 5	0.252	-0.959	0.041
166157540	TMEM222	transmembrane protein 222	0.253	-0.980	0.020
672052951	Zfp618	zinc finger protein 618	0.254	-0.955	0.045
40254779	EFNB1	ephrin B1	0.255	-0.957	0.043
77627757	IQUB	IQ motif and ubiquitin domain containing	0.257	-0.980	0.020
119388826	TFPT	TCF3 fusion partner	0.258	-0.986	0.014
281427178	CEP76	centrosomal protein 76	0.262	-0.989	0.011
67078454	SLC25A51	solute carrier family 25 member 51	0.263	-0.998	0.002
380877082	NAXE	NAD(P)HX epimerase	0.263	-0.986	0.014
148687213	COX19	cytochrome c oxidase assembly factor COX19	0.264	-0.982	0.018
564353880	DDI2	DNA damage inducible 1 homolog 2	0.264	-0.984	0.016
148696370	PANK2	pantothenate kinase 2	0.270	-0.955	0.045
564394925	TENT4B	terminal nucleotidyltransferase 4B	0.276	-0.992	0.008
197386048	PTRHD1	peptidyl-tRNA hydrolase domain containing 1	0.280	-0.954	0.046
149052738	RGD1561277	RGD1561277	0.285	-0.993	0.007
62078733	MAK16	MAK16 homolog	0.287	-0.997	0.003
672020915	VCPKMT	valosin containing protein lysine methyltransferase	0.288	-0.982	0.018
76559919	N4BP3	NEDD4 binding protein 3	0.292	-0.983	0.017
149031601	H1f2	H1.2 linker histone, cluster member	0.294	-0.961	0.039
68163385	GPATCH4	G-patch domain containing 4	0.297	-0.991	0.009
157821997	MED28	mediator complex subunit 28	0.297	-0.965	0.035
74220037	FAM107B	family with sequence similarity 107 member B	0.299	-0.970	0.030
157822519	CBLN4	cerebellin 4 precursor	0.299	-0.977	0.023
13929168	FAT1	FAT atypical cadherin 1	0.300	-0.967	0.033
157822367	PUS3	pseudouridine synthase 3	0.301	-0.952	0.048
157819365	TBC1D25	TBC1 domain family member 25	0.302	-0.955	0.045
157819315	OSBPL11	oxysterol binding protein like 11	0.306	-0.998	0.002
51491900	TOR1A	torsin family 1 member A	0.308	-0.998	0.002
50510821	AMIGO1	adhesion molecule with Ig like domain 1	0.310	-0.964	0.036
672043577	Rprd2	regulation of nuclear pre-mRNA domain containing 2	0.312	-0.955	0.045
300793780	ZNF251	zinc finger protein 251	0.321	-0.993	0.007
41386747	ZC3H18	zinc finger CCCH-type containing 18	0.322	-0.982	0.018
672030183	H2AC12	H2A clustered histone 12	0.323	-0.993	0.007

76362828	TEF	TEF transcription factor, PAR bZIP family member	0.327	-0.985	0.015
68163537	NXPE4	neurexophilin and PC-esterase domain family member 4	0.329	-0.974	0.026
157817260	LTO1	LTO1 maturation factor of ABCE1	0.330	-0.979	0.021
62244083	PDRG1	p53 and DNA damage regulated 1	0.331	-0.962	0.038
564333920	PPRC1	PPARG related coactivator 1	0.333	-0.999	0.001
156627555	NT5C3B	5'-nucleotidase, cytosolic IIIB	0.335	-0.983	0.017
564350836	MELK	maternal embryonic leucine zipper kinase	0.341	-0.952	0.048
141803183	ZKSCAN3	zinc finger with KRAB and SCAN domains 3	0.351	-0.980	0.020
40018598	ANGPTL4	angiopoietin like 4	0.356	-0.977	0.023
148673748	FAM110B	family with sequence similarity 110 member B	0.357	-0.985	0.015
57528321	RIOK2	RIO kinase 2	0.360	-0.975	0.025
157822027	CSRNP2	cysteine and serine rich nuclear protein 2	0.361	-0.989	0.011
300794219	OPN3	opsin 3	0.362	-0.983	0.017
300797828	KAT14	lysine acetyltransferase 14	0.365	-0.999	0.001
157821875	PTCD2	pentatricopeptide repeat domain 2	0.372	-0.966	0.034
672036551	ZDHHC13	zinc finger DHHC-type palmitoyltransferase 13	0.373	-0.979	0.021
157817720	SLC16A14	solute carrier family 16 member 14	0.374	-0.999	0.001
157821747	MDM2	MDM2 proto-oncogene	0.377	-0.960	0.040
164565364	ITPKB	inositol-trisphosphate 3-kinase B	0.380	-0.987	0.013
564382837	LIN54	lin-54 DREAM MuvB core complex component	0.387	-0.972	0.028
148681067	VASH2	vasohibin 2	0.397	-0.952	0.048
40789237	PCDHA4	protocadherin alpha 4	0.398	-0.978	0.022
157821403	RASSF7	Ras association domain family member 7	0.399	-0.991	0.009
157817446	LINGO2	leucine rich repeat and Ig domain containing 2	0.403	-0.976	0.024
282158061	Ttc41	tetratricopeptide repeat domain 41	0.404	-0.962	0.038
62945262	PIK3IP1	phosphoinositide-3-kinase interacting protein 1	0.404	-0.956	0.044
293345066	PPIL6	peptidylprolyl isomerase like 6	0.408	-0.988	0.012
66730347	PTPRCAP	protein tyrosine phosphatase receptor type C associated protein	0.415	-0.965	0.035
67846052	DCUN1D3	defective in cullin neddylation 1 domain containing 3	0.416	-0.970	0.030
56090305	NFATC2IP	nuclear factor of activated T cells 2 interacting protein	0.430	-0.977	0.023

564395313	OTUD4	OTU deubiquitinase 4	0.434	-0.967	0.033
157823891	ING2	inhibitor of growth family member 2	0.439	-0.957	0.043
564311452	TMEM131	transmembrane protein 131	0.443	-0.991	0.009
51980294	COQ3	coenzyme Q3, methyltransferase	0.452	-0.956	0.044
53850630	LOC100362724/ MGC95208	similar to 4930453N24Rik protein	0.456	-0.999	0.001
8392993	BMP3	bone morphogenetic protein 3	0.461	-0.956	0.044
148706598	PKDCC	protein kinase domain containing, cytoplasmic	0.469	-0.996	0.004
51948492	NUDT19	nudix hydrolase 19	0.470	-1.000	0.000
70912374	CCNQ	cyclin Q	0.479	-0.976	0.024
19424300	GCHFR	GTP cyclohydrolase I feedback regulator	0.519	-0.962	0.038
89145411	SULT2B1	sulfotransferase family 2B member 1	0.524	-0.981	0.019
148687591	TMEM132D	transmembrane protein 132D	0.550	-0.983	0.017
213688370	EXOSC7	exosome component 7	0.551	-0.989	0.011
219879771	PGAP3	post-GPI attachment to proteins phospholipase 3	0.560	-0.996	0.004
149056609	DEDD2	death effector domain containing 2	0.565	-0.967	0.033
149023178	CEP152	centrosomal protein 152	0.572	-0.988	0.012
564372912	GPS2	G protein pathway suppressor 2	0.577	-0.956	0.044
148664537	Gm10269	ribosomal protein L35 pseudogene	0.582	-0.994	0.006
555290059	MED7	mediator complex subunit 7	0.584	-0.972	0.028
511094004	RUNX2	RUNX family transcription factor 2	0.585	-0.990	0.010
24415396	GPR3	G protein-coupled receptor 3	0.604	-0.990	0.010
21703842	RTCB	RNA 2',3'-cyclic phosphate and 5'-OH ligase	0.609	-0.962	0.038
22122541	LRRC3B	leucine rich repeat containing 3B	0.615	-0.954	0.046
157822359	PELI2	pellino E3 ubiquitin protein ligase family member 2	0.620	-0.988	0.012
1083798	Bmpr1b	bone morphogenetic protein receptor type 1B	0.630	-0.962	0.038
197386066	ZNF784	zinc finger protein 784	0.633	-0.994	0.006
157823803	DOK3	docking protein 3	0.639	-0.988	0.012
348041347	CENPL	centromere protein L	0.708	-0.983	0.017
392354293	Hmgb3	high mobility group box 3	0.709	-0.994	0.006
80861398	CRY1	cryptochrome circadian regulator 1	0.720	-0.958	0.042
293339965	RAB11FIP3	RAB11 family interacting protein 3	0.750	-0.962	0.038
564377118	WDR53	WD repeat domain 53	0.807	-0.967	0.033
294979130	FOXP3	forkhead box P3	0.830	-0.951	0.049
672086719	FAM184A	family with sequence similarity 184 member A	0.836	-0.997	0.003
189011634	ARMC7	armadillo repeat containing 7	0.850	-0.963	0.037
672025117	MBTD1	mbt domain containing 1	0.886	-0.964	0.036

392339806	CFAP69	cilia and flagella associated protein 69	0.915	-0.986	0.014
9506775	HES2	hes family bHLH transcription factor 2	0.963	-0.990	0.010
148693657	DDX6	DEAD-box helicase 6	0.969	-0.991	0.009
82654234	LILRA6	leukocyte immunoglobulin like receptor A6	1.000	-0.999	0.001
148235584	CLEC4A	C-type lectin domain family 4 member A	1.000	-0.990	0.010
149065466	ARHGEF5	Rho guanine nucleotide exchange factor 5	1.000	-0.990	0.010
404501522	NXNL1	nucleoredoxin like 1	1.037	-0.990	0.010
148674299	Gm14176	ubiquitin-conjugating enzyme E2I pseudogene	1.041	-0.969	0.031
149067796	TMEM219	transmembrane protein 219	1.064	-0.978	0.022
19424314	KCNE2	potassium voltage-gated channel subfamily E regulatory subunit 2	1.066	-0.981	0.019
564296988	ZNF235	zinc finger protein 235	1.072	-0.992	0.008
188536090	FAM241B	family with sequence similarity 241 member B	1.127	-1.000	0.000
62078917	PAQR5	progestin and adipoQ receptor family member 5	1.181	-0.990	0.010
6978493	ALOX5	arachidonate 5-lipoxygenase	1.193	-0.952	0.048
564317923	SACS	sacsin molecular chaperone	1.308	-0.962	0.038
392338392	PCNT	pericentrin	1.342	-0.960	0.040
157817264	ANKRD23	ankyrin repeat domain 23	1.505	-0.972	0.028
158533972	SPTA1	spectrin alpha, erythrocytic 1	1.585	-0.990	0.010
149058209	SELE	selectin E	1.585	-0.990	0.010
13928980	AQP3	aquaporin 3 (Gill blood group)	1.585	-0.978	0.022
58865680	CES5A	carboxylesterase 5A	1.585	-0.963	0.037
24308466	ITGB3	integrin subunit beta 3	1.596	-0.965	0.035
157818463	Zfp93	zinc finger protein 93	1.597	-0.980	0.020
672013187	DMWD	DM1 locus, WD repeat containing	1.605	-0.982	0.018
41054896	FUT7	fucosyltransferase 7	1.700	-0.964	0.036
568990288	NIPBL	NIPBL cohesin loading factor	1.705	-0.991	0.009
569001477	MTCL1	microtubule crosslinking factor 1	1.726	-0.976	0.024
149032888	LOC100910237	uncharacterized LOC100910237	1.751	-0.999	0.001
157820135	CHRDL2	chordin like 2	1.762	-0.999	0.001
157821823	Ngp	neutrophilic granule protein	1.807	-0.959	0.041
564318930	WDR17	WD repeat domain 17	1.807	-0.959	0.041
392342449	PRSS56	serine protease 56	1.848	-0.963	0.037
149042883	LOC100365365	rCG32328-like	1.861	-0.965	0.035
189181736	LAD1	ladinin 1	1.874	-0.980	0.020
16758254	CNGA1	cyclic nucleotide gated channel subunit alpha 1	1.874	-0.962	0.038

197381585	Urah	urate (5-hydroxyiso-) hydrolase	1.976	-0.990	0.010
160961485	MYLK3	myosin light chain kinase 3	2.000	-0.990	0.010
568979594	SYT16	synaptotagmin 16	2.059	-0.953	0.047
672070295	BAHCC1	BAH domain and coiled-coil containing 1	2.083	-0.964	0.036
28972866	CSMD3	CUB and Sushi multiple domains 3	2.140	-0.998	0.002
564297852	CRTC3	CREB regulated transcription coactivator 3	2.151	-0.951	0.049
293352381	PAN3	poly(A) specific ribonuclease subunit PAN3	2.239	-0.979	0.021
71896592	IGFALS	insulin like growth factor binding protein acid labile subunit	2.322	-0.990	0.010
8393941	PADI4	peptidyl arginine deiminase 4	2.322	-0.990	0.010
157818163	POF1B	POF1B actin binding protein	2.392	-0.976	0.024
157819659	RRH	retinal pigment epithelium-derived rhodopsin homolog	2.447	-0.971	0.029
564329376	SRPK3	SRSF protein kinase 3	2.585	-0.990	0.010
58866038	XKRX	XK related X-linked	2.585	-0.990	0.010
11120690	NR1H4	nuclear receptor subfamily 1 group H member 4	2.585	-0.990	0.010
13540693	MYOC	myocilin	2.585	-0.990	0.010
57222314	OAS3	2'-5'-oligoadenylate synthetase 3	2.585	-0.990	0.010
8394529	VDR	vitamin D receptor	2.585	-0.990	0.010
25742760	AMH	anti-Mullerian hormone	2.807	-0.990	0.010
157787002	Dpt	dermatopontin	2.807	-0.990	0.010
197384923	C1orf87	chromosome 1 open reading frame 87	3.000	-0.990	0.010
13591993	MMP9	matrix metallopeptidase 9	3.000	-0.990	0.010
300796937	ESPNL	espin like	3.000	-0.990	0.010
564347547	LOC103690120	probable N-acetyltransferase CML1	3.030	-0.991	0.009
281332212	SH2D4B	SH2 domain containing 4B	3.170	-0.990	0.010
260099641	MSH5	mutS homolog 5	3.170	-0.990	0.010
564392795	MOCOS	molybdenum cofactor sulfurase	3.248	-0.967	0.033
61556961	THEG	theg spermatid protein	3.322	-0.990	0.010
28174920	RPL17	ribosomal protein L17	3.389	-0.998	0.002
25282405	BPIFA1	BPI fold containing family A member 1	3.459	-0.990	0.010
16758550	BCL2L10	BCL2 like 10	3.459	-0.990	0.010
148670929	BATF	basic leucine zipper ATF-like transcription factor	3.700	-0.990	0.010
21245088	Ly6a (includes others)	lymphocyte antigen 6 complex, locus A	3.807	-0.990	0.010
148747510	BAAT	bile acid-CoA:amino acid N-acyltransferase	3.807	-0.990	0.010
158187515	OAZ3	ornithine decarboxylase antizyme 3	3.807	-0.990	0.010

672052120	RBM12B	RNA binding motif protein 12B	3.907	-0.955	0.045
8393641	AADAT	aminoadipate aminotransferase	4.248	-0.990	0.010
27545443	CEACAM4	CEA cell adhesion molecule 4	4.392	-0.984	0.016
149038931	CNTRL	centriolin	4.492	-0.989	0.011
341940965	MOS	MOS proto-oncogene, serine/threonine kinase	4.492	-0.956	0.044
9506733	GJB5	gap junction protein beta 5	4.907	-0.990	0.010
16758218	Hamp	hepcidin antimicrobial peptide	5.267	-0.990	0.010
20301998	PROK2	prokineticin 2	5.358	-0.979	0.021
672052120	RBM12B	RNA binding motif protein 12B	6.366	-0.990	0.010
293347435	PTPRD	protein tyrosine phosphatase receptor type D	7.710	-0.990	0.010
149057336	ZSCAN2	zinc finger and SCAN domain containing 2	7.758	-0.980	0.020
62650795	DACT1	dishevelled binding antagonist of beta catenin 1	7.762	-0.984	0.016
109472884	UBE3C	ubiquitin protein ligase E3C	8.197	-0.990	0.010

Supplementary Table S11. The list of genes that are differentially expressed in the offspring hippocampus in response to prenatal BPA exposure that exhibited the changes in the expression levels correlated with the cell density in the granular cell layer of the dentate gyrus. The transcriptome profiling data of DEGs in male and female rat offspring prenatally exposed to BPA (n = 6, male pups n = 3 and female pups n = 3, from independent litters) or the vehicle control (n = 6, male pups n = 3 and female pups n = 3, from independent litters) were obtained and used for the PTM analyses to identify DEGs that exhibited the changes in the expression levels correlated with the cell density in the granular cell layer of the dentate gyrus.

ID	Symbol	Entrez Gene Name	log2(FC)	R values	P-values
293347435	PTPRD	protein tyrosine phosphatase receptor type D	-7.731	0.985	0.015
564375502	Mxra7	matrix-remodelling associated 7	-7.209	0.972	0.028
392339806	CFAP69	cilia and flagella associated protein 69	-6.820	0.953	0.047
564310188	IGDCC4	immunoglobulin superfamily DCC subclass member 4	-6.728	0.961	0.039
564314389	DZIP3	DAZ interacting zinc finger protein 3	-6.700	0.994	0.006
149020633	TAF1D	TATA-box binding protein associated factor, RNA polymerase I subunit D	-6.476	0.994	0.006
157818475	SMIM22	small integral membrane protein 22	-5.615	0.992	0.008
567315993	LOC102550396	LRRGT00188	-5.600	0.975	0.025
564312230	LOC100912948	multidrug resistance-associated protein 1-like	-5.285	0.972	0.028
197384778	Snorc	secondary ossification center associated regulator of chondrocyte maturation	-5.044	0.991	0.009
564323305	LOC681300	similar to CXXC finger 5	-4.672	0.994	0.006
209447125	Ctf2	cardiotrophin 2	-4.392	0.972	0.028
201860265	NRN1L	neuritin 1 like	-4.358	0.979	0.021
300798104	IFNLR1	interferon lambda receptor 1	-4.248	0.990	0.010
51591901	MPIG6B	megakaryocyte and platelet inhibitory receptor G6b	-4.170	0.972	0.028
564298047	GDPD5	glycerophosphodiester phosphodiesterase domain containing 5	-4.163	0.999	0.001
300798035	NRG4	neuregulin 4	-4.044	0.980	0.020
61556838	Raet1d/Raet1e	retinoic acid early transcript 1E	-4.044	0.980	0.020
114145748	LOC680227	LRRGT00193	-3.907	0.963	0.037
194474016	SLC30A8	solute carrier family 30 member 8	-3.807	0.955	0.045
6978515	APOA1	apolipoprotein A1	-3.807	0.972	0.028
117647210	CTRC	chymotrypsin C	-3.807	0.972	0.028
8392926	ASGR2	asialoglycoprotein receptor 2	-3.700	0.969	0.031
48040447	SUCNR1	succinate receptor 1	-3.700	0.972	0.028

194473646	UPK3A	uroplakin 3A	-3.585	0.972	0.028
164518908	RAB25	RAB25, member RAS oncogene family	-3.459	0.972	0.028
285026465	HS3ST3A1	heparan sulfate-glucosamine 3-sulfotransferase 3A1	-3.322	0.972	0.028
148710035	PITX3	paired like homeodomain 3	-3.322	0.972	0.028
9506451	CA5A	carbonic anhydrase 5A	-3.248	0.986	0.014
56788780	KRT19	keratin 19	-3.248	0.998	0.002
56090299	ODF4	outer dense fiber of sperm tails 4	-3.170	0.952	0.048
157822159	CCDC42	coiled-coil domain containing 42	-3.170	0.972	0.028
123173794	GSG1	germ cell associated 1	-3.170	0.972	0.028
392339806	CFAP69	cilia and flagella associated protein 69	-2.947	0.975	0.025
20302091	PLB1	phospholipase B1	-2.907	0.955	0.045
157821903	Slc7a15	solute carrier family 7 (cationic amino acid transporter, y+ system), member 15	-2.907	0.955	0.045
77917534	CBLC	Cbl proto-oncogene C	-2.807	0.972	0.028
157822121	LRMDA	leucine rich melanocyte differentiation associated	-2.747	0.952	0.048
51948496	SLC22A18	solute carrier family 22 member 18	-2.687	0.992	0.008
298566276	Ces1a	carboxylesterase 1A	-2.585	0.972	0.028
57222328	PFN4	profilin family member 4	-2.585	0.972	0.028
157786772	KREMEN2	kringle containing transmembrane protein 2	-2.585	0.972	0.028
306482632	BPIFB4	BPI fold containing family B member 4	-2.585	0.972	0.028
57222300	Klra2	killer cell lectin-like receptor, subfamily A, member 2	-2.585	0.972	0.028
157822463	Nkx6-3	NK6 homeobox 3	-2.585	0.972	0.028
157822853	GUCA1A	guanylate cyclase activator 1A	-2.509	0.968	0.032
162417984	Vom2r12 (includes others)	vomeronasal 2 receptor, 80	-2.459	0.995	0.005
564391680	MAP3K8	mitogen-activated protein kinase kinase kinase 8	-2.426	0.979	0.021
62945342	LAX1	lymphocyte transmembrane adaptor 1	-2.322	0.972	0.028
187469451	CLEC7A	C-type lectin domain containing 7A	-2.322	0.972	0.028
568914626	GARNL3	GTPase activating Rap/RanGAP domain like 3	-2.261	0.966	0.034
62079023	ADTRP	androgen dependent TFPI regulating protein	-2.246	0.995	0.005
404247470	OLR1	oxidized low density lipoprotein receptor 1	-2.170	0.973	0.027
66730349	SPIB	Spi-B transcription factor	-2.170	0.994	0.006
148672128	SMAGP	small cell adhesion glycoprotein	-2.077	0.990	0.010

55741882	ZPBP2	zona pellucida binding protein 2	-2.022	0.977	0.023
157822587	PDE6B	phosphodiesterase 6B	-2.000	0.972	0.028
157818091	TMEM182	transmembrane protein 182	-2.000	0.972	0.028
296483047	SIX1	SIX homeobox 1	-2.000	0.972	0.028
117647206	DDX4	DEAD-box helicase 4	-2.000	0.972	0.028
19424304	CHRNB3	cholinergic receptor nicotinic beta 3 subunit	-2.000	0.972	0.028
47577151	Olfr1441	olfactory receptor 1441	-1.972	0.972	0.028
157820583	ANKRD34C	ankyrin repeat domain 34C	-1.939	0.966	0.034
117647214	EDN3	endothelin 3	-1.913	0.991	0.009
157823875	EPS8L1	EPS8 like 1	-1.907	0.980	0.020
81295367	Abcg3	ATP binding cassette subfamily G member 3	-1.898	0.961	0.039
672029702	TUT7	terminal uridylyl transferase 7	-1.854	0.960	0.040
13592031	PTGER2	prostaglandin E receptor 2	-1.845	0.960	0.040
19424240	PCSK4	proprotein convertase subtilisin/kexin type 4	-1.740	0.979	0.021
9910234	IFIT1B	interferon induced protein with tetratricopeptide repeats 1B	-1.678	0.972	0.028
31542125	ALOX15	arachidonate 15-lipoxygenase	-1.611	0.988	0.012
58331159	GSTA3	glutathione S-transferase alpha 3	-1.603	0.952	0.048
76443687	SLC4A1	solute carrier family 4 member 1 (Diego blood group)	-1.599	0.991	0.009
157821527	RHOD	ras homolog family member D	-1.597	0.969	0.031
209870037	INSRR	insulin receptor related receptor	-1.585	0.987	0.013
157818603	CLCA2	chloride channel accessory 2	-1.585	0.972	0.028
685156911	NLRP4	NLR family pyrin domain containing 4	-1.585	0.972	0.028
194473618	SCX	scleraxis bHLH transcription factor	-1.585	0.994	0.006
149034165	GALNT15	polypeptide N-acetylgalactosaminyltransferase 15	-1.561	0.971	0.029
13929066	CPZ	carboxypeptidase Z	-1.549	0.960	0.040
13929126	GALNT5	polypeptide N-acetylgalactosaminyltransferase 5	-1.478	0.981	0.019
81295349	SLC52A3	solute carrier family 52 member 3	-1.453	0.994	0.006
6981148	LEP	leptin	-1.436	0.951	0.049
283806636	ZNF831	zinc finger protein 831	-1.433	0.994	0.006
157822105	SLC49A3	solute carrier family 49 member 3	-1.389	0.953	0.047
564314671	VPS8	VPS8 subunit of CORVET complex	-1.386	0.957	0.043
6978717	CTRB2	chymotrypsinogen B2	-1.342	0.956	0.044
157786780	MELTF	melanotransferrin	-1.333	0.986	0.014
29789044	SNAI2	snail family transcriptional repressor 2	-1.328	0.993	0.007
157823345	LRR1	leucine rich repeat protein 1	-1.322	0.969	0.031
307746876	Pzp	PZP, alpha-2-macroglobulin like	-1.322	0.972	0.028

148694035	SENP8	SUMO peptidase family member, NEDD8 specific	-1.276	0.995	0.005
62656582	KIAA0100	KIAA0100	-1.266	0.980	0.020
187281975	DENND1C	DENN domain containing 1C	-1.216	0.992	0.008
56119141	BTK	Bruton tyrosine kinase	-1.205	0.982	0.018
564300462	DCHS2	dachsous cadherin-related 2	-1.190	1.000	0.000
58866008	TMC5	transmembrane channel like 5	-1.180	0.986	0.014
392334475	Myb	MYB proto-oncogene, transcription factor	-1.175	0.971	0.029
71795615	UPP1	uridine phosphorylase 1	-1.167	0.965	0.035
157823801	SLC50A1	solute carrier family 50 member 1	-1.136	0.990	0.010
125347412	FAM72A	family with sequence similarity 72 member A	-1.106	0.991	0.009
197385083	C1orf194	chromosome 1 open reading frame 194	-1.090	0.962	0.038
24308484	SLC17A8	solute carrier family 17 member 8	-1.089	0.973	0.027
157819247	CPA4	carboxypeptidase A4	-1.087	0.972	0.028
320089574	FAM161A	FAM161 centrosomal protein A	-1.080	0.969	0.031
300797017	ATG16L2	autophagy related 16 like 2	-1.079	0.952	0.048
9910378	CDC42SE2	CDC42 small effector 2	-1.069	0.979	0.021
197384727	Smco4	single-pass membrane protein with coiled-coil domains 4	-1.065	0.966	0.034
672020326	MTA3	metastasis associated 1 family member 3	-1.047	0.988	0.012
300797728	MGST3	microsomal glutathione S-transferase 3	-1.011	0.967	0.033
156231008	PRND	prion like protein doppel	-1.000	0.972	0.028
765826426	Acot6	acyl-CoA thioesterase 6	-1.000	0.957	0.043
59676595	FAM20A	FAM20A golgi associated secretory pathway pseudokinase	-1.000	0.965	0.035
569009290	TENM1	teneurin transmembrane protein 1	-0.985	0.987	0.013
407228396	THEMIS2	thymocyte selection associated family member 2	-0.980	0.979	0.021
157817157	FAM166C	family with sequence similarity 166 member C	-0.979	0.980	0.020
672033256	LOC100912904	disks large homolog 5-like	-0.960	0.952	0.048
157820951	PRSS53	serine protease 53	-0.959	0.954	0.046
157817989	MDFI	MyoD family inhibitor	-0.958	0.984	0.016
672052120	RBM12B	RNA binding motif protein 12B	-0.957	0.973	0.027
564392197	LOC684327	similar to inter-alpha (globulin) inhibitor H5	-0.954	0.983	0.017
157818275	KCNG4	potassium voltage-gated channel modifier subfamily G member 4	-0.952	0.999	0.001
148680846	HIC1	HIC ZBTB transcriptional repressor 1	-0.940	0.965	0.035

157823079	RBKS	ribokinase	-0.939	0.964	0.036
198442873	CDC14A	cell division cycle 14A	-0.936	0.982	0.018
71043648	LOC499742	LRRG00137	-0.931	0.973	0.027
62945330	SLC8B1	solute carrier family 8 member B1	-0.929	0.978	0.022
50657416	C1RL	complement C1r subcomponent like	-0.918	0.972	0.028
157819487	TACO1	translational activator of cytochrome c oxidase I	-0.917	0.952	0.048
564318923	WDR17	WD repeat domain 17	-0.904	0.967	0.033
14861868	Ptprv	protein tyrosine phosphatase, receptor type, V	-0.893	0.970	0.030
149056256	FXYD5	FXYD domain containing ion transport regulator 5	-0.880	0.953	0.047
392351290	DNAH9	dynein axonemal heavy chain 9	-0.875	0.973	0.027
209870105	GPR37L1	G protein-coupled receptor 37 like 1	-0.870	0.965	0.035
157817185	PPCDC	phosphopantothenoylcysteine decarboxylase	-0.866	0.960	0.040
57114338	SCN4B	sodium voltage-gated channel beta subunit 4	-0.859	0.953	0.047
149031998	ACVRL1	activin A receptor like type 1	-0.852	0.974	0.026
6980958	SLC2A4	solute carrier family 2 member 4	-0.837	0.984	0.016
392334002	CCDC3	coiled-coil domain containing 3	-0.833	0.979	0.021
56605720	GADD45B	growth arrest and DNA damage inducible beta	-0.828	0.976	0.024
157817065	KCNK16	potassium two pore domain channel subfamily K member 16	-0.807	0.969	0.031
564396113	ZCCHC14	zinc finger CCHC-type containing 14	-0.797	0.961	0.039
564345556	CROT	carnitine O-octanoyltransferase	-0.790	0.993	0.007
300798165	ZBTB40	zinc finger and BTB domain containing 40	-0.788	0.997	0.003
942523340	CAPRIN2	caprin family member 2	-0.774	0.968	0.032
157819205	EFHC2	EF-hand domain containing 2	-0.772	0.966	0.034
57528269	ABHD14A	abhydrolase domain containing 14A	-0.760	0.983	0.017
13591914	ANPEP	alanyl aminopeptidase, membrane	-0.757	0.980	0.020
672069802	C1QTNF1	C1q and TNF related 1	-0.749	0.951	0.049
564312886	DNAH2	dynein axonemal heavy chain 2	-0.740	0.982	0.018
157786850	TUBD1	tubulin delta 1	-0.740	0.993	0.007
208973286	RBM46	RNA binding motif protein 46	-0.724	0.984	0.016
19173756	ERG	ETS transcription factor ERG	-0.718	0.987	0.013
149047075	Spaca6	sperm acrosome associated 6	-0.715	0.986	0.014
564378828	TFR2	transferrin receptor 2	-0.707	0.995	0.005
55742713	ECM1	extracellular matrix protein 1	-0.699	0.988	0.012
157816997	BDH2	3-hydroxybutyrate dehydrogenase 2	-0.698	0.991	0.009
48675865	PDP2	pyruvate dehyrogenase phosphatase catalytic subunit 2	-0.692	0.957	0.043

38454234	COL27A1	collagen type XXVII alpha 1 chain	-0.677	0.970	0.030
564320335	TMEM241	transmembrane protein 241	-0.675	0.977	0.023
74142284	DSE	dermatan sulfate epimerase	-0.667	0.997	0.003
157818909	Zim1	zinc finger, imprinted 1	-0.665	0.994	0.006
67514566	POLA2	DNA polymerase alpha 2, accessory subunit	-0.665	0.965	0.035
672057459	DGKA	diacylglycerol kinase alpha	-0.664	0.970	0.030
13592057	RPL18	ribosomal protein L18	-0.660	0.960	0.040
114145782	MORN5	MORN repeat containing 5	-0.657	0.971	0.029
77020281	CD55	CD55 molecule (Cromer blood group)	-0.648	0.985	0.015
672039742	TKFC	triokinase and FMN cyclase	-0.646	0.972	0.028
68534736	ERAP1	endoplasmic reticulum aminopeptidase 1	-0.646	0.959	0.041
13786136	PDGFC	platelet derived growth factor C	-0.642	0.959	0.041
149061352	ADAM12	ADAM metallopeptidase domain 12	-0.638	0.998	0.002
672060362	ELFN2	extracellular leucine rich repeat and fibronectin type III domain containing 2	-0.636	0.977	0.023
58865948	CREB3L2	cAMP responsive element binding protein 3 like 2	-0.634	0.965	0.035
50657355	TOP1MT	DNA topoisomerase I mitochondrial	-0.632	0.966	0.034
397529557	C8orf58	chromosome 8 open reading frame 58	-0.629	0.998	0.002
58865854	SCRN2	secernin 2	-0.627	0.967	0.033
16758622	IFT172	intraflagellar transport 172	-0.627	0.993	0.007
478732983	MAP3K5	mitogen-activated protein kinase kinase kinase 5	-0.616	0.988	0.012
402478640	HTRA3	HtrA serine peptidase 3	-0.608	0.953	0.047
149068766	PLEKHB1	pleckstrin homology domain containing B1	-0.608	0.970	0.030
157819065	ADAMTS15	ADAM metallopeptidase with thrombospondin type 1 motif 15	-0.604	0.997	0.003
157818491	DUS2	dihydrouridine synthase 2	-0.602	0.965	0.035
27465529	SLC9A4	solute carrier family 9 member A4	-0.601	0.967	0.033
293347888	SRBD1	S1 RNA binding domain 1	-0.598	0.992	0.008
564387543	UGGT2	UDP-glucose glycoprotein glucosyltransferase 2	-0.596	0.960	0.040
108935976	DISC1	DISC1 scaffold protein	-0.590	0.993	0.007
197927123	LYRM7	LYR motif containing 7	-0.587	0.982	0.018
672086986	SLC38A5	solute carrier family 38 member 5	-0.583	0.974	0.026
564394999	CLGN	calmegin	-0.582	0.986	0.014
16758560	WIF1	WNT inhibitory factor 1	-0.573	0.982	0.018
78486556	RUSF1	RUS family member 1	-0.572	0.955	0.045
564390348	Klhl3	kelch-like family member 3	-0.566	0.967	0.033

62078799	QRSL1	glutaminyl-tRNA amidotransferase subunit QRSL1	-0.566	0.976	0.024
157817903	Dcaf12l1	DDB1 and CUL4 associated factor 12-like 1	-0.565	0.961	0.039
57012436	Krt10	keratin 10	-0.562	0.968	0.032
300798350	LRRK1	leucine rich repeat kinase 1	-0.554	0.966	0.034
199562000	USP40	ubiquitin specific peptidase 40	-0.552	0.983	0.017
171846640	FBLN1	fibulin 1	-0.543	0.996	0.004
672066171	GIN1	gypsy retrotransposon integrase 1	-0.539	0.964	0.036
6754808	NDP	norrin cystine knot growth factor NDP	-0.538	0.952	0.048
16758390	CLIC5	chloride intracellular channel 5	-0.535	0.969	0.031
20302097	PIGL	phosphatidylinositol glycan anchor biosynthesis class L	-0.534	0.962	0.038
148683700	TMEM98	transmembrane protein 98	-0.533	0.966	0.034
56090301	NUDT5	nudix hydrolase 5	-0.526	0.956	0.044
40018594	POPDC2	popeye domain containing 2	-0.525	0.955	0.045
19424232	CSF2RB	colony stimulating factor 2 receptor subunit beta	-0.524	0.988	0.012
124244050	PPIP5K1	diphosphoinositol pentakisphosphate kinase 1	-0.522	0.959	0.041
157822555	RIN3	Ras and Rab interactor 3	-0.520	0.997	0.003
568966731	HMG20B	high mobility group 20B	-0.518	0.971	0.029
6981180	MAOB	monoamine oxidase B	-0.518	0.963	0.037
148692356	ARHGEF1	Rho guanine nucleotide exchange factor 1	-0.517	0.986	0.014
149047863	LOC690190	hypothetical protein LOC690190	-0.514	0.994	0.006
147900684	TLR7	toll like receptor 7	-0.511	0.986	0.014
308044487	KIAA0319	KIAA0319	-0.509	0.981	0.019
148698795	GPX7	glutathione peroxidase 7	-0.507	0.995	0.005
564355112	EMILIN1	elastin microfibril interfacer 1	-0.503	0.955	0.045
654824082	Fbxl21	F-box and leucine-rich repeat protein 21	-0.500	0.991	0.009
50233928	TMEM159	transmembrane protein 159	-0.497	0.994	0.006
564330609	SYT17	synaptotagmin 17	-0.494	0.984	0.016
84662732	DNASE1L1	deoxyribonuclease 1 like 1	-0.494	0.970	0.030
62078635	CCDC153	coiled-coil domain containing 153	-0.494	0.985	0.015
399220341	SLC2A13	solute carrier family 2 member 13	-0.492	0.991	0.009
157819347	CDC6	cell division cycle 6	-0.492	0.989	0.011
6978867	GABRB1	gamma-aminobutyric acid type A receptor subunit beta1	-0.487	0.981	0.019
149058686	PIGR	polymeric immunoglobulin receptor	-0.485	0.969	0.031
164607119	SUMF2	sulfatase modifying factor 2	-0.483	0.964	0.036
395759219	AQP4	aquaporin 4	-0.482	0.953	0.047

16758322	SYNGR2	synaptogyrin 2	-0.481	0.953	0.047
157073947	C1orf74	chromosome 1 open reading frame 74	-0.478	0.991	0.009
189011606	NCEH1	neutral cholesterol ester hydrolase 1	-0.476	0.976	0.024
157816939	WASHC3	WASH complex subunit 3	-0.469	0.958	0.042
157823399	COG4	component of oligomeric golgi complex 4	-0.468	0.982	0.018
75905809	AKAP12	A-kinase anchoring protein 12	-0.466	0.973	0.027
41056215	XRCC5	X-ray repair cross complementing 5	-0.466	0.955	0.045
672044191	TBCK	TBC1 domain containing kinase	-0.465	0.992	0.008
56090632	DMAC2L	distal membrane arm assembly complex 2 like	-0.463	0.999	0.001
148669431	DNAJC27	DnaJ heat shock protein family (Hsp40) member C27	-0.459	1.000	0.000
56090411	POLE3	DNA polymerase epsilon 3, accessory subunit	-0.456	0.987	0.013
157817743	CDH5	cadherin 5	-0.454	0.971	0.029
9437326	SLC4A4	solute carrier family 4 member 4	-0.453	0.956	0.044
157817911	C21orf62	chromosome 21 open reading frame 62	-0.451	0.979	0.021
33414515	PXK	PX domain containing serine/threonine kinase like	-0.450	0.973	0.027
58865380	STAT2	signal transducer and activator of transcription 2	-0.450	0.993	0.007
77993368	ACSF2	acyl-CoA synthetase family member 2	-0.444	0.987	0.013
392331978	CDR2L	cerebellar degeneration related protein 2 like	-0.442	0.970	0.030
157786756	CDC45	cell division cycle 45	-0.442	0.989	0.011
149058661	RAB7B	RAB7B, member RAS oncogene family	-0.439	0.968	0.032
148686921	SLC24A4	solute carrier family 24 member 4	-0.435	0.981	0.019
404312655	SDR42E1	short chain dehydrogenase/reductase family 42E, member 1	-0.435	1.000	0.000
157823259	TMEM229A	transmembrane protein 229A	-0.424	0.956	0.044
76443683	LOC100912042/Surf2	surfeit 2	-0.423	0.971	0.029
300797330	PTPRU	protein tyrosine phosphatase receptor type U	-0.422	0.965	0.035
210032365	HSP90B1	heat shock protein 90 beta family member 1	-0.421	0.987	0.013
56090564	GALM	galactose mutarotase	-0.420	0.989	0.011
157823279	CGNL1	cingulin like 1	-0.418	0.953	0.047
74218228	HNRNPC	heterogeneous nuclear ribonucleoprotein C	-0.418	0.960	0.040

13994179	SLC24A2	solute carrier family 24 member 2	-0.416	0.997	0.003
62078539	Pagr1	Paxip1-associated glutamate-rich protein 1	-0.415	0.958	0.042
62945312	CXCL16	C-X-C motif chemokine ligand 16	-0.414	0.990	0.010
50811823	NENF	neudesin neurotrophic factor	-0.413	0.959	0.041
68342019	LRRC17	leucine rich repeat containing 17	-0.413	0.964	0.036
157822913	LHFPL2	LHFPL tetraspan subfamily member 2	-0.410	0.953	0.047
13591949	GATM	glycine amidinotransferase	-0.409	0.987	0.013
672061813	ACSBG1	acyl-CoA synthetase bubblegum family member 1	-0.409	0.972	0.028
157786690	PRKCA	protein kinase C alpha	-0.408	0.971	0.029
109480433	GNPTAB	N-acetylglucosamine-1-phosphate transferase subunits alpha and beta	-0.407	0.960	0.040
564351356	PAPPA	pappalysin 1	-0.407	0.961	0.039
13162347	FDXR	ferredoxin reductase	-0.405	0.977	0.023
6981664	TNFRSF1A	TNF receptor superfamily member 1A	-0.403	0.962	0.038
149049048	RECQL	RecQ like helicase	-0.403	0.972	0.028
127140886	EML6	EMAP like 6	-0.403	0.973	0.027
122065191	ABAT	4-aminobutyrate aminotransferase	-0.401	0.976	0.024
58865466	SLC37A1	solute carrier family 37 member 1	-0.401	0.983	0.017
77695926	STAT1	signal transducer and activator of transcription 1	-0.399	0.950	0.050
149041576	REXO2	RNA exonuclease 2	-0.398	0.986	0.014
148666792	ARHGAP25	Rho GTPase activating protein 25	-0.396	0.979	0.021
78187977	TCF19	transcription factor 19	-0.393	0.999	0.001
77917572	LIPA	lipase A, lysosomal acid type	-0.392	0.980	0.020
149052857	KCNJ12	potassium inwardly rectifying channel subfamily J member 12	-0.392	0.990	0.010
157820327	THSD1	thrombospondin type 1 domain containing 1	-0.391	0.963	0.037
187937124	TMEM126B	transmembrane protein 126B	-0.389	0.952	0.048
51948488	SIRT5	sirtuin 5	-0.388	0.992	0.008
296040479	TXNRD3	thioredoxin reductase 3	-0.387	0.978	0.022
148690851	RCN3	reticulocalbin 3	-0.381	0.981	0.019
564305413	E130308A19Rik	RIKEN cDNA E130308A19 gene	-0.381	0.970	0.030
149036529	DGUOK	deoxyguanosine kinase	-0.381	0.957	0.043
40786487	GPR108	G protein-coupled receptor 108	-0.379	0.979	0.021
18426846	DCBLD2	discoidin, CUB and LCCL domain containing 2	-0.378	0.964	0.036
564299653	FAM169A	family with sequence similarity 169 member A	-0.372	0.962	0.038

113061	CHRNA3	cholinergic receptor nicotinic alpha 3 subunit	-0.372	0.990	0.010
198386343	TRPS1	transcriptional repressor GATA binding 1	-0.370	0.951	0.049
8393861	HPCAL4	hippocalcin like 4	-0.370	0.988	0.012
157819959	PCDHB2	protocadherin beta 2	-0.369	0.983	0.017
158303308	PCCA	propionyl-CoA carboxylase subunit alpha	-0.369	0.987	0.013
9247217	MSX1	msh homeobox 1	-0.368	0.959	0.041
564343748	CDK5RAP1	CDK5 regulatory subunit associated protein 1	-0.368	0.952	0.048
157822461	C20orf194	chromosome 20 open reading frame 194	-0.366	0.956	0.044
158534064	RET	ret proto-oncogene	-0.365	0.995	0.005
6978761	DGKG	diacylglycerol kinase gamma	-0.362	0.969	0.031
16758712	PDIA4	protein disulfide isomerase family A member 4	-0.362	0.988	0.012
55741549	MRPL13	mitochondrial ribosomal protein L13	-0.360	0.952	0.048
29293811	SERPINF1	serpin family F member 1	-0.359	0.954	0.046
149066868	MDM1	Mdm1 nuclear protein	-0.357	0.952	0.048
215272398	HIP1	huntingtin interacting protein 1	-0.356	0.952	0.048
672052705	FRRS1L	ferric chelate reductase 1 like	-0.354	0.971	0.029
16758024	SYT9	synaptotagmin 9	-0.351	0.988	0.012
149060100	AIFM1	apoptosis inducing factor mitochondria associated 1	-0.351	0.982	0.018
564329392	FLNA	filamin A	-0.347	0.960	0.040
56119120	SNF8	SNF8 subunit of ESCRT-II	-0.343	0.980	0.020
62078847	TSEN2	tRNA splicing endonuclease subunit 2	-0.343	0.995	0.005
77157795	MAL2	mal, T cell differentiation protein 2 (gene/pseudogene)	-0.342	0.973	0.027
157820807	GCDH	glutaryl-CoA dehydrogenase	-0.339	0.952	0.048
11693162	INSIG1	insulin induced gene 1	-0.339	0.968	0.032
56605710	LTBR	lymphotoxin beta receptor	-0.338	0.968	0.032
71361669	CIT	citron rho-interacting serine/threonine kinase	-0.336	0.967	0.033
57527061	ZGPAT	zinc finger CCCH-type and G-patch domain containing	-0.335	0.990	0.010
148683194	INTS3	integrator complex subunit 3	-0.335	0.998	0.002
672085480	MEAK7	MTOR associated protein, eak-7 homolog	-0.334	0.952	0.048
78486544	SLC5A2	solute carrier family 5 member 2	-0.334	0.961	0.039
300794275	MFSD10	major facilitator superfamily domain containing 10	-0.330	0.994	0.006
672038615	GSG1L	GSG1 like	-0.330	0.990	0.010

564309649	CCDC159	coiled-coil domain containing 159	-0.329	0.977	0.023
22024392	KIF1C	kinesin family member 1C	-0.328	0.994	0.006
77628027	PSMC3IP	PSMC3 interacting protein	-0.328	0.976	0.024
61557206	ZBTB16	zinc finger and BTB domain containing 16	-0.326	0.954	0.046
564344373	ZMYND8	zinc finger MYND-type containing 8	-0.325	0.992	0.008
149045696	Ccl27a	chemokine (C-C motif) ligand 27A	-0.325	0.988	0.012
51036680	SLC29A3	solute carrier family 29 member 3	-0.322	0.992	0.008
58865398	LAP3	leucine aminopeptidase 3	-0.320	0.996	0.004
672050244	APLF	aprataxin and PNKP like factor	-0.318	0.951	0.049
6678297	TEX261	testis expressed 261	-0.318	0.964	0.036
157823879	NUDT12	nudix hydrolase 12	-0.317	0.954	0.046
157819457	MAP3K14	mitogen-activated protein kinase kinase kinase 14	-0.317	0.968	0.032
51948466	TMED3	transmembrane p24 trafficking protein 3	-0.316	0.958	0.042
40018538	ADI1	acireductone dioxygenase 1	-0.316	0.998	0.002
300797242	SPG11	SPG11 vesicle trafficking associated, spatacsin	-0.316	0.954	0.046
57528352	DMAC2	distal membrane arm assembly complex 2	-0.315	0.979	0.021
281332095	RB1	RB transcriptional corepressor 1	-0.313	0.966	0.034
149041432	THY1	Thy-1 cell surface antigen	-0.313	0.968	0.032
157786608	MRPL55	mitochondrial ribosomal protein L55	-0.313	0.989	0.011
564336403	EXOSC8	exosome component 8	-0.309	0.964	0.036
167860097	FN3KRP	fructosamine 3 kinase related protein	-0.308	0.992	0.008
966975500	MMP17	matrix metallopeptidase 17	-0.306	0.964	0.036
29789369	PTPRG	protein tyrosine phosphatase receptor type G	-0.306	0.981	0.019
545532952	EIF4E3	eukaryotic translation initiation factor 4E family member 3	-0.305	0.951	0.049
564372688	RPA1	replication protein A1	-0.305	0.972	0.028
399124777	GLS2	glutaminase 2	-0.305	0.966	0.034
300796069	THADA	THADA armadillo repeat containing	-0.303	0.967	0.033
198041989	PARVB	parvin beta	-0.302	0.983	0.017
157820737	NUSAP1	nucleolar and spindle associated protein 1	-0.298	0.987	0.013
149032924	ARG1	arginase 1	-0.296	0.981	0.019
6978888	GFRA1	GDNF family receptor alpha 1	-0.294	0.961	0.039
564358911	CHPT1	choline phosphotransferase 1	-0.292	0.995	0.005
157817979	Egfem1	EGF-like and EMI domain containing 1	-0.291	0.984	0.016
74223968	5031425E22Rik	RIKEN cDNA 5031425E22 gene	-0.290	0.991	0.009
149057745	NEK3	NIMA related kinase 3	-0.290	0.985	0.015

149063995	GMPR2	guanosine monophosphate reductase 2	-0.289	0.987	0.013
162287200	CD82	CD82 molecule	-0.289	0.978	0.022
157820517	CARD6	caspase recruitment domain family member 6	-0.287	0.957	0.043
564387894	BTD	biotinidase	-0.287	0.996	0.004
157820833	HERC3	HECT and RLD domain containing E3 ubiquitin protein ligase 3	-0.287	0.963	0.037
51948412	ETFB	electron transfer flavoprotein subunit beta	-0.286	0.954	0.046
76559913	COG7	component of oligomeric golgi complex 7	-0.283	0.960	0.040
149034870	RNF6	ring finger protein 6	-0.283	0.992	0.008
142349612	GLUL	glutamate-ammonia ligase	-0.279	0.972	0.028
564364473	RNF111	ring finger protein 111	-0.276	0.979	0.021
62078997	WDR1	WD repeat domain 1	-0.273	0.989	0.011
56605664	METTL23	methyltransferase like 23	-0.272	0.976	0.024
149022319	AGPS	alkylglycerone phosphate synthase	-0.270	0.999	0.001
157819337	SLC35B4	solute carrier family 35 member B4	-0.270	0.965	0.035
57164113	NSDHL	NAD(P) dependent steroid dehydrogenase-like	-0.269	0.987	0.013
74354506	ACBD5	acyl-CoA binding domain containing 5	-0.269	0.995	0.005
392339847	CADPS2	calcium dependent secretion activator 2	-0.268	0.986	0.014
38181552	SCG2	secretogranin II	-0.267	0.976	0.024
157819753	RCN1	reticulocalbin 1	-0.266	0.998	0.002
300795339	RYR2	ryanodine receptor 2	-0.265	0.983	0.017
564382316	HSD11B1	hydroxysteroid 11-beta dehydrogenase 1	-0.265	0.996	0.004
208973280	TRIM65	tripartite motif containing 65	-0.265	0.954	0.046
149041411	SC5D	sterol-C5-desaturase	-0.264	0.965	0.035
148372343	RAMP2	receptor activity modifying protein 2	-0.264	0.984	0.016
564344520	LOC102555457	engulfment and cell motility protein 2-like	-0.261	0.977	0.023
564382292	ANGEL2	angel homolog 2	-0.258	0.972	0.028
17865325	GLRB	glycine receptor beta	-0.256	0.974	0.026
672041250	ARHGEF28	Rho guanine nucleotide exchange factor 28	-0.253	0.990	0.010
8394502	UBC	ubiquitin C	-0.249	0.970	0.030
149057830	Hgsnat	heparan-alpha-glucosaminide N-acetyltransferase	-0.248	0.972	0.028
157823901	TSPAN9	tetraspanin 9	-0.247	0.979	0.021
157819077	TRIM37	tripartite motif containing 37	-0.246	0.959	0.041

6978631	CD4	CD4 molecule	-0.245	0.990	0.010
290563168	DUSP3	dual specificity phosphatase 3	-0.243	0.994	0.006
764020083	CLUH	clustered mitochondria homolog	-0.242	0.958	0.042
197382169	CNTROB	centrobin, centriole duplication and spindle assembly protein	-0.241	0.959	0.041
12831215	KCNK10	potassium two pore domain channel subfamily K member 10	-0.240	0.965	0.035
58865958	RDH11	retinol dehydrogenase 11	-0.240	0.960	0.040
57192	P3H4	prolyl 3-hydroxylase family member 4 (inactive)	-0.240	0.989	0.011
584277046	SLC1A3	solute carrier family 1 member 3	-0.232	0.986	0.014
149053021	TMEM107	transmembrane protein 107	-0.232	0.954	0.046
293346766	TCAF1	TRPM8 channel associated factor 1	-0.232	0.987	0.013
9457244	RBBP9	RB binding protein 9, serine hydrolase	-0.231	0.995	0.005
157820653	TMEM63C	transmembrane protein 63C	-0.229	0.978	0.022
58865718	HERC4	HECT and RLD domain containing E3 ubiquitin protein ligase 4	-0.228	0.993	0.007
149062310	BSCL2	BSCL2 lipid droplet biogenesis associated, seipin	-0.228	0.987	0.013
281485606	STT3B	STT3 oligosaccharyltransferase complex catalytic subunit B	-0.228	0.992	0.008
293345175	DHX29	DExH-box helicase 29	-0.225	0.968	0.032
148697866	FAM3A	FAM3 metabolism regulating signaling molecule A	-0.222	0.960	0.040
157786994	C1orf21	chromosome 1 open reading frame 21	-0.222	0.999	0.001
78369663	SLC38A9	solute carrier family 38 member 9	-0.222	0.971	0.029
148689145	CPNE4	copine 4	-0.222	0.971	0.029
148695758	CAPRIN1	cell cycle associated protein 1	-0.221	0.964	0.036
157822873	FBH1	F-box DNA helicase 1	-0.219	0.983	0.017
564298436	WDR11	WD repeat domain 11	-0.216	0.959	0.041
6978890	GGH	gamma-glutamyl hydrolase	-0.215	0.977	0.023
8393643	KCNAB1	potassium voltage-gated channel subfamily A member regulatory beta subunit 1	-0.214	0.960	0.040
57526927	LARS1	leucyl-tRNA synthetase 1	-0.213	0.952	0.048
672066638	CLEC16A	C-type lectin domain containing 16A	-0.212	0.995	0.005
13928780	POR	cytochrome p450 oxidoreductase	-0.211	0.957	0.043
158749540	NPEPPS	aminopeptidase puromycin sensitive	-0.210	0.953	0.047
18959250	PRKCD	protein kinase C delta	-0.210	0.996	0.004
149058126	ALDH9A1	aldehyde dehydrogenase 9 family member A1	-0.208	0.978	0.022
37359962	PLPPR4	phospholipid phosphatase related 4	-0.207	0.998	0.002
225543229	TIAM1	TIAM Rac1 associated GEF 1	-0.207	0.984	0.016

148667192	LRTM2	leucine rich repeats and transmembrane domains 2	-0.207	0.997	0.003
6981504	ATXN1	ataxin 1	-0.204	0.978	0.022
157818191	SETD6	SET domain containing 6, protein lysine methyltransferase	-0.203	0.953	0.047
564391231	SERPINB9	serpin family B member 9	-0.203	0.986	0.014
13027430	WDR7	WD repeat domain 7	-0.202	0.951	0.049
142385975	RNF25	ring finger protein 25	-0.202	0.954	0.046
55741502	ACAT2	acetyl-CoA acetyltransferase 2	-0.202	0.973	0.027
48675867	PLPP3	phospholipid phosphatase 3	-0.201	0.996	0.004
38454284	PPM1E	protein phosphatase, Mg2+/Mn2+ dependent 1E	-0.201	0.969	0.031
149053315	CAMKK1	calcium/calmodulin dependent protein kinase kinase 1	-0.200	0.995	0.005
77404395	SND1	staphylococcal nuclease and tudor domain containing 1	-0.199	0.955	0.045
61556891	OSBPL2	oxysterol binding protein like 2	-0.198	0.969	0.031
564334053	SORCS1	sortilin related VPS10 domain containing receptor 1	-0.195	0.957	0.043
17530977	ECHS1	enoyl-CoA hydratase, short chain 1	-0.191	0.976	0.024
148696094	TUBGCP4	tubulin gamma complex associated protein 4	-0.189	0.969	0.031
68163551	TBC1D22B	TBC1 domain family member 22B	-0.187	0.955	0.045
564334013	GBF1	golgi brefeldin A resistant guanine nucleotide exchange factor 1	-0.187	0.985	0.015
157821901	PNMA3	PNMA family member 3	-0.186	0.980	0.020
672088942	ATP2B3	ATPase plasma membrane Ca2+ transporting 3	-0.186	0.995	0.005
312283667	WNK1	WNK lysine deficient protein kinase 1	-0.185	0.988	0.012
197209847	JAK1	Janus kinase 1	-0.185	0.996	0.004
59891444	FUT10	fucosyltransferase 10	-0.185	0.975	0.025
16923964	CNTN1	contactin 1	-0.185	0.998	0.002
76881830	Kcnip2	potassium voltage-gated channel interacting protein 2	-0.183	0.995	0.005
32185285	BCL2L2	BCL2 like 2	-0.181	0.966	0.034
157817620	PSD2	pleckstrin and Sec7 domain containing 2	-0.180	0.990	0.010
12621120	SFXN3	sideroflexin 3	-0.180	0.974	0.026
187468990	DNAJB2	DnaJ heat shock protein family (Hsp40) member B2	-0.180	0.993	0.007
293349000	ARHGAP42	Rho GTPase activating protein 42	-0.178	0.977	0.023
201023331	MAPK11	mitogen-activated protein kinase 11	-0.176	0.981	0.019
564390898	KIF13A	kinesin family member 13A	-0.176	0.961	0.039

157818193	TTPAL	alpha tocopherol transfer protein like	-0.176	0.977	0.023
78000203	Tpm1	tropomyosin 1, alpha	-0.174	0.997	0.003
564387640	DOCK9	dedicator of cytokinesis 9	-0.172	0.983	0.017
61557212	CIAO3	cytosolic iron-sulfur assembly component 3	-0.170	0.998	0.002
817472062	CDK5RAP3	CDK5 regulatory subunit associated protein 3	-0.167	0.977	0.023
564335900	ZFHX4	zinc finger homeobox 4	-0.167	0.983	0.017
19705545	RAB3IL1	RAB3A interacting protein like 1	-0.166	0.957	0.043
157817201	NETO1	neuropilin and tolloid like 1	-0.165	0.988	0.012
13242243	AXIN2	axin 2	-0.164	0.957	0.043
157819829	HACD3	3-hydroxyacyl-CoA dehydratase 3	-0.159	0.952	0.048
158186708	PDCD11	programmed cell death 11	-0.159	0.960	0.040
198278547	TMEM41A	transmembrane protein 41A	-0.159	0.994	0.006
56605830	CLBA1	clathrin binding box of aftiphilin containing 1	-0.158	0.951	0.049
209529636	PPA2	inorganic pyrophosphatase 2	-0.158	0.981	0.019
18034785	ABCB6	ATP binding cassette subfamily B member 6 (Langereis blood group)	-0.158	0.999	0.001
564299821	PARP8	poly(ADP-ribose) polymerase family member 8	-0.155	0.965	0.035
19173802	PPP1R14C	protein phosphatase 1 regulatory inhibitor subunit 14C	-0.155	0.952	0.048
18266726	PAICS	phosphoribosylaminoimidazole carboxylase and phosphoribosylaminoimidazolesuccino carboxamide synthase	-0.155	0.957	0.043
6649914	GDF11	growth differentiation factor 11	-0.154	0.957	0.043
113461996	COA5	cytochrome c oxidase assembly factor 5	-0.149	0.988	0.012
50510837	KIAA1191	KIAA1191	-0.148	0.985	0.015
717324516	SCN8A	sodium voltage-gated channel alpha subunit 8	-0.147	0.951	0.049
300794996	NDST3	N-deacetylase and N-sulfotransferase 3	-0.147	0.980	0.020
730229363	RALGAPA1	Ral GTPase activating protein catalytic subunit alpha 1	-0.142	0.979	0.021
9507043	RGS12	regulator of G protein signaling 12	-0.139	0.970	0.030
201066369	LGI2	leucine rich repeat LGI family member 2	-0.139	0.961	0.039
157823401	PIGH	phosphatidylinositol glycan anchor biosynthesis class H	-0.135	0.983	0.017
564338482	SORT1	sortilin 1	-0.134	0.969	0.031
300794317	SFI1	SFI1 centrin binding protein	-0.133	0.952	0.048

564345487	RINT1	RAD50 interactor 1	-0.132	0.990	0.010
56605798	RNF167	ring finger protein 167	-0.131	0.978	0.022
148747541	HNRNPU	heterogeneous nuclear ribonucleoprotein U	-0.130	0.997	0.003
568985444	CADPS	calcium dependent secretion activator	-0.129	0.990	0.010
37359832	SCRN1	secernin 1	-0.127	0.956	0.044
77415383	HSPA8	heat shock protein family A (Hsp70) member 8	-0.125	0.969	0.031
58865700	GRWD1	glutamate rich WD repeat containing 1	-0.123	0.995	0.005
157820919	POLE4	DNA polymerase epsilon 4, accessory subunit	-0.123	0.989	0.011
149064207	COMMD10	COMM domain containing 10	-0.123	0.961	0.039
253683488	NTRK2	neurotrophic receptor tyrosine kinase 2	-0.121	0.969	0.031
158254369	CDK10	cyclin dependent kinase 10	-0.121	0.981	0.019
74229032	TPCN1	two pore segment channel 1	-0.121	1.000	0.000
476007242	EPS8	epidermal growth factor receptor pathway substrate 8	-0.117	0.974	0.026
201066352	ANKRD6	ankyrin repeat domain 6	-0.116	0.952	0.048
8393390	GABRB3	gamma-aminobutyric acid type A receptor subunit beta3	-0.115	0.997	0.003
29789269	GRIA1	glutamate ionotropic receptor AMPA type subunit 1	-0.115	0.961	0.039
149059529	LOC100910558	uncharacterized LOC100910558	-0.114	0.989	0.011
11560079	KIT	KIT proto-oncogene, receptor tyrosine kinase	-0.114	0.950	0.050
404247435	YLPM1	YLP motif containing 1	-0.113	0.968	0.032
564353678	USP48	ubiquitin specific peptidase 48	-0.112	0.985	0.015
694981804	CLDN5	claudin 5	-0.108	0.963	0.037
89337260	FTO	FTO alpha-ketoglutarate dependent dioxygenase	-0.107	0.989	0.011
298231200	INSR	insulin receptor	-0.104	0.956	0.044
149036808	ARL6IP5	ADP ribosylation factor like GTPase 6 interacting protein 5	-0.099	0.959	0.041
403377905	SRGAP2	SLIT-ROBO Rho GTPase activating protein 2	-0.098	0.998	0.002
149060725	CEP19	centrosomal protein 19	-0.095	0.983	0.017
154800420	GNL3L	G protein nucleolar 3 like	-0.093	0.969	0.031
8980843	GRIPAP1	GRIP1 associated protein 1	-0.089	0.986	0.014
297206894	E4F1	E4F transcription factor 1	-0.074	0.998	0.002
564375060	SLC39A11	solute carrier family 39 member 11	-0.070	0.983	0.017
148747528	PTK2B	protein tyrosine kinase 2 beta	-0.066	0.965	0.035

255918181	NUS1	NUS1 dehydrodolichyl diphosphate synthase subunit	-0.062	0.981	0.019
20302113	STIP1	stress induced phosphoprotein 1	-0.060	0.952	0.048
408535187	PRDM11	PR/SET domain 11	-0.058	0.990	0.010
13385318	KDELR2	KDEL endoplasmic reticulum protein retention receptor 2	-0.057	0.998	0.002
162287208	FADS1	fatty acid desaturase 1	-0.050	0.969	0.031
189027133	TTC30B	tetratricopeptide repeat domain 30B	-0.044	0.952	0.048
157820825	IFT57	intraflagellar transport 57	0.053	-0.968	0.032
58865796	PTDSS1	phosphatidylserine synthase 1	0.057	-0.959	0.041
62078979	AMZ2	archaelysin family metallopeptidase 2	0.061	-0.951	0.049
25453374	PEX14	peroxisomal biogenesis factor 14	0.067	-0.964	0.036
62089200	ZDHHC9	zinc finger DHHC-type palmitoyltransferase 9	0.068	-0.982	0.018
83649695	SMIM14	small integral membrane protein 14	0.082	-0.986	0.014
60360532	OSBPL6	oxysterol binding protein like 6	0.083	-0.955	0.045
672074758	NCSTN	nicastrin	0.083	-0.970	0.030
149038024	RIPOR1	RHO family interacting cell polarization regulator 1	0.091	-0.966	0.034
189491673	FXR2	FMR1 autosomal homolog 2	0.097	-0.982	0.018
970596961	MAPK10	mitogen-activated protein kinase 10	0.099	-0.985	0.015
166064004	GTF3A	general transcription factor IIIA	0.101	-0.980	0.020
157818159	AAR2	AAR2 splicing factor	0.110	-1.000	0.000
157786720	HIVEP1	HIVEP zinc finger 1	0.111	-0.999	0.001
564360651	LRRC14	leucine rich repeat containing 14	0.115	-0.986	0.014
47847438	EXOC3	exocyst complex component 3	0.118	-0.972	0.028
68163425	TMEM199	transmembrane protein 199	0.118	-0.995	0.005
293344794	FAM160B1	family with sequence similarity 160 member B1	0.121	-0.984	0.016
58866022	MGAT4A	alpha-1,3-mannosyl-glycoprotein 4-beta-N-acetylglucosaminyltransferase A	0.127	-0.970	0.030
672068548	SUPT6H	SPT6 homolog, histone chaperone and transcription elongation factor	0.127	-0.954	0.046
60359978	KIF3C	kinesin family member 3C	0.131	-0.998	0.002
148693260	TIMM29	translocase of inner mitochondrial membrane 29	0.131	-0.970	0.030
157823607	ALDH18A1	aldehyde dehydrogenase 18 family member A1	0.134	-0.972	0.028
61557130	B3GALNT1	beta-1,3-N-acetylgalactosaminyltransferase 1 (globoside blood group)	0.136	-0.975	0.025
157821401	UQCC1	ubiquinol-cytochrome c reductase complex assembly factor 1	0.136	-0.982	0.018

564398139	FYN	FYN proto-oncogene, Src family tyrosine kinase	0.137	-0.952	0.048
58865952	UBAP1	ubiquitin associated protein 1	0.139	-0.965	0.035
9624979	ENSA	endosulfine alpha	0.144	-0.987	0.013
672024670	INSYN2B	inhibitory synaptic factor family member 2B	0.145	-0.993	0.007
157820585	SART3	spliceosome associated factor 3, U4/U6 recycling protein	0.146	-0.981	0.019
149064388	Hmgxb3	HMG-box containing 3	0.147	-0.994	0.006
148665617	NAA50	N-alpha-acetyltransferase 50, NatE catalytic subunit	0.149	-0.953	0.047
157821581	PSMD13	proteasome 26S subunit, non-ATPase 13	0.150	-0.994	0.006
40786455	BPGM	bisphosphoglycerate mutase	0.150	-0.976	0.024
564311031	CLPP	caseinolytic mitochondrial matrix peptidase proteolytic subunit	0.150	-0.974	0.026
208973276	TMEM185A	transmembrane protein 185A	0.151	-0.989	0.011
300253233	LEMD3	LEM domain containing 3	0.154	-0.979	0.021
66730376	Arxes1/Arxes2	adipocyte-related X-chromosome expressed sequence 2	0.155	-0.974	0.026
300793740	TANC2	tetratricopeptide repeat, ankyrin repeat and coiled-coil containing 2	0.158	-0.968	0.032
404312665	DKK3	dickkopf WNT signaling pathway inhibitor 3	0.160	-0.985	0.015
213688411	LPCAT1	lysophosphatidylcholine acyltransferase 1	0.162	-0.962	0.038
149033480	Zfp956	zinc finger protein 956	0.163	-0.994	0.006
672072928	CUX2	cut like homeobox 2	0.164	-0.974	0.026
157822501	MCM3AP	minichromosome maintenance complex component 3 associated protein	0.164	-0.996	0.004
564340867	MMADHC	metabolism of cobalamin associated D	0.165	-0.956	0.044
76559929	NOC2L	NOC2 like nucleolar associated transcriptional repressor	0.165	-0.986	0.014
198278423	IQCJ-SCHIP1	IQCJ-SCHIP1 readthrough	0.166	-0.969	0.031
408772026	Afg3l1	AFG3-like AAA ATPase 1	0.166	-0.954	0.046
55926133	RFC2	replication factor C subunit 2	0.168	-0.967	0.033
157823165	DNAJB1	DnaJ heat shock protein family (Hsp40) member B1	0.171	-0.951	0.049
149044496	PLAA	phospholipase A2 activating protein	0.171	-0.958	0.042
21489987	PCYOX1	prenylcysteine oxidase 1	0.172	-0.971	0.029
33356154	UBE2H	ubiquitin conjugating enzyme E2 H	0.172	-0.998	0.002
56605790	HCFC2	host cell factor C2	0.173	-0.998	0.002

149054120	ORMDL3	ORMDL sphingolipid biosynthesis regulator 3	0.179	-0.979	0.021
61556927	EIF3G	eukaryotic translation initiation factor 3 subunit G	0.180	-0.971	0.029
62079005	SLAIN1	SLAIN motif family member 1	0.180	-0.996	0.004
6981296	NUP50	nucleoporin 50	0.181	-0.969	0.031
157817773	ZNF641	zinc finger protein 641	0.183	-0.972	0.028
401709959	Ppp1cc	protein phosphatase 1 catalytic subunit gamma	0.183	-0.982	0.018
9507007	PTGFRN	prostaglandin F2 receptor inhibitor	0.184	-0.993	0.007
58865626	UBXN4	UBX domain protein 4	0.186	-0.969	0.031
564384443	EIF4ENIF1	eukaryotic translation initiation factor 4E nuclear import factor 1	0.187	-0.980	0.020
167555101	STRADB	STE20 related adaptor beta	0.188	-0.961	0.039
148670058	PRMT6	protein arginine methyltransferase 6	0.188	-0.966	0.034
66730335	SUMO3	small ubiquitin like modifier 3	0.188	-0.984	0.016
149047323	ZNF518B	zinc finger protein 518B	0.190	-0.971	0.029
672085227	USP10	ubiquitin specific peptidase 10	0.191	-0.989	0.011
148683335	SLC25A44	solute carrier family 25 member 44	0.191	-0.977	0.023
197313795	MTX1	metaxin 1	0.192	-1.000	0.000
62079229	PDSS2	decaprenyl diphosphate synthase subunit 2	0.193	-0.961	0.039
296489017	BEND5	BEN domain containing 5	0.196	-0.982	0.018
157821953	NXPE3	neurexophilin and PC-esterase domain family member 3	0.197	-0.967	0.033
213511844	ALG2	ALG2 alpha-1,3/1,6-mannosyltransferase	0.200	-0.998	0.002
281604227	CEP104	centrosomal protein 104	0.203	-0.959	0.041
219277692	NDUFB2	NADH:ubiquinone oxidoreductase subunit B2	0.203	-0.999	0.001
61557021	BFAR	bifunctional apoptosis regulator	0.204	-0.955	0.045
672046840	UBOX5	U-box domain containing 5	0.206	-0.971	0.029
157817674	ATP5MF-PTCD1	ATP5MF-PTCD1 readthrough	0.207	-0.989	0.011
40018540	DDX24	DEAD-box helicase 24	0.208	-0.988	0.012
61557082	TERF2IP	TERF2 interacting protein	0.211	-0.968	0.032
61556748	TSPYL1	TSPY like 1	0.211	-0.982	0.018
58865624	NUF2	NUF2 component of NDC80 kinetochore complex	0.214	-0.956	0.044
639869	CHKA	choline kinase alpha	0.216	-0.956	0.044
189163477	SCAF4	SR-related CTD associated factor 4	0.216	-0.972	0.028
2804296	CDH8	cadherin 8	0.221	-0.972	0.028
148669751	SMNDC1	survival motor neuron domain containing 1	0.223	-0.996	0.004
197252056	MED1	mediator complex subunit 1	0.223	-0.956	0.044

58865962	RNF41	ring finger protein 41	0.231	-0.960	0.040
148670791	ZFYVE1	zinc finger FYVE-type containing 1	0.232	-0.998	0.002
798974764	SRRD	SRR1 domain containing	0.233	-0.974	0.026
564340133	GTF3C4	general transcription factor IIIC subunit 4	0.236	-0.996	0.004
37360264	TRMT6	tRNA methyltransferase 6	0.236	-0.980	0.020
19424174	DNPH1	2'-deoxynucleoside 5'-phosphate N-hydrolase 1	0.243	-0.967	0.033
57527612	SLC17A5	solute carrier family 17 member 5	0.245	-0.968	0.032
158631258	KDSR	3-ketodihydrosphingosine reductase	0.249	-0.969	0.031
291042683	DCAF5	DDB1 and CUL4 associated factor 5	0.252	-0.983	0.017
166157540	TMEM222	transmembrane protein 222	0.253	-0.971	0.029
57164019	B4GALT3	beta-1,4-galactosyltransferase 3	0.256	-0.953	0.047
71361655	MRPL12	mitochondrial ribosomal protein L12	0.257	-0.967	0.033
77627757	IQUB	IQ motif and ubiquitin domain containing	0.257	-0.997	0.003
119388826	TFPT	TCF3 fusion partner	0.258	-1.000	0.000
281427178	CEP76	centrosomal protein 76	0.262	-0.955	0.045
67078454	SLC25A51	solute carrier family 25 member 51	0.263	-0.978	0.022
380877082	NAXE	NAD(P)HX epimerase	0.263	-0.974	0.026
148687213	COX19	cytochrome c oxidase assembly factor COX19	0.264	-0.975	0.025
404312698	GOLM2	golgi membrane protein 2	0.270	-0.979	0.021
564394925	TENT4B	terminal nucleotidyltransferase 4B	0.276	-0.998	0.002
59937915	ARIH2	ariadne RBR E3 ubiquitin protein ligase 2	0.280	-0.985	0.015
157821915	MSANTD3	Myb/SANT DNA binding domain containing 3	0.283	-0.973	0.027
149052738	RGD1561277	RGD1561277	0.285	-0.969	0.031
62078733	MAK16	MAK16 homolog	0.287	-0.994	0.006
672020915	VCPKMT	valosin containing protein lysine methyltransferase	0.288	-0.999	0.001
672053062	FKBP15	FKBP prolyl isomerase 15	0.290	-0.961	0.039
76559919	N4BP3	NEDD4 binding protein 3	0.292	-0.967	0.033
149031601	H1f2	H1.2 linker histone, cluster member	0.294	-0.984	0.016
68163385	GPATCH4	G-patch domain containing 4	0.297	-1.000	0.000
157821997	MED28	mediator complex subunit 28	0.297	-0.980	0.020
74220037	FAM107B	family with sequence similarity 107 member B	0.299	-0.995	0.005
13929168	FAT1	FAT atypical cadherin 1	0.300	-0.954	0.046
157822367	PUS3	pseudouridine synthase 3	0.301	-0.981	0.019
7339838	SUV39H1	suppressor of variegation 3-9 homolog 1	0.302	-0.960	0.040
157819315	OSBPL11	oxysterol binding protein like 11	0.306	-0.978	0.022

300798436	NME6	NME/NM23 nucleoside diphosphate kinase 6	0.308	-0.957	0.043
51491900	TOR1A	torsin family 1 member A	0.308	-0.985	0.015
349501022	2410002F23Rik	RIKEN cDNA 2410002F23 gene	0.309	-0.958	0.042
672043577	Rprd2	regulation of nuclear pre-mRNA domain containing 2	0.312	-0.951	0.049
109480728	TMEM74	transmembrane protein 74	0.313	-0.957	0.043
300793780	ZNF251	zinc finger protein 251	0.321	-0.980	0.020
41386747	ZC3H18	zinc finger CCCH-type containing 18	0.322	-0.952	0.048
672030183	H2AC12	H2A clustered histone 12	0.323	-0.976	0.024
76362828	TEF	TEF transcription factor, PAR bZIP family member	0.327	-0.956	0.044
68163537	NXPE4	neurexophilin and PC-esterase domain family member 4	0.329	-0.983	0.017
157817260	LTO1	LTO1 maturation factor of ABCE1	0.330	-0.959	0.041
564333920	PPRC1	PPARG related coactivator 1	0.333	-0.993	0.007
156627555	NT5C3B	5'-nucleotidase, cytosolic IIIB	0.335	-0.983	0.017
6680007	GJC1	gap junction protein gamma 1	0.336	-0.951	0.049
73990974	LZTS3	leucine zipper tumor suppressor family member 3	0.345	-0.983	0.017
141803183	ZKSCAN3	zinc finger with KRAB and SCAN domains 3	0.351	-0.987	0.013
148673748	FAM110B	family with sequence similarity 110 member B	0.357	-0.996	0.004
157822027	CSRNP2	cysteine and serine rich nuclear protein 2	0.361	-0.975	0.025
300794219	OPN3	opsin 3	0.362	-0.963	0.037
300797828	KAT14	lysine acetyltransferase 14	0.365	-0.995	0.005
148710078	TAF5	TATA-box binding protein associated factor 5	0.366	-0.984	0.016
238859603	ISLR2	immunoglobulin superfamily containing leucine rich repeat 2	0.369	-0.961	0.039
157821875	PTCD2	pentatricopeptide repeat domain 2	0.372	-0.971	0.029
672036551	ZDHHC13	zinc finger DHHC-type palmitoyltransferase 13	0.373	-0.972	0.028
157817720	SLC16A14	solute carrier family 16 member 14	0.374	-0.992	0.008
157821747	MDM2	MDM2 proto-oncogene	0.377	-0.977	0.023
164565364	ITPKB	inositol-trisphosphate 3-kinase B	0.380	-1.000	0.000
148681067	VASH2	vasohibin 2	0.397	-0.970	0.030
40789237	PCDHA4	protocadherin alpha 4	0.398	-0.955	0.045
157821403	RASSF7	Ras association domain family member 7	0.399	-0.974	0.026
157817446	LINGO2	leucine rich repeat and Ig domain containing 2	0.403	-0.951	0.049

282158061	Ttc41	tetratricopeptide repeat domain 41	0.404	-0.986	0.014
62945262	PIK3IP1	phosphoinositide-3-kinase interacting protein 1	0.404	-0.979	0.021
293345066	PPIL6	peptidylprolyl isomerase like 6	0.408	-0.971	0.029
226371633	CABLES1	Cdk5 and Abl enzyme substrate 1	0.409	-0.969	0.031
66730347	PTPRCAP	protein tyrosine phosphatase receptor type C associated protein	0.415	-0.977	0.023
67846052	DCUN1D3	defective in cullin neddylation 1 domain containing 3	0.416	-0.958	0.042
293348214	CCDC88C	coiled-coil domain containing 88C	0.421	-0.952	0.048
56090305	NFATC2IP	nuclear factor of activated T cells 2 interacting protein	0.430	-0.997	0.003
56090289	PELO	pelota mRNA surveillance and ribosome rescue factor	0.432	-0.976	0.024
564395313	OTUD4	OTU deubiquitinase 4	0.434	-0.951	0.049
157823891	ING2	inhibitor of growth family member 2	0.439	-0.985	0.015
564311452	TMEM131	transmembrane protein 131	0.443	-0.972	0.028
51980294	COQ3	coenzyme Q3, methyltransferase	0.452	-0.980	0.020
157820727	RPL27A	ribosomal protein L27a	0.452	-0.957	0.043
53850630	LOC100362724/ MGC95208	similar to 4930453N24Rik protein	0.456	-0.989	0.011
148706598	PKDCC	protein kinase domain containing, cytoplasmic	0.469	-0.992	0.008
51948492	NUDT19	nudix hydrolase 19	0.470	-0.990	0.010
70608121	Dmrtc1a	DMRT-like family C1a	0.479	-0.966	0.034
149024753	DFFB	DNA fragmentation factor subunit beta	0.487	-0.985	0.015
212549645	KIF18A	kinesin family member 18A	0.505	-0.960	0.040
19424300	GCHFR	GTP cyclohydrolase I feedback regulator	0.519	-0.974	0.026
148687591	TMEM132D	transmembrane protein 132D	0.550	-0.987	0.013
213688370	EXOSC7	exosome component 7	0.551	-0.961	0.039
219879771	PGAP3	post-GPI attachment to proteins phospholipase 3	0.560	-0.979	0.021
149023178	CEP152	centrosomal protein 152	0.572	-1.000	0.000
564372912	GPS2	G protein pathway suppressor 2	0.577	-0.989	0.011
148664537	Gm10269	ribosomal protein L35 pseudogene	0.582	-0.984	0.016
555290059	MED7	mediator complex subunit 7	0.584	-0.991	0.009
511094004	RUNX2	RUNX family transcription factor 2	0.585	-0.972	0.028
24415396	GPR3	G protein-coupled receptor 3	0.604	-0.972	0.028
157822359	PELI2	pellino E3 ubiquitin protein ligase family member 2	0.620	-0.971	0.029
197386066	ZNF784	zinc finger protein 784	0.633	-0.980	0.020
157823803	DOK3	docking protein 3	0.639	-0.995	0.005

149016574	ZNF324	zinc finger protein 324	0.689	-0.960	0.040
348041347	CENPL	centromere protein L	0.708	-0.978	0.022
392354293	Hmgb3	high mobility group box 3	0.709	-0.979	0.021
672031995	Kdm6a	lysine demethylase 6A	0.794	-0.960	0.040
564297423	FAM71E1	family with sequence similarity 71 member E1	0.803	-0.960	0.040
564377118	WDR53	WD repeat domain 53	0.807	-0.992	0.008
294979130	FOXP3	forkhead box P3	0.830	-0.977	0.023
672086719	FAM184A	family with sequence similarity 184 member A	0.836	-0.993	0.007
26024223	ABCG5	ATP binding cassette subfamily G member 5	0.841	-0.969	0.031
392339806	CFAP69	cilia and flagella associated protein 69	0.915	-0.959	0.041
9506775	HES2	hes family bHLH transcription factor 2	0.963	-0.972	0.028
148693657	DDX6	DEAD-box helicase 6	0.969	-0.960	0.040
82654234	LILRA6	leukocyte immunoglobulin like receptor A6	1.000	-0.994	0.006
148235584	CLEC4A	C-type lectin domain family 4 member A	1.000	-0.972	0.028
149065466	ARHGEF5	Rho guanine nucleotide exchange factor 5	1.000	-0.972	0.028
404501522	NXNL1	nucleoredoxin like 1	1.037	-0.972	0.028
148674299	Gm14176	ubiquitin-conjugating enzyme E2I pseudogene	1.041	-0.995	0.005
149067796	TMEM219	transmembrane protein 219	1.064	-0.994	0.006
564296988	ZNF235	zinc finger protein 235	1.072	-0.973	0.027
188536090	FAM241B	family with sequence similarity 241 member B	1.127	-0.990	0.010
62078917	PAQR5	progestin and adipoQ receptor family member 5	1.181	-0.969	0.031
149045964	PTH2R	parathyroid hormone 2 receptor	1.222	-0.969	0.031
564317923	SACS	sacsin molecular chaperone	1.308	-0.986	0.014
58865680	CES5A	carboxylesterase 5A	1.585	-0.981	0.019
158533972	SPTA1	spectrin alpha, erythrocytic 1	1.585	-0.972	0.028
149058209	SELE	selectin E	1.585	-0.972	0.028
13928980	AQP3	aquaporin 3 (Gill blood group)	1.585	-0.963	0.037
24308466	ITGB3	integrin subunit beta 3	1.596	-0.992	0.008
157818463	Zfp93	zinc finger protein 93	1.597	-0.997	0.003
672013187	DMWD	DM1 locus, WD repeat containing	1.605	-0.985	0.015
568990288	NIPBL	NIPBL cohesin loading factor	1.705	-0.976	0.024
157816947	GUCA1B	guanylate cyclase activator 1B	1.708	-0.952	0.048
157820841	GP1BA	glycoprotein Ib platelet subunit alpha	1.726	-0.959	0.041
569001477	MTCL1	microtubule crosslinking factor 1	1.726	-0.982	0.018

149046722	IBSP	integrin binding sialoprotein	1.737	-0.963	0.037
149032888	LOC100910237	uncharacterized LOC100910237	1.751	-0.993	0.007
157820135	CHRDL2	chordin like 2	1.762	-0.994	0.006
157816967	Gm4925	predicted gene 4925	1.781	-0.963	0.037
392342449	PRSS56	serine protease 56	1.848	-0.975	0.025
189181736	LAD1	ladinin 1	1.874	-0.987	0.013
16758254	CNGA1	cyclic nucleotide gated channel subunit alpha 1	1.874	-0.974	0.026
109488483	KIAA0753	KIAA0753	1.905	-0.961	0.039
197381585	Urah	urate (5-hydroxyiso-) hydrolase	1.976	-0.972	0.028
160961485	MYLK3	myosin light chain kinase 3	2.000	-0.972	0.028
568979594	SYT16	synaptotagmin 16	2.059	-0.980	0.020
672070295	BAHCC1	BAH domain and coiled-coil containing 1	2.083	-0.960	0.040
28972866	CSMD3	CUB and Sushi multiple domains 3	2.140	-0.985	0.015
568974167	SLC26A11	solute carrier family 26 member 11	2.149	-0.972	0.028
564297852	CRTC3	CREB regulated transcription coactivator 3	2.151	-0.985	0.015
293352381	PAN3	poly(A) specific ribonuclease subunit PAN3	2.239	-0.974	0.026
56912237	KRT28	keratin 28	2.241	-0.974	0.026
71896592	IGFALS	insulin like growth factor binding protein acid labile subunit	2.322	-0.972	0.028
8393941	PADI4	peptidyl arginine deiminase 4	2.322	-0.972	0.028
157819659	RRH	retinal pigment epithelium-derived rhodopsin homolog	2.447	-0.975	0.025
564329376	SRPK3	SRSF protein kinase 3	2.585	-0.972	0.028
58866038	XKRX	XK related X-linked	2.585	-0.972	0.028
11120690	NR1H4	nuclear receptor subfamily 1 group H member 4	2.585	-0.972	0.028
13540693	MYOC	myocilin	2.585	-0.972	0.028
57222314	OAS3	2'-5'-oligoadenylate synthetase 3	2.585	-0.972	0.028
8394529	VDR	vitamin D receptor	2.585	-0.972	0.028
25742760	AMH	anti-Mullerian hormone	2.807	-0.972	0.028
157787002	Dpt	dermatopontin	2.807	-0.972	0.028
197384923	C1orf87	chromosome 1 open reading frame 87	3.000	-0.972	0.028
13591993	MMP9	matrix metallopeptidase 9	3.000	-0.972	0.028
300796937	ESPNL	espin like	3.000	-0.972	0.028
564347547	LOC103690120	probable N-acetyltransferase CML1	3.030	-0.961	0.039
281332212	SH2D4B	SH2 domain containing 4B	3.170	-0.972	0.028
260099641	MSH5	mutS homolog 5	3.170	-0.972	0.028
61556961	THEG	theg spermatid protein	3.322	-0.972	0.028
28174920	RPL17	ribosomal protein L17	3.389	-0.995	0.005

25282405	BPIFA1	BPI fold containing family A member 1	3.459	-0.972	0.028
16758550	BCL2L10	BCL2 like 10	3.459	-0.972	0.028
299473749	C1orf226	chromosome 1 open reading frame 226	3.496	-0.971	0.029
148670929	BATF	basic leucine zipper ATF-like transcription factor	3.700	-0.972	0.028
21245088	Ly6a (includes others)	lymphocyte antigen 6 complex, locus A	3.807	-0.972	0.028
148747510	BAAT	bile acid-CoA:amino acid N-acyltransferase	3.807	-0.972	0.028
158187515	OAZ3	ornithine decarboxylase antizyme 3	3.807	-0.972	0.028
23463315	Cyp2d1/Cyp2d5	cytochrome P450, family 2, subfamily d, polypeptide 1	4.170	-0.955	0.045
8393641	AADAT	aminoadipate aminotransferase	4.248	-0.972	0.028
149038931	CNTRL	centriolin	4.492	-0.975	0.025
149034317	NUCKS1	nuclear casein kinase and cyclin dependent kinase substrate 1	4.833	-0.964	0.036
9506733	GJB5	gap junction protein beta 5	4.907	-0.972	0.028
293349510	STAC	SH3 and cysteine rich domain	4.954	-0.956	0.044
16758218	Hamp	hepcidin antimicrobial peptide	5.267	-0.969	0.031
157818205	NOC3L	NOC3 like DNA replication regulator	5.833	-0.965	0.035
672052120	RBM12B	RNA binding motif protein 12B	6.366	-0.973	0.027
293347435	PTPRD	protein tyrosine phosphatase receptor type D	7.710	-0.963	0.037
109472884	UBE3C	ubiquitin protein ligase E3C	8.197	-0.967	0.033

Supplementary Table S12. The list of genes that are differentially expressed in the offspring hippocampus in response to prenatal BPA exposure that exhibited the changes in the expression levels correlated with the neurite length of primary hippocampal cells at DIV3. The transcriptome profiling data of DEGs in male and female rat offspring prenatally exposed to BPA (n = 6, male pups n = 3 and female pups n = 3, from independent litters) or the vehicle control (n = 6, male pups n = 3 and female pups n = 3, from independent litters) were obtained and used for the PTM analyses to identify DEGs that exhibited the changes in the expression levels correlated with the neurite length of primary hippocampal cells at DIV3.

ID	Symbol	Entrez Gene Name	log2(FC)	R values	P-values
18543363	FGF22	fibroblast growth factor 22	-1.830	-0.953	0.047
31745164	HAX1	HCLS1 associated protein X-1	-1.703	-0.966	0.034
564301284	Ttf1	transcription termination factor, RNA polymerase I	-1.633	-0.976	0.024
840084406	Lypd2	Ly6/Plaur domain containing 2	-1.506	-0.967	0.033
210032999	MBOAT4	membrane bound O-acyltransferase domain containing 4	-1.441	-0.994	0.006
672065933	DOCK10	dedicator of cytokinesis 10	-1.436	-0.992	0.008
157818865	INCA1	inhibitor of CDK, cyclin A1 interacting protein 1	-1.433	-0.955	0.045
568994911	PHLDB2	pleckstrin homology like domain family B member 2	-1.350	-0.998	0.002
157786628	ALOX12	arachidonate 12-lipoxygenase, 12S type	-1.322	-0.985	0.015
149047788	ACVR1C	activin A receptor type 1C	-1.322	-0.993	0.007
1236083	LSR	lipolysis stimulated lipoprotein receptor	-1.290	-0.988	0.012
61556810	ADPRHL1	ADP-ribosylhydrolase like 1	-1.285	-0.998	0.002
816197606	HTR6	5-hydroxytryptamine receptor 6	-1.283	-0.975	0.025
162287073	CRYBB1	crystallin beta B1	-1.263	-0.999	0.001
224500890	ARMH1	armadillo like helical domain containing 1	-1.209	-0.978	0.022
50979278	IL22RA2	interleukin 22 receptor subunit alpha 2	-1.202	-0.995	0.005
62945350	C4orf36	chromosome 4 open reading frame 36	-1.198	-1.000	0.000
11968076	RHCE/RHD	Rh blood group D antigen	-1.158	-0.976	0.024
282721071	Iqca1l	IQ motif containing with AAA domain 1 like	-1.138	-1.000	0.000
57527560	TMEM140	transmembrane protein 140	-1.128	-0.979	0.021
67078444	EPN3	epsin 3	-1.090	-0.975	0.025
157817410	ZNF474	zinc finger protein 474	-1.034	-0.967	0.033
564296180	CEP72	centrosomal protein 72	-1.005	-0.989	0.011
149053793	TSPOAP1	TSPO associated protein 1	-0.970	-0.987	0.013

148675846	FAM114A2	family with sequence similarity 114 member A2	-0.814	-0.970	0.030
564342320	FSIP1	fibrous sheath interacting protein 1	-0.813	-0.960	0.040
149042883	LOC100365365	rCG32328-like	-0.812	-0.954	0.046
672045595	RIF1	replication timing regulatory factor 1	-0.799	-1.000	0.000
199561637	PSD4	pleckstrin and Sec7 domain containing 4	-0.794	-0.977	0.023
157818565	CFAP126	cilia and flagella associated protein 126	-0.775	-0.980	0.020
55741827	TERT	telomerase reverse transcriptase	-0.771	-0.980	0.020
197386131	Acad10	acyl-CoA dehydrogenase family, member 10	-0.768	-0.976	0.024
564298020	KCTD14	potassium channel tetramerization domain containing 14	-0.767	-0.953	0.047
194473640	PHETA2	PH domain containing endocytic trafficking adaptor 2	-0.746	-0.996	0.004
53850642	AKAP3	A-kinase anchoring protein 3	-0.737	-0.967	0.033
148701845	RACK1	receptor for activated C kinase 1	-0.710	-0.990	0.010
54312100	DNAJB13	DnaJ heat shock protein family (Hsp40) member B13	-0.703	-0.972	0.028
62644808	ADAMTSL2	ADAMTS like 2	-0.699	-0.974	0.026
189491877	MYADML2	myeloid associated differentiation marker like 2	-0.671	-0.994	0.006
157817426	MISP	mitotic spindle positioning	-0.652	-0.967	0.033
187469467	SMPD5	sphingomyelin phosphodiesterase 5, pseudogene	-0.642	-0.974	0.026
401709944	MPP7	membrane palmitoylated protein 7	-0.603	-0.968	0.032
28461161	LDLR	low density lipoprotein receptor	-0.591	-0.966	0.034
21245102	PLPP2	phospholipid phosphatase 2	-0.589	-0.957	0.043
7549765	HK2	hexokinase 2	-0.563	-1.000	0.000
57114344	UHRF1	ubiquitin like with PHD and ring finger domains 1	-0.558	-0.986	0.014
293349510	STAC	SH3 and cysteine rich domain	-0.550	-0.958	0.042
564369844	NEWGENE_1308624	sialidase 4	-0.534	-0.956	0.044
254675172	AK7	adenylate kinase 7	-0.531	-0.954	0.046
157822527	Prorsd1	prolyl-tRNA synthetase domain containing 1	-0.527	-0.987	0.013
157822879	EFS	embryonal Fyn-associated substrate	-0.523	-0.960	0.040
61556921	UBXN10	UBX domain protein 10	-0.515	-0.987	0.013
145207953	PLAU	plasminogen activator, urokinase	-0.491	-0.966	0.034
7106349	LYNX1	Ly6/neurotoxin 1	-0.487	-0.963	0.037
293350806	ZRSR2	zinc finger CCCH-type, RNA binding motif and serine/arginine rich 2	-0.485	-0.976	0.024

157786894	PYCR1	pyrroline-5-carboxylate reductase 1	-0.485	-0.981	0.019
58865450	BAG3	BAG cochaperone 3	-0.484	-0.976	0.024
761631363	EPGN	epithelial mitogen	-0.479	-0.979	0.021
58865984	TRAF3IP1	TRAF3 interacting protein 1	-0.471	-0.977	0.023
589269168	WDR34	WD repeat domain 34	-0.467	-0.978	0.022
16758600	RGS14	regulator of G protein signaling 14	-0.457	-0.977	0.023
121722562	CA9	carbonic anhydrase 9	-0.455	-0.996	0.004
13562118	LRP2	LDL receptor related protein 2	-0.454	-0.964	0.036
62945352	C4orf19	chromosome 4 open reading frame 19	-0.452	-0.957	0.043
149026322	PTGER3	prostaglandin E receptor 3	-0.445	-0.956	0.044
62078835	TTLL9	tubulin tyrosine ligase like 9	-0.439	-0.996	0.004
71361639	GLI4	GLI family zinc finger 4	-0.437	-0.961	0.039
56605988	FANK1	fibronectin type III and ankyrin repeat domains 1	-0.431	-0.961	0.039
157787135	DUSP10	dual specificity phosphatase 10	-0.428	-0.956	0.044
149061527	PGGHG	protein-glucosylgalactosylhydroxylysine glucosidase	-0.423	-0.985	0.015
293344558	PCNX3	pecanex 3	-0.420	-0.993	0.007
58743349	FAM89A	family with sequence similarity 89 member A	-0.419	-0.953	0.047
73746573	TGFB1I1	transforming growth factor beta 1 induced transcript 1	-0.411	-0.957	0.043
672023055	TLN2	talin 2	-0.408	-0.968	0.032
198278450	CPT1C	carnitine palmitoyltransferase 1C	-0.406	-0.961	0.039
62078719	HAUS4	HAUS augmin like complex subunit 4	-0.397	-0.964	0.036
164663795	KCNB2	potassium voltage-gated channel subfamily B member 2	-0.388	-0.976	0.024
16758268	TEKT1	tektin 1	-0.386	-0.993	0.007
71043764	C20orf27	chromosome 20 open reading frame 27	-0.384	-0.989	0.011
158081747	PDGFB	platelet derived growth factor subunit B	-0.384	-0.953	0.047
157820337	GGCT	gamma-glutamylcyclotransferase	-0.383	-0.980	0.020
71043890	SMPDL3B	sphingomyelin phosphodiesterase acid like 3B	-0.379	-0.962	0.038
564370911	CHTF18	chromosome transmission fidelity factor 18	-0.378	-0.987	0.013
157817249	CDC14B	cell division cycle 14B	-0.377	-0.973	0.027
672037039	TTC23	tetratricopeptide repeat domain 23	-0.375	-0.991	0.009
205235	Slc6a7	solute carrier family 6 member 7	-0.375	-0.951	0.049
162287337	APOE	apolipoprotein E	-0.375	-0.954	0.046
25282445	ENTPD2	ectonucleoside triphosphate diphosphohydrolase 2	-0.375	-0.950	0.050

157822743	KIF20A	kinesin family member 20A	-0.374	-0.964	0.036
257900470	B3GALT4	beta-1,3-galactosyltransferase 4	-0.372	-0.976	0.024
392337475	SIX5	SIX homeobox 5	-0.371	-0.993	0.007
568970276	SH3PXD2B*	SH3 and PX domains 2B	-0.369	-0.986	0.014
157824128	SOX17	SRY-box transcription factor 17	-0.367	-0.956	0.044
672028474	CDH24	cadherin 24	-0.367	-0.995	0.005
398650618	MMP11	matrix metallopeptidase 11	-0.361	-0.969	0.031
939319594	CPNE7	copine 7	-0.350	-0.999	0.001
55741540	KATNAL1	katanin catalytic subunit A1 like 1	-0.345	-0.964	0.036
568956384	ADAMTS18	ADAM metallopeptidase with thrombospondin type 1 motif 18	-0.345	-0.960	0.040
157819433	MAP6D1	MAP6 domain containing 1	-0.344	-0.988	0.012
11024664	LTBP1	latent transforming growth factor beta binding protein 1	-0.342	-0.972	0.028
160333093	TPRG1L	tumor protein p63 regulated 1 like	-0.340	-0.983	0.017
60360648	KLHL2	kelch like family member 2	-0.338	-0.967	0.033
300797558	ROPN1L	rhophilin associated tail protein 1 like	-0.337	-0.972	0.028
564317005	TBC1D1*	TBC1 domain family member 1	-0.335	-0.996	0.004
672073977	LOC103690089	pleckstrin homology domain-containing family A member 6-like	-0.334	-0.984	0.016
77539756	MED24	mediator complex subunit 24	-0.331	-0.976	0.024
157823597	SLC39A5	solute carrier family 39 member 5	-0.331	-0.985	0.015
84781644	TMEM176A	transmembrane protein 176A	-0.329	-0.957	0.043
58865810	NAGA	alpha-N-acetylgalactosaminidase	-0.328	-0.990	0.010
157822761	MICAL1	microtubule associated monooxygenase, calponin and LIM domain containing 1	-0.328	-0.989	0.011
157787183	KCND1	potassium voltage-gated channel subfamily D member 1	-0.328	-0.965	0.035
18266696	PDE7B	phosphodiesterase 7B	-0.324	-0.957	0.043
672026392	PNPLA6	patatin like phospholipase domain containing 6	-0.318	-0.965	0.035
672043401	POGZ	pogo transposable element derived with ZNF domain	-0.317	-0.955	0.045
149052198	NPRL3	NPR3 like, GATOR1 complex subunit	-0.315	-0.967	0.033
158631250	HAUS8	HAUS augmin like complex subunit 8	-0.315	-0.967	0.033
210033118	COG1	component of oligomeric golgi complex 1	-0.311	-0.964	0.036
63101489	ACHE	acetylcholinesterase (Cartwright blood group)	-0.310	-0.958	0.042
157823815	ILVBL	ilvB acetolactate synthase like	-0.310	-0.967	0.033
171847060	TTC8	tetratricopeptide repeat domain 8	-0.306	-0.991	0.009

149024818	MIB2	mindbomb E3 ubiquitin protein ligase 2	-0.300	-0.992	0.008
21245116	Nradd	neurotrophin receptor associated death domain	-0.299	-0.991	0.009
158635969	FLAD1	flavin adenine dinucleotide synthetase 1	-0.299	-0.975	0.025
210031334	NGEF	neuronal guanine nucleotide exchange factor	-0.298	-0.970	0.030
149024084	COL16A1	collagen type XVI alpha 1 chain	-0.296	-0.995	0.005
83025052	ANKS6	ankyrin repeat and sterile alpha motif domain containing 6	-0.296	-0.962	0.038
51036684	G6PC3	glucose-6-phosphatase catalytic subunit 3	-0.296	-0.963	0.037
403048729	PAQR9	progestin and adipoQ receptor family member 9	-0.294	-0.954	0.046
164519052	ARSA	arylsulfatase A	-0.292	-0.978	0.022
197313640	TMEM132E	transmembrane protein 132E	-0.290	-0.976	0.024
310772205	MAP7	microtubule associated protein 7	-0.289	-0.984	0.016
9507083	SEMA4F	ssemaphorin 4F	-0.286	-0.968	0.032
16758138	POMT1	protein O-mannosyltransferase 1	-0.285	-0.965	0.035
300669604	ADAM15	ADAM metallopeptidase domain 15	-0.285	-0.971	0.029
564361358	PPARA	peroxisome proliferator activated receptor alpha	-0.280	-0.964	0.036
402747140	SPN	sialophorin	-0.279	-0.993	0.007
72255507	CD68	CD68 molecule	-0.278	-0.955	0.045
56090612	CDCA3	cell division cycle associated 3	-0.276	-0.983	0.017
213972556	OXSM	3-oxoacyl-ACP synthase, mitochondrial	-0.273	-0.961	0.039
197387642	ZNF710	zinc finger protein 710	-0.270	-0.985	0.015
300795283	SHROOM4	shroom family member 4	-0.269	-0.954	0.046
10242377	GRIK4	glutamate ionotropic receptor kainate type subunit 4	-0.267	-0.961	0.039
157786622	ALOXE3	arachidonate lipoxygenase 3	-0.266	-0.987	0.013
672083937	TSHZ1	teashirt zinc finger homeobox 1	-0.264	-0.992	0.008
157821525	GLYCTK	glycerate kinase	-0.261	-0.958	0.042
157821191	CHST11	carbohydrate sulfotransferase 11	-0.259	-0.956	0.044
21245094	MAN2C1	mannosidase alpha class 2C member 1	-0.256	-0.951	0.049
255708437	PIK3CD	phosphatidylinositol-4,5-bisphosphate 3-kinase catalytic subunit delta	-0.255	-0.991	0.009
9507167	SYNGR1	synaptogyrin 1	-0.254	-0.969	0.031
57164145	NT5DC2	5'-nucleotidase domain containing 2	-0.254	-0.987	0.013
564346652	ZNF775	zinc finger protein 775	-0.253	-0.963	0.037
157823169	LRRC61	leucine rich repeat containing 61	-0.250	-0.964	0.036

59858990	UNC13A	unc-13 homolog A	-0.246	-0.964	0.036
54312094	DAGLA	diacylglycerol lipase alpha	-0.244	-0.972	0.028
164607158	PTPRR	protein tyrosine phosphatase receptor type R	-0.244	-0.979	0.021
67078466	CYP2U1	cytochrome P450 family 2 subfamily U member 1	-0.244	-0.979	0.021
13095924	DRP2	dystrophin related protein 2	-0.242	-0.990	0.010
299829287	DISP2	dispatched RND transporter family member 2	-0.242	-0.957	0.043
54035529	SS18	SS18 subunit of BAF chromatin remodeling complex	-0.241	-0.955	0.045
6978513	APEH	acylaminoacyl-peptide hydrolase	-0.240	-0.981	0.019
564342542	MAP1A	microtubule associated protein 1A	-0.236	-0.971	0.029
149016209	SLC4A3	solute carrier family 4 member 3	-0.234	-0.982	0.018
62655853	TELO2	telomere maintenance 2	-0.228	-0.966	0.034
56799390	ATP1B2	ATPase Na+/K+ transporting subunit beta 2	-0.226	-0.980	0.020
209529673	PLEKHG2	pleckstrin homology and RhoGEF domain containing G2	-0.222	-0.951	0.049
62945338	EIF2D	eukaryotic translation initiation factor 2D	-0.221	-0.993	0.007
197927395	CCDC40	coiled-coil domain containing 40	-0.220	-0.987	0.013
148690402	SLC9A3R2	SLC9A3 regulator 2	-0.220	-0.965	0.035
57527353	TOR3A	torsin family 3 member A	-0.219	-0.959	0.041
402794954	MINK1	misshapen like kinase 1	-0.219	-0.955	0.045
564387660	UBAC2	UBA domain containing 2	-0.217	-0.983	0.017
38174623	FXYD7	FXYD domain containing ion transport regulator 7	-0.215	-0.960	0.040
827475641	CCDC151	coiled-coil domain containing 151	-0.213	-0.980	0.020
29789305	PTPRN	protein tyrosine phosphatase receptor type N	-0.212	-0.976	0.024
61557385	RNASEH2A	ribonuclease H2 subunit A	-0.207	-0.986	0.014
62078695	MLEC	malectin	-0.206	-0.983	0.017
402794666	NRG1	neuregulin 1	-0.205	-0.968	0.032
31377525	ASL	argininosuccinate lyase	-0.205	-0.995	0.005
206558322	JMJD8	jumonji domain containing 8	-0.205	-0.969	0.031
403310680	GAMT	guanidinoacetate N-methyltransferase	-0.205	-0.980	0.020
188536071	DLC1	DLC1 Rho GTPase activating protein	-0.204	-0.978	0.022
564361015	TRIOBP	TRIO and F-actin binding protein	-0.204	-0.999	0.001
149046617	MAGI2	membrane associated guanylate kinase, WW and PDZ domain containing 2	-0.202	-0.970	0.030
293340174	DNAH9	dynein axonemal heavy chain 9	-0.202	-0.998	0.002
149024371	SH2D5	SH2 domain containing 5	-0.197	-0.989	0.011

840088206	INTS11	integrator complex subunit 11	-0.194	-0.988	0.012
568941844	IQSEC3	IQ motif and Sec7 domain ArfGEF 3	-0.194	-0.963	0.037
564325125	PLEKHG1	pleckstrin homology and RhoGEF domain containing G1	-0.193	-0.959	0.041
198278525	RIC8A	RIC8 guanine nucleotide exchange factor A	-0.192	-0.979	0.021
56090379	POMGNT1	protein O-linked mannose N-acetylglucosaminyltransferase 1 (beta 1,2-)	-0.191	-0.962	0.038
157821335	GPR162	G protein-coupled receptor 162	-0.191	-0.969	0.031
149027971	ATF6B	activating transcription factor 6 beta	-0.189	-0.995	0.005
568950414	ATXN2L	ataxin 2 like	-0.189	-0.974	0.026
72255531	EFHD2	EF-hand domain family member D2	-0.189	-0.965	0.035
149063212	TMEM132C	transmembrane protein 132C	-0.187	-0.992	0.008
149046124	SPATS2L	spermatogenesis associated serine rich 2 like	-0.186	-0.972	0.028
169234826	ELP4	elongator acetyltransferase complex subunit 4	-0.186	-0.970	0.030
52138739	HEXA	hexosaminidase subunit alpha	-0.185	-0.995	0.005
285026506	IDUA	alpha-L-iduronidase	-0.185	-0.988	0.012
201025393	TTC7A	tetratricopeptide repeat domain 7A	-0.180	-0.987	0.013
149051484	AKAP5	A-kinase anchoring protein 5	-0.180	-0.966	0.034
169642755	Pms2	PMS1 homolog 2, mismatch repair system component	-0.179	-0.991	0.009
672042162	TRPC3	transient receptor potential cation channel subfamily C member 3	-0.178	-0.981	0.019
18677755	ADGRL3	adhesion G protein-coupled receptor L3	-0.177	-0.983	0.017
166063985	PKN1	protein kinase N1	-0.175	-0.963	0.037
77628157	ST18	ST18 C2H2C-type zinc finger transcription factor	-0.172	-0.987	0.013
149019021	Sh3bgrl2	SH3 domain binding glutamate-rich protein like 2	-0.170	-0.988	0.012
149028240	Fsd1	fibronectin type III and SPRY domain containing 1	-0.170	-0.980	0.020
149030303	ELP3	elongator acetyltransferase complex subunit 3	-0.170	-0.994	0.006
16758188	SLC7A8	solute carrier family 7 member 8	-0.169	-0.965	0.035
9506591	FDFT1	farnesyl-diphosphate farnesyltransferase 1	-0.166	-0.984	0.016
62945358	SCFD2	sec1 family domain containing 2	-0.165	-0.962	0.038
149062946	EPHB4	EPH receptor B4	-0.165	-0.991	0.009
568992887	PRPF40B	pre-mRNA processing factor 40 homolog B	-0.165	-0.956	0.044

197245729	CPSF1	cleavage and polyadenylation specific factor 1	-0.163	-0.978	0.022
67846010	ROGDI	rogdi atypical leucine zipper	-0.163	-0.956	0.044
109488292	POLR2A	RNA polymerase II subunit A	-0.161	-0.975	0.025
55741778	SMPD1	sphingomyelin phosphodiesterase 1	-0.153	-0.955	0.045
564388617	KXD1	KxDL motif containing 1	-0.150	-0.959	0.041
564370968	MAPK8IP3	mitogen-activated protein kinase 8 interacting protein 3	-0.150	-0.998	0.002
564388440	MYO9B	myosin IXB	-0.148	-0.952	0.048
564321893	ICOSLG/LOC102723996	inducible T cell costimulator ligand	-0.145	-0.987	0.013
564383481	SRD5A3	steroid 5 alpha-reductase 3	-0.142	-0.969	0.031
300793996	TCHP	trichoplein keratin filament binding	-0.141	-0.991	0.009
281485565	RASGRF1	Ras protein specific guanine nucleotide releasing factor 1	-0.136	-0.985	0.015
157817708	PPP6R2	protein phosphatase 6 regulatory subunit 2	-0.134	-0.990	0.010
40288195	GABRG3	gamma-aminobutyric acid type A receptor subunit gamma3	-0.134	-0.985	0.015
672080550	TDRP	testis development related protein	-0.133	-0.969	0.031
62078691	Lrwd1	leucine-rich repeats and WD repeat domain containing 1	-0.132	-0.998	0.002
20302117	FNBP1	formin binding protein 1	-0.132	-0.985	0.015
50510357	GPD1L	glycerol-3-phosphate dehydrogenase 1 like	-0.132	-0.960	0.040
66730427	CENPT	centromere protein T	-0.129	-0.991	0.009
399498531	NDRG2	NDRG family member 2	-0.123	-0.969	0.031
564326713	ZC3H4	zinc finger CCCH-type containing 4	-0.121	-0.965	0.035
157817346	ATP9B	ATPase phospholipid transporting 9B (putative)	-0.121	-0.975	0.025
197387125	CCSER1	coiled-coil serine rich protein 1	-0.118	-0.953	0.047
672041232	PDE8B	phosphodiesterase 8B	-0.118	-0.951	0.049
399124797	KIFC2	kinesin family member C2	-0.114	-0.971	0.029
56912225	CIAO1	cytosolic iron-sulfur assembly component 1	-0.114	-0.986	0.014
157823591	MTMR14	myotubularin related protein 14	-0.113	-0.968	0.032
164663858	VAV2	vav guanine nucleotide exchange factor 2	-0.104	-0.975	0.025
13928818	PTPRN2	protein tyrosine phosphatase receptor type N2	-0.102	-0.970	0.030
564368924	STK16	serine/threonine kinase 16	-0.101	-0.987	0.013
61889092	AK1	adenylate kinase 1	-0.099	-0.975	0.025
157786742	HIC2	HIC ZBTB transcriptional repressor 2	-0.097	-0.985	0.015
734703982	SAFB2	scaffold attachment factor B2	-0.095	-0.972	0.028

197927388	GART	phosphoribosylglycinamide formyltransferase, phosphoribosylglycinamide synthetase, phosphoribosylaminoimidazole synthetase	-0.094	-0.963	0.037
46485440	GPI	glucose-6-phosphate isomerase	-0.093	-0.966	0.034
403420618	NUMA1	nuclear mitotic apparatus protein 1	-0.090	-0.989	0.011
6978465	GRK2	G protein-coupled receptor kinase 2	-0.089	-0.959	0.041
564350262	CHD7	chromodomain helicase DNA binding protein 7	-0.088	-0.959	0.041
564370751	HCFC1R1	host cell factor C1 regulator 1	-0.086	-0.954	0.046
157817492	PAK6	p21 (RAC1) activated kinase 6	-0.072	-0.985	0.015
13786182	FADS2	fatty acid desaturase 2	-0.066	-0.993	0.007
149017294	Pcdhb7	protocadherin beta 7	-0.060	-0.999	0.001
149040371	XPNPEP1	X-prolyl aminopeptidase 1	-0.059	-0.985	0.015
157822951	SPIRE1	spire type actin nucleation factor 1	-0.055	-0.985	0.015
149065158	AHCYL2	adenosylhomocysteinase like 2	-0.052	-0.957	0.043
28189917	Ubb	ubiquitin B	-0.043	-0.956	0.044
57527209	UBA5	ubiquitin like modifier activating enzyme 5	-0.025	-0.998	0.002
74185161	PSMC3	proteasome 26S subunit, ATPase 3	-0.018	-0.995	0.005
564301354	PRRC2B	proline rich coiled-coil 2B	0.015	0.986	0.014
157820811	PCDHB14	protocadherin beta 14	0.015	0.968	0.032
4507133	SNRPG	small nuclear ribonucleoprotein polypeptide G	0.037	0.954	0.046
62945264	IST1	IST1 factor associated with ESCRT-III	0.042	0.994	0.006
22129759	ZWINT	ZW10 interacting kinetochore protein	0.045	0.993	0.007
183985961	MAN1B1	mannosidase alpha class 1B member 1	0.046	0.968	0.032
29789096	PRKAR2A	protein kinase cAMP-dependent type II regulatory subunit alpha	0.047	0.976	0.024
157821879	ATF6	activating transcription factor 6	0.067	0.995	0.005
111494225	SELENOF	selenoprotein F	0.068	0.999	0.001
68163473	THEM4	thioesterase superfamily member 4	0.069	0.972	0.028
564347292	ST3GAL5	ST3 beta-galactoside alpha-2,3-sialyltransferase 5	0.071	0.954	0.046
392338110	TBC1D12	TBC1 domain family member 12	0.071	0.988	0.012
40018592	HP1BP3	heterochromatin protein 1 binding protein 3	0.077	0.962	0.038
109692276	UBE2E3	ubiquitin conjugating enzyme E2 E3	0.078	0.975	0.025
149032182	TMEM106C	transmembrane protein 106C	0.081	0.971	0.029

58865982	PIGC	phosphatidylinositol glycan anchor biosynthesis class C	0.084	0.981	0.019
40786445	GET1	guided entry of tail-anchored proteins factor 1	0.089	0.998	0.002
189163515	USP47	ubiquitin specific peptidase 47	0.089	0.972	0.028
62078823	RMDN3	regulator of microtubule dynamics 3	0.090	0.998	0.002
281604140	THUMPD3	THUMP domain containing 3	0.091	0.962	0.038
12621140	PIK3C3	phosphatidylinositol 3-kinase catalytic subunit type 3	0.100	0.953	0.047
28972173	KIF3B	kinesin family member 3B	0.103	0.986	0.014
148697875	GDI1	GDP dissociation inhibitor 1	0.103	0.994	0.006
80751173	PCDHGA1	protocadherin gamma subfamily A, 1	0.106	0.987	0.013
564377243	OPA1	OPA1 mitochondrial dynamin like GTPase	0.107	0.954	0.046
672050419	SLC6A6	solute carrier family 6 member 6	0.110	0.971	0.029
38454206	PSMD6	proteasome 26S subunit, non-ATPase 6	0.110	0.990	0.010
13928860	IGBP1	immunoglobulin binding protein 1	0.112	0.956	0.044
404501464	IFNAR1	interferon alpha and beta receptor subunit 1	0.119	0.971	0.029
157822659	RIOK3	RIO kinase 3	0.122	0.996	0.004
393716310	WAC	WW domain containing adaptor with coiled-coil	0.124	0.989	0.011
162287304	CDC42EP3	CDC42 effector protein 3	0.124	0.990	0.010
25742623	UGCG	UDP-glucose ceramide glucosyltransferase	0.125	0.954	0.046
148677380	MSANTD4	Myb/SANT DNA binding domain containing 4 with coiled-coils	0.129	0.994	0.006
148747275	MCM7	minichromosome maintenance complex component 7	0.130	0.969	0.031
68299787	TAF9	TATA-box binding protein associated factor 9	0.131	0.988	0.012
83582792	FAM117B	family with sequence similarity 117 member B	0.133	0.962	0.038
158749598	LRIG2	leucine rich repeats and immunoglobulin like domains 2	0.133	0.956	0.044
564397283	FKBP5	FKBP prolyl isomerase 5	0.134	0.998	0.002
14010879	PSMD1	proteasome 26S subunit, non-ATPase 1	0.136	0.996	0.004
61556945	MOAP1	modulator of apoptosis 1	0.136	0.977	0.023
18266706	ELP1	elongator complex protein 1	0.137	0.968	0.032
71043634	PDCL3	phosducin like 3	0.137	0.957	0.043
157821921	QSOX2	quiescin sulfhydryl oxidase 2	0.141	0.953	0.047
71043834	RBMX	RNA binding motif protein X-linked	0.143	0.961	0.039

672056381	PCNX4	pecanex 4	0.143	0.987	0.013
55715816	GLYR1	glyoxylate reductase 1 homolog	0.144	0.962	0.038
30017419	NREP	neuronal regeneration related protein	0.145	0.988	0.012
399124804	SLC4A8	solute carrier family 4 member 8	0.146	0.965	0.035
157818165	SMURF2	SMAD specific E3 ubiquitin protein ligase 2	0.148	0.980	0.020
403225011	SAMHD1	SAM and HD domain containing deoxynucleoside triphosphate triphosphohydrolase 1	0.148	1.000	0.000
149022577	FNBP4	formin binding protein 4	0.149	0.997	0.003
157819885	SETD5	SET domain containing 5	0.150	0.963	0.037
198278505	RPL7	ribosomal protein L7	0.151	0.999	0.001
157817718	DCAF10	DDB1 and CUL4 associated factor 10	0.153	0.968	0.032
402692225	RNF168	ring finger protein 168	0.156	0.986	0.014
197927166	AGPAT5	1-acylglycerol-3-phosphate O-acyltransferase 5	0.157	0.992	0.008
41054820	CHRDL1	chordin like 1	0.157	0.994	0.006
197333849	ATP23	ATP23 metallopeptidase and ATP synthase assembly factor homolog	0.158	0.958	0.042
170016030	DDX31	DEAD-box helicase 31	0.161	0.960	0.040
672042487	NBEA	neurobeachin	0.163	0.998	0.002
70794797	USP3	ubiquitin specific peptidase 3	0.164	0.960	0.040
158854035	RNF146	ring finger protein 146	0.166	0.998	0.002
672028565	Setdb2	SET domain bifurcated histone lysine methyltransferase 2	0.168	0.952	0.048
56605826	LAMTOR3	late endosomal/lysosomal adaptor, MAPK and MTOR activator 3	0.168	0.973	0.027
148707518	RNF2	ring finger protein 2	0.171	0.967	0.033
157823942	COMMD2	COMM domain containing 2	0.172	0.973	0.027
47155561	DNAJC7	DnaJ heat shock protein family (Hsp40) member C7	0.173	0.999	0.001
157786908	ZBED5	zinc finger BED-type containing 5	0.173	0.960	0.040
60360266	PPP2R2A	protein phosphatase 2 regulatory subunit Balpha	0.175	0.980	0.020
564382144	LIN9	lin-9 DREAM MuvB core complex component	0.176	0.959	0.041
209915609	PRICKLE1	prickle planar cell polarity protein 1	0.177	0.997	0.003
149064951	DYNC1I1	dynein cytoplasmic 1 intermediate chain 1	0.189	0.967	0.033
50511227	ZBTB34	zinc finger and BTB domain containing 34	0.190	0.992	0.008
19705519	AAGAB	alpha and gamma adaptin binding protein	0.191	0.984	0.016

67078422	TMX1	thioredoxin related transmembrane protein 1	0.191	0.981	0.019
300795677	IL12RB2	interleukin 12 receptor subunit beta 2	0.192	0.967	0.033
149067040	LIN7A	lin-7 homolog A, crumbs cell polarity complex component	0.196	0.967	0.033
148674377	OSER1	oxidative stress responsive serine rich 1	0.197	0.970	0.030
171846592	GPBP1	GC-rich promoter binding protein 1	0.198	0.962	0.038
28174943	RPL24	ribosomal protein L24	0.200	0.973	0.027
148667150	ZNF22	zinc finger protein 22	0.202	0.991	0.009
4506681	RPS11	ribosomal protein S11	0.203	0.966	0.034
148702333	DDX42	DEAD-box helicase 42	0.205	0.951	0.049
672024181	OBSL1	obscurin like cytoskeletal adaptor 1	0.207	0.952	0.048
210147441	ATXN7L3B	ataxin 7 like 3B	0.211	0.970	0.030
157823667	UTP15	UTP15 small subunit processome component	0.212	0.955	0.045
207318	TMSB10/TMSB4X	thymosin beta 4 X-linked	0.213	0.988	0.012
157822663	RAB22A	RAB22A, member RAS oncogene family	0.214	0.969	0.031
42627869	VKORC1L1	vitamin K epoxide reductase complex subunit 1 like 1	0.221	0.981	0.019
149030883	GSS	glutathione synthetase	0.222	0.952	0.048
564340181	SETX	senataxin	0.223	0.969	0.031
6978787	DYRK1A	dual specificity tyrosine phosphorylation regulated kinase 1A	0.228	0.956	0.044
187937036	ZC3HC1	zinc finger C3HC-type containing 1	0.234	0.950	0.050
158636018	LRRC6	leucine rich repeat containing 6	0.240	0.958	0.042
157820771	ZNRF2	zinc and ring finger 2	0.248	0.971	0.029
189491614	SLC25A46	solute carrier family 25 member 46	0.249	0.957	0.043
58865438	TRIP13	thyroid hormone receptor interactor 13	0.250	0.970	0.030
672028116	TAPT1	transmembrane anterior posterior transformation 1	0.259	0.986	0.014
425384	CAMK4	calcium/calmodulin dependent protein kinase IV	0.262	0.960	0.040
6693638	MORC3	MORC family CW-type zinc finger 3	0.269	0.984	0.016
157819221	NAA30	N-alpha-acetyltransferase 30, NatC catalytic subunit	0.286	0.979	0.021
585866350	PEMT	phosphatidylethanolamine N-methyltransferase	0.286	0.990	0.010
206734	RPL5	ribosomal protein L5	0.290	0.996	0.004
149064973	ASNS	asparagine synthetase (glutamine-hydrolyzing)	0.293	0.980	0.020

8394196	NTM	neurotrimin	0.297	0.978	0.022
61557316	ST3GAL1	ST3 beta-galactoside alpha-2,3-sialyltransferase 1	0.303	0.974	0.026
56605644	TFB2M	transcription factor B2, mitochondrial	0.303	0.961	0.039
285002227	SH3BP1	SH3 domain binding protein 1	0.304	0.991	0.009
28212232	GNL3	G protein nucleolar 3	0.305	0.974	0.026
34882672	ETAA1	ETAA1 activator of ATR kinase	0.322	0.951	0.049
880805457	GPR161	G protein-coupled receptor 161	0.324	1.000	0.000
157824026	TCEANC	transcription elongation factor A N-terminal and central domain containing	0.341	0.993	0.007
157787079	NVL	nuclear VCP like	0.347	0.986	0.014
291167790	Fam13a	family with sequence similarity 13, member A	0.347	0.966	0.034
157786710	CEP112	centrosomal protein 112	0.347	1.000	0.000
564368492	CARF	calcium responsive transcription factor	0.351	0.992	0.008
201066365	PAPSS2	3'-phosphoadenosine 5'-phosphosulfate synthase 2	0.359	0.965	0.035
157822721	NUP42	nucleoporin 42	0.361	0.969	0.031
755542850	RGS6	regulator of G protein signaling 6	0.361	0.983	0.017
58865996	TRIM13	tripartite motif containing 13	0.361	0.960	0.040
209529638	RSBN1L	round spermatid basic protein 1 like	0.369	0.971	0.029
157820949	SAMD5	sterile alpha motif domain containing 5	0.372	0.966	0.034
40538878	NEXN	nexilin F-actin binding protein	0.379	0.976	0.024
148747270	PTGS2	prostaglandin-endoperoxide synthase 2	0.408	0.978	0.022
16758574	CNTN5	contactin 5	0.414	0.956	0.044
564362348	Fam76b	family with sequence similarity 76, member B	0.429	0.995	0.005
157819193	DOK5	docking protein 5	0.430	0.977	0.023
129772	PENK	proenkephalin	0.433	0.969	0.031
74196108	NECTIN4	nectin cell adhesion molecule 4	0.442	0.960	0.040
157817047	NEDD1	NEDD1 gamma-tubulin ring complex targeting factor	0.451	0.955	0.045
149066425	A930017M01Rik	Smg-5 homolog, nonsense mediated mRNA decay factor pseudogene	0.454	0.962	0.038
564382848	Hnrnpdl	heterogeneous nuclear ribonucleoprotein D-like	0.464	0.980	0.020
7949105	PBX3	PBX homeobox 3	0.468	0.993	0.007
17530969	SLC8A3	solute carrier family 8 member A3	0.483	0.959	0.041
149053039	KCNAB3	potassium voltage-gated channel subfamily A regulatory beta subunit 3	0.488	0.967	0.033

11560065	GPR85	G protein-coupled receptor 85	0.502	0.992	0.008
149017375	ARAP3	ArfGAP with RhoGAP domain, ankyrin repeat and PH domain 3	0.513	0.954	0.046
195539325	MTBP	MDM2 binding protein	0.523	0.985	0.015
62078463	LPP	LIM domain containing preferred translocation partner in lipoma	0.528	0.953	0.047
21955136	MXD3	MAX dimerization protein 3	0.535	0.994	0.006
114145465	LOC689840	LRRGT00142	0.604	0.961	0.039
672068300	TOP3A	DNA topoisomerase III alpha	0.629	0.957	0.043
564389552	LOC100910854	zinc finger MYND domain-containing protein 19-like	0.635	0.965	0.035
39104628	SORBS1	sorbin and SH3 domain containing 1	0.639	0.993	0.007
568907669	NYAP2	neuronal tyrosine-phosphorylated phosphoinositide-3-kinase adaptor 2	0.645	0.963	0.037
281604200	COL9A1	collagen type IX alpha 1 chain	0.648	0.992	0.008
149062619	CBWD1	COBW domain containing 1	0.717	0.983	0.017
6681177	TWIST2	twist family bHLH transcription factor 2	0.737	0.967	0.033
58865510	GIMAP6	GTPase, IMAP family member 6	0.783	0.995	0.005
564316247	CEP170	centrosomal protein 170	0.793	0.991	0.009
300795362	PTCHD1	patched domain containing 1	0.820	0.960	0.040
158517955	PKHD1L1	PKHD1 like 1	0.848	0.997	0.003
293353154	TBC1D1*	TBC1 domain family member 1	0.949	0.998	0.002
564395190	LOC100909409 (includes others)	RGD1562660	0.962	0.989	0.011
961454277	Dmd	dystrophin	1.007	0.999	0.001
149042010	Dapk2	death-associated protein kinase 2	1.010	0.989	0.011
281306708	VSX2	visual system homeobox 2	1.108	0.979	0.021
392339412	PLA2G4E	phospholipase A2 group IVE	1.128	0.970	0.030
12083683	SRD5A2	steroid 5 alpha-reductase 2	1.128	0.992	0.008
37693512	TLR9	toll like receptor 9	1.160	0.957	0.043
157818969	SEC14L4	SEC14 like lipid binding 4	1.263	0.987	0.013
149020413	Zfp599	zinc finger protein 599	1.322	0.990	0.010
564300485	LOC102551095	uncharacterized LOC102551095	1.355	0.951	0.049
577019502	OVOL3	ovo like zinc finger 3	1.363	0.981	0.019
31745152	GPR151	G protein-coupled receptor 151	1.379	0.967	0.033
126517485	TBC1D10C	TBC1 domain family member 10C	1.392	0.979	0.021
57012346	HLA-DQA1	major histocompatibility complex, class II, DQ alpha 1	1.415	0.967	0.033
24899633	SLC12A8	solute carrier family 12 member 8	1.519	0.978	0.022
94400879	HRH2	histamine receptor H2	1.585	0.983	0.017
19424330	SLC25A21	solute carrier family 25 member 21	1.683	0.957	0.043
11464989	CD86	CD86 molecule	1.700	0.989	0.011
157817658	VIL1	villin 1	1.700	0.993	0.007

672035395	DMWD	DM1 locus, WD repeat containing	1.706	0.969	0.031
300794357	AOAH	acyloxyacyl hydrolase	1.755	1.000	0.000
149024390	PLA2G2D	phospholipase A2 group IID	1.914	0.959	0.041
392354688	LVRN	laeverin	2.121	0.978	0.022
672067367	BC049762	cDNA sequence BC049762	2.126	0.953	0.047
149061720	Tnnt3	troponin T3, fast skeletal type	2.182	0.996	0.004
148696062	LRRC57	leucine rich repeat containing 57	2.603	0.992	0.008
392334596	RSPH3	radial spoke head 3	2.755	0.968	0.032
148705984	Spink2	serine peptidase inhibitor, Kazal type 2	6.000	0.951	0.049

Supplementary Table S13. The list of genes that are differentially expressed in the offspring hippocampus in response to prenatal BPA exposure that exhibited the changes in the expression levels correlated with the neurite length of primary hippocampal cells at DIV7. The transcriptome profiling data of DEGs in male and female rat offspring prenatally exposed to BPA (n = 6, male pups n = 3 and female pups n = 3, from independent litters) or the vehicle control (n = 6, male pups n = 3 and female pups n = 3, from independent litters) were obtained and used for the PTM analyses to identify DEGs that exhibited the changes in the expression levels correlated with the neurite length of primary hippocampal cells at DIV7.

ID	Symbol	Entrez Gene Name	log2(FC)	R values	P-values
293347435	PTPRD	protein tyrosine phosphatase receptor type D	-7.731	-0.976	0.024
564375502	Mxra7	matrix-remodelling associated 7	-7.209	-0.962	0.038
564310188	IGDCC4	immunoglobulin superfamily DCC subclass member 4	-6.728	-0.952	0.048
564314389	DZIP3	DAZ interacting zinc finger protein 3	-6.700	-0.985	0.015
149020633	TAF1D	TATA-box binding protein associated factor, RNA polymerase I subunit D	-6.476	-0.985	0.015
157818475	SMIM22	small integral membrane protein 22	-5.615	-0.982	0.018
567315993	LOC102550396	LRRGT00188	-5.600	-0.965	0.035
564312230	LOC100912948	multidrug resistance-associated protein 1-like	-5.285	-0.962	0.038
197384778	Snorc	secondary ossification center associated regulator of chondrocyte maturation	-5.044	-0.981	0.019
564323305	LOC681300	similar to CXXC finger 5	-4.672	-0.985	0.015
209447125	Ctf2	cardiotrophin 2	-4.392	-0.962	0.038
201860265	NRN1L	neuritin 1 like	-4.358	-0.969	0.031
300798104	IFNLR1	interferon lambda receptor 1	-4.248	-0.981	0.019
51591901	MPIG6B	megakaryocyte and platelet inhibitory receptor G6b	-4.170	-0.962	0.038
564298047	GDPD5	glycerophosphodiester phosphodiesterase domain containing 5	-4.163	-0.969	0.031
300798035	NRG4	neuregulin 4	-4.044	-0.971	0.029
61556838	Raet1d/Raet1e	retinoic acid early transcript 1E	-4.044	-0.971	0.029
114145748	LOC680227	LRRGT00193	-3.907	-0.953	0.047
6978515	APOA1	apolipoprotein A1	-3.807	-0.962	0.038
117647210	CTRC	chymotrypsin C	-3.807	-0.962	0.038
48040447	SUCNR1	succinate receptor 1	-3.700	-0.962	0.038
8392926	ASGR2	asialoglycoprotein receptor 2	-3.700	-0.960	0.040
194473646	UPK3A	uroplakin 3A	-3.585	-0.962	0.038

164518908	RAB25	RAB25, member RAS oncogene family	-3.459	-0.962	0.038
285026465	HS3ST3A1	heparan sulfate-glucosamine 3-sulfotransferase 3A1	-3.322	-0.962	0.038
148710035	PITX3	paired like homeodomain 3	-3.322	-0.962	0.038
9506451	CA5A	carbonic anhydrase 5A	-3.248	-0.976	0.024
56788780	KRT19	keratin 19	-3.248	-0.951	0.049
56090299	ODF4	outer dense fiber of sperm tails 4	-3.170	-0.981	0.019
157822159	CCDC42	coiled-coil domain containing 42	-3.170	-0.962	0.038
123173794	GSG1	germ cell associated 1	-3.170	-0.962	0.038
77917534	CBLC	Cbl proto-oncogene C	-2.807	-0.962	0.038
157822121	LRMDA	leucine rich melanocyte differentiation associated	-2.747	-0.999	0.001
51948496	SLC22A18	solute carrier family 22 member 18	-2.687	-0.982	0.018
672032217	REPS2	RALBP1 associated Eps domain containing 2	-2.683	-0.998	0.002
298566276	Ces1a	carboxylesterase 1A	-2.585	-0.962	0.038
57222328	PFN4	profilin family member 4	-2.585	-0.962	0.038
157786772	KREMEN2	kringle containing transmembrane protein 2	-2.585	-0.962	0.038
306482632	BPIFB4	BPI fold containing family B member 4	-2.585	-0.962	0.038
57222300	Klra2	killer cell lectin-like receptor, subfamily A, member 2	-2.585	-0.962	0.038
157822463	Nkx6-3	NK6 homeobox 3	-2.585	-0.962	0.038
564391680	MAP3K8	mitogen-activated protein kinase kinase kinase 8	-2.426	-0.970	0.030
62945342	LAX1	lymphocyte transmembrane adaptor 1	-2.322	-0.962	0.038
187469451	CLEC7A	C-type lectin domain containing 7A	-2.322	-0.962	0.038
157820151	ERAS	ES cell expressed Ras	-2.248	-0.987	0.013
564320724	Fbxo38	F-box protein 38	-2.220	-0.977	0.023
66730349	SPIB	Spi-B transcription factor	-2.170	-0.985	0.015
25742828	SCN7A	sodium voltage-gated channel alpha subunit 7	-2.129	-0.950	0.050
300797305	TMEM45A	transmembrane protein 45A	-2.093	-0.992	0.008
148672128	SMAGP	small cell adhesion glycoprotein	-2.077	-0.981	0.019
55741882	ZPBP2	zona pellucida binding protein 2	-2.022	-0.968	0.032
157822587	PDE6B	phosphodiesterase 6B	-2.000	-0.962	0.038
157818091	TMEM182	transmembrane protein 182	-2.000	-0.962	0.038
296483047	SIX1	SIX homeobox 1	-2.000	-0.962	0.038
117647206	DDX4	DEAD-box helicase 4	-2.000	-0.962	0.038
19424304	CHRNB3	cholinergic receptor nicotinic beta 3 subunit	-2.000	-0.962	0.038
47577151	Olfr1441	olfactory receptor 1441	-1.972	-0.962	0.038

56676350	PRSS35	serine protease 35	-1.962	-0.995	0.005
157820217	Gsta4	glutathione S-transferase, alpha 4	-1.930	-0.962	0.038
117647214	EDN3	endothelin 3	-1.913	-0.982	0.018
81295367	Abcg3	ATP binding cassette subfamily G member 3	-1.898	-0.990	0.010
672029702	TUT7	terminal uridylyl transferase 7	-1.854	-0.975	0.025
13592031	PTGER2	prostaglandin E receptor 2	-1.845	-0.999	0.001
297374767	TPSAB1/TPSB2	tryptase alpha/beta 1	-1.834	-0.969	0.031
149064260	PRDM6	PR/SET domain 6	-1.816	-0.954	0.046
9910234	IFIT1B	interferon induced protein with tetratricopeptide repeats 1B	-1.678	-0.962	0.038
153792385	Vom2r34	vomeronasal 2 receptor, 34	-1.648	-0.955	0.045
31542125	ALOX15	arachidonate 15-lipoxygenase	-1.611	-0.961	0.039
58331159	GSTA3	glutathione S-transferase alpha 3	-1.603	-0.999	0.001
76443687	SLC4A1	solute carrier family 4 member 1 (Diego blood group)	-1.599	-0.963	0.037
157821527	RHOD	ras homolog family member D	-1.597	-0.998	0.002
157819393	NNMT	nicotinamide N-methyltransferase	-1.585	-0.998	0.002
209870037	INSRR	insulin receptor related receptor	-1.585	-0.977	0.023
157818603	CLCA2	chloride channel accessory 2	-1.585	-0.962	0.038
685156911	NLRP4	NLR family pyrin domain containing 4	-1.585	-0.962	0.038
149034165	GALNT15	polypeptide N-acetylgalactosaminyltransferase 15	-1.561	-0.960	0.040
13929126	GALNT5	polypeptide N-acetylgalactosaminyltransferase 5	-1.478	-0.972	0.028
71043750	SYNGR4	synaptogyrin 4	-1.464	-0.978	0.022
81295349	SLC52A3	solute carrier family 52 member 3	-1.453	-0.985	0.015
283806636	ZNF831	zinc finger protein 831	-1.433	-0.984	0.016
31377521	S1PR5	sphingosine-1-phosphate receptor 5	-1.426	-0.979	0.021
77917586	GRAP2	GRB2 related adaptor protein 2	-1.412	-0.977	0.023
157822105	SLC49A3	solute carrier family 49 member 3	-1.389	-1.000	0.000
672025117	MBTD1	mbt domain containing 1	-1.344	-0.989	0.011
6978717	CTRB2	chymotrypsinogen B2	-1.342	-0.983	0.017
29789044	SNAI2	snail family transcriptional repressor 2	-1.328	-0.984	0.016
307746876	Pzp	PZP, alpha-2-macroglobulin like	-1.322	-0.962	0.038
157823345	LRR1	leucine rich repeat protein 1	-1.322	-0.960	0.040
148694035	SENP8	SUMO peptidase family member, NEDD8 specific	-1.276	-0.970	0.030
62656582	KIAA0100	KIAA0100	-1.266	-0.995	0.005
187281975	DENND1C	DENN domain containing 1C	-1.216	-0.955	0.045
16758338	FTCD	formimidoyltransferase cyclodeaminase	-1.208	-0.966	0.034
56119141	BTK	Bruton tyrosine kinase	-1.205	-0.972	0.028

564300462	DCHS2	dachsous cadherin-related 2	-1.190	-0.967	0.033
392334475	Myb	MYB proto-oncogene, transcription factor	-1.175	-0.992	0.008
71795615	UPP1	uridine phosphorylase 1	-1.167	-0.955	0.045
281332082	THBS2	thrombospondin 2	-1.164	-0.988	0.012
157823801	SLC50A1	solute carrier family 50 member 1	-1.136	-0.989	0.011
60223053	SEPTIN1	septin 1	-1.133	-0.998	0.002
157819247	CPA4	carboxypeptidase A4	-1.087	-0.962	0.038
157818961	UBA7	ubiquitin like modifier activating enzyme 7	-1.078	-0.958	0.042
197386139	SSC5D	scavenger receptor cysteine rich family member with 5 domains	-1.060	-0.964	0.036
157821423	TBX6	T-box transcription factor 6	-1.017	-0.957	0.043
300797728	MGST3	microsomal glutathione S-transferase 3	-1.011	-0.981	0.019
25453414	ASS1	argininosuccinate synthase 1	-1.000	-0.982	0.018
156231008	PRND	prion like protein doppel	-1.000	-0.962	0.038
765826426	Acot6	acyl-CoA thioesterase 6	-1.000	-0.974	0.026
59676595	FAM20A	FAM20A golgi associated secretory pathway pseudokinase	-1.000	-0.970	0.030
569009290	TENM1	teneurin transmembrane protein 1	-0.985	-0.991	0.009
407228396	THEMIS2	thymocyte selection associated family member 2	-0.980	-0.970	0.030
157817157	FAM166C	family with sequence similarity 166 member C	-0.979	-0.970	0.030
215276950	PKP2	plakophilin 2	-0.973	-0.992	0.008
672033256	LOC100912904	disks large homolog 5-like	-0.960	-0.959	0.041
157818989	LRRC71	leucine rich repeat containing 71	-0.952	-0.994	0.006
157818275	KCNG4	potassium voltage-gated channel modifier subfamily G member 4	-0.952	-0.971	0.029
198442873	CDC14A	cell division cycle 14A	-0.936	-0.996	0.004
62945330	SLC8B1	solute carrier family 8 member B1	-0.929	-0.997	0.003
50657416	C1RL	complement C1r subcomponent like	-0.918	-0.962	0.038
157819487	TACO1	translational activator of cytochrome c oxidase I	-0.917	-0.975	0.025
47059114	LTB	lymphotoxin beta	-0.916	-0.981	0.019
564325648	Zfp54	zinc finger protein 54	-0.907	-0.986	0.014
564318923	WDR17	WD repeat domain 17	-0.904	-0.997	0.003
209870105	GPR37L1	G protein-coupled receptor 37 like 1	-0.870	-0.987	0.013
57114338	SCN4B	sodium voltage-gated channel beta subunit 4	-0.859	-0.998	0.002
149031998	ACVRL1	activin A receptor like type 1	-0.852	-0.997	0.003
157822457	SYNC	syncoilin, intermediate filament protein	-0.833	-0.957	0.043

564373460	SLFN13	schlafen family member 13	-0.831	-0.979	0.021
56605720	GADD45B	growth arrest and DNA damage inducible beta	-0.828	-0.998	0.002
58865784	GPR157	G protein-coupled receptor 157	-0.812	-0.985	0.015
157817065	KCNK16	potassium two pore domain channel subfamily K member 16	-0.807	-0.960	0.040
259089426	AGER	advanced glycosylation end-product specific receptor	-0.804	-0.999	0.001
18426812	ADA	adenosine deaminase	-0.787	-0.969	0.031
307548437	NYAP2	neuronal tyrosine-phosphorylated phosphoinositide-3-kinase adaptor 2	-0.780	-0.997	0.003
942523340	CAPRIN2	caprin family member 2	-0.774	-0.999	0.001
157819205	EFHC2	EF-hand domain containing 2	-0.772	-0.979	0.021
61097937	VEGFB	vascular endothelial growth factor B	-0.766	-0.957	0.043
56090397	CYB5D2	cytochrome b5 domain containing 2	-0.764	-0.957	0.043
27465577	Cyp4f16/Cyp4f37	cytochrome P450, family 4, subfamily f, polypeptide 16	-0.758	-0.989	0.011
13591914	ANPEP	alanyl aminopeptidase, membrane	-0.757	-0.981	0.019
672069802	C1QTNF1	C1q and TNF related 1	-0.749	-0.998	0.002
157786850	TUBD1	tubulin delta 1	-0.740	-0.970	0.030
56119147	ARRDC3	arrestin domain containing 3	-0.732	-0.969	0.031
208973286	RBM46	RNA binding motif protein 46	-0.724	-0.952	0.048
16758232	PLCB2	phospholipase C beta 2	-0.721	-0.964	0.036
19173756	ERG	ETS transcription factor ERG	-0.718	-0.972	0.028
149047075	Spaca6	sperm acrosome associated 6	-0.715	-0.987	0.013
564378828	TFR2	transferrin receptor 2	-0.707	-0.952	0.048
564317714	Ktn1	kinectin 1	-0.687	-0.972	0.028
300794452	PTPRH	protein tyrosine phosphatase receptor type H	-0.687	-0.954	0.046
74142284	DSE	dermatan sulfate epimerase	-0.667	-0.970	0.030
157818909	Zim1	zinc finger, imprinted 1	-0.665	-0.966	0.034
672057459	DGKA	diacylglycerol kinase alpha	-0.664	-0.959	0.041
70912395	CFAP20DC	CFAP20 domain containing	-0.659	-0.952	0.048
201861690	TPK1	thiamin pyrophosphokinase 1	-0.657	-0.956	0.044
157824216	RRAS	RAS related	-0.655	-0.981	0.019
9845261	LGALS1	galectin 1	-0.655	-0.973	0.027
164565435	SYNJ2	synaptojanin 2	-0.654	-0.950	0.050
157823283	Coch	cochlin	-0.653	-0.988	0.012
77020281	CD55	CD55 molecule (Cromer blood group)	-0.648	-0.955	0.045
68534736	ERAP1	endoplasmic reticulum aminopeptidase 1	-0.646	-0.957	0.043
13786136	PDGFC	platelet derived growth factor C	-0.642	-0.982	0.018
293340128	MIEF2	mitochondrial elongation factor 2	-0.642	-0.956	0.044
149061352	ADAM12	ADAM metallopeptidase domain 12	-0.638	-0.976	0.024

34734058	HCK	HCK proto-oncogene, Src family tyrosine kinase	-0.631	-0.991	0.009
397529557	C8orf58	chromosome 8 open reading frame 58	-0.629	-0.975	0.025
157824208	NTNG1	netrin G1	-0.628	-0.983	0.017
16758622	IFT172	intraflagellar transport 172	-0.627	-0.981	0.019
564395215	LOC100909409 (includes others)	RGD1562660	-0.626	-0.973	0.027
402478640	HTRA3	HtrA serine peptidase 3	-0.608	-0.997	0.003
157819065	ADAMTS15	ADAM metallopeptidase with thrombospondin type 1 motif 15	-0.604	-0.973	0.027
157818491	DUS2	dihydrouridine synthase 2	-0.602	-0.970	0.030
27465529	SLC9A4	solute carrier family 9 member A4	-0.601	-0.983	0.017
56605940	RXFP3	relaxin family peptide receptor 3	-0.599	-0.985	0.015
293347888	SRBD1	S1 RNA binding domain 1	-0.598	-0.986	0.014
6978737	CYP1B1	cytochrome P450 family 1 subfamily B member 1	-0.593	-0.957	0.043
567316103	Ac1576	uncharacterized LOC102552783	-0.587	-0.991	0.009
197927123	LYRM7	LYR motif containing 7	-0.587	-0.996	0.004
300793858	PARP14	poly(ADP-ribose) polymerase family member 14	-0.585	-0.991	0.009
564394999	CLGN	calmegin	-0.582	-0.986	0.014
564300485	LOC102551095	uncharacterized LOC102551095	-0.576	-0.974	0.026
16758560	WIF1	WNT inhibitory factor 1	-0.573	-0.993	0.007
564366772	MGC116197 (includes others)	similar to RIKEN cDNA 1700001E04	-0.569	-0.979	0.021
55741859	XRCC4	X-ray repair cross complementing 4	-0.569	-0.988	0.012
157816941	PLXDC1	plexin domain containing 1	-0.567	-0.989	0.011
62078799	QRSL1	glutaminyl-tRNA amidotransferase subunit QRSL1	-0.566	-0.998	0.002
57012436	Krt10	keratin 10	-0.562	-0.977	0.023
75832150	GALNT3	polypeptide N-acetylgalactosaminyltransferase 3	-0.557	-0.998	0.002
213385268	Gm10778	predicted gene 10778	-0.554	-0.970	0.030
300798350	LRRK1	leucine rich repeat kinase 1	-0.554	-0.957	0.043
9507045	RGS5	regulator of G protein signaling 5	-0.538	-0.980	0.020
148747464	SCD	stearoyl-CoA desaturase	-0.524	-0.977	0.023
19424232	CSF2RB	colony stimulating factor 2 receptor subunit beta	-0.524	-0.979	0.021
6981180	MAOB	monoamine oxidase B	-0.518	-0.986	0.014
148692356	ARHGEF1	Rho guanine nucleotide exchange factor 1	-0.517	-0.988	0.012
149037033	PRDM5	PR/SET domain 5	-0.516	-0.987	0.013
149047863	LOC690190	hypothetical protein LOC690190	-0.514	-0.967	0.033
147900684	TLR7	toll like receptor 7	-0.511	-0.976	0.024

62078773	CCDC81	coiled-coil domain containing 81	-0.511	-0.998	0.002
654824082	Fbxl21	F-box and leucine-rich repeat protein 21	-0.500	-0.984	0.016
50233928	TMEM159	transmembrane protein 159	-0.497	-0.966	0.034
157822759	PARP2	poly(ADP-ribose) polymerase 2	-0.493	-0.965	0.035
399220341	SLC2A13	solute carrier family 2 member 13	-0.492	-0.976	0.024
157819347	CDC6	cell division cycle 6	-0.492	-0.987	0.013
12738847	MERTK	MER proto-oncogene, tyrosine kinase	-0.488	-0.953	0.047
395759219	AQP4	aquaporin 4	-0.482	-0.997	0.003
189011606	NCEH1	neutral cholesterol ester hydrolase 1	-0.476	-0.990	0.010
41056215	XRCC5	X-ray repair cross complementing 5	-0.466	-0.996	0.004
148356229	CCND1	cyclin D1	-0.464	-0.953	0.047
56090632	DMAC2L	distal membrane arm assembly complex 2 like	-0.463	-0.964	0.036
148669431	DNAJC27	DnaJ heat shock protein family (Hsp40) member C27	-0.459	-0.953	0.047
68163403	SLC46A3	solute carrier family 46 member 3	-0.458	-0.976	0.024
219275548	DUSP19	dual specificity phosphatase 19	-0.458	-0.955	0.045
56090411	POLE3	DNA polymerase epsilon 3, accessory subunit	-0.456	-0.991	0.009
149067372	MTERF2	mitochondrial transcription termination factor 2	-0.455	-0.955	0.045
157817743	CDH5	cadherin 5	-0.454	-0.990	0.010
9437326	SLC4A4	solute carrier family 4 member 4	-0.453	-0.970	0.030
13027400	GUCY1A2	guanylate cyclase 1 soluble subunit alpha 2	-0.453	-0.957	0.043
158138494	PTPRC	protein tyrosine phosphatase receptor type C	-0.451	-0.965	0.035
33414515	PXK	PX domain containing serine/threonine kinase like	-0.450	-0.979	0.021
58865380	STAT2	signal transducer and activator of transcription 2	-0.450	-0.969	0.031
392331978	CDR2L	cerebellar degeneration related protein 2 like	-0.442	-0.978	0.022
149022245	SCRN3	secernin 3	-0.437	-0.962	0.038
68534547	NUDT18	nudix hydrolase 18	-0.437	-0.985	0.015
148686921	SLC24A4	solute carrier family 24 member 4	-0.435	-0.969	0.031
404312655	SDR42E1	short chain dehydrogenase/reductase family 42E, member 1	-0.435	-0.956	0.044
61889119	TNFSF12	TNF superfamily member 12	-0.432	-0.996	0.004
157822677	LGI3	leucine rich repeat LGI family member 3	-0.431	-0.951	0.049
157823259	TMEM229A	transmembrane protein 229A	-0.424	-0.995	0.005
300796997	ARHGAP28	Rho GTPase activating protein 28	-0.423	-0.976	0.024

210032365	HSP90B1	heat shock protein 90 beta family member 1	-0.421	-0.985	0.015
56090564	GALM	galactose mutarotase	-0.420	-0.984	0.016
62945312	CXCL16	C-X-C motif chemokine ligand 16	-0.414	-0.984	0.016
194473652	TTC38	tetratricopeptide repeat domain 38	-0.413	-0.952	0.048
9506405	ARPC1B	actin related protein 2/3 complex subunit 1B	-0.410	-0.974	0.026
13591949	GATM	glycine amidinotransferase	-0.409	-0.992	0.008
13162347	FDXR	ferredoxin reductase	-0.405	-0.994	0.006
195973006	EGFLAM	EGF like, fibronectin type III and laminin G domains	-0.403	-0.961	0.039
56605714	NDUFAF7	NADH:ubiquinone oxidoreductase complex assembly factor 7	-0.399	-0.961	0.039
148666792	ARHGAP25	Rho GTPase activating protein 25	-0.396	-0.977	0.023
78187977	TCF19	transcription factor 19	-0.393	-0.962	0.038
157820327	THSD1	thrombospondin type 1 domain containing 1	-0.391	-0.975	0.025
187937124	TMEM126B	transmembrane protein 126B	-0.389	-0.978	0.022
166999225	GRM1	glutamate metabotropic receptor 1	-0.385	-0.961	0.039
149034469	GNG7	G protein subunit gamma 7	-0.381	-0.996	0.004
149036529	DGUOK	deoxyguanosine kinase	-0.381	-0.974	0.026
929981595	NPHP1	nephrocystin 1	-0.372	-0.996	0.004
157824002	ATG10	autophagy related 10	-0.372	-0.962	0.038
564299653	FAM169A	family with sequence similarity 169 member A	-0.372	-1.000	0.000
198386343	TRPS1	transcriptional repressor GATA binding 1	-0.370	-0.998	0.002
8393861	HPCAL4	hippocalcin like 4	-0.370	-0.986	0.014
158303308	PCCA	propionyl-CoA carboxylase subunit alpha	-0.369	-0.965	0.035
564343748	CDK5RAP1	CDK5 regulatory subunit associated protein 1	-0.368	-0.992	0.008
564357619	ITGB8	integrin subunit beta 8	-0.366	-0.991	0.009
16758712	PDIA4	protein disulfide isomerase family A member 4	-0.362	-0.991	0.009
55741549	MRPL13	mitochondrial ribosomal protein L13	-0.360	-0.997	0.003
149066868	MDM1	Mdm1 nuclear protein	-0.357	-0.999	0.001
157822187	WWOX	WW domain containing oxidoreductase	-0.355	-0.963	0.037
11693172	CALR	calreticulin	-0.354	-0.980	0.020
564331077	HIRIP3	HIRA interacting protein 3	-0.352	-0.987	0.013
56605656	DONSON	DNA replication fork stabilization factor DONSON	-0.350	-0.969	0.031
451172073	CHRM3	cholinergic receptor muscarinic 3	-0.349	-0.988	0.012

62078847	TSEN2	tRNA splicing endonuclease subunit 2	-0.343	-0.975	0.025
77157795	MAL2	mal, T cell differentiation protein 2 (gene/pseudogene)	-0.342	-0.998	0.002
11693162	INSIG1	insulin induced gene 1	-0.339	-0.978	0.022
56605710	LTBR	lymphotoxin beta receptor	-0.338	-0.951	0.049
300795679	CD84	CD84 molecule	-0.338	-0.976	0.024
13786174	TIMELESS	timeless circadian regulator	-0.332	-0.974	0.026
300794275	MFSD10	major facilitator superfamily domain containing 10	-0.330	-0.973	0.027
564309649	CCDC159	coiled-coil domain containing 159	-0.329	-0.976	0.024
281604125	Fam50a/LOC100910130	family with sequence similarity 50, member A	-0.327	-0.972	0.028
149045696	Ccl27a	chemokine (C-C motif) ligand 27A	-0.325	-0.965	0.035
62078809	TNFAIP8L2	TNF alpha induced protein 8 like 2	-0.324	-0.958	0.042
51036680	SLC29A3	solute carrier family 29 member 3	-0.322	-0.964	0.036
451172111	HINT3	histidine triad nucleotide binding protein 3	-0.321	-0.993	0.007
40018538	ADI1	acireductone dioxygenase 1	-0.316	-0.951	0.049
300797242	SPG11	SPG11 vesicle trafficking associated, spatacsin	-0.316	-0.953	0.047
149041432	THY1	Thy-1 cell surface antigen	-0.313	-0.969	0.031
157786608	MRPL55	mitochondrial ribosomal protein L55	-0.313	-0.959	0.041
564336403	EXOSC8	exosome component 8	-0.309	-0.988	0.012
62078551	GNB4	G protein subunit beta 4	-0.309	-0.951	0.049
149063353	IFT81	intraflagellar transport 81	-0.309	-0.998	0.002
167860097	FN3KRP	fructosamine 3 kinase related protein	-0.308	-0.964	0.036
12621108	NR1I3	nuclear receptor subfamily 1 group I member 3	-0.308	-0.951	0.049
61556910	SNX10	sorting nexin 10	-0.306	-0.988	0.012
31982028	RSU1	Ras suppressor protein 1	-0.304	-0.989	0.011
300796069	THADA	THADA armadillo repeat containing	-0.303	-0.984	0.016
56090361	EPDR1	ependymin related 1	-0.303	-0.973	0.027
198041989	PARVB	parvin beta	-0.302	-0.953	0.047
157820737	NUSAP1	nucleolar and spindle associated protein 1	-0.298	-0.958	0.042
38259192	TOP2A	DNA topoisomerase II alpha	-0.297	-0.993	0.007
162287198	HSD17B4	hydroxysteroid 17-beta dehydrogenase 4	-0.297	-0.957	0.043
6978888	GFRA1	GDNF family receptor alpha 1	-0.294	-0.968	0.032
672035779	Proser3	proline and serine rich 3	-0.293	-0.967	0.033
564358911	CHPT1	choline phosphotransferase 1	-0.292	-0.971	0.029
157817979	Egfem1	EGF-like and EMI domain containing 1	-0.291	-0.980	0.020
402765953	0610009B22Rik	RIKEN cDNA 0610009B22 gene	-0.287	-0.951	0.049

149034870	RNF6	ring finger protein 6	-0.283	-0.952	0.048
21489989	KCNH8	potassium voltage-gated channel subfamily H member 8	-0.282	-0.951	0.049
157823996	ELK3	ETS transcription factor ELK3	-0.282	-0.965	0.035
564317068	CCDC149	coiled-coil domain containing 149	-0.281	-0.960	0.040
197313643	GLTP	glycolipid transfer protein	-0.280	-0.979	0.021
142349612	GLUL	glutamate-ammonia ligase	-0.279	-0.983	0.017
564364473	RNF111	ring finger protein 111	-0.276	-0.960	0.040
197333840	CAMKMT	calmodulin-lysine N-methyltransferase	-0.270	-0.968	0.032
149022319	AGPS	alkylglycerone phosphate synthase	-0.270	-0.967	0.033
149068830	SLCO2B1	solute carrier organic anion transporter family member 2B1	-0.270	-0.972	0.028
148701892	EBF1	EBF transcription factor 1	-0.270	-0.964	0.036
57164113	NSDHL	NAD(P) dependent steroid dehydrogenase-like	-0.269	-0.976	0.024
576796148	MAP7D2	MAP7 domain containing 2	-0.269	-0.988	0.012
74354506	ACBD5	acyl-CoA binding domain containing 5	-0.269	-0.982	0.018
197313676	AIG1	androgen induced 1	-0.268	-0.987	0.013
38181552	SCG2	secretogranin II	-0.267	-0.960	0.040
157819753	RCN1	reticulocalbin 1	-0.266	-0.969	0.031
564382316	HSD11B1	hydroxysteroid 11-beta dehydrogenase 1	-0.265	-0.973	0.027
564382292	ANGEL2	angel homolog 2	-0.258	-0.977	0.023
17865325	GLRB	glycine receptor beta	-0.256	-0.998	0.002
149046389	ARID5A	AT-rich interaction domain 5A	-0.249	-0.961	0.039
157819311	LRGUK	leucine rich repeats and guanylate kinase domain containing	-0.248	-0.965	0.035
157819077	TRIM37	tripartite motif containing 37	-0.246	-0.988	0.012
15805026	ZFAND6	zinc finger AN1-type containing 6	-0.245	-0.994	0.006
6978631	CD4	CD4 molecule	-0.245	-0.990	0.010
56090433	GLT8D1	glycosyltransferase 8 domain containing 1	-0.243	-0.953	0.047
187937143	C2orf42	chromosome 2 open reading frame 42	-0.241	-0.974	0.026
57527332	PSPH	phosphoserine phosphatase	-0.241	-0.958	0.042
58865958	RDH11	retinol dehydrogenase 11	-0.240	-0.985	0.015
57192	P3H4	prolyl 3-hydroxylase family member 4 (inactive)	-0.240	-0.963	0.037
672047003	CDAN1	codanin 1	-0.236	-0.960	0.040
157817480	RWDD2A	RWD domain containing 2A	-0.235	-0.967	0.033
77404265	JAM2	junctional adhesion molecule 2	-0.233	-0.982	0.018
584277046	SLC1A3	solute carrier family 1 member 3	-0.232	-0.988	0.012
293346766	TCAF1	TRPM8 channel associated factor 1	-0.232	-0.983	0.017

9457244	RBBP9	RB binding protein 9, serine hydrolase	-0.231	-0.978	0.022
157817839	SEMA5A	semaphorin 5A	-0.230	-0.974	0.026
564397761	GCC2	GRIP and coiled-coil domain containing 2	-0.229	-0.998	0.002
58865718	HERC4	HECT and RLD domain containing E3 ubiquitin protein ligase 4	-0.228	-0.980	0.020
149062310	BSCL2	BSCL2 lipid droplet biogenesis associated, seipin	-0.228	-0.955	0.045
672050038	NDNF	neuron derived neurotrophic factor	-0.226	-0.982	0.018
205277356	TVP23B	trans-golgi network vesicle protein 23 homolog B	-0.226	-0.962	0.038
157817710	FER	FER tyrosine kinase	-0.225	-0.981	0.019
293345175	DHX29	DExH-box helicase 29	-0.225	-0.999	0.001
564383995	EVC	EvC ciliary complex subunit 1	-0.225	-0.956	0.044
25742576	NXF1	nuclear RNA export factor 1	-0.224	-0.974	0.026
78369663	SLC38A9	solute carrier family 38 member 9	-0.222	-0.968	0.032
148689145	CPNE4	copine 4	-0.222	-0.994	0.006
148695758	CAPRIN1	cell cycle associated protein 1	-0.221	-0.988	0.012
157822873	FBH1	F-box DNA helicase 1	-0.219	-0.951	0.049
9506469	CD47	CD47 molecule	-0.218	-0.954	0.046
564298436	WDR11	WD repeat domain 11	-0.216	-0.967	0.033
148687519	CALN1	calneuron 1	-0.214	-0.987	0.013
8393643	KCNAB1	potassium voltage-gated channel subfamily A member regulatory beta subunit 1	-0.214	-0.993	0.007
57526927	LARS1	leucyl-tRNA synthetase 1	-0.213	-0.999	0.001
392338550	IPO11	importin 11	-0.212	-0.984	0.016
415703079	NEBL	nebulette	-0.211	-0.970	0.030
18959250	PRKCD	protein kinase C delta	-0.210	-0.959	0.041
149058126	ALDH9A1	aldehyde dehydrogenase 9 family member A1	-0.208	-0.980	0.020
148667192	LRTM2	leucine rich repeats and transmembrane domains 2	-0.207	-0.977	0.023
6981504	ATXN1	ataxin 1	-0.204	-0.953	0.047
148747414	GDA	guanine deaminase	-0.203	-0.994	0.006
564391231	SERPINB9	serpin family B member 9	-0.203	-0.985	0.015
55741502	ACAT2	acetyl-CoA acetyltransferase 2	-0.202	-0.998	0.002
48675867	PLPP3	phospholipid phosphatase 3	-0.201	-0.981	0.019
68163417	FAHD1	fumarylacetoacetate hydrolase domain containing 1	-0.201	-0.950	0.050
11560055	KHDRBS3	KH RNA binding domain containing, signal transduction associated 3	-0.195	-0.993	0.007

564334053	SORCS1	sortilin related VPS10 domain containing receptor 1	-0.195	-0.999	0.001
17530977	ECHS1	enoyl-CoA hydratase, short chain 1	-0.191	-0.993	0.007
157822043	PLGRKT	plasminogen receptor with a C-terminal lysine	-0.191	-0.957	0.043
148696094	TUBGCP4	tubulin gamma complex associated protein 4	-0.189	-0.999	0.001
564399352	TAF9B	TATA-box binding protein associated factor 9b	-0.187	-0.978	0.022
672088942	ATP2B3	ATPase plasma membrane Ca2+ transporting 3	-0.186	-0.954	0.046
197209847	JAK1	Janus kinase 1	-0.185	-0.981	0.019
16923964	CNTN1	contactin 1	-0.185	-0.967	0.033
157817420	NRIP3	nuclear receptor interacting protein 3	-0.183	-0.964	0.036
76881830	Kcnip2	potassium voltage-gated channel interacting protein 2	-0.183	-0.981	0.019
32185285	BCL2L2	BCL2 like 2	-0.181	-0.986	0.014
187468990	DNAJB2	DnaJ heat shock protein family (Hsp40) member B2	-0.180	-0.963	0.037
39930507	KCNK15	potassium two pore domain channel subfamily K member 15	-0.180	-0.962	0.038
13929208	Scd2	stearoyl-Coenzyme A desaturase 2	-0.178	-0.984	0.016
201023331	MAPK11	mitogen-activated protein kinase 11	-0.176	-0.956	0.044
157818193	TTPAL	alpha tocopherol transfer protein like	-0.176	-0.965	0.035
564395567	NFATC3	nuclear factor of activated T cells 3	-0.173	-0.967	0.033
61557212	CIAO3	cytosolic iron-sulfur assembly component 3	-0.170	-0.976	0.024
396941666	Dync1i2	dynein cytoplasmic 1 intermediate chain 2	-0.169	-0.978	0.022
148668175	EDNRB	endothelin receptor type B	-0.168	-0.959	0.041
186910267	LYRM2	LYR motif containing 2	-0.167	-0.952	0.048
19705545	RAB3IL1	RAB3A interacting protein like 1	-0.166	-0.996	0.004
148747253	ATP1B1	ATPase Na+/K+ transporting subunit beta 1	-0.164	-0.966	0.034
19173766	LONP1	lon peptidase 1, mitochondrial	-0.164	-0.998	0.002
16758808	EPB41L3	erythrocyte membrane protein band 4.1 like 3	-0.159	-0.980	0.020
52345385	PDIA6	protein disulfide isomerase family A member 6	-0.159	-0.970	0.030
62078637	LCA5	lebercilin LCA5	-0.158	-0.955	0.045
209529636	PPA2	inorganic pyrophosphatase 2	-0.158	-0.988	0.012
18034785	ABCB6	ATP binding cassette subfamily B member 6 (Langereis blood group)	-0.158	-0.972	0.028

18266726	PAICS	phosphoribosylaminoimidazole carboxylase and phosphoribosylaminoimidazolesuccino carboxamide synthase	-0.155	-0.954	0.046
6649914	GDF11	growth differentiation factor 11	-0.154	-0.998	0.002
149032539	HECW1	HECT, C2 and WW domain containing E3 ubiquitin protein ligase 1	-0.150	-0.952	0.048
71043650	SRPK1	SRSF protein kinase 1	-0.150	-0.994	0.006
157819977	CERS4	ceramide synthase 4	-0.149	-0.977	0.023
113461996	COA5	cytochrome c oxidase assembly factor 5	-0.149	-0.954	0.046
50510837	KIAA1191	KIAA1191	-0.148	-0.991	0.009
109505096	NID1	nidogen 1	-0.148	-0.964	0.036
48976085	GM2A	GM2 ganglioside activator	-0.147	-0.980	0.020
148672025	MAP3K12	mitogen-activated protein kinase kinase kinase 12	-0.144	-0.980	0.020
16758736	NLGN1	neuroligin 1	-0.139	-0.965	0.035
157823401	PIGH	phosphatidylinositol glycan anchor biosynthesis class H	-0.135	-0.991	0.009
564345487	RINT1	RAD50 interactor 1	-0.132	-0.958	0.042
56605798	RNF167	ring finger protein 167	-0.131	-0.993	0.007
148747541	HNRNPU	heterogeneous nuclear ribonucleoprotein U	-0.130	-0.976	0.024
158749632	DBT	dihydrolipoamide branched chain transacylase E2	-0.129	-0.954	0.046
158711729	HACE1	HECT domain and ankyrin repeat containing E3 ubiquitin protein ligase 1	-0.128	-0.976	0.024
398650648	SLC8A1	solute carrier family 8 member A1	-0.127	-0.988	0.012
37359832	SCRN1	secernin 1	-0.127	-0.989	0.011
149035673	FAF1	Fas associated factor 1	-0.126	-0.954	0.046
77415383	HSPA8	heat shock protein family A (Hsp70) member 8	-0.125	-0.968	0.032
564339312	FUBP1	far upstream element binding protein 1	-0.123	-0.969	0.031
58865700	GRWD1	glutamate rich WD repeat containing 1	-0.123	-0.983	0.017
253683488	NTRK2	neurotrophic receptor tyrosine kinase 2	-0.121	-0.984	0.016
158254369	CDK10	cyclin dependent kinase 10	-0.121	-0.959	0.041
74229032	TPCN1	two pore segment channel 1	-0.121	-0.960	0.040
209870013	ITSN1	intersectin 1	-0.121	-0.954	0.046

71896549	UTP14A	UTP14A small subunit processome component	-0.118	-0.954	0.046
398303839	SH3GL2	SH3 domain containing GRB2 like 2, endophilin A1	-0.117	-0.997	0.003
8393390	GABRB3	gamma-aminobutyric acid type A receptor subunit beta3	-0.115	-0.979	0.021
52138635	ETFDH	electron transfer flavoprotein dehydrogenase	-0.115	-0.955	0.045
149059529	LOC100910558	uncharacterized LOC100910558	-0.114	-0.981	0.019
6978621	CCNG1	cyclin G1	-0.114	-0.997	0.003
78126149	SDF4	stromal cell derived factor 4	-0.113	-0.950	0.050
404247435	YLPM1	YLP motif containing 1	-0.113	-0.999	0.001
56090463	GORASP2	golgi reassembly stacking protein 2	-0.113	-0.973	0.027
157786602	NHP2	NHP2 ribonucleoprotein	-0.110	-0.995	0.005
57164133	NDUFC2	NADH:ubiquinone oxidoreductase subunit C2	-0.108	-0.956	0.044
274326692	UQCC3	ubiquinol-cytochrome c reductase complex assembly factor 3	-0.106	-0.994	0.006
58865936	SIKE1	suppressor of IKBKE 1	-0.100	-0.979	0.021
403377905	SRGAP2	SLIT-ROBO Rho GTPase activating protein 2	-0.098	-0.963	0.037
672057488	CD63	CD63 molecule	-0.096	-0.958	0.042
149060725	CEP19	centrosomal protein 19	-0.095	-0.976	0.024
154800420	GNL3L	G protein nucleolar 3 like	-0.093	-0.982	0.018
16258813	VHL	von Hippel-Lindau tumor suppressor	-0.089	-0.955	0.045
8980843	GRIPAP1	GRIP1 associated protein 1	-0.089	-0.969	0.031
16758578	DPP3	dipeptidyl peptidase 3	-0.084	-0.982	0.018
50054266	NLN	neurolysin	-0.082	-0.988	0.012
564343911	RPN2	ribophorin II	-0.076	-0.965	0.035
297206894	E4F1	E4F transcription factor 1	-0.074	-0.952	0.048
564375060	SLC39A11	solute carrier family 39 member 11	-0.070	-0.994	0.006
148747528	PTK2B	protein tyrosine kinase 2 beta	-0.066	-0.998	0.002
255918181	NUS1	NUS1 dehydrodolichyl diphosphate synthase subunit	-0.062	-0.989	0.011
20302113	STIP1	stress induced phosphoprotein 1	-0.060	-0.981	0.019
213688386	GTF2E1	general transcription factor IIE subunit 1	-0.058	-0.953	0.047
13385318	KDELR2	KDEL endoplasmic reticulum protein retention receptor 2	-0.057	-0.963	0.037
149049470	TPI1	triosephosphate isomerase 1	-0.057	-0.982	0.018
162287208	FADS1	fatty acid desaturase 1	-0.050	-0.998	0.002
76443681	USP11	ubiquitin specific peptidase 11	0.053	0.994	0.006
149039803	UBQLN1	ubiquilin 1	0.065	0.962	0.038

157822779	DNAJC11	DnaJ heat shock protein family (Hsp40) member C11	0.066	0.989	0.011
564353714	FBXO42	F-box protein 42	0.074	0.982	0.018
564326269	RPL28	ribosomal protein L28	0.075	0.959	0.041
157817783	SNX18	sorting nexin 18	0.079	0.984	0.016
83649695	SMIM14	small integral membrane protein 14	0.082	0.982	0.018
60360532	OSBPL6	oxysterol binding protein like 6	0.083	0.972	0.028
214010118	TMEM59	transmembrane protein 59	0.085	0.980	0.020
6978449	ADD2	adducin 2	0.085	0.970	0.030
290560659	ZNF609	zinc finger protein 609	0.090	0.999	0.001
564363529	NCAM1	neural cell adhesion molecule 1	0.091	0.968	0.032
149038024	RIPOR1	RHO family interacting cell polarization regulator 1	0.091	1.000	0.000
564332984	OSBP	oxysterol binding protein	0.094	0.990	0.010
14389301	SMPD2	sphingomyelin phosphodiesterase 2	0.096	0.971	0.029
166064004	GTF3A	general transcription factor IIIA	0.101	0.965	0.035
62543537	TBC1D10A	TBC1 domain family member 10A	0.108	0.964	0.036
157818159	AAR2	AAR2 splicing factor	0.110	0.958	0.042
157786720	HIVEP1	HIVEP zinc finger 1	0.111	0.951	0.049
30794434	SRRM4	serine/arginine repetitive matrix 4	0.112	0.969	0.031
157817811	C5orf22	chromosome 5 open reading frame 22	0.113	0.963	0.037
170295834	NDUFA10	NADH:ubiquinone oxidoreductase subunit A10	0.114	0.965	0.035
564360651	LRRC14	leucine rich repeat containing 14	0.115	0.954	0.046
51948396	TUSC3	tumor suppressor candidate 3	0.116	0.960	0.040
274321371	CRLF3	cytokine receptor like factor 3	0.118	0.974	0.026
18959272	KCNQ2	potassium voltage-gated channel subfamily Q member 2	0.119	0.970	0.030
293344794	FAM160B1	family with sequence similarity 160 member B1	0.121	0.972	0.028
114326177	SHMT1	serine hydroxymethyltransferase 1	0.123	0.968	0.032
157818629	HEYL	hes related family bHLH transcription factor with YRPW motif like	0.126	0.956	0.044
58866022	MGAT4A	alpha-1,3-mannosyl-glycoprotein 4-beta-N-acetylglucosaminyltransferase A	0.127	0.983	0.017
454601639	NCOA6	nuclear receptor coactivator 6	0.127	0.975	0.025
672068548	SUPT6H	SPT6 homolog, histone chaperone and transcription elongation factor	0.127	0.993	0.007
564331450	EEF1AKMT2	EEF1A lysine methyltransferase 2	0.129	0.986	0.014
564363852	SNUPN	snurportin 1	0.131	0.971	0.029
300797157	TBC1D8	TBC1 domain family member 8	0.131	0.964	0.036
60359978	KIF3C	kinesin family member 3C	0.131	0.965	0.035

148693260	TIMM29	translocase of inner mitochondrial membrane 29	0.131	0.972	0.028
157824037	USP4	ubiquitin specific peptidase 4	0.131	0.982	0.018
74139306	TMED9	transmembrane p24 trafficking protein 9	0.143	0.988	0.012
9624979	ENSA	endosulfine alpha	0.144	0.955	0.045
157820585	SART3	spliceosome associated factor 3, U4/U6 recycling protein	0.146	0.979	0.021
149064388	Hmgxb3	HMG-box containing 3	0.147	0.960	0.040
40786455	BPGM	bisphosphoglycerate mutase	0.150	0.993	0.007
564311031	CLPP	caseinolytic mitochondrial matrix peptidase proteolytic subunit	0.150	0.950	0.050
300253233	LEMD3	LEM domain containing 3	0.154	0.988	0.012
209863130	SEMA3F	semaphorin 3F	0.155	0.964	0.036
50510427	IP6K1	inositol hexakisphosphate kinase 1	0.156	0.976	0.024
77917548	DUS3L	dihydrouridine synthase 3 like	0.158	0.997	0.003
300793740	TANC2	tetratricopeptide repeat, ankyrin repeat and coiled-coil containing 2	0.158	0.958	0.042
672043520	PI4KB	phosphatidylinositol 4-kinase beta	0.159	0.957	0.043
58219518	RND2	Rho family GTPase 2	0.161	0.950	0.050
149033480	Zfp956	zinc finger protein 956	0.163	0.985	0.015
672072928	CUX2	cut like homeobox 2	0.164	0.994	0.006
672067460	RGD1560464	similar to hypothetical protein FLJ38426	0.164	0.953	0.047
60359854	POLDIP3	DNA polymerase delta interacting protein 3	0.164	0.962	0.038
564340867	MMADHC	metabolism of cobalamin associated D	0.165	0.977	0.023
76559929	NOC2L	NOC2 like nucleolar associated transcriptional repressor	0.165	0.992	0.008
293359997	SGPP1	sphingosine-1-phosphate phosphatase 1	0.170	0.961	0.039
157818643	KCTD3	potassium channel tetramerization domain containing 3	0.170	0.973	0.027
33356154	UBE2H	ubiquitin conjugating enzyme E2 H	0.172	0.952	0.048
56605790	HCFC2	host cell factor C2	0.173	0.975	0.025
568972665	TSPOAP1	TSPO associated protein 1	0.173	0.959	0.041
564298823	EML3	EMAP like 3	0.177	0.952	0.048
149054120	ORMDL3	ORMDL sphingolipid biosynthesis regulator 3	0.179	0.978	0.022
149044006	TEDC1	tubulin epsilon and delta complex 1	0.179	0.971	0.029
392333209	DLG5	discs large MAGUK scaffold protein 5	0.180	0.988	0.012
62079005	SLAIN1	SLAIN motif family member 1	0.180	0.960	0.040

157817773	ZNF641	zinc finger protein 641	0.183	0.973	0.027
762006019	FAM8A1	family with sequence similarity 8 member A1	0.185	0.978	0.022
564384443	EIF4ENIF1	eukaryotic translation initiation factor 4E nuclear import factor 1	0.187	0.956	0.044
66730335	SUMO3	small ubiquitin like modifier 3	0.188	0.977	0.023
672085227	USP10	ubiquitin specific peptidase 10	0.191	0.991	0.009
148683335	SLC25A44	solute carrier family 25 member 44	0.191	0.991	0.009
197313795	MTX1	metaxin 1	0.192	0.967	0.033
157823197	NDUFB7	NADH:ubiquinone oxidoreductase subunit B7	0.194	0.994	0.006
157821953	NXPE3	neurexophilin and PC-esterase domain family member 3	0.197	0.966	0.034
157820969	SBNO2	strawberry notch homolog 2	0.197	0.985	0.015
157820401	ABHD2	abhydrolase domain containing 2, acylglycerol lipase	0.197	0.975	0.025
57164107	NIPSNAP3A	nipsnap homolog 3A	0.199	0.990	0.010
157821579	BICD1	BICD cargo adaptor 1	0.202	0.977	0.023
219277692	NDUFB2	NADH:ubiquinone oxidoreductase subunit B2	0.203	0.972	0.028
61557021	BFAR	bifunctional apoptosis regulator	0.204	0.975	0.025
564383487	SLAIN2	SLAIN motif family member 2	0.206	0.960	0.040
157817674	ATP5MF-PTCD1	ATP5MF-PTCD1 readthrough	0.207	0.990	0.010
61556748	TSPYL1	TSPY like 1	0.211	0.992	0.008
84781638	KLHL25	kelch like family member 25	0.215	0.969	0.031
189163477	SCAF4	SR-related CTD associated factor 4	0.216	0.986	0.014
396080328	ADCYAP1R1	ADCYAP receptor type I	0.217	0.972	0.028
66911118	NFX1	nuclear transcription factor, X-box binding 1	0.217	0.960	0.040
213688373	GADD45GIP1	GADD45G interacting protein 1	0.218	0.958	0.042
765099233	LMNB2	lamin B2	0.219	0.968	0.032
2804296	CDH8	cadherin 8	0.221	0.965	0.035
288541353	CMTM4	CKLF like MARVEL transmembrane domain containing 4	0.222	0.977	0.023
148669751	SMNDC1	survival motor neuron domain containing 1	0.223	0.971	0.029
117940043	MED22	mediator complex subunit 22	0.225	0.964	0.036
148696931	ARRDC2	arrestin domain containing 2	0.227	0.972	0.028
148670791	ZFYVE1	zinc finger FYVE-type containing 1	0.232	0.965	0.035
564340133	GTF3C4	general transcription factor IIIC subunit 4	0.236	0.960	0.040
37360264	TRMT6	tRNA methyltransferase 6	0.236	0.977	0.023
40018556	NOB1	NIN1 (RPN12) binding protein 1 homolog	0.237	0.969	0.031

564359486	TBC1D30	TBC1 domain family member 30	0.237	0.975	0.025
31415868	MAFB	MAF bZIP transcription factor B	0.246	0.957	0.043
157821413	USP30	ubiquitin specific peptidase 30	0.250	0.968	0.032
157823719	TRAIP	TRAF interacting protein	0.252	0.980	0.020
209954792	PDCD2	programmed cell death 2	0.254	0.970	0.030
157821283	C19orf47	chromosome 19 open reading frame 47	0.257	0.962	0.038
119388826	TFPT	TCF3 fusion partner	0.258	0.959	0.041
564367958	SEMA4C	semaphorin 4C	0.258	0.978	0.022
281427178	CEP76	centrosomal protein 76	0.262	0.991	0.009
67078454	SLC25A51	solute carrier family 25 member 51	0.263	0.985	0.015
197382256	PHF12	PHD finger protein 12	0.263	0.980	0.020
380877082	NAXE	NAD(P)HX epimerase	0.263	0.950	0.050
564353880	DDI2	DNA damage inducible 1 homolog 2	0.264	0.981	0.019
148696370	PANK2	pantothenate kinase 2	0.270	0.989	0.011
564394925	TENT4B	terminal nucleotidyltransferase 4B	0.276	0.960	0.040
197386048	PTRHD1	peptidyl-tRNA hydrolase domain containing 1	0.280	0.953	0.047
157821325	TWNK	twinkle mtDNA helicase	0.283	0.979	0.021
149052738	RGD1561277	RGD1561277	0.285	0.998	0.002
62078733	MAK16	MAK16 homolog	0.287	0.985	0.015
672020915	VCPKMT	valosin containing protein lysine methyltransferase	0.288	0.955	0.045
76559919	N4BP3	NEDD4 binding protein 3	0.292	0.997	0.003
166795897	PIMREG	PICALM interacting mitotic regulator	0.293	0.986	0.014
56789732	VSTM5	V-set and transmembrane domain containing 5	0.295	0.979	0.021
68163385	GPATCH4	G-patch domain containing 4	0.297	0.966	0.034
157821997	MED28	mediator complex subunit 28	0.297	0.962	0.038
157822519	CBLN4	cerebellin 4 precursor	0.299	0.997	0.003
157819365	TBC1D25	TBC1 domain family member 25	0.302	0.991	0.009
157819315	OSBPL11	oxysterol binding protein like 11	0.306	0.991	0.009
51491900	TOR1A	torsin family 1 member A	0.308	0.994	0.006
56605776	TAF11	TATA-box binding protein associated factor 11	0.309	0.951	0.049
50510821	AMIGO1	adhesion molecule with Ig like domain 1	0.310	0.961	0.039
300793780	ZNF251	zinc finger protein 251	0.321	0.996	0.004
149051028	RNF144A	ring finger protein 144A	0.322	0.986	0.014
41386747	ZC3H18	zinc finger CCCH-type containing 18	0.322	0.999	0.001
672030183	H2AC12	H2A clustered histone 12	0.323	0.998	0.002
76362828	TEF	TEF transcription factor, PAR bZIP family member	0.327	0.966	0.034

68163537	NXPE4	neurexophilin and PC-esterase domain family member 4	0.329	0.973	0.027
582015198	CRY2	cryptochrome circadian regulator 2	0.332	0.974	0.026
564333920	PPRC1	PPARG related coactivator 1	0.333	0.977	0.023
564307081	ATXN7L1	ataxin 7 like 1	0.339	0.966	0.034
564350836	MELK	maternal embryonic leucine zipper kinase	0.341	0.983	0.017
40018598	ANGPTL4	angiopoietin like 4	0.356	0.971	0.029
57528321	RIOK2	RIO kinase 2	0.360	0.999	0.001
157822027	CSRNP2	cysteine and serine rich nuclear protein 2	0.361	0.997	0.003
300794219	OPN3	opsin 3	0.362	0.950	0.050
300797828	KAT14	lysine acetyltransferase 14	0.365	0.979	0.021
157817720	SLC16A14	solute carrier family 16 member 14	0.374	0.978	0.022
213972545	MXD1	MAX dimerization protein 1	0.378	0.982	0.018
164565364	ITPKB	inositol-trisphosphate 3-kinase B	0.380	0.954	0.046
14388593	SPATA2	spermatogenesis associated 2	0.385	0.986	0.014
8392855	ADCYAP1	adenylate cyclase activating polypeptide 1	0.397	0.962	0.038
157821403	RASSF7	Ras association domain family member 7	0.399	0.998	0.002
293345066	PPIL6	peptidylprolyl isomerase like 6	0.408	0.957	0.043
66730347	PTPRCAP	protein tyrosine phosphatase receptor type C associated protein	0.415	0.967	0.033
38454286	STIMATE-MUSTN1	STIMATE-MUSTN1 readthrough	0.425	0.959	0.041
564311452	TMEM131	transmembrane protein 131	0.443	0.965	0.035
3676248	Prim1	DNA primase subunit 1	0.450	0.961	0.039
53850630	LOC100362724/ MGC95208	similar to 4930453N24Rik protein	0.456	0.979	0.021
255708448	KATNA1	katanin catalytic subunit A1	0.466	0.976	0.024
148706598	PKDCC	protein kinase domain containing, cytoplasmic	0.469	0.988	0.012
51948492	NUDT19	nudix hydrolase 19	0.470	0.981	0.019
70912374	CCNQ	cyclin Q	0.479	0.977	0.023
19424300	GCHFR	GTP cyclohydrolase I feedback regulator	0.519	0.965	0.035
89145411	SULT2B1	sulfotransferase family 2B member 1	0.524	0.994	0.006
404434380	ZNF133	zinc finger protein 133	0.530	0.987	0.013
148687591	TMEM132D	transmembrane protein 132D	0.550	0.981	0.019
213688370	EXOSC7	exosome component 7	0.551	0.971	0.029
219879771	PGAP3	post-GPI attachment to proteins phospholipase 3	0.560	0.997	0.003
149023178	CEP152	centrosomal protein 152	0.572	0.963	0.037

148664537	Gm10269	ribosomal protein L35 pseudogene	0.582	0.994	0.006
511094004	RUNX2	RUNX family transcription factor 2	0.585	0.962	0.038
24415396	GPR3	G protein-coupled receptor 3	0.604	0.962	0.038
13994119	KHK	ketohexokinase	0.612	0.958	0.042
22122541	LRRC3B	leucine rich repeat containing 3B	0.615	0.982	0.018
157822359	PELI2	pellino E3 ubiquitin protein ligase family member 2	0.620	0.998	0.002
157820433	CPEB1	cytoplasmic polyadenylation element binding protein 1	0.624	0.971	0.029
1083798	Bmpr1b	bone morphogenetic protein receptor type 1B	0.630	0.979	0.021
197386066	ZNF784	zinc finger protein 784	0.633	0.997	0.003
157823803	DOK3	docking protein 3	0.639	0.976	0.024
149025186	RPS6KL1	ribosomal protein S6 kinase like 1	0.661	0.980	0.020
392354293	Hmgb3	high mobility group box 3	0.709	0.997	0.003
80861398	CRY1	cryptochrome circadian regulator 1	0.720	0.992	0.008
38454200	CHDH	choline dehydrogenase	0.734	0.973	0.027
293339965	RAB11FIP3	RAB11 family interacting protein 3	0.750	0.993	0.007
672086719	FAM184A	family with sequence similarity 184 member A	0.836	0.986	0.014
189011634	ARMC7	armadillo repeat containing 7	0.850	0.968	0.032
672025117	MBTD1	mbt domain containing 1	0.886	0.983	0.017
392339806	CFAP69	cilia and flagella associated protein 69	0.915	0.966	0.034
9506775	HES2	hes family bHLH transcription factor 2	0.963	0.962	0.038
157821687	NEURL2	neuralized E3 ubiquitin protein ligase 2	0.966	0.972	0.028
148693657	DDX6	DEAD-box helicase 6	0.969	0.991	0.009
148235584	CLEC4A	C-type lectin domain family 4 member A	1.000	0.962	0.038
149065466	ARHGEF5	Rho guanine nucleotide exchange factor 5	1.000	0.962	0.038
82654234	LILRA6	leukocyte immunoglobulin like receptor A6	1.000	0.984	0.016
404501522	NXNL1	nucleoredoxin like 1	1.037	0.962	0.038
149067796	TMEM219	transmembrane protein 219	1.064	0.962	0.038
19424314	KCNE2	potassium voltage-gated channel subfamily E regulatory subunit 2	1.066	0.999	0.001
564296988	ZNF235	zinc finger protein 235	1.072	0.966	0.034
188536090	FAM241B	family with sequence similarity 241 member B	1.127	0.980	0.020
62078917	PAQR5	progestin and adipoQ receptor family member 5	1.181	1.000	0.000
6978493	ALOX5	arachidonate 5-lipoxygenase	1.193	0.989	0.011

157787081	WNT1	Wnt family member 1	1.222	0.982	0.018
569012000	KLF8	Kruppel like factor 8	1.406	0.973	0.027
157817264	ANKRD23	ankyrin repeat domain 23	1.505	0.996	0.004
158533972	SPTA1	spectrin alpha, erythrocytic 1	1.585	0.962	0.038
149058209	SELE	selectin E	1.585	0.962	0.038
157818463	Zfp93	zinc finger protein 93	1.597	0.958	0.042
672013187	DMWD	DM1 locus, WD repeat containing	1.605	0.983	0.017
41054896	FUT7	fucosyltransferase 7	1.700	0.975	0.025
568990288	NIPBL	NIPBL cohesin loading factor	1.705	0.960	0.040
157819799	IQCH	IQ motif containing H	1.716	0.979	0.021
576080555	GAPDH	glyceraldehyde-3-phosphate dehydrogenase	1.720	0.959	0.041
149032888	LOC100910237	uncharacterized LOC100910237	1.751	0.986	0.014
157820135	CHRDL2	chordin like 2	1.762	0.984	0.016
564318930	WDR17	WD repeat domain 17	1.807	0.973	0.027
157821823	Ngp	neutrophilic granule protein	1.807	0.973	0.027
392342449	PRSS56	serine protease 56	1.848	0.966	0.034
149042883	LOC100365365	rCG32328-like	1.861	0.993	0.007
16758254	CNGA1	cyclic nucleotide gated channel subunit alpha 1	1.874	0.965	0.035
189181736	LAD1	ladinin 1	1.874	0.977	0.023
197381585	Urah	urate (5-hydroxyiso-) hydrolase	1.976	0.962	0.038
160961485	MYLK3	myosin light chain kinase 3	2.000	0.962	0.038
672070295	BAHCC1	BAH domain and coiled-coil containing 1	2.083	0.982	0.018
28972866	CSMD3	CUB and Sushi multiple domains 3	2.140	0.994	0.006
293352381	PAN3	poly(A) specific ribonuclease subunit PAN3	2.239	0.989	0.011
197385133	RGD1561157	RGD1561157	2.303	0.964	0.036
71896592	IGFALS	insulin like growth factor binding protein acid labile subunit	2.322	0.962	0.038
8393941	PADI4	peptidyl arginine deiminase 4	2.322	0.962	0.038
564324736	L3MBTL3	L3MBTL histone methyl-lysine binding protein 3	2.353	0.959	0.041
157818163	POF1B	POF1B actin binding protein	2.392	0.997	0.003
564329376	SRPK3	SRSF protein kinase 3	2.585	0.962	0.038
58866038	XKRX	XK related X-linked	2.585	0.962	0.038
11120690	NR1H4	nuclear receptor subfamily 1 group H member 4	2.585	0.962	0.038
13540693	MYOC	myocilin	2.585	0.962	0.038
57222314	OAS3	2'-5'-oligoadenylate synthetase 3	2.585	0.962	0.038
8394529	VDR	vitamin D receptor	2.585	0.962	0.038
25742760	AMH	anti-Mullerian hormone	2.807	0.962	0.038
157787002	Dpt	dermatopontin	2.807	0.962	0.038

197384923	C1orf87	chromosome 1 open reading frame 87	3.000	0.962	0.038
13591993	MMP9	matrix metallopeptidase 9	3.000	0.962	0.038
300796937	ESPNL	espin like	3.000	0.962	0.038
564347547	LOC103690120	probable N-acetyltransferase CML1	3.030	0.997	0.003
281332212	SH2D4B	SH2 domain containing 4B	3.170	0.962	0.038
260099641	MSH5	mutS homolog 5	3.170	0.962	0.038
564392795	MOCOS	molybdenum cofactor sulfurase	3.248	0.976	0.024
61556961	THEG	theg spermatid protein	3.322	0.962	0.038
28174920	RPL17	ribosomal protein L17	3.389	0.976	0.024
569009290	TENM1	teneurin transmembrane protein 1	3.450	0.973	0.027
25282405	BPIFA1	BPI fold containing family A member 1	3.459	0.962	0.038
16758550	BCL2L10	BCL2 like 10	3.459	0.962	0.038
148670929	BATF	basic leucine zipper ATF-like transcription factor	3.700	0.962	0.038
62078779	ORAI3	ORAI calcium release-activated calcium modulator 3	3.807	0.952	0.048
21245088	Ly6a (includes others)	lymphocyte antigen 6 complex, locus A	3.807	0.962	0.038
148747510	BAAT	bile acid-CoA:amino acid N-acyltransferase	3.807	0.962	0.038
158187515	OAZ3	ornithine decarboxylase antizyme 3	3.807	0.962	0.038
672052120	RBM12B	RNA binding motif protein 12B	3.907	0.971	0.029
564309734	IGSF9B	immunoglobulin superfamily member 9B	4.173	0.966	0.034
8393641	AADAT	aminoadipate aminotransferase	4.248	0.962	0.038
27545443	CEACAM4	CEA cell adhesion molecule 4	4.392	0.977	0.023
149038931	CNTRL	centriolin	4.492	0.956	0.044
341940965	MOS	MOS proto-oncogene, serine/threonine kinase	4.492	0.972	0.028
9506733	GJB5	gap junction protein beta 5	4.907	0.962	0.038
59709455	EPOR	erythropoietin receptor	5.170	0.957	0.043
8394516	PLAUR	plasminogen activator, urokinase receptor	5.229	0.963	0.037
16758218	Hamp	hepcidin antimicrobial peptide	5.267	0.965	0.035
20301998	PROK2	prokineticin 2	5.358	0.978	0.022
672052120	RBM12B	RNA binding motif protein 12B	6.366	0.961	0.039
293347435	PTPRD	protein tyrosine phosphatase receptor type D	7.710	0.972	0.028
149057336	ZSCAN2	zinc finger and SCAN domain containing 2	7.758	0.978	0.022
62650795	DACT1	dishevelled binding antagonist of beta catenin 1	7.762	0.977	0.023
109472884	UBE3C	ubiquitin protein ligase E3C	8.197	0.969	0.031

672029702	TUT7	terminal uridylyl transferase 7	8.441	0.951	0.049

Supplementary Table S14. The list of genes that are differentially expressed in the offspring hippocampus in response to prenatal BPA exposure that exhibited the changes in the expression levels correlated with the number of neurites (≥ 20 µm) of primary hippocampal cells at DIV7. The transcriptome profiling data of DEGs in male and female rat offspring prenatally exposed to BPA (n = 6, male pups n = 3 and female pups n = 3, from independent litters) or the vehicle control (n = 6, male pups n = 3 and female pups n = 3, from independent litters) were obtained and used for the PTM analyses to identify DEGs that exhibited the changes in the expression levels correlated with the number of neurites (≥ 20 µm) of primary hippocampal cells at DIV7.

ID	Symbol	Entrez Gene Name	log2(FC)	R values	P-values
293347435	PTPRD	protein tyrosine phosphatase receptor type D	-7.731	-0.986	0.014
564307173	HEATR5A	HEAT repeat containing 5A	-6.735	-0.957	0.043
564310188	IGDCC4	immunoglobulin superfamily DCC subclass member 4	-6.728	-0.984	0.016
564314389	DZIP3	DAZ interacting zinc finger protein 3	-6.700	-0.974	0.026
672031167	C19orf57	chromosome 19 open reading frame 57	-6.476	-0.971	0.029
149020633	TAF1D	TATA-box binding protein associated factor, RNA polymerase I subunit D	-6.476	-0.970	0.030
149052470	ZNF454	zinc finger protein 454	-5.700	-0.968	0.032
157818475	SMIM22	small integral membrane protein 22	-5.615	-0.982	0.018
754169051	EPPIN	epididymal peptidase inhibitor	-5.066	-0.961	0.039
197384778	Snorc	secondary ossification center associated regulator of chondrocyte maturation	-5.044	-0.983	0.017
564323305	LOC681300	similar to CXXC finger 5	-4.672	-0.969	0.031
300798104	IFNLR1	interferon lambda receptor 1	-4.248	-0.983	0.017
564298047	GDPD5	glycerophosphodiester phosphodiesterase domain containing 5	-4.163	-0.955	0.045
114145748	LOC680227	LRRGT00193	-3.907	-0.984	0.016
194474016	SLC30A8	solute carrier family 30 member 8	-3.807	-0.981	0.019
8392926	ASGR2	asialoglycoprotein receptor 2	-3.700	-0.986	0.014
148675704	TBX15	T-box transcription factor 15	-3.268	-0.973	0.027
564324736	L3MBTL3	L3MBTL histone methyl-lysine binding protein 3	-3.262	-0.986	0.014
564375434	CEP295NL	CEP295 N-terminal like	-3.248	-0.978	0.022
12408310	N5	DNA binding protein N5	-3.000	-0.975	0.025
20302091	PLB1	phospholipase B1	-2.907	-0.981	0.019
157821903	Slc7a15	solute carrier family 7 (cationic amino acid transporter, y+ system), member 15	-2.907	-0.981	0.019

564355126	ADGRF3	adhesion G protein-coupled receptor F3	-2.907	-0.954	0.046
157822121	LRMDA	leucine rich melanocyte differentiation associated	-2.747	-0.989	0.011
156071424	Vom2r18 (includes others)	vomeronasal 2 receptor, 18	-2.700	-0.971	0.029
51948496	SLC22A18	solute carrier family 22 member 18	-2.687	-0.957	0.043
672032217	REPS2	RALBP1 associated Eps domain containing 2	-2.683	-0.983	0.017
564297387	Zfp658	zinc finger protein 658	-2.369	-0.972	0.028
157820151	ERAS	ES cell expressed Ras	-2.248	-0.990	0.010
564320724	Fbxo38	F-box protein 38	-2.220	-0.985	0.015
300798413	FSD2	fibronectin type III and SPRY domain containing 2	-2.170	-0.975	0.025
66730349	SPIB	Spi-B transcription factor	-2.170	-0.969	0.031
25742828	SCN7A	sodium voltage-gated channel alpha subunit 7	-2.129	-0.989	0.011
300797305	TMEM45A	transmembrane protein 45A	-2.093	-0.997	0.003
148672128	SMAGP	small cell adhesion glycoprotein	-2.077	-0.952	0.048
56676350	PRSS35	serine protease 35	-1.962	-0.977	0.023
157820217	Gsta4	glutathione S-transferase, alpha 4	-1.930	-0.953	0.047
117647214	EDN3	endothelin 3	-1.913	-0.955	0.045
81295367	Abcg3	ATP binding cassette subfamily G member 3	-1.898	-0.952	0.048
672029702	TUT7	terminal uridylyl transferase 7	-1.854	-0.997	0.003
13592031	PTGER2	prostaglandin E receptor 2	-1.845	-0.978	0.022
297374767	TPSAB1/TPSB2	tryptase alpha/beta 1	-1.834	-0.997	0.003
569009290	TENM1*	teneurin transmembrane protein 1	-1.824	-0.958	0.042
149064260	PRDM6	PR/SET domain 6	-1.816	-0.989	0.011
19424240	PCSK4	proprotein convertase subtilisin/kexin type 4	-1.740	-0.964	0.036
480306394	Mcpt4	mast cell protease 4	-1.739	-0.985	0.015
153792385	Vom2r34	vomeronasal 2 receptor, 34	-1.648	-0.991	0.009
58331159	GSTA3	glutathione S-transferase alpha 3	-1.603	-0.987	0.013
157821527	RHOD	ras homolog family member D	-1.597	-0.973	0.027
295391913	LOC100366054	Da1-10-like	-1.590	-0.972	0.028
157819393	NNMT	nicotinamide N-methyltransferase	-1.585	-0.990	0.010
209870037	INSRR	insulin receptor related receptor	-1.585	-0.986	0.014
62079089	MALL	mal, T cell differentiation protein like	-1.585	-0.968	0.032
13929066	CPZ	carboxypeptidase Z	-1.549	-0.955	0.045
158186711	F13A1	coagulation factor XIII A chain	-1.510	-0.970	0.030
72255569	Abca17	ATP-binding cassette, sub-family A (ABC1), member 17	-1.495	-0.959	0.041

13929126	GALNT5	polypeptide N-acetylgalactosaminyltransferase 5	-1.478	-0.987	0.013
71043750	SYNGR4	synaptogyrin 4	-1.464	-0.961	0.039
81295349	SLC52A3	solute carrier family 52 member 3	-1.453	-0.971	0.029
283806636	ZNF831	zinc finger protein 831	-1.433	-0.976	0.024
19924087	Akr1c14	aldo-keto reductase family 1, member C14	-1.406	-0.952	0.048
157822105	SLC49A3	solute carrier family 49 member 3	-1.389	-0.983	0.017
18426832	IL23A	interleukin 23 subunit alpha	-1.381	-0.962	0.038
672025117	MBTD1	mbt domain containing 1	-1.344	-0.989	0.011
29789044	SNAI2	snail family transcriptional repressor 2	-1.328	-0.963	0.037
157823345	LRR1	leucine rich repeat protein 1	-1.322	-0.986	0.014
57114286	HLA-DRB5	major histocompatibility complex, class II, DR beta 5	-1.284	-0.959	0.041
71043878	PROCR	protein C receptor	-1.270	-0.986	0.014
62656582	KIAA0100	KIAA0100	-1.266	-0.972	0.028
187281975	DENND1C	DENN domain containing 1C	-1.216	-0.965	0.035
16758338	FTCD	formimidoyltransferase cyclodeaminase	-1.208	-0.966	0.034
56119141	BTK	Bruton tyrosine kinase	-1.205	-0.987	0.013
155369293	AEBP1	AE binding protein 1	-1.198	-0.958	0.042
564300462	DCHS2	dachsous cadherin-related 2	-1.190	-0.956	0.044
392334475	Myb	MYB proto-oncogene, transcription factor	-1.175	-0.996	0.004
71795615	UPP1	uridine phosphorylase 1	-1.167	-0.985	0.015
281332082	THBS2	thrombospondin 2	-1.164	-0.954	0.046
157823801	SLC50A1	solute carrier family 50 member 1	-1.136	-0.971	0.029
60223053	SEPTIN1	septin 1	-1.133	-0.988	0.012
149053909	COL1A1	collagen type I alpha 1 chain	-1.092	-0.969	0.031
197384727	Smco4	single-pass membrane protein with coiled-coil domains 4	-1.065	-0.973	0.027
197386139	SSC5D	scavenger receptor cysteine rich family member with 5 domains	-1.060	-0.994	0.006
11560040	PTGDR	prostaglandin D2 receptor	-1.037	-0.985	0.015
300797728	MGST3	microsomal glutathione S-transferase 3	-1.011	-0.997	0.003
569009290	TENM1*	teneurin transmembrane protein 1	-0.985	-0.971	0.029
300795496	LAYN	layilin	-0.983	-0.960	0.040
158341649	FAM227B	family with sequence similarity 227 member B	-0.981	-0.968	0.032
215276950	PKP2	plakophilin 2	-0.973	-0.984	0.016
20302089	GABRR3	gamma-aminobutyric acid type A receptor subunit rho3 (gene/pseudogene)	-0.972	-0.958	0.042

564340633	OLFML2A	olfactomedin like 2A	-0.968	-0.951	0.049
157818989	LRRC71	leucine rich repeat containing 71	-0.952	-0.983	0.017
157818275	KCNG4	potassium voltage-gated channel modifier subfamily G member 4	-0.952	-0.961	0.039
157823055	EGFL6	EGF like domain multiple 6	-0.947	-0.951	0.049
198442873	CDC14A	cell division cycle 14A	-0.936	-0.978	0.022
62945330	SLC8B1	solute carrier family 8 member B1	-0.929	-0.986	0.014
157819487	TACO1	translational activator of cytochrome c oxidase I	-0.917	-0.998	0.002
47059114	LTB	lymphotoxin beta	-0.916	-0.964	0.036
564325648	Zfp54	zinc finger protein 54	-0.907	-0.957	0.043
564318923	WDR17	WD repeat domain 17	-0.904	-0.994	0.006
13540656	EMP3	epithelial membrane protein 3	-0.883	-0.950	0.050
57114338	SCN4B	sodium voltage-gated channel beta subunit 4	-0.859	-0.974	0.026
149031998	ACVRL1	activin A receptor like type 1	-0.852	-0.991	0.009
157822457	SYNC	syncoilin, intermediate filament protein	-0.833	-0.958	0.042
56605720	GADD45B	growth arrest and DNA damage inducible beta	-0.828	-0.988	0.012
149038682	Srgn	serglycin	-0.823	-0.967	0.033
157817065	KCNK16	potassium two pore domain channel subfamily K member 16	-0.807	-0.986	0.014
259089426	AGER	advanced glycosylation end-product specific receptor	-0.804	-0.986	0.014
307548437	NYAP2	neuronal tyrosine-phosphorylated phosphoinositide-3-kinase adaptor 2	-0.780	-0.972	0.028
942523340	CAPRIN2	caprin family member 2	-0.774	-0.988	0.012
157819467	C19orf71	chromosome 19 open reading frame 71	-0.769	-0.983	0.017
27465577	Cyp4f16/Cyp4f37	cytochrome P450, family 4, subfamily f, polypeptide 16	-0.758	-0.963	0.037
13591914	ANPEP	alanyl aminopeptidase, membrane	-0.757	-0.991	0.009
672069802	C1QTNF1	C1q and TNF related 1	-0.749	-0.974	0.026
157786850	TUBD1	tubulin delta 1	-0.740	-0.975	0.025
56119147	ARRDC3	arrestin domain containing 3	-0.732	-0.975	0.025
13929156	MYBPH	myosin binding protein H	-0.728	-0.962	0.038
208973286	RBM46	RNA binding motif protein 46	-0.724	-0.971	0.029
16758232	PLCB2	phospholipase C beta 2	-0.721	-0.995	0.005
149047075	Spaca6	sperm acrosome associated 6	-0.715	-0.989	0.011
564378828	TFR2	transferrin receptor 2	-0.707	-0.958	0.042
55742713	ECM1	extracellular matrix protein 1	-0.699	-0.952	0.048
148671621	VIP	vasoactive intestinal peptide	-0.696	-0.956	0.044
293343546	C5orf49	chromosome 5 open reading frame 49	-0.693	-0.979	0.021

564317714	Ktn1	kinectin 1	-0.687	-0.998	0.002
300794452	PTPRH	protein tyrosine phosphatase receptor type H	-0.687	-0.984	0.016
11993952	SRPX	sushi repeat containing protein X-linked	-0.677	-0.964	0.036
157818909	Zim1	zinc finger, imprinted 1	-0.665	-0.972	0.028
305682588	PDZD7	PDZ domain containing 7	-0.660	-0.962	0.038
201861690	TPK1	thiamin pyrophosphokinase 1	-0.657	-0.992	0.008
157824216	RRAS	RAS related	-0.655	-0.984	0.016
9845261	LGALS1	galectin 1	-0.655	-0.977	0.023
164565435	SYNJ2	synaptojanin 2	-0.654	-0.990	0.010
157822365	LAMC3	laminin subunit gamma 3	-0.651	-0.952	0.048
13786136	PDGFC	platelet derived growth factor C	-0.642	-0.999	0.001
300794353	FANCL	FA complementation group L	-0.639	-0.986	0.014
157786864	PHOSPHO1	phosphoethanolamine/phosphocholine phosphatase 1	-0.638	-0.976	0.024
392355027	TANGO6	transport and golgi organization 6 homolog	-0.638	-0.954	0.046
149061352	ADAM12	ADAM metallopeptidase domain 12	-0.638	-0.964	0.036
672060362	ELFN2	extracellular leucine rich repeat and fibronectin type III domain containing 2	-0.636	-0.951	0.049
187282311	ISLR	immunoglobulin superfamily containing leucine rich repeat	-0.635	-0.968	0.032
34734058	HCK	HCK proto-oncogene, Src family tyrosine kinase	-0.631	-0.999	0.001
397529557	C8orf58	chromosome 8 open reading frame 58	-0.629	-0.963	0.037
13591916	ABCC6	ATP binding cassette subfamily C member 6	-0.628	-0.962	0.038
16758622	IFT172	intraflagellar transport 172	-0.627	-0.956	0.044
157817396	MIS18BP1	MIS18 binding protein 1	-0.621	-0.971	0.029
402478640	HTRA3	HtrA serine peptidase 3	-0.608	-0.994	0.006
157819065	ADAMTS15	ADAM metallopeptidase with thrombospondin type 1 motif 15	-0.604	-0.951	0.049
672014740	MAMDC2	MAM domain containing 2	-0.603	-0.964	0.036
293347888	SRBD1	S1 RNA binding domain 1	-0.598	-0.980	0.020
564387543	UGGT2	UDP-glucose glycoprotein glucosyltransferase 2	-0.596	-0.962	0.038
6978737	CYP1B1	cytochrome P450 family 1 subfamily B member 1	-0.593	-0.992	0.008
567316103	Ac1576	uncharacterized LOC102552783	-0.587	-0.991	0.009
197927123	LYRM7	LYR motif containing 7	-0.587	-0.984	0.016
300793858	PARP14	poly(ADP-ribose) polymerase family member 14	-0.585	-0.964	0.036

564394999	CLGN	calmegin	-0.582	-0.956	0.044
16758560	WIF1	WNT inhibitory factor 1	-0.573	-0.989	0.011
148690852	FCGRT	Fc fragment of IgG receptor and transporter	-0.573	-0.979	0.021
55741859	XRCC4	X-ray repair cross complementing 4	-0.569	-0.994	0.006
19173754	TESK2	testis associated actin remodelling kinase 2	-0.567	-0.976	0.024
564390348	Klhl3	kelch-like family member 3	-0.566	-0.971	0.029
62078799	QRSL1	glutaminyl-tRNA amidotransferase subunit QRSL1	-0.566	-0.984	0.016
62078887	CLEC14A	C-type lectin domain containing 14A	-0.564	-0.968	0.032
57012436	Krt10	keratin 10	-0.562	-0.995	0.005
75832150	GALNT3	polypeptide N-acetylgalactosaminyltransferase 3	-0.557	-0.984	0.016
300798350	LRRK1	leucine rich repeat kinase 1	-0.554	-0.985	0.015
9507045	RGS5	regulator of G protein signaling 5	-0.538	-0.997	0.003
11560101	GCNT1	glucosaminyl (N-acetyl) transferase 1	-0.537	-0.979	0.021
46310239	SIDT1	SID1 transmembrane family member 1	-0.528	-0.970	0.030
157818843	EXTL1	exostosin like glycosyltransferase 1	-0.527	-0.960	0.040
19424232	CSF2RB	colony stimulating factor 2 receptor subunit beta	-0.524	-0.985	0.015
148692356	ARHGEF1	Rho guanine nucleotide exchange factor 1	-0.517	-0.988	0.012
293348472	ZFR2	zinc finger RNA binding protein 2	-0.516	-0.974	0.026
62078773	CCDC81	coiled-coil domain containing 81	-0.511	-0.991	0.009
293344916	COL6A1	collagen type VI alpha 1 chain	-0.508	-0.957	0.043
654824082	Fbxl21	F-box and leucine-rich repeat protein 21	-0.500	-0.960	0.040
58865396	FIGNL1	fidgetin like 1	-0.497	-0.959	0.041
281371499	COL5A2	collagen type V alpha 2 chain	-0.495	-0.966	0.034
157817292	TPCN2	two pore segment channel 2	-0.495	-0.969	0.031
84662732	DNASE1L1	deoxyribonuclease 1 like 1	-0.494	-0.977	0.023
157822759	PARP2	poly(ADP-ribose) polymerase 2	-0.493	-0.969	0.031
399220341	SLC2A13	solute carrier family 2 member 13	-0.492	-0.982	0.018
157819347	CDC6	cell division cycle 6	-0.492	-0.985	0.015
564399546	STARD8	StAR related lipid transfer domain containing 8	-0.484	-0.975	0.025
392333084	CC2D2A	coiled-coil and C2 domain containing 2A	-0.483	-0.960	0.040
395759219	AQP4	aquaporin 4	-0.482	-0.968	0.032
189011606	NCEH1	neutral cholesterol ester hydrolase 1	-0.476	-0.957	0.043
157816939	WASHC3	WASH complex subunit 3	-0.469	-0.983	0.017
114145710	SEC61G	SEC61 translocon subunit gamma	-0.469	-0.950	0.050

75905809	AKAP12	A-kinase anchoring protein 12	-0.466	-0.967	0.033
41056215	XRCC5	X-ray repair cross complementing 5	-0.466	-0.996	0.004
149031202	Saysd1	SAYSVFN motif domain containing 1	-0.464	-0.966	0.034
56090632	DMAC2L	distal membrane arm assembly complex 2 like	-0.463	-0.961	0.039
56605808	CENPN	centromere protein N	-0.462	-0.958	0.042
68163403	SLC46A3	solute carrier family 46 member 3	-0.458	-0.999	0.001
56090411	POLE3	DNA polymerase epsilon 3, accessory subunit	-0.456	-0.985	0.015
149067372	MTERF2	mitochondrial transcription termination factor 2	-0.455	-0.989	0.011
157817743	CDH5	cadherin 5	-0.454	-0.997	0.003
9437326	SLC4A4	solute carrier family 4 member 4	-0.453	-0.996	0.004
13027400	GUCY1A2	guanylate cyclase 1 soluble subunit alpha 2	-0.453	-0.990	0.010
158138494	PTPRC	protein tyrosine phosphatase receptor type C	-0.451	-0.995	0.005
33414515	PXK	PX domain containing serine/threonine kinase like	-0.450	-0.994	0.006
157073937	PARP9	poly(ADP-ribose) polymerase family member 9	-0.443	-0.975	0.025
16758186	SLCO1C1	solute carrier organic anion transporter family member 1C1	-0.441	-0.970	0.030
149022245	SCRN3	secernin 3	-0.437	-0.995	0.005
68534547	NUDT18	nudix hydrolase 18	-0.437	-0.971	0.029
148686921	SLC24A4	solute carrier family 24 member 4	-0.435	-0.985	0.015
61889119	TNFSF12	TNF superfamily member 12	-0.432	-0.974	0.026
157822677	LGI3	leucine rich repeat LGI family member 3	-0.431	-0.987	0.013
13928796	PXMP2	peroxisomal membrane protein 2	-0.426	-0.961	0.039
157821557	CD248	CD248 molecule	-0.426	-0.955	0.045
157823259	TMEM229A	transmembrane protein 229A	-0.424	-0.963	0.037
76443683	LOC100912042/Surf2	surfeit 2	-0.423	-0.969	0.031
300796997	ARHGAP28	Rho GTPase activating protein 28	-0.423	-0.999	0.001
210032365	HSP90B1	heat shock protein 90 beta family member 1	-0.421	-0.987	0.013
56090564	GALM	galactose mutarotase	-0.420	-0.985	0.015
564342737	DTWD1	DTW domain containing 1	-0.416	-0.984	0.016
62078539	Pagr1	Paxip1-associated glutamate-rich protein 1	-0.415	-0.962	0.038
62945312	CXCL16	C-X-C motif chemokine ligand 16	-0.414	-0.984	0.016
68342019	LRRC17	leucine rich repeat containing 17	-0.413	-0.953	0.047
13591949	GATM	glycine amidinotransferase	-0.409	-0.983	0.017

13162347	FDXR	ferredoxin reductase	-0.405	-0.968	0.032
149049048	RECQL	RecQ like helicase	-0.403	-0.968	0.032
127140886	EML6	EMAP like 6	-0.403	-0.959	0.041
195973006	EGFLAM	EGF like, fibronectin type III and laminin G domains	-0.403	-0.984	0.016
61889088	CYP2J2	cytochrome P450 family 2 subfamily J member 2	-0.401	-0.961	0.039
56605714	NDUFAF7	NADH:ubiquinone oxidoreductase complex assembly factor 7	-0.399	-0.995	0.005
187937124	TMEM126B	transmembrane protein 126B	-0.389	-0.999	0.001
58865650	LIAS	lipoic acid synthetase	-0.388	-0.963	0.037
166999225	GRM1	glutamate metabotropic receptor 1	-0.385	-0.990	0.010
149034469	GNG7	G protein subunit gamma 7	-0.381	-0.992	0.008
219282679	ZNF43	zinc finger protein 43	-0.374	-0.988	0.012
672083553	SLC14A1	solute carrier family 14 member 1 (Kidd blood group)	-0.373	-0.966	0.034
189011677	GLB1L	galactosidase beta 1 like	-0.373	-0.986	0.014
929981595	NPHP1	nephrocystin 1	-0.372	-0.968	0.032
157824002	ATG10	autophagy related 10	-0.372	-0.965	0.035
564299653	FAM169A	family with sequence similarity 169 member A	-0.372	-0.984	0.016
113061	CHRNA3	cholinergic receptor nicotinic alpha 3 subunit	-0.372	-0.958	0.042
198386343	TRPS1	transcriptional repressor GATA binding 1	-0.370	-0.974	0.026
8393861	HPCAL4	hippocalcin like 4	-0.370	-0.986	0.014
158303308	PCCA	propionyl-CoA carboxylase subunit alpha	-0.369	-0.979	0.021
564343748	CDK5RAP1	CDK5 regulatory subunit associated protein 1	-0.368	-0.955	0.045
564357619	ITGB8	integrin subunit beta 8	-0.366	-0.968	0.032
16758712	PDIA4	protein disulfide isomerase family A member 4	-0.362	-0.984	0.016
55741549	MRPL13	mitochondrial ribosomal protein L13	-0.360	-0.995	0.005
149066868	MDM1	Mdm1 nuclear protein	-0.357	-0.986	0.014
564331077	HIRIP3	HIRA interacting protein 3	-0.352	-0.991	0.009
56605656	DONSON	DNA replication fork stabilization factor DONSON	-0.350	-0.958	0.042
451172073	CHRM3	cholinergic receptor muscarinic 3	-0.349	-0.973	0.027
56119120	SNF8	SNF8 subunit of ESCRT-II	-0.343	-0.952	0.048
62078847	TSEN2	tRNA splicing endonuclease subunit 2	-0.343	-0.975	0.025
300795140	TAF1	TATA-box binding protein associated factor 1	-0.342	-0.986	0.014

77157795	MAL2	mal, T cell differentiation protein 2 (gene/pseudogene)	-0.342	-0.976	0.024
300795679	CD84	CD84 molecule	-0.338	-0.988	0.012
300794275	MFSD10	major facilitator superfamily domain containing 10	-0.330	-0.975	0.025
564309649	CCDC159	coiled-coil domain containing 159	-0.329	-0.992	0.008
564393980	ME2	malic enzyme 2	-0.327	-0.976	0.024
61557206	ZBTB16	zinc finger and BTB domain containing 16	-0.326	-0.967	0.033
149045696	Ccl27a	chemokine (C-C motif) ligand 27A	-0.325	-0.978	0.022
51036680	SLC29A3	solute carrier family 29 member 3	-0.322	-0.972	0.028
45267819	CAV2	caveolin 2	-0.321	-0.953	0.047
451172111	HINT3	histidine triad nucleotide binding protein 3	-0.321	-0.966	0.034
672063876	MGC116197 (includes others)	similar to RIKEN cDNA 1700001E04	-0.320	-0.969	0.031
300797242	SPG11	SPG11 vesicle trafficking associated, spatacsin	-0.316	-0.987	0.013
57528352	DMAC2	distal membrane arm assembly complex 2	-0.315	-0.971	0.029
281332095	RB1	RB transcriptional corepressor 1	-0.313	-0.966	0.034
157786608	MRPL55	mitochondrial ribosomal protein L55	-0.313	-0.972	0.028
564336403	EXOSC8	exosome component 8	-0.309	-0.999	0.001
149063353	IFT81	intraflagellar transport 81	-0.309	-0.991	0.009
167860097	FN3KRP	fructosamine 3 kinase related protein	-0.308	-0.973	0.027
12621108	NR1I3	nuclear receptor subfamily 1 group I member 3	-0.308	-0.990	0.010
61556910	SNX10	sorting nexin 10	-0.306	-0.961	0.039
545532952	EIF4E3	eukaryotic translation initiation factor 4E family member 3	-0.305	-0.985	0.015
568961602	VPS13C	vacuolar protein sorting 13 homolog C	-0.304	-0.979	0.021
31982028	RSU1	Ras suppressor protein 1	-0.304	-0.973	0.027
300796069	THADA	THADA armadillo repeat containing	-0.303	-0.998	0.002
198041989	PARVB	parvin beta	-0.302	-0.973	0.027
198278496	C1R	complement C1r	-0.299	-0.958	0.042
157820737	NUSAP1	nucleolar and spindle associated protein 1	-0.298	-0.972	0.028
564397303	CCDC167	coiled-coil domain containing 167	-0.297	-0.958	0.042
19173736	SCPEP1	serine carboxypeptidase 1	-0.297	-0.984	0.016
38259192	TOP2A	DNA topoisomerase II alpha	-0.297	-0.989	0.011
162287198	HSD17B4	hydroxysteroid 17-beta dehydrogenase 4	-0.297	-0.993	0.007

109491454	UTP6	UTP6 small subunit processome component	-0.296	-0.960	0.040
8393992	PMP22	peripheral myelin protein 22	-0.295	-0.963	0.037
6978888	GFRA1	GDNF family receptor alpha 1	-0.294	-0.994	0.006
167560911	SGF29	SAGA complex associated factor 29	-0.293	-0.978	0.022
149057745	NEK3	NIMA related kinase 3	-0.290	-0.964	0.036
402765953	0610009B22Rik	RIKEN cDNA 0610009B22 gene	-0.287	-0.961	0.039
149034870	RNF6	ring finger protein 6	-0.283	-0.962	0.038
21489989	KCNH8	potassium voltage-gated channel subfamily H member 8	-0.282	-0.978	0.022
157823996	ELK3	ETS transcription factor ELK3	-0.282	-0.996	0.004
564317068	CCDC149	coiled-coil domain containing 149	-0.281	-0.993	0.007
197313643	GLTP	glycolipid transfer protein	-0.280	-0.982	0.018
142349612	GLUL	glutamate-ammonia ligase	-0.279	-0.996	0.004
13928886	MAP2K1	mitogen-activated protein kinase kinase 1	-0.278	-0.976	0.024
157820147	TNFRSF10A	TNF receptor superfamily member 10a	-0.272	-0.964	0.036
197333840	CAMKMT	calmodulin-lysine N-methyltransferase	-0.270	-0.997	0.003
149022319	AGPS	alkylglycerone phosphate synthase	-0.270	-0.961	0.039
149068830	SLCO2B1	solute carrier organic anion transporter family member 2B1	-0.270	-0.998	0.002
66730535	Armcx1/LOC102554790	armadillo repeat containing, X-linked 1	-0.270	-0.965	0.035
57164113	NSDHL	NAD(P) dependent steroid dehydrogenase-like	-0.269	-0.985	0.015
576796148	MAP7D2	MAP7 domain containing 2	-0.269	-0.997	0.003
74354506	ACBD5	acyl-CoA binding domain containing 5	-0.269	-0.975	0.025
197313676	AIG1	androgen induced 1	-0.268	-1.000	0.000
392339847	CADPS2	calcium dependent secretion activator 2	-0.268	-0.964	0.036
38181552	SCG2	secretogranin II	-0.267	-0.982	0.018
564382316	HSD11B1	hydroxysteroid 11-beta dehydrogenase 1	-0.265	-0.973	0.027
149041411	SC5D	sterol-C5-desaturase	-0.264	-0.969	0.031
281427203	TMEM260	transmembrane protein 260	-0.262	-0.980	0.020
114145534	Mtap	methylthioadenosine phosphorylase	-0.258	-0.979	0.021
17865325	GLRB	glycine receptor beta	-0.256	-0.985	0.015
291190715	ITGA8	integrin subunit alpha 8	-0.251	-0.953	0.047
157819311	LRGUK	leucine rich repeats and guanylate kinase domain containing	-0.248	-0.985	0.015
6978485	ALAS2	5'-aminolevulinate synthase 2	-0.248	-0.968	0.032

392341280	NCAPG2	non-SMC condensin II complex subunit G2	-0.248	-0.958	0.042
187469267	GPRC5B	G protein-coupled receptor class C group 5 member B	-0.247	-0.971	0.029
15805026	ZFAND6	zinc finger AN1-type containing 6	-0.245	-0.994	0.006
6978631	CD4	CD4 molecule	-0.245	-0.981	0.019
56090433	GLT8D1	glycosyltransferase 8 domain containing 1	-0.243	-0.989	0.011
187937143	C2orf42	chromosome 2 open reading frame 42	-0.241	-0.998	0.002
97537309	SYNJ1	synaptojanin 1	-0.240	-0.979	0.021
158749602	TRAM1L1	translocation associated membrane protein 1 like 1	-0.237	-0.952	0.048
584277046	SLC1A3	solute carrier family 1 member 3	-0.232	-0.988	0.012
213688380	GXYLT1	glucoside xylosyltransferase 1	-0.232	-0.955	0.045
293346766	TCAF1	TRPM8 channel associated factor 1	-0.232	-0.987	0.013
9457244	RBBP9	RB binding protein 9, serine hydrolase	-0.231	-0.955	0.045
157817839	SEMA5A	semaphorin 5A	-0.230	-0.994	0.006
13994225	HSD17B10	hydroxysteroid 17-beta dehydrogenase 10	-0.229	-0.973	0.027
564397761	GCC2	GRIP and coiled-coil domain containing 2	-0.229	-0.990	0.010
58865718	HERC4	HECT and RLD domain containing E3 ubiquitin protein ligase 4	-0.228	-0.980	0.020
12248187	P2RY12	purinergic receptor P2Y12	-0.228	-0.969	0.031
149062310	BSCL2	BSCL2 lipid droplet biogenesis associated, seipin	-0.228	-0.971	0.029
672050038	NDNF	neuron derived neurotrophic factor	-0.226	-1.000	0.000
205277356	TVP23B	trans-golgi network vesicle protein 23 homolog B	-0.226	-0.995	0.005
293345175	DHX29	DExH-box helicase 29	-0.225	-0.977	0.023
25742576	NXF1	nuclear RNA export factor 1	-0.224	-0.989	0.011
672040941	ATRNL1	attractin like 1	-0.223	-0.974	0.026
78369663	SLC38A9	solute carrier family 38 member 9	-0.222	-0.990	0.010
148689145	CPNE4	copine 4	-0.222	-0.963	0.037
148695758	CAPRIN1	cell cycle associated protein 1	-0.221	-0.999	0.001
158186672	Nedd4	neural precursor cell expressed, developmentally down-regulated 4	-0.220	-0.956	0.044
157822873	FBH1	F-box DNA helicase 1	-0.219	-0.971	0.029
9506469	CD47	CD47 molecule	-0.218	-0.992	0.008
564298436	WDR11	WD repeat domain 11	-0.216	-0.993	0.007
148687519	CALN1	calneuron 1	-0.214	-0.993	0.007

8393643	KCNAB1	potassium voltage-gated channel subfamily A member regulatory beta subunit 1	-0.214	-0.998	0.002
57526927	LARS1	leucyl-tRNA synthetase 1	-0.213	-0.990	0.010
392338550	IPO11	importin 11	-0.212	-0.971	0.029
415703079	NEBL	nebulette	-0.211	-0.997	0.003
25742763	HSPA5	heat shock protein family A (Hsp70) member 5	-0.211	-0.975	0.025
18959250	PRKCD	protein kinase C delta	-0.210	-0.961	0.039
225543229	TIAM1	TIAM Rac1 associated GEF 1	-0.207	-0.968	0.032
148667192	LRTM2	leucine rich repeats and transmembrane domains 2	-0.207	-0.969	0.031
6981504	ATXN1	ataxin 1	-0.204	-0.976	0.024
148747414	GDA	guanine deaminase	-0.203	-0.970	0.030
564391231	SERPINB9	serpin family B member 9	-0.203	-0.955	0.045
142385975	RNF25	ring finger protein 25	-0.202	-0.968	0.032
55741502	ACAT2	acetyl-CoA acetyltransferase 2	-0.202	-0.975	0.025
48675867	PLPP3	phospholipid phosphatase 3	-0.201	-0.968	0.032
62078801	MEF2A	myocyte enhancer factor 2A	-0.199	-0.967	0.033
26006243	KCND2	potassium voltage-gated channel subfamily D member 2	-0.197	-0.984	0.016
11560055	KHDRBS3	KH RNA binding domain containing, signal transduction associated 3	-0.195	-0.961	0.039
564334053	SORCS1	sortilin related VPS10 domain containing receptor 1	-0.195	-0.977	0.023
564347675	AAK1	AP2 associated kinase 1	-0.195	-0.959	0.041
157818397	MFSD4A	major facilitator superfamily domain containing 4A	-0.194	-0.970	0.030
63706033	Gm5174 (includes others)	serine/threonine kinase, pseudogene 1	-0.192	-0.952	0.048
17530977	ECHS1	enoyl-CoA hydratase, short chain 1	-0.191	-0.963	0.037
157822043	PLGRKT	plasminogen receptor with a C-terminal lysine	-0.191	-0.961	0.039
25282457	CCNB1	cyclin B1	-0.191	-0.958	0.042
148696094	TUBGCP4	tubulin gamma complex associated protein 4	-0.189	-0.983	0.017
564399352	TAF9B	TATA-box binding protein associated factor 9b	-0.187	-0.996	0.004
157821901	PNMA3	PNMA family member 3	-0.186	-0.972	0.028
197209847	JAK1	Janus kinase 1	-0.185	-0.964	0.036
157817420	NRIP3	nuclear receptor interacting protein 3	-0.183	-0.972	0.028
76881830	Kcnip2	potassium voltage-gated channel interacting protein 2	-0.183	-0.962	0.038
56605704	SERINC3	serine incorporator 3	-0.182	-0.966	0.034

71037403	MYL12B	myosin light chain 12B	-0.181	-0.953	0.047
32185285	BCL2L2	BCL2 like 2	-0.181	-0.998	0.002
39930507	KCNK15	potassium two pore domain channel subfamily K member 15	-0.180	-0.994	0.006
6981672	Tpm4	tropomyosin 4	-0.178	-0.971	0.029
13569846	PARVA	parvin alpha	-0.178	-0.966	0.034
201023331	MAPK11	mitogen-activated protein kinase 11	-0.176	-0.977	0.023
157818193	TTPAL	alpha tocopherol transfer protein like	-0.176	-0.985	0.015
61557085	SPTBN1	spectrin beta, non-erythrocytic 1	-0.174	-0.980	0.020
564395567	NFATC3	nuclear factor of activated T cells 3	-0.173	-0.993	0.007
61557212	CIAO3	cytosolic iron-sulfur assembly component 3	-0.170	-0.965	0.035
396941666	Dync1i2	dynein cytoplasmic 1 intermediate chain 2	-0.169	-0.997	0.003
148668175	EDNRB	endothelin receptor type B	-0.168	-0.963	0.037
186910267	LYRM2	LYR motif containing 2	-0.167	-0.987	0.013
19705545	RAB3IL1	RAB3A interacting protein like 1	-0.166	-0.964	0.036
149046296	CREG2	cellular repressor of E1A stimulated genes 2	-0.165	-0.951	0.049
19173766	LONP1	lon peptidase 1, mitochondrial	-0.164	-0.977	0.023
40786447	CFDP1	craniofacial development protein 1	-0.160	-0.956	0.044
157819829	HACD3	3-hydroxyacyl-CoA dehydratase 3	-0.159	-0.964	0.036
52345385	PDIA6	protein disulfide isomerase family A member 6	-0.159	-0.997	0.003
209529636	PPA2	inorganic pyrophosphatase 2	-0.158	-0.992	0.008
18034785	ABCB6	ATP binding cassette subfamily B member 6 (Langereis blood group)	-0.158	-0.962	0.038
149016230	ACSL3	acyl-CoA synthetase long chain family member 3	-0.157	-0.977	0.023
18266726	PAICS	phosphoribosylaminoimidazole carboxylase and phosphoribosylaminoimidazolesuccino carboxamide synthase	-0.155	-0.987	0.013
6649914	GDF11	growth differentiation factor 11	-0.154	-0.994	0.006
158534079	CHRNA5	cholinergic receptor nicotinic alpha 5 subunit	-0.154	-0.957	0.043
71043650	SRPK1	SRSF protein kinase 1	-0.150	-0.994	0.006
113461996	COA5	cytochrome c oxidase assembly factor 5	-0.149	-0.969	0.031
50510837	KIAA1191	KIAA1191	-0.148	-0.988	0.012
109505096	NID1	nidogen 1	-0.148	-0.992	0.008
157821569	ASH2L	ASH2 like, histone lysine methyltransferase complex subunit	-0.148	-0.966	0.034
48976085	GM2A	GM2 ganglioside activator	-0.147	-0.954	0.046

71043702	TM9SF4	transmembrane 9 superfamily member 4	-0.145	-0.982	0.018
148672025	MAP3K12	mitogen-activated protein kinase kinase kinase 12	-0.144	-0.996	0.004
16758736	NLGN1	neuroligin 1	-0.139	-0.996	0.004
25742686	ELOVL6	ELOVL fatty acid elongase 6	-0.136	-0.989	0.011
157823401	PIGH	phosphatidylinositol glycan anchor biosynthesis class H	-0.135	-0.990	0.010
157823181	SKP2	S-phase kinase associated protein 2	-0.134	-0.960	0.040
564345487	RINT1	RAD50 interactor 1	-0.132	-0.970	0.030
56605798	RNF167	ring finger protein 167	-0.131	-0.966	0.034
148747541	HNRNPU	heterogeneous nuclear ribonucleoprotein U	-0.130	-0.957	0.043
158749632	DBT	dihydrolipoamide branched chain transacylase E2	-0.129	-0.958	0.042
398650648	SLC8A1	solute carrier family 8 member A1	-0.127	-0.998	0.002
37359832	SCRN1	secernin 1	-0.127	-1.000	0.000
149035673	FAF1	Fas associated factor 1	-0.126	-0.960	0.040
55926219	DDX39A	DExD-box helicase 39A	-0.126	-0.962	0.038
77415383	HSPA8	heat shock protein family A (Hsp70) member 8	-0.125	-0.991	0.009
564398462	Slc9a7	solute carrier family 9 member A7	-0.125	-0.963	0.037
149053938	SLC35B1	solute carrier family 35 member B1	-0.125	-0.959	0.041
564339312	FUBP1	far upstream element binding protein 1	-0.123	-0.982	0.018
58865700	GRWD1	glutamate rich WD repeat containing 1	-0.123	-0.972	0.028
166158339	REEP3	receptor accessory protein 3	-0.121	-0.979	0.021
158254369	CDK10	cyclin dependent kinase 10	-0.121	-0.979	0.021
74229032	TPCN1	two pore segment channel 1	-0.121	-0.951	0.049
401709975	GPR176	G protein-coupled receptor 176	-0.121	-0.954	0.046
209870013	ITSN1	intersectin 1	-0.121	-0.991	0.009
71896549	UTP14A	UTP14A small subunit processome component	-0.118	-0.989	0.011
398303839	SH3GL2	SH3 domain containing GRB2 like 2, endophilin A1	-0.117	-0.971	0.029
8393390	GABRB3	gamma-aminobutyric acid type A receptor subunit beta3	-0.115	-0.964	0.036
149059529	LOC100910558	uncharacterized LOC100910558	-0.114	-0.985	0.015
6978621	CCNG1	cyclin G1	-0.114	-0.992	0.008
404247435	YLPM1	YLP motif containing 1	-0.113	-0.990	0.010
148673922	HSPH1	heat shock protein family H (Hsp110) member 1	-0.110	-0.951	0.049
157786602	NHP2	NHP2 ribonucleoprotein	-0.110	-0.972	0.028

57164133	NDUFC2	NADH:ubiquinone oxidoreductase subunit C2	-0.108	-0.971	0.029
274326692	UQCC3	ubiquinol-cytochrome c reductase complex assembly factor 3	-0.106	-0.997	0.003
672065125	ADAM23	ADAM metallopeptidase domain 23	-0.104	-0.957	0.043
58865936	SIKE1	suppressor of IKBKE 1	-0.100	-0.955	0.045
157819175	Gpr165	G protein-coupled receptor 165	-0.099	-0.980	0.020
403377905	SRGAP2	SLIT-ROBO Rho GTPase activating protein 2	-0.098	-0.961	0.039
224967068	PLCB1	phospholipase C beta 1	-0.098	-0.976	0.024
41152510	PLPPR1	phospholipid phosphatase related 1	-0.096	-0.976	0.024
154800420	GNL3L	G protein nucleolar 3 like	-0.093	-0.997	0.003
40363268	WASHC2A/WASHC2C	WASH complex subunit 2A	-0.092	-0.982	0.018
274326531	HSF1	heat shock transcription factor 1	-0.090	-0.976	0.024
50054266	NLN	neurolysin	-0.082	-0.963	0.037
149060735	SENP5	SUMO specific peptidase 5	-0.077	-0.957	0.043
34536836	EHD3	EH domain containing 3	-0.071	-0.983	0.017
564375060	SLC39A11	solute carrier family 39 member 11	-0.070	-0.988	0.012
672055279	PREPL	prolyl endopeptidase like	-0.068	-0.952	0.048
148747528	PTK2B	protein tyrosine kinase 2 beta	-0.066	-0.993	0.007
255918181	NUS1	NUS1 dehydrodolichyl diphosphate synthase subunit	-0.062	-0.992	0.008
20302113	STIP1	stress induced phosphoprotein 1	-0.060	-1.000	0.000
149049470	TPI1	triosephosphate isomerase 1	-0.057	-0.973	0.027
162287208	FADS1	fatty acid desaturase 1	-0.050	-0.975	0.025
16758168	FGF13	fibroblast growth factor 13	-0.049	-0.955	0.045
189027133	TTC30B	tetratricopeptide repeat domain 30B	-0.044	-0.980	0.020
20302061	ATP5PO	ATP synthase peripheral stalk subunit OSCP	-0.040	-0.955	0.045
62079109	LANCL2	LanC like 2	-0.034	-0.973	0.027
157818471	PPM1L	protein phosphatase, Mg2+/Mn2+ dependent 1L	-0.032	-0.990	0.010
76443681	USP11	ubiquitin specific peptidase 11	0.053	0.965	0.035
300798222	SIAH3	siah E3 ubiquitin protein ligase family member 3	0.054	0.963	0.037
58865796	PTDSS1	phosphatidylserine synthase 1	0.057	0.953	0.047
157816927	GMEB1	glucocorticoid modulatory element binding protein 1	0.063	0.985	0.015
157822779	DNAJC11	DnaJ heat shock protein family (Hsp40) member C11	0.066	0.990	0.010
25453374	PEX14	peroxisomal biogenesis factor 14	0.067	0.961	0.039
62089200	ZDHHC9	zinc finger DHHC-type palmitoyltransferase 9	0.068	0.966	0.034

25742568	DPYSL3	dihydropyrimidinase like 3	0.069	0.973	0.027
564326269	RPL28	ribosomal protein L28	0.075	0.969	0.031
157817783	SNX18	sorting nexin 18	0.079	0.952	0.048
68341979	PLEKHO1	pleckstrin homology domain containing O1	0.080	0.979	0.021
60360532	OSBPL6	oxysterol binding protein like 6	0.083	0.997	0.003
672074758	NCSTN	nicastrin	0.083	0.960	0.040
157817696	PIN1	peptidylprolyl cis/trans isomerase, NIMA-interacting 1	0.083	0.953	0.047
148747227	SV2A	synaptic vesicle glycoprotein 2A	0.084	0.969	0.031
6978449	ADD2	adducin 2	0.085	0.988	0.012
290560659	ZNF609	zinc finger protein 609	0.090	0.980	0.020
564363529	NCAM1	neural cell adhesion molecule 1	0.091	0.997	0.003
149038024	RIPOR1	RHO family interacting cell polarization regulator 1	0.091	0.982	0.018
14389301	SMPD2	sphingomyelin phosphodiesterase 2	0.096	0.986	0.014
119618921	RAN	RAN, member RAS oncogene family	0.098	0.970	0.030
970596961	MAPK10	mitogen-activated protein kinase 10	0.099	0.961	0.039
291084664	TRAPPC10	trafficking protein particle complex 10	0.099	0.963	0.037
166064004	GTF3A	general transcription factor IIIA	0.101	0.983	0.017
58865712	RRP1	ribosomal RNA processing 1	0.104	0.957	0.043
46391106	ARL10	ADP ribosylation factor like GTPase 10	0.105	0.985	0.015
564309116	RTL6	retrotransposon Gag like 6	0.115	0.959	0.041
564360651	LRRC14	leucine rich repeat containing 14	0.115	0.971	0.029
51948396	TUSC3	tumor suppressor candidate 3	0.116	0.965	0.035
274321371	CRLF3	cytokine receptor like factor 3	0.118	0.960	0.040
18959272	KCNQ2	potassium voltage-gated channel subfamily Q member 2	0.119	0.997	0.003
293344794	FAM160B1	family with sequence similarity 160 member B1	0.121	0.985	0.015
109510888	FAM155B	family with sequence similarity 155 member B	0.121	0.973	0.027
58865648	SAMD8	sterile alpha motif domain containing 8	0.122	0.975	0.025
157818629	HEYL	hes related family bHLH transcription factor with YRPW motif like	0.126	0.963	0.037
58866022	MGAT4A	alpha-1,3-mannosyl-glycoprotein 4-beta-N-acetylglucosaminyltransferase A	0.127	0.997	0.003
672068548	SUPT6H	SPT6 homolog, histone chaperone and transcription elongation factor	0.127	0.998	0.002
300797157	TBC1D8	TBC1 domain family member 8	0.131	0.969	0.031

60359978	KIF3C	kinesin family member 3C	0.131	0.964	0.036
157824037	USP4	ubiquitin specific peptidase 4	0.131	0.986	0.014
564396315	TAF5L	TATA-box binding protein associated factor 5 like	0.132	0.964	0.036
564398139	FYN	FYN proto-oncogene, Src family tyrosine kinase	0.137	0.974	0.026
20301952	SLC2A1	solute carrier family 2 member 1	0.142	0.959	0.041
74139306	TMED9	transmembrane p24 trafficking protein 9	0.143	0.998	0.002
13928926	MYBBP1A	MYB binding protein 1a	0.146	0.962	0.038
40786455	BPGM	bisphosphoglycerate mutase	0.150	0.963	0.037
564311031	CLPP	caseinolytic mitochondrial matrix peptidase proteolytic subunit	0.150	0.976	0.024
300253233	LEMD3	LEM domain containing 3	0.154	0.993	0.007
209863130	SEMA3F	semaphorin 3F	0.155	0.995	0.005
50510427	IP6K1	inositol hexakisphosphate kinase 1	0.156	0.999	0.001
564393951	MBD1	methyl-CpG binding domain protein 1	0.157	0.954	0.046
77917548	DUS3L	dihydrouridine synthase 3 like	0.158	0.994	0.006
672043520	PI4KB	phosphatidylinositol 4-kinase beta	0.159	0.979	0.021
76559935	TUT1	terminal uridylyl transferase 1, U6 snRNA-specific	0.160	0.961	0.039
404312665	DKK3	dickkopf WNT signaling pathway inhibitor 3	0.160	0.955	0.045
213688411	LPCAT1	lysophosphatidylcholine acyltransferase 1	0.162	0.977	0.023
149033480	Zfp956	zinc finger protein 956	0.163	0.970	0.030
672072928	CUX2	cut like homeobox 2	0.164	0.965	0.035
672067460	RGD1560464	similar to hypothetical protein FLJ38426	0.164	0.991	0.009
60359854	POLDIP3	DNA polymerase delta interacting protein 3	0.164	0.964	0.036
300798499	AFF3	AF4/FMR2 family member 3	0.164	0.980	0.020
564340867	MMADHC	metabolism of cobalamin associated D	0.165	0.998	0.002
76559929	NOC2L	NOC2 like nucleolar associated transcriptional repressor	0.165	0.986	0.014
403225023	BRAP	BRCA1 associated protein	0.166	0.982	0.018
55926133	RFC2	replication factor C subunit 2	0.168	0.956	0.044
157818643	KCTD3	potassium channel tetramerization domain containing 3	0.170	0.992	0.008
149067744	Znf48	zinc finger protein 48	0.170	0.963	0.037
157823165	DNAJB1	DnaJ heat shock protein family (Hsp40) member B1	0.171	0.980	0.020
157822067	BAP1	BRCA1 associated protein 1	0.172	0.961	0.039

56605790	HCFC2	host cell factor C2	0.173	0.958	0.042
149044006	TEDC1	tubulin epsilon and delta complex 1	0.179	0.971	0.029
28972780	TLE3	TLE family member 3, transcriptional corepressor	0.180	0.967	0.033
392333209	DLG5	discs large MAGUK scaffold protein 5	0.180	0.981	0.019
62079005	SLAIN1	SLAIN motif family member 1	0.180	0.964	0.036
405113028	TAF3	TATA-box binding protein associated factor 3	0.181	0.976	0.024
762006019	FAM8A1	family with sequence similarity 8 member A1	0.185	0.996	0.004
564384443	EIF4ENIF1	eukaryotic translation initiation factor 4E nuclear import factor 1	0.187	0.977	0.023
66730335	SUMO3	small ubiquitin like modifier 3	0.188	0.988	0.012
300796253	ALDH1L2	aldehyde dehydrogenase 1 family member L2	0.190	0.983	0.017
157823447	MFHAS1	malignant fibrous histiocytoma amplified sequence 1	0.191	0.973	0.027
672085227	USP10	ubiquitin specific peptidase 10	0.191	0.975	0.025
148683335	SLC25A44	solute carrier family 25 member 44	0.191	0.994	0.006
197313795	MTX1	metaxin 1	0.192	0.953	0.047
157823197	NDUFB7	NADH:ubiquinone oxidoreductase subunit B7	0.194	0.995	0.005
157820969	SBNO2	strawberry notch homolog 2	0.197	1.000	0.000
157820401	ABHD2	abhydrolase domain containing 2, acylglycerol lipase	0.197	0.999	0.001
57164107	NIPSNAP3A	nipsnap homolog 3A	0.199	0.999	0.001
81295375	SLC35B2	solute carrier family 35 member B2	0.200	0.965	0.035
77797839	UBXN1	UBX domain protein 1	0.202	0.979	0.021
219277692	NDUFB2	NADH:ubiquinone oxidoreductase subunit B2	0.203	0.965	0.035
564384353	SH3BP2	SH3 domain binding protein 2	0.204	0.960	0.040
187469679	LDB1	LIM domain binding 1	0.205	0.967	0.033
157817674	ATP5MF-PTCD1	ATP5MF-PTCD1 readthrough	0.207	0.982	0.018
61556748	TSPYL1	TSPY like 1	0.211	0.965	0.035
310616720	DHX37	DEAH-box helicase 37	0.212	0.985	0.015
67078512	SNX15	sorting nexin 15	0.215	0.959	0.041
84781638	KLHL25	kelch like family member 25	0.215	0.981	0.019
213688373	GADD45GIP1	GADD45G interacting protein 1	0.218	0.993	0.007
765099233	LMNB2	lamin B2	0.219	0.988	0.012
564355517	CMPK2	cytidine/uridine monophosphate kinase 2	0.222	0.956	0.044
288541353	CMTM4	CKLF like MARVEL transmembrane domain containing 4	0.222	0.987	0.013

117940043	MED22	mediator complex subunit 22	0.225	0.957	0.043
70794793	MAP2K7	mitogen-activated protein kinase kinase 7	0.228	0.966	0.034
6978483	ALAD	aminolevulinate dehydratase	0.231	0.956	0.044
58865962	RNF41	ring finger protein 41	0.231	0.967	0.033
148670791	ZFYVE1	zinc finger FYVE-type containing 1	0.232	0.963	0.037
62664711	DIPK1C	divergent protein kinase domain 1C	0.235	0.964	0.036
70794766	MRPS25	mitochondrial ribosomal protein S25	0.235	0.976	0.024
564340133	GTF3C4	general transcription factor IIIC subunit 4	0.236	0.964	0.036
37360264	TRMT6	tRNA methyltransferase 6	0.236	0.990	0.010
564359486	TBC1D30	TBC1 domain family member 30	0.237	0.980	0.020
19173746	STK17B	serine/threonine kinase 17b	0.237	0.963	0.037
62078913	OAF	out at first homolog	0.242	0.957	0.043
31415868	MAFB	MAF bZIP transcription factor B	0.246	0.989	0.011
209529662	LOC100911166/Rpusd2	RNA pseudouridine synthase domain containing 2	0.252	0.975	0.025
291042683	DCAF5	DDB1 and CUL4 associated factor 5	0.252	0.967	0.033
157823719	TRAIP	TRAF interacting protein	0.252	0.960	0.040
209954792	PDCD2	programmed cell death 2	0.254	0.992	0.008
57164019	B4GALT3	beta-1,4-galactosyltransferase 3	0.256	0.970	0.030
157821283	C19orf47	chromosome 19 open reading frame 47	0.257	0.992	0.008
119388826	TFPT	TCF3 fusion partner	0.258	0.951	0.049
564367958	SEMA4C	semaphorin 4C	0.258	0.996	0.004
281427178	CEP76	centrosomal protein 76	0.262	0.953	0.047
197382256	PHF12	PHD finger protein 12	0.263	0.994	0.006
40254721	AMIGO2	adhesion molecule with Ig like domain 2	0.264	0.953	0.047
148696370	PANK2	pantothenate kinase 2	0.270	0.970	0.030
157823125	MRPS30	mitochondrial ribosomal protein S30	0.276	0.972	0.028
402794103	ATG101	autophagy related 101	0.279	0.955	0.045
149052738	RGD1561277	RGD1561277	0.285	0.975	0.025
62078733	MAK16	MAK16 homolog	0.287	0.971	0.029
672020915	VCPKMT	valosin containing protein lysine methyltransferase	0.288	0.952	0.048
143359181	SLC66A2	solute carrier family 66 member 2	0.288	0.961	0.039
51948506	AK8	adenylate kinase 8	0.290	0.967	0.033
76559919	N4BP3	NEDD4 binding protein 3	0.292	0.994	0.006
166795897	PIMREG	PICALM interacting mitotic regulator	0.293	0.989	0.011
157818273	CDC42EP4	CDC42 effector protein 4	0.297	0.961	0.039
68163385	GPATCH4	G-patch domain containing 4	0.297	0.953	0.047
157821997	MED28	mediator complex subunit 28	0.297	0.982	0.018
157822519	CBLN4	cerebellin 4 precursor	0.299	0.971	0.029

157822367	PUS3	pseudouridine synthase 3	0.301	0.958	0.042
157819365	TBC1D25	TBC1 domain family member 25	0.302	0.989	0.011
157819315	OSBPL11	oxysterol binding protein like 11	0.306	0.959	0.041
51491900	TOR1A	torsin family 1 member A	0.308	0.976	0.024
56605776	TAF11	TATA-box binding protein associated factor 11	0.309	0.982	0.018
349501022	2410002F23Rik	RIKEN cDNA 2410002F23 gene	0.309	0.972	0.028
300793780	ZNF251	zinc finger protein 251	0.321	0.985	0.015
149051028	RNF144A	ring finger protein 144A	0.322	0.970	0.030
41386747	ZC3H18	zinc finger CCCH-type containing 18	0.322	0.981	0.019
672030183	H2AC12	H2A clustered histone 12	0.323	0.985	0.015
157820119	LRRTM1	leucine rich repeat transmembrane neuronal 1	0.328	0.968	0.032
68163537	NXPE4	neurexophilin and PC-esterase domain family member 4	0.329	0.987	0.013
564333920	PPRC1	PPARG related coactivator 1	0.333	0.951	0.049
157824124	NUAK1	NUAK family kinase 1	0.335	0.971	0.029
564350836	MELK	maternal embryonic leucine zipper kinase	0.341	0.999	0.001
30017415	ITPKC	inositol-trisphosphate 3-kinase C	0.343	0.980	0.020
564365330	CDC25A	cell division cycle 25A	0.348	0.989	0.011
57528321	RIOK2	RIO kinase 2	0.360	0.986	0.014
392342123	ALS2CL	ALS2 C-terminal like	0.360	0.986	0.014
157822027	CSRNP2	cysteine and serine rich nuclear protein 2	0.361	0.990	0.010
300797828	KAT14	lysine acetyltransferase 14	0.365	0.957	0.043
213972545	MXD1	MAX dimerization protein 1	0.378	0.984	0.016
14388593	SPATA2	spermatogenesis associated 2	0.385	0.969	0.031
157821403	RASSF7	Ras association domain family member 7	0.399	0.987	0.013
226371633	CABLES1	Cdk5 and Abl enzyme substrate 1	0.409	0.977	0.023
66730347	PTPRCAP	protein tyrosine phosphatase receptor type C associated protein	0.415	0.987	0.013
293348214	CCDC88C	coiled-coil domain containing 88C	0.421	0.959	0.041
38454286	STIMATE-MUSTN1	STIMATE-MUSTN1 readthrough	0.425	0.965	0.035
293348129	DACT1	dishevelled binding antagonist of beta catenin 1	0.433	0.961	0.039
157823891	ING2	inhibitor of growth family member 2	0.439	0.955	0.045
893846521	MARCHF11	membrane associated ring-CH-type finger 11	0.445	0.973	0.027
3676248	Prim1	DNA primase subunit 1	0.450	0.992	0.008
255708448	KATNA1	katanin catalytic subunit A1	0.466	0.974	0.026

148706598	PKDCC	protein kinase domain containing, cytoplasmic	0.469	0.977	0.023
51948492	NUDT19	nudix hydrolase 19	0.470	0.953	0.047
564347830	ZXDC	ZXD family zinc finger C	0.490	0.983	0.017
212549645	KIF18A	kinesin family member 18A	0.505	0.952	0.048
19424300	GCHFR	GTP cyclohydrolase I feedback regulator	0.519	0.987	0.013
89145411	SULT2B1	sulfotransferase family 2B member 1	0.524	0.962	0.038
404434380	ZNF133	zinc finger protein 133	0.530	0.991	0.009
148687591	TMEM132D	transmembrane protein 132D	0.550	0.986	0.014
219879771	PGAP3	post-GPI attachment to proteins phospholipase 3	0.560	0.977	0.023
149023178	CEP152	centrosomal protein 152	0.572	0.955	0.045
148692940	WAPL	WAPL cohesin release factor	0.573	0.969	0.031
148664537	Gm10269	ribosomal protein L35 pseudogene	0.582	0.985	0.015
148705473	FAM53A	family with sequence similarity 53 member A	0.585	0.962	0.038
13994119	KHK	ketohexokinase	0.612	0.968	0.032
22122541	LRRC3B	leucine rich repeat containing 3B	0.615	1.000	0.000
157822359	PELI2	pellino E3 ubiquitin protein ligase family member 2	0.620	0.990	0.010
157820433	CPEB1	cytoplasmic polyadenylation element binding protein 1	0.624	0.953	0.047
197386066	ZNF784	zinc finger protein 784	0.633	0.983	0.017
157823803	DOK3	docking protein 3	0.639	0.975	0.025
149025186	RPS6KL1	ribosomal protein S6 kinase like 1	0.661	0.998	0.002
149016574	ZNF324	zinc finger protein 324	0.689	0.964	0.036
392354293	Hmgb3	high mobility group box 3	0.709	0.984	0.016
80861398	CRY1	cryptochrome circadian regulator 1	0.720	0.980	0.020
38454200	CHDH	choline dehydrogenase	0.734	0.988	0.012
293339965	RAB11FIP3	RAB11 family interacting protein 3	0.750	0.995	0.005
672086719	FAM184A	family with sequence similarity 184 member A	0.836	0.972	0.028
149025439	DICER1	dicer 1, ribonuclease III	0.846	0.952	0.048
568959785	PRDM10	PR/SET domain 10	0.865	0.953	0.047
157821687	NEURL2	neuralized E3 ubiquitin protein ligase 2	0.966	0.958	0.042
209571573	ZNF707	zinc finger protein 707	0.966	0.974	0.026
148693657	DDX6	DEAD-box helicase 6	0.969	0.954	0.046
82654234	LILRA6	leukocyte immunoglobulin like receptor A6	1.000	0.965	0.035
149067796	TMEM219	transmembrane protein 219	1.064	0.969	0.031
19424314	KCNE2	potassium voltage-gated channel subfamily E regulatory subunit 2	1.066	0.978	0.022

188536090	FAM241B	family with sequence similarity 241 member B	1.127	0.951	0.049
62078917	PAQR5	progestin and adipoQ receptor family member 5	1.181	0.986	0.014
6978493	ALOX5	arachidonate 5-lipoxygenase	1.193	0.988	0.012
157787081	WNT1	Wnt family member 1	1.222	0.986	0.014
300793935	GSX1	GS homeobox 1	1.225	0.963	0.037
672068318	PITPNM3	PITPNM family member 3	1.283	0.955	0.045
569012000	KLF8	Kruppel like factor 8	1.406	0.979	0.021
157817264	ANKRD23	ankyrin repeat domain 23	1.505	0.975	0.025
575403049	ERBIN	erbb2 interacting protein	1.596	0.951	0.049
157818463	Zfp93	zinc finger protein 93	1.597	0.961	0.039
672013187	DMWD	DM1 locus, WD repeat containing	1.605	0.989	0.011
157819799	IQCH	IQ motif containing H	1.716	0.997	0.003
576080555	GAPDH	glyceraldehyde-3-phosphate dehydrogenase	1.720	0.991	0.009
149032888	LOC100910237	uncharacterized LOC100910237	1.751	0.969	0.031
157820135	CHRDL2	chordin like 2	1.762	0.965	0.035
392342449	PRSS56	serine protease 56	1.848	0.987	0.013
149042883	LOC100365365	rCG32328-like	1.861	0.969	0.031
189181736	LAD1	ladinin 1	1.874	0.986	0.014
16758254	CNGA1	cyclic nucleotide gated channel subunit alpha 1	1.874	0.987	0.013
568979594	SYT16	synaptotagmin 16	2.059	0.963	0.037
672070295	BAHCC1	BAH domain and coiled-coil containing 1	2.083	0.999	0.001
28972866	CSMD3	CUB and Sushi multiple domains 3	2.140	0.976	0.024
293352381	PAN3	poly(A) specific ribonuclease subunit PAN3	2.239	0.996	0.004
157818163	POF1B	POF1B actin binding protein	2.392	0.972	0.028
564347547	LOC103690120	probable N-acetyltransferase CML1	3.030	0.967	0.033
28174920	RPL17	ribosomal protein L17	3.389	0.950	0.050

Supplementary Table S15. The list of genes that are differentially expressed in the offspring hippocampus in response to prenatal BPA exposure that exhibited the changes in the expression levels correlated with the number of neurites (≥ 100 μm) of primary hippocampal cells at DIV7. The transcriptome profiling data of DEGs in male and female rat offspring prenatally exposed to BPA (n = 6, male pups n = 3 and female pups n = 3, from independent litters) or the vehicle control (n = 6, male pups n = 3 and female pups n = 3, from independent litters) were obtained and used for the PTM analyses to identify DEGs that exhibited the changes in the expression levels correlated with the number of neurites (≥ 100 μm) of primary hippocampal cells at DIV7.

ID	Symbol	Entrez Gene Name	log2(FC)	R values	P-values
564316241	CEP170	centrosomal protein 170	-8.600	-0.958	0.042
293347435	PTPRD	protein tyrosine phosphatase receptor type D	-7.731	-0.995	0.005
564307173	HEATR5A	HEAT repeat containing 5A	-6.735	-0.972	0.028
564310188	IGDCC4	immunoglobulin superfamily DCC subclass member 4	-6.728	-0.995	0.005
564314389	DZIP3	DAZ interacting zinc finger protein 3	-6.700	-0.979	0.021
672031167	C19orf57	chromosome 19 open reading frame 57	-6.476	-0.985	0.015
149020633	TAF1D	TATA-box binding protein associated factor, RNA polymerase I subunit D	-6.476	-0.975	0.025
149052470	ZNF454	zinc finger protein 454	-5.700	-0.982	0.018
157818475	SMIM22	small integral membrane protein 22	-5.615	-0.988	0.012
754169051	EPPIN	epididymal peptidase inhibitor	-5.066	-0.976	0.024
197384778	Snorc	secondary ossification center associated regulator of chondrocyte maturation	-5.044	-0.990	0.010
564323305	LOC681300	similar to CXXC finger 5	-4.672	-0.973	0.027
300798104	IFNLR1	interferon lambda receptor 1	-4.248	-0.991	0.009
564298047	GDPD5	glycerophosphodiester phosphodiesterase domain containing 5	-4.163	-0.977	0.023
114145748	LOC680227	LRRGT00193	-3.907	-0.995	0.005
194474016	SLC30A8	solute carrier family 30 member 8	-3.807	-0.993	0.007
8392926	ASGR2	asialoglycoprotein receptor 2	-3.700	-0.996	0.004
72255533	ANXA8/ANXA8L1	annexin A8 like 1	-3.644	-0.964	0.036
157823827	S1PR4	sphingosine-1-phosphate receptor 4	-3.392	-0.961	0.039
148675704	TBX15	T-box transcription factor 15	-3.268	-0.962	0.038
564324736	L3MBTL3	L3MBTL histone methyl-lysine binding protein 3	-3.262	-0.966	0.034
564375434	CEP295NL	CEP295 N-terminal like	-3.248	-0.991	0.009
56788780	KRT19	keratin 19	-3.248	-0.964	0.036

62078965	SLC47A1	solute carrier family 47 member 1	-3.135	-0.951	0.049
12408310	N5	DNA binding protein N5	-3.000	-0.988	0.012
20302091	PLB1	phospholipase B1	-2.907	-0.993	0.007
157821903	Slc7a15	solute carrier family 7 (cationic amino acid transporter, y+ system), member 15	-2.907	-0.993	0.007
564355126	ADGRF3	adhesion G protein-coupled receptor F3	-2.907	-0.969	0.031
156071424	Vom2r18 (includes others)	vomeronasal 2 receptor, 18	-2.700	-0.985	0.015
51948496	SLC22A18	solute carrier family 22 member 18	-2.687	-0.960	0.040
162417984	Vom2r12 (includes others)	vomeronasal 2 receptor, 80	-2.459	-0.980	0.020
62079023	ADTRP	androgen dependent TFPI regulating protein	-2.246	-0.988	0.012
300798413	FSD2	fibronectin type III and SPRY domain containing 2	-2.170	-0.988	0.012
66730349	SPIB	Spi-B transcription factor	-2.170	-0.973	0.027
25742828	SCN7A	sodium voltage-gated channel alpha subunit 7	-2.129	-0.980	0.020
148669850	GFRA1	GDNF family receptor alpha 1	-2.118	-0.959	0.041
300797305	TMEM45A	transmembrane protein 45A	-2.093	-0.953	0.047
148672128	SMAGP	small cell adhesion glycoprotein	-2.077	-0.954	0.046
157820583	ANKRD34C	ankyrin repeat domain 34C	-1.939	-0.976	0.024
117647214	EDN3	endothelin 3	-1.913	-0.958	0.042
157823875	EPS8L1	EPS8 like 1	-1.907	-0.987	0.013
11067389	BMP15	bone morphogenetic protein 15	-1.858	-0.951	0.049
672029702	TUT7	terminal uridylyl transferase 7	-1.854	-0.986	0.014
297374767	TPSAB1/TPSB2	tryptase alpha/beta 1	-1.834	-0.969	0.031
19424240	PCSK4	proprotein convertase subtilisin/kexin type 4	-1.740	-1.000	0.000
480306394	Mcpt4	mast cell protease 4	-1.739	-0.987	0.013
153792385	Vom2r34	vomeronasal 2 receptor, 34	-1.648	-0.958	0.042
295391913	LOC100366054	Da1-10-like	-1.590	-0.958	0.042
117647198	CFD	complement factor D	-1.585	-0.975	0.025
209870037	INSRR	insulin receptor related receptor	-1.585	-0.994	0.006
62079089	MALL	mal, T cell differentiation protein like	-1.585	-0.972	0.028
194473618	SCX	scleraxis bHLH transcription factor	-1.585	-0.980	0.020
13929066	CPZ	carboxypeptidase Z	-1.549	-0.996	0.004
158186711	F13A1	coagulation factor XIII A chain	-1.510	-0.984	0.016
13929126	GALNT5	polypeptide N-acetylgalactosaminyltransferase 5	-1.478	-0.996	0.004
18677739	CDKN2B	cyclin dependent kinase inhibitor 2B	-1.454	-0.991	0.009
81295349	SLC52A3	solute carrier family 52 member 3	-1.453	-0.976	0.024

6981148	LEP	leptin	-1.436	-0.993	0.007
283806636	ZNF831	zinc finger protein 831	-1.433	-0.982	0.018
19924087	Akr1c14	aldo-keto reductase family 1, member C14	-1.406	-0.968	0.032
564314671	VPS8	VPS8 subunit of CORVET complex	-1.386	-0.953	0.047
149065466	ARHGEF5	Rho guanine nucleotide exchange factor 5	-1.382	-0.980	0.020
48675870	PPP1R3B	protein phosphatase 1 regulatory subunit 3B	-1.379	-0.984	0.016
157786780	MELTF	melanotransferrin	-1.333	-0.954	0.046
29789044	SNAI2	snail family transcriptional repressor 2	-1.328	-0.967	0.033
157823345	LRR1	leucine rich repeat protein 1	-1.322	-0.996	0.004
66730461	Clec2d (includes others)	C-type lectin domain family 2, member D	-1.282	-0.960	0.040
148694035	SENP8	SUMO peptidase family member, NEDD8 specific	-1.276	-0.958	0.042
71043878	PROCR	protein C receptor	-1.270	-0.959	0.041
62656582	KIAA0100	KIAA0100	-1.266	-0.954	0.046
187281975	DENND1C	DENN domain containing 1C	-1.216	-0.997	0.003
56119141	BTK	Bruton tyrosine kinase	-1.205	-0.996	0.004
58865664	SH2D4A	SH2 domain containing 4A	-1.204	-0.985	0.015
155369293	AEBP1	AE binding protein 1	-1.198	-0.990	0.010
564300462	DCHS2	dachsous cadherin-related 2	-1.190	-0.981	0.019
392334475	Myb	MYB proto-oncogene, transcription factor	-1.175	-0.978	0.022
71795615	UPP1	uridine phosphorylase 1	-1.167	-0.996	0.004
157823801	SLC50A1	solute carrier family 50 member 1	-1.136	-0.968	0.032
125347412	FAM72A	family with sequence similarity 72 member A	-1.106	-0.971	0.029
300798739	MYO3B	myosin IIIB	-1.100	-0.952	0.048
149053909	COL1A1	collagen type I alpha 1 chain	-1.092	-0.977	0.023
197385083	C1orf194	chromosome 1 open reading frame 194	-1.090	-0.984	0.016
9910378	CDC42SE2	CDC42 small effector 2	-1.069	-0.993	0.007
197384727	Smco4	single-pass membrane protein with coiled-coil domains 4	-1.065	-0.998	0.002
197386139	SSC5D	scavenger receptor cysteine rich family member with 5 domains	-1.060	-0.953	0.047
297206838	ARNTL2	aryl hydrocarbon receptor nuclear translocator like 2	-1.020	-0.952	0.048
58865898	LIMS2	LIM zinc finger domain containing 2	-1.018	-0.964	0.036
300797728	MGST3	microsomal glutathione S-transferase 3	-1.011	-0.986	0.014
569009290	TENM1	teneurin transmembrane protein 1	-0.985	-0.964	0.036

300795496	LAYN	layilin	-0.983	-0.975	0.025
20302089	GABRR3	gamma-aminobutyric acid type A receptor subunit rho3 (gene/pseudogene)	-0.972	-0.973	0.027
157819493	Igbp1b	immunoglobulin (CD79A) binding protein 1b	-0.966	-0.957	0.043
564392197	LOC684327	similar to inter-alpha (globulin) inhibitor H5	-0.954	-0.961	0.039
157818275	KCNG4	potassium voltage-gated channel modifier subfamily G member 4	-0.952	-0.982	0.018
148680846	HIC1	HIC ZBTB transcriptional repressor 1	-0.940	-0.964	0.036
157823079	RBKS	ribokinase	-0.939	-0.977	0.023
198442873	CDC14A	cell division cycle 14A	-0.936	-0.962	0.038
6981176	MAK	male germ cell associated kinase	-0.931	-0.950	0.050
62945330	SLC8B1	solute carrier family 8 member B1	-0.929	-0.966	0.034
157819487	TACO1	translational activator of cytochrome c oxidase I	-0.917	-0.981	0.019
564318923	WDR17	WD repeat domain 17	-0.904	-0.966	0.034
13540656	EMP3	epithelial membrane protein 3	-0.883	-0.966	0.034
149031998	ACVRL1	activin A receptor like type 1	-0.852	-0.970	0.030
201861483	LOC102548396 (includes others)	zinc finger protein 951	-0.848	-0.972	0.028
392334002	CCDC3	coiled-coil domain containing 3	-0.833	-0.965	0.035
564366187	LOC100361039 (includes others)	similar to nidogen 2	-0.833	-0.956	0.044
56605720	GADD45B	growth arrest and DNA damage inducible beta	-0.828	-0.966	0.034
157817065	KCNK16	potassium two pore domain channel subfamily K member 16	-0.807	-0.996	0.004
564396113	ZCCHC14	zinc finger CCHC-type containing 14	-0.797	-0.982	0.018
564345556	CROT	carnitine O-octanoyltransferase	-0.790	-0.964	0.036
300798165	ZBTB40	zinc finger and BTB domain containing 40	-0.788	-0.964	0.036
942523340	CAPRIN2	caprin family member 2	-0.774	-0.959	0.041
392352101	LRCH3	leucine rich repeats and calponin homology domain containing 3	-0.773	-0.983	0.017
13591914	ANPEP	alanyl aminopeptidase, membrane	-0.757	-0.991	0.009
564312886	DNAH2	dynein axonemal heavy chain 2	-0.740	-0.959	0.041
157786850	TUBD1	tubulin delta 1	-0.740	-0.995	0.005
68163370	CARNMT1	carnosine N-methyltransferase 1	-0.724	-0.973	0.027
208973286	RBM46	RNA binding motif protein 46	-0.724	-1.000	0.000
402534539	ECRG4	ECRG4 augurin precursor	-0.723	-0.955	0.045
16758232	PLCB2	phospholipase C beta 2	-0.721	-0.970	0.030
149047075	Spaca6	sperm acrosome associated 6	-0.715	-0.986	0.014

564378828	TFR2	transferrin receptor 2	-0.707	-0.994	0.006
55742713	ECM1	extracellular matrix protein 1	-0.699	-0.996	0.004
157816997	BDH2	3-hydroxybutyrate dehydrogenase 2	-0.698	-0.982	0.018
281332190	APBB1IP	amyloid beta precursor protein binding family B member 1 interacting protein	-0.696	-0.976	0.024
148671621	VIP	vasoactive intestinal peptide	-0.696	-0.957	0.043
401461786	CP	ceruloplasmin	-0.695	-0.975	0.025
564393107	STING1	stimulator of interferon response cGAMP interactor 1	-0.693	-0.952	0.048
113206040	LRRC34	leucine rich repeat containing 34	-0.690	-0.970	0.030
564317714	Ktn1	kinectin 1	-0.687	-0.964	0.036
74142284	DSE	dermatan sulfate epimerase	-0.667	-0.965	0.035
157818909	Zim1	zinc finger, imprinted 1	-0.665	-0.995	0.005
305682588	PDZD7	PDZ domain containing 7	-0.660	-0.966	0.034
201861690	TPK1	thiamin pyrophosphokinase 1	-0.657	-0.969	0.031
114145782	MORN5	MORN repeat containing 5	-0.657	-0.974	0.026
164565435	SYNJ2	synaptojanin 2	-0.654	-0.974	0.026
157822365	LAMC3	laminin subunit gamma 3	-0.651	-0.980	0.020
13786136	PDGFC	platelet derived growth factor C	-0.642	-0.980	0.020
158508544	DDR2	discoidin domain receptor tyrosine kinase 2	-0.640	-0.965	0.035
300794353	FANCL	FA complementation group L	-0.639	-0.976	0.024
157786864	PHOSPHO1	phosphoethanolamine/phosphocholine phosphatase 1	-0.638	-0.989	0.011
392355027	TANGO6	transport and golgi organization 6 homolog	-0.638	-0.971	0.029
149061352	ADAM12	ADAM metallopeptidase domain 12	-0.638	-0.980	0.020
672060362	ELFN2	extracellular leucine rich repeat and fibronectin type III domain containing 2	-0.636	-0.998	0.002
187282311	ISLR	immunoglobulin superfamily containing leucine rich repeat	-0.635	-0.961	0.039
19424350	GBP2	guanylate binding protein 2	-0.635	-0.985	0.015
58865948	CREB3L2	cAMP responsive element binding protein 3 like 2	-0.634	-0.955	0.045
34734058	HCK	HCK proto-oncogene, Src family tyrosine kinase	-0.631	-0.960	0.040
397529557	C8orf58	chromosome 8 open reading frame 58	-0.629	-0.980	0.020
13591916	ABCC6	ATP binding cassette subfamily C member 6	-0.628	-0.977	0.023
16758622	IFT172	intraflagellar transport 172	-0.627	-0.961	0.039
478732983	MAP3K5	mitogen-activated protein kinase kinase kinase 5	-0.616	-0.980	0.020

402478640	HTRA3	HtrA serine peptidase 3	-0.608	-0.955	0.045
157819065	ADAMTS15	ADAM metallopeptidase with thrombospondin type 1 motif 15	-0.604	-0.968	0.032
293347888	SRBD1	S1 RNA binding domain 1	-0.598	-0.983	0.017
564387543	UGGT2	UDP-glucose glycoprotein glucosyltransferase 2	-0.596	-0.997	0.003
157817670	SLC2A10	solute carrier family 2 member 10	-0.595	-0.980	0.020
6978737	CYP1B1	cytochrome P450 family 1 subfamily B member 1	-0.593	-0.952	0.048
108935976	DISC1	DISC1 scaffold protein	-0.590	-0.958	0.042
197927123	LYRM7	LYR motif containing 7	-0.587	-0.970	0.030
672086986	SLC38A5	solute carrier family 38 member 5	-0.583	-0.958	0.042
564394999	CLGN	calmegin	-0.582	-0.950	0.050
164519095	SLC9A2	solute carrier family 9 member A2	-0.578	-0.962	0.038
16758560	WIF1	WNT inhibitory factor 1	-0.573	-0.977	0.023
19173754	TESK2	testis associated actin remodelling kinase 2	-0.567	-0.985	0.015
564390348	Klhl3	kelch-like family member 3	-0.566	-0.998	0.002
62078799	QRSL1	glutaminyl-tRNA amidotransferase subunit QRSL1	-0.566	-0.962	0.038
57012436	Krt10	keratin 10	-0.562	-0.989	0.011
8393469	S1PR2	sphingosine-1-phosphate receptor 2	-0.561	-0.983	0.017
569009290	TENM1	teneurin transmembrane protein 1	-0.559	-0.985	0.015
300798350	LRRK1	leucine rich repeat kinase 1	-0.554	-0.996	0.004
42476287	TGM2	transglutaminase 2	-0.553	-0.964	0.036
171846640	FBLN1	fibulin 1	-0.543	-0.976	0.024
6754808	NDP	norrin cystine knot growth factor NDP	-0.538	-0.957	0.043
9507045	RGS5	regulator of G protein signaling 5	-0.538	-0.951	0.049
20302097	PIGL	phosphatidylinositol glycan anchor biosynthesis class L	-0.534	-0.970	0.030
46310239	SIDT1	SID1 transmembrane family member 1	-0.528	-0.973	0.027
564319108	ADGRA2	adhesion G protein-coupled receptor A2	-0.527	-0.951	0.049
157818843	EXTL1	exostosin like glycosyltransferase 1	-0.527	-0.984	0.016
19424232	CSF2RB	colony stimulating factor 2 receptor subunit beta	-0.524	-0.993	0.007
124244050	PPIP5K1	diphosphoinositol pentakisphosphate kinase 1	-0.522	-0.979	0.021
157822555	RIN3	Ras and Rab interactor 3	-0.520	-0.980	0.020
148692356	ARHGEF1	Rho guanine nucleotide exchange factor 1	-0.517	-0.985	0.015
392338379	SLC26A8	solute carrier family 26 member 8	-0.514	-0.968	0.032

149047863	LOC690190	hypothetical protein LOC690190	-0.514	-0.954	0.046
293344916	COL6A1	collagen type VI alpha 1 chain	-0.508	-0.973	0.027
148698795	GPX7	glutathione peroxidase 7	-0.507	-0.987	0.013
312922352	TTF2	transcription termination factor 2	-0.506	-0.980	0.020
654824082	Fbxl21	F-box and leucine-rich repeat protein 21	-0.500	-0.961	0.039
37693510	Bst2	bone marrow stromal cell antigen 2	-0.500	-0.980	0.020
58865396	FIGNL1	fidgetin like 1	-0.497	-0.969	0.031
50233928	TMEM159	transmembrane protein 159	-0.497	-0.955	0.045
84662732	DNASE1L1	deoxyribonuclease 1 like 1	-0.494	-0.999	0.001
62078635	CCDC153	coiled-coil domain containing 153	-0.494	-0.988	0.012
399220341	SLC2A13	solute carrier family 2 member 13	-0.492	-0.993	0.007
157819347	CDC6	cell division cycle 6	-0.492	-0.985	0.015
564301698	LY75	lymphocyte antigen 75	-0.489	-0.955	0.045
149058686	PIGR	polymeric immunoglobulin receptor	-0.485	-0.968	0.032
209954806	PIGN	phosphatidylinositol glycan anchor biosynthesis class N	-0.485	-0.969	0.031
564399546	STARD8	StAR related lipid transfer domain containing 8	-0.484	-0.988	0.012
157073947	C1orf74	chromosome 1 open reading frame 74	-0.478	-0.975	0.025
57528252	QPRT	quinolinate phosphoribosyltransferase	-0.475	-0.964	0.036
392332443	PRKDC	protein kinase, DNA-activated, catalytic subunit	-0.475	-0.957	0.043
148695091	BBS5	Bardet-Biedl syndrome 5	-0.473	-0.956	0.044
312922379	TNN	tenascin N	-0.469	-0.982	0.018
157816939	WASHC3	WASH complex subunit 3	-0.469	-0.994	0.006
19924069	SPON2	spondin 2	-0.467	-0.980	0.020
75905809	AKAP12	A-kinase anchoring protein 12	-0.466	-1.000	0.000
41056215	XRCC5	X-ray repair cross complementing 5	-0.466	-0.959	0.041
672044191	TBCK	TBC1 domain containing kinase	-0.465	-0.954	0.046
56090632	DMAC2L	distal membrane arm assembly complex 2 like	-0.463	-0.988	0.012
148669431	DNAJC27	DnaJ heat shock protein family (Hsp40) member C27	-0.459	-0.980	0.020
68163403	SLC46A3	solute carrier family 46 member 3	-0.458	-0.976	0.024
148699893	COL6A1	collagen type VI alpha 1 chain	-0.457	-0.981	0.019
56090411	POLE3	DNA polymerase epsilon 3, accessory subunit	-0.456	-0.978	0.022
149067372	MTERF2	mitochondrial transcription termination factor 2	-0.455	-0.988	0.012
157817743	CDH5	cadherin 5	-0.454	-0.980	0.020
9437326	SLC4A4	solute carrier family 4 member 4	-0.453	-0.986	0.014
158138494	PTPRC	protein tyrosine phosphatase receptor type C	-0.451	-0.979	0.021

33414515	PXK	PX domain containing serine/threonine kinase like	-0.450	-0.990	0.010
58865380	STAT2	signal transducer and activator of transcription 2	-0.450	-0.954	0.046
201066407	EAPP	E2F associated phosphoprotein	-0.444	-0.985	0.015
157786756	CDC45	cell division cycle 45	-0.442	-0.976	0.024
16758186	SLCO1C1	solute carrier organic anion transporter family member 1C1	-0.441	-0.970	0.030
149022245	SCRN3	secernin 3	-0.437	-0.965	0.035
8393057	SERPINH1	serpin family H member 1	-0.436	-0.973	0.027
148686921	SLC24A4	solute carrier family 24 member 4	-0.435	-0.997	0.003
404312655	SDR42E1	short chain dehydrogenase/reductase family 42E, member 1	-0.435	-0.979	0.021
172045714	MIIP	migration and invasion inhibitory protein	-0.433	-0.968	0.032
157821557	CD248	CD248 molecule	-0.426	-0.957	0.043
76443683	LOC100912042/Surf2	surfeit 2	-0.423	-0.999	0.001
300796997	ARHGAP28	Rho GTPase activating protein 28	-0.423	-0.975	0.025
210032365	HSP90B1	heat shock protein 90 beta family member 1	-0.421	-0.988	0.012
56090564	GALM	galactose mutarotase	-0.420	-0.988	0.012
157823279	CGNL1	cingulin like 1	-0.418	-0.986	0.014
74218228	HNRNPC	heterogeneous nuclear ribonucleoprotein C	-0.418	-0.989	0.011
564329859	COA4	cytochrome c oxidase assembly factor 4 homolog	-0.417	-0.958	0.042
83642834	NAGK	N-acetylglucosamine kinase	-0.416	-0.974	0.026
13994179	SLC24A2	solute carrier family 24 member 2	-0.416	-0.967	0.033
564342737	DTWD1	DTW domain containing 1	-0.416	-0.959	0.041
62078539	Pagr1	Paxip1-associated glutamate-rich protein 1	-0.415	-0.996	0.004
62945312	CXCL16	C-X-C motif chemokine ligand 16	-0.414	-0.988	0.012
68342019	LRRC17	leucine rich repeat containing 17	-0.413	-0.996	0.004
672085293	FANCA	FA complementation group A	-0.412	-0.958	0.042
13591949	GATM	glycine amidinotransferase	-0.409	-0.976	0.024
23463307	RIOX2	ribosomal oxygenase 2	-0.409	-0.973	0.027
157786690	PRKCA	protein kinase C alpha	-0.408	-0.977	0.023
149049048	RECQL	RecQ like helicase	-0.403	-0.999	0.001
127140886	EML6	EMAP like 6	-0.403	-0.999	0.001
58865466	SLC37A1	solute carrier family 37 member 1	-0.401	-0.951	0.049
77695926	STAT1	signal transducer and activator of transcription 1	-0.399	-0.986	0.014

56605714	NDUFAF7	NADH:ubiquinone oxidoreductase complex assembly factor 7	-0.399	-0.972	0.028
78187977	TCF19	transcription factor 19	-0.393	-0.971	0.029
149052857	KCNJ12	potassium inwardly rectifying channel subfamily J member 12	-0.392	-0.974	0.026
187937124	TMEM126B	transmembrane protein 126B	-0.389	-0.979	0.021
124286858	B230217C12Rik	RIKEN cDNA B230217C12 gene	-0.389	-0.970	0.030
51948488	SIRT5	sirtuin 5	-0.388	-0.951	0.049
148690851	RCN3	reticulocalbin 3	-0.381	-0.972	0.028
564305413	E130308A19Rik	RIKEN cDNA E130308A19 gene	-0.381	-0.995	0.005
18426846	DCBLD2	discoidin, CUB and LCCL domain containing 2	-0.378	-0.969	0.031
219282679	ZNF43	zinc finger protein 43	-0.374	-0.974	0.026
672083553	SLC14A1	solute carrier family 14 member 1 (Kidd blood group)	-0.373	-0.978	0.022
189011677	GLB1L	galactosidase beta 1 like	-0.373	-0.959	0.041
113061	CHRNA3	cholinergic receptor nicotinic alpha 3 subunit	-0.372	-0.997	0.003
8393861	HPCAL4	hippocalcin like 4	-0.370	-0.986	0.014
157819959	PCDHB2	protocadherin beta 2	-0.369	-0.985	0.015
158303308	PCCA	propionyl-CoA carboxylase subunit alpha	-0.369	-0.998	0.002
157819753	RCN1	reticulocalbin 1	-0.368	-0.953	0.047
9247217	MSX1	msh homeobox 1	-0.368	-0.966	0.034
157822461	C20orf194	chromosome 20 open reading frame 194	-0.366	-0.971	0.029
158534064	RET	ret proto-oncogene	-0.365	-0.963	0.037
219278723	ZNF23	zinc finger protein 23	-0.362	-0.955	0.045
16758712	PDIA4	protein disulfide isomerase family A member 4	-0.362	-0.978	0.022
281604225	PUS7	pseudouridine synthase 7	-0.362	-0.983	0.017
55741549	MRPL13	mitochondrial ribosomal protein L13	-0.360	-0.955	0.045
29293811	SERPINF1	serpin family F member 1	-0.359	-0.992	0.008
384368019	Snhg11	small nucleolar RNA host gene 11	-0.356	-0.979	0.021
78042613	NICN1	nicolin 1	-0.354	-0.965	0.035
594191048	C19orf54	chromosome 19 open reading frame 54	-0.352	-0.969	0.031
61557118	PCGF6	polycomb group ring finger 6	-0.351	-0.967	0.033
16758024	SYT9	synaptotagmin 9	-0.351	-0.968	0.032
148747194	SLC16A7	solute carrier family 16 member 7	-0.349	-0.953	0.047
564329392	FLNA	filamin A	-0.347	-0.973	0.027
56119120	SNF8	SNF8 subunit of ESCRT-II	-0.343	-0.998	0.002
62078847	TSEN2	tRNA splicing endonuclease subunit 2	-0.343	-0.990	0.010

300795140	TAF1	TATA-box binding protein associated factor 1	-0.342	-0.964	0.036
77157795	MAL2	mal, T cell differentiation protein 2 (gene/pseudogene)	-0.342	-0.950	0.050
71361669	CIT	citron rho-interacting serine/threonine kinase	-0.336	-0.980	0.020
57527061	ZGPAT	zinc finger CCCH-type and G-patch domain containing	-0.335	-0.971	0.029
148683194	INTS3	integrator complex subunit 3	-0.335	-0.984	0.016
157819569	TEAD2	TEA domain transcription factor 2	-0.333	-0.958	0.042
300794275	MFSD10	major facilitator superfamily domain containing 10	-0.330	-0.992	0.008
564309649	CCDC159	coiled-coil domain containing 159	-0.329	-0.993	0.007
22024392	KIF1C	kinesin family member 1C	-0.328	-0.992	0.008
77628027	PSMC3IP	PSMC3 interacting protein	-0.328	-0.993	0.007
61557206	ZBTB16	zinc finger and BTB domain containing 16	-0.326	-0.995	0.005
564344373	ZMYND8	zinc finger MYND-type containing 8	-0.325	-0.975	0.025
149045696	Ccl27a	chemokine (C-C motif) ligand 27A	-0.325	-0.998	0.002
51036680	SLC29A3	solute carrier family 29 member 3	-0.322	-0.997	0.003
58865398	LAP3	leucine aminopeptidase 3	-0.320	-0.976	0.024
672033554	LOC102557335	uncharacterized LOC102557335	-0.319	-0.959	0.041
564304076	FGD5	FYVE, RhoGEF and PH domain containing 5	-0.319	-0.984	0.016
672050244	APLF	aprataxin and PNKP like factor	-0.318	-0.990	0.010
148702301	CYB561	cytochrome b561	-0.317	-0.976	0.024
157823879	NUDT12	nudix hydrolase 12	-0.317	-0.991	0.009
157819457	MAP3K14	mitogen-activated protein kinase kinase kinase 14	-0.317	-0.996	0.004
40018538	ADI1	acireductone dioxygenase 1	-0.316	-0.964	0.036
300797242	SPG11	SPG11 vesicle trafficking associated, spatacsin	-0.316	-0.991	0.009
57528352	DMAC2	distal membrane arm assembly complex 2	-0.315	-1.000	0.000
281332095	RB1	RB transcriptional corepressor 1	-0.313	-0.998	0.002
157786608	MRPL55	mitochondrial ribosomal protein L55	-0.313	-0.998	0.002
672084625	LOC100909409 (includes others)	RGD1562660	-0.312	-0.968	0.032
109484871	HERC1	HECT and RLD domain containing E3 ubiquitin protein ligase family member 1	-0.310	-0.964	0.036
564336403	EXOSC8	exosome component 8	-0.309	-0.979	0.021
167860097	FN3KRP	fructosamine 3 kinase related protein	-0.308	-0.997	0.003

12621108	NR1I3	nuclear receptor subfamily 1 group I member 3	-0.308	-0.973	0.027
966975500	MMP17	matrix metallopeptidase 17	-0.306	-0.951	0.049
29789369	PTPRG	protein tyrosine phosphatase receptor type G	-0.306	-0.950	0.050
545532952	EIF4E3	eukaryotic translation initiation factor 4E family member 3	-0.305	-0.991	0.009
399124777	GLS2	glutaminase 2	-0.305	-0.994	0.006
568961602	VPS13C	vacuolar protein sorting 13 homolog C	-0.304	-0.964	0.036
300796069	THADA	THADA armadillo repeat containing	-0.303	-0.984	0.016
198041989	PARVB	parvin beta	-0.302	-1.000	0.000
157820737	NUSAP1	nucleolar and spindle associated protein 1	-0.298	-0.999	0.001
19173736	SCPEP1	serine carboxypeptidase 1	-0.297	-0.965	0.035
162287198	HSD17B4	hydroxysteroid 17-beta dehydrogenase 4	-0.297	-0.962	0.038
149032924	ARG1	arginase 1	-0.296	-0.961	0.039
8393992	PMP22	peripheral myelin protein 22	-0.295	-0.986	0.014
6978888	GFRA1	GDNF family receptor alpha 1	-0.294	-0.990	0.010
53850628	NDUFS1	NADH:ubiquinone oxidoreductase core subunit S1	-0.294	-0.961	0.039
167560911	SGF29	SAGA complex associated factor 29	-0.293	-0.992	0.008
564358911	CHPT1	choline phosphotransferase 1	-0.292	-0.959	0.041
27545388	ABCA5	ATP binding cassette subfamily A member 5	-0.291	-0.969	0.031
74223968	5031425E22Rik	RIKEN cDNA 5031425E22 gene	-0.290	-0.975	0.025
149057745	NEK3	NIMA related kinase 3	-0.290	-0.999	0.001
149063995	GMPR2	guanosine monophosphate reductase 2	-0.289	-0.996	0.004
12844128	CCDC90B	coiled-coil domain containing 90B	-0.288	-0.985	0.015
157820517	CARD6	caspase recruitment domain family member 6	-0.287	-0.967	0.033
564387894	BTD	biotinidase	-0.287	-0.965	0.035
564329612	ME3	malic enzyme 3	-0.284	-0.972	0.028
149034870	RNF6	ring finger protein 6	-0.283	-0.997	0.003
157823996	ELK3	ETS transcription factor ELK3	-0.282	-0.978	0.022
564367076	Mocs1	molybdenum cofactor synthesis 1	-0.281	-0.971	0.029
564317068	CCDC149	coiled-coil domain containing 149	-0.281	-0.981	0.019
142349612	GLUL	glutamate-ammonia ligase	-0.279	-0.987	0.013
13928886	MAP2K1	mitogen-activated protein kinase kinase 1	-0.278	-0.991	0.009
226874871	OMG	oligodendrocyte myelin glycoprotein	-0.274	-0.965	0.035
134948398	PDS5A	PDS5 cohesin associated factor A	-0.274	-0.969	0.031

157822627	PLXDC2	plexin domain containing 2	-0.273	-0.961	0.039
62078997	WDR1	WD repeat domain 1	-0.273	-0.954	0.046
157820147	TNFRSF10A	TNF receptor superfamily member 10a	-0.272	-0.968	0.032
197333840	CAMKMT	calmodulin-lysine N-methyltransferase	-0.270	-0.977	0.023
149022319	AGPS	alkylglycerone phosphate synthase	-0.270	-0.986	0.014
149068830	SLCO2B1	solute carrier organic anion transporter family member 2B1	-0.270	-0.975	0.025
57164113	NSDHL	NAD(P) dependent steroid dehydrogenase-like	-0.269	-0.994	0.006
576796148	MAP7D2	MAP7 domain containing 2	-0.269	-0.950	0.050
157817953	RPGRIP1L	RPGRIP1 like	-0.269	-0.980	0.020
74354506	ACBD5	acyl-CoA binding domain containing 5	-0.269	-0.985	0.015
197313676	AIG1	androgen induced 1	-0.268	-0.965	0.035
392339847	CADPS2	calcium dependent secretion activator 2	-0.268	-0.999	0.001
38181552	SCG2	secretogranin II	-0.267	-0.998	0.002
149017535	HDAC10	histone deacetylase 10	-0.267	-0.979	0.021
157819753	RCN1	reticulocalbin 1	-0.266	-0.968	0.032
300795339	RYR2	ryanodine receptor 2	-0.265	-0.957	0.043
564382316	HSD11B1	hydroxysteroid 11-beta dehydrogenase 1	-0.265	-0.991	0.009
149041411	SC5D	sterol-C5-desaturase	-0.264	-0.998	0.002
281427203	TMEM260	transmembrane protein 260	-0.262	-0.952	0.048
564344520	LOC102555457	engulfment and cell motility protein 2-like	-0.261	-0.960	0.040
149060525	FSTL1	follistatin like 1	-0.258	-0.965	0.035
114145534	Mtap	methylthioadenosine phosphorylase	-0.258	-0.958	0.042
17865325	GLRB	glycine receptor beta	-0.256	-0.961	0.039
16758538	RASGRF2	Ras protein specific guanine nucleotide releasing factor 2	-0.254	-0.973	0.027
61097902	ABCC4	ATP binding cassette subfamily C member 4	-0.248	-0.951	0.049
187469267	GPRC5B	G protein-coupled receptor class C group 5 member B	-0.247	-0.981	0.019
296439269	PHF10	PHD finger protein 10	-0.245	-0.953	0.047
6978631	CD4	CD4 molecule	-0.245	-0.978	0.022
564391295	DUSP22	dual specificity phosphatase 22	-0.244	-0.958	0.042
290563168	DUSP3	dual specificity phosphatase 3	-0.243	-0.978	0.022
12018300	AKAP6	A-kinase anchoring protein 6	-0.242	-0.986	0.014
300793894	URB1	URB1 ribosome biogenesis homolog	-0.242	-0.970	0.030
187937143	C2orf42	chromosome 2 open reading frame 42	-0.241	-0.959	0.041

97537309	SYNJ1	synaptojanin 1	-0.240	-0.973	0.027
48040475	GCNT2	glucosaminyl (N-acetyl) transferase 2 (I blood group)	-0.233	-0.955	0.045
584277046	SLC1A3	solute carrier family 1 member 3	-0.232	-0.984	0.016
213688380	GXYLT1	glucoside xylosyltransferase 1	-0.232	-0.971	0.029
293346766	TCAF1	TRPM8 channel associated factor 1	-0.232	-0.990	0.010
9457244	RBBP9	RB binding protein 9, serine hydrolase	-0.231	-0.965	0.035
58865718	HERC4	HECT and RLD domain containing E3 ubiquitin protein ligase 4	-0.228	-0.989	0.011
149062310	BSCL2	BSCL2 lipid droplet biogenesis associated, seipin	-0.228	-0.999	0.001
62079099	ORC5	origin recognition complex subunit 5	-0.228	-0.966	0.034
672050038	NDNF	neuron derived neurotrophic factor	-0.226	-0.974	0.026
205277356	TVP23B	trans-golgi network vesicle protein 23 homolog B	-0.226	-0.974	0.026
62079015	PRXL2A	peroxiredoxin like 2A	-0.226	-0.953	0.047
392348438	NIPAL3	NIPA like domain containing 3	-0.223	-0.968	0.032
157786994	C1orf21	chromosome 1 open reading frame 21	-0.222	-0.979	0.021
78369663	SLC38A9	solute carrier family 38 member 9	-0.222	-0.995	0.005
56605770	RRP8	ribosomal RNA processing 8	-0.221	-0.990	0.010
148695758	CAPRIN1	cell cycle associated protein 1	-0.221	-0.978	0.022
158631207	YIF1A	Yip1 interacting factor homolog A, membrane trafficking protein	-0.220	-0.973	0.027
158186672	Nedd4	neural precursor cell expressed, developmentally down-regulated 4	-0.220	-0.980	0.020
157822873	FBH1	F-box DNA helicase 1	-0.219	-1.000	0.000
9506469	CD47	CD47 molecule	-0.218	-0.966	0.034
564298436	WDR11	WD repeat domain 11	-0.216	-0.989	0.011
8393643	KCNAB1	potassium voltage-gated channel subfamily A member regulatory beta subunit 1	-0.214	-0.970	0.030
672066638	CLEC16A	C-type lectin domain containing 16A	-0.212	-0.967	0.033
564397593	RAB36	RAB36, member RAS oncogene family	-0.212	-0.969	0.031
415703079	NEBL	nebulette	-0.211	-0.973	0.027
25742763	HSPA5	heat shock protein family A (Hsp70) member 5	-0.211	-0.990	0.010
158749540	NPEPPS	aminopeptidase puromycin sensitive	-0.210	-0.971	0.029
18959250	PRKCD	protein kinase C delta	-0.210	-0.993	0.007
37359962	PLPPR4	phospholipid phosphatase related 4	-0.207	-0.969	0.031
225543229	TIAM1	TIAM Rac1 associated GEF 1	-0.207	-1.000	0.000
148667192	LRTM2	leucine rich repeats and transmembrane domains 2	-0.207	-0.984	0.016

564372562	PFAS	phosphoribosylformylglycinamidine synthase	-0.206	-0.952	0.048
54019432	PCDHA7	protocadherin alpha 7	-0.205	-0.967	0.033
6981504	ATXN1	ataxin 1	-0.204	-1.000	0.000
157818191	SETD6	SET domain containing 6, protein lysine methyltransferase	-0.203	-0.989	0.011
13027430	WDR7	WD repeat domain 7	-0.202	-0.952	0.048
142385975	RNF25	ring finger protein 25	-0.202	-0.995	0.005
48675867	PLPP3	phospholipid phosphatase 3	-0.201	-0.978	0.022
157786582	C16orf89	chromosome 16 open reading frame 89	-0.201	-0.955	0.045
38454284	PPM1E	protein phosphatase, Mg2+/Mn2+ dependent 1E	-0.201	-0.974	0.026
148707634	SHISA4	shisa family member 4	-0.201	-0.953	0.047
149053315	CAMKK1	calcium/calmodulin dependent protein kinase kinase 1	-0.200	-0.972	0.028
62078801	MEF2A	myocyte enhancer factor 2A	-0.199	-0.961	0.039
61556891	OSBPL2	oxysterol binding protein like 2	-0.198	-0.995	0.005
564347675	AAK1	AP2 associated kinase 1	-0.195	-0.981	0.019
63706033	Gm5174 (includes others)	serine/threonine kinase, pseudogene 1	-0.192	-0.980	0.020
148696094	TUBGCP4	tubulin gamma complex associated protein 4	-0.189	-0.953	0.047
157818171	PLK4	polo like kinase 4	-0.188	-0.962	0.038
68163551	TBC1D22B	TBC1 domain family member 22B	-0.187	-0.971	0.029
564334013	GBF1	golgi brefeldin A resistant guanine nucleotide exchange factor 1	-0.187	-0.951	0.049
157821901	PNMA3	PNMA family member 3	-0.186	-1.000	0.000
672088942	ATP2B3	ATPase plasma membrane Ca2+ transporting 3	-0.186	-0.955	0.045
312283667	WNK1	WNK lysine deficient protein kinase 1	-0.185	-0.984	0.016
197209847	JAK1	Janus kinase 1	-0.185	-0.973	0.027
16923964	CNTN1	contactin 1	-0.185	-0.967	0.033
76881830	Kcnip2	potassium voltage-gated channel interacting protein 2	-0.183	-0.970	0.030
84490431	DNM3	dynamin 3	-0.182	-0.969	0.031
32185285	BCL2L2	BCL2 like 2	-0.181	-0.981	0.019
157817620	PSD2	pleckstrin and Sec7 domain containing 2	-0.180	-0.991	0.009
672088357	ZCCHC18	zinc finger CCHC-type containing 18	-0.180	-0.971	0.029
19173794	LOC678813/Marf1	meiosis regulator and mRNA stability factor 1	-0.180	-0.967	0.033

187468990	DNAJB2	DnaJ heat shock protein family (Hsp40) member B2	-0.180	-0.951	0.049
39930507	KCNK15	potassium two pore domain channel subfamily K member 15	-0.180	-0.956	0.044
6981672	Tpm4	tropomyosin 4	-0.178	-0.984	0.016
293349000	ARHGAP42	Rho GTPase activating protein 42	-0.178	-0.976	0.024
13569846	PARVA	parvin alpha	-0.178	-0.986	0.014
201023331	MAPK11	mitogen-activated protein kinase 11	-0.176	-1.000	0.000
564390898	KIF13A	kinesin family member 13A	-0.176	-0.953	0.047
157818193	TTPAL	alpha tocopherol transfer protein like	-0.176	-0.997	0.003
78000203	Tpm1	tropomyosin 1, alpha	-0.174	-0.967	0.033
61557085	SPTBN1	spectrin beta, non-erythrocytic 1	-0.174	-0.987	0.013
392333710	COL4A2	collagen type IV alpha 2 chain	-0.173	-0.974	0.026
564387640	DOCK9	dedicator of cytokinesis 9	-0.172	-0.976	0.024
61557212	CIAO3	cytosolic iron-sulfur assembly component 3	-0.170	-0.981	0.019
396941666	Dync1i2	dynein cytoplasmic 1 intermediate chain 2	-0.169	-0.951	0.049
62339281	ADAM9	ADAM metallopeptidase domain 9	-0.168	-0.957	0.043
817472062	CDK5RAP3	CDK5 regulatory subunit associated protein 3	-0.167	-0.975	0.025
564335900	ZFHX4	zinc finger homeobox 4	-0.167	-0.964	0.036
6981076	IDE	insulin degrading enzyme	-0.165	-0.970	0.030
149046296	CREG2	cellular repressor of E1A stimulated genes 2	-0.165	-0.987	0.013
13242243	AXIN2	axin 2	-0.164	-0.961	0.039
392344250	SPTY2D1	SPT2 chromatin protein domain containing 1	-0.161	-0.957	0.043
149052635	TNIP1	TNFAIP3 interacting protein 1	-0.161	-0.953	0.047
157819829	HACD3	3-hydroxyacyl-CoA dehydratase 3	-0.159	-0.994	0.006
198278547	TMEM41A	transmembrane protein 41A	-0.159	-0.985	0.015
52345385	PDIA6	protein disulfide isomerase family A member 6	-0.159	-0.962	0.038
61556993	HIBCH	3-hydroxyisobutyryl-CoA hydrolase	-0.158	-0.966	0.034
209529636	PPA2	inorganic pyrophosphatase 2	-0.158	-0.985	0.015
18034785	ABCB6	ATP binding cassette subfamily B member 6 (Langereis blood group)	-0.158	-0.983	0.017
564299821	PARP8	poly(ADP-ribose) polymerase family member 8	-0.155	-0.961	0.039
18266726	PAICS	phosphoribosylaminoimidazole carboxylase and phosphoribosylaminoimidazolesuccino carboxamide synthase	-0.155	-0.993	0.007
6649914	GDF11	growth differentiation factor 11	-0.154	-0.958	0.042

564385664	FERMT2	fermitin family member 2	-0.154	-0.961	0.039
113461996	COA5	cytochrome c oxidase assembly factor 5	-0.149	-0.999	0.001
50510837	KIAA1191	KIAA1191	-0.148	-0.981	0.019
717324516	SCN8A	sodium voltage-gated channel alpha subunit 8	-0.147	-0.990	0.010
672016955	MAP3K20	mitogen-activated protein kinase kinase kinase 20	-0.147	-0.956	0.044
300794996	NDST3	N-deacetylase and N-sulfotransferase 3	-0.147	-0.955	0.045
55742755	CTNNA1	catenin alpha 1	-0.146	-0.972	0.028
71043702	TM9SF4	transmembrane 9 superfamily member 4	-0.145	-0.950	0.050
730229363	RALGAPA1	Ral GTPase activating protein catalytic subunit alpha 1	-0.142	-0.992	0.008
16758736	NLGN1	neuroligin 1	-0.139	-0.965	0.035
25742686	ELOVL6	ELOVL fatty acid elongase 6	-0.136	-0.968	0.032
157823401	PIGH	phosphatidylinositol glycan anchor biosynthesis class H	-0.135	-0.982	0.018
6981166	PLAGL1	PLAG1 like zinc finger 1	-0.133	-0.988	0.012
564345487	RINT1	RAD50 interactor 1	-0.132	-0.998	0.002
148747541	HNRNPU	heterogeneous nuclear ribonucleoprotein U	-0.130	-0.972	0.028
9507177	USO1	USO1 vesicle transport factor	-0.130	-0.971	0.029
398650648	SLC8A1	solute carrier family 8 member A1	-0.127	-0.956	0.044
37359832	SCRN1	secernin 1	-0.127	-0.972	0.028
55926219	DDX39A	DExD-box helicase 39A	-0.126	-0.985	0.015
77415383	HSPA8	heat shock protein family A (Hsp70) member 8	-0.125	-0.994	0.006
58865700	GRWD1	glutamate rich WD repeat containing 1	-0.123	-0.980	0.020
157820919	POLE4	DNA polymerase epsilon 4, accessory subunit	-0.123	-0.968	0.032
564301782	CERS6	ceramide synthase 6	-0.122	-0.971	0.029
158254369	CDK10	cyclin dependent kinase 10	-0.121	-0.999	0.001
74229032	TPCN1	two pore segment channel 1	-0.121	-0.983	0.017
209870013	ITSN1	intersectin 1	-0.121	-0.961	0.039
476007242	EPS8	epidermal growth factor receptor pathway substrate 8	-0.117	-0.997	0.003
201066352	ANKRD6	ankyrin repeat domain 6	-0.116	-0.965	0.035
8393390	GABRB3	gamma-aminobutyric acid type A receptor subunit beta3	-0.115	-0.976	0.024
29789269	GRIA1	glutamate ionotropic receptor AMPA type subunit 1	-0.115	-0.970	0.030

149059529	LOC100910558	uncharacterized LOC100910558	-0.114	-0.991	0.009
404247435	YLPM1	YLP motif containing 1	-0.113	-0.961	0.039
60360272	KLHL5	kelch like family member 5	-0.113	-0.962	0.038
564353678	USP48	ubiquitin specific peptidase 48	-0.112	-0.991	0.009
8393038	CAPN2	calpain 2	-0.111	-0.966	0.034
148673922	HSPH1	heat shock protein family H (Hsp110) member 1	-0.110	-0.978	0.022
694981804	CLDN5	claudin 5	-0.108	-0.968	0.032
89337260	FTO	FTO alpha-ketoglutarate dependent dioxygenase	-0.107	-0.961	0.039
274326692	UQCC3	ubiquinol-cytochrome c reductase complex assembly factor 3	-0.106	-0.956	0.044
300794843	IQGAP3	IQ motif containing GTPase activating protein 3	-0.100	-0.958	0.042
392353562	ATP8A2	ATPase phospholipid transporting 8A2	-0.099	-0.970	0.030
157819175	Gpr165	G protein-coupled receptor 165	-0.099	-0.972	0.028
403377905	SRGAP2	SLIT-ROBO Rho GTPase activating protein 2	-0.098	-0.989	0.011
154800420	GNL3L	G protein nucleolar 3 like	-0.093	-0.986	0.014
54035294	ADH5	alcohol dehydrogenase 5 (class III), chi polypeptide	-0.080	-0.984	0.016
148747375	CDS1	CDP-diacylglycerol synthase 1	-0.078	-0.960	0.040
568986834	KCNMA1	potassium calcium-activated channel subfamily M alpha 1	-0.075	-0.967	0.033
297206894	E4F1	E4F transcription factor 1	-0.074	-0.964	0.036
34536836	EHD3	EH domain containing 3	-0.071	-0.990	0.010
564375060	SLC39A11	solute carrier family 39 member 11	-0.070	-0.977	0.023
148747528	PTK2B	protein tyrosine kinase 2 beta	-0.066	-0.963	0.037
52138624	SLC25A20	solute carrier family 25 member 20	-0.066	-0.958	0.042
255918181	NUS1	NUS1 dehydrodolichyl diphosphate synthase subunit	-0.062	-0.984	0.016
77020248	PFKFB2	6-phosphofructo-2-kinase/fructose-2,6-biphosphatase 2	-0.060	-0.960	0.040
20302113	STIP1	stress induced phosphoprotein 1	-0.060	-0.976	0.024
408535187	PRDM11	PR/SET domain 11	-0.058	-0.966	0.034
13385318	KDELR2	KDEL endoplasmic reticulum protein retention receptor 2	-0.057	-0.966	0.034
189027133	TTC30B	tetratricopeptide repeat domain 30B	-0.044	-0.993	0.007
20809990	XPA	XPA, DNA damage recognition and repair factor	-0.043	-0.956	0.044
62079109	LANCL2	LanC like 2	-0.034	-0.963	0.037
157818471	PPM1L	protein phosphatase, Mg2+/Mn2+ dependent 1L	-0.032	-0.971	0.029

45478098	CMTR1	cap methyltransferase 1	0.046	0.983	0.017
55250051	TXNRD1	thioredoxin reductase 1	0.056	0.964	0.036
58865796	PTDSS1	phosphatidylserine synthase 1	0.057	0.995	0.005
25453374	PEX14	peroxisomal biogenesis factor 14	0.067	0.997	0.003
62089200	ZDHHC9	zinc finger DHHC-type palmitoyltransferase 9	0.068	1.000	0.000
25742568	DPYSL3	dihydropyrimidinase like 3	0.069	0.990	0.010
755492511	MAP4K4	mitogen-activated protein kinase kinase kinase kinase 4	0.078	0.984	0.016
60360532	OSBPL6	oxysterol binding protein like 6	0.083	0.984	0.016
672074758	NCSTN	nicastrin	0.083	0.999	0.001
564363529	NCAM1	neural cell adhesion molecule 1	0.091	0.978	0.022
209447030	DDX27	DEAD-box helicase 27	0.097	0.987	0.013
119618921	RAN	RAN, member RAS oncogene family	0.098	0.958	0.042
970596961	MAPK10	mitogen-activated protein kinase 10	0.099	0.999	0.001
166064004	GTF3A	general transcription factor IIIA	0.101	0.998	0.002
157821483	BORCS5	BLOC-1 related complex subunit 5	0.103	0.955	0.045
42476292	TALDO1	transaldolase 1	0.106	0.982	0.018
672054841	RCC2	regulator of chromosome condensation 2	0.107	0.951	0.049
564382285	RPS6KC1	ribosomal protein S6 kinase C1	0.107	0.950	0.050
157818159	AAR2	AAR2 splicing factor	0.110	0.974	0.026
157786720	HIVEP1	HIVEP zinc finger 1	0.111	0.974	0.026
564360651	LRRC14	leucine rich repeat containing 14	0.115	0.999	0.001
47847438	EXOC3	exocyst complex component 3	0.118	0.997	0.003
68163425	TMEM199	transmembrane protein 199	0.118	0.953	0.047
18959272	KCNQ2	potassium voltage-gated channel subfamily Q member 2	0.119	0.964	0.036
293344794	FAM160B1	family with sequence similarity 160 member B1	0.121	0.996	0.004
672044181	HS2ST1	heparan sulfate 2-O-sulfotransferase 1	0.124	0.974	0.026
19705555	IPMK	inositol polyphosphate multikinase	0.126	0.950	0.050
58866022	MGAT4A	alpha-1,3-mannosyl-glycoprotein 4-beta-N-acetylglucosaminyltransferase A	0.127	0.986	0.014
672068548	SUPT6H	SPT6 homolog, histone chaperone and transcription elongation factor	0.127	0.965	0.035
60359978	KIF3C	kinesin family member 3C	0.131	0.990	0.010
58865976	KLHDC3	kelch domain containing 3	0.133	0.976	0.024
157823607	ALDH18A1	aldehyde dehydrogenase 18 family member A1	0.134	0.983	0.017
451770387	PRRT2	proline rich transmembrane protein 2	0.136	0.963	0.037

157821401	UQCC1	ubiquinol-cytochrome c reductase complex assembly factor 1	0.136	0.951	0.049
564398139	FYN	FYN proto-oncogene, Src family tyrosine kinase	0.137	0.994	0.006
672069253	SOCS7	suppressor of cytokine signaling 7	0.141	0.951	0.049
20301952	SLC2A1	solute carrier family 2 member 1	0.142	0.990	0.010
451172120	DUSP7	dual specificity phosphatase 7	0.142	0.953	0.047
74139306	TMED9	transmembrane p24 trafficking protein 9	0.143	0.953	0.047
672024670	INSYN2B	inhibitory synaptic factor family member 2B	0.145	0.973	0.027
149064388	Hmgxb3	HMG-box containing 3	0.147	0.952	0.048
148665617	NAA50	N-alpha-acetyltransferase 50, NatE catalytic subunit	0.149	0.975	0.025
157821581	PSMD13	proteasome 26S subunit, non-ATPase 13	0.150	0.965	0.035
564311031	CLPP	caseinolytic mitochondrial matrix peptidase proteolytic subunit	0.150	0.999	0.001
208973276	TMEM185A	transmembrane protein 185A	0.151	0.960	0.040
300253233	LEMD3	LEM domain containing 3	0.154	0.985	0.015
209863130	SEMA3F	semaphorin 3F	0.155	0.960	0.040
50510427	IP6K1	inositol hexakisphosphate kinase 1	0.156	0.967	0.033
77917548	DUS3L	dihydrouridine synthase 3 like	0.158	0.950	0.050
76559935	TUT1	terminal uridylyl transferase 1, U6 snRNA-specific	0.160	0.968	0.032
157819421	CEP97	centrosomal protein 97	0.160	0.975	0.025
404312665	DKK3	dickkopf WNT signaling pathway inhibitor 3	0.160	0.998	0.002
213688411	LPCAT1	lysophosphatidylcholine acyltransferase 1	0.162	0.997	0.003
149033480	Zfp956	zinc finger protein 956	0.163	0.974	0.026
672067460	RGD1560464	similar to hypothetical protein FLJ38426	0.164	0.970	0.030
157822501	MCM3AP	minichromosome maintenance complex component 3 associated protein	0.164	0.989	0.011
564340867	MMADHC	metabolism of cobalamin associated D	0.165	0.982	0.018
76559929	NOC2L	NOC2 like nucleolar associated transcriptional repressor	0.165	0.978	0.022
403225023	BRAP	BRCA1 associated protein	0.166	0.960	0.040
408772026	Afg3l1	AFG3-like AAA ATPase 1	0.166	0.984	0.016
55926133	RFC2	replication factor C subunit 2	0.168	0.997	0.003
422398900	CREBZF	CREB/ATF bZIP transcription factor	0.169	0.982	0.018

157823165	DNAJB1	DnaJ heat shock protein family (Hsp40) member B1	0.171	0.992	0.008
33356154	UBE2H	ubiquitin conjugating enzyme E2 H	0.172	0.968	0.032
56605790	HCFC2	host cell factor C2	0.173	0.974	0.026
119569672	BUB3	BUB3 mitotic checkpoint protein	0.176	0.982	0.018
77695933	NELL2	neural EGFL like 2	0.180	0.955	0.045
61556927	EIF3G	eukaryotic translation initiation factor 3 subunit G	0.180	0.996	0.004
62079005	SLAIN1	SLAIN motif family member 1	0.180	0.994	0.006
6981296	NUP50	nucleoporin 50	0.181	0.960	0.040
6981518	SDC1	syndecan 1	0.182	0.970	0.030
9507007	PTGFRN	prostaglandin F2 receptor inhibitor	0.184	0.989	0.011
11139303	JTB	jumping translocation breakpoint	0.185	0.950	0.050
564384443	EIF4ENIF1	eukaryotic translation initiation factor 4E nuclear import factor 1	0.187	1.000	0.000
66730335	SUMO3	small ubiquitin like modifier 3	0.188	0.994	0.006
149047323	ZNF518B	zinc finger protein 518B	0.190	0.958	0.042
672085227	USP10	ubiquitin specific peptidase 10	0.191	0.970	0.030
148683335	SLC25A44	solute carrier family 25 member 44	0.191	0.981	0.019
72004267	AKIRIN1	akirin 1	0.192	0.975	0.025
197313795	MTX1	metaxin 1	0.192	0.978	0.022
140971918	Hnrnpab	heterogeneous nuclear ribonucleoprotein A/B	0.194	0.972	0.028
296489017	BEND5	BEN domain containing 5	0.196	0.968	0.032
157820969	SBNO2	strawberry notch homolog 2	0.197	0.970	0.030
157820401	ABHD2	abhydrolase domain containing 2, acylglycerol lipase	0.197	0.976	0.024
57164107	NIPSNAP3A	nipsnap homolog 3A	0.199	0.962	0.038
213511844	ALG2	ALG2 alpha-1,3/1,6-mannosyltransferase	0.200	0.983	0.017
81295375	SLC35B2	solute carrier family 35 member B2	0.200	0.954	0.046
77797839	UBXN1	UBX domain protein 1	0.202	0.959	0.041
14277700	RPS12	ribosomal protein S12	0.203	0.972	0.028
219277692	NDUFB2	NADH:ubiquinone oxidoreductase subunit B2	0.203	0.984	0.016
564384353	SH3BP2	SH3 domain binding protein 2	0.204	0.976	0.024
157817674	ATP5MF-PTCD1	ATP5MF-PTCD1 readthrough	0.207	0.978	0.022
40018540	DDX24	DEAD-box helicase 24	0.208	0.972	0.028
61557082	TERF2IP	TERF2 interacting protein	0.211	0.990	0.010
61556748	TSPYL1	TSPY like 1	0.211	0.951	0.049
37359818	KCTD5	potassium channel tetramerization domain containing 5	0.212	0.951	0.049
310616720	DHX37	DEAH-box helicase 37	0.212	0.968	0.032

58865624	NUF2	NUF2 component of NDC80 kinetochore complex	0.214	0.954	0.046
24025618	DAB1	DAB adaptor protein 1	0.217	0.958	0.042
213688373	GADD45GIP1	GADD45G interacting protein 1	0.218	0.973	0.027
24638440	RIMS4	regulating synaptic membrane exocytosis 4	0.220	0.988	0.012
148669751	SMNDC1	survival motor neuron domain containing 1	0.223	0.961	0.039
70794793	MAP2K7	mitogen-activated protein kinase kinase 7	0.228	0.970	0.030
58865962	RNF41	ring finger protein 41	0.231	0.997	0.003
148670791	ZFYVE1	zinc finger FYVE-type containing 1	0.232	0.990	0.010
70794766	MRPS25	mitochondrial ribosomal protein S25	0.235	0.984	0.016
564340133	GTF3C4	general transcription factor IIIC subunit 4	0.236	0.994	0.006
37360264	TRMT6	tRNA methyltransferase 6	0.236	0.993	0.007
76096328	COMMD9	COMM domain containing 9	0.238	0.960	0.040
71043628	OGFRL1	opioid growth factor receptor like 1	0.245	0.980	0.020
291042683	DCAF5	DDB1 and CUL4 associated factor 5	0.252	1.000	0.000
57164019	B4GALT3	beta-1,4-galactosyltransferase 3	0.256	0.994	0.006
71361655	MRPL12	mitochondrial ribosomal protein L12	0.257	0.953	0.047
77627757	IQUB	IQ motif and ubiquitin domain containing	0.257	0.967	0.033
119388826	TFPT	TCF3 fusion partner	0.258	0.984	0.016
40254721	AMIGO2	adhesion molecule with Ig like domain 2	0.264	0.979	0.021
404312698	GOLM2	golgi membrane protein 2	0.270	0.970	0.030
564394925	TENT4B	terminal nucleotidyltransferase 4B	0.276	0.964	0.036
402794103	ATG101	autophagy related 101	0.279	0.970	0.030
59937915	ARIH2	ariadne RBR E3 ubiquitin protein ligase 2	0.280	0.987	0.013
157821915	MSANTD3	Myb/SANT DNA binding domain containing 3	0.283	0.971	0.029
148679437	HAS3	hyaluronan synthase 3	0.284	0.980	0.020
62078733	MAK16	MAK16 homolog	0.287	0.976	0.024
672020915	VCPKMT	valosin containing protein lysine methyltransferase	0.288	0.987	0.013
672053062	FKBP15	FKBP prolyl isomerase 15	0.290	0.988	0.012
51948506	AK8	adenylate kinase 8	0.290	0.990	0.010
76559919	N4BP3	NEDD4 binding protein 3	0.292	0.967	0.033
34328151	TBR1	T-box brain transcription factor 1	0.295	0.959	0.041
157821125	COA7	cytochrome c oxidase assembly factor 7 (putative)	0.295	0.952	0.048
68163385	GPATCH4	G-patch domain containing 4	0.297	0.979	0.021

157821997	MED28	mediator complex subunit 28	0.297	0.999	0.001
74220037	FAM107B	family with sequence similarity 107 member B	0.299	0.991	0.009
157822367	PUS3	pseudouridine synthase 3	0.301	0.999	0.001
29789082	COIL	coilin	0.305	0.971	0.029
300798436	NME6	NME/NM23 nucleoside diphosphate kinase 6	0.308	0.983	0.017
51491900	TOR1A	torsin family 1 member A	0.308	0.964	0.036
349501022	2410002F23Rik	RIKEN cDNA 2410002F23 gene	0.309	0.996	0.004
62078923	DZIP1L	DAZ interacting zinc finger protein 1 like	0.315	0.986	0.014
300793780	ZNF251	zinc finger protein 251	0.321	0.968	0.032
672030183	H2AC12	H2A clustered histone 12	0.323	0.963	0.037
68163537	NXPE4	neurexophilin and PC-esterase domain family member 4	0.329	0.996	0.004
564333920	PPRC1	PPARG related coactivator 1	0.333	0.959	0.041
564303955	EMX1	empty spiracles homeobox 1	0.335	0.971	0.029
564350836	MELK	maternal embryonic leucine zipper kinase	0.341	0.961	0.039
73990974	LZTS3	leucine zipper tumor suppressor family member 3	0.345	0.989	0.011
564365330	CDC25A	cell division cycle 25A	0.348	0.952	0.048
281604129	HELQ	helicase, POLQ like	0.354	0.985	0.015
148673748	FAM110B	family with sequence similarity 110 member B	0.357	0.959	0.041
392342123	ALS2CL	ALS2 C-terminal like	0.360	0.981	0.019
37360398	ISLR2	immunoglobulin superfamily containing leucine rich repeat 2	0.360	0.951	0.049
157822027	CSRNP2	cysteine and serine rich nuclear protein 2	0.361	0.969	0.031
77627740	ING3	inhibitor of growth family member 3	0.363	0.967	0.033
300797828	KAT14	lysine acetyltransferase 14	0.365	0.965	0.035
406362836	HS6ST3	heparan sulfate 6-O-sulfotransferase 3	0.365	0.979	0.021
148710078	TAF5	TATA-box binding protein associated factor 5	0.366	0.984	0.016
157817720	SLC16A14	solute carrier family 16 member 14	0.374	0.956	0.044
164565364	ITPKB	inositol-trisphosphate 3-kinase B	0.380	0.975	0.025
564352668	MYCL	MYCL proto-oncogene, bHLH transcription factor	0.392	0.968	0.032
157821403	RASSF7	Ras association domain family member 7	0.399	0.963	0.037
16758238	SPA17	sperm autoantigenic protein 17	0.402	0.969	0.031
23097354	FADD	Fas associated via death domain	0.403	0.967	0.033

226371633	CABLES1	Cdk5 and Abl enzyme substrate 1	0.409	0.998	0.002
66730347	PTPRCAP	protein tyrosine phosphatase receptor type C associated protein	0.415	0.997	0.003
293348214	CCDC88C	coiled-coil domain containing 88C	0.421	0.994	0.006
56090305	NFATC2IP	nuclear factor of activated T cells 2 interacting protein	0.430	0.989	0.011
672031975	LOC299312	similar to G protein-binding protein CRFG	0.436	0.963	0.037
157823891	ING2	inhibitor of growth family member 2	0.439	0.998	0.002
893846521	MARCHF11	membrane associated ring-CH-type finger 11	0.445	0.958	0.042
157820727	RPL27A	ribosomal protein L27a	0.452	0.992	0.008
53850630	LOC100362724/ MGC95208	similar to 4930453N24Rik protein	0.456	0.950	0.050
148706598	PKDCC	protein kinase domain containing, cytoplasmic	0.469	0.978	0.022
51948492	NUDT19	nudix hydrolase 19	0.470	0.954	0.046
70608121	Dmrtc1a	DMRT-like family C1a	0.479	0.980	0.020
149024753	DFFB	DNA fragmentation factor subunit beta	0.487	0.984	0.016
564347830	ZXDC	ZXD family zinc finger C	0.490	0.979	0.021
212549645	KIF18A	kinesin family member 18A	0.505	0.995	0.005
19424300	GCHFR	GTP cyclohydrolase I feedback regulator	0.519	0.997	0.003
148687591	TMEM132D	transmembrane protein 132D	0.550	0.992	0.008
219879771	PGAP3	post-GPI attachment to proteins phospholipase 3	0.560	0.957	0.043
149023178	CEP152	centrosomal protein 152	0.572	0.984	0.016
564372912	GPS2	G protein pathway suppressor 2	0.577	0.981	0.019
148664537	Gm10269	ribosomal protein L35 pseudogene	0.582	0.973	0.027
555290059	MED7	mediator complex subunit 7	0.584	0.953	0.047
148705473	FAM53A	family with sequence similarity 53 member A	0.585	0.954	0.046
22122541	LRRC3B	leucine rich repeat containing 3B	0.615	0.968	0.032
157822359	PELI2	pellino E3 ubiquitin protein ligase family member 2	0.620	0.964	0.036
38016150	QRFPR	pyroglutamylated RFamide peptide receptor	0.625	0.956	0.044
197386066	ZNF784	zinc finger protein 784	0.633	0.966	0.034
157823803	DOK3	docking protein 3	0.639	0.990	0.010
149025186	RPS6KL1	ribosomal protein S6 kinase like 1	0.661	0.957	0.043
256220048	PCDHGC5	protocadherin gamma subfamily C, 5	0.666	0.969	0.031
149016574	ZNF324	zinc finger protein 324	0.689	0.997	0.003
112984482	SBSN	suprabasin	0.700	0.959	0.041

392354293	Hmgb3	high mobility group box 3	0.709	0.965	0.035
672070295	BAHCC1	BAH domain and coiled-coil containing 1	0.769	0.959	0.041
672031995	Kdm6a	lysine demethylase 6A	0.794	0.978	0.022
564377118	WDR53	WD repeat domain 53	0.807	0.963	0.037
672086719	FAM184A	family with sequence similarity 184 member A	0.836	0.975	0.025
26024223	ABCG5	ATP binding cassette subfamily G member 5	0.841	0.958	0.042
149030324	CHRNA2	cholinergic receptor nicotinic alpha 2 subunit	0.994	0.976	0.024
82654234	LILRA6	leukocyte immunoglobulin like receptor A6	1.000	0.969	0.031
157817241	ISCA2	iron-sulfur cluster assembly 2	1.006	0.964	0.036
148674299	Gm14176	ubiquitin-conjugating enzyme E2I pseudogene	1.041	0.980	0.020
149067796	TMEM219	transmembrane protein 219	1.064	0.996	0.004
188536090	FAM241B	family with sequence similarity 241 member B	1.127	0.953	0.047
62078917	PAQR5	progestin and adipoQ receptor family member 5	1.181	0.956	0.044
6978525	FASLG	Fas ligand	1.186	0.979	0.021
149045964	PTH2R	parathyroid hormone 2 receptor	1.222	0.968	0.032
300793935	GSX1	GS homeobox 1	1.225	0.978	0.022
124487463	GPR161	G protein-coupled receptor 161	1.523	0.961	0.039
24308466	ITGB3	integrin subunit beta 3	1.596	0.970	0.030
575403049	ERBIN	erbb2 interacting protein	1.596	0.983	0.017
157818463	Zfp93	zinc finger protein 93	1.597	0.993	0.007
672013187	DMWD	DM1 locus, WD repeat containing	1.605	0.990	0.010
157816947	GUCA1B	guanylate cyclase activator 1B	1.708	0.983	0.017
149032888	LOC100910237	uncharacterized LOC100910237	1.751	0.971	0.029
157820135	CHRDL2	chordin like 2	1.762	0.969	0.031
392342449	PRSS56	serine protease 56	1.848	0.997	0.003
189181736	LAD1	ladinin 1	1.874	0.994	0.006
16758254	CNGA1	cyclic nucleotide gated channel subunit alpha 1	1.874	0.997	0.003
51243038	LY6G6D	lymphocyte antigen 6 family member G6D	2.000	0.951	0.049
568979594	SYT16	synaptotagmin 16	2.059	1.000	0.000
672070295	BAHCC1	BAH domain and coiled-coil containing 1	2.083	0.981	0.019
28972866	CSMD3	CUB and Sushi multiple domains 3	2.140	0.965	0.035
568974167	SLC26A11	solute carrier family 26 member 11	2.149	0.980	0.020

564297852	CRTC3	CREB regulated transcription coactivator 3	2.151	0.992	0.008
293352381	PAN3	poly(A) specific ribonuclease subunit PAN3	2.239	0.983	0.017
56912237	KRT28	keratin 28	2.241	0.966	0.034
28174920	RPL17	ribosomal protein L17	3.389	0.962	0.038

Supplementary Table S16. The list of genes that are differentially expressed in the offspring hippocampus in response to prenatal BPA exposure that exhibited the changes in the expression levels correlated with the number of primary neurites of primary hippocampal cells at DIV7. The transcriptome profiling data of DEGs in male and female rat offspring prenatally exposed to BPA (n = 6, male pups n = 3 and female pups n = 3, from independent litters) or the vehicle control (n = 6, male pups n = 3 and female pups n = 3, from independent litters) were obtained and used for the PTM analyses to identify DEGs that exhibited the changes in the expression levels correlated with the number of primary neurites of primary hippocampal cells at DIV7.

ID	Symbol	Entrez Gene Name	log2(FC)	R values	P-values
62078965	SLC47A1	solute carrier family 47 member 1	-3.135	-0.956	0.044
13928714	Ccl2	chemokine (C-C motif) ligand 2	-2.497	-0.951	0.049
148669850	GFRA1	GDNF family receptor alpha 1	-2.118	-0.997	0.003
156119589	FOXC2	forkhead box C2	-2.000	-0.981	0.019
157820583	ANKRD34C	ankyrin repeat domain 34C	-1.939	-0.974	0.026
157818655	MPZL2	myelin protein zero like 2	-1.922	-0.997	0.003
564380050	2410141K09Rik	RIKEN cDNA 2410141K09 gene	-1.920	-0.996	0.004
58000421	Ggnbp1	gametogenetin binding protein 1	-1.918	-0.979	0.021
157823875	EPS8L1	EPS8 like 1	-1.907	-0.961	0.039
454526968	PDE4C	phosphodiesterase 4C	-1.874	-0.986	0.014
16758014	HPX	hemopexin	-1.830	-0.984	0.016
300797073	CCDC27	coiled-coil domain containing 27	-1.778	-0.982	0.018
57222306	Oas1f	2'-5' oligoadenylate synthetase 1F	-1.755	-0.986	0.014
817473312	GATA6	GATA binding protein 6	-1.678	-0.977	0.023
157822811	Fmo9	flavin containing monooxygenase 9	-1.678	-0.977	0.023
117647198	CFD	complement factor D	-1.585	-0.978	0.022
71896590	AOC3	amine oxidase copper containing 3	-1.585	-0.970	0.030
392332008	SLC38A10	solute carrier family 38 member 10	-1.568	-0.979	0.021
13929066	CPZ	carboxypeptidase Z	-1.549	-0.957	0.043
21426773	ASPG	asparaginase	-1.524	-0.974	0.026
157819105	C2orf73	chromosome 2 open reading frame 73	-1.515	-0.996	0.004
157821867	BMP8A	bone morphogenetic protein 8a	-1.509	-0.996	0.004
74202463	EYA2	EYA transcriptional coactivator and phosphatase 2	-1.497	-0.986	0.014
194474002	MEI1	meiotic double-stranded break formation protein 1	-1.476	-0.976	0.024
162138928	SLC13A3	solute carrier family 13 member 3	-1.467	-0.981	0.019
16758434	DAO	D-amino acid oxidase	-1.459	-0.997	0.003
18677739	CDKN2B	cyclin dependent kinase inhibitor 2B	-1.454	-0.966	0.034
157820117	FBXL8	F-box and leucine rich repeat protein 8	-1.441	-0.997	0.003
6981148	LEP	leptin	-1.436	-0.962	0.038

157818369	Hils1	histone H1-like protein in spermatids 1	-1.411	-0.952	0.048
672042718	ZBBX	zinc finger B-box domain containing	-1.409	-0.994	0.006
564314671	VPS8	VPS8 subunit of CORVET complex	-1.386	-0.966	0.034
149065466	ARHGEF5	Rho guanine nucleotide exchange factor 5	-1.382	-0.987	0.013
157822063	Gm6377	predicted gene 6377	-1.379	-0.995	0.005
48675870	PPP1R3B	protein phosphatase 1 regulatory subunit 3B	-1.379	-0.970	0.030
827012496	NLRC4	NLR family CARD domain containing 4	-1.346	-0.976	0.024
58865654	EFEMP1	EGF containing fibulin extracellular matrix protein 1	-1.284	-0.954	0.046
6981068	ICAM1	intercellular adhesion molecule 1	-1.240	-0.953	0.047
8392900	RUNX1	RUNX family transcription factor 1	-1.222	-0.957	0.043
58865664	SH2D4A	SH2 domain containing 4A	-1.204	-0.967	0.033
157820725	SUN5	Sad1 and UNC84 domain containing 5	-1.187	-0.962	0.038
157820223	TBX4	T-box transcription factor 4	-1.138	-0.991	0.009
300798739	MYO3B	myosin IIIB	-1.100	-0.961	0.039
197385083	C1orf194	chromosome 1 open reading frame 194	-1.090	-0.978	0.022
9910378	CDC42SE2	CDC42 small effector 2	-1.069	-0.960	0.040
7106240	AKR7A3	aldo-keto reductase family 7 member A3	-1.026	-0.991	0.009
297206838	ARNTL2	aryl hydrocarbon receptor nuclear translocator like 2	-1.020	-0.990	0.010
58865898	LIMS2	LIM zinc finger domain containing 2	-1.018	-0.966	0.034
62078849	USP18	ubiquitin specific peptidase 18	-1.000	-0.962	0.038
197384591	CYTL1	cytokine like 1	-1.000	-0.995	0.005
68163517	CCDC146	coiled-coil domain containing 146	-0.971	-0.972	0.028
157819493	Igbp1b	immunoglobulin (CD79A) binding protein 1b	-0.966	-0.986	0.014
148680846	HIC1	HIC ZBTB transcriptional repressor 1	-0.940	-0.966	0.034
157823079	RBKS	ribokinase	-0.939	-0.976	0.024
6981176	MAK	male germ cell associated kinase	-0.931	-0.995	0.005
564377419	THPO	thrombopoietin	-0.882	-0.996	0.004
564321163	CHD9	chromodomain helicase DNA binding protein 9	-0.868	-0.998	0.002
201861483	LOC102548396 (includes others)	zinc finger protein 951	-0.848	-0.986	0.014
564366187	LOC100361039 (includes others)	similar to nidogen 2	-0.833	-0.990	0.010
564396113	ZCCHC14	zinc finger CCHC-type containing 14	-0.797	-0.980	0.020

157821153	ECHDC2	enoyl-CoA hydratase domain containing 2	-0.796	-0.986	0.014
109480098	SMARCC2	SWI/SNF related, matrix associated, actin dependent regulator of chromatin subfamily c member 2	-0.795	-0.986	0.014
67846074	EHD2	EH domain containing 2	-0.782	-0.997	0.003
392352101	LRCH3	leucine rich repeats and calponin homology domain containing 3	-0.773	-0.965	0.035
197385174	ENO4	enolase 4	-0.766	-0.962	0.038
148664646	GYPC	glycophorin C (Gerbich blood group)	-0.760	-0.964	0.036
13929084	THBD	thrombomodulin	-0.732	-0.989	0.011
68163370	CARNMT1	carnosine N-methyltransferase 1	-0.724	-0.969	0.031
402534539	ECRG4	ECRG4 augurin precursor	-0.723	-0.986	0.014
40018618	CBX7	chromobox 7	-0.698	-0.987	0.013
401461786	CP	ceruloplasmin	-0.695	-0.955	0.045
564393107	STING1	stimulator of interferon response cGAMP interactor 1	-0.693	-0.998	0.002
114145782	MORN5	MORN repeat containing 5	-0.657	-0.967	0.033
300794803	SYNPO2	synaptopodin 2	-0.655	-0.973	0.027
82617598	SLC5A3	solute carrier family 5 member 3	-0.652	-0.968	0.032
119226202	CDC42EP1	CDC42 effector protein 1	-0.651	-0.951	0.049
47059173	IER3	immediate early response 3	-0.649	-0.990	0.010
260593702	Slc26a10	solute carrier family 26, member 10	-0.646	-0.995	0.005
157816915	ZNF438	zinc finger protein 438	-0.643	-0.965	0.035
13591940	DPYD	dihydropyrimidine dehydrogenase	-0.642	-0.996	0.004
25742776	MC4R	melanocortin 4 receptor	-0.641	-0.979	0.021
158508544	DDR2	discoidin domain receptor tyrosine kinase 2	-0.640	-0.975	0.025
71043760	RRM2	ribonucleotide reductase regulatory subunit M2	-0.637	-0.986	0.014
672060362	ELFN2	extracellular leucine rich repeat and fibronectin type III domain containing 2	-0.636	-0.955	0.045
19424350	GBP2	guanylate binding protein 2	-0.635	-0.976	0.024
58865948	CREB3L2	cAMP responsive element binding protein 3 like 2	-0.634	-0.956	0.044
1763306	UNC13C	unc-13 homolog C	-0.626	-0.994	0.006
16758284	SLC5A7	solute carrier family 5 member 7	-0.618	-0.971	0.029
29789038	BMP6	bone morphogenetic protein 6	-0.600	-0.962	0.038
564387543	UGGT2	UDP-glucose glycoprotein glucosyltransferase 2	-0.596	-0.950	0.050
157817670	SLC2A10	solute carrier family 2 member 10	-0.595	-0.985	0.015
164519095	SLC9A2	solute carrier family 9 member A2	-0.578	-0.961	0.039
8393469	S1PR2	sphingosine-1-phosphate receptor 2	-0.561	-0.983	0.017

569009290	TENM1	teneurin transmembrane protein 1	-0.559	-0.971	0.029
197313645	SMTN	smoothelin	-0.558	-0.980	0.020
42476287	TGM2	transglutaminase 2	-0.553	-0.994	0.006
300795183	SNTG1	syntrophin gamma 1	-0.543	-0.965	0.035
6754808	NDP	norrin cystine knot growth factor NDP	-0.538	-0.975	0.025
20302097	PIGL	phosphatidylinositol glycan anchor biosynthesis class L	-0.534	-0.976	0.024
569012000	KLF8	Kruppel like factor 8	-0.531	-0.960	0.040
300794555	TMC7	transmembrane channel like 7	-0.531	-0.998	0.002
672051145	PARP11	poly(ADP-ribose) polymerase family member 11	-0.529	-0.992	0.008
157820317	STXBP4	syntaxin binding protein 4	-0.524	-0.976	0.024
148678784	PTHLH	parathyroid hormone like hormone	-0.523	-0.996	0.004
672071899	BHLHA15	basic helix-loop-helix family member a15	-0.522	-0.959	0.041
124244050	PPIP5K1	diphosphoinositol pentakisphosphate kinase 1	-0.522	-0.981	0.019
392338379	SLC26A8	solute carrier family 26 member 8	-0.514	-0.992	0.008
312922352	TTF2	transcription termination factor 2	-0.506	-0.967	0.033
564399060	PIGA	phosphatidylinositol glycan anchor biosynthesis class A	-0.505	-0.976	0.024
37693510	Bst2	bone marrow stromal cell antigen 2	-0.500	-0.986	0.014
209529675	TXLNB	taxilin beta	-0.498	-0.952	0.048
62078635	CCDC153	coiled-coil domain containing 153	-0.494	-0.953	0.047
149058686	PIGR	polymeric immunoglobulin receptor	-0.485	-0.964	0.036
281427229	COL6A2	collagen type VI alpha 2 chain	-0.485	-0.998	0.002
13591971	HNMT	histamine N-methyltransferase	-0.481	-0.999	0.001
62078713	ZNF385D	zinc finger protein 385D	-0.476	-0.998	0.002
148695091	BBS5	Bardet-Biedl syndrome 5	-0.473	-0.998	0.002
312922379	TNN	tenascin N	-0.469	-0.984	0.016
19924069	SPON2	spondin 2	-0.467	-0.982	0.018
564380929	KCNT2	potassium sodium-activated channel subfamily T member 2	-0.465	-0.997	0.003
9506953	PCOLCE	procollagen C-endopeptidase enhancer	-0.458	-0.970	0.030
148699893	COL6A1	collagen type VI alpha 1 chain	-0.457	-0.967	0.033
157819513	ABCA4	ATP binding cassette subfamily A member 4	-0.453	-0.997	0.003
157820241	Marveld1	MARVEL domain containing 1	-0.452	-0.992	0.008
201066407	EAPP	E2F associated phosphoprotein	-0.444	-0.978	0.022
27436863	HACL1	2-hydroxyacyl-CoA lyase 1	-0.441	-0.958	0.042
8393057	SERPINH1	serpin family H member 1	-0.436	-0.991	0.009

172045714	MIIP	migration and invasion inhibitory protein	-0.433	-0.951	0.049
157822319	EVC2	EvC ciliary complex subunit 2	-0.428	-0.961	0.039
149038013	SLC9A5	solute carrier family 9 member A5	-0.426	-0.960	0.040
1346731	HAPLN1	hyaluronan and proteoglycan link protein 1	-0.423	-0.999	0.001
157823279	CGNL1	cingulin like 1	-0.418	-0.981	0.019
74218228	HNRNPC	heterogeneous nuclear ribonucleoprotein C	-0.418	-0.976	0.024
564329859	COA4	cytochrome c oxidase assembly factor 4 homolog	-0.417	-0.997	0.003
83642834	NAGK	N-acetylglucosamine kinase	-0.416	-0.990	0.010
68342019	LRRC17	leucine rich repeat containing 17	-0.413	-0.958	0.042
672041704	NIPBL*	NIPBL cohesin loading factor	-0.412	-0.964	0.036
672085293	FANCA	FA complementation group A	-0.412	-0.990	0.010
23463307	RIOX2	ribosomal oxygenase 2	-0.409	-0.988	0.012
157786690	PRKCA	protein kinase C alpha	-0.408	-0.969	0.031
46402488	NOS3	nitric oxide synthase 3	-0.405	-0.950	0.050
7949020	CDK2	cyclin dependent kinase 2	-0.405	-0.969	0.031
672070295	BAHCC1*	BAH domain and coiled-coil containing 1	-0.404	-0.985	0.015
127140886	EML6	EMAP like 6	-0.403	-0.950	0.050
157819163	SYPL2	synaptophysin like 2	-0.400	-0.995	0.005
77695926	STAT1	signal transducer and activator of transcription 1	-0.399	-0.980	0.020
564342244	NUTM1	NUT midline carcinoma family member 1	-0.390	-0.990	0.010
157823193	LOXL3	lysyl oxidase like 3	-0.387	-0.957	0.043
569000267	MDC1	mediator of DNA damage checkpoint 1	-0.384	-0.952	0.048
564305413	E130308A19Rik	RIKEN cDNA E130308A19 gene	-0.381	-0.965	0.035
18426846	DCBLD2	discoidin, CUB and LCCL domain containing 2	-0.378	-0.972	0.028
157823373	TRHDE	thyrotropin releasing hormone degrading enzyme	-0.373	-0.999	0.001
157819959	PCDHB2	protocadherin beta 2	-0.369	-0.955	0.045
157819753	RCN1	reticulocalbin 1	-0.368	-0.998	0.002
9247217	MSX1	msh homeobox 1	-0.368	-0.976	0.024
53734563	ACCS	1-aminocyclopropane-1-carboxylate synthase homolog (inactive)	-0.367	-0.997	0.003
157822461	C20orf194	chromosome 20 open reading frame 194	-0.366	-0.982	0.018
281604225	PUS7	pseudouridine synthase 7	-0.362	-0.952	0.048

61556907	GALT	galactose-1-phosphate uridylyltransferase	-0.360	-0.990	0.010
157820839	Dnajb3	DnaJ heat shock protein family (Hsp40) member B3	-0.360	-0.964	0.036
29293811	SERPINF1	serpin family F member 1	-0.359	-0.967	0.033
25453410	CACNA1B	calcium voltage-gated channel subunit alpha1 B	-0.357	-0.953	0.047
384368019	Snhg11	small nucleolar RNA host gene 11	-0.356	-0.985	0.015
78042613	NICN1	nicolin 1	-0.354	-0.987	0.013
594191048	C19orf54	chromosome 19 open reading frame 54	-0.352	-0.955	0.045
61557118	PCGF6	polycomb group ring finger 6	-0.351	-0.967	0.033
149023022	Oip5	Opa interacting protein 5	-0.350	-0.976	0.024
149053793	TSPOAP1	TSPO associated protein 1	-0.349	-0.976	0.024
148747194	SLC16A7	solute carrier family 16 member 7	-0.349	-0.976	0.024
564329392	FLNA	filamin A	-0.347	-0.979	0.021
56119120	SNF8	SNF8 subunit of ESCRT-II	-0.343	-0.952	0.048
81158091	PCDHGA9	protocadherin gamma subfamily A, 9	-0.342	-0.952	0.048
71361669	CIT	citron rho-interacting serine/threonine kinase	-0.336	-0.975	0.025
672081597	AGTR1	angiotensin II receptor type 1	-0.336	-0.996	0.004
148680122	UNC5C	unc-5 netrin receptor C	-0.335	-0.997	0.003
157819569	TEAD2	TEA domain transcription factor 2	-0.333	-0.994	0.006
157824146	ITGA5	integrin subunit alpha 5	-0.329	-0.975	0.025
77628027	PSMC3IP	PSMC3 interacting protein	-0.328	-0.964	0.036
672043651	TENT5C	terminal nucleotidyltransferase 5C	-0.324	-0.973	0.027
50510855	RIMKLB	ribosomal modification protein rimK like family member B	-0.320	-0.953	0.047
672033554	LOC102557335	uncharacterized LOC102557335	-0.319	-0.957	0.043
564304076	FGD5	FYVE, RhoGEF and PH domain containing 5	-0.319	-0.983	0.017
18426850	LCP2	lymphocyte cytosolic protein 2	-0.318	-0.965	0.035
672050244	APLF	aprataxin and PNKP like factor	-0.318	-0.972	0.028
148702301	CYB561	cytochrome b561	-0.317	-0.984	0.016
157823879	NUDT12	nudix hydrolase 12	-0.317	-0.972	0.028
157819457	MAP3K14	mitogen-activated protein kinase kinase kinase 14	-0.317	-0.961	0.039
564318054	R3hcc1	R3H domain and coiled-coil containing 1	-0.316	-0.960	0.040
61557127	NNT	nicotinamide nucleotide transhydrogenase	-0.313	-0.996	0.004
672084625	LOC100909409 (includes others)	RGD1562660	-0.312	-0.992	0.008
68163523	TTC26	tetratricopeptide repeat domain 26	-0.311	-0.971	0.029

109484871	HERC1	HECT and RLD domain containing E3 ubiquitin protein ligase family member 1	-0.310	-0.995	0.005
197313632	FRZB	frizzled related protein	-0.308	-0.955	0.045
966975500	MMP17	matrix metallopeptidase 17	-0.306	-0.952	0.048
399124777	GLS2	glutaminase 2	-0.305	-0.968	0.032
149023323	GFRA4	GDNF family receptor alpha 4	-0.303	-0.998	0.002
564352420	MKNK1	MAPK interacting serine/threonine kinase 1	-0.302	-0.953	0.047
124107592	MYO1C	myosin IC	-0.298	-0.998	0.002
404247454	COL26A1	collagen type XXVI alpha 1 chain	-0.295	-0.967	0.033
27545388	ABCA5	ATP binding cassette subfamily A member 5	-0.291	-0.986	0.014
12844128	CCDC90B	coiled-coil domain containing 90B	-0.288	-0.954	0.046
157820517	CARD6	caspase recruitment domain family member 6	-0.287	-0.978	0.022
564329612	ME3	malic enzyme 3	-0.284	-0.985	0.015
392338478	TTC37	tetratricopeptide repeat domain 37	-0.283	-0.991	0.009
564367076	Mocs1	molybdenum cofactor synthesis 1	-0.281	-0.992	0.008
226874871	OMG	oligodendrocyte myelin glycoprotein	-0.274	-0.988	0.012
18959266	KHDRBS2	KH RNA binding domain containing, signal transduction associated 2	-0.274	-0.963	0.037
157822627	PLXDC2	plexin domain containing 2	-0.273	-0.990	0.010
157817953	RPGRIP1L	RPGRIP1 like	-0.269	-0.974	0.026
219804406	DOCK1	dedicator of cytokinesis 1	-0.269	-0.998	0.002
25742783	PLK1	polo like kinase 1	-0.269	-0.995	0.005
149016965	GRB10	growth factor receptor bound protein 10	-0.268	-0.983	0.017
928136440	SRRT	serrate, RNA effector molecule	-0.268	-0.980	0.020
149040074	FAM107A	family with sequence similarity 107 member A	-0.267	-0.996	0.004
149017535	HDAC10	histone deacetylase 10	-0.267	-0.987	0.013
13786142	SLIT3	slit guidance ligand 3	-0.266	-0.962	0.038
219275534	VPS13A	vacuolar protein sorting 13 homolog A	-0.264	-0.985	0.015
149020634	TAF1D	TATA-box binding protein associated factor, RNA polymerase I subunit D	-0.262	-0.996	0.004
564335541	Cplane1	ciliogenesis and planar polarity effector 1	-0.262	-0.997	0.003
77627987	DARS2	aspartyl-tRNA synthetase 2, mitochondrial	-0.261	-0.960	0.040
13929166	CLIC4	chloride intracellular channel 4	-0.259	-0.958	0.042
149060525	FSTL1	follistatin like 1	-0.258	-0.974	0.026
157787147	TEK	TEK receptor tyrosine kinase	-0.258	-0.954	0.046

300795738	RASSF8	Ras association domain family member 8	-0.255	-0.960	0.040
140971205	GRIN2A	glutamate ionotropic receptor NMDA type subunit 2A	-0.255	-0.983	0.017
564344961	RTEL1	regulator of telomere elongation helicase 1	-0.254	-0.996	0.004
16758538	RASGRF2	Ras protein specific guanine nucleotide releasing factor 2	-0.254	-0.991	0.009
61097902	ABCC4	ATP binding cassette subfamily C member 4	-0.248	-0.993	0.007
296439269	PHF10	PHD finger protein 10	-0.245	-0.950	0.050
568974832	YBX2	Y-box binding protein 2	-0.245	-0.973	0.027
564391295	DUSP22	dual specificity phosphatase 22	-0.244	-0.986	0.014
13162287	DDT	D-dopachrome tautomerase	-0.243	-0.960	0.040
12018300	AKAP6	A-kinase anchoring protein 6	-0.242	-0.978	0.022
300793894	URB1	URB1 ribosome biogenesis homolog	-0.242	-0.987	0.013
309319799	EIF2AK4	eukaryotic translation initiation factor 2 alpha kinase 4	-0.239	-0.983	0.017
119310200	KDM4D	lysine demethylase 4D	-0.237	-0.975	0.025
291490673	NGFR	nerve growth factor receptor	-0.236	-0.965	0.035
48040475	GCNT2	glucosaminyl (N-acetyl) transferase 2 (I blood group)	-0.233	-0.953	0.047
62079099	ORC5	origin recognition complex subunit 5	-0.228	-0.995	0.005
54312088	ATP2B4	ATPase plasma membrane Ca2+ transporting 4	-0.228	-0.977	0.023
62079015	PRXL2A	peroxiredoxin like 2A	-0.226	-0.994	0.006
392348438	NIPAL3	NIPA like domain containing 3	-0.223	-0.964	0.036
56605770	RRP8	ribosomal RNA processing 8	-0.221	-0.973	0.027
296010823	UBR1	ubiquitin protein ligase E3 component n-recognin 1	-0.220	-0.958	0.042
158631207	YIF1A	Yip1 interacting factor homolog A, membrane trafficking protein	-0.220	-0.990	0.010
402766107	ALDH7A1	aldehyde dehydrogenase 7 family member A1	-0.220	-0.996	0.004
5174513	SMAD3	SMAD family member 3	-0.219	-0.996	0.004
62079019	UFSP2	UFM1 specific peptidase 2	-0.218	-0.980	0.020
564315812	NAV1	neuron navigator 1	-0.217	-1.000	0.000
348605146	HDAC11	histone deacetylase 11	-0.216	-0.962	0.038
564397593	RAB36	RAB36, member RAS oncogene family	-0.212	-0.960	0.040
149066531	VPS13B	vacuolar protein sorting 13 homolog B	-0.212	-0.993	0.007
158749540	NPEPPS	aminopeptidase puromycin sensitive	-0.210	-0.984	0.016
281306814	RPS6KA2	ribosomal protein S6 kinase A2	-0.210	-0.978	0.022

281371490	LAMC1	laminin subunit gamma 1	-0.209	-0.963	0.037
300798704	TLL2	tolloid like 2	-0.206	-0.997	0.003
155369301	ARHGEF37	Rho guanine nucleotide exchange factor 37	-0.206	-0.979	0.021
564372562	PFAS	phosphoribosylformylglycinamidine synthase	-0.206	-0.986	0.014
54019432	PCDHA7	protocadherin alpha 7	-0.205	-0.986	0.014
564343174	PLCB4	phospholipase C beta 4	-0.204	-0.993	0.007
157818191	SETD6	SET domain containing 6, protein lysine methyltransferase	-0.203	-0.975	0.025
13027430	WDR7	WD repeat domain 7	-0.202	-0.972	0.028
157786582	C16orf89	chromosome 16 open reading frame 89	-0.201	-0.978	0.022
38454284	PPM1E	protein phosphatase, Mg2+/Mn2+ dependent 1E	-0.201	-0.970	0.030
148707634	SHISA4	shisa family member 4	-0.201	-0.967	0.033
61556891	OSBPL2	oxysterol binding protein like 2	-0.198	-0.964	0.036
157818171	PLK4	polo like kinase 4	-0.188	-0.988	0.012
68163551	TBC1D22B	TBC1 domain family member 22B	-0.187	-0.982	0.018
219803038	PDE2A	phosphodiesterase 2A	-0.186	-0.952	0.048
157822539	ANK1	ankyrin 1	-0.184	-0.979	0.021
300796855	PARD3B	par-3 family cell polarity regulator beta	-0.183	-0.994	0.006
56605784	MRRF	mitochondrial ribosome recycling factor	-0.182	-0.999	0.001
84490431	DNM3	dynamin 3	-0.182	-0.993	0.007
672088357	ZCCHC18	zinc finger CCHC-type containing 18	-0.180	-0.981	0.019
9506957	PCSK7	proprotein convertase subtilisin/kexin type 7	-0.180	-0.971	0.029
13592087	SOAT1	sterol O-acyltransferase 1	-0.180	-0.999	0.001
293349000	ARHGAP42	Rho GTPase activating protein 42	-0.178	-0.960	0.040
564390898	KIF13A	kinesin family member 13A	-0.176	-0.960	0.040
149049332	TULP3	TUB like protein 3	-0.175	-0.997	0.003
392333710	COL4A2	collagen type IV alpha 2 chain	-0.173	-0.991	0.009
18034783	ABCC3	ATP binding cassette subfamily C member 3	-0.170	-0.980	0.020
62339281	ADAM9	ADAM metallopeptidase domain 9	-0.168	-0.993	0.007
817472062	CDK5RAP3	CDK5 regulatory subunit associated protein 3	-0.167	-0.958	0.042
20127390	RNF112	ring finger protein 112	-0.166	-0.968	0.032
6981076	IDE	insulin degrading enzyme	-0.165	-0.988	0.012
149046296	CREG2	cellular repressor of E1A stimulated genes 2	-0.165	-0.954	0.046
13242243	AXIN2	axin 2	-0.164	-0.974	0.026

149052635	TNIP1	TNFAIP3 interacting protein 1	-0.161	-0.987	0.013
61556993	HIBCH	3-hydroxyisobutyryl-CoA hydrolase	-0.158	-0.963	0.037
62078609	Dync2li1	dynein cytoplasmic 2 light intermediate chain 1	-0.157	-0.982	0.018
564299821	PARP8	poly(ADP-ribose) polymerase family member 8	-0.155	-0.963	0.037
564385664	FERMT2	fermitin family member 2	-0.154	-0.995	0.005
157817678	BUB1	BUB1 mitotic checkpoint serine/threonine kinase	-0.153	-0.998	0.002
76880459	THOC6	THO complex 6	-0.149	-0.954	0.046
717324516	SCN8A	sodium voltage-gated channel alpha subunit 8	-0.147	-0.973	0.027
672016955	MAP3K20	mitogen-activated protein kinase kinase kinase 20	-0.147	-0.978	0.022
157818033	ATG4C	autophagy related 4C cysteine peptidase	-0.146	-0.987	0.013
157820591	AVEN	apoptosis and caspase activation inhibitor	-0.146	-0.990	0.010
55742755	CTNNA1	catenin alpha 1	-0.146	-0.973	0.027
157820561	NAT10	N-acetyltransferase 10	-0.144	-0.999	0.001
730229363	RALGAPA1	Ral GTPase activating protein catalytic subunit alpha 1	-0.142	-0.961	0.039
56090241	THTPA	thiamine triphosphatase	-0.141	-0.964	0.036
6981166	PLAGL1	PLAG1 like zinc finger 1	-0.133	-0.969	0.031
9507177	USO1	USO1 vesicle transport factor	-0.130	-0.976	0.024
8394354	SQLE	squalene epoxidase	-0.129	-0.960	0.040
144445950	XRCC6	X-ray repair cross complementing 6	-0.129	-1.000	0.000
564382183	EPRS1	glutamyl-prolyl-tRNA synthetase 1	-0.126	-0.988	0.012
281604211	RAB3GAP2	RAB3 GTPase activating non-catalytic protein subunit 2	-0.123	-1.000	0.000
157817043	ACOT13	acyl-CoA thioesterase 13	-0.122	-0.960	0.040
564301782	CERS6	ceramide synthase 6	-0.122	-0.957	0.043
157822001	RACGAP1	Rac GTPase activating protein 1	-0.120	-0.985	0.015
476007242	EPS8	epidermal growth factor receptor pathway substrate 8	-0.117	-0.958	0.042
293339963	RAB11FIP3	RAB11 family interacting protein 3	-0.116	-0.972	0.028
201066352	ANKRD6	ankyrin repeat domain 6	-0.116	-0.982	0.018
29789269	GRIA1	glutamate ionotropic receptor AMPA type subunit 1	-0.115	-0.976	0.024
11560079	KIT	KIT proto-oncogene, receptor tyrosine kinase	-0.114	-0.954	0.046
60360272	KLHL5	kelch like family member 5	-0.113	-0.993	0.007
148698492	RIMKLA	ribosomal modification protein rimK like family member A	-0.112	-0.964	0.036

564353678	USP48	ubiquitin specific peptidase 48	-0.112	-0.953	0.047
694981804	CLDN5	claudin 5	-0.108	-0.972	0.028
184160976	PRMT5	protein arginine methyltransferase 5	-0.107	-0.996	0.004
61556863	BTBD9	BTB domain containing 9	-0.106	-0.983	0.017
564311487	LONRF2	LON peptidase N-terminal domain and ring finger 2	-0.106	-0.998	0.002
672066454	Mrtfb	myocardin related transcription factor B	-0.106	-0.988	0.012
282158057	ASTN1	astrotactin 1	-0.103	-0.977	0.023
10946604	SEC61A2	SEC61 translocon subunit alpha 2	-0.100	-0.997	0.003
157818589	TSPAN7	tetraspanin 7	-0.100	-0.981	0.019
300794843	IQGAP3	IQ motif containing GTPase activating protein 3	-0.100	-0.980	0.020
392353562	ATP8A2	ATPase phospholipid transporting 8A2	-0.099	-0.961	0.039
9507157	STRN	striatin	-0.094	-0.978	0.022
401461792	GOT1	glutamic-oxaloacetic transaminase 1	-0.094	-0.999	0.001
54035294	ADH5	alcohol dehydrogenase 5 (class III), chi polypeptide	-0.080	-0.982	0.018
148747375	CDS1	CDP-diacylglycerol synthase 1	-0.078	-0.997	0.003
390979616	ITGA3	integrin subunit alpha 3	-0.077	-0.977	0.023
568986834	KCNMA1	potassium calcium-activated channel subfamily M alpha 1	-0.075	-0.958	0.042
62657153	EFTUD2	elongation factor Tu GTP binding domain containing 2	-0.068	-0.996	0.004
52138624	SLC25A20	solute carrier family 25 member 20	-0.066	-0.978	0.022
77020248	PFKFB2	6-phosphofructo-2-kinase/fructose-2,6-biphosphatase 2	-0.060	-0.983	0.017
29789275	PDGFRB	platelet derived growth factor receptor beta	-0.059	-0.995	0.005
13027436	APAF1	apoptotic peptidase activating factor 1	-0.056	-0.992	0.008
672066031	DGKD	diacylglycerol kinase delta	0.044	0.985	0.015
157819577	SAP30BP	SAP30 binding protein	0.045	0.986	0.014
77627906	KLHDC2	kelch domain containing 2	0.045	0.999	0.001
45478098	CMTR1	cap methyltransferase 1	0.046	0.962	0.038
33468857	HINT1	histidine triad nucleotide binding protein 1	0.055	0.991	0.009
55250051	TXNRD1	thioredoxin reductase 1	0.056	0.994	0.006
148706586	TMEM178A	transmembrane protein 178A	0.057	0.982	0.018
58865796	PTDSS1	phosphatidylserine synthase 1	0.057	0.959	0.041
148697565	FAM83H	family with sequence similarity 83 member H	0.059	0.984	0.016
157819485	PLPPR5	phospholipid phosphatase related 5	0.064	0.957	0.043

25453374	PEX14	peroxisomal biogenesis factor 14	0.067	0.951	0.049
70778952	RAD23B	RAD23 homolog B, nucleotide excision repair protein	0.069	0.983	0.017
149042824	UBE2V1	ubiquitin conjugating enzyme E2 V1	0.070	0.983	0.017
8393746	MAP2K5	mitogen-activated protein kinase kinase 5	0.072	0.981	0.019
62078453	INPP5K	inositol polyphosphate-5-phosphatase K	0.077	0.993	0.007
755492511	MAP4K4	mitogen-activated protein kinase kinase kinase kinase 4	0.078	0.954	0.046
9507059	RNF5	ring finger protein 5	0.080	0.987	0.013
37360414	NPLOC4	NPL4 homolog, ubiquitin recognition factor	0.081	0.987	0.013
672074758	NCSTN	nicastrin	0.083	0.951	0.049
157819911	CSGALNACT2	chondroitin sulfate N-acetylgalactosaminyltransferase 2	0.088	0.968	0.032
672075032	MARK1	microtubule affinity regulating kinase 1	0.089	0.970	0.030
26346731	UBE2Z	ubiquitin conjugating enzyme E2 Z	0.091	0.987	0.013
564356795	ALKBH1	alkB homolog 1, histone H2A dioxygenase	0.094	0.999	0.001
209447030	DDX27	DEAD-box helicase 27	0.097	0.965	0.035
300797976	ANKRD50	ankyrin repeat domain 50	0.100	0.984	0.016
42476292	TALDO1	transaldolase 1	0.106	0.985	0.015
564382285	RPS6KC1	ribosomal protein S6 kinase C1	0.107	0.962	0.038
149063993	NEDD8	NEDD8 ubiquitin like modifier	0.109	0.982	0.018
47847438	EXOC3	exocyst complex component 3	0.118	0.959	0.041
149050200	GPR180	G protein-coupled receptor 180	0.120	0.960	0.040
58865512	STRAP	serine/threonine kinase receptor associated protein	0.120	0.976	0.024
672044181	HS2ST1	heparan sulfate 2-O-sulfotransferase 1	0.124	0.990	0.010
19705555	IPMK	inositol polyphosphate multikinase	0.126	0.999	0.001
209977101	TRPM4	transient receptor potential cation channel subfamily M member 4	0.127	0.985	0.015
157823607	ALDH18A1	aldehyde dehydrogenase 18 family member A1	0.134	0.970	0.030
13242322	ATF4	activating transcription factor 4	0.135	0.999	0.001
451770387	PRRT2	proline rich transmembrane protein 2	0.136	0.971	0.029
672069253	SOCS7	suppressor of cytokine signaling 7	0.141	0.997	0.003
451172120	DUSP7	dual specificity phosphatase 7	0.142	0.975	0.025
60360118	FAM168B	family with sequence similarity 168 member B	0.142	0.986	0.014
61555249	NUDT11	nudix hydrolase 11	0.142	0.953	0.047

157073939	LOC728392	uncharacterized LOC728392	0.143	0.993	0.007
148665617	NAA50	N-alpha-acetyltransferase 50, NatE catalytic subunit	0.149	0.985	0.015
82654218	AIMP2	aminoacyl tRNA synthetase complex interacting multifunctional protein 2	0.151	0.997	0.003
55741780	SPG21	SPG21 abhydrolase domain containing, maspardin	0.152	0.983	0.017
148679797	DEF8	differentially expressed in FDCP 8 homolog	0.155	0.966	0.034
157824174	HIGD2A	HIG1 hypoxia inducible domain family member 2A	0.157	0.994	0.006
157819421	CEP97	centrosomal protein 97	0.160	0.967	0.033
221040576	MPPED1	metallophosphoesterase domain containing 1	0.163	0.955	0.045
149042848	LOC100911177	uncharacterized LOC100911177	0.165	0.988	0.012
672042133	SPATA5	spermatogenesis associated 5	0.165	0.970	0.030
408772026	Afg3l1	AFG3-like AAA ATPase 1	0.166	0.983	0.017
55926133	RFC2	replication factor C subunit 2	0.168	0.956	0.044
422398900	CREBZF	CREB/ATF bZIP transcription factor	0.169	0.959	0.041
189491869	KCMF1	potassium channel modulatory factor 1	0.176	0.999	0.001
119569672	BUB3	BUB3 mitotic checkpoint protein	0.176	0.984	0.016
164565387	TBC1D14	TBC1 domain family member 14	0.177	0.956	0.044
564323153	ARMCX5	armadillo repeat containing X-linked 5	0.180	0.964	0.036
77695933	NELL2	neural EGFL like 2	0.180	0.985	0.015
61556927	EIF3G	eukaryotic translation initiation factor 3 subunit G	0.180	0.962	0.038
6981296	NUP50	nucleoporin 50	0.181	0.956	0.044
6981518	SDC1	syndecan 1	0.182	0.993	0.007
157823413	THOC3	THO complex 3	0.183	0.997	0.003
148747146	PPP2R2D	protein phosphatase 2 regulatory subunit Bdelta	0.185	0.957	0.043
568983220	TENT4A	terminal nucleotidyltransferase 4A	0.187	1.000	0.000
72004267	AKIRIN1	akirin 1	0.192	0.978	0.022
140971918	Hnrnpab	heterogeneous nuclear ribonucleoprotein A/B	0.194	0.975	0.025
58865352	NUBP2	nucleotide binding protein 2	0.200	0.954	0.046
14277700	RPS12	ribosomal protein S12	0.203	0.968	0.032
157823465	CHSY1	chondroitin sulfate synthase 1	0.204	0.994	0.006
61557082	TERF2IP	TERF2 interacting protein	0.211	0.972	0.028
58865624	NUF2	NUF2 component of NDC80 kinetochore complex	0.214	0.967	0.033
639869	CHKA	choline kinase alpha	0.216	0.963	0.037

24025618	DAB1	DAB adaptor protein 1	0.217	0.982	0.018
157817121	TCTE1	t-complex-associated-testis-expressed 1	0.219	0.972	0.028
24638440	RIMS4	regulating synaptic membrane exocytosis 4	0.220	0.975	0.025
197252056	MED1	mediator complex subunit 1	0.223	0.960	0.040
76096328	COMMD9	COMM domain containing 9	0.238	0.971	0.029
157821267	RFC5	replication factor C subunit 5	0.243	0.956	0.044
71043628	OGFRL1	opioid growth factor receptor like 1	0.245	0.961	0.039
8923415	MARCHF5	membrane associated ring-CH-type finger 5	0.250	0.978	0.022
71361655	MRPL12	mitochondrial ribosomal protein L12	0.257	0.950	0.050
404312698	GOLM2	golgi membrane protein 2	0.270	0.951	0.049
37360160	ATP11B*	ATPase phospholipid transporting 11B (putative)	0.278	0.952	0.048
59937915	ARIH2	ariadne RBR E3 ubiquitin protein ligase 2	0.280	0.953	0.047
157821915	MSANTD3	Myb/SANT DNA binding domain containing 3	0.283	0.963	0.037
148679437	HAS3	hyaluronan synthase 3	0.284	0.962	0.038
564307173	HEATR5A	HEAT repeat containing 5A	0.284	0.992	0.008
672053062	FKBP15	FKBP prolyl isomerase 15	0.290	0.978	0.022
34328151	TBR1	T-box brain transcription factor 1	0.295	0.996	0.004
157821125	COA7	cytochrome c oxidase assembly factor 7 (putative)	0.295	0.992	0.008
187937148	CXorf38	chromosome X open reading frame 38	0.303	0.971	0.029
29789082	COIL	coilin	0.305	0.992	0.008
13592081	SCTR	secretin receptor	0.306	0.988	0.012
157818733	ZBTB2	zinc finger and BTB domain containing 2	0.306	0.999	0.001
300798436	NME6	NME/NM23 nucleoside diphosphate kinase 6	0.308	0.982	0.018
62078923	DZIP1L	DAZ interacting zinc finger protein 1 like	0.315	0.976	0.024
109479851	NRDE2	NRDE-2, necessary for RNA interference, domain containing	0.330	0.968	0.032
73990974	LZTS3	leucine zipper tumor suppressor family member 3	0.345	0.956	0.044
281604129	HELQ	helicase, POLQ like	0.354	0.975	0.025
37360398	ISLR2	immunoglobulin superfamily containing leucine rich repeat 2	0.360	0.993	0.007
88853859	UBE3D	ubiquitin protein ligase E3D	0.361	0.964	0.036
77627740	ING3	inhibitor of growth family member 3	0.363	0.988	0.012

148710078	TAF5	TATA-box binding protein associated factor 5	0.366	0.954	0.046
564352668	MYCL	MYCL proto-oncogene, bHLH transcription factor	0.392	0.977	0.023
16758238	SPA17	sperm autoantigenic protein 17	0.402	0.985	0.015
23097354	FADD	Fas associated via death domain	0.403	0.970	0.030
293348214	CCDC88C	coiled-coil domain containing 88C	0.421	0.952	0.048
157820727	RPL27A	ribosomal protein L27a	0.452	0.971	0.029
70608121	Dmrtc1a	DMRT-like family C1a	0.479	0.976	0.024
149024753	DFFB	DNA fragmentation factor subunit beta	0.487	0.951	0.049
212549645	KIF18A	kinesin family member 18A	0.505	0.960	0.040
256220048	PCDHGC5	protocadherin gamma subfamily C, 5	0.666	0.988	0.012
157823385	SLITRK6	SLIT and NTRK like family member 6	0.667	0.991	0.009
112984482	SBSN	suprabasin	0.700	0.997	0.003
148704285	CBLN3	cerebellin 3 precursor	0.724	0.985	0.015
564297338	ZNF816	zinc finger protein 816	0.748	0.978	0.022
672070295	BAHCC1*	BAH domain and coiled-coil containing 1	0.769	0.983	0.017
672031995	Kdm6a	lysine demethylase 6A	0.794	0.980	0.020
26024223	ABCG5	ATP binding cassette subfamily G member 5	0.841	0.953	0.047
16758266	NME3	NME/NM23 nucleoside diphosphate kinase 3	0.921	0.999	0.001
755515866	BRAF	B-Raf proto-oncogene, serine/threonine kinase	0.990	0.980	0.020
149030324	CHRNA2	cholinergic receptor nicotinic alpha 2 subunit	0.994	0.962	0.038
157817241	ISCA2	iron-sulfur cluster assembly 2	1.006	0.973	0.027
300796953	SYCE2	synaptonemal complex central element protein 2	1.163	0.956	0.044
6978525	FASLG	Fas ligand	1.186	0.987	0.013
149045964	PTH2R	parathyroid hormone 2 receptor	1.222	0.964	0.036
56605846	DPEP3	dipeptidase 3	1.348	0.997	0.003
672084787	DPEP2	dipeptidase 2	1.392	0.984	0.016
62079153	PLET1	placenta expressed transcript 1	1.433	0.986	0.014
124487463	GPR161	G protein-coupled receptor 161	1.523	0.990	0.010
568914628	GARNL3	GTPase activating Rap/RanGAP domain like 3	1.578	0.999	0.001
157816947	GUCA1B	guanylate cyclase activator 1B	1.708	0.984	0.016
300795020	DEF6	DEF6 guanine nucleotide exchange factor	1.726	0.954	0.046
564297942	LOC103690302	AF4/FMR2 family member 2	1.909	0.979	0.021

51243038	LY6G6D	lymphocyte antigen 6 family member G6D	2.000	0.985	0.015
568974167	SLC26A11	solute carrier family 26 member 11	2.149	0.969	0.031
564297852	CRTC3	CREB regulated transcription coactivator 3	2.151	0.952	0.048
56912237	KRT28	keratin 28	2.241	0.954	0.046

Supplementary Table S17. The list of genes that are differentially expressed in the offspring hippocampus in response to prenatal BPA exposure that exhibited the changes in the expression levels correlated with the number of branches of primary hippocampal cells at DIV7. The transcriptome profiling data of DEGs in male and female rat offspring prenatally exposed to BPA (n = 6, male pups n = 3 and female pups n = 3, from independent litters) or the vehicle control (n = 6, male pups n = 3 and female pups n = 3, from independent litters) were obtained and used for the PTM analyses to identify DEGs that exhibited the changes in the expression levels correlated with the number of branches of primary hippocampal cells at DIV7.

ID	Symbol	Entrez Gene Name	log2(FC)	R values	P-values
293347435	PTPRD	protein tyrosine phosphatase receptor type D	-7.731	-0.988	0.012
564375502	Mxra7	matrix-remodelling associated 7	-7.209	-0.961	0.039
564310188	IGDCC4	immunoglobulin superfamily DCC subclass member 4	-6.728	-0.968	0.032
564314389	DZIP3	DAZ interacting zinc finger protein 3	-6.700	-0.992	0.008
149020633	TAF1D	TATA-box binding protein associated factor, RNA polymerase I subunit D	-6.476	-0.991	0.009
157818475	SMIM22	small integral membrane protein 22	-5.615	-0.992	0.008
567315993	LOC102550396	LRRGT00188	-5.600	-0.965	0.035
564312230	LOC100912948	multidrug resistance-associated protein 1-like	-5.285	-0.961	0.039
197384778	Snorc	secondary ossification center associated regulator of chondrocyte maturation	-5.044	-0.991	0.009
564323305	LOC681300	similar to CXXC finger 5	-4.672	-0.991	0.009
209447125	Ctf2	cardiotrophin 2	-4.392	-0.961	0.039
201860265	NRN1L	neuritin 1 like	-4.358	-0.969	0.031
300798104	IFNLR1	interferon lambda receptor 1	-4.248	-0.991	0.009
51591901	MPIG6B	megakaryocyte and platelet inhibitory receptor G6b	-4.170	-0.961	0.039
564298047	GDPD5	glycerophosphodiester phosphodiesterase domain containing 5	-4.163	-0.998	0.002
300798035	NRG4	neuregulin 4	-4.044	-0.971	0.029
61556838	Raet1d/Raet1e	retinoic acid early transcript 1E	-4.044	-0.971	0.029
114145748	LOC680227	LRRGT00193	-3.907	-0.969	0.031
194474016	SLC30A8	solute carrier family 30 member 8	-3.807	-0.962	0.038
6978515	APOA1	apolipoprotein A1	-3.807	-0.961	0.039
117647210	CTRC	chymotrypsin C	-3.807	-0.961	0.039
8392926	ASGR2	asialoglycoprotein receptor 2	-3.700	-0.975	0.025
48040447	SUCNR1	succinate receptor 1	-3.700	-0.961	0.039
194473646	UPK3A	uroplakin 3A	-3.585	-0.961	0.039

164518908	RAB25	RAB25, member RAS oncogene family	-3.459	-0.961	0.039
285026465	HS3ST3A1	heparan sulfate-glucosamine 3-sulfotransferase 3A1	-3.322	-0.961	0.039
148710035	PITX3	paired like homeodomain 3	-3.322	-0.961	0.039
56788780	KRT19	keratin 19	-3.248	-0.995	0.005
9506451	CA5A	carbonic anhydrase 5A	-3.248	-0.978	0.022
564375434	CEP295NL	CEP295 N-terminal like	-3.248	-0.955	0.045
157822159	CCDC42	coiled-coil domain containing 42	-3.170	-0.961	0.039
123173794	GSG1	germ cell associated 1	-3.170	-0.961	0.039
392339806	CFAP69	cilia and flagella associated protein 69	-2.947	-0.968	0.032
20302091	PLB1	phospholipase B1	-2.907	-0.962	0.038
157821903	Slc7a15	solute carrier family 7 (cationic amino acid transporter, y+ system), member 15	-2.907	-0.962	0.038
77917534	CBLC	Cbl proto-oncogene C	-2.807	-0.961	0.039
51948496	SLC22A18	solute carrier family 22 member 18	-2.687	-0.987	0.013
298566276	Ces1a	carboxylesterase 1A	-2.585	-0.961	0.039
57222328	PFN4	profilin family member 4	-2.585	-0.961	0.039
157786772	KREMEN2	kringle containing transmembrane protein 2	-2.585	-0.961	0.039
306482632	BPIFB4	BPI fold containing family B member 4	-2.585	-0.961	0.039
57222300	Klra2	killer cell lectin-like receptor, subfamily A, member 2	-2.585	-0.961	0.039
157822463	Nkx6-3	NK6 homeobox 3	-2.585	-0.961	0.039
157822853	GUCA1A	guanylate cyclase activator 1A	-2.509	-0.962	0.038
162417984	Vom2r12 (includes others)	vomeronasal 2 receptor, 80	-2.459	-0.997	0.003
564391680	MAP3K8	mitogen-activated protein kinase kinase kinase 8	-2.426	-0.970	0.030
62945342	LAX1	lymphocyte transmembrane adaptor 1	-2.322	-0.961	0.039
187469451	CLEC7A	C-type lectin domain containing 7A	-2.322	-0.961	0.039
568914626	GARNL3	GTPase activating Rap/RanGAP domain like 3	-2.261	-0.966	0.034
62079023	ADTRP	androgen dependent TFPI regulating protein	-2.246	-0.998	0.002
66730349	SPIB	Spi-B transcription factor	-2.170	-0.991	0.009
404247470	OLR1	oxidized low density lipoprotein receptor 1	-2.170	-0.967	0.033
148672128	SMAGP	small cell adhesion glycoprotein	-2.077	-0.984	0.016
55741882	ZPBP2	zona pellucida binding protein 2	-2.022	-0.968	0.032
157822587	PDE6B	phosphodiesterase 6B	-2.000	-0.961	0.039
157818091	TMEM182	transmembrane protein 182	-2.000	-0.961	0.039

296483047	SIX1	SIX homeobox 1	-2.000	-0.961	0.039
117647206	DDX4	DEAD-box helicase 4	-2.000	-0.961	0.039
19424304	CHRNB3	cholinergic receptor nicotinic beta 3 subunit	-2.000	-0.961	0.039
47577151	Olfr1441	olfactory receptor 1441	-1.972	-0.961	0.039
157820583	ANKRD34C	ankyrin repeat domain 34C	-1.939	-0.975	0.025
117647214	EDN3	endothelin 3	-1.913	-0.986	0.014
157823875	EPS8L1	EPS8 like 1	-1.907	-0.987	0.013
81295367	Abcg3	ATP binding cassette subfamily G member 3	-1.898	-0.951	0.049
672029702	TUT7	terminal uridylyl transferase 7	-1.854	-0.963	0.037
13592031	PTGER2	prostaglandin E receptor 2	-1.845	-0.953	0.047
19424240	PCSK4	proprotein convertase subtilisin/kexin type 4	-1.740	-0.985	0.015
480306394	Mcpt4	mast cell protease 4	-1.739	-0.951	0.049
9910234	IFIT1B	interferon induced protein with tetratricopeptide repeats 1B	-1.678	-0.961	0.039
31542125	ALOX15	arachidonate 15-lipoxygenase	-1.611	-0.981	0.019
76443687	SLC4A1	solute carrier family 4 member 1 (Diego blood group)	-1.599	-0.984	0.016
157821527	RHOD	ras homolog family member D	-1.597	-0.962	0.038
209870037	INSRR	insulin receptor related receptor	-1.585	-0.988	0.012
194473618	SCX	scleraxis bHLH transcription factor	-1.585	-0.996	0.004
157818603	CLCA2	chloride channel accessory 2	-1.585	-0.961	0.039
685156911	NLRP4	NLR family pyrin domain containing 4	-1.585	-0.961	0.039
149034165	GALNT15	polypeptide N-acetylgalactosaminyltransferase 15	-1.561	-0.960	0.040
13929066	CPZ	carboxypeptidase Z	-1.549	-0.970	0.030
13929126	GALNT5	polypeptide N-acetylgalactosaminyltransferase 5	-1.478	-0.985	0.015
18677739	CDKN2B	cyclin dependent kinase inhibitor 2B	-1.454	-0.959	0.041
81295349	SLC52A3	solute carrier family 52 member 3	-1.453	-0.991	0.009
6981148	LEP	leptin	-1.436	-0.962	0.038
283806636	ZNF831	zinc finger protein 831	-1.433	-0.992	0.008
564314671	VPS8	VPS8 subunit of CORVET complex	-1.386	-0.965	0.035
149065466	ARHGEF5	Rho guanine nucleotide exchange factor 5	-1.382	-0.957	0.043
157786780	MELTF	melanotransferrin	-1.333	-0.987	0.013
29789044	SNAI2	snail family transcriptional repressor 2	-1.328	-0.989	0.011
157823345	LRR1	leucine rich repeat protein 1	-1.322	-0.975	0.025
307746876	Pzp	PZP, alpha-2-macroglobulin like	-1.322	-0.961	0.039
148694035	SENP8	SUMO peptidase family member, NEDD8 specific	-1.276	-0.989	0.011

62656582	KIAA0100	KIAA0100	-1.266	-0.974	0.026
187281975	DENND1C	DENN domain containing 1C	-1.216	-0.995	0.005
56119141	BTK	Bruton tyrosine kinase	-1.205	-0.985	0.015
155369293	AEBP1	AE binding protein 1	-1.198	-0.952	0.048
564300462	DCHS2	dachsous cadherin-related 2	-1.190	-0.999	0.001
157820725	SUN5	Sad1 and UNC84 domain containing 5	-1.187	-0.953	0.047
58866008	TMC5	transmembrane channel like 5	-1.180	-0.984	0.016
392334475	Myb	MYB proto-oncogene, transcription factor	-1.175	-0.971	0.029
71795615	UPP1	uridine phosphorylase 1	-1.167	-0.971	0.029
157823801	SLC50A1	solute carrier family 50 member 1	-1.136	-0.986	0.014
125347412	FAM72A	family with sequence similarity 72 member A	-1.106	-0.993	0.007
197385083	C1orf194	chromosome 1 open reading frame 194	-1.090	-0.973	0.027
24308484	SLC17A8	solute carrier family 17 member 8	-1.089	-0.967	0.033
157819247	CPA4	carboxypeptidase A4	-1.087	-0.961	0.039
320089574	FAM161A	FAM161 centrosomal protein A	-1.080	-0.962	0.038
9910378	CDC42SE2	CDC42 small effector 2	-1.069	-0.986	0.014
197384727	Smco4	single-pass membrane protein with coiled-coil domains 4	-1.065	-0.974	0.026
672020326	MTA3	metastasis associated 1 family member 3	-1.047	-0.986	0.014
300797728	MGST3	microsomal glutathione S-transferase 3	-1.011	-0.969	0.031
156231008	PRND	prion like protein doppel	-1.000	-0.961	0.039
59676595	FAM20A	FAM20A golgi associated secretory pathway pseudokinase	-1.000	-0.954	0.046
569009290	TENM1	teneurin transmembrane protein 1	-0.985	-0.982	0.018
407228396	THEMIS2	thymocyte selection associated family member 2	-0.980	-0.970	0.030
157817157	FAM166C	family with sequence similarity 166 member C	-0.979	-0.971	0.029
157819493	Igbp1b	immunoglobulin (CD79A) binding protein 1b	-0.966	-0.953	0.047
157817989	MDFI	MyoD family inhibitor	-0.958	-0.977	0.023
672052120	RBM12B	RNA binding motif protein 12B	-0.957	-0.964	0.036
564392197	LOC684327	similar to inter-alpha (globulin) inhibitor H5	-0.954	-0.986	0.014
157818275	KCNG4	potassium voltage-gated channel modifier subfamily G member 4	-0.952	-0.998	0.002
148680846	HIC1	HIC ZBTB transcriptional repressor 1	-0.940	-0.973	0.027
157823079	RBKS	ribokinase	-0.939	-0.974	0.026

198442873	CDC14A	cell division cycle 14A	-0.936	-0.977	0.023
71043648	LOC499742	LRRG00137	-0.931	-0.975	0.025
62945330	SLC8B1	solute carrier family 8 member B1	-0.929	-0.974	0.026
50657416	C1RL	complement C1r subcomponent like	-0.918	-0.961	0.039
157819487	TACO1	translational activator of cytochrome c oxidase I	-0.917	-0.955	0.045
564318923	WDR17	WD repeat domain 17	-0.904	-0.964	0.036
14861868	Ptprv	protein tyrosine phosphatase, receptor type, V	-0.893	-0.964	0.036
149056256	FXYD5	FXYD domain containing ion transport regulator 5	-0.880	-0.951	0.049
392351290	DNAH9	dynein axonemal heavy chain 9	-0.875	-0.965	0.035
209870105	GPR37L1	G protein-coupled receptor 37 like 1	-0.870	-0.955	0.045
149031998	ACVRL1	activin A receptor like type 1	-0.852	-0.972	0.028
6980958	SLC2A4	solute carrier family 2 member 4	-0.837	-0.978	0.022
392334002	CCDC3	coiled-coil domain containing 3	-0.833	-0.984	0.016
56605720	GADD45B	growth arrest and DNA damage inducible beta	-0.828	-0.972	0.028
157817065	KCNK16	potassium two pore domain channel subfamily K member 16	-0.807	-0.975	0.025
564396113	ZCCHC14	zinc finger CCHC-type containing 14	-0.797	-0.971	0.029
564345556	CROT	carnitine O-octanoyltransferase	-0.790	-0.993	0.007
300798165	ZBTB40	zinc finger and BTB domain containing 40	-0.788	-0.995	0.005
942523340	CAPRIN2	caprin family member 2	-0.774	-0.964	0.036
157819205	EFHC2	EF-hand domain containing 2	-0.772	-0.955	0.045
57528269	ABHD14A	abhydrolase domain containing 14A	-0.760	-0.984	0.016
13591914	ANPEP	alanyl aminopeptidase, membrane	-0.757	-0.982	0.018
564312886	DNAH2	dynein axonemal heavy chain 2	-0.740	-0.985	0.015
157786850	TUBD1	tubulin delta 1	-0.740	-0.995	0.005
208973286	RBM46	RNA binding motif protein 46	-0.724	-0.989	0.011
19173756	ERG	ETS transcription factor ERG	-0.718	-0.979	0.021
149047075	Spaca6	sperm acrosome associated 6	-0.715	-0.985	0.015
564378828	TFR2	transferrin receptor 2	-0.707	-0.998	0.002
55742713	ECM1	extracellular matrix protein 1	-0.699	-0.993	0.007
157816997	BDH2	3-hydroxybutyrate dehydrogenase 2	-0.698	-0.995	0.005
48675865	PDP2	pyruvate dehyrogenase phosphatase catalytic subunit 2	-0.692	-0.954	0.046
38454234	COL27A1	collagen type XXVII alpha 1 chain	-0.677	-0.967	0.033
564320335	TMEM241	transmembrane protein 241	-0.675	-0.971	0.029
74142284	DSE	dermatan sulfate epimerase	-0.667	-0.993	0.007
157818909	Zim1	zinc finger, imprinted 1	-0.665	-0.996	0.004
67514566	POLA2	DNA polymerase alpha 2, accessory subunit	-0.665	-0.957	0.043

672057459	DGKA	diacylglycerol kinase alpha	-0.664	-0.959	0.041
13592057	RPL18	ribosomal protein L18	-0.660	-0.958	0.042
114145782	MORN5	MORN repeat containing 5	-0.657	-0.979	0.021
77020281	CD55	CD55 molecule (Cromer blood group)	-0.648	-0.977	0.023
672039742	TKFC	triokinase and FMN cyclase	-0.646	-0.961	0.039
13786136	PDGFC	platelet derived growth factor C	-0.642	-0.961	0.039
157786864	PHOSPHO1	phosphoethanolamine/phosphocholine phosphatase 1	-0.638	-0.951	0.049
149061352	ADAM12	ADAM metallopeptidase domain 12	-0.638	-0.996	0.004
672060362	ELFN2	extracellular leucine rich repeat and fibronectin type III domain containing 2	-0.636	-0.985	0.015
19424350	GBP2	guanylate binding protein 2	-0.635	-0.952	0.048
58865948	CREB3L2	cAMP responsive element binding protein 3 like 2	-0.634	-0.972	0.028
50657355	TOP1MT	DNA topoisomerase I mitochondrial	-0.632	-0.958	0.042
397529557	C8orf58	chromosome 8 open reading frame 58	-0.629	-0.996	0.004
58865854	SCRN2	secernin 2	-0.627	-0.957	0.043
16758622	IFT172	intraflagellar transport 172	-0.627	-0.988	0.012
478732983	MAP3K5	mitogen-activated protein kinase kinase kinase 5	-0.616	-0.992	0.008
149068766	PLEKHB1	pleckstrin homology domain containing B1	-0.608	-0.972	0.028
157819065	ADAMTS15	ADAM metallopeptidase with thrombospondin type 1 motif 15	-0.604	-0.994	0.006
157818491	DUS2	dihydrouridine synthase 2	-0.602	-0.954	0.046
27465529	SLC9A4	solute carrier family 9 member A4	-0.601	-0.956	0.044
293347888	SRBD1	S1 RNA binding domain 1	-0.598	-0.991	0.009
564387543	UGGT2	UDP-glucose glycoprotein glucosyltransferase 2	-0.596	-0.970	0.030
157817670	SLC2A10	solute carrier family 2 member 10	-0.595	-0.952	0.048
108935976	DISC1	DISC1 scaffold protein	-0.590	-0.992	0.008
197927123	LYRM7	LYR motif containing 7	-0.587	-0.978	0.022
672086986	SLC38A5	solute carrier family 38 member 5	-0.583	-0.979	0.021
564394999	CLGN	calmegin	-0.582	-0.979	0.021
16758560	WIF1	WNT inhibitory factor 1	-0.573	-0.980	0.020
78486556	RUSF1	RUS family member 1	-0.572	-0.954	0.046
564390348	Klhl3	kelch-like family member 3	-0.566	-0.975	0.025
62078799	QRSL1	glutaminyl-tRNA amidotransferase subunit QRSL1	-0.566	-0.972	0.028
157817903	Dcaf12l1	DDB1 and CUL4 associated factor 12-like 1	-0.565	-0.954	0.046
57012436	Krt10	keratin 10	-0.562	-0.971	0.029
8393469	S1PR2	sphingosine-1-phosphate receptor 2	-0.561	-0.955	0.045

300798350	LRRK1	leucine rich repeat kinase 1	-0.554	-0.972	0.028
199562000	USP40	ubiquitin specific peptidase 40	-0.552	-0.979	0.021
171846640	FBLN1	fibulin 1	-0.543	-0.997	0.003
672066171	GIN1	gypsy retrotransposon integrase 1	-0.539	-0.955	0.045
6754808	NDP	norrin cystine knot growth factor NDP	-0.538	-0.962	0.038
16758390	CLIC5	chloride intracellular channel 5	-0.535	-0.966	0.034
20302097	PIGL	phosphatidylinositol glycan anchor biosynthesis class L	-0.534	-0.971	0.029
148683700	TMEM98	transmembrane protein 98	-0.533	-0.958	0.042
19424232	CSF2RB	colony stimulating factor 2 receptor subunit beta	-0.524	-0.990	0.010
124244050	PPIP5K1	diphosphoinositol pentakisphosphate kinase 1	-0.522	-0.970	0.030
157822555	RIN3	Ras and Rab interactor 3	-0.520	-0.999	0.001
568966731	HMG20B	high mobility group 20B	-0.518	-0.961	0.039
6981180	MAOB	monoamine oxidase B	-0.518	-0.952	0.048
148692356	ARHGEF1	Rho guanine nucleotide exchange factor 1	-0.517	-0.985	0.015
392338379	SLC26A8	solute carrier family 26 member 8	-0.514	-0.952	0.048
149047863	LOC690190	hypothetical protein LOC690190	-0.514	-0.988	0.012
147900684	TLR7	toll like receptor 7	-0.511	-0.978	0.022
308044487	KIAA0319	KIAA0319	-0.509	-0.981	0.019
148698795	GPX7	glutathione peroxidase 7	-0.507	-0.998	0.002
654824082	Fbxl21	F-box and leucine-rich repeat protein 21	-0.500	-0.986	0.014
37693510	Bst2	bone marrow stromal cell antigen 2	-0.500	-0.955	0.045
50233928	TMEM159	transmembrane protein 159	-0.497	-0.989	0.011
564330609	SYT17	synaptotagmin 17	-0.494	-0.982	0.018
84662732	DNASE1L1	deoxyribonuclease 1 like 1	-0.494	-0.977	0.023
62078635	CCDC153	coiled-coil domain containing 153	-0.494	-0.991	0.009
399220341	SLC2A13	solute carrier family 2 member 13	-0.492	-0.992	0.008
157819347	CDC6	cell division cycle 6	-0.492	-0.989	0.011
6978867	GABRB1	gamma-aminobutyric acid type A receptor subunit beta1	-0.487	-0.973	0.027
149058686	PIGR	polymeric immunoglobulin receptor	-0.485	-0.977	0.023
164607119	SUMF2	sulfatase modifying factor 2	-0.483	-0.963	0.037
16758322	SYNGR2	synaptogyrin 2	-0.481	-0.951	0.049
157073947	C1orf74	chromosome 1 open reading frame 74	-0.478	-0.994	0.006
189011606	NCEH1	neutral cholesterol ester hydrolase 1	-0.476	-0.968	0.032
312922379	TNN	tenascin N	-0.469	-0.960	0.040
157816939	WASHC3	WASH complex subunit 3	-0.469	-0.965	0.035
157823399	COG4	component of oligomeric golgi complex 4	-0.468	-0.974	0.026

75905809	AKAP12	A-kinase anchoring protein 12	-0.466	-0.981	0.019
41056215	XRCC5	X-ray repair cross complementing 5	-0.466	-0.953	0.047
672044191	TBCK	TBC1 domain containing kinase	-0.465	-0.990	0.010
56090632	DMAC2L	distal membrane arm assembly complex 2 like	-0.463	-0.999	0.001
148669431	DNAJC27	DnaJ heat shock protein family (Hsp40) member C27	-0.459	-1.000	0.000
56090411	POLE3	DNA polymerase epsilon 3, accessory subunit	-0.456	-0.985	0.015
149067372	MTERF2	mitochondrial transcription termination factor 2	-0.455	-0.956	0.044
157817743	CDH5	cadherin 5	-0.454	-0.971	0.029
9437326	SLC4A4	solute carrier family 4 member 4	-0.453	-0.960	0.040
157817911	C21orf62	chromosome 21 open reading frame 62	-0.451	-0.975	0.025
33414515	PXK	PX domain containing serine/threonine kinase like	-0.450	-0.976	0.024
58865380	STAT2	signal transducer and activator of transcription 2	-0.450	-0.988	0.012
201066407	EAPP	E2F associated phosphoprotein	-0.444	-0.953	0.047
77993368	ACSF2	acyl-CoA synthetase family member 2	-0.444	-0.985	0.015
392331978	CDR2L	cerebellar degeneration related protein 2 like	-0.442	-0.960	0.040
157786756	CDC45	cell division cycle 45	-0.442	-0.992	0.008
149058661	RAB7B	RAB7B, member RAS oncogene family	-0.439	-0.971	0.029
148686921	SLC24A4	solute carrier family 24 member 4	-0.435	-0.985	0.015
404312655	SDR42E1	short chain dehydrogenase/reductase family 42E, member 1	-0.435	-0.999	0.001
149038013	SLC9A5	solute carrier family 9 member A5	-0.426	-0.955	0.045
76443683	LOC100912042/Surf2	surfeit 2	-0.423	-0.978	0.022
300797330	PTPRU	protein tyrosine phosphatase receptor type U	-0.422	-0.960	0.040
210032365	HSP90B1	heat shock protein 90 beta family member 1	-0.421	-0.987	0.013
56090564	GALM	galactose mutarotase	-0.420	-0.989	0.011
157823279	CGNL1	cingulin like 1	-0.418	-0.965	0.035
74218228	HNRNPC	heterogeneous nuclear ribonucleoprotein C	-0.418	-0.971	0.029
83642834	NAGK	N-acetylglucosamine kinase	-0.416	-0.955	0.045
13994179	SLC24A2	solute carrier family 24 member 2	-0.416	-0.996	0.004

62078539	Pagr1	Paxip1-associated glutamate-rich protein 1	-0.415	-0.967	0.033
62945312	CXCL16	C-X-C motif chemokine ligand 16	-0.414	-0.990	0.010
68342019	LRRC17	leucine rich repeat containing 17	-0.413	-0.974	0.026
13591949	GATM	glycine amidinotransferase	-0.409	-0.984	0.016
23463307	RIOX2	ribosomal oxygenase 2	-0.409	-0.960	0.040
672061813	ACSBG1	acyl-CoA synthetase bubblegum family member 1	-0.409	-0.961	0.039
157786690	PRKCA	protein kinase C alpha	-0.408	-0.979	0.021
564351356	PAPPA	pappalysin 1	-0.407	-0.961	0.039
13162347	FDXR	ferredoxin reductase	-0.405	-0.970	0.030
6981664	TNFRSF1A	TNF receptor superfamily member 1A	-0.403	-0.959	0.041
149049048	RECQL	RecQ like helicase	-0.403	-0.980	0.020
127140886	EML6	EMAP like 6	-0.403	-0.981	0.019
122065191	ABAT	4-aminobutyrate aminotransferase	-0.401	-0.969	0.031
58865466	SLC37A1	solute carrier family 37 member 1	-0.401	-0.984	0.016
77695926	STAT1	signal transducer and activator of transcription 1	-0.399	-0.962	0.038
149041576	REXO2	RNA exonuclease 2	-0.398	-0.981	0.019
148666792	ARHGAP25	Rho GTPase activating protein 25	-0.396	-0.970	0.030
78187977	TCF19	transcription factor 19	-0.393	-0.997	0.003
77917572	LIPA	lipase A, lysosomal acid type	-0.392	-0.975	0.025
149052857	KCNJ12	potassium inwardly rectifying channel subfamily J member 12	-0.392	-0.993	0.007
157820327	THSD1	thrombospondin type 1 domain containing 1	-0.391	-0.952	0.048
187937124	TMEM126B	transmembrane protein 126B	-0.389	-0.955	0.045
51948488	SIRT5	sirtuin 5	-0.388	-0.989	0.011
296040479	TXNRD3	thioredoxin reductase 3	-0.387	-0.972	0.028
148690851	RCN3	reticulocalbin 3	-0.381	-0.987	0.013
564305413	E130308A19Rik	RIKEN cDNA E130308A19 gene	-0.381	-0.979	0.021
40786487	GPR108	G protein-coupled receptor 108	-0.379	-0.973	0.027
18426846	DCBLD2	discoidin, CUB and LCCL domain containing 2	-0.378	-0.973	0.027
564299653	FAM169A	family with sequence similarity 169 member A	-0.372	-0.956	0.044
113061	CHRNA3	cholinergic receptor nicotinic alpha 3 subunit	-0.372	-0.995	0.005
8393861	HPCAL4	hippocalcin like 4	-0.370	-0.988	0.012
157819959	PCDHB2	protocadherin beta 2	-0.369	-0.990	0.010
158303308	PCCA	propionyl-CoA carboxylase subunit alpha	-0.369	-0.991	0.009
9247217	MSX1	msh homeobox 1	-0.368	-0.968	0.032

157822461	C20orf194	chromosome 20 open reading frame 194	-0.366	-0.966	0.034
158534064	RET	ret proto-oncogene	-0.365	-0.994	0.006
6978761	DGKG	diacylglycerol kinase gamma	-0.362	-0.969	0.031
16758712	PDIA4	protein disulfide isomerase family A member 4	-0.362	-0.985	0.015
29293811	SERPINF1	serpin family F member 1	-0.359	-0.965	0.035
78042613	NICN1	nicolin 1	-0.354	-0.958	0.042
672052705	FRRS1L	ferric chelate reductase 1 like	-0.354	-0.960	0.040
16758024	SYT9	synaptotagmin 9	-0.351	-0.991	0.009
149060100	AIFM1	apoptosis inducing factor mitochondria associated 1	-0.351	-0.975	0.025
564329392	FLNA	filamin A	-0.347	-0.970	0.030
56119120	SNF8	SNF8 subunit of ESCRT-II	-0.343	-0.987	0.013
62078847	TSEN2	tRNA splicing endonuclease subunit 2	-0.343	-0.996	0.004
77157795	MAL2	mal, T cell differentiation protein 2 (gene/pseudogene)	-0.342	-0.966	0.034
11693162	INSIG1	insulin induced gene 1	-0.339	-0.958	0.042
56605710	LTBR	lymphotoxin beta receptor	-0.338	-0.957	0.043
71361669	CIT	citron rho-interacting serine/threonine kinase	-0.336	-0.976	0.024
57527061	ZGPAT	zinc finger CCCH-type and G-patch domain containing	-0.335	-0.992	0.008
148683194	INTS3	integrator complex subunit 3	-0.335	-0.999	0.001
78486544	SLC5A2	solute carrier family 5 member 2	-0.334	-0.960	0.040
300794275	MFSD10	major facilitator superfamily domain containing 10	-0.330	-0.995	0.005
672038615	GSG1L	GSG1 like	-0.330	-0.987	0.013
564309649	CCDC159	coiled-coil domain containing 159	-0.329	-0.979	0.021
22024392	KIF1C	kinesin family member 1C	-0.328	-0.997	0.003
77628027	PSMC3IP	PSMC3 interacting protein	-0.328	-0.984	0.016
61557206	ZBTB16	zinc finger and BTB domain containing 16	-0.326	-0.964	0.036
564344373	ZMYND8	zinc finger MYND-type containing 8	-0.325	-0.994	0.006
149045696	Ccl27a	chemokine (C-C motif) ligand 27A	-0.325	-0.991	0.009
51036680	SLC29A3	solute carrier family 29 member 3	-0.322	-0.995	0.005
58865398	LAP3	leucine aminopeptidase 3	-0.320	-0.997	0.003
564304076	FGD5	FYVE, RhoGEF and PH domain containing 5	-0.319	-0.961	0.039
672050244	APLF	aprataxin and PNKP like factor	-0.318	-0.963	0.037
6678297	TEX261	testis expressed 261	-0.318	-0.954	0.046
157823879	NUDT12	nudix hydrolase 12	-0.317	-0.965	0.035
157819457	MAP3K14	mitogen-activated protein kinase kinase kinase 14	-0.317	-0.977	0.023

40018538	ADI1	acireductone dioxygenase 1	-0.316	-0.995	0.005
300797242	SPG11	SPG11 vesicle trafficking associated, spatacsin	-0.316	-0.960	0.040
57528352	DMAC2	distal membrane arm assembly complex 2	-0.315	-0.985	0.015
281332095	RB1	RB transcriptional corepressor 1	-0.313	-0.974	0.026
149041432	THY1	Thy-1 cell surface antigen	-0.313	-0.957	0.043
157786608	MRPL55	mitochondrial ribosomal protein L55	-0.313	-0.993	0.007
564336403	EXOSC8	exosome component 8	-0.309	-0.965	0.035
167860097	FN3KRP	fructosamine 3 kinase related protein	-0.308	-0.995	0.005
966975500	MMP17	matrix metallopeptidase 17	-0.306	-0.970	0.030
29789369	PTPRG	protein tyrosine phosphatase receptor type G	-0.306	-0.982	0.018
545532952	EIF4E3	eukaryotic translation initiation factor 4E family member 3	-0.305	-0.958	0.042
564372688	RPA1	replication protein A1	-0.305	-0.968	0.032
399124777	GLS2	glutaminase 2	-0.305	-0.976	0.024
300796069	THADA	THADA armadillo repeat containing	-0.303	-0.969	0.031
198041989	PARVB	parvin beta	-0.302	-0.988	0.012
157820737	NUSAP1	nucleolar and spindle associated protein 1	-0.298	-0.992	0.008
149032924	ARG1	arginase 1	-0.296	-0.984	0.016
6978888	GFRA1	GDNF family receptor alpha 1	-0.294	-0.965	0.035
167560911	SGF29	SAGA complex associated factor 29	-0.293	-0.957	0.043
564358911	CHPT1	choline phosphotransferase 1	-0.292	-0.990	0.010
157817979	Egfem1	EGF-like and EMI domain containing 1	-0.291	-0.976	0.024
74223968	5031425E22Rik	RIKEN cDNA 5031425E22 gene	-0.290	-0.994	0.006
149057745	NEK3	NIMA related kinase 3	-0.290	-0.991	0.009
149063995	GMPR2	guanosine monophosphate reductase 2	-0.289	-0.993	0.007
162287200	CD82	CD82 molecule	-0.289	-0.980	0.020
157820517	CARD6	caspase recruitment domain family member 6	-0.287	-0.967	0.033
564387894	BTD	biotinidase	-0.287	-0.995	0.005
157820833	HERC3	HECT and RLD domain containing E3 ubiquitin protein ligase 3	-0.287	-0.951	0.049
149034870	RNF6	ring finger protein 6	-0.283	-0.996	0.004
564367076	Mocs1	molybdenum cofactor synthesis 1	-0.281	-0.950	0.050
142349612	GLUL	glutamate-ammonia ligase	-0.279	-0.974	0.026
13928886	MAP2K1	mitogen-activated protein kinase kinase 1	-0.278	-0.956	0.044
564364473	RNF111	ring finger protein 111	-0.276	-0.969	0.031
226874871	OMG	oligodendrocyte myelin glycoprotein	-0.274	-0.956	0.044

62078997	WDR1	WD repeat domain 1	-0.273	-0.988	0.012
56605664	METTL23	methyltransferase like 23	-0.272	-0.977	0.023
149022319	AGPS	alkylglycerone phosphate synthase	-0.270	-0.999	0.001
157819337	SLC35B4	solute carrier family 35 member B4	-0.270	-0.959	0.041
57164113	NSDHL	NAD(P) dependent steroid dehydrogenase-like	-0.269	-0.989	0.011
74354506	ACBD5	acyl-CoA binding domain containing 5	-0.269	-0.994	0.006
392339847	CADPS2	calcium dependent secretion activator 2	-0.268	-0.991	0.009
928136440	SRRT	serrate, RNA effector molecule	-0.268	-0.952	0.048
38181552	SCG2	secretogranin II	-0.267	-0.981	0.019
149017535	HDAC10	histone deacetylase 10	-0.267	-0.954	0.046
157819753	RCN1	reticulocalbin 1	-0.266	-0.995	0.005
300795339	RYR2	ryanodine receptor 2	-0.265	-0.985	0.015
564382316	HSD11B1	hydroxysteroid 11-beta dehydrogenase 1	-0.265	-0.996	0.004
149041411	SC5D	sterol-C5-desaturase	-0.264	-0.973	0.027
148372343	RAMP2	receptor activity modifying protein 2	-0.264	-0.978	0.022
564344520	LOC102555457	engulfment and cell motility protein 2-like	-0.261	-0.981	0.019
564382292	ANGEL2	angel homolog 2	-0.258	-0.961	0.039
17865325	GLRB	glycine receptor beta	-0.256	-0.970	0.030
672041250	ARHGEF28	Rho guanine nucleotide exchange factor 28	-0.253	-0.985	0.015
8394502	UBC	ubiquitin C	-0.249	-0.970	0.030
149057830	Hgsnat	heparan-alpha-glucosaminide N-acetyltransferase	-0.248	-0.968	0.032
157823901	TSPAN9	tetraspanin 9	-0.247	-0.979	0.021
6978631	CD4	CD4 molecule	-0.245	-0.987	0.013
564391295	DUSP22	dual specificity phosphatase 22	-0.244	-0.954	0.046
290563168	DUSP3	dual specificity phosphatase 3	-0.243	-0.996	0.004
12018300	AKAP6	A-kinase anchoring protein 6	-0.242	-0.956	0.044
764020083	CLUH	clustered mitochondria homolog	-0.242	-0.958	0.042
300793894	URB1	URB1 ribosome biogenesis homolog	-0.242	-0.960	0.040
197382169	CNTROB	centrobin, centriole duplication and spindle assembly protein	-0.241	-0.957	0.043
12831215	KCNK10	potassium two pore domain channel subfamily K member 10	-0.240	-0.964	0.036
57192	P3H4	prolyl 3-hydroxylase family member 4 (inactive)	-0.240	-0.982	0.018
584277046	SLC1A3	solute carrier family 1 member 3	-0.232	-0.985	0.015
293346766	TCAF1	TRPM8 channel associated factor 1	-0.232	-0.988	0.012

9457244	RBBP9	RB binding protein 9, serine hydrolase	-0.231	-0.991	0.009
157820653	TMEM63C	transmembrane protein 63C	-0.229	-0.976	0.024
58865718	HERC4	HECT and RLD domain containing E3 ubiquitin protein ligase 4	-0.228	-0.993	0.007
149062310	BSCL2	BSCL2 lipid droplet biogenesis associated, seipin	-0.228	-0.992	0.008
281485606	STT3B	STT3 oligosaccharyltransferase complex catalytic subunit B	-0.228	-0.988	0.012
672050038	NDNF	neuron derived neurotrophic factor	-0.226	-0.952	0.048
293345175	DHX29	DExH-box helicase 29	-0.225	-0.962	0.038
148697866	FAM3A	FAM3 metabolism regulating signaling molecule A	-0.222	-0.951	0.049
157786994	C1orf21	chromosome 1 open reading frame 21	-0.222	-0.999	0.001
78369663	SLC38A9	solute carrier family 38 member 9	-0.222	-0.975	0.025
148689145	CPNE4	copine 4	-0.222	-0.962	0.038
56605770	RRP8	ribosomal RNA processing 8	-0.221	-0.961	0.039
148695758	CAPRIN1	cell cycle associated protein 1	-0.221	-0.964	0.036
158631207	YIF1A	Yip1 interacting factor homolog A, membrane trafficking protein	-0.220	-0.955	0.045
157822873	FBH1	F-box DNA helicase 1	-0.219	-0.988	0.012
564298436	WDR11	WD repeat domain 11	-0.216	-0.964	0.036
6978890	GGH	gamma-glutamyl hydrolase	-0.215	-0.970	0.030
8393643	KCNAB1	potassium voltage-gated channel subfamily A member regulatory beta subunit 1	-0.214	-0.959	0.041
672066638	CLEC16A	C-type lectin domain containing 16A	-0.212	-0.995	0.005
13928780	POR	cytochrome p450 oxidoreductase	-0.211	-0.957	0.043
25742763	HSPA5	heat shock protein family A (Hsp70) member 5	-0.211	-0.954	0.046
158749540	NPEPPS	aminopeptidase puromycin sensitive	-0.210	-0.964	0.036
18959250	PRKCD	protein kinase C delta	-0.210	-0.999	0.001
281306814	RPS6KA2	ribosomal protein S6 kinase A2	-0.210	-0.953	0.047
149058126	ALDH9A1	aldehyde dehydrogenase 9 family member A1	-0.208	-0.969	0.031
37359962	PLPPR4	phospholipid phosphatase related 4	-0.207	-0.997	0.003
225543229	TIAM1	TIAM Rac1 associated GEF 1	-0.207	-0.989	0.011
148667192	LRTM2	leucine rich repeats and transmembrane domains 2	-0.207	-0.996	0.004
564372562	PFAS	phosphoribosylformylglycinamidine synthase	-0.206	-0.950	0.050
6981504	ATXN1	ataxin 1	-0.204	-0.984	0.016
157818191	SETD6	SET domain containing 6, protein lysine methyltransferase	-0.203	-0.965	0.035

564391231	SERPINB9	serpin family B member 9	-0.203	-0.979	0.021
13027430	WDR7	WD repeat domain 7	-0.202	-0.961	0.039
142385975	RNF25	ring finger protein 25	-0.202	-0.963	0.037
55741502	ACAT2	acetyl-CoA acetyltransferase 2	-0.202	-0.966	0.034
48675867	PLPP3	phospholipid phosphatase 3	-0.201	-0.994	0.006
157786582	C16orf89	chromosome 16 open reading frame 89	-0.201	-0.958	0.042
38454284	PPM1E	protein phosphatase, Mg2+/Mn2+ dependent 1E	-0.201	-0.977	0.023
149053315	CAMKK1	calcium/calmodulin dependent protein kinase kinase 1	-0.200	-0.996	0.004
61556891	OSBPL2	oxysterol binding protein like 2	-0.198	-0.978	0.022
17530977	ECHS1	enoyl-CoA hydratase, short chain 1	-0.191	-0.969	0.031
148696094	TUBGCP4	tubulin gamma complex associated protein 4	-0.189	-0.963	0.037
157818171	PLK4	polo like kinase 4	-0.188	-0.955	0.045
68163551	TBC1D22B	TBC1 domain family member 22B	-0.187	-0.966	0.034
564334013	GBF1	golgi brefeldin A resistant guanine nucleotide exchange factor 1	-0.187	-0.985	0.015
157821901	PNMA3	PNMA family member 3	-0.186	-0.986	0.014
672088942	ATP2B3	ATPase plasma membrane Ca2+ transporting 3	-0.186	-0.991	0.009
312283667	WNK1	WNK lysine deficient protein kinase 1	-0.185	-0.993	0.007
197209847	JAK1	Janus kinase 1	-0.185	-0.993	0.007
59891444	FUT10	fucosyltransferase 10	-0.185	-0.972	0.028
16923964	CNTN1	contactin 1	-0.185	-0.995	0.005
76881830	Kcnip2	potassium voltage-gated channel interacting protein 2	-0.183	-0.992	0.008
32185285	BCL2L2	BCL2 like 2	-0.181	-0.967	0.033
157817620	PSD2	pleckstrin and Sec7 domain containing 2	-0.180	-0.995	0.005
12621120	SFXN3	sideroflexin 3	-0.180	-0.968	0.032
187468990	DNAJB2	DnaJ heat shock protein family (Hsp40) member B2	-0.180	-0.987	0.013
293349000	ARHGAP42	Rho GTPase activating protein 42	-0.178	-0.983	0.017
201023331	MAPK11	mitogen-activated protein kinase 11	-0.176	-0.986	0.014
564390898	KIF13A	kinesin family member 13A	-0.176	-0.968	0.032
157818193	TTPAL	alpha tocopherol transfer protein like	-0.176	-0.982	0.018
78000203	Tpm1	tropomyosin 1, alpha	-0.174	-0.996	0.004
564387640	DOCK9	dedicator of cytokinesis 9	-0.172	-0.988	0.012
61557212	CIAO3	cytosolic iron-sulfur assembly component 3	-0.170	-0.997	0.003

817472062	CDK5RAP3	CDK5 regulatory subunit associated protein 3	-0.167	-0.984	0.016
564335900	ZFHX4	zinc finger homeobox 4	-0.167	-0.986	0.014
157817201	NETO1	neuropilin and tolloid like 1	-0.165	-0.982	0.018
6981076	IDE	insulin degrading enzyme	-0.165	-0.958	0.042
13242243	AXIN2	axin 2	-0.164	-0.967	0.033
157819829	HACD3	3-hydroxyacyl-CoA dehydratase 3	-0.159	-0.962	0.038
158186708	PDCD11	programmed cell death 11	-0.159	-0.965	0.035
198278547	TMEM41A	transmembrane protein 41A	-0.159	-0.997	0.003
209529636	PPA2	inorganic pyrophosphatase 2	-0.158	-0.981	0.019
18034785	ABCB6	ATP binding cassette subfamily B member 6 (Langereis blood group)	-0.158	-0.998	0.002
564299821	PARP8	poly(ADP-ribose) polymerase family member 8	-0.155	-0.973	0.027
18266726	PAICS	phosphoribosylaminoimidazole carboxylase and phosphoribosylaminoimidazolesuccinocarboxamide synthase	-0.155	-0.963	0.037
6649914	GDF11	growth differentiation factor 11	-0.154	-0.954	0.046
113461996	COA5	cytochrome c oxidase assembly factor 5	-0.149	-0.993	0.007
50510837	KIAA1191	KIAA1191	-0.148	-0.983	0.017
717324516	SCN8A	sodium voltage-gated channel alpha subunit 8	-0.147	-0.963	0.037
300794996	NDST3	N-deacetylase and N-sulfotransferase 3	-0.147	-0.982	0.018
730229363	RALGAPA1	Ral GTPase activating protein catalytic subunit alpha 1	-0.142	-0.987	0.013
9507043	RGS12	regulator of G protein signaling 12	-0.139	-0.971	0.029
157823401	PIGH	phosphatidylinositol glycan anchor biosynthesis class H	-0.135	-0.982	0.018
564338482	SORT1	sortilin 1	-0.134	-0.967	0.033
6981166	PLAGL1	PLAG1 like zinc finger 1	-0.133	-0.954	0.046
564345487	RINT1	RAD50 interactor 1	-0.132	-0.994	0.006
56605798	RNF167	ring finger protein 167	-0.131	-0.971	0.029
148747541	HNRNPU	heterogeneous nuclear ribonucleoprotein U	-0.130	-0.994	0.006
568985444	CADPS	calcium dependent secretion activator	-0.129	-0.986	0.014
37359832	SCRN1	secernin 1	-0.127	-0.957	0.043
77415383	HSPA8	heat shock protein family A (Hsp70) member 8	-0.125	-0.973	0.027
58865700	GRWD1	glutamate rich WD repeat containing 1	-0.123	-0.993	0.007

157820919	POLE4	DNA polymerase epsilon 4, accessory subunit	-0.123	-0.991	0.009
149064207	COMMD10	COMM domain containing 10	-0.123	-0.963	0.037
253683488	NTRK2	neurotrophic receptor tyrosine kinase 2	-0.121	-0.959	0.041
158254369	CDK10	cyclin dependent kinase 10	-0.121	-0.986	0.014
74229032	TPCN1	two pore segment channel 1	-0.121	-1.000	0.000
476007242	EPS8	epidermal growth factor receptor pathway substrate 8	-0.117	-0.982	0.018
201066352	ANKRD6	ankyrin repeat domain 6	-0.116	-0.962	0.038
8393390	GABRB3	gamma-aminobutyric acid type A receptor subunit beta3	-0.115	-0.994	0.006
29789269	GRIA1	glutamate ionotropic receptor AMPA type subunit 1	-0.115	-0.971	0.029
149059529	LOC100910558	uncharacterized LOC100910558	-0.114	-0.990	0.010
11560079	KIT	KIT proto-oncogene, receptor tyrosine kinase	-0.114	-0.957	0.043
404247435	YLPM1	YLP motif containing 1	-0.113	-0.964	0.036
564353678	USP48	ubiquitin specific peptidase 48	-0.112	-0.991	0.009
694981804	CLDN5	claudin 5	-0.108	-0.972	0.028
89337260	FTO	FTO alpha-ketoglutarate dependent dioxygenase	-0.107	-0.990	0.010
300794843	IQGAP3	IQ motif containing GTPase activating protein 3	-0.100	-0.959	0.041
403377905	SRGAP2	SLIT-ROBO Rho GTPase activating protein 2	-0.098	-0.999	0.001
149060725	CEP19	centrosomal protein 19	-0.095	-0.975	0.025
154800420	GNL3L	G protein nucleolar 3 like	-0.093	-0.971	0.029
8980843	GRIPAP1	GRIP1 associated protein 1	-0.089	-0.979	0.021
54035294	ADH5	alcohol dehydrogenase 5 (class III), chi polypeptide	-0.080	-0.962	0.038
392346010	DNTTIP2	deoxynucleotidyltransferase terminal interacting protein 2	-0.075	-0.951	0.049
297206894	E4F1	E4F transcription factor 1	-0.074	-0.995	0.005
34536836	EHD3	EH domain containing 3	-0.071	-0.955	0.045
564375060	SLC39A11	solute carrier family 39 member 11	-0.070	-0.981	0.019
157816897	UHRF1BP1L	UHRF1 binding protein 1 like	-0.066	-0.953	0.047
148747528	PTK2B	protein tyrosine kinase 2 beta	-0.066	-0.962	0.038
255918181	NUS1	NUS1 dehydrodolichyl diphosphate synthase subunit	-0.062	-0.981	0.019
77020248	PFKFB2	6-phosphofructo-2-kinase/fructose-2,6-biphosphatase 2	-0.060	-0.958	0.042
20302113	STIP1	stress induced phosphoprotein 1	-0.060	-0.954	0.046
408535187	PRDM11	PR/SET domain 11	-0.058	-0.992	0.008

13385318	KDELR2	KDEL endoplasmic reticulum protein retention receptor 2	-0.057	-0.995	0.005
162287208	FADS1	fatty acid desaturase 1	-0.050	-0.962	0.038
189027133	TTC30B	tetratricopeptide repeat domain 30B	-0.044	-0.960	0.040
157820825	IFT57	intraflagellar transport 57	0.053	0.961	0.039
58865796	PTDSS1	phosphatidylserine synthase 1	0.057	0.970	0.030
62078979	AMZ2	archaelysin family metallopeptidase 2	0.061	0.951	0.049
25453374	PEX14	peroxisomal biogenesis factor 14	0.067	0.973	0.027
62089200	ZDHHC9	zinc finger DHHC-type palmitoyltransferase 9	0.068	0.988	0.012
25742568	DPYSL3	dihydropyrimidinase like 3	0.069	0.953	0.047
83649695	SMIM14	small integral membrane protein 14	0.082	0.979	0.021
60360532	OSBPL6	oxysterol binding protein like 6	0.083	0.959	0.041
672074758	NCSTN	nicastrin	0.083	0.979	0.021
149038024	RIPOR1	RHO family interacting cell polarization regulator 1	0.091	0.960	0.040
189491673	FXR2	FMR1 autosomal homolog 2	0.097	0.980	0.020
209447030	DDX27	DEAD-box helicase 27	0.097	0.950	0.050
970596961	MAPK10	mitogen-activated protein kinase 10	0.099	0.990	0.010
166064004	GTF3A	general transcription factor IIIA	0.101	0.984	0.016
42476292	TALDO1	transaldolase 1	0.106	0.955	0.045
157818159	AAR2	AAR2 splicing factor	0.110	0.998	0.002
157786720	HIVEP1	HIVEP zinc finger 1	0.111	0.998	0.002
564360651	LRRC14	leucine rich repeat containing 14	0.115	0.990	0.010
47847438	EXOC3	exocyst complex component 3	0.118	0.981	0.019
68163425	TMEM199	transmembrane protein 199	0.118	0.990	0.010
293344794	FAM160B1	family with sequence similarity 160 member B1	0.121	0.987	0.013
672044181	HS2ST1	heparan sulfate 2-O-sulfotransferase 1	0.124	0.954	0.046
58866022	MGAT4A	alpha-1,3-mannosyl-glycoprotein 4-beta-N-acetylglucosaminyltransferase A	0.127	0.972	0.028
672068548	SUPT6H	SPT6 homolog, histone chaperone and transcription elongation factor	0.127	0.953	0.047
60359978	KIF3C	kinesin family member 3C	0.131	0.999	0.001
148693260	TIMM29	translocase of inner mitochondrial membrane 29	0.131	0.960	0.040
157823607	ALDH18A1	aldehyde dehydrogenase 18 family member A1	0.134	0.981	0.019
61557130	B3GALNT1	beta-1,3-N-acetylgalactosaminyltransferase 1 (globoside blood group)	0.136	0.975	0.025

157821401	UQCC1	ubiquinol-cytochrome c reductase complex assembly factor 1	0.136	0.983	0.017
564398139	FYN	FYN proto-oncogene, Src family tyrosine kinase	0.137	0.961	0.039
58865952	UBAP1	ubiquitin associated protein 1	0.139	0.954	0.046
20301952	SLC2A1	solute carrier family 2 member 1	0.142	0.953	0.047
9624979	ENSA	endosulfine alpha	0.144	0.980	0.020
672024670	INSYN2B	inhibitory synaptic factor family member 2B	0.145	0.995	0.005
157820585	SART3	spliceosome associated factor 3, U4/U6 recycling protein	0.146	0.972	0.028
149064388	Hmgxb3	HMG-box containing 3	0.147	0.988	0.012
148665617	NAA50	N-alpha-acetyltransferase 50, NatE catalytic subunit	0.149	0.964	0.036
157821581	PSMD13	proteasome 26S subunit, non-ATPase 13	0.150	0.994	0.006
40786455	BPGM	bisphosphoglycerate mutase	0.150	0.969	0.031
564311031	CLPP	caseinolytic mitochondrial matrix peptidase proteolytic subunit	0.150	0.980	0.020
208973276	TMEM185A	transmembrane protein 185A	0.151	0.990	0.010
300253233	LEMD3	LEM domain containing 3	0.154	0.979	0.021
66730376	Arxes1/Arxes2	adipocyte-related X-chromosome expressed sequence 2	0.155	0.969	0.031
300793740	TANC2	tetratricopeptide repeat, ankyrin repeat and coiled-coil containing 2	0.158	0.957	0.043
157817763	NEK9	NIMA related kinase 9	0.159	0.956	0.044
404312665	DKK3	dickkopf WNT signaling pathway inhibitor 3	0.160	0.991	0.009
213688411	LPCAT1	lysophosphatidylcholine acyltransferase 1	0.162	0.969	0.031
149033480	Zfp956	zinc finger protein 956	0.163	0.991	0.009
672072928	CUX2	cut like homeobox 2	0.164	0.967	0.033
157822501	MCM3AP	minichromosome maintenance complex component 3 associated protein	0.164	0.999	0.001
564340867	MMADHC	metabolism of cobalamin associated D	0.165	0.959	0.041
76559929	NOC2L	NOC2 like nucleolar associated transcriptional repressor	0.165	0.984	0.016
198278423	IQCJ-SCHIP1	IQCJ-SCHIP1 readthrough	0.166	0.966	0.034
408772026	Afg3l1	AFG3-like AAA ATPase 1	0.166	0.965	0.035
55926133	RFC2	replication factor C subunit 2	0.168	0.976	0.024
157823165	DNAJB1	DnaJ heat shock protein family (Hsp40) member B1	0.171	0.959	0.041

149044496	PLAA	phospholipase A2 activating protein	0.171	0.960	0.040
21489987	PCYOX1	prenylcysteine oxidase 1	0.172	0.971	0.029
33356154	UBE2H	ubiquitin conjugating enzyme E2 H	0.172	0.996	0.004
56605790	HCFC2	host cell factor C2	0.173	0.995	0.005
119569672	BUB3	BUB3 mitotic checkpoint protein	0.176	0.955	0.045
164565387	TBC1D14	TBC1 domain family member 14	0.177	0.952	0.048
149054120	ORMDL3	ORMDL sphingolipid biosynthesis regulator 3	0.179	0.971	0.029
77695933	NELL2	neural EGFL like 2	0.180	0.953	0.047
61556927	EIF3G	eukaryotic translation initiation factor 3 subunit G	0.180	0.980	0.020
62079005	SLAIN1	SLAIN motif family member 1	0.180	0.998	0.002
6981296	NUP50	nucleoporin 50	0.181	0.975	0.025
157817773	ZNF641	zinc finger protein 641	0.183	0.962	0.038
401709959	Ppp1cc	protein phosphatase 1 catalytic subunit gamma	0.183	0.982	0.018
9507007	PTGFRN	prostaglandin F2 receptor inhibitor	0.184	0.997	0.003
58865626	UBXN4	UBX domain protein 4	0.186	0.966	0.034
564384443	EIF4ENIF1	eukaryotic translation initiation factor 4E nuclear import factor 1	0.187	0.985	0.015
167555101	STRADB	STE20 related adaptor beta	0.188	0.965	0.035
148670058	PRMT6	protein arginine methyltransferase 6	0.188	0.969	0.031
66730335	SUMO3	small ubiquitin like modifier 3	0.188	0.986	0.014
149047323	ZNF518B	zinc finger protein 518B	0.190	0.977	0.023
672085227	USP10	ubiquitin specific peptidase 10	0.191	0.985	0.015
148683335	SLC25A44	solute carrier family 25 member 44	0.191	0.977	0.023
197313795	MTX1	metaxin 1	0.192	0.998	0.002
62079229	PDSS2	decaprenyl diphosphate synthase subunit 2	0.193	0.964	0.036
296489017	BEND5	BEN domain containing 5	0.196	0.987	0.013
157821953	NXPE3	neurexophilin and PC-esterase domain family member 3	0.197	0.956	0.044
213511844	ALG2	ALG2 alpha-1,3/1,6-mannosyltransferase	0.200	0.999	0.001
219277692	NDUFB2	NADH:ubiquinone oxidoreductase subunit B2	0.203	0.998	0.002
672046840	UBOX5	U-box domain containing 5	0.206	0.969	0.031
157817674	ATP5MF-PTCD1	ATP5MF-PTCD1 readthrough	0.207	0.987	0.013
40018540	DDX24	DEAD-box helicase 24	0.208	0.991	0.009
61557082	TERF2IP	TERF2 interacting protein	0.211	0.978	0.022
61556748	TSPYL1	TSPY like 1	0.211	0.975	0.025
58865624	NUF2	NUF2 component of NDC80 kinetochore complex	0.214	0.965	0.035
639869	CHKA	choline kinase alpha	0.216	0.963	0.037

189163477	SCAF4	SR-related CTD associated factor 4	0.216	0.963	0.037
24025618	DAB1	DAB adaptor protein 1	0.217	0.957	0.043
24638440	RIMS4	regulating synaptic membrane exocytosis 4	0.220	0.959	0.041
2804296	CDH8	cadherin 8	0.221	0.962	0.038
148669751	SMNDC1	survival motor neuron domain containing 1	0.223	0.991	0.009
197252056	MED1	mediator complex subunit 1	0.223	0.964	0.036
58865962	RNF41	ring finger protein 41	0.231	0.969	0.031
148670791	ZFYVE1	zinc finger FYVE-type containing 1	0.232	0.999	0.001
798974764	SRRD	SRR1 domain containing	0.233	0.966	0.034
564340133	GTF3C4	general transcription factor IIIC subunit 4	0.236	0.998	0.002
37360264	TRMT6	tRNA methyltransferase 6	0.236	0.983	0.017
19424174	DNPH1	2'-deoxynucleoside 5'-phosphate N-hydrolase 1	0.243	0.961	0.039
57527612	SLC17A5	solute carrier family 17 member 5	0.245	0.968	0.032
158631258	KDSR	3-ketodihydrosphingosine reductase	0.249	0.967	0.033
291042683	DCAF5	DDB1 and CUL4 associated factor 5	0.252	0.989	0.011
166157540	TMEM222	transmembrane protein 222	0.253	0.961	0.039
57164019	B4GALT3	beta-1,4-galactosyltransferase 3	0.256	0.962	0.038
71361655	MRPL12	mitochondrial ribosomal protein L12	0.257	0.972	0.028
77627757	IQUB	IQ motif and ubiquitin domain containing	0.257	0.996	0.004
119388826	TFPT	TCF3 fusion partner	0.258	1.000	0.000
67078454	SLC25A51	solute carrier family 25 member 51	0.263	0.969	0.031
380877082	NAXE	NAD(P)HX epimerase	0.263	0.963	0.037
148687213	COX19	cytochrome c oxidase assembly factor COX19	0.264	0.966	0.034
404312698	GOLM2	golgi membrane protein 2	0.270	0.984	0.016
564394925	TENT4B	terminal nucleotidyltransferase 4B	0.276	0.994	0.006
59937915	ARIH2	ariadne RBR E3 ubiquitin protein ligase 2	0.280	0.991	0.009
157821915	MSANTD3	Myb/SANT DNA binding domain containing 3	0.283	0.980	0.020
149052738	RGD1561277	RGD1561277	0.285	0.961	0.039
62078733	MAK16	MAK16 homolog	0.287	0.992	0.008
672020915	VCPKMT	valosin containing protein lysine methyltransferase	0.288	1.000	0.000
51948506	AK8	adenylate kinase 8	0.290	0.953	0.047
672053062	FKBP15	FKBP prolyl isomerase 15	0.290	0.971	0.029
76559919	N4BP3	NEDD4 binding protein 3	0.292	0.965	0.035
149031601	H1f2	H1.2 linker histone, cluster member	0.294	0.982	0.018
68163385	GPATCH4	G-patch domain containing 4	0.297	0.998	0.002

157821997	MED28	mediator complex subunit 28	0.297	0.985	0.015
74220037	FAM107B	family with sequence similarity 107 member B	0.299	0.998	0.002
157822367	PUS3	pseudouridine synthase 3	0.301	0.988	0.012
7339838	SUV39H1	suppressor of variegation 3-9 homolog 1	0.302	0.963	0.037
157819315	OSBPL11	oxysterol binding protein like 11	0.306	0.971	0.029
300798436	NME6	NME/NM23 nucleoside diphosphate kinase 6	0.308	0.968	0.032
51491900	TOR1A	torsin family 1 member A	0.308	0.980	0.020
349501022	2410002F23Rik	RIKEN cDNA 2410002F23 gene	0.309	0.966	0.034
109480728	TMEM74	transmembrane protein 74	0.313	0.958	0.042
62078923	DZIP1L	DAZ interacting zinc finger protein 1 like	0.315	0.954	0.046
300793780	ZNF251	zinc finger protein 251	0.321	0.976	0.024
672030183	H2AC12	H2A clustered histone 12	0.323	0.972	0.028
68163537	NXPE4	neurexophilin and PC-esterase domain family member 4	0.329	0.986	0.014
564333920	PPRC1	PPARG related coactivator 1	0.333	0.988	0.012
156627555	NT5C3B	5'-nucleotidase, cytosolic IIIB	0.335	0.975	0.025
6680007	GJC1	gap junction protein gamma 1	0.336	0.951	0.049
73990974	LZTS3	leucine zipper tumor suppressor family member 3	0.345	0.990	0.010
141803183	ZKSCAN3	zinc finger with KRAB and SCAN domains 3	0.351	0.982	0.018
281604129	HELQ	helicase, POLQ like	0.354	0.952	0.048
148673748	FAM110B	family with sequence similarity 110 member B	0.357	0.993	0.007
88853859	UBE3D	ubiquitin protein ligase E3D	0.361	0.956	0.044
157822027	CSRNP2	cysteine and serine rich nuclear protein 2	0.361	0.972	0.028
300794219	OPN3	opsin 3	0.362	0.951	0.049
300797828	KAT14	lysine acetyltransferase 14	0.365	0.990	0.010
148710078	TAF5	TATA-box binding protein associated factor 5	0.366	0.990	0.010
238859603	ISLR2	immunoglobulin superfamily containing leucine rich repeat 2	0.369	0.966	0.034
157821875	PTCD2	pentatricopeptide repeat domain 2	0.372	0.964	0.036
672036551	ZDHHC13	zinc finger DHHC-type palmitoyltransferase 13	0.373	0.962	0.038
157817720	SLC16A14	solute carrier family 16 member 14	0.374	0.986	0.014
157821747	MDM2	MDM2 proto-oncogene	0.377	0.972	0.028
164565364	ITPKB	inositol-trisphosphate 3-kinase B	0.380	0.998	0.002
148681067	VASH2	vasohibin 2	0.397	0.965	0.035

157821403	RASSF7	Ras association domain family member 7	0.399	0.970	0.030
282158061	Ttc41	tetratricopeptide repeat domain 41	0.404	0.985	0.015
62945262	PIK3IP1	phosphoinositide-3-kinase interacting protein 1	0.404	0.977	0.023
293345066	PPIL6	peptidylprolyl isomerase like 6	0.408	0.960	0.040
226371633	CABLES1	Cdk5 and Abl enzyme substrate 1	0.409	0.976	0.024
66730347	PTPRCAP	protein tyrosine phosphatase receptor type C associated protein	0.415	0.981	0.019
293348214	CCDC88C	coiled-coil domain containing 88C	0.421	0.963	0.037
564316243	CEP170	centrosomal protein 170	0.430	0.950	0.050
56090305	NFATC2IP	nuclear factor of activated T cells 2 interacting protein	0.430	1.000	0.000
56090289	PELO	pelota mRNA surveillance and ribosome rescue factor	0.432	0.974	0.026
157823891	ING2	inhibitor of growth family member 2	0.439	0.991	0.009
564311452	TMEM131	transmembrane protein 131	0.443	0.962	0.038
51980294	COQ3	coenzyme Q3, methyltransferase	0.452	0.979	0.021
157820727	RPL27A	ribosomal protein L27a	0.452	0.968	0.032
53850630	LOC100362724/ MGC95208	similar to 4930453N24Rik protein	0.456	0.982	0.018
148706598	PKDCC	protein kinase domain containing, cytoplasmic	0.469	0.989	0.011
51948492	NUDT19	nudix hydrolase 19	0.470	0.984	0.016
70608121	Dmrtc1a	DMRT-like family C1a	0.479	0.975	0.025
149024753	DFFB	DNA fragmentation factor subunit beta	0.487	0.991	0.009
212549645	KIF18A	kinesin family member 18A	0.505	0.970	0.030
19424300	GCHFR	GTP cyclohydrolase I feedback regulator	0.519	0.979	0.021
148687591	TMEM132D	transmembrane protein 132D	0.550	0.988	0.012
219879771	PGAP3	post-GPI attachment to proteins phospholipase 3	0.560	0.973	0.027
149023178	CEP152	centrosomal protein 152	0.572	0.999	0.001
564372912	GPS2	G protein pathway suppressor 2	0.577	0.993	0.007
148664537	Gm10269	ribosomal protein L35 pseudogene	0.582	0.981	0.019
555290059	MED7	mediator complex subunit 7	0.584	0.990	0.010
511094004	RUNX2	RUNX family transcription factor 2	0.585	0.961	0.039
24415396	GPR3	G protein-coupled receptor 3	0.604	0.961	0.039
157822359	PELI2	pellino E3 ubiquitin protein ligase family member 2	0.620	0.968	0.032
197386066	ZNF784	zinc finger protein 784	0.633	0.976	0.024
157823803	DOK3	docking protein 3	0.639	0.996	0.004
149016574	ZNF324	zinc finger protein 324	0.689	0.969	0.031

348041347	CENPL	centromere protein L	0.708	0.970	0.030
392354293	Hmgb3	high mobility group box 3	0.709	0.975	0.025
672070295	BAHCC1	BAH domain and coiled-coil containing 1	0.769	0.957	0.043
672031995	Kdm6a	lysine demethylase 6A	0.794	0.970	0.030
564297423	FAM71E1	family with sequence similarity 71 member E1	0.803	0.960	0.040
564377118	WDR53	WD repeat domain 53	0.807	0.992	0.008
294979130	FOXP3	forkhead box P3	0.830	0.975	0.025
672086719	FAM184A	family with sequence similarity 184 member A	0.836	0.990	0.010
26024223	ABCG5	ATP binding cassette subfamily G member 5	0.841	0.976	0.024
9506775	HES2	hes family bHLH transcription factor 2	0.963	0.961	0.039
148693657	DDX6	DEAD-box helicase 6	0.969	0.950	0.050
148235584	CLEC4A	C-type lectin domain family 4 member A	1.000	0.961	0.039
149065466	ARHGEF5	Rho guanine nucleotide exchange factor 5	1.000	0.961	0.039
82654234	LILRA6	leukocyte immunoglobulin like receptor A6	1.000	0.990	0.010
404501522	NXNL1	nucleoredoxin like 1	1.037	0.961	0.039
148674299	Gm14176	ubiquitin-conjugating enzyme E2I pseudogene	1.041	0.997	0.003
149067796	TMEM219	transmembrane protein 219	1.064	0.996	0.004
564296988	ZNF235	zinc finger protein 235	1.072	0.962	0.038
188536090	FAM241B	family with sequence similarity 241 member B	1.127	0.984	0.016
62078917	PAQR5	progestin and adipoQ receptor family member 5	1.181	0.964	0.036
6978525	FASLG	Fas ligand	1.186	0.956	0.044
149045964	PTH2R	parathyroid hormone 2 receptor	1.222	0.977	0.023
564317923	SACS	sacsin molecular chaperone	1.308	0.985	0.015
13928980	AQP3	aquaporin 3 (Gill blood group)	1.585	0.952	0.048
158533972	SPTA1	spectrin alpha, erythrocytic 1	1.585	0.961	0.039
149058209	SELE	selectin E	1.585	0.961	0.039
58865680	CES5A	carboxylesterase 5A	1.585	0.978	0.022
24308466	ITGB3	integrin subunit beta 3	1.596	0.994	0.006
157818463	Zfp93	zinc finger protein 93	1.597	0.999	0.001
672013187	DMWD	DM1 locus, WD repeat containing	1.605	0.986	0.014
568990288	NIPBL	NIPBL cohesin loading factor	1.705	0.966	0.034
157816947	GUCA1B	guanylate cyclase activator 1B	1.708	0.963	0.037
157820841	GP1BA	glycoprotein Ib platelet subunit alpha	1.726	0.964	0.036

569001477	MTCL1	microtubule crosslinking factor 1	1.726	0.976	0.024
149046722	IBSP	integrin binding sialoprotein	1.737	0.967	0.033
149032888	LOC100910237	uncharacterized LOC100910237	1.751	0.989	0.011
157820135	CHRDL2	chordin like 2	1.762	0.990	0.010
157816967	Gm4925	predicted gene 4925	1.781	0.957	0.043
392342449	PRSS56	serine protease 56	1.848	0.980	0.020
16758254	CNGA1	cyclic nucleotide gated channel subunit alpha 1	1.874	0.979	0.021
189181736	LAD1	ladinin 1	1.874	0.988	0.012
109488483	KIAA0753	KIAA0753	1.905	0.967	0.033
197381585	Urah	urate (5-hydroxyiso-) hydrolase	1.976	0.961	0.039
160961485	MYLK3	myosin light chain kinase 3	2.000	0.961	0.039
568979594	SYT16	synaptotagmin 16	2.059	0.987	0.013
672070295	BAHCC1	BAH domain and coiled-coil containing 1	2.083	0.962	0.038
28972866	CSMD3	CUB and Sushi multiple domains 3	2.140	0.981	0.019
568974167	SLC26A11	solute carrier family 26 member 11	2.149	0.980	0.020
564297852	CRTC3	CREB regulated transcription coactivator 3	2.151	0.991	0.009
293352381	PAN3	poly(A) specific ribonuclease subunit PAN3	2.239	0.975	0.025
56912237	KRT28	keratin 28	2.241	0.980	0.020
71896592	IGFALS	insulin like growth factor binding protein acid labile subunit	2.322	0.961	0.039
8393941	PADI4	peptidyl arginine deiminase 4	2.322	0.961	0.039
157819659	RRH	retinal pigment epithelium-derived rhodopsin homolog	2.447	0.968	0.032
564329376	SRPK3	SRSF protein kinase 3	2.585	0.961	0.039
58866038	XKRX	XK related X-linked	2.585	0.961	0.039
11120690	NR1H4	nuclear receptor subfamily 1 group H member 4	2.585	0.961	0.039
13540693	MYOC	myocilin	2.585	0.961	0.039
57222314	OAS3	2'-5'-oligoadenylate synthetase 3	2.585	0.961	0.039
8394529	VDR	vitamin D receptor	2.585	0.961	0.039
25742760	AMH	anti-Mullerian hormone	2.807	0.961	0.039
157787002	Dpt	dermatopontin	2.807	0.961	0.039
197384923	C1orf87	chromosome 1 open reading frame 87	3.000	0.961	0.039
13591993	MMP9	matrix metallopeptidase 9	3.000	0.961	0.039
300796937	ESPNL	espin like	3.000	0.961	0.039
564347547	LOC103690120	probable N-acetyltransferase CML1	3.030	0.952	0.048
281332212	SH2D4B	SH2 domain containing 4B	3.170	0.961	0.039
260099641	MSH5	mutS homolog 5	3.170	0.961	0.039
61556961	THEG	theg spermatid protein	3.322	0.961	0.039
28174920	RPL17	ribosomal protein L17	3.389	0.990	0.010

25282405	BPIFA1	BPI fold containing family A member 1	3.459	0.961	0.039
16758550	BCL2L10	BCL2 like 10	3.459	0.961	0.039
299473749	C1orf226	chromosome 1 open reading frame 226	3.496	0.970	0.030
148670929	BATF	basic leucine zipper ATF-like transcription factor	3.700	0.961	0.039
21245088	Ly6a (includes others)	lymphocyte antigen 6 complex, locus A	3.807	0.961	0.039
148747510	BAAT	bile acid-CoA:amino acid N-acyltransferase	3.807	0.961	0.039
158187515	OAZ3	ornithine decarboxylase antizyme 3	3.807	0.961	0.039
23463315	Cyp2d1/Cyp2d5	cytochrome P450, family 2, subfamily d, polypeptide 1	4.170	0.951	0.049
8393641	AADAT	aminoadipate aminotransferase	4.248	0.961	0.039
149038931	CNTRL	centriolin	4.492	0.965	0.035
149034317	NUCKS1	nuclear casein kinase and cyclin dependent kinase substrate 1	4.833	0.958	0.042
9506733	GJB5	gap junction protein beta 5	4.907	0.961	0.039
293349510	STAC	SH3 and cysteine rich domain	4.954	0.951	0.049
16758218	Hamp	hepcidin antimicrobial peptide	5.267	0.959	0.041
157818205	NOC3L	NOC3 like DNA replication regulator	5.833	0.959	0.041
672052120	RBM12B	RNA binding motif protein 12B	6.366	0.962	0.038
293347435	PTPRD	protein tyrosine phosphatase receptor type D	7.710	0.951	0.049
109472884	UBE3C	ubiquitin protein ligase E3C	8.197	0.955	0.045

Supplementary Table S18. The list of genes that are differentially expressed in the offspring hippocampus in response to prenatal BPA exposure that exhibited the changes in the expression levels correlated with the NOR discrimination index of the rat offspring. The transcriptome profiling data of DEGs in male and female rat offspring prenatally exposed to BPA (n = 6, male pups n = 3 and female pups n = 3, from independent litters) or the vehicle control (n = 6, male pups n = 3 and female pups n = 3, from independent litters) were obtained and used for the PTM analyses to identify DEGs that exhibited the changes in the expression levels correlated with the NOR discrimination index of the rat offspring.

ID	Symbol	Entrez Gene Name	log2(FC)	R values	P-values
157822121	LRMDA	leucine rich melanocyte differentiation associated	-2.747	0.971	0.029
672032217	REPS2	RALBP1 associated Eps domain containing 2	-2.683	0.977	0.023
564297387	Zfp658	zinc finger protein 658	-2.369	0.960	0.040
157820151	ERAS	ES cell expressed Ras	-2.248	0.992	0.008
564320724	Fbxo38	F-box protein 38	-2.220	0.997	0.003
300797305	TMEM45A	transmembrane protein 45A	-2.093	0.980	0.020
56676350	PRSS35	serine protease 35	-1.962	0.979	0.021
157820217	Gsta4	glutathione S-transferase, alpha 4	-1.930	0.996	0.004
13592031	PTGER2	prostaglandin E receptor 2	-1.845	0.957	0.043
297374767	TPSAB1/TPSB2	tryptase alpha/beta 1	-1.834	0.963	0.037
569009290	TENM1	teneurin transmembrane protein 1	-1.824	0.999	0.001
149064260	PRDM6	PR/SET domain 6	-1.816	0.973	0.027
226698394	UNC80	unc-80 homolog, NALCN channel complex subunit	-1.781	0.950	0.050
157819477	GLOD5	glyoxalase domain containing 5	-1.768	0.994	0.006
153792385	Vom2r34	vomeronasal 2 receptor, 34	-1.648	0.963	0.037
672019578	MYSM1	Myb like, SWIRM and MPN domains 1	-1.645	0.980	0.020
58331159	GSTA3	glutathione S-transferase alpha 3	-1.603	0.971	0.029
157819393	NNMT	nicotinamide N-methyltransferase	-1.585	0.975	0.025
564378315	Zfp853	zinc finger protein 853	-1.574	0.986	0.014
6978837	FGF10	fibroblast growth factor 10	-1.548	0.968	0.032
16758884	Cd52	CD52 antigen	-1.546	0.966	0.034
6981326	S100A4	S100 calcium binding protein A4	-1.532	0.968	0.032
72255569	Abca17	ATP-binding cassette, sub-family A (ABC1), member 17	-1.495	0.982	0.018
94400795	PRLHR	prolactin releasing hormone receptor	-1.474	0.960	0.040
71043750	SYNGR4	synaptogyrin 4	-1.464	0.991	0.009
157823445	PABPC4L	poly(A) binding protein cytoplasmic 4 like	-1.453	0.970	0.030
31377521	S1PR5	sphingosine-1-phosphate receptor 5	-1.426	0.953	0.047

157820935	Prss32	protease, serine 32	-1.392	0.964	0.036
157822105	SLC49A3	solute carrier family 49 member 3	-1.389	0.966	0.034
18426832	IL23A	interleukin 23 subunit alpha	-1.381	0.993	0.007
672025117	MBTD1	mbt domain containing 1	-1.344	0.991	0.009
568950651	CYP2R1	cytochrome P450 family 2 subfamily R member 1	-1.277	0.960	0.040
71043878	PROCR	protein C receptor	-1.270	0.952	0.048
16758338	FTCD	formimidoyltransferase cyclodeaminase	-1.208	0.999	0.001
218505769	Zfp7	zinc finger protein 7	-1.191	0.990	0.010
392334475	Myb	MYB proto-oncogene, transcription factor	-1.175	0.953	0.047
281332082	THBS2	thrombospondin 2	-1.164	0.963	0.037
60223053	SEPTIN1	septin 1	-1.133	0.978	0.022
157818961	UBA7	ubiquitin like modifier activating enzyme 7	-1.078	0.992	0.008
197386139	SSC5D	scavenger receptor cysteine rich family member with 5 domains	-1.060	0.975	0.025
11560040	PTGDR	prostaglandin D2 receptor	-1.037	0.978	0.022
157821423	TBX6	T-box transcription factor 6	-1.017	0.956	0.044
158341649	FAM227B	family with sequence similarity 227 member B	-0.981	0.997	0.003
215276950	PKP2	plakophilin 2	-0.973	0.988	0.012
157818989	LRRC71	leucine rich repeat containing 71	-0.952	0.985	0.015
157823055	EGFL6	EGF like domain multiple 6	-0.947	0.979	0.021
157819487	TACO1	translational activator of cytochrome c oxidase I	-0.917	0.953	0.047
47059114	LTB	lymphotoxin beta	-0.916	0.990	0.010
564325648	Zfp54	zinc finger protein 54	-0.907	0.976	0.024
564318923	WDR17	WD repeat domain 17	-0.904	0.960	0.040
564312627	ZFP62	ZFP62 zinc finger protein	-0.871	0.978	0.022
57114338	SCN4B	sodium voltage-gated channel beta subunit 4	-0.859	0.958	0.042
149031998	ACVRL1	activin A receptor like type 1	-0.852	0.951	0.049
157822283	HS3ST6	heparan sulfate-glucosamine 3-sulfotransferase 6	-0.835	0.994	0.006
564387882	IL17RB	interleukin 17 receptor B	-0.835	0.989	0.011
157822457	SYNC	syncoilin, intermediate filament protein	-0.833	0.999	0.001
564373460	SLFN13	schlafen family member 13	-0.831	0.970	0.030
149038682	Srgn	serglycin	-0.823	0.971	0.029
259089426	AGER	advanced glycosylation end-product specific receptor	-0.804	0.971	0.029

402692079	PFKFB1	6-phosphofructo-2-kinase/fructose-2,6-biphosphatase 1	-0.788	0.993	0.007
307548437	NYAP2	neuronal tyrosine-phosphorylated phosphoinositide-3-kinase adaptor 2	-0.780	0.962	0.038
942523340	CAPRIN2	caprin family member 2	-0.774	0.957	0.043
157819467	C19orf71	chromosome 19 open reading frame 71	-0.769	0.977	0.023
56090397	CYB5D2	cytochrome b5 domain containing 2	-0.764	0.955	0.045
27465577	Cyp4f16/Cyp4f37	cytochrome P450, family 4, subfamily f, polypeptide 16	-0.758	0.977	0.023
71361637	STRA6	signaling receptor and transporter of retinol STRA6	-0.757	0.995	0.005
672069802	C1QTNF1	C1q and TNF related 1	-0.749	0.960	0.040
12621132	FAT2	FAT atypical cadherin 2	-0.737	0.969	0.031
56119147	ARRDC3	arrestin domain containing 3	-0.732	1.000	0.000
16758232	PLCB2	phospholipase C beta 2	-0.721	0.959	0.041
148669751	SMNDC1	survival motor neuron domain containing 1	-0.717	0.998	0.002
149062459	MS4A2	membrane spanning 4-domains A2	-0.716	0.961	0.039
293343546	C5orf49	chromosome 5 open reading frame 49	-0.693	0.970	0.030
564317714	Ktn1	kinectin 1	-0.687	0.970	0.030
149066381	DSCC1	DNA replication and sister chromatid cohesion 1	-0.687	0.955	0.045
300794452	PTPRH	protein tyrosine phosphatase receptor type H	-0.687	0.986	0.014
58866014	CATSPER2	cation channel sperm associated 2	-0.664	0.982	0.018
70912395	CFAP20DC	CFAP20 domain containing	-0.659	0.990	0.010
201861690	TPK1	thiamin pyrophosphokinase 1	-0.657	0.954	0.046
157824216	RRAS	RAS related	-0.655	0.996	0.004
9845261	LGALS1	galectin 1	-0.655	0.999	0.001
157819783	IRF6	interferon regulatory factor 6	-0.648	0.990	0.010
564316927	FRYL	FRY like transcription coactivator	-0.643	0.976	0.024
13786136	PDGFC	platelet derived growth factor C	-0.642	0.955	0.045
293340128	MIEF2	mitochondrial elongation factor 2	-0.642	0.974	0.026
34734058	HCK	HCK proto-oncogene, Src family tyrosine kinase	-0.631	0.976	0.024
157818465	P2ry10b	purinergic receptor P2Y, G-protein coupled 10B	-0.624	0.965	0.035
402478640	HTRA3	HtrA serine peptidase 3	-0.608	0.973	0.027
672014740	MAMDC2	MAM domain containing 2	-0.603	0.989	0.011
6978737	CYP1B1	cytochrome P450 family 1 subfamily B member 1	-0.593	0.971	0.029
567316103	Ac1576	uncharacterized LOC102552783	-0.587	0.988	0.012
56090245	TCP11	t-complex 11	-0.585	0.956	0.044

300793858	PARP14	poly(ADP-ribose) polymerase family member 14	-0.585	0.975	0.025
148690852	FCGRT	Fc fragment of IgG receptor and transporter	-0.573	0.953	0.047
564366772	MGC116197 (includes others)	similar to RIKEN cDNA 1700001E04	-0.569	0.951	0.049
55741859	XRCC4	X-ray repair cross complementing 4	-0.569	0.988	0.012
62078887	CLEC14A	C-type lectin domain containing 14A	-0.564	0.990	0.010
114145407	CNTNAP5	contactin associated protein family member 5	-0.563	0.993	0.007
75832150	GALNT3	polypeptide N-acetylgalactosaminyltransferase 3	-0.557	0.977	0.023
9507045	RGS5	regulator of G protein signaling 5	-0.538	0.983	0.017
11560101	GCNT1	glucosaminyl (N-acetyl) transferase 1	-0.537	0.967	0.033
13929182	VAMP8	vesicle associated membrane protein 8	-0.522	0.986	0.014
293348472	ZFR2	zinc finger RNA binding protein 2	-0.516	0.952	0.048
62078773	CCDC81	coiled-coil domain containing 81	-0.511	0.976	0.024
154937382	MYL9	myosin light chain 9	-0.510	0.996	0.004
157816963	IRF4	interferon regulatory factor 4	-0.503	0.951	0.049
281371499	COL5A2	collagen type V alpha 2 chain	-0.495	0.983	0.017
157822847	PSMB11	proteasome subunit beta 11	-0.495	0.961	0.039
157822759	PARP2	poly(ADP-ribose) polymerase 2	-0.493	1.000	0.000
41386749	PCLAF	PCNA clamp associated factor	-0.492	0.988	0.012
392333084	CC2D2A	coiled-coil and C2 domain containing 2A	-0.483	0.982	0.018
395759219	AQP4	aquaporin 4	-0.482	0.952	0.048
56090459	CNDP1	carnosine dipeptidase 1	-0.479	0.991	0.009
293340174	DNAH9	dynein axonemal heavy chain 9	-0.479	0.954	0.046
225007623	TCFL5	transcription factor like 5	-0.472	0.964	0.036
41056215	XRCC5	X-ray repair cross complementing 5	-0.466	0.971	0.029
149031202	Saysd1	SAYSVFN motif domain containing 1	-0.464	0.986	0.014
56605808	CENPN	centromere protein N	-0.462	0.987	0.013
68163403	SLC46A3	solute carrier family 46 member 3	-0.458	0.960	0.040
219275548	DUSP19	dual specificity phosphatase 19	-0.458	0.974	0.026
157821107	MYO1F	myosin IF	-0.456	0.966	0.034
157817743	CDH5	cadherin 5	-0.454	0.951	0.049
149028405	EBP	EBP cholestenol delta-isomerase	-0.454	0.960	0.040
13027400	GUCY1A2	guanylate cyclase 1 soluble subunit alpha 2	-0.453	0.976	0.024
157073937	PARP9	poly(ADP-ribose) polymerase family member 9	-0.443	0.951	0.049
149022245	SCRN3	secernin 3	-0.437	0.963	0.037
68534547	NUDT18	nudix hydrolase 18	-0.437	0.990	0.010

61889119	TNFSF12	TNF superfamily member 12	-0.432	0.971	0.029
157822677	LGI3	leucine rich repeat LGI family member 3	-0.431	0.977	0.023
13928796	PXMP2	peroxisomal membrane protein 2	-0.426	0.974	0.026
300796997	ARHGAP28	Rho GTPase activating protein 28	-0.423	0.961	0.039
195973006	EGFLAM	EGF like, fibronectin type III and laminin G domains	-0.403	0.992	0.008
56605714	NDUFAF7	NADH:ubiquinone oxidoreductase complex assembly factor 7	-0.399	0.955	0.045
157819229	RPA3	replication protein A3	-0.393	0.998	0.002
187937124	TMEM126B	transmembrane protein 126B	-0.389	0.956	0.044
166999225	GRM1	glutamate metabotropic receptor 1	-0.385	0.983	0.017
6981210	MME	membrane metalloendopeptidase	-0.382	0.968	0.032
149034469	GNG7	G protein subunit gamma 7	-0.381	0.980	0.020
189011677	GLB1L	galactosidase beta 1 like	-0.373	0.951	0.049
929981595	NPHP1	nephrocystin 1	-0.372	0.961	0.039
157824002	ATG10	autophagy related 10	-0.372	1.000	0.000
564299653	FAM169A	family with sequence similarity 169 member A	-0.372	0.960	0.040
672087893	Dmrtc1b	DMRT-like family C1b	-0.371	0.958	0.042
198386343	TRPS1	transcriptional repressor GATA binding 1	-0.370	0.960	0.040
564357619	ITGB8	integrin subunit beta 8	-0.366	0.978	0.022
55741549	MRPL13	mitochondrial ribosomal protein L13	-0.360	0.974	0.026
149066868	MDM1	Mdm1 nuclear protein	-0.357	0.970	0.030
564331077	HIRIP3	HIRA interacting protein 3	-0.352	0.991	0.009
157823944	SUSD2	sushi domain containing 2	-0.351	0.969	0.031
56605656	DONSON	DNA replication fork stabilization factor DONSON	-0.350	0.995	0.005
451172073	CHRM3	cholinergic receptor muscarinic 3	-0.349	0.989	0.011
149048968	ITPR2	inositol 1,4,5-trisphosphate receptor type 2	-0.348	0.993	0.007
16758580	AURKB	aurora kinase B	-0.343	0.971	0.029
300795679	CD84	CD84 molecule	-0.338	0.995	0.005
672038342	XYLT1	xylosyltransferase 1	-0.329	0.965	0.035
564393980	ME2	malic enzyme 2	-0.327	0.960	0.040
149066394	SAMD12	sterile alpha motif domain containing 12	-0.325	0.991	0.009
31377530	RASGRP1	RAS guanyl releasing protein 1	-0.324	0.989	0.011
62078809	TNFAIP8L2	TNF alpha induced protein 8 like 2	-0.324	0.984	0.016
45267819	CAV2	caveolin 2	-0.321	0.995	0.005
451172111	HINT3	histidine triad nucleotide binding protein 3	-0.321	0.972	0.028

672063876	MGC116197 (includes others)	similar to RIKEN cDNA 1700001E04	-0.320	0.973	0.027
564343903	DSN1	DSN1 component of MIS12 kinetochore complex	-0.317	0.985	0.015
564336403	EXOSC8	exosome component 8	-0.309	0.957	0.043
149063353	IFT81	intraflagellar transport 81	-0.309	0.977	0.023
61556910	SNX10	sorting nexin 10	-0.306	0.977	0.023
31982028	RSU1	Ras suppressor protein 1	-0.304	0.988	0.012
564376043	PIGP	phosphatidylinositol glycan anchor biosynthesis class P	-0.302	0.968	0.032
564397303	CCDC167	coiled-coil domain containing 167	-0.297	0.989	0.011
38259192	TOP2A	DNA topoisomerase II alpha	-0.297	0.987	0.013
160961483	Serinc4	serine incorporator 4	-0.297	0.983	0.017
162287198	HSD17B4	hydroxysteroid 17-beta dehydrogenase 4	-0.297	0.962	0.038
672035779	Proser3	proline and serine rich 3	-0.293	0.960	0.040
28972363	DOCK4	dedicator of cytokinesis 4	-0.292	0.972	0.028
27465571	BRINP3	BMP/retinoic acid inducible neural specific 3	-0.287	0.979	0.021
402765953	0610009B22Rik	RIKEN cDNA 0610009B22 gene	-0.287	0.999	0.001
8394443	TFPI	tissue factor pathway inhibitor	-0.286	0.980	0.020
21489989	KCNH8	potassium voltage-gated channel subfamily H member 8	-0.282	0.991	0.009
157823996	ELK3	ETS transcription factor ELK3	-0.282	0.950	0.050
392338823	TIPARP	TCDD inducible poly(ADP-ribose) polymerase	-0.281	0.968	0.032
197313643	GLTP	glycolipid transfer protein	-0.280	0.997	0.003
60097941	HP	haptoglobin	-0.279	0.960	0.040
56090313	MOCS2	molybdenum cofactor synthesis 2	-0.275	0.969	0.031
197333840	CAMKMT	calmodulin-lysine N-methyltransferase	-0.270	0.955	0.045
149068830	SLCO2B1	solute carrier organic anion transporter family member 2B1	-0.270	0.959	0.041
148701892	EBF1	EBF transcription factor 1	-0.270	0.975	0.025
66730535	Armcx1/LOC102554790	armadillo repeat containing, X-linked 1	-0.270	0.964	0.036
576796148	MAP7D2	MAP7 domain containing 2	-0.269	0.983	0.017
197313676	AIG1	androgen induced 1	-0.268	0.973	0.027
672033256	LOC100912904	disks large homolog 5-like	-0.267	0.979	0.021
52138628	RAP1B	RAP1B, member of RAS oncogene family	-0.267	0.954	0.046
148703340	SERTM1	serine rich and transmembrane domain containing 1	-0.263	0.959	0.041

564389540	MGC116197 (includes others)	similar to RIKEN cDNA 1700001E04	-0.260	0.987	0.013
12018276	HPSE	heparanase	-0.249	0.985	0.015
6978435	ACADVL	acyl-CoA dehydrogenase very long chain	-0.249	0.974	0.026
157819311	LRGUK	leucine rich repeats and guanylate kinase domain containing	-0.248	0.993	0.007
6978485	ALAS2	5'-aminolevulinate synthase 2	-0.248	0.962	0.038
27754155	CLIP4	CAP-Gly domain containing linker protein family member 4	-0.246	0.995	0.005
15805026	ZFAND6	zinc finger AN1-type containing 6	-0.245	0.983	0.017
62543513	PTGR2	prostaglandin reductase 2	-0.244	0.963	0.037
56090433	GLT8D1	glycosyltransferase 8 domain containing 1	-0.243	0.973	0.027
187937143	C2orf42	chromosome 2 open reading frame 42	-0.241	0.975	0.025
149033803	CDKL2	cyclin dependent kinase like 2	-0.237	0.995	0.005
158749602	TRAM1L1	translocation associated membrane protein 1 like 1	-0.237	0.998	0.002
672047003	CDAN1	codanin 1	-0.236	0.956	0.044
157817839	SEMA5A	semaphorin 5A	-0.230	0.986	0.014
13994225	HSD17B10	hydroxysteroid 17-beta dehydrogenase 10	-0.229	0.976	0.024
564397761	GCC2	GRIP and coiled-coil domain containing 2	-0.229	0.977	0.023
564389730	PLAT	plasminogen activator, tissue type	-0.228	0.984	0.016
672050038	NDNF	neuron derived neurotrophic factor	-0.226	0.964	0.036
205277356	TVP23B	trans-golgi network vesicle protein 23 homolog B	-0.226	0.953	0.047
564383995	EVC	EvC ciliary complex subunit 1	-0.225	0.956	0.044
25742576	NXF1	nuclear RNA export factor 1	-0.224	0.994	0.006
672040941	ATRNL1	attractin like 1	-0.223	0.978	0.022
157820311	OTUD6B	OTU deubiquitinase 6B	-0.222	0.984	0.016
158711736	SMC2	structural maintenance of chromosomes 2	-0.221	0.971	0.029
148695758	CAPRIN1	cell cycle associated protein 1	-0.221	0.958	0.042
9506469	CD47	CD47 molecule	-0.218	0.955	0.045
157821513	HEBP1	heme binding protein 1	-0.215	0.986	0.014
148687519	CALN1	calneuron 1	-0.214	0.990	0.010
8393643	KCNAB1	potassium voltage-gated channel subfamily A member regulatory beta subunit 1	-0.214	0.965	0.035
564398053	MAN1A1	mannosidase alpha class 1A member 1	-0.213	0.958	0.042
57526927	LARS1	leucyl-tRNA synthetase 1	-0.213	0.972	0.028

392338550	IPO11	importin 11	-0.212	0.992	0.008
415703079	NEBL	nebulette	-0.211	0.960	0.040
158635998	SLC40A1	solute carrier family 40 member 1	-0.211	0.996	0.004
164414419	SP1	Sp1 transcription factor	-0.209	0.979	0.021
148747414	GDA	guanine deaminase	-0.203	0.976	0.024
68163417	FAHD1	fumarylacetoacetate hydrolase domain containing 1	-0.201	0.979	0.021
148664829	NMRAL1	NmrA like redox sensor 1	-0.200	0.963	0.037
46485382	BHLHB9	basic helix-loop-helix family member b9	-0.200	0.978	0.022
26006243	KCND2	potassium voltage-gated channel subfamily D member 2	-0.197	0.972	0.028
11560055	KHDRBS3	KH RNA binding domain containing, signal transduction associated 3	-0.195	0.959	0.041
564334053	SORCS1	sortilin related VPS10 domain containing receptor 1	-0.195	0.958	0.042
157818397	MFSD4A	major facilitator superfamily domain containing 4A	-0.194	0.988	0.012
11072106	NUCB2	nucleobindin 2	-0.193	0.950	0.050
283046651	PTPRZ1	protein tyrosine phosphatase receptor type Z1	-0.193	0.972	0.028
157822043	PLGRKT	plasminogen receptor with a C-terminal lysine	-0.191	1.000	0.000
25282457	CCNB1	cyclin B1	-0.191	0.972	0.028
148696094	TUBGCP4	tubulin gamma complex associated protein 4	-0.189	0.952	0.048
157822625	USP28	ubiquitin specific peptidase 28	-0.188	0.951	0.049
564399352	TAF9B	TATA-box binding protein associated factor 9b	-0.187	0.985	0.015
38051886	RABGGTB	Rab geranylgeranyltransferase subunit beta	-0.185	0.995	0.005
157820421	SMIM17	small integral membrane protein 17	-0.183	0.977	0.023
157817420	NRIP3	nuclear receptor interacting protein 3	-0.183	1.000	0.000
56605704	SERINC3	serine incorporator 3	-0.182	0.996	0.004
71037403	MYL12B	myosin light chain 12B	-0.181	0.991	0.009
32185285	BCL2L2	BCL2 like 2	-0.181	0.953	0.047
39930507	KCNK15	potassium two pore domain channel subfamily K member 15	-0.180	0.971	0.029
156139151	PDS5B	PDS5 cohesin associated factor B	-0.177	0.960	0.040
564395567	NFATC3	nuclear factor of activated T cells 3	-0.173	0.983	0.017
166091519	GRHPR	glyoxylate and hydroxypyruvate reductase	-0.172	0.985	0.015
396941666	Dync1i2	dynein cytoplasmic 1 intermediate chain 2	-0.169	0.982	0.018

148668175	EDNRB	endothelin receptor type B	-0.168	1.000	0.000
186910267	LYRM2	LYR motif containing 2	-0.167	0.976	0.024
19173766	LONP1	lon peptidase 1, mitochondrial	-0.164	0.967	0.033
12018278	ILKAP	ILK associated serine/threonine phosphatase	-0.163	0.996	0.004
6981542	SLC16A1	solute carrier family 16 member 1	-0.163	0.980	0.020
40786447	CFDP1	craniofacial development protein 1	-0.160	0.994	0.006
16758808	EPB41L3	erythrocyte membrane protein band 4.1 like 3	-0.159	0.975	0.025
52345385	PDIA6	protein disulfide isomerase family A member 6	-0.159	0.971	0.029
387157884	INO80	INO80 complex ATPase subunit	-0.158	0.977	0.023
18777747	OGA	O-GlcNAcase	-0.158	0.970	0.030
6649914	GDF11	growth differentiation factor 11	-0.154	0.969	0.031
149020656	MRE11	MRE11 homolog, double strand break repair nuclease	-0.152	0.971	0.029
71043650	SRPK1	SRSF protein kinase 1	-0.150	0.982	0.018
118150676	CCNA2	cyclin A2	-0.149	0.956	0.044
157819977	CERS4	ceramide synthase 4	-0.149	0.968	0.032
109505096	NID1	nidogen 1	-0.148	0.981	0.019
564390319	GKAP1	G kinase anchoring protein 1	-0.147	0.978	0.022
48976085	GM2A	GM2 ganglioside activator	-0.147	0.981	0.019
71043702	TM9SF4	transmembrane 9 superfamily member 4	-0.145	0.952	0.048
148672025	MAP3K12	mitogen-activated protein kinase kinase kinase 12	-0.144	0.986	0.014
109472884	UBE3C	ubiquitin protein ligase E3C	-0.142	0.968	0.032
157823986	MAN1A2	mannosidase alpha class 1A member 2	-0.142	0.992	0.008
6756037	YWHAH	tyrosine 3-monooxygenase/tryptophan 5-monooxygenase activation protein eta	-0.142	0.978	0.022
149065077	FAM3C	FAM3 metabolism regulating signaling molecule C	-0.141	0.979	0.021
16758736	NLGN1	neuroligin 1	-0.139	0.964	0.036
6978751	CYP51A1	cytochrome P450 family 51 subfamily A member 1	-0.138	0.951	0.049
62079259	CEP83	centrosomal protein 83	-0.135	0.955	0.045
672044529	MIGA1	mitoguardin 1	-0.135	0.952	0.048
157823181	SKP2	S-phase kinase associated protein 2	-0.134	0.998	0.002
158749632	DBT	dihydrolipoamide branched chain transacylase E2	-0.129	0.999	0.001

158711729	HACE1	HECT domain and ankyrin repeat containing E3 ubiquitin protein ligase 1	-0.128	0.963	0.037
398650648	SLC8A1	solute carrier family 8 member A1	-0.127	0.980	0.020
37359832	SCRN1	secernin 1	-0.127	0.965	0.035
149035673	FAF1	Fas associated factor 1	-0.126	1.000	0.000
114052913	CADM2	cell adhesion molecule 2	-0.126	0.955	0.045
564398462	Slc9a7	solute carrier family 9 member A7	-0.125	0.990	0.010
564339312	FUBP1	far upstream element binding protein 1	-0.123	0.997	0.003
166158339	REEP3	receptor accessory protein 3	-0.121	0.988	0.012
401709975	GPR176	G protein-coupled receptor 176	-0.121	0.999	0.001
209870013	ITSN1	intersectin 1	-0.121	0.961	0.039
27229314	FIBP	FGF1 intracellular binding protein	-0.118	0.953	0.047
71896549	UTP14A	UTP14A small subunit processome component	-0.118	0.975	0.025
398303839	SH3GL2	SH3 domain containing GRB2 like 2, endophilin A1	-0.117	0.966	0.034
52138635	ETFDH	electron transfer flavoprotein dehydrogenase	-0.115	0.994	0.006
6978621	CCNG1	cyclin G1	-0.114	0.978	0.022
78126149	SDF4	stromal cell derived factor 4	-0.113	0.998	0.002
404247435	YLPM1	YLP motif containing 1	-0.113	0.959	0.041
157786602	NHP2	NHP2 ribonucleoprotein	-0.110	0.975	0.025
57164133	NDUFC2	NADH:ubiquinone oxidoreductase subunit C2	-0.108	0.999	0.001
274326692	UQCC3	ubiquinol-cytochrome c reductase complex assembly factor 3	-0.106	0.977	0.023
58865384	NDUFS2	NADH:ubiquinone oxidoreductase core subunit S2	-0.106	0.956	0.044
157816887	LOC500028/Yae1	YAE1 maturation factor of ABCE1	-0.105	0.950	0.050
672065125	ADAM23	ADAM metallopeptidase domain 23	-0.104	0.996	0.004
67846036	DNPEP	aspartyl aminopeptidase	-0.101	0.996	0.004
55741823	TARS1	threonyl-tRNA synthetase 1	-0.101	0.976	0.024
58865936	SIKE1	suppressor of IKBKE 1	-0.100	0.984	0.016
281427192	CDH11	cadherin 11	-0.096	0.994	0.006
41152510	PLPPR1	phospholipid phosphatase related 1	-0.096	0.979	0.021
672057488	CD63	CD63 molecule	-0.096	0.994	0.006
148700731	ELMO1	engulfment and cell motility 1	-0.092	0.990	0.010
71682358	HIPK3	homeodomain interacting protein kinase 3	-0.092	0.977	0.023
40363268	WASHC2A/WASHC2C	WASH complex subunit 2A	-0.092	0.958	0.042

157820113	RANBP1	RAN binding protein 1	-0.092	0.985	0.015
148682688	CDK14	cyclin dependent kinase 14	-0.090	0.968	0.032
274326531	HSF1	heat shock transcription factor 1	-0.090	0.974	0.026
18093100	PLCD4	phospholipase C delta 4	-0.089	0.978	0.022
530362302	KPNA6	karyopherin subunit alpha 6	-0.087	0.961	0.039
157822747	CLVS1	clavesin 1	-0.085	0.993	0.007
149022387	NCKAP1	NCK associated protein 1	-0.084	0.994	0.006
50054266	NLN	neurolysin	-0.082	0.978	0.022
149060735	SENP5	SUMO specific peptidase 5	-0.077	0.983	0.017
148747528	PTK2B	protein tyrosine kinase 2 beta	-0.066	0.962	0.038
20302113	STIP1	stress induced phosphoprotein 1	-0.060	0.960	0.040
149049470	TPI1	triosephosphate isomerase 1	-0.057	0.994	0.006
16758168	FGF13	fibroblast growth factor 13	-0.049	0.998	0.002
20302061	ATP5PO	ATP synthase peripheral stalk subunit OSCP	-0.040	0.981	0.019
82830420	CTSB	cathepsin B	-0.040	0.966	0.034
157819063	SRPK2	SRSF protein kinase 2	-0.022	0.952	0.048
157819459	MRPS2	mitochondrial ribosomal protein S2	-0.011	0.970	0.030
392333100	FAM193A	family with sequence similarity 193 member A	0.027	-0.968	0.032
12004970	RNF11	ring finger protein 11	0.033	-0.966	0.034
162135934	TPST1	tyrosylprotein sulfotransferase 1	0.044	-0.969	0.031
300797122	FRMD4A	FERM domain containing 4A	0.051	-0.966	0.034
76443681	USP11	ubiquitin specific peptidase 11	0.053	-0.967	0.033
300798222	SIAH3	siah E3 ubiquitin protein ligase family member 3	0.054	-0.972	0.028
157816927	GMEB1	glucocorticoid modulatory element binding protein 1	0.063	-0.965	0.035
149039803	UBQLN1	ubiquilin 1	0.065	-0.968	0.032
157822779	DNAJC11	DnaJ heat shock protein family (Hsp40) member C11	0.066	-0.991	0.009
37360568	RANGAP1	Ran GTPase activating protein 1	0.068	-0.959	0.041
764020110	HNRNPUL2	heterogeneous nuclear ribonucleoprotein U like 2	0.070	-0.988	0.012
672044697	EHMT1	euchromatic histone lysine methyltransferase 1	0.070	-0.987	0.013
564353714	FBXO42	F-box protein 42	0.074	-0.963	0.037
564326269	RPL28	ribosomal protein L28	0.075	-1.000	0.000
157817783	SNX18	sorting nexin 18	0.079	-0.971	0.029
68341979	PLEKHO1	pleckstrin homology domain containing O1	0.080	-0.950	0.050
157817696	PIN1	peptidylprolyl cis/trans isomerase, NIMA-interacting 1	0.083	-0.986	0.014
148747227	SV2A	synaptic vesicle glycoprotein 2A	0.084	-0.979	0.021

214010118	TMEM59	transmembrane protein 59	0.085	-0.969	0.031
6978449	ADD2	adducin 2	0.085	-0.993	0.007
290560659	ZNF609	zinc finger protein 609	0.090	-0.969	0.031
564363529	NCAM1	neural cell adhesion molecule 1	0.091	-0.952	0.048
149038024	RIPOR1	RHO family interacting cell polarization regulator 1	0.091	-0.954	0.046
149016466	NIPBL	NIPBL cohesin loading factor	0.092	-0.989	0.011
161760661	KCNK9	potassium two pore domain channel subfamily K member 9	0.095	-0.973	0.027
14389301	SMPD2	sphingomyelin phosphodiesterase 2	0.096	-0.995	0.005
291084664	TRAPPC10	trafficking protein particle complex 10	0.099	-0.985	0.015
157818285	C12orf49	chromosome 12 open reading frame 49	0.099	-0.967	0.033
347921120	SLC23A2	solute carrier family 23 member 2	0.101	-0.955	0.045
71043624	AZI2	5-azacytidine induced 2	0.101	-0.975	0.025
46391106	ARL10	ADP ribosylation factor like GTPase 10	0.105	-0.960	0.040
149040413	GPAM	glycerol-3-phosphate acyltransferase, mitochondrial	0.107	-0.962	0.038
158631164	IGSF3	immunoglobulin superfamily member 3	0.110	-0.989	0.011
672048159	RAE1	ribonucleic acid export 1	0.112	-0.975	0.025
564309116	RTL6	retrotransposon Gag like 6	0.115	-0.969	0.031
51948396	TUSC3	tumor suppressor candidate 3	0.116	-1.000	0.000
755502295	SAMD10	sterile alpha motif domain containing 10	0.117	-0.954	0.046
274321371	CRLF3	cytokine receptor like factor 3	0.118	-0.993	0.007
18959272	KCNQ2	potassium voltage-gated channel subfamily Q member 2	0.119	-0.969	0.031
109510888	FAM155B	family with sequence similarity 155 member B	0.121	-0.991	0.009
672045179	MAPKAP1	MAPK associated protein 1	0.122	-0.976	0.024
114326177	SHMT1	serine hydroxymethyltransferase 1	0.123	-0.966	0.034
157818629	HEYL	hes related family bHLH transcription factor with YRPW motif like	0.126	-1.000	0.000
16758194	RGS2	regulator of G protein signaling 2	0.127	-0.963	0.037
672068548	SUPT6H	SPT6 homolog, histone chaperone and transcription elongation factor	0.127	-0.971	0.029
564363852	SNUPN	snurportin 1	0.131	-0.967	0.033
300797157	TBC1D8	TBC1 domain family member 8	0.131	-1.000	0.000
157824037	USP4	ubiquitin specific peptidase 4	0.131	-0.996	0.004
77404168	WDR77	WD repeat domain 77	0.138	-0.980	0.020
158081739	B4GALT1	beta-1,4-galactosyltransferase 1	0.140	-0.953	0.047

19924073	TTL	tubulin tyrosine ligase	0.142	-0.964	0.036
56090235	UBAC1	UBA domain containing 1	0.142	-0.989	0.011
40385881	ACVR1B	activin A receptor type 1B	0.143	-0.995	0.005
74139306	TMED9	transmembrane p24 trafficking protein 9	0.143	-0.982	0.018
564370907	ZNF598	zinc finger protein 598, E3 ubiquitin ligase	0.144	-0.972	0.028
157818521	COG8	component of oligomeric golgi complex 8	0.146	-0.990	0.010
13928926	MYBBP1A	MYB binding protein 1a	0.146	-0.976	0.024
564321656	TCF25	transcription factor 25	0.149	-0.951	0.049
209863130	SEMA3F	semaphorin 3F	0.155	-0.968	0.032
50510427	IP6K1	inositol hexakisphosphate kinase 1	0.156	-0.969	0.031
224593264	BORCS8	BLOC-1 related complex subunit 8	0.156	-0.986	0.014
77917548	DUS3L	dihydrouridine synthase 3 like	0.158	-0.977	0.023
6978805	EMD	emerin	0.159	-0.981	0.019
672043520	PI4KB	phosphatidylinositol 4-kinase beta	0.159	-0.995	0.005
6981442	PTPN1	protein tyrosine phosphatase non-receptor type 1	0.160	-0.955	0.045
58219518	RND2	Rho family GTPase 2	0.161	-0.993	0.007
672067460	RGD1560464	similar to hypothetical protein FLJ38426	0.164	-0.951	0.049
60359854	POLDIP3	DNA polymerase delta interacting protein 3	0.164	-1.000	0.000
300798499	AFF3	AF4/FMR2 family member 3	0.164	-0.954	0.046
197927315	YJU2	YJU2 splicing factor homolog	0.164	-0.990	0.010
564340867	MMADHC	metabolism of cobalamin associated D	0.165	-0.952	0.048
164663909	SDE2	SDE2 telomere maintenance homolog	0.165	-0.959	0.041
293359997	SGPP1	sphingosine-1-phosphate phosphatase 1	0.170	-0.979	0.021
157818643	KCTD3	potassium channel tetramerization domain containing 3	0.170	-0.989	0.011
149067744	Znf48	zinc finger protein 48	0.170	-0.962	0.038
157823565	COQ10A	coenzyme Q10A	0.174	-0.963	0.037
37360004	KDM1A	lysine demethylase 1A	0.177	-0.956	0.044
149044006	TEDC1	tubulin epsilon and delta complex 1	0.179	-0.999	0.001
28972780	TLE3	TLE family member 3, transcriptional corepressor	0.180	-0.959	0.041
392333209	DLG5	discs large MAGUK scaffold protein 5	0.180	-0.993	0.007
405113028	TAF3	TATA-box binding protein associated factor 3	0.181	-0.955	0.045

762006019	FAM8A1	family with sequence similarity 8 member A1	0.185	-0.984	0.016
60360108	BRD2	bromodomain containing 2	0.187	-0.991	0.009
300796253	ALDH1L2	aldehyde dehydrogenase 1 family member L2	0.190	-0.966	0.034
157823447	MFHAS1	malignant fibrous histiocytoma amplified sequence 1	0.191	-0.967	0.033
564393080	WDR33	WD repeat domain 33	0.193	-0.992	0.008
392332910	TP53BP2	tumor protein p53 binding protein 2	0.194	-0.952	0.048
157823197	NDUFB7	NADH:ubiquinone oxidoreductase subunit B7	0.194	-0.981	0.019
157823639	PPP1R13B	protein phosphatase 1 regulatory subunit 13B	0.196	-0.959	0.041
157820969	SBNO2	strawberry notch homolog 2	0.197	-0.968	0.032
157820401	ABHD2	abhydrolase domain containing 2, acylglycerol lipase	0.197	-0.959	0.041
148674304	RPRD1B	regulation of nuclear pre-mRNA domain containing 1B	0.198	-0.964	0.036
347800639	GFER	growth factor, augmenter of liver regeneration	0.199	-0.965	0.035
57164107	NIPSNAP3A	nipsnap homolog 3A	0.199	-0.974	0.026
157817801	ADCK1	aarF domain containing kinase 1	0.199	-0.955	0.045
84781688	GAS8	growth arrest specific 8	0.202	-0.957	0.043
187469679	LDB1	LIM domain binding 1	0.205	-0.991	0.009
67078512	SNX15	sorting nexin 15	0.215	-0.955	0.045
84781638	KLHL25	kelch like family member 25	0.215	-0.998	0.002
157819363	ZNF282	zinc finger protein 282	0.215	-0.982	0.018
213688373	GADD45GIP1	GADD45G interacting protein 1	0.218	-0.950	0.050
765099233	LMNB2	lamin B2	0.219	-0.992	0.008
564355517	CMPK2	cytidine/uridine monophosphate kinase 2	0.222	-0.964	0.036
288541353	CMTM4	CKLF like MARVEL transmembrane domain containing 4	0.222	-0.996	0.004
568992461	DIP2B	disco interacting protein 2 homolog B	0.223	-0.964	0.036
117940043	MED22	mediator complex subunit 22	0.225	-0.997	0.003
157823503	PLPBP	pyridoxal phosphate binding protein	0.228	-0.961	0.039
270483881	CBFA2T2	CBFA2/RUNX1 partner transcriptional co-repressor 2	0.231	-0.956	0.044
62664711	DIPK1C	divergent protein kinase domain 1C	0.235	-0.983	0.017
77736608	XYLT2	xylosyltransferase 2	0.235	-0.986	0.014
40018556	NOB1	NIN1 (RPN12) binding protein 1 homolog	0.237	-0.955	0.045
564359486	TBC1D30	TBC1 domain family member 30	0.237	-0.998	0.002
162951835	CYTH1	cytohesin 1	0.237	-0.982	0.018

114145762	WDR83	WD repeat domain 83	0.238	-0.980	0.020
62078913	OAF	out at first homolog	0.242	-0.996	0.004
58865780	ZBTB17	zinc finger and BTB domain containing 17	0.243	-0.962	0.038
31415868	MAFB	MAF bZIP transcription factor B	0.246	-0.980	0.020
672055562	LBH	LBH regulator of WNT signaling pathway	0.250	-0.989	0.011
209529662	LOC100911166/Rpusd2	RNA pseudouridine synthase domain containing 2	0.252	-0.954	0.046
157823719	TRAIP	TRAF interacting protein	0.252	-0.988	0.012
158187529	LMX1A	LIM homeobox transcription factor 1 alpha	0.253	-0.978	0.022
209954792	PDCD2	programmed cell death 2	0.254	-0.988	0.012
157821283	C19orf47	chromosome 19 open reading frame 47	0.257	-0.977	0.023
564367958	SEMA4C	semaphorin 4C	0.258	-0.984	0.016
197382256	PHF12	PHD finger protein 12	0.263	-0.989	0.011
8393652	KCNJ2	potassium inwardly rectifying channel subfamily J member 2	0.267	-0.963	0.037
148696370	PANK2	pantothenate kinase 2	0.270	-0.984	0.016
157823125	MRPS30	mitochondrial ribosomal protein S30	0.276	-0.992	0.008
68341997	GPR160	G protein-coupled receptor 160	0.281	-0.969	0.031
32451765	FBXO10	F-box protein 10	0.283	-0.982	0.018
157821325	TWNK	twinkle mtDNA helicase	0.283	-0.969	0.031
672029178	CCSER2	coiled-coil serine rich protein 2	0.285	-0.996	0.004
76559919	N4BP3	NEDD4 binding protein 3	0.292	-0.960	0.040
166795897	PIMREG	PICALM interacting mitotic regulator	0.293	-0.993	0.007
564363988	ISLR2	immunoglobulin superfamily containing leucine rich repeat 2	0.293	-0.970	0.030
149024496	SPEN	spen family transcriptional repressor	0.293	-0.984	0.016
56789732	VSTM5	V-set and transmembrane domain containing 5	0.295	-0.976	0.024
157818273	CDC42EP4	CDC42 effector protein 4	0.297	-0.970	0.030
157822519	CBLN4	cerebellin 4 precursor	0.299	-0.966	0.034
564327642	CEP89	centrosomal protein 89	0.300	-0.991	0.009
157819365	TBC1D25	TBC1 domain family member 25	0.302	-0.989	0.011
300796434	FOXRED2	FAD dependent oxidoreductase domain containing 2	0.305	-0.990	0.010
56605628	SFT2D1	SFT2 domain containing 1	0.306	-0.958	0.042
62078701	UTP25	UTP25 small subunit processor component	0.308	-0.972	0.028
300797262	BRPF1	bromodomain and PHD finger containing 1	0.308	-0.952	0.048

56605776	TAF11	TATA-box binding protein associated factor 11	0.309	-0.987	0.013
148707009	HMGCS2	3-hydroxy-3-methylglutaryl-CoA synthase 2	0.315	-0.979	0.021
149051028	RNF144A	ring finger protein 144A	0.322	-0.990	0.010
41386747	ZC3H18	zinc finger CCCH-type containing 18	0.322	-0.966	0.034
157820119	LRRTM1	leucine rich repeat transmembrane neuronal 1	0.328	-0.976	0.024
582015198	CRY2	cryptochrome circadian regulator 2	0.332	-0.953	0.047
564307081	ATXN7L1	ataxin 7 like 1	0.339	-0.954	0.046
564350836	MELK	maternal embryonic leucine zipper kinase	0.341	-0.976	0.024
30017415	ITPKC	inositol-trisphosphate 3-kinase C	0.343	-0.957	0.043
564365330	CDC25A	cell division cycle 25A	0.348	-0.965	0.035
57528321	RIOK2	RIO kinase 2	0.360	-0.975	0.025
564344754	PMEPA1	prostate transmembrane protein, androgen induced 1	0.360	-0.950	0.050
62543527	TGIF1	TGFB induced factor homeobox 1	0.362	-0.977	0.023
213972545	MXD1	MAX dimerization protein 1	0.378	-0.996	0.004
568997192	PRDM15	PR/SET domain 15	0.383	-0.951	0.049
14388593	SPATA2	spermatogenesis associated 2	0.385	-0.988	0.012
8392855	ADCYAP1	adenylate cyclase activating polypeptide 1	0.397	-0.986	0.014
149025439	DICER1	dicer 1, ribonuclease III	0.398	-0.984	0.016
11560016	HTR1B	5-hydroxytryptamine receptor 1B	0.402	-0.978	0.022
61889068	MXI1	MAX interactor 1, dimerization protein	0.405	-0.964	0.036
157819301	ZNF777	zinc finger protein 777	0.406	-0.987	0.013
157821351	EXO1	exonuclease 1	0.420	-0.975	0.025
14861862	CRYGD	crystallin gamma D	0.421	-0.955	0.045
68163435	MBLAC1	metallo-beta-lactamase domain containing 1	0.423	-0.962	0.038
38454286	STIMATE-MUSTN1	STIMATE-MUSTN1 readthrough	0.425	-1.000	0.000
293348129	DACT1	dishevelled binding antagonist of beta catenin 1	0.433	-0.978	0.022
56090592	EMP2	epithelial membrane protein 2	0.436	-0.988	0.012
58865998	PCDHGB7	protocadherin gamma subfamily B, 7	0.443	-0.996	0.004
3676248	Prim1	DNA primase subunit 1	0.450	-0.976	0.024
149041559	BUD13	BUD13 homolog	0.456	-0.993	0.007
157818699	TSEN54	tRNA splicing endonuclease subunit 54	0.458	-0.990	0.010
255708448	KATNA1	katanin catalytic subunit A1	0.466	-0.998	0.002
672047066	CEP152	centrosomal protein 152	0.480	-0.974	0.026

148689230	MAPKAPK3	MAPK activated protein kinase 3	0.490	-0.987	0.013
89145411	SULT2B1	sulfotransferase family 2B member 1	0.524	-0.954	0.046
404434380	ZNF133	zinc finger protein 133	0.530	-0.991	0.009
474451689	RASEF	RAS and EF-hand domain containing	0.560	-0.953	0.047
13928942	PER2	period circadian regulator 2	0.562	-0.995	0.005
62640766	GDPGP1	GDP-D-glucose phosphorylase 1	0.562	-0.972	0.028
148692940	WAPL	WAPL cohesin release factor	0.573	-0.985	0.015
74186677	SIN3B	SIN3 transcription regulator family member B	0.578	-0.987	0.013
13994119	KHK	ketohexokinase	0.612	-1.000	0.000
22122541	LRRC3B	leucine rich repeat containing 3B	0.615	-0.969	0.031
157822359	PELI2	pellino E3 ubiquitin protein ligase family member 2	0.620	-0.954	0.046
157820433	CPEB1	cytoplasmic polyadenylation element binding protein 1	0.624	-0.991	0.009
564303706	OSBPL3	oxysterol binding protein like 3	0.637	-0.971	0.029
149025186	RPS6KL1	ribosomal protein S6 kinase like 1	0.661	-0.978	0.022
80861398	CRY1	cryptochrome circadian regulator 1	0.720	-0.987	0.013
38454200	CHDH	choline dehydrogenase	0.734	-0.995	0.005
293339965	RAB11FIP3	RAB11 family interacting protein 3	0.750	-0.984	0.016
149025439	DICER1	dicer 1, ribonuclease III	0.846	-0.996	0.004
672058654	CAPS2	calcyphosine 2	0.874	-0.980	0.020
672025117	MBTD1	mbt domain containing 1	0.886	-0.951	0.049
71795623	ELMO3	engulfment and cell motility 3	0.910	-0.978	0.022
157821687	NEURL2	neuralized E3 ubiquitin protein ligase 2	0.966	-0.994	0.006
209571573	ZNF707	zinc finger protein 707	0.966	-0.964	0.036
1438906	NPY5R	neuropeptide Y receptor Y5	1.000	-0.965	0.035
86129546	ZDHHC22	zinc finger DHHC-type palmitoyltransferase 22	1.056	-0.970	0.030
19424314	KCNE2	potassium voltage-gated channel subfamily E regulatory subunit 2	1.066	-0.966	0.034
16758572	DLK1	delta like non-canonical Notch ligand 1	1.086	-0.972	0.028
149042270	LAS1L	LAS1 like ribosome biogenesis factor	1.169	-0.993	0.007
62078917	PAQR5	progestin and adipoQ receptor family member 5	1.181	-0.954	0.046
6978493	ALOX5	arachidonate 5-lipoxygenase	1.193	-0.991	0.009
157787081	WNT1	Wnt family member 1	1.222	-0.995	0.005
564385630	ACOX2	acyl-CoA oxidase 2	1.222	-0.969	0.031
569012000	KLF8	Kruppel like factor 8	1.406	-0.999	0.001
6981312	OTC	ornithine carbamoyltransferase	1.415	-0.970	0.030
19173800	Actn3	actinin alpha 3	1.415	-0.994	0.006
270133003	H2-M5	histocompatibility 2, M region locus 5	1.429	-0.969	0.031

157817264	ANKRD23	ankyrin repeat domain 23	1.505	-0.974	0.026
74004170	H2AC17	H2A clustered histone 17	1.549	-0.970	0.030
157819799	IQCH	IQ motif containing H	1.716	-0.983	0.017
576080555	GAPDH	glyceraldehyde-3-phosphate dehydrogenase	1.720	-0.978	0.022
157787012	SLAMF9	SLAM family member 9	1.841	-0.953	0.047
149042883	LOC100365365	rCG32328-like	1.861	-0.977	0.023
672070295	BAHCC1	BAH domain and coiled-coil containing 1	2.083	-0.955	0.045
564329926	EMSY	EMSY transcriptional repressor, BRCA2 interacting	2.094	-0.959	0.041
564312627	ZFP62	ZFP62 zinc finger protein	2.139	-0.953	0.047
568941582	IQSEC1	IQ motif and Sec7 domain ArfGEF 1	2.308	-0.978	0.022
564324736	L3MBTL3	L3MBTL histone methyl-lysine binding protein 3	2.353	-0.995	0.005
157818163	POF1B	POF1B actin binding protein	2.392	-0.968	0.032
149034139	TMEM273	transmembrane protein 273	2.406	-0.992	0.008
50370130	PALLD	palladin, cytoskeletal associated protein	3.361	-0.963	0.037
569009290	TENM1	teneurin transmembrane protein 1	3.450	-0.978	0.022
564309734	IGSF9B	immunoglobulin superfamily member 9B	4.173	-0.963	0.037

Supplementary Table S19. The list of genes that are differentially expressed in the offspring hippocampus in response to prenatal BPA exposure that exhibited the changes in the expression levels correlated with the percentage of alternation of the rat offspring in the T-maze test. The transcriptome profiling data of DEGs in male and female rat offspring prenatally exposed to BPA (n = 6, male pups n = 3 and female pups n = 3, from independent litters) or the vehicle control (n = 6, male pups n = 3 and female pups n = 3, from independent litters) were obtained and used for the PTM analyses to identify DEGs that exhibited the changes in the expression levels correlated with the percentage of alternation of the rat offspring in the T-maze test.

ID	Symbol	Entrez Gene Name	log2(FC)	R values	P-values
392343022	ZNF157	zinc finger protein 157	0.034	-0.962	0.038
564311678	PLEKHM3	pleckstrin homology domain containing M3	-1.152	0.994	0.006
564312627	ZFP62	ZFP62 zinc finger protein	-1.253	0.981	0.019
564349125	TEAD4	TEA domain transcription factor 4	-0.967	1.000	0.000
24638442	RLN3	relaxin 3	-0.949	1.000	0.000
157819465	CLEC9A	C-type lectin domain containing 9A	-0.967	0.999	0.001
16758094	FABP4	fatty acid binding protein 4	-0.933	1.000	0.000
149020413	Zfp599	zinc finger protein 599	-1.342	0.956	0.044
47576123	Olr1387/Olr1388	olfactory receptor 1387	-0.893	1.000	0.000
157822605	OTOR	otoraplin	-0.893	1.000	0.000
672036437	ACP4	acid phosphatase 4	-0.862	1.000	0.000
564343851	BPIFB1	BPI fold containing family B member 1	-0.807	1.000	0.000
53791211	PHOX2A	paired like homeobox 2A	-0.807	1.000	0.000
16758272	CPN1	carboxypeptidase N subunit 1	-0.807	1.000	0.000
124486586	AUTS2	activator of transcription and developmental regulator AUTS2	-0.941	0.988	0.012
300797609	ELOVL7	ELOVL fatty acid elongase 7	7.451	-0.975	0.025
392342139	TTC21A	tetratricopeptide repeat domain 21A	2.389	-0.968	0.032
392333013	CEP135	centrosomal protein 135	4.858	-0.993	0.007
402747041	FAM217A	family with sequence similarity 217 member A	3.954	-0.978	0.022
672084625	LOC100909409 (includes others)	RGD1562660	1.407	-0.961	0.039
148685413	ATP2A1	ATPase sarcoplasmic/endoplasmic reticulum Ca2+ transporting 1	2.000	-0.985	0.015
158508517	SDS	serine dehydratase	2.202	-0.971	0.029
11024668	AIPL1	aryl hydrocarbon receptor interacting protein like 1	2.188	-0.972	0.028
293340128	MIEF2	mitochondrial elongation factor 2	3.426	-0.998	0.002
157820271	LOXL4	lysyl oxidase like 4	2.585	-0.963	0.037
672078236	Gucy1b2	guanylate cyclase 1, soluble, beta 2	3.322	-0.958	0.042

112984288	STEAP4	STEAP4 metalloreductase	3.248	-0.977	0.023
392351087	HAGHL	hydroxyacylglutathione hydrolase like	4.120	-0.979	0.021
564329920	EMSY	EMSY transcriptional repressor, BRCA2 interacting	3.112	-0.976	0.024
157823427	KLHL31	kelch like family member 31	2.000	-0.980	0.020
58866012	TRIM55	tripartite motif containing 55	1.644	-1.000	0.000
11560026	STC2	stanniocalcin 2	1.585	-1.000	0.000
300794644	FREM3	FRAS1 related extracellular matrix 3	2.087	-0.987	0.013
106879208	MYH4	myosin heavy chain 4	1.415	-0.977	0.023
672014266	TMEM219	transmembrane protein 219	0.735	-0.961	0.039
672056787	PRIMA1	proline rich membrane anchor 1	1.234	-0.961	0.039
157822485	H2BC15	H2B clustered histone 15	1.265	-1.000	0.000
564374250	NAGS	N-acetylglutamate synthase	2.000	-0.966	0.034
13277927	RPLP0	ribosomal protein lateral stalk subunit P0	1.431	-0.978	0.022
293347270	OSGIN2	oxidative stress induced growth inhibitor family member 2	0.861	-0.977	0.023
56606104	Aox4	aldehyde oxidase 4	0.807	-1.000	0.000
6978663	CLCN1	chloride voltage-gated channel 1	0.742	-0.954	0.046
149042882	ZNF334	zinc finger protein 334	0.779	-0.968	0.032
755566692	HUWE1	HECT, UBA and WWE domain containing E3 ubiquitin protein ligase 1	0.787	-0.978	0.022
197387536	TEX26	testis expressed 26	0.706	-0.965	0.035
157786962	NANOS3	nanos C2HC-type zinc finger 3	0.596	-1.000	0.000
564298396	ZNF764	zinc finger protein 764	0.777	-0.978	0.022
16924020	XPNPEP2	X-prolyl aminopeptidase 2	0.585	-1.000	0.000
564303143	KMT2C*	lysine methyltransferase 2C	0.593	-0.968	0.032
564320454	SAP130*	Sin3A associated protein 130	0.559	-0.992	0.008
197386987	HDX	highly divergent homeobox	0.620	-0.984	0.016
281306771	ADAMTS4	ADAM metallopeptidase with thrombospondin type 1 motif 4	0.489	-0.979	0.021
157819737	SARS2	seryl-tRNA synthetase 2, mitochondrial	0.463	-0.972	0.028
67078462	SOX18	SRY-box transcription factor 18	0.460	-0.988	0.012
672043573	LOC100363520	mCG16729-like	0.643	-0.954	0.046
52851389	OSMR	oncostatin M receptor	0.570	-0.971	0.029
56090421	PXYLP1	2-phosphoxylose phosphatase 1	0.429	-0.991	0.009
171846573	FBXL4	F-box and leucine rich repeat protein 4	0.393	-0.992	0.008
126722629	HSPBAP1	HSPB1 associated protein 1	0.460	-0.970	0.030
157817797	PDCD2L	programmed cell death 2 like	0.468	-0.958	0.042
76096340	ANKRD16	ankyrin repeat domain 16	0.354	-0.964	0.036

149048116	KHDC4	KH domain containing 4, pre-mRNA splicing factor	0.314	-0.987	0.013
6978497	AMBP	alpha-1-microglobulin/bikunin precursor	0.308	-1.000	0.000
13928740	RGN	regucalcin	0.303	-1.000	0.000
164518930	SDK1	sidekick cell adhesion molecule 1	0.347	-0.974	0.026
347921954	Lilrb2	leukocyte immunoglobulin like receptor B2	0.290	-1.000	0.000
157817592	HEXIM2	HEXIM P-TEFb complex subunit 2	0.366	-0.957	0.043
157819887	LACTB	lactamase beta	0.314	-0.985	0.015
31543579	RELN	reelin	0.268	-0.994	0.006
157816943	MCM8	minichromosome maintenance 8 homologous recombination repair factor	0.316	-0.963	0.037
148683687	RHBDL3	rhomboid like 3	0.301	-0.983	0.017
157819581	SESN2	sestrin 2	0.267	-0.994	0.006
564320608	SEMA6A	semaphorin 6A	0.214	-0.998	0.002
109470195	TNKS1BP1	tankyrase 1 binding protein 1	0.213	-0.986	0.014
157819811	C21orf91	chromosome 21 open reading frame 91	0.275	-0.990	0.010
672036088	KMT2B	lysine methyltransferase 2B	0.253	-0.987	0.013
149062169	MEN1	menin 1	0.236	-0.994	0.006
564303143	KMT2C*	lysine methyltransferase 2C	0.176	-0.953	0.047
11560052	DUSP12	dual specificity phosphatase 12	0.234	-0.959	0.041
672023059	TLN2*	talin 2	0.240	-0.957	0.043
392355126	HAUS2	HAUS augmin like complex subunit 2	0.175	-0.979	0.021
157823683	HDDC2	HD domain containing 2	0.189	-0.992	0.008
270483881	CBFA2T2	CBFA2/RUNX1 partner transcriptional co-repressor 2	0.231	-0.953	0.047
300797915	Rbm33	RNA binding motif protein 33	0.166	-0.969	0.031
6981572	SP4	Sp4 transcription factor	0.169	-0.981	0.019
11968114	MRPL23	mitochondrial ribosomal protein L23	0.127	-0.953	0.047
149044495	CAAP1	caspase activity and apoptosis inhibitor 1	0.172	-0.964	0.036
300797562	BCOR	BCL6 corepressor	0.183	-0.975	0.025
392332910	TP53BP2	tumor protein p53 binding protein 2	0.194	-0.958	0.042
300796412	ATMIN	ATM interactor	0.160	-0.979	0.021
41386755	FGFR1OP2	FGFR1 oncogene partner 2	0.148	-0.995	0.005
41053837	GPX3	glutathione peroxidase 3	0.159	-0.987	0.013
37360236	SMG5	SMG5 nonsense mediated mRNA decay factor	0.169	-0.969	0.031
57528294	NEPRO	nucleolus and neural progenitor protein	0.169	-0.960	0.040
62656582	KIAA0100	KIAA0100	0.130	-0.990	0.010

157818167	PDPR	pyruvate dehydrogenase phosphatase regulatory subunit	0.160	-0.975	0.025
274325505	Pwp2	PWP2 periodic tryptophan protein homolog (yeast)	0.115	-0.989	0.011
157818061	2510002D24Rik	RIKEN cDNA 2510002D24 gene	0.120	-0.992	0.008
148491097	DYNC1H1	dynein cytoplasmic 1 heavy chain 1	0.137	-0.991	0.009
164663909	SDE2	SDE2 telomere maintenance homolog	0.165	-0.950	0.050
61556860	MRPL46	mitochondrial ribosomal protein L46	0.120	-1.000	0.000
392333209	DLG5	discs large MAGUK scaffold protein 5	0.146	-0.979	0.021
564398269	SCML4	Scm polycomb group protein like 4	0.135	-0.986	0.014
81158095	PCDHGA3	protocadherin gamma subfamily A, 3	0.118	-0.996	0.004
157820897	MTHFD2	methylenetetrahydrofolate dehydrogenase (NADP+ dependent) 2, methenyltetrahydrofolate cyclohydrolase	0.141	-0.964	0.036
564388185	ERCC6	ERCC excision repair 6, chromatin remodeling factor	0.112	-0.965	0.035
148673403	GRSF1	G-rich RNA sequence binding factor 1	0.108	-0.973	0.027
13162349	ASIC1	acid sensing ion channel subunit 1	0.082	-0.998	0.002
347921120	SLC23A2	solute carrier family 23 member 2	0.101	-0.954	0.046
89363040	PCDHGA11	protocadherin gamma subfamily A, 11	0.075	-0.997	0.003
157786974	Wdr83os	WD repeat domain 83 opposite strand	0.067	-0.998	0.002
564356550	PCNX1	pecanex 1	0.070	-0.971	0.029
18644718	RGS3	regulator of G protein signaling 3	0.057	-0.994	0.006
149049696	MKRN2	makorin ring finger protein 2	0.052	-0.960	0.040
9506875	SMAD4	SMAD family member 4	0.047	-0.966	0.034
157821407	FBXO28	F-box protein 28	0.039	-0.959	0.041
672046314	AMBRA1	autophagy and beclin 1 regulator 1	0.034	-0.972	0.028
157819701	Ctla2a	cytotoxic T lymphocyte-associated protein 2 alpha	-0.966	0.953	0.047
74178753	DENND2D	DENN domain containing 2D	-0.778	1.000	0.000
61556945	MOAP1	modulator of apoptosis 1	-0.795	0.999	0.001
672022227	RGS22	regulator of G protein signaling 22	-0.855	0.972	0.028
62821825	OPALIN	oligodendrocytic myelin paranodal and inner loop protein	-0.678	1.000	0.000
293349343	MYO6	myosin VI	-0.755	0.983	0.017
8394221	Rps3a1	ribosomal protein S3A1	-0.630	1.000	0.000
62078563	CD302	CD302 molecule	-0.613	0.996	0.004
760997729	SYNPO2L	synaptopodin 2 like	-0.621	0.977	0.023
568927637	ADAMTSL1	ADAMTS like 1	-0.716	0.966	0.034
672076564	DDC	dopa decarboxylase	-0.632	0.984	0.016

11067395	Tcam1	testicular cell adhesion molecule 1	-0.516	0.961	0.039
16758778	EFNA5	ephrin A5	-0.372	0.973	0.027
188497675	RADX	RPA1 related single stranded DNA binding protein, X-linked	-0.542	0.979	0.021
77020250	PCSK9	proprotein convertase subtilisin/kexin type 9	-0.485	1.000	0.000
293355224	Rps12-ps24	ribosomal protein S12, pseudogene 24	-0.509	0.990	0.010
564346692	GIMAP8	GTPase, IMAP family member 8	-0.455	0.985	0.015
469663646	NDUFA13	NADH:ubiquinone oxidoreductase subunit A13	-0.433	0.999	0.001
392331598	MPV17L*	MPV17 mitochondrial inner membrane protein like	-0.549	0.963	0.037
6978629	CD38	CD38 molecule	-0.440	0.968	0.032
148709823	PCGF5	polycomb group ring finger 5	-0.460	0.976	0.024
213512607	CLYBL	citramalyl-CoA lyase	-0.365	0.979	0.021
40352944	NXT2	nuclear transport factor 2 like export factor 2	-0.302	0.990	0.010
157821925	IFT88	intraflagellar transport 88	-0.320	0.953	0.047
12621142	RASSF9	Ras association domain family member 9	-0.299	0.959	0.041
564302385	SHLD1	shieldin complex subunit 1	-0.289	0.986	0.014
62078973	MIF4GD	MIF4G domain containing	-0.253	0.995	0.005
62078935	FLACC1	flagellum associated containing coiled-coil domains 1	-0.233	0.966	0.034
564319191	MCPH1	microcephalin 1	-0.284	0.969	0.031
564320452	SAP130*	Sin3A associated protein 130	-0.253	0.988	0.012
52138628	RAP1B	RAP1B, member of RAS oncogene family	-0.267	0.955	0.045
564332776	LRRN4CL	LRRN4 C-terminal like	-0.193	1.000	0.000
157819089	EOLA1/EOLA2	endothelium and lymphocyte associated ASCH domain 1	-0.213	0.991	0.009
9506425	BET1	Bet1 golgi vesicular membrane trafficking protein	-0.215	0.964	0.036
62543563	KYAT3	kynurenine aminotransferase 3	-0.233	0.971	0.029
51948390	HSD17B11	hydroxysteroid 17-beta dehydrogenase 11	-0.213	0.961	0.039
188536087	RAMAC	RNA guanine-7 methyltransferase activating subunit	-0.201	0.969	0.031
112984202	FZD8	frizzled class receptor 8	-0.183	0.969	0.031
18266704	TRPC5	transient receptor potential cation channel subfamily C member 5	-0.206	0.974	0.026
401664552	MRPS7	mitochondrial ribosomal protein S7	-0.159	0.994	0.006
149016262	Col4a4	collagen type IV alpha 4 chain	-0.195	0.957	0.043

346989661	CPEB2	cytoplasmic polyadenylation element binding protein 2	-0.162	0.997	0.003
12838537	C19orf81	chromosome 19 open reading frame 81	-0.156	0.991	0.009
50356003	SCP2	sterol carrier protein 2	-0.170	0.978	0.022
189027115	AIDA	axin interactor, dorsalization associated	-0.140	0.996	0.004
157820049	LRFN5	leucine rich repeat and fibronectin type III domain containing 5	-0.161	0.981	0.019
149057384	C15orf40	chromosome 15 open reading frame 40	-0.163	0.967	0.033
149040047	SYNPR	synaptoporin	-0.167	0.958	0.042
51948478	FARSB	phenylalanyl-tRNA synthetase subunit beta	-0.138	0.985	0.015
40254752	PGK1	phosphoglycerate kinase 1	-0.146	0.980	0.020
6980978	GPD2	glycerol-3-phosphate dehydrogenase 2	-0.146	0.964	0.036
341823648	RAPH1	Ras association (RalGDS/AF-6) and pleckstrin homology domains 1	-0.103	0.998	0.002
148707802	DARS1	aspartyl-tRNA synthetase 1	-0.103	0.990	0.010
81884516	Rhno1	RAD9-HUS1-RAD1 interacting nuclear orphan 1	-0.107	0.952	0.048
56605990	LRPPRC	leucine rich pentatricopeptide repeat containing	-0.115	0.984	0.016
164519053	FAM131B	family with sequence similarity 131 member B	-0.101	0.978	0.022
564355419	Nbas	NBAS subunit of NRZ tethering complex	-0.110	0.950	0.050
58865384	NDUFS2	NADH:ubiquinone oxidoreductase core subunit S2	-0.106	0.953	0.047
157818729	SPATA33	spermatogenesis associated 33	-0.065	0.988	0.012
300794036	TMEM185B	transmembrane protein 185B	-0.056	0.999	0.001
157817861	NDUFA2	NADH:ubiquinone oxidoreductase subunit A2	-0.057	0.985	0.015
186910247	MRPS21	mitochondrial ribosomal protein S21	-0.059	0.994	0.006
71795619	SLC19A2	solute carrier family 19 member 2	-0.052	0.994	0.006
83320121	RBM8A	RNA binding motif protein 8A	-0.052	0.967	0.033
24638208	EXOC2	exocyst complex component 2	-0.041	0.997	0.003
18426824	KHDRBS1	KH RNA binding domain containing, signal transduction associated 1	-0.047	0.987	0.013
60678266	ENPP5	ectonucleotide pyrophosphatase/phosphodiesterase family member 5	-0.049	0.975	0.025
402743461	DPY19L3	dpy-19 like C-mannosyltransferase 3	-0.046	0.986	0.014

157823867	TLL1	tolloid like 1	-0.044	0.954	0.046

Supplementary Table S20. The characteristics of neonatal rat pups used for RNA-seq and qRT-PCR analyses.

Analysis	No.	Treatment	Birth Weight (g)	Sex	Number of Littermates			Milk band
					Total	Male	Female	
RNA-seq	1	BPA	5.8	Male	11	4	7	Yes
	2	BPA	6	Male	10	3	7	Yes
	3	BPA	5.5	Male	13	4	9	Yes
	4	BPA	5.6	Female	11	4	7	Yes
	5	BPA	5.3	Female	11	4	7	Yes
	6	BPA	6.3	Female	10	3	7	Yes
	7	Control	5.2	Male	12	9	3	Yes
	8	Control	7	Male	12	3	9	Yes
	9	Control	7.6	Male	13	4	9	Yes
	10	Control	5.1	Female	12	9	3	Yes
	11	Control	7	Female	12	3	9	Yes
	12	Control	6.6	Female	13	4	9	Yes
qRT-PCR	1	BPA	5.6	Male	11	4	7	Yes
	2	BPA	5.4	Male	11	4	7	Yes
	3	BPA	6.4	Male	11	4	7	Yes
	4	BPA	5.2	Female	13	4	9	Yes
	5	BPA	5.8	Female	11	4	7	Yes
	6	BPA	6.3	Female	10	3	7	Yes
	7	BPA	5.8	Male	11	4	7	Yes
	8	BPA	6	Male	10	3	7	Yes
	9	BPA	5.5	Male	13	4	9	Yes
	10	BPA	5.6	Female	11	4	7	Yes
	11	BPA	5.3	Female	11	4	7	Yes
	12	BPA	6.3	Female	10	3	7	Yes
	13	Control	5.9	Male	10	7	3	Yes
	14	Control	7.3	Male	12	6	6	Yes
	15	Control	6.3	Male	10	7	3	Yes
	16	Control	6.5	Female	12	6	6	Yes
	17	Control	5.4	Female	11	2	9	Yes
	18	Control	5	Female	10	7	3	Yes
	19	Control	5.2	Male	12	9	3	Yes
	20	Control	7	Male	12	3	9	Yes
	21	Control	7.6	Male	13	4	9	Yes
	22	Control	5.1	Female	12	9	3	Yes
	23	Control	7	Female	12	3	9	Yes
	24	Control	6.6	Female	13	4	9	Yes

Supplementary Table S21. List of primers for qRT-PCR analysis.

Gene	Forward primer (5'-> 3')	Reverse primer (5'-> 3')
Mief2	GCTCGTTTGGTGCTAGGTGT	CTCAGCAGGCTCAGTTCCTT
Eif3h	TAAGCAGCAGCAGCAGAAAC	GGGCTTGAAGAGTTTGGAGA
Tp53bp1	ATAGCCTTGAGGAGCAACGA	GCTCCAGGAAGTTCTGCTGT
Npas3	GGATGCAGAAGAATGGAGGT	ATGTCCATGGGTGTGTCCTT
Cux1	GGATATGAAGCGGATGGAGA	TTCTTCAGCTGGTGTTGTGG
Kdm5c	CTTGCAGCAGAACACTTGGA	CAAATTCTCCTGCACACTGG
Arhgap32	CCCTCCGCTCAGCTAAAAGT	TCCATTGAAGGAGGCAGAGA
Itga4	GTGACCCCAACTTCGTTTGT	AGCCATGCTAATGCCAGTGT
Atp8a1	CAGGAGCTCGAAGCAAAATC	GGAGCAGGTTCTGTTGTAAGG
Kmt2a	CTAAGGAGGCGGTTGGTGT	CTTCTCACGCTTGTCCGTCT
Cadps2	ATCTGGCCGACACCTACATT	TGATGAGCGTCTTCAGTTGG
Grm4	CCATGTACACCACCTGCATT	ACTGAAGCGCTCAGACTCAC
Abca7	AGCGCTCAGCATCTCAAAAG	AGCTCAGCATCAGGGAATGT
Rn18s	CTGGATACCGCAGCTAGGAA	GAATTTCACCTCTAGCGGCG

Supplementary Table S22. Compositions of the media used for primary hippocampal cell culture in this study

**Dissecting medium**	**Volume**
1X HBSS (Invitrogen, USA)	300 ml
Glucose (100 g/l) (Sigma-Aldrich, USA)	14 ml
2 mM HEPES (GE Healthcare Bio-Sciences, USA)	630 µl
$NaHCO_3$ (Sigma-Aldrich, USA)	7 ml

**Maintenance medium (Total volume 40 ml)**	**Volume**
Neurobasal medium (1X), no phenol red (Invitrogen, USA)	38.4 ml
B-27 serum-free supplement (Invitrogen, USA)	800 µl
200 mM L-glutamine (100X) (Invitrogen, USA)	400 µl
Antibiotic-Antimycotic (100X) (Invitrogen, USA)	400 µl

**Plating medium (Total volume 40 ml)**	**Volume**
MEM Eagle's with Earle's BSS (GE Healthcare Bio-Sciences, USA)	34.6 ml
Fetal bovine serum (Sigma-Aldrich, USA)	4 ml
100 mM sodium pyruvate (100X) (Sigma-Aldrich, USA)	400 µl
Glucose (100 g/l) (Sigma-Aldrich, USA)	180 µl
200 mM L-glutamine (100X) (Invitrogen, USA)	400 µl
Antibiotic-Antimycotic (100X) (Invitrogen, USA)	400 µl
